# Identification of Latent Oncogenes with a Network Embedding Method and Random Forest

**DOI:** 10.1155/2020/5160396

**Published:** 2020-09-23

**Authors:** Ran Zhao, Bin Hu, Lei Chen, Bo Zhou

**Affiliations:** ^1^College of Information Engineering, Shanghai Maritime University, Shanghai 201306, China; ^2^State Key Laboratory of Livestock and Poultry Breeding, Guangdong Public Laboratory of Animal Breeding and Nutrition, Guangdong Provincial Key Laboratory of Animal Breeding and Nutrition, Institute of Animal Science, Guangdong Academy of Agricultural Sciences, Guangzhou 510640, China; ^3^Shanghai University of Medicine & Health Sciences, Shanghai 201318, China

## Abstract

Oncogene is a special type of genes, which can promote the tumor initiation. Good study on oncogenes is helpful for understanding the cause of cancers. Experimental techniques in early time are quite popular in detecting oncogenes. However, their defects become more and more evident in recent years, such as high cost and long time. The newly proposed computational methods provide an alternative way to study oncogenes, which can provide useful clues for further investigations on candidate genes. Considering the limitations of some previous computational methods, such as lack of learning procedures and terming genes as individual subjects, a novel computational method was proposed in this study. The method adopted the features derived from multiple protein networks, viewing proteins in a system level. A classic machine learning algorithm, random forest, was applied on these features to capture the essential characteristic of oncogenes, thereby building the prediction model. All genes except validated oncogenes were ranked with a measurement yielded by the prediction model. Top genes were quite different from potential oncogenes discovered by previous methods, and they can be confirmed to become novel oncogenes. It was indicated that the newly identified genes can be essential supplements for previous results.

## 1. Introduction

Cancer is the second cause of human deaths in the world, following the cardiovascular disease. Lots of people directly died from cancer per year [1]. Although several efforts have been made in recent years, the mechanism of cancers has not been fully uncovered, which makes difficulties in designing effective treatments. Genetic background and environmental factors are widely accepted to be major causes of cancers [2]. Investigation on the mechanism of cancers with related genes is an essential way to understand the tumor initiation and development.

Oncogene is an important type of cancer-related genes, which can promote the tumor initiation. Thus, it is essential to identify latent oncogenes as much as possible, promoting the understanding of cancers. In early time, experimental techniques performed on typical cell lines or animal models are the main way for detecting oncogenes. However, this way is time-consuming and with high cost. In recent years, with the development of computer science, this procedure can be improved aided by designing computational methods. The computational methods can give a deep insight into a large-scale data and learn hidden associations between cancers and genes, thereby making useful clues and providing latent candidates. Experimenters can do targeted tests to confirm the results. Two pioneer studies have been proposed in this regard recently. The first study proposed a network method for inferring novel oncogenes based on validated oncogenes reported in some online databases [3]. The method applied the shortest path (SP) algorithm on a protein-protein interaction (PPI) network to extract the shortest paths connecting any two proteins of oncogenes. Proteins lying on these paths were picked up and screened by three measurements. 37 possible oncogenes were obtained by this method. The second study investigated oncogenes in a quite different way [4]. It tried to uncover the functions, including Gene Ontology (GO) terms and biological pathways, of oncogenes with machine learning algorithms. They first extracted essential GOs and pathways that can indicate the differences of oncogenes and other general genes and made prediction with them. More than 800 genes were predicted to be novel oncogenes. All of the above two studies proposed some putative oncogenes; some of which were extensively discussed. However, the limitations also exit. For the network method proposed in the first study [3], it did not contain a learning procedure, indicated that it cannot capture the essential features of oncogenes, inducing several false positive oncogenes. Although the second method [4] contained a learning procedure, it did not include the protein association information. As proteins with strong associations always share common functions, the protein association information is powerful materials for discovering novel oncogenes.

In view of the limitations of the above two studies, this study proposed a new computational method. The protein networks, derived from protein associations, were constructed, from which informative features were extracted to represent genes. The classic machine learning algorithm, random forest (RF) [5], was adopted to capture essential features of oncogenes and build the model. The latent oncogenes were ranked by a measurement yielded by the proposed method. Top latent oncogenes were quite different from those reported in previous two studies. We also analyzed some top latent oncogenes to confirm their likelihood of being oncogenes.

## 2. Materials and Methods

### 2.1. Materials

Validated oncogenes were directly downloaded from a previous study [3], which were collected from HUGO Gene Nomenclature Committee (HGNC, https://www.genenames.org/) [6] and Gene Set Enrichment Analysis Molecular Signatures Database (GSEA MSigDB, https://www.broadinstitute.org/gsea/msigdb/gene_families.jsp) [7, 8]. From HGNC, 251 oncogenes were collected and 330 oncogenes were retrieved from GSEA MSigDB. 543 oncogenes were obtained after combining above two sets of oncogenes. Because we used protein-protein interaction (PPI) networks to identify latent oncogenes, where proteins were represented by Ensembl IDs, proteins encoded by these 543 oncogenes were extracted and they were further mapped onto their Ensembl IDs. After excluding Ensembl IDs that were not in the PPI networks, we finally accessed 481 Ensembl IDs. With these IDs, we designed a computational method to identify new possible IDs. These new IDs suggested latent oncogenes.

### 2.2. Protein-Protein Interaction and Network Construction

In recent years, it is quite popular to adopt networks for investigating various diseases [3, 9–14]. Networks can organize data and information in a system level. It is beneficial to study different problems in a more complete view. Thus, we employed PPI networks to identify novel oncogenes in present study.

In this study, we adopted the PPI data reported in STRING (https://string-db.org/, Version 10.0) [15, 16], a well-known public database collecting known and predicted PPIs. Currently, 9,643,763 proteins from 2,031 organisms comprise huge numbers of PPIs, which were collected from a variety of sources, such as genomic background prediction, high-throughput laboratory experiments, (conservative) coexpression, automated textualization, and prior knowledge in databases. Thus, each interaction contains the physical and functional associations of two proteins and can widely measure the linkage between proteins. Compared with the PPIs reported in other databases, such as DIP (Database of Interaction Proteins) database [17] and BioGRID [18] which only include experimentally validated PPIs, PPIs in STRING contain more information and are more helpful for building models with a complete view. For human, 4,274,001 PPIs are reported in STRING, covering 19,247 human proteins. Each PPI consists of two proteins, encoded by Ensembl IDs. Further, STRING evaluates the strength of each PPI from eight different aspects and assigns eight scores to each PPI, titled by “Neighborhood”, “Fusion”, “Cooccurrence”, “Coexpression”, “Experiment”, “Database”, “Textmining”, and “Combined_score”. The last score combines other seven scores in a naive Bayesian fashion [16]. It was not adopted in the present study because it may produce redundancy with other seven scores. For each of the other scores, one PPI network was constructed in the following manner. We took the “Neighborhood” score as an example. First, 19,247 proteins were picked up as nodes. Second, two nodes were adjacent if and only if the “Neighborhood” score between corresponding proteins was larger than zero. Finally, such “Neighborhood” score was assigned to the corresponding edge as its weight. Accordingly, seven PPI networks were constructed, which were denoted by *N*_*N*_, *N*_*F*_, *N*_CO_, *N*_CE_, *N*_*E*_, *N*_*D*_, and *N*_*T*_, respectively. The sizes (numbers of edges) of seven networks were quite different. *N*_*T*_ had most edges (3816497), followed by *N*_*E*_ (1736931 edges), *N*_CE_ (768962 edges), *N*_*D*_ (212430 edges), *N*_*N*_ (76214 edges), *N*_CO_ (23739 edges), and *N*_*F*_ (2060 edges).

### 2.3. Feature Engineering

Network is excellent to organize the associations of the proteins, which can view a specific protein in a system level. However, there is a gap between it and the traditional machine learning algorithms because these algorithms always need numerical vectors as input. Fortunately, some network embedding algorithms, such as Mashup [19], Node2vec [20], and Deepwalk [21], were proposed in recent years, which can abstract relationship in the network and output a feature vector for each node in the network. The occurrence of these algorithms connects the network and the traditional machine learning algorithm. Considering the fact that seven networks were involved in this study, Mashup [19] was adopted. It can tackle multiple networks, which is the greatest merit compared with other network embedding algorithms. Its brief descriptions were as follows.

The procedures of Mashup encoding each node consist of two stages. In the first stage, it applies the random walk with the restart (RWR) algorithm [22–26] on each network to construct a raw feature vector for each node in this network. In detail, for a network *N*_*j*_ (*j* ∈ {*N*, *F*, *CO*, *CE*, *E*, *D*, *T*}) constructed in Section 2.2, each node *p*_*i*_ in this network was picked up as the node seed of the RWR algorithm one by one. When the RWR algorithm stopped, each node in this network was assigned a probability, indicating its associations to the seed node. A raw feature vector of *p*_*i*_ was built by collecting all these probabilities, which was denoted by *V*_*j*_^*i*^. Because some nodes can occur in multiple networks, several feature vectors were generated for these nodes. It is necessary to fuse them into one feature vector in a rigorous way. On the other hand, a dimensionality reduction procedure is also necessary due to the large dimension of the raw feature vectors. All these are the purpose of the second stage of Mashup. Let *X*^*i*^ be the final vector of protein *p*_*i*_ and *W*_*j*_^*i*^ be a context feature vector of *p*_*i*_ in the network *N*_*j*_. It is clear that Mashup tries to determine the optimal components in *X*^*i*^ and *W*_*j*_^*i*^, which retain the essential information in *V*_*j*_^*i*^ as much as possible. Based on *X*^*i*^ and *W*_*j*_^*i*^, a vector, denoted by ${\tilde{V}}_{j}^{i}$, can be constructed. Its components were defined as follows:

(1) $$\begin{array}{c} {\tilde{V}}_{jk}^{i}=\frac{\exp\left({\left(X^{i}\right)}^{T}W_{j}^{k}\right)}{\sum_{k\prime }\exp\left({\left(X^{i}\right)}^{T}W_{j}^{k\prime }\right)},k=1,2,\cdots ,n \end{array}$$

where *n* is the total number of different nodes (proteins) in seven networks. The following problem is to find out the optimal components in *X*^*i*^ and *W*_*j*_^*i*^, which can generate ${\tilde{V}}_{j}^{i}$ approximating *V*_*j*_^*i*^ as much as possible. An optimization problem is set up to determine the optimal components, which is formulated as below:

(2) $$\begin{array}{c} \underset{X^{i},W_{j}^{i}}{\mathrm{minimize}}\frac{1}{n}\overset{m}{\underset{j=1}{\sum }}\overset{n}{\underset{i=1}{\sum }}D_{KL}\left(V_{j}^{i}\left\|{\tilde{V}}_{j}^{i}\right.\right), \end{array}$$

where *m* stands for the total number of networks and *D*_*KL*_(•) stands for the function of KL-divergence (relative entropy).

The present study used the Mashup program obtained from http://cb.csail.mit.edu/cb/mashup/. The dimension of the output vector is a main parameter of the Mashup. Several dimensions, varying from 100 to 1000, were tried in this study because we did not know which dimension was best.

### 2.4. Random Forest

The network embedding algorithm, Mashup, connects the networks and traditional machine learning algorithms. With the feature vectors extracted from seven protein networks via Mashup, a specific machine learning algorithm can deeply study the characteristic of vectors of oncogenes, thereby building a prediction model. This study selected the classic machine learning algorithm, RF [5], due to its wide applications in bioinformatics and medical informatics [27–34].

RF is an ensemble classification algorithm, which consists of several decision trees. Given a dataset with *n* samples and *m* features, RF constructs each decision tree in the following manner. Randomly selected *n* samples, with replacement, from the original dataset. A decision tree is constructed based on selected samples. When the tree is grown at a node, randomly select *m*′ features, where *m*′ is much smaller than *m*, and the optimal splitting way is determined based on these *m*′ features. For an input sample, each decision tree first makes its prediction. RF integrates these predictions by majority voting. Although, decision tree is a weak classifier, RF is deemed to be much strong and competitive compared with other advanced classification algorithms [35].

In this study, a tool “RandomForest” in Weka [36] was directly employed, which implements the above-mentioned RF. Default parameters were used, where the number of decision trees was set to ten.

### 2.5. The Proposed Method for Inferring Latent Oncogenes

Among the 19,247 proteins occurring in seven protein networks, 481 are encoded by validated oncogenes, whereas the rest 18,766 proteins have not been labelled. It is clear that some proteins may be encoded by latent oncogenes. The proposed method can discover novel latent oncogenes with the feature vectors obtained Section 2.3 and RF described in Section 2.4.

For 18,766 unlabelled proteins, the proposed method evaluated their likelihood of being oncogenes in the following manner. For technique reasons, these 18,766 unlabelled proteins were termed as negative samples, whereas 481 proteins of oncogenes were deemed as positive samples. Evidently, negative samples were much more than positive samples (about 39 times). Thus, we randomly divided the negative samples into 39 parts. Negative samples in each part were combined with positive samples to construct a dataset, thereby yielding 39 datasets. On each dataset, a prediction model was built with RF as the classification algorithm. Accordingly, 39 RF models were produced. For each unlabelled protein *p*, it was fed into 38 RF models, except the RF model containing it. Each model assigned a probability to *p*, suggesting its likelihood to be an oncogene. The mean of all its probabilities was finally calculated to fully measure the likelihood of it being an oncogene. For an easy description, such mean value was called level value.

After all 18,766 unlabelled proteins had been evaluated with the above procedures, we ranked them in a list with the decreasing order of their level values. Evidently, those with high level values were more likely to be oncogenes.

**Figure 1:**
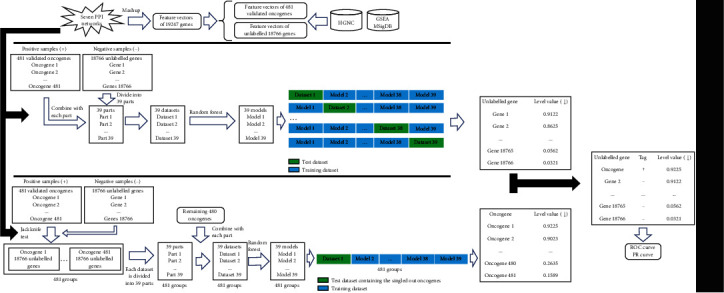
Flow chart to show the procedures of the method for inferring latent oncogenes. Seven protein-protein interaction (PPI) networks are constructed using the PPI information reported in STRING, from which feature vectors of oncogenes and unlabelled genes are extracted via Mashup. Unlabelled genes are divided into 39 parts; each of which combines the oncogenes to comprise a dataset. Each dataset induces a random forest (RF) model. A level value is computed for each unlabelled gene, which is the average of the probabilities yielded by 38 RF models. Unlabelled genes are ranked by the decreasing order of their level values. The same procedures are done for each oncogene, thereby ranking oncogenes. Finally, all genes are sorted in a list and ROC and PR curves are plotted to evaluate the performance of the method.

The entire procedures of the method are illustrated in Figure 1.

### 2.6. Performance Evaluation

To evaluate the utility of the proposed method, a procedure similar to the jackknife test [37–39] was adopted. Each of 481 proteins encoded by oncogenes was singled out one by one as unlabelled proteins. For a specific singled out protein, we want to know whether the rest 480 proteins of oncogenes can identify it. According to the procedures of the abovementioned method, 18,767 unlabelled proteins (one singled out protein and 18,766 actual unlabelled proteins) were also randomly divided into 39 parts. Each part and the positive sample set were combined to generate a dataset, thereby producing 39 datasets. Then, the same procedures of the methods followed to yield the level value of the singled out protein. After all proteins encoded by oncogenes had been tested, they were all assigned a level value. A protein list was created by ranking all 19,247 proteins, including proteins of oncogenes and unlabelled proteins, with the decreasing order of their level values. Some measurements can be calculated to evaluate such list, thereby indicating the utility of the method.

Given a protein list, which sorted proteins with decreasing order of their level values, whether a protein was predicted to be an oncogene (positive sample) was determined after a threshold of level value was set; that is, proteins with level values larger than the threshold were predicted to be oncogenes (positive samples); otherwise, they were predicted to be nononcogenes (negative samples). Accordingly, four values, true positive (TP), false negative (FN), false positive (FP) and true negative (TN), can be counted. Then, the sensitivity (SN) (same as recall), specificity (SP), and precision can be computed by

(3) $$\begin{array}{c} \text{SN}\left(\text{recall}\right)=\frac{\text{TP}}{\text{TP}+\text{FN}},\\ \text{SP}=\frac{\text{TN}}{\text{TN}+\text{FP}},\\ \text{Precision}=\frac{\text{TP}}{\text{TP}+\text{FP}}. \end{array}$$

After setting several thresholds, we can obtain a number of SNs, SPs, and precisions. A receiver operating characteristic (ROC) [40] curve and a precision-recall (PR) curve were plotted, where the ROC curve sets SN as *y*-axis and 1-SP as *x*-axis, whereas PR curve adopts precision as *y*-axis and recall as *x*-axis. Furthermore, the area under each of above two curves can be calculated, which were called AUROC and AUPRC, respectively, in this study. Evidently, the higher the area was, the better the method was.

## 3. Results and Discussion

### 3.1. Performance of the Method with Different Dimensions

**Figure 2:**
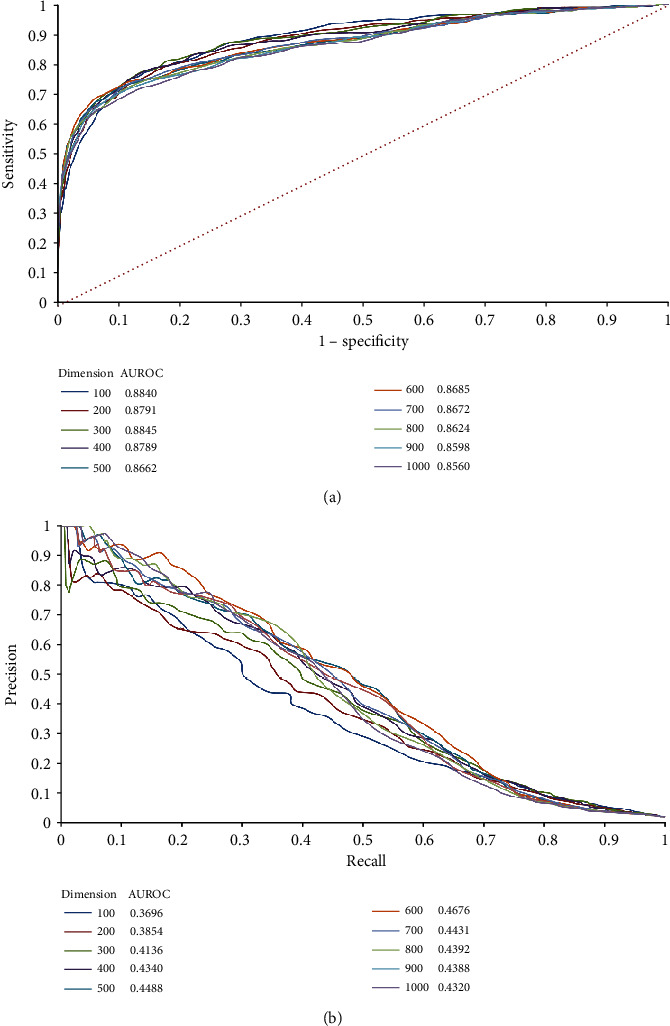
ROC and PR curves to show the performance of the method with different dimensions varying from 100 to 1000. (a) ROC curves, the dimension 300 yields the highest AUROC of 0.8845; (b) PR curves, the dimension 600 yields the highest AUPRC of 0.4676.

**Figure 3:**
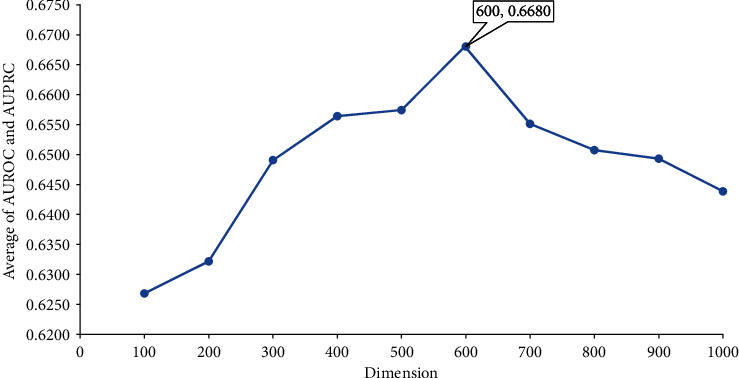
Scatter diagram to show the performance of the method with different dimensions varying from 100 to 1000. The *x*-axis represents dimension and the *y*-axis indicates the average of AUROC and AUPRC. The method with dimension 600 produces best performance.

In this study, we used features derived from seven protein networks. Several dimensions were tried to select the best one. For each dimension, the proposed method was evaluated in the way described in Section 2.6. A ROC curve and a PR curve was plotted, as shown in Figure 2. From Figure 2(a), the method with dimension 300 yielded the highest AUROC of 0.8845, whereas the method with dimension 600 gave the highest AUPRC of 0.4676 from Figure 2(b). In general, the PR curve is a more accurate measurement than the ROC curve if the dataset is greatly imbalanced. In our study, the negative samples were about 39 times as many as positive samples. Thus, we selected the method with dimension 600 as the proposed method. To further elaborate that this selection is reasonable, we calculated the average of AUROC and AUPRC for each dimension and plotted a scatter diagram to show these averages, as illustrated in Figure 3. Evidently, the dimension 600 gave the highest average of 0.6680, supporting the above selection. The trend of average on dimension shown in Figure 3 proved the reliability of the results. Before 600, the average showed an increasing trend, while it generally descended after 600. It is reasonable because when the dimension was small, several essential information cannot be included, where the dimension was large, lots of noisy was included. All these results supported the method that with dimension 600 was the best choice because it can recover actual oncogenes (positive samples) as much as possible. The unlabelled proteins predicted to be positive samples by this method were more reliable.

### 3.2. Inferred Oncogenes Obtained by the Proposed Method

**Figure 4:**
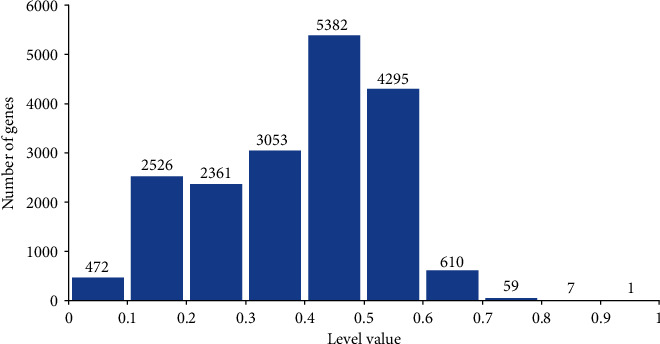
Distribution of level values on 18,766 unlabelled proteins. Only 677 unlabelled proteins (~3.61%) are assigned the level values higher than 0.6. These proteins are more likely to be encoded by latent oncogenes than others.

As mentioned in Section 3.1, we selected the method with dimension 600 as the proposed method. For each unlabelled protein, it was assigned a level value by the method to indicate its likelihood of being oncogenes. The level values of all 18,766 unlabelled proteins are provided in Supplementary Material S1.  Figure 4 shows the distribution of all level values. It can be observed that one unlabelled protein was assigned the level value larger than 0.9, seven proteins were with level values between 0.8 and 0.9. These proteins are more likely to be encoded by latent oncogenes. On the other hand, majority proteins (about 96.39%) received the level values smaller than 0.6.

### 3.3. Comparison of Previous Studies

Two previous computational methods have been proposed for identifying possible oncogenes. The one method adopted SP algorithm to search novel oncogenes in a PPI network; thus, this method was called the SP-based method. The other method investigated oncogenes from the point view of their functions; it was termed as function-based method in this study. These previous methods all yielded some latent oncogenes. A comparison was performed in this section.

**Figure 5:**
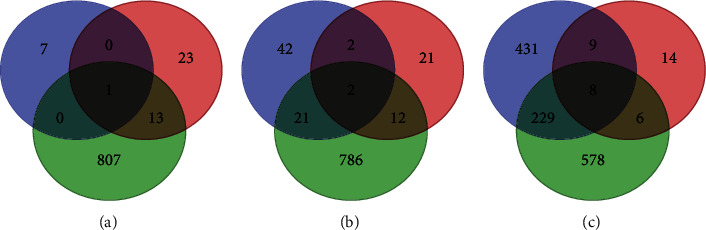
Venn diagrams to show the intersection of inferred oncogenes yielded by three methods. Blue, red, and green circles represent inferred oncogenes obtained by the proposed method, SP-based method, and function-based method, respectively. (a) Inferred oncogenes with level values larger than 0.8 are considered. (b) Inferred oncogenes with level values larger than 0.7 are considered. (c) Inferred oncogenes with level values larger than 0.6 are considered.

As our method only ranks the candidates with their level values, we set some thresholds to select inferred genes to make comparisons. The thresholds included 0.8, 0.7, and 0.6, yielding eight, 67 and 677 inferred oncogenes, respectively. The intersection of these inferred oncogene sets and two oncogene sets yielded by previous methods is illustrated in Figure 5. When the threshold was set to 0.8, only one gene (HOXA10) was also identified by the SP-based method. 25 inferred oncogenes were shared by either SP- or function-based methods when the threshold was 0.7, where two genes (HOXA10, AR) were inferred by all three methods. For the threshold 0.6, this number was 246, where eight genes (HOXA10, AR, ESR1, NOTCH3, PTPN6, MYO5A, KIAA0100, and MAP2K1) were shared by all methods. The exclusive oncogenes yielded by the proposed method occupied 87.5% when 0.8 was set as the threshold. Such percent was 62.69% and 63.66% for the thresholds 0.7 and 0.6, respectively. These results indicate that majority top inferred oncogenes of our method were not discovered by previous methods, indicating that our method can discover novel latent oncogenes that cannot be identified by previous methods.

### 3.4. Analysis of Top Inferred Oncogenes

In this study, some latent oncogenes were inferred by the proposed computational method. Each gene was assigned a level value to indicate its likelihood of being oncogenes.  Table 1 lists the top fifteen inferred oncogenes. This section selected four of them for detailed analysis.

#### 3.4.1. RAB31

Such gene is the top identification with level value 0.9105. RAB31 (Ras-related protein in brain 31), a member of the RAB family, encodes a protein belonging to the Ras superfamily of small GTPases. Because it was a significant homology with RAB22 (71% sequence identity), RAB31 was also named RAB22b. Similar to other members of the RAB family, it functions as molecular switches and plays critical roles in cell adhesion molecules and membrane trafficking of growth factor receptors [41]. Therefore, it is also conceivable that RAB31-mediated dysregulation in endocytosis or recirculation may result in failure to control cell proliferation, adhesion, and migration. As expected, its promotive effect on tumor progression has been reported in several types of cancers [42]. In breast cancer, it was confirmed to be overexpressed in patients with estrogen receptor (ER) positive breast cancer [43]. It is reported that high expression of RAB31 mRNA has a significant correlation with the poor prognosis of lymph node-negative breast cancer patients [44]. Further in vivo and in vitro experiments confirmed that the overexpression of RAB31 promoted cell proliferation of breast cancer cells [45]. Immunohistochemical staining revealed that the expression of RAB31 in liver cancer tissue was significantly higher than that in adjacent liver tissue. Overexpression of RAB31 in liver cancer tissue after hepatectomy is considered to be related to a poor prognosis [46]. In addition, it was found that RAB31 is associated with the survival of glioblastoma [47]. A recent study also confirmed that overexpression of RAB31 in gastric cancer tissues was significantly related to specific clinicopathological features and shorter survival time, strongly suggesting that RAB31 can be a new oncogene for gastric cancer [48].

#### 3.4.2. ACSL5

This gene received the level value of 0.8184. It encodes a specific transcription factor of the long-chain acyl-CoA synthetase (ACSL) family. In fatty acid metabolism, the first and essential step is the activation of fatty acids. ACSLs, responsible for activation of the most abundant long-chain fatty acids (12-20 carbons) in the diet into acyl-CoA thioesters, are generally deregulated in cancer. Such deregulation is also related to poor survival in patients with cancer [49]. The role of ACSL5 in cancer is quite complex. ACSL5 was reported to be downregulated in colorectal carcinomas [50, 51], breast cancer [52, 53], bladder cancer [53], and pancreas cancer [54]. Furthermore, ACSL5 lower regulation predicted a worse prognosis in breast cancer [52]. However, opposing results were also reported in studies on glioma [55] and gastric cancer [56], where ACSL5 was upregulated. In addition, fibroblast growth factor receptor 2 (FGFR2) -ACSL5 chimera RNA caused clinical gastric cancer cells to be resistant to the treatment with FGFR inhibitors [57]. Evidences showed that high levels of ACSL5, as a potential downstream target of the transcription factor ONECUT2 (OC2), together with OC2, may cooperatively promote intestinal metaplasia and gastric cancer progression [56]. These contradicting results indicated that the roles of ACSL5 were different among the different cancer types. Lastly, exon 20 skipped variant of ACSL5 protein (splice, Spl) was identified. Results showed that the growth inhibitory effect produced by the Spl protein was opposed to the growth-promoting activity of the nonsplice (NSpl) isoform [58]. Therefore, due to both isoforms, ACSL5 may act either as a tumor suppressor gene or an oncogene.

#### 3.4.3. WNT7B

This gene was assigned a level value of 0.8053. WNT7B is an extracelluar matrix protein of Wnt family protein [59]. The Wnt (Wingless-INT) was derived from the wingless gene related to visual mutations in Drosophila and the Int1 gene related to mouse breast cancer. Wnt signaling is a well-conserved pathway via canonical (*β*-catenin) and noncanonical (planar cell polarity and calcium) signaling [60]. WNT7B, as an activator of canonical Wnt/*β*-catenin signaling [61], plays a critical role in normal development and tumorigenesis [62]. Because Wnt protein was first isolated from mouse breast cancer, the role of WNT7B on breast cancer has also been increasingly reported. Huguet et al. [63] explored differential expression of human Wnt Genes 2, 3, 4, and 7B in human breast cell lines and normal and disease states of human breast tissue. They further found that in 10% of tumors WNT7B expression was 30-fold higher than in normal or benign breast tissues. In addition to confirming results consistent with them, Ojalvo et al. [64] and Chen et al. [65] further validated that WNT7B expression makes connections with markers of poor prognosis. Yeo et al. [59] built a Csf1r-icre mouse model using a WNT7B deletion, which also illustrated a critical role of myeloid WNT7B in breast cancer progression, including the levels of angiogenesis, invasion, and metastasis. In addition to breast cancer, abnormal expression of the WNT7b leads to the pathogenesis of many other cancers. Arensman et al. [66] confirmed that WNT7B expression was increased with high activity levels of autocrine Wnt/*β*-catenin signaling in pancreatic adenocarcinoma. Zheng et al. [67] found that expression of WNT7B is essential for the growth of prostate cancer cells and this effect is enhanced under androgen-deprived conditions. Their further analyses revealed that WNT7B promotes androgen-independent growth of CRPC cells likely via the activation of protein kinase C isozymes. Their results further showed that prostate cancer-produced WNT7B maked osteoblast differentiation in vitro and in vivo. As for osteosarcoma (OS) [68], WNT7B expression is dramatically upregulated in OS tissue samples and cells, especially in metastatic OS cell lines. Liu et al. [68] also found that WNT7B silence within OS cells remarkably inhibited the viability and invasion and enhanced the apoptosis of OS cells, suggesting that knocking down WNT7B could inhibit the OS cell growth. Therefore, we presume that WNT7B may function as an oncogene in carcinoma tissue types.

#### 3.4.4. FLT1

This gene was assigned a level value of 0.7763. Fms-related tyrosine kinase 1 (FLT1, also known as VEGFR-1) is a gene that encodes for a member of the VEGFR family, which presents a critical point in angiogenesis and subsequent cancer progression [69]. The expression of FLT1 is not limited to vascular endothelial cells. It is also found in cells of the hematopoietic lineage (i.e., monocytes and macrophages), dendritic cells, osteoclasts, pericytes, liver cells, placental trophoblasts [70], and smooth muscle cells [71], where it has a regulatory function. Therefore, with respect to carcinogenesis, the role of FLT1 may be more complex [72]. Recent reports also indicate that FLT1 is directly expressed on tumor cells from breast, colon, and skin origin. It is an important oncogenic driver in above cells, boosting survival, cell proliferation, and invasion in an independent manner [73–76]. In head and neck squamous cell carcinoma (HNSSC), FLT1 was selectively overexpressed in tumor tissue. FLT1 was further identified as an important oncogenic driver of HNSCC survival and resistance to radiotherapy with a shRNAmir-based dropout screening setup [72]. Qian et al. [77] found that FLT1 labels a subset of macrophages in human breast cancers which are significantly enriched in metastatic sites. Furthermore, using several genetic models, they elaborated that macrophage FLT1 is important for tumor cell seeding and persistent growth during the distal metastasis. Jiang et al. [78] identified that FLT1 promotes invasion and migration of glioblastoma cells through the modulation of sonic hedgehog (SHH) signaling pathway. A further study has indicated that FLT1 knockdown can prevent the spread of glioblastoma cells in vivo. FLT1 may be a novel oncogene, and therefore, inhibition of FLT1 may serve as a potential target for the development of therapies against metastatic events.

## 4. Conclusions

This study proposed a computational method for the identification of latent oncogenes. From seven protein networks, informative features of proteins were extracted via a powerful network embedding algorithm. Obtained features were learned by random forest, thereby setting up the prediction model. The principle of our method was quite different from previous methods and provided some novel latent oncogenes. Some inferred genes can be confirmed to be novel oncogenes, suggesting that the newly identified oncogenes can be essential supplements for previous studies. It is hoped that the new findings reported in this study can promote the research process of cancers.

## Figures and Tables

**Table 1:** Top fifteen inferred oncogenes.

Rank	Ensembl ID	Gene symbol	Description	Level value
1	ENSP00000304565	RAB31	RAB31, member RAS oncogene family	0.9105
2	ENSP00000421799	ENSG00000257184	—	0.8868
3	ENSP00000469872	RAB4B-EGLN2	RAB4B-EGLN2 Readthrough (NMD candidate)	0.8500
4	ENSP00000283921	HOXA10	Homeobox A10	0.8289
5	ENSP00000385586	HOXD12	Homeobox D12	0.8184
6	ENSP00000348429	ACSL5	Acyl-CoA synthetase long chain family member 5	0.8184
7	ENSP00000256953	RERG	RAS-like estrogen regulated growth inhibitor	0.8105
8	ENSP00000341032	WNT7B	Wnt family member 7B	0.8053
9	ENSP00000321805	RIT2	Ras like without CAAX 2	0.7763
10	ENSP00000285735	RHOC	Ras homolog family member C	0.7763
11	ENSP00000282397	FLT1	Fms related receptor tyrosine kinase 1	0.7763
12	ENSP00000264711	DNAJC27	DnaJ heat shock protein family (Hsp40) member C27	0.7737
13	ENSP00000339787	ACSL4	Acyl-CoA synthetase long chain family member 4	0.7737
14	ENSP00000357306	RIT1	Ras like without CAAX 1	0.7684
15	ENSP00000301068	RHEBL1	RHEB like 1	0.7684

## Data Availability

The validated oncogenes were collected from HUGO Gene Nomenclature Committee and Gene Set Enrichment Analysis Molecular Signatures Database.

## Supplementary Materials

Supplementary Materials Supplementary material S1 Level values of candidate oncogenes.
