# Supplementary material for: Identification of Latent Oncogenes with a Network Embedding Method and Random Forest
Source: Biomed Res Int. 2020 Sep 23;2020:5160396. doi: 10.1155/2020/5160396 (PMC7530476; doi:10.1155/2020/5160396)
Supplement: Supplementary Materials — Supplementary material S1 Level values of candidate oncogenes. [file 5160396.f1.pdf]

**Supplementary Material S1.** Level values of candidate oncogenes.

| Ensembl ID | Gene symbol | Level value |
|---|---|---|
| ENSP00000304565 | RAB31 | 0.9105 |
| ENSP00000421799 | ENSG00000257184 | 0.8868 |
| ENSP00000469872 | RAB4B-EGLN2 | 0.8500 |
| ENSP00000283921 | HOXA10 | 0.8289 |
| ENSP00000385586 | HOXD12 | 0.8184 |
| ENSP00000348429 | ACSL5 | 0.8184 |
| ENSP00000256953 | RERG | 0.8105 |
| ENSP00000341032 | WNT7B | 0.8053 |
| ENSP00000321805 | RIT2 | 0.7763 |
| ENSP00000285735 | RHOC | 0.7763 |
| ENSP00000282397 | FLT1 | 0.7763 |
| ENSP00000264711 | DNAJC27 | 0.7737 |
| ENSP00000339787 | ACSL4 | 0.7737 |
| ENSP00000357306 | RIT1 | 0.7684 |
| ENSP00000301068 | RHEBL1 | 0.7684 |
| ENSP00000267996 | TPM1 | 0.7632 |
| ENSP00000354219 | TPM2 | 0.7579 |
| ENSP00000249501 | HOXD10 | 0.7579 |
| ENSP00000249499 | HOXD9 | 0.7553 |
| ENSP00000292408 | FGFR4 | 0.7553 |
| ENSP00000358698 | WNT2B | 0.7500 |
| ENSP00000369375 | TEK | 0.7500 |
| ENSP00000313572 | NIM1 | 0.7500 |
| ENSP00000296870 | IL3 | 0.7474 |
| ENSP00000263253 | EP300 | 0.7447 |
| ENSP00000308576 | RHOD | 0.7395 |
| ENSP00000241463 | RASL11A | 0.7395 |
| ENSP00000224337 | BLNK | 0.7368 |
| ENSP00000272369 | MEIS1 | 0.7342 |
| ENSP00000222139 | EPOR | 0.7342 |
| ENSP00000310036 | CD34 | 0.7342 |
| ENSP00000258873 | ACSBG1 | 0.7342 |
| ENSP00000265441 | WNT2 | 0.7316 |
| ENSP00000325836 | DIRAS1 | 0.7316 |
| ENSP00000264818 | TYK2 | 0.7263 |
| ENSP00000302836 | HOXC9 | 0.7263 |
| ENSP00000262187 | RHEB | 0.7237 |
| ENSP00000337088 | MEN1 | 0.7237 |
| ENSP00000358162 | HIST2H4A | 0.7237 |
| ENSP00000239882 | ELF1 | 0.7237 |
| ENSP00000252669 | ACSBG2 | 0.7237 |
| ENSP00000264834 | KLF1 | 0.7184 |
| ENSP00000222462 | WNT16 | 0.7158 |
| ENSP00000295713 | SRGAP2 | 0.7158 |
| ENSP00000267205 | RHOF | 0.7158 |
| ENSP00000307321 | HOXC10 | 0.7158 |
| ENSP00000378702 | BRD2 | 0.7158 |
| ENSP00000331327 | WT1 | 0.7105 |
| ENSP00000471921 | SPIB | 0.7105 |
| ENSP00000309439 | HOXB9 | 0.7105 |

| | | |
|---|---|---|
| ENSP00000348258 | HIST1H4L | 0.7105 |
| ENSP00000339007 | GRB2 | 0.7105 |
| ENSP00000259089 | BLK | 0.7105 |
| ENSP00000347198 | SRGAP1 | 0.7079 |
| ENSP00000343477 | RUNX3 | 0.7079 |
| ENSP00000396259 | PAX2 | 0.7079 |
| ENSP00000363822 | AR | 0.7079 |
| ENSP00000215781 | OSM | 0.7053 |
| ENSP00000366974 | HIST1H4F | 0.7053 |
| ENSP00000262995 | GAB1 | 0.7053 |
| ENSP00000242159 | HOXA7 | 0.7026 |
| ENSP00000350159 | HIST1H4K | 0.7026 |
| ENSP00000265495 | ELF2 | 0.7026 |
| ENSP00000364919 | DIRAS2 | 0.7026 |
| ENSP00000263239 | DDX18 | 0.7026 |
| ENSP00000305422 | CEBPB | 0.7026 |
| ENSP00000394932 | CDKN2A | 0.7026 |
| ENSP00000310170 | FOSL1 | 0.7000 |
| ENSP00000338018 | HIF1A | 0.6974 |
| ENSP00000216101 | RASL10A | 0.6947 |
| ENSP00000312436 | NFE2 | 0.6947 |
| ENSP00000234091 | ID2 | 0.6947 |
| ENSP00000368632 | GATA3 | 0.6947 |
| ENSP00000220003 | CSK | 0.6947 |
| ENSP00000341208 | STAT5A | 0.6921 |
| ENSP00000419692 | RXRA | 0.6921 |
| ENSP00000360493 | RUNX2 | 0.6921 |
| ENSP00000466680 | RND2 | 0.6921 |
| ENSP00000314151 | KLK3 | 0.6921 |
| ENSP00000358153 | HIST2H4B | 0.6921 |
| ENSP00000365012 | HCK | 0.6921 |
| ENSP00000352673 | ELF3 | 0.6921 |
| ENSP00000264972 | ZAP70 | 0.6895 |
| ENSP00000290167 | WNT4 | 0.6895 |
| ENSP00000220507 | RHOV | 0.6895 |
| ENSP00000249071 | RAC2 | 0.6895 |
| ENSP00000226574 | NFKB1 | 0.6895 |
| ENSP00000356234 | KDM5B | 0.6895 |
| ENSP00000261937 | FLT4 | 0.6895 |
| ENSP00000380033 | DDX17 | 0.6895 |
| ENSP00000362195 | CSF3R | 0.6895 |
| ENSP00000155926 | TRIB2 | 0.6868 |
| ENSP00000320147 | EZH2 | 0.6868 |
| ENSP00000300134 | STAT6 | 0.6842 |
| ENSP00000364190 | PBX2 | 0.6842 |
| ENSP00000353624 | HIST1H4E | 0.6842 |
| ENSP00000226317 | CXCL6 | 0.6842 |
| ENSP00000256495 | BHLHE40 | 0.6842 |
| ENSP00000242261 | TWIST1 | 0.6816 |
| ENSP00000380921 | SH3KBP1 | 0.6816 |
| ENSP00000263895 | RND3 | 0.6816 |
| ENSP00000339467 | RHOG | 0.6816 |
| ENSP00000352336 | PLCG2 | 0.6816 |

| | | |
|---|---|---|
| ENSP00000229307 | NANOG | 0.6816 |
| ENSP00000353483 | MAPK8 | 0.6816 |
| ENSP00000290295 | HOXB13 | 0.6816 |
| ENSP00000311010 | ELF5 | 0.6816 |
| ENSP00000296027 | CXCL5 | 0.6816 |
| ENSP00000336790 | ATF4 | 0.6816 |
| ENSP00000281455 | ACSL1 | 0.6816 |
| ENSP00000369981 | SH3GL2 | 0.6789 |
| ENSP00000277120 | NTRK2 | 0.6789 |
| ENSP00000379330 | NFATC2 | 0.6789 |
| ENSP00000376309 | HNRNPA3 | 0.6789 |
| ENSP00000366581 | HIST1H4B | 0.6789 |
| ENSP00000271450 | FCGR2A | 0.6789 |
| ENSP00000248070 | EPS15L1 | 0.6789 |
| ENSP00000316338 | BAIAP2 | 0.6789 |
| ENSP00000362634 | TSSK3 | 0.6763 |
| ENSP00000274376 | RASA1 | 0.6763 |
| ENSP00000364163 | NOTCH4 | 0.6763 |
| ENSP00000357470 | IL6R | 0.6763 |
| ENSP00000350767 | HIST4H4 | 0.6763 |
| ENSP00000357615 | FRK | 0.6763 |
| ENSP00000384675 | SOS1 | 0.6737 |
| ENSP00000401303 | SHC1 | 0.6737 |
| ENSP00000220062 | RASL12 | 0.6737 |
| ENSP00000304283 | RAC3 | 0.6737 |
| ENSP00000228280 | KITLG | 0.6737 |
| ENSP00000343282 | HIST1H4D | 0.6737 |
| ENSP00000294702 | GFI1 | 0.6737 |
| ENSP00000272164 | WNT9A | 0.6711 |
| ENSP00000346440 | TCF4 | 0.6711 |
| ENSP00000346879 | NKX2-1 | 0.6711 |
| ENSP00000363689 | ID3 | 0.6711 |
| ENSP00000328928 | HOXB4 | 0.6711 |
| ENSP00000322898 | EBF1 | 0.6711 |
| ENSP00000297268 | COL1A2 | 0.6711 |
| ENSP00000384869 | CLHC1 | 0.6711 |
| ENSP00000339179 | TSSK4 | 0.6684 |
| ENSP00000375081 | TSSK1B | 0.6684 |
| ENSP00000370421 | PDX1 | 0.6684 |
| ENSP00000378485 | MATK | 0.6684 |
| ENSP00000364550 | KDM5C | 0.6684 |
| ENSP00000298229 | INPPL1 | 0.6684 |
| ENSP00000367034 | HIST1H4C | 0.6684 |
| ENSP00000354952 | GAB2 | 0.6684 |
| ENSP00000289902 | FCER1G | 0.6684 |
| ENSP00000343925 | ESR2 | 0.6684 |
| ENSP00000435835 | EHF | 0.6684 |
| ENSP00000244741 | CDKN1A | 0.6684 |
| ENSP00000378699 | CDK1 | 0.6684 |
| ENSP00000354568 | BRDT | 0.6684 |
| ENSP00000308461 | RND1 | 0.6658 |
| ENSP00000358525 | NGF | 0.6658 |
| ENSP00000323178 | KSR1 | 0.6658 |

| | | |
|---|---|---|
| ENSP00000356946 | FCGR3A | 0.6658 |
| ENSP00000225474 | CSF3 | 0.6658 |
| ENSP00000359424 | CHUK | 0.6658 |
| ENSP00000302564 | BCL2L1 | 0.6658 |
| ENSP00000370912 | TEC | 0.6632 |
| ENSP00000368401 | PAX6 | 0.6632 |
| ENSP00000289352 | HIST1H4H | 0.6632 |
| ENSP00000206249 | ESR1 | 0.6632 |
| ENSP00000264634 | WNT5A | 0.6605 |
| ENSP00000265440 | TFEC | 0.6605 |
| ENSP00000358092 | PRDM1 | 0.6605 |
| ENSP00000274335 | PIK3R1 | 0.6605 |
| ENSP00000362588 | PBX3 | 0.6605 |
| ENSP00000288986 | NCK1 | 0.6605 |
| ENSP00000352980 | HIST1H4A | 0.6605 |
| ENSP00000330054 | EEF1A1 | 0.6605 |
| ENSP00000344352 | ATF3 | 0.6605 |
| ENSP00000293328 | STAT5B | 0.6579 |
| ENSP00000348461 | RAC1 | 0.6579 |
| ENSP00000332353 | PTCH1 | 0.6579 |
| ENSP00000362690 | NR5A1 | 0.6579 |
| ENSP00000254227 | NR0B2 | 0.6579 |
| ENSP00000352834 | MYO1C | 0.6579 |
| ENSP00000269243 | MYH10 | 0.6579 |
| ENSP00000222390 | HGF | 0.6579 |
| ENSP00000356694 | FASLG | 0.6579 |
| ENSP00000386165 | CEBPD | 0.6579 |
| ENSP00000231656 | CDX1 | 0.6579 |
| ENSP00000293549 | WNT1 | 0.6553 |
| ENSP00000320493 | TRIP10 | 0.6553 |
| ENSP00000264731 | TP63 | 0.6553 |
| ENSP00000329357 | SP1 | 0.6553 |
| ENSP00000329418 | SOCS1 | 0.6553 |
| ENSP00000400175 | RHOA | 0.6553 |
| ENSP00000268712 | NCOR1 | 0.6553 |
| ENSP00000250003 | MYOD1 | 0.6553 |
| ENSP00000444293 | KDM5D | 0.6553 |
| ENSP00000347168 | HIST1H4J | 0.6553 |
| ENSP00000266000 | DAXX | 0.6553 |
| ENSP00000437940 | CD19 | 0.6553 |
| ENSP00000290015 | WNT9B | 0.6526 |
| ENSP00000396211 | WASF2 | 0.6526 |
| ENSP00000446280 | PCSK5 | 0.6526 |
| ENSP00000353427 | NR4A1 | 0.6526 |
| ENSP00000263388 | NOTCH3 | 0.6526 |
| ENSP00000380252 | NFE2L2 | 0.6526 |
| ENSP00000384018 | NCOR2 | 0.6526 |
| ENSP00000344782 | GFI1B | 0.6526 |
| ENSP00000252723 | EPO | 0.6526 |
| ENSP00000265372 | CREM | 0.6526 |
| ENSP00000369889 | COL2A1 | 0.6526 |
| ENSP00000302216 | ATOH1 | 0.6526 |
| ENSP00000405176 | TWIST2 | 0.6500 |

| | | |
|---|---|---|
| ENSP00000350720 | SMARCA4 | 0.6500 |
| ENSP00000265717 | PRKAR2B | 0.6500 |
| ENSP00000216797 | NFKBIA | 0.6500 |
| ENSP00000306157 | IL7R | 0.6500 |
| ENSP00000243103 | HOXC12 | 0.6500 |
| ENSP00000341826 | HNRNPA1 | 0.6500 |
| ENSP00000268171 | FURIN | 0.6500 |
| ENSP00000350512 | COPS5 | 0.6500 |
| ENSP00000351671 | CCL20 | 0.6500 |
| ENSP00000413234 | AP2A2 | 0.6500 |
| ENSP00000223023 | WASL | 0.6474 |
| ENSP00000264554 | SHC2 | 0.6474 |
| ENSP00000300954 | PCSK4 | 0.6474 |
| ENSP00000262545 | PCSK2 | 0.6474 |
| ENSP00000231509 | NR3C1 | 0.6474 |
| ENSP00000245503 | MYH2 | 0.6474 |
| ENSP00000344544 | IKZF3 | 0.6474 |
| ENSP00000222726 | HOXA5 | 0.6474 |
| ENSP00000346839 | FN1 | 0.6474 |
| ENSP00000206513 | CEBPE | 0.6474 |
| ENSP00000411552 | CDKN1C | 0.6474 |
| ENSP00000265741 | CDK14 | 0.6474 |
| ENSP00000308774 | BMX | 0.6474 |
| ENSP00000215115 | BCL7C | 0.6474 |
| ENSP00000391669 | BCAR1 | 0.6474 |
| ENSP00000348602 | AMPH | 0.6474 |
| ENSP00000308887 | WNT5B | 0.6447 |
| ENSP00000297261 | SHH | 0.6447 |
| ENSP00000284957 | RABGEF1 | 0.6447 |
| ENSP00000354855 | NFE2L1 | 0.6447 |
| ENSP00000046794 | LCP2 | 0.6447 |
| ENSP00000365280 | ID1 | 0.6447 |
| ENSP00000313199 | HNRNPD | 0.6447 |
| ENSP00000230882 | GHR | 0.6447 |
| ENSP00000295822 | EIF5A2 | 0.6447 |
| ENSP00000369461 | ECI2 | 0.6447 |
| ENSP00000345571 | E2F1 | 0.6447 |
| ENSP00000342136 | CREB3 | 0.6447 |
| ENSP00000343818 | CDK5RAP2 | 0.6447 |
| ENSP00000419782 | CDK5 | 0.6447 |
| ENSP00000308176 | BTK | 0.6447 |
| ENSP00000233948 | WNT6 | 0.6421 |
| ENSP00000447173 | VDR | 0.6421 |
| ENSP00000269305 | TP53 | 0.6421 |
| ENSP00000264657 | STAT3 | 0.6421 |
| ENSP00000364995 | SHC3 | 0.6421 |
| ENSP00000361120 | RALGDS | 0.6421 |
| ENSP00000327850 | NFATC1 | 0.6421 |
| ENSP00000317580 | NEUROG1 | 0.6421 |
| ENSP00000361066 | NCOA3 | 0.6421 |
| ENSP00000301067 | MLL2 | 0.6421 |
| ENSP00000346389 | MEF2A | 0.6421 |
| ENSP00000362649 | HDAC1 | 0.6421 |

| | | |
|---|---|---|
| ENSP00000266970 | CDK2 | 0.6421 |
| ENSP00000243440 | BATF3 | 0.6421 |
| ENSP00000364893 | ARHGEF7 | 0.6421 |
| ENSP00000288266 | APPL1 | 0.6421 |
| ENSP00000284523 | WNT3A | 0.6395 |
| ENSP00000301061 | WNT10B | 0.6395 |
| ENSP00000222270 | WBP7 | 0.6395 |
| ENSP00000256474 | VHL | 0.6395 |
| ENSP00000356999 | USF1 | 0.6395 |
| ENSP00000364912 | SPEN | 0.6395 |
| ENSP00000356022 | SOD2 | 0.6395 |
| ENSP00000338345 | SNCA | 0.6395 |
| ENSP00000341551 | SMAD4 | 0.6395 |
| ENSP00000391592 | PTPN6 | 0.6395 |
| ENSP00000251203 | PBX4 | 0.6395 |
| ENSP00000263800 | LTK | 0.6395 |
| ENSP00000303830 | INSR | 0.6395 |
| ENSP00000264716 | FOSL2 | 0.6395 |
| ENSP00000389934 | EXOC5 | 0.6395 |
| ENSP00000317955 | EEA1 | 0.6395 |
| ENSP00000296871 | CSF2 | 0.6395 |
| ENSP00000364979 | COL4A1 | 0.6395 |
| ENSP00000282050 | ATP5A1 | 0.6395 |
| ENSP00000264110 | ATF2 | 0.6395 |
| ENSP00000303909 | ABR | 0.6395 |
| ENSP00000283195 | RANBP2 | 0.6368 |
| ENSP00000382177 | MYO5A | 0.6368 |
| ENSP00000378845 | LAT | 0.6368 |
| ENSP00000370719 | ITSN1 | 0.6368 |
| ENSP00000333950 | FMN1 | 0.6368 |
| ENSP00000338934 | EZR | 0.6368 |
| ENSP00000342626 | EYA1 | 0.6368 |
| ENSP00000295206 | EN1 | 0.6368 |
| ENSP00000332171 | DMTF1 | 0.6368 |
| ENSP00000400088 | CDK3 | 0.6368 |
| ENSP00000431445 | CD3G | 0.6368 |
| ENSP00000295095 | ARHGAP15 | 0.6368 |
| ENSP00000257430 | APC | 0.6368 |
| ENSP00000216037 | XBP1 | 0.6342 |
| ENSP00000368924 | TFAP2A | 0.6342 |
| ENSP00000309572 | TERT | 0.6342 |
| ENSP00000473233 | SPIB | 0.6342 |
| ENSP00000244745 | SOX4 | 0.6342 |
| ENSP00000231487 | SKP1 | 0.6342 |
| ENSP00000329668 | SHC4 | 0.6342 |
| ENSP00000332296 | RARB | 0.6342 |
| ENSP00000291547 | PKNOX1 | 0.6342 |
| ENSP00000325120 | PGR | 0.6342 |
| ENSP00000344220 | PDPK1 | 0.6342 |
| ENSP00000254846 | KDM6B | 0.6342 |
| ENSP00000358997 | IRAK1 | 0.6342 |
| ENSP00000263851 | IL7 | 0.6342 |
| ENSP00000258743 | IL6 | 0.6342 |

| | | |
|---|---|---|
| ENSP00000359531 | GTF2B | 0.6342 |
| ENSP00000321797 | FGF8 | 0.6342 |
| ENSP00000325527 | FBN1 | 0.6342 |
| ENSP00000324464 | CSNK1D | 0.6342 |
| ENSP00000427550 | CEP192 | 0.6342 |
| ENSP00000382271 | CEP152 | 0.6342 |
| ENSP00000284000 | CEBPG | 0.6342 |
| ENSP00000361359 | CD40 | 0.6342 |
| ENSP00000359206 | BTRC | 0.6342 |
| ENSP00000362777 | ATOH7 | 0.6342 |
| ENSP00000264316 | TXK | 0.6316 |
| ENSP00000217233 | TRIB3 | 0.6316 |
| ENSP00000265354 | SRF | 0.6316 |
| ENSP00000332973 | SMAD3 | 0.6316 |
| ENSP00000316729 | RHOJ | 0.6316 |
| ENSP00000244007 | PLCG1 | 0.6316 |
| ENSP00000346508 | PDGFA | 0.6316 |
| ENSP00000398177 | KCTD7 | 0.6316 |
| ENSP00000343745 | DICER1 | 0.6316 |
| ENSP00000300692 | CD3D | 0.6316 |
| ENSP00000269321 | ARHGDIA | 0.6316 |
| ENSP00000325526 | WNT11 | 0.6289 |
| ENSP00000352802 | WIPF1 | 0.6289 |
| ENSP00000361125 | VEGFA | 0.6289 |
| ENSP00000405574 | TBL1XR1 | 0.6289 |
| ENSP00000323588 | SOX2 | 0.6289 |
| ENSP00000234677 | SARS | 0.6289 |
| ENSP00000357721 | S100A8 | 0.6289 |
| ENSP00000345206 | RBPJ | 0.6289 |
| ENSP00000299440 | RAG1 | 0.6289 |
| ENSP00000262033 | PTGES3 | 0.6289 |
| ENSP00000371432 | PRLR | 0.6289 |
| ENSP00000345494 | PLSCR1 | 0.6289 |
| ENSP00000234313 | PLEK | 0.6289 |
| ENSP00000355759 | PARP1 | 0.6289 |
| ENSP00000327758 | NKX2-5 | 0.6289 |
| ENSP00000368759 | NEDD9 | 0.6289 |
| ENSP00000348634 | MYH6 | 0.6289 |
| ENSP00000389338 | MAPK9 | 0.6289 |
| ENSP00000365016 | IRS2 | 0.6289 |
| ENSP00000343464 | HELT | 0.6289 |
| ENSP00000408617 | HDAC9 | 0.6289 |
| ENSP00000365840 | ENTPD6 | 0.6289 |
| ENSP00000262904 | E2F3 | 0.6289 |
| ENSP00000339845 | DROSHA | 0.6289 |
| ENSP00000311825 | CTBP2 | 0.6289 |
| ENSP00000357622 | CRTC2 | 0.6289 |
| ENSP00000257287 | CEP135 | 0.6289 |
| ENSP00000260967 | CDK15 | 0.6289 |
| ENSP00000085219 | CD22 | 0.6289 |
| ENSP00000370803 | CCP110 | 0.6289 |
| ENSP00000247843 | YEATS4 | 0.6263 |
| ENSP00000251413 | TUBG1 | 0.6263 |

| | | |
|---|---|---|
| ENSP00000230354 | TBP | 0.6263 |
| ENSP00000352121 | PIK3CG | 0.6263 |
| ENSP00000305056 | PCSK6 | 0.6263 |
| ENSP00000352572 | PCNT | 0.6263 |
| ENSP00000368253 | NR0B1 | 0.6263 |
| ENSP00000465655 | NFIC | 0.6263 |
| ENSP00000363318 | IL2RG | 0.6263 |
| ENSP00000430684 | IKBKB | 0.6263 |
| ENSP00000253083 | HIP1R | 0.6263 |
| ENSP00000324806 | GSK3B | 0.6263 |
| ENSP00000339186 | GRAP2 | 0.6263 |
| ENSP00000339004 | FOXG1 | 0.6263 |
| ENSP00000250448 | FOXA1 | 0.6263 |
| ENSP00000408632 | CDH13 | 0.6263 |
| ENSP00000278616 | ATM | 0.6263 |
| ENSP00000309503 | YWHAZ | 0.6237 |
| ENSP00000361626 | YBX1 | 0.6237 |
| ENSP00000245932 | VASP | 0.6237 |
| ENSP00000324856 | STK11 | 0.6237 |
| ENSP00000353089 | SRSF2 | 0.6237 |
| ENSP00000354130 | SOX10 | 0.6237 |
| ENSP00000330341 | SOCS3 | 0.6237 |
| ENSP00000364649 | SDHB | 0.6237 |
| ENSP00000238738 | RHOQ | 0.6237 |
| ENSP00000248706 | RASL11B | 0.6237 |
| ENSP00000356840 | POU2F1 | 0.6237 |
| ENSP00000289153 | PIK3CB | 0.6237 |
| ENSP00000384625 | NLK | 0.6237 |
| ENSP00000347134 | NET1 | 0.6237 |
| ENSP00000358022 | MCL1 | 0.6237 |
| ENSP00000254301 | LGALS3 | 0.6237 |
| ENSP00000263754 | KAT2B | 0.6237 |
| ENSP00000240874 | KALRN | 0.6237 |
| ENSP00000311113 | JUP | 0.6237 |
| ENSP00000245414 | IRF1 | 0.6237 |
| ENSP00000400717 | GNA13 | 0.6237 |
| ENSP00000393725 | GFRA1 | 0.6237 |
| ENSP00000334458 | GATA4 | 0.6237 |
| ENSP00000315955 | FOXA2 | 0.6237 |
| ENSP00000293831 | EIF4A1 | 0.6237 |
| ENSP00000344115 | CDH5 | 0.6237 |
| ENSP00000223368 | BCL7B | 0.6237 |
| ENSP00000393583 | AZI1 | 0.6237 |
| ENSP00000269260 | ARRB2 | 0.6237 |
| ENSP00000428340 | ANGPT1 | 0.6237 |
| ENSP00000261439 | TBC1D1 | 0.6211 |
| ENSP00000366306 | SPRY2 | 0.6211 |
| ENSP00000262160 | SMAD2 | 0.6211 |
| ENSP00000271640 | SETDB1 | 0.6211 |
| ENSP00000274031 | SETD7 | 0.6211 |
| ENSP00000299759 | RRAD | 0.6211 |
| ENSP00000267396 | REM2 | 0.6211 |
| ENSP00000267163 | RB1 | 0.6211 |

| | | |
|---|---|---|
| ENSP00000216127 | RASD2 | 0.6211 |
| ENSP00000225688 | RASD1 | 0.6211 |
| ENSP00000361266 | PTCH2 | 0.6211 |
| ENSP00000344479 | NR4A2 | 0.6211 |
| ENSP00000353679 | MME | 0.6211 |
| ENSP00000262189 | MLL3 | 0.6211 |
| ENSP00000306512 | IL8 | 0.6211 |
| ENSP00000232424 | HES1 | 0.6211 |
| ENSP00000264606 | HDAC4 | 0.6211 |
| ENSP00000406209 | EPN1 | 0.6211 |
| ENSP00000355809 | ENAH | 0.6211 |
| ENSP00000217182 | EEF1A2 | 0.6211 |
| ENSP00000355718 | DLL1 | 0.6211 |
| ENSP00000358490 | CD2 | 0.6211 |
| ENSP00000257347 | CARS2 | 0.6211 |
| ENSP00000290551 | BTG2 | 0.6211 |
| ENSP00000227758 | BIRC2 | 0.6211 |
| ENSP00000357177 | ARHGEF11 | 0.6211 |
| ENSP00000292807 | AP2M1 | 0.6211 |
| ENSP00000264335 | YWHAE | 0.6184 |
| ENSP00000300145 | XRCC6BP1 | 0.6184 |
| ENSP00000320924 | WIPF2 | 0.6184 |
| ENSP00000222305 | USF2 | 0.6184 |
| ENSP00000344818 | UBC | 0.6184 |
| ENSP00000354168 | TSSK6 | 0.6184 |
| ENSP00000382544 | TSSK2 | 0.6184 |
| ENSP00000367545 | TP73 | 0.6184 |
| ENSP00000362221 | STK40 | 0.6184 |
| ENSP00000274255 | SKP2 | 0.6184 |
| ENSP00000355652 | RHOU | 0.6184 |
| ENSP00000313420 | PRKDC | 0.6184 |
| ENSP00000346148 | PRKAA1 | 0.6184 |
| ENSP00000297494 | NOS3 | 0.6184 |
| ENSP00000172229 | NGFR | 0.6184 |
| ENSP00000359212 | LBX1 | 0.6184 |
| ENSP00000261233 | IRAK3 | 0.6184 |
| ENSP00000327890 | IL3RA | 0.6184 |
| ENSP00000341285 | HNRNPA1L2 | 0.6184 |
| ENSP00000357348 | HEY2 | 0.6184 |
| ENSP00000403459 | GRB7 | 0.6184 |
| ENSP00000281708 | FBXW7 | 0.6184 |
| ENSP00000297375 | EN2 | 0.6184 |
| ENSP00000328547 | DNMT3B | 0.6184 |
| ENSP00000253452 | COX4I1 | 0.6184 |
| ENSP00000276052 | CDK16 | 0.6184 |
| ENSP00000325690 | CARM1 | 0.6184 |
| ENSP00000302625 | AXIN2 | 0.6184 |
| ENSP00000349960 | ACTB | 0.6184 |
| ENSP00000364749 | VPS29 | 0.6158 |
| ENSP00000352425 | TUSC3 | 0.6158 |
| ENSP00000371308 | SUGT1P3 | 0.6158 |
| ENSP00000217188 | SRMS | 0.6158 |
| ENSP00000308413 | SGK3 | 0.6158 |

| | | |
|---|---|---|
| ENSP00000282030 | SETBP1 | 0.6158 |
| ENSP00000272317 | RPS27A | 0.6158 |
| ENSP00000201979 | REM1 | 0.6158 |
| ENSP00000268864 | RASL10B | 0.6158 |
| ENSP00000262735 | PPARA | 0.6158 |
| ENSP00000405965 | NANOGP1 | 0.6158 |
| ENSP00000255381 | MYH4 | 0.6158 |
| ENSP00000271555 | MEF2D | 0.6158 |
| ENSP00000324948 | MCM10 | 0.6158 |
| ENSP00000409007 | MAT1A | 0.6158 |
| ENSP00000304802 | ITGB3 | 0.6158 |
| ENSP00000268182 | IQGAP1 | 0.6158 |
| ENSP00000307786 | GNB1 | 0.6158 |
| ENSP00000262493 | GNAO1 | 0.6158 |
| ENSP00000078429 | GNA11 | 0.6158 |
| ENSP00000252997 | GATA5 | 0.6158 |
| ENSP00000364265 | FKBP5 | 0.6158 |
| ENSP00000357555 | ENSG00000259131 | 0.6158 |
| ENSP00000339428 | EMX2 | 0.6158 |
| ENSP00000398632 | EARS2 | 0.6158 |
| ENSP00000341698 | DAB1 | 0.6158 |
| ENSP00000262653 | CBFA2T2 | 0.6158 |
| ENSP00000241052 | CAT | 0.6158 |
| ENSP00000229922 | CAP2 | 0.6158 |
| ENSP00000312987 | BMPR1A | 0.6158 |
| ENSP00000242728 | BHLHE41 | 0.6158 |
| ENSP00000312856 | BCL10 | 0.6158 |
| ENSP00000349547 | ARHGAP18 | 0.6158 |
| ENSP00000452871 | ABLIM1 | 0.6158 |
| ENSP00000293813 | DLG4 | 0.6158 |
| ENSP00000245960 | ZC3H12A | 0.6132 |
| ENSP00000324422 | WASH6P | 0.6132 |
| ENSP00000358708 | U2SURP | 0.6132 |
| ENSP00000234420 | TUBB8 | 0.6132 |
| ENSP00000263036 | SMGA | 0.6132 |
| ENSP00000258411 | RPS4XP21 | 0.6132 |
| ENSP00000326804 | PRF1 | 0.6132 |
| ENSP00000347507 | ONECUT2 | 0.6132 |
| ENSP00000020945 | NUDT2 | 0.6132 |
| ENSP00000358918 | NPIPL3 | 0.6132 |
| ENSP00000230449 | MRRFP1 | 0.6132 |
| ENSP00000264935 | MAPKAPK5 | 0.6132 |
| ENSP00000321656 | FBXW4 | 0.6132 |
| ENSP00000344871 | ENSG00000258508 | 0.6132 |
| ENSP00000387282 | ENSG00000258447 | 0.6132 |
| ENSP00000324884 | ENSG00000258210 | 0.6132 |
| ENSP00000311505 | ENSG00000248354 | 0.6132 |
| ENSP00000242338 | ENSG00000236954 | 0.6132 |
| ENSP00000239144 | ENSG00000232856 | 0.6132 |
| ENSP00000232607 | DRD1 | 0.6132 |
| ENSP00000340347 | CCNYL2 | 0.6132 |
| ENSP00000358795 | CATSPER2 | 0.6132 |
| ENSP00000357292 | CAND1 | 0.6132 |

| | | |
|---|---|---|
| ENSP00000262320 | C1R | 0.6132 |
| ENSP00000219070 | AARSD1 | 0.6132 |
| ENSP00000365735 | ZSCAN20 | 0.6105 |
| ENSP00000354876 | VWA2 | 0.6105 |
| ENSP00000249636 | TMSB4XP4 | 0.6105 |
| ENSP00000338548 | STAP2 | 0.6105 |
| ENSP00000313829 | SEPT7L | 0.6105 |
| ENSP00000382595 | NPIPP1 | 0.6105 |
| ENSP00000291442 | MST1L | 0.6105 |
| ENSP00000314067 | MCOLN3 | 0.6105 |
| ENSP00000351273 | LPPR1 | 0.6105 |
| ENSP00000264664 | KIAA0100 | 0.6105 |
| ENSP00000362361 | HMGN4 | 0.6105 |
| ENSP00000262407 | hCG_33128 | 0.6105 |
| ENSP00000365233 | GABRR3 | 0.6105 |
| ENSP00000365380 | FXYD4 | 0.6105 |
| ENSP00000391774 | FGL1 | 0.6105 |
| ENSP00000444972 | FAM74A2 | 0.6105 |
| ENSP00000231790 | ENSG00000267149 | 0.6105 |
| ENSP00000423665 | ENSG00000262314 | 0.6105 |
| ENSP00000219409 | ENSG00000260527 | 0.6105 |
| ENSP00000426909 | ENSG00000250765 | 0.6105 |
| ENSP00000366843 | ENSG00000235153 | 0.6105 |
| ENSP00000269216 | DNAAF3 | 0.6105 |
| ENSP00000219476 | DGAT2L7P | 0.6105 |
| ENSP00000326514 | DENND4C | 0.6105 |
| ENSP00000391372 | CMAHP | 0.6105 |
| ENSP00000355249 | CCNJ | 0.6105 |
| ENSP00000253792 | BTNL2 | 0.6105 |
| ENSP00000383042 | ANKRD36BP1 | 0.6105 |
| ENSP00000372170 | ZNF730 | 0.6079 |
| ENSP00000174618 | ZNF623 | 0.6079 |
| ENSP00000352264 | ZNF503-AS2 | 0.6079 |
| ENSP00000263734 | SNX29P2 | 0.6079 |
| ENSP00000336702 | SNX29 | 0.6079 |
| ENSP00000230658 | SNURFL | 0.6079 |
| ENSP00000336524 | SNHG16 | 0.6079 |
| ENSP00000314414 | SKA2L | 0.6079 |
| ENSP00000239165 | POM121L4P | 0.6079 |
| ENSP00000360561 | POM121L1P | 0.6079 |
| ENSP00000338283 | OR5V1 | 0.6079 |
| ENSP00000343027 | NG38 | 0.6079 |
| ENSP00000351490 | NBPF4 | 0.6079 |
| ENSP00000367629 | MIR7-3HG | 0.6079 |
| ENSP00000264867 | MICA | 0.6079 |
| ENSP00000245539 | LOC619207 | 0.6079 |
| ENSP00000225983 | LINC00493 | 0.6079 |
| ENSP00000417653 | LINC00273 | 0.6079 |
| ENSP00000260762 | GYG2P1 | 0.6079 |
| ENSP00000216223 | GNRHR2 | 0.6079 |
| ENSP00000365576 | FLJ00096 | 0.6079 |
| ENSP00000239243 | FAM74A1 | 0.6079 |
| ENSP00000353099 | FAM205B | 0.6079 |

| | | |
|---|---|---|
| ENSP00000347979 | FAM201A | 0.6079 |
| ENSP00000344666 | FAM182A | 0.6079 |
| ENSP00000365745 | FAM157A | 0.6079 |
| ENSP00000300161 | ENSG00000259141 | 0.6079 |
| ENSP00000229002 | ENSG00000257755 | 0.6079 |
| ENSP00000366563 | ENSG00000240215 | 0.6079 |
| ENSP00000227378 | ENSG00000229450 | 0.6079 |
| ENSP00000278568 | ENSG00000228006 | 0.6079 |
| ENSP00000284981 | ENSG00000197511 | 0.6079 |
| ENSP00000427802 | ENSG00000197426 | 0.6079 |
| ENSP00000362208 | ENSG00000178803 | 0.6079 |
| ENSP00000430706 | EIF5AL1 | 0.6079 |
| ENSP00000254231 | DEFB117 | 0.6079 |
| ENSP00000239151 | CC2D2B | 0.6079 |
| ENSP00000377941 | C18orf63 | 0.6079 |
| ENSP00000245552 | C12orf52 | 0.6079 |
| ENSP00000253861 | C10orf131 | 0.6079 |
| ENSP00000295108 | BRWD1-IT2 | 0.6079 |
| ENSP00000037243 | ASB16-AS1 | 0.6079 |
| ENSP00000351255 | STAT4 | 0.6053 |
| ENSP00000305769 | SMAD1 | 0.6053 |
| ENSP00000212015 | SIRT1 | 0.6053 |
| ENSP00000357727 | S100A9 | 0.6053 |
| ENSP00000356530 | RNASEL | 0.6053 |
| ENSP00000362768 | RBL1 | 0.6053 |
| ENSP00000303208 | PCSK9 | 0.6053 |
| ENSP00000301905 | PBK | 0.6053 |
| ENSP00000377721 | NR2F2 | 0.6053 |
| ENSP00000240055 | NFYB | 0.6053 |
| ENSP00000056233 | NFE2L3 | 0.6053 |
| ENSP00000451211 | NEDD1 | 0.6053 |
| ENSP00000226209 | MYH3 | 0.6053 |
| ENSP00000401399 | MYD88 | 0.6053 |
| ENSP00000363533 | MDH1B | 0.6053 |
| ENSP00000352157 | MAPK10 | 0.6053 |
| ENSP00000301653 | KRT16 | 0.6053 |
| ENSP00000309148 | KLK6 | 0.6053 |
| ENSP00000254958 | JAG1 | 0.6053 |
| ENSP00000304895 | IRS1 | 0.6053 |
| ENSP00000299767 | HSP90B1 | 0.6053 |
| ENSP00000343246 | HOXA1 | 0.6053 |
| ENSP00000266458 | GABARAPL1 | 0.6053 |
| ENSP00000342307 | FOXM1 | 0.6053 |
| ENSP00000366534 | FOXH1 | 0.6053 |
| ENSP00000360216 | DNTT | 0.6053 |
| ENSP00000347890 | DNM2 | 0.6053 |
| ENSP00000354923 | DMD | 0.6053 |
| ENSP00000360020 | DIRAS3 | 0.6053 |
| ENSP00000422753 | DDX41 | 0.6053 |
| ENSP00000228872 | CDKN1B | 0.6053 |
| ENSP00000354566 | CD3E | 0.6053 |
| ENSP00000377978 | CCL4L2 | 0.6053 |
| ENSP00000418960 | BRCA1 | 0.6053 |

| | | |
|---|---|---|
| ENSP00000349076 | ATN1 | 0.6053 |
| ENSP00000343535 | USP7 | 0.6026 |
| ENSP00000362481 | UPRT | 0.6026 |
| ENSP00000337853 | TRAF6 | 0.6026 |
| ENSP00000262953 | TLE2 | 0.6026 |
| ENSP00000361554 | TIE1 | 0.6026 |
| ENSP00000245479 | SOX9 | 0.6026 |
| ENSP00000363812 | RXRB | 0.6026 |
| ENSP00000356480 | RNF2 | 0.6026 |
| ENSP00000265335 | RAD50 | 0.6026 |
| ENSP00000356346 | PTPRC | 0.6026 |
| ENSP00000366413 | POU4F1 | 0.6026 |
| ENSP00000407509 | PNPLA6 | 0.6026 |
| ENSP00000222254 | PIK3R2 | 0.6026 |
| ENSP00000465676 | PIAS2 | 0.6026 |
| ENSP00000362413 | PGK1 | 0.6026 |
| ENSP00000334188 | PFDN5 | 0.6026 |
| ENSP00000371875 | NKX3-2 | 0.6026 |
| ENSP00000370253 | NKX3-1 | 0.6026 |
| ENSP00000345702 | NFYA | 0.6026 |
| ENSP00000228644 | MYF5 | 0.6026 |
| ENSP00000302486 | MAP2K1 | 0.6026 |
| ENSP00000339952 | KSR2 | 0.6026 |
| ENSP00000267569 | JDP2 | 0.6026 |
| ENSP00000263339 | IL1A | 0.6026 |
| ENSP00000363827 | HSPG2 | 0.6026 |
| ENSP00000338272 | HEY1 | 0.6026 |
| ENSP00000371236 | GART | 0.6026 |
| ENSP00000360025 | GADD45A | 0.6026 |
| ENSP00000384708 | FSHR | 0.6026 |
| ENSP00000360157 | FOXD3 | 0.6026 |
| ENSP00000307940 | EEF2 | 0.6026 |
| ENSP00000266991 | DHH | 0.6026 |
| ENSP00000352929 | CSNK1E | 0.6026 |
| ENSP00000384053 | CSF2RB | 0.6026 |
| ENSP00000327513 | CSF1 | 0.6026 |
| ENSP00000255465 | CCNA1 | 0.6026 |
| ENSP00000272298 | CALM2 | 0.6026 |
| ENSP00000264705 | CAD | 0.6026 |
| ENSP00000293288 | BAX | 0.6026 |
| ENSP00000266744 | ASCL1 | 0.6026 |
| ENSP00000351926 | AP2A1 | 0.6026 |
| ENSP00000291582 | AIRE | 0.6026 |
| ENSP00000221561 | AES | 0.6026 |
| ENSP00000251412 | TUBG2 | 0.6000 |
| ENSP00000365682 | TLE1 | 0.6000 |
| ENSP00000281537 | TJP1 | 0.6000 |
| ENSP00000219548 | STUB1 | 0.6000 |
| ENSP00000354394 | STAT1 | 0.6000 |
| ENSP00000231061 | SPARC | 0.6000 |
| ENSP00000356832 | SGK1 | 0.6000 |
| ENSP00000368884 | RPS6KA3 | 0.6000 |
| ENSP00000317985 | ROCK2 | 0.6000 |

| | | |
|---|---|---|
| ENSP00000296273 | RFC4 | 0.6000 |
| ENSP00000342026 | PRDX6 | 0.6000 |
| ENSP00000342931 | POU1F1 | 0.6000 |
| ENSP00000363387 | PDIK1L | 0.6000 |
| ENSP00000355865 | PARK2 | 0.6000 |
| ENSP00000302189 | NHLH1 | 0.6000 |
| ENSP00000297689 | NFIL3 | 0.6000 |
| ENSP00000418842 | NDUFA6 | 0.6000 |
| ENSP00000233154 | NCK2 | 0.6000 |
| ENSP00000262873 | MYH7B | 0.6000 |
| ENSP00000362036 | MRPS16 | 0.6000 |
| ENSP00000340874 | MEF2C | 0.6000 |
| ENSP00000178640 | MAP2K5 | 0.6000 |
| ENSP00000349785 | MAP1LC3C | 0.6000 |
| ENSP00000265854 | MAD1L1 | 0.6000 |
| ENSP00000040584 | HOXC8 | 0.6000 |
| ENSP00000297596 | GEM | 0.6000 |
| ENSP00000242480 | EGR2 | 0.6000 |
| ENSP00000246069 | DSTN | 0.6000 |
| ENSP00000381657 | DOT1L | 0.6000 |
| ENSP00000304669 | CTNNA1 | 0.6000 |
| ENSP00000204604 | CHRD | 0.6000 |
| ENSP00000266557 | CD27 | 0.6000 |
| ENSP00000320866 | CALR | 0.6000 |
| ENSP00000337261 | ARHGEF1 | 0.6000 |
| ENSP00000262017 | ITGB3 | 0.6000 |
| ENSP00000248975 | YWHAH | 0.5974 |
| ENSP00000411532 | TOP2A | 0.5974 |
| ENSP00000291232 | TNFRSF13C | 0.5974 |
| ENSP00000363089 | TLR4 | 0.5974 |
| ENSP00000320935 | SLC2A4 | 0.5974 |
| ENSP00000322775 | RHBDF2 | 0.5974 |
| ENSP00000369411 | RFC3 | 0.5974 |
| ENSP00000405963 | RASGRF1 | 0.5974 |
| ENSP00000348812 | PTPRD | 0.5974 |
| ENSP00000360683 | PTPN1 | 0.5974 |
| ENSP00000332816 | PTK2B | 0.5974 |
| ENSP00000362334 | PSMB2 | 0.5974 |
| ENSP00000361512 | PRPS1 | 0.5974 |
| ENSP00000415481 | PROM1 | 0.5974 |
| ENSP00000326031 | PPP1CA | 0.5974 |
| ENSP00000329170 | POU3F2 | 0.5974 |
| ENSP00000355031 | PER3 | 0.5974 |
| ENSP00000308024 | PCSK1 | 0.5974 |
| ENSP00000262077 | NUP153 | 0.5974 |
| ENSP00000327251 | NOS2 | 0.5974 |
| ENSP00000345530 | NEDD4 | 0.5974 |
| ENSP00000296474 | MST1R | 0.5974 |
| ENSP00000272418 | MRPS5 | 0.5974 |
| ENSP00000300651 | MED1 | 0.5974 |
| ENSP00000215832 | MAPK1 | 0.5974 |
| ENSP00000251047 | LMAN1 | 0.5974 |
| ENSP00000294954 | LHCGR | 0.5974 |

| | | |
|---|---|---|
| ENSP00000419923 | KLF6 | 0.5974 |
| ENSP00000363804 | KLF4 | 0.5974 |
| ENSP00000414982 | KLC1 | 0.5974 |
| ENSP00000363998 | ITCH | 0.5974 |
| ENSP00000342557 | IL4I1 | 0.5974 |
| ENSP00000301585 | ICT1 | 0.5974 |
| ENSP00000270112 | HUNK | 0.5974 |
| ENSP00000318687 | HSPH1 | 0.5974 |
| ENSP00000257555 | HNF1A | 0.5974 |
| ENSP00000328773 | HEXIM1 | 0.5974 |
| ENSP00000322542 | GTF2I | 0.5974 |
| ENSP00000229239 | GAPDH | 0.5974 |
| ENSP00000273550 | FTH1 | 0.5974 |
| ENSP00000265171 | EGF | 0.5974 |
| ENSP00000381932 | DYRK1A | 0.5974 |
| ENSP00000313391 | DAB2 | 0.5974 |
| ENSP00000260433 | CYP19A1 | 0.5974 |
| ENSP00000409346 | CISH | 0.5974 |
| ENSP00000372023 | CHEK2 | 0.5974 |
| ENSP00000314458 | CDC42 | 0.5974 |
| ENSP00000286186 | CASP10 | 0.5974 |
| ENSP00000376943 | BCL2L11 | 0.5974 |
| ENSP00000244769 | ATXN1 | 0.5974 |
| ENSP00000314897 | ANGPT2 | 0.5974 |
| ENSP00000367869 | GNB1 | 0.5974 |
| ENSP00000262238 | YY1 | 0.5947 |
| ENSP00000238081 | YWHAQ | 0.5947 |
| ENSP00000264951 | XRN1 | 0.5947 |
| ENSP00000340677 | WNT8B | 0.5947 |
| ENSP00000277575 | USP6NL | 0.5947 |
| ENSP00000312150 | TRIB1 | 0.5947 |
| ENSP00000467176 | TNNT1 | 0.5947 |
| ENSP00000385834 | TF | 0.5947 |
| ENSP00000376611 | TDG | 0.5947 |
| ENSP00000376076 | SUMO1 | 0.5947 |
| ENSP00000359025 | STXBP3 | 0.5947 |
| ENSP00000244020 | SRSF6 | 0.5947 |
| ENSP00000377892 | SRSF5 | 0.5947 |
| ENSP00000464034 | SOCS7 | 0.5947 |
| ENSP00000351593 | SMTN | 0.5947 |
| ENSP00000352900 | RXRG | 0.5947 |
| ENSP00000473172 | RUVBL2 | 0.5947 |
| ENSP00000259808 | RIPK1 | 0.5947 |
| ENSP00000308620 | RAG2 | 0.5947 |
| ENSP00000331602 | PRKCD | 0.5947 |
| ENSP00000360290 | PRKAA2 | 0.5947 |
| ENSP00000321746 | PDLIM5 | 0.5947 |
| ENSP00000361949 | PABPC4 | 0.5947 |
| ENSP00000318165 | OS9 | 0.5947 |
| ENSP00000351894 | NCOA6 | 0.5947 |
| ENSP00000241651 | MYOG | 0.5947 |
| ENSP00000285039 | MYO5B | 0.5947 |
| ENSP00000364361 | MLTK | 0.5947 |

| | | |
|---|---|---|
| ENSP00000327070 | MDH2 | 0.5947 |
| ENSP00000264156 | MCM6 | 0.5947 |
| ENSP00000262105 | MCM4 | 0.5947 |
| ENSP00000362717 | LHX2 | 0.5947 |
| ENSP00000307078 | KIF5B | 0.5947 |
| ENSP00000361202 | IRS4 | 0.5947 |
| ENSP00000268638 | IRF8 | 0.5947 |
| ENSP00000256458 | IRAK2 | 0.5947 |
| ENSP00000256079 | IPO8 | 0.5947 |
| ENSP00000369293 | IL2RA | 0.5947 |
| ENSP00000308252 | HOXB3 | 0.5947 |
| ENSP00000353151 | HOXA4 | 0.5947 |
| ENSP00000349168 | HNRNPH1 | 0.5947 |
| ENSP00000260372 | HAUS2 | 0.5947 |
| ENSP00000398131 | GSPT1 | 0.5947 |
| ENSP00000334448 | GNG2 | 0.5947 |
| ENSP00000371790 | FGF9 | 0.5947 |
| ENSP00000384169 | FBLN2 | 0.5947 |
| ENSP00000334100 | EXOC7 | 0.5947 |
| ENSP00000281172 | EPS8 | 0.5947 |
| ENSP00000351209 | EPHA2 | 0.5947 |
| ENSP00000351520 | ENTPD4 | 0.5947 |
| ENSP00000329715 | DRG1 | 0.5947 |
| ENSP00000233668 | DOK1 | 0.5947 |
| ENSP00000280333 | DOCK1 | 0.5947 |
| ENSP00000264709 | DNMT3A | 0.5947 |
| ENSP00000052754 | DCN | 0.5947 |
| ENSP00000382004 | CTNND1 | 0.5947 |
| ENSP00000264010 | CTCF | 0.5947 |
| ENSP00000370938 | CDK8 | 0.5947 |
| ENSP00000245451 | BMP4 | 0.5947 |
| ENSP00000257749 | BACH2 | 0.5947 |
| ENSP00000341071 | ARHGEF3 | 0.5947 |
| ENSP00000371897 | ARHGAP5 | 0.5947 |
| ENSP00000260526 | ARHGAP29 | 0.5947 |
| ENSP00000350199 | AP1B1 | 0.5947 |
| ENSP00000421592 | ALYREF | 0.5947 |
| ENSP00000216194 | ADSL | 0.5947 |
| ENSP00000355645 | ACTA1 | 0.5947 |
| ENSP00000375977 | XRCC5 | 0.5921 |
| ENSP00000457230 | WWOX | 0.5921 |
| ENSP00000345195 | UBQLN2 | 0.5921 |
| ENSP00000200457 | TRIP6 | 0.5921 |
| ENSP00000296795 | TLR3 | 0.5921 |
| ENSP00000358635 | SYNCRIP | 0.5921 |
| ENSP00000365877 | SUV39H1 | 0.5921 |
| ENSP00000315768 | STAT2 | 0.5921 |
| ENSP00000229390 | SRSF9 | 0.5921 |
| ENSP00000357130 | SPTA1 | 0.5921 |
| ENSP00000400591 | SNRPE | 0.5921 |
| ENSP00000322457 | SIAH2 | 0.5921 |
| ENSP00000435412 | RPS6KA1 | 0.5921 |
| ENSP00000382697 | ROCK1 | 0.5921 |

| | | |
|---|---|---|
| ENSP00000288199 | RNF111 | 0.5921 |
| ENSP00000361021 | PTEN | 0.5921 |
| ENSP00000263125 | PRKCQ | 0.5921 |
| ENSP00000314949 | POLR2A | 0.5921 |
| ENSP00000300093 | PLK1 | 0.5921 |
| ENSP00000441875 | PHB2 | 0.5921 |
| ENSP00000220597 | PAG1 | 0.5921 |
| ENSP00000327801 | P4HB | 0.5921 |
| ENSP00000325819 | NR2F1 | 0.5921 |
| ENSP00000396620 | NFYC | 0.5921 |
| ENSP00000358815 | NEURL1B | 0.5921 |
| ENSP00000332643 | NDN | 0.5921 |
| ENSP00000318195 | NCL | 0.5921 |
| ENSP00000381599 | MX1 | 0.5921 |
| ENSP00000368174 | MCM8 | 0.5921 |
| ENSP00000311005 | MAPK7 | 0.5921 |
| ENSP00000296509 | MAD2L1 | 0.5921 |
| ENSP00000390651 | IRAK4 | 0.5921 |
| ENSP00000355140 | HOXB1 | 0.5921 |
| ENSP00000302967 | HDAC3 | 0.5921 |
| ENSP00000294117 | GNG3 | 0.5921 |
| ENSP00000262958 | GNA15 | 0.5921 |
| ENSP00000386200 | FOXP2 | 0.5921 |
| ENSP00000334472 | FOXE3 | 0.5921 |
| ENSP00000264498 | FGF2 | 0.5921 |
| ENSP00000290246 | ELMO2 | 0.5921 |
| ENSP00000385269 | ELAVL1 | 0.5921 |
| ENSP00000269349 | EIF4A3 | 0.5921 |
| ENSP00000391944 | EEF1D | 0.5921 |
| ENSP00000352516 | DNMT1 | 0.5921 |
| ENSP00000286648 | DCK | 0.5921 |
| ENSP00000365782 | CEP78 | 0.5921 |
| ENSP00000362613 | CDX4 | 0.5921 |
| ENSP00000276925 | CDKN2B | 0.5921 |
| ENSP00000222005 | CDC37 | 0.5921 |
| ENSP00000330237 | CASP9 | 0.5921 |
| ENSP00000349467 | CALM1 | 0.5921 |
| ENSP00000414303 | BDNF | 0.5921 |
| ENSP00000318585 | BACE1 | 0.5921 |
| ENSP00000320485 | ARID1A | 0.5921 |
| ENSP00000242057 | AHR | 0.5921 |
| ENSP00000312618 | ACAD9 | 0.5921 |
| ENSP00000381739 | WNT8A | 0.5895 |
| ENSP00000262640 | VAMP7 | 0.5895 |
| ENSP00000381045 | UBE3A | 0.5895 |
| ENSP00000291552 | U2AF1 | 0.5895 |
| ENSP00000166345 | TRIP13 | 0.5895 |
| ENSP00000246957 | TRAP1 | 0.5895 |
| ENSP00000312143 | TNS3 | 0.5895 |
| ENSP00000246112 | TLE6 | 0.5895 |
| ENSP00000201031 | TFAP2C | 0.5895 |
| ENSP00000252996 | TAF4 | 0.5895 |
| ENSP00000295987 | SYN1 | 0.5895 |

| | | |
|---|---|---|
| ENSP00000398273 | SOX5 | 0.5895 |
| ENSP00000373637 | SMYD3 | 0.5895 |
| ENSP00000441954 | SMAD5 | 0.5895 |
| ENSP00000280551 | SEC24D | 0.5895 |
| ENSP00000262752 | RPS6KA6 | 0.5895 |
| ENSP00000349959 | RICTOR | 0.5895 |
| ENSP00000338629 | RERE | 0.5895 |
| ENSP00000377947 | RARG | 0.5895 |
| ENSP00000217185 | PTK6 | 0.5895 |
| ENSP00000424595 | PRPS1L1 | 0.5895 |
| ENSP00000312735 | POLR2B | 0.5895 |
| ENSP00000263246 | PACSIN2 | 0.5895 |
| ENSP00000455507 | OBSCN | 0.5895 |
| ENSP00000246672 | NR1D1 | 0.5895 |
| ENSP00000264051 | NGEF | 0.5895 |
| ENSP00000250495 | NEDD8 | 0.5895 |
| ENSP00000354251 | NCKAP1 | 0.5895 |
| ENSP00000356505 | NCF2 | 0.5895 |
| ENSP00000420477 | NACAD | 0.5895 |
| ENSP00000279022 | MYL9 | 0.5895 |
| ENSP00000384330 | MYH8 | 0.5895 |
| ENSP00000258455 | MRPS9 | 0.5895 |
| ENSP00000216122 | MCM5 | 0.5895 |
| ENSP00000265056 | MCM2 | 0.5895 |
| ENSP00000354649 | MAGT1 | 0.5895 |
| ENSP00000265165 | LEF1 | 0.5895 |
| ENSP00000296585 | ITGA2 | 0.5895 |
| ENSP00000349770 | IRF5 | 0.5895 |
| ENSP00000352575 | INPP5D | 0.5895 |
| ENSP00000362115 | INPP5B | 0.5895 |
| ENSP00000268035 | IGF1R | 0.5895 |
| ENSP00000331201 | HGS | 0.5895 |
| ENSP00000365807 | GNA14 | 0.5895 |
| ENSP00000296805 | GFM2 | 0.5895 |
| ENSP00000369538 | GDI2 | 0.5895 |
| ENSP00000346886 | GABPA | 0.5895 |
| ENSP00000378529 | FZR1 | 0.5895 |
| ENSP00000361672 | FOXO6 | 0.5895 |
| ENSP00000352414 | FGF17 | 0.5895 |
| ENSP00000265094 | FBXW11 | 0.5895 |
| ENSP00000239938 | EGR1 | 0.5895 |
| ENSP00000005340 | DVL2 | 0.5895 |
| ENSP00000078445 | CREB3L3 | 0.5895 |
| ENSP00000258424 | COX5B | 0.5895 |
| ENSP00000331902 | COL4A5 | 0.5895 |
| ENSP00000278935 | CEP164 | 0.5895 |
| ENSP00000231021 | CDH9 | 0.5895 |
| ENSP00000332293 | ASCL2 | 0.5895 |
| ENSP00000350297 | ASAP1 | 0.5895 |
| ENSP00000250617 | ARHGEF6 | 0.5895 |
| ENSP00000298316 | ARF6 | 0.5895 |
| ENSP00000332744 | ALX4 | 0.5895 |
| ENSP00000363965 | ALPL | 0.5895 |

| | | |
|---|---|---|
| ENSP00000262211 | SGK3 | 0.5895 |
| ENSP00000322439 | TUFM | 0.5868 |
| ENSP00000262395 | TRAF4 | 0.5868 |
| ENSP00000327959 | TGIF1 | 0.5868 |
| ENSP00000257566 | TBX3 | 0.5868 |
| ENSP00000258962 | SRSF1 | 0.5868 |
| ENSP00000341327 | SOCS4 | 0.5868 |
| ENSP00000472847 | SARS2 | 0.5868 |
| ENSP00000262133 | RBL2 | 0.5868 |
| ENSP00000311857 | PTPN2 | 0.5868 |
| ENSP00000304350 | PRPF8 | 0.5868 |
| ENSP00000309591 | PRKACA | 0.5868 |
| ENSP00000324804 | PPP2R1A | 0.5868 |
| ENSP00000270861 | PLK4 | 0.5868 |
| ENSP00000247970 | PIN1 | 0.5868 |
| ENSP00000254657 | PER2 | 0.5868 |
| ENSP00000362824 | OGT | 0.5868 |
| ENSP00000253727 | NR1H2 | 0.5868 |
| ENSP00000369519 | MTAP | 0.5868 |
| ENSP00000263056 | MAP3K8 | 0.5868 |
| ENSP00000357283 | LMNA | 0.5868 |
| ENSP00000265713 | KAT6A | 0.5868 |
| ENSP00000261023 | ITGAV | 0.5868 |
| ENSP00000295731 | IHH | 0.5868 |
| ENSP00000324173 | HSPA5 | 0.5868 |
| ENSP00000283179 | HNRNPU | 0.5868 |
| ENSP00000368698 | HIVEP1 | 0.5868 |
| ENSP00000282728 | HHEX | 0.5868 |
| ENSP00000417401 | HELZ2 | 0.5868 |
| ENSP00000231121 | HAND1 | 0.5868 |
| ENSP00000264424 | GUCY1B3 | 0.5868 |
| ENSP00000354040 | GTPBP4 | 0.5868 |
| ENSP00000308610 | GPD2 | 0.5868 |
| ENSP00000358867 | GNAI3 | 0.5868 |
| ENSP00000264895 | FRAS1 | 0.5868 |
| ENSP00000322909 | FHL2 | 0.5868 |
| ENSP00000267843 | FGF7 | 0.5868 |
| ENSP00000294312 | FGF19 | 0.5868 |
| ENSP00000000442 | ESRRA | 0.5868 |
| ENSP00000419449 | EIF1 | 0.5868 |
| ENSP00000236957 | EEF1B2 | 0.5868 |
| ENSP00000378735 | DNAJC6 | 0.5868 |
| ENSP00000341692 | DAP3 | 0.5868 |
| ENSP00000271651 | CTSK | 0.5868 |
| ENSP00000290921 | CTBP1 | 0.5868 |
| ENSP00000304408 | COL3A1 | 0.5868 |
| ENSP00000355370 | CNTF | 0.5868 |
| ENSP00000359663 | CD40LG | 0.5868 |
| ENSP00000293272 | CCL5 | 0.5868 |
| ENSP00000367408 | CASK | 0.5868 |
| ENSP00000339740 | CAMK2D | 0.5868 |
| ENSP00000379098 | CAMK2B | 0.5868 |
| ENSP00000369497 | BRCA2 | 0.5868 |

| | | |
|---|---|---|
| ENSP00000220531 | BLOC1S6 | 0.5868 |
| ENSP00000316779 | BIN1 | 0.5868 |
| ENSP00000286639 | BATF | 0.5868 |
| ENSP00000452780 | B2M | 0.5868 |
| ENSP00000218548 | ATP12A | 0.5868 |
| ENSP00000393539 | ARHGAP23 | 0.5868 |
| ENSP00000252699 | ACTN4 | 0.5868 |
| ENSP00000171887 | TNS1 | 0.5842 |
| ENSP00000239849 | TNFSF11 | 0.5842 |
| ENSP00000162749 | TNFRSF1A | 0.5842 |
| ENSP00000358931 | TKTL1 | 0.5842 |
| ENSP00000366863 | TBC1D4 | 0.5842 |
| ENSP00000240327 | SPOP | 0.5842 |
| ENSP00000262435 | SMURF2 | 0.5842 |
| ENSP00000354621 | SMURF1 | 0.5842 |
| ENSP00000263121 | SMARCB1 | 0.5842 |
| ENSP00000262519 | SETD1A | 0.5842 |
| ENSP00000348068 | SERPINA1 | 0.5842 |
| ENSP00000357998 | SEC63 | 0.5842 |
| ENSP00000321845 | SEC24C | 0.5842 |
| ENSP00000312122 | SEC13 | 0.5842 |
| ENSP00000337632 | SARNP | 0.5842 |
| ENSP00000292123 | SAFB | 0.5842 |
| ENSP00000374036 | RPH3A | 0.5842 |
| ENSP00000341243 | RBPJL | 0.5842 |
| ENSP00000369424 | RBBP7 | 0.5842 |
| ENSP00000286364 | RASA2 | 0.5842 |
| ENSP00000231572 | RARS | 0.5842 |
| ENSP00000367830 | PRKCZ | 0.5842 |
| ENSP00000351410 | PRKAR1A | 0.5842 |
| ENSP00000418447 | PPP2CA | 0.5842 |
| ENSP00000296440 | PLXNB1 | 0.5842 |
| ENSP00000206542 | OSGEP | 0.5842 |
| ENSP00000363216 | OGDHL | 0.5842 |
| ENSP00000265371 | NRP1 | 0.5842 |
| ENSP00000327213 | NRIP1 | 0.5842 |
| ENSP00000353452 | MYLK | 0.5842 |
| ENSP00000241600 | MRPS2 | 0.5842 |
| ENSP00000325863 | MRE11A | 0.5842 |
| ENSP00000395535 | MECP2 | 0.5842 |
| ENSP00000358335 | MAP3K7 | 0.5842 |
| ENSP00000262445 | MAP2K4 | 0.5842 |
| ENSP00000363970 | MAP1LC3A | 0.5842 |
| ENSP00000340684 | MAOA | 0.5842 |
| ENSP00000392466 | LDB1 | 0.5842 |
| ENSP00000377954 | LARS | 0.5842 |
| ENSP00000171111 | KEAP1 | 0.5842 |
| ENSP00000200181 | ITGB4 | 0.5842 |
| ENSP00000305244 | IRX1 | 0.5842 |
| ENSP00000357178 | INSRR | 0.5842 |
| ENSP00000310219 | HSPA6 | 0.5842 |
| ENSP00000243108 | HOXC6 | 0.5842 |
| ENSP00000304229 | HINT1 | 0.5842 |

| | | |
|---|---|---|
| ENSP00000355689 | GUK1 | 0.5842 |
| ENSP00000298743 | GAS1 | 0.5842 |
| ENSP00000326371 | FOXC2 | 0.5842 |
| ENSP00000264748 | FGFRL1 | 0.5842 |
| ENSP00000258106 | EMX1 | 0.5842 |
| ENSP00000392094 | EFTUD2 | 0.5842 |
| ENSP00000368686 | E2F4 | 0.5842 |
| ENSP00000370376 | DUT | 0.5842 |
| ENSP00000325817 | CYFIP2 | 0.5842 |
| ENSP00000357981 | CTSS | 0.5842 |
| ENSP00000315791 | CSTF3 | 0.5842 |
| ENSP00000406751 | CRY2 | 0.5842 |
| ENSP00000247655 | COX7C | 0.5842 |
| ENSP00000249923 | COPB1 | 0.5842 |
| ENSP00000222792 | CHN2 | 0.5842 |
| ENSP00000264982 | CEP70 | 0.5842 |
| ENSP00000289746 | CDH15 | 0.5842 |
| ENSP00000434614 | CDC27 | 0.5842 |
| ENSP00000308450 | CDC20 | 0.5842 |
| ENSP00000274026 | CCNA2 | 0.5842 |
| ENSP00000368104 | BMP2 | 0.5842 |
| ENSP00000262623 | ATP4A | 0.5842 |
| ENSP00000409581 | ARRB1 | 0.5842 |
| ENSP00000228945 | ARHGDIB | 0.5842 |
| ENSP00000310491 | ARHGAP1 | 0.5842 |
| ENSP00000220592 | AGO2 | 0.5842 |
| ENSP00000367220 | ACTR2 | 0.5842 |
| ENSP00000326630 | ZFPM1 | 0.5816 |
| ENSP00000211998 | VCL | 0.5816 |
| ENSP00000379500 | UEVLD | 0.5816 |
| ENSP00000352645 | TYW1 | 0.5816 |
| ENSP00000328973 | TSPO | 0.5816 |
| ENSP00000241261 | TNFSF10 | 0.5816 |
| ENSP00000364133 | TGFBR1 | 0.5816 |
| ENSP00000377265 | TFAP2B | 0.5816 |
| ENSP00000319756 | TENC1 | 0.5816 |
| ENSP00000265304 | SSBP1 | 0.5816 |
| ENSP00000216774 | SRP54 | 0.5816 |
| ENSP00000310301 | SP3 | 0.5816 |
| ENSP00000260653 | SIX3 | 0.5816 |
| ENSP00000247182 | SIX1 | 0.5816 |
| ENSP00000332995 | SETD8 | 0.5816 |
| ENSP00000318094 | SCMH1 | 0.5816 |
| ENSP00000433821 | RPS3 | 0.5816 |
| ENSP00000435777 | RPS13 | 0.5816 |
| ENSP00000437142 | RPP14 | 0.5816 |
| ENSP00000384892 | RIMS2 | 0.5816 |
| ENSP00000380271 | RAPGEF4 | 0.5816 |
| ENSP00000164139 | PYGM | 0.5816 |
| ENSP00000368318 | PUSL1 | 0.5816 |
| ENSP00000341189 | PTK2 | 0.5816 |
| ENSP00000268835 | PRPSAP2 | 0.5816 |
| ENSP00000302150 | PRL | 0.5816 |

| | | |
|---|---|---|
| ENSP00000336831 | PLS1 | 0.5816 |
| ENSP00000367570 | PHF11 | 0.5816 |
| ENSP00000363640 | PHF1 | 0.5816 |
| ENSP00000360154 | OCRL | 0.5816 |
| ENSP00000356331 | NR5A2 | 0.5816 |
| ENSP00000387946 | NR1H3 | 0.5816 |
| ENSP00000275015 | NFKBIE | 0.5816 |
| ENSP00000240922 | NAA50 | 0.5816 |
| ENSP00000401678 | MYOCD | 0.5816 |
| ENSP00000262269 | MYH14 | 0.5816 |
| ENSP00000226207 | MYH1 | 0.5816 |
| ENSP00000265081 | MSH3 | 0.5816 |
| ENSP00000343867 | MRRF | 0.5816 |
| ENSP00000263390 | MED26 | 0.5816 |
| ENSP00000305059 | MAP1LC3B2 | 0.5816 |
| ENSP00000356357 | LHX9 | 0.5816 |
| ENSP00000269576 | KRT10 | 0.5816 |
| ENSP00000248553 | HSPB1 | 0.5816 |
| ENSP00000420914 | HSD17B4 | 0.5816 |
| ENSP00000315949 | HOXD8 | 0.5816 |
| ENSP00000365439 | HNRNPK | 0.5816 |
| ENSP00000437409 | GRAPL | 0.5816 |
| ENSP00000314499 | GAK | 0.5816 |
| ENSP00000419266 | EVX1 | 0.5816 |
| ENSP00000340660 | ELSPBP1 | 0.5816 |
| ENSP00000312185 | ELMO1 | 0.5816 |
| ENSP00000362688 | EIF3I | 0.5816 |
| ENSP00000358140 | EIF3A | 0.5816 |
| ENSP00000319635 | CXCR2 | 0.5816 |
| ENSP00000303939 | CTLA4 | 0.5816 |
| ENSP00000353654 | COL4A2 | 0.5816 |
| ENSP00000320949 | CNOT1 | 0.5816 |
| ENSP00000245816 | CLPP | 0.5816 |
| ENSP00000283635 | CD8A | 0.5816 |
| ENSP00000332049 | CD86 | 0.5816 |
| ENSP00000262643 | CCNE1 | 0.5816 |
| ENSP00000312664 | CASP2 | 0.5816 |
| ENSP00000410076 | CASP1 | 0.5816 |
| ENSP00000356919 | ATF6 | 0.5816 |
| ENSP00000376204 | ASH1L | 0.5816 |
| ENSP00000374357 | ARNTL | 0.5816 |
| ENSP00000316845 | ARHGEF4 | 0.5816 |
| ENSP00000377233 | ARAP1 | 0.5816 |
| ENSP00000298545 | AK8 | 0.5816 |
| ENSP00000263238 | ACTR3 | 0.5816 |
| ENSP00000355537 | ACTN2 | 0.5816 |
| ENSP00000242592 | ACADS | 0.5816 |
| ENSP00000271015 | BCL10 | 0.5816 |
| ENSP00000314214 | VAMP2 | 0.5789 |
| ENSP00000302239 | USP8 | 0.5789 |
| ENSP00000320797 | TUBD1 | 0.5789 |
| ENSP00000216160 | TAB1 | 0.5789 |
| ENSP00000326261 | SRRM1 | 0.5789 |

| | | |
|---|---|---|
| ENSP00000261531 | SNW1 | 0.5789 |
| ENSP00000262613 | SLC9A3R1 | 0.5789 |
| ENSP00000267197 | SETD1B | 0.5789 |
| ENSP00000306881 | SEC23A | 0.5789 |
| ENSP00000226218 | SEBOX | 0.5789 |
| ENSP00000427015 | RPS6KA2 | 0.5789 |
| ENSP00000377640 | RPL24 | 0.5789 |
| ENSP00000055077 | RFC2 | 0.5789 |
| ENSP00000342830 | RDX | 0.5789 |
| ENSP00000310244 | RASGRP1 | 0.5789 |
| ENSP00000309871 | RACGAP1 | 0.5789 |
| ENSP00000447300 | RAB3IP | 0.5789 |
| ENSP00000262193 | PSMB1 | 0.5789 |
| ENSP00000265563 | PRKAR2A | 0.5789 |
| ENSP00000384610 | PNPLA7 | 0.5789 |
| ENSP00000265340 | PITX1 | 0.5789 |
| ENSP00000378307 | PBRM1 | 0.5789 |
| ENSP00000250416 | PARP2 | 0.5789 |
| ENSP00000219255 | PARD6A | 0.5789 |
| ENSP00000222673 | OGDH | 0.5789 |
| ENSP00000007516 | NDUFAB1 | 0.5789 |
| ENSP00000300119 | MYO1A | 0.5789 |
| ENSP00000263205 | MED15 | 0.5789 |
| ENSP00000229854 | MCM3 | 0.5789 |
| ENSP00000262948 | MAP2K2 | 0.5789 |
| ENSP00000294816 | LMX1A | 0.5789 |
| ENSP00000302100 | LGALS4 | 0.5789 |
| ENSP00000265537 | LARS2 | 0.5789 |
| ENSP00000307156 | LAMB2 | 0.5789 |
| ENSP00000305702 | KCTD19 | 0.5789 |
| ENSP00000380073 | IRF9 | 0.5789 |
| ENSP00000329411 | IRF7 | 0.5789 |
| ENSP00000275525 | IGFBP1 | 0.5789 |
| ENSP00000219700 | HMOX2 | 0.5789 |
| ENSP00000430432 | HDAC2 | 0.5789 |
| ENSP00000282561 | GJA1 | 0.5789 |
| ENSP00000304286 | FOXI1 | 0.5789 |
| ENSP00000370256 | FOXC1 | 0.5789 |
| ENSP00000347294 | EYA4 | 0.5789 |
| ENSP00000340691 | EIF4EBP1 | 0.5789 |
| ENSP00000310040 | EIF3F | 0.5789 |
| ENSP00000320516 | EHD1 | 0.5789 |
| ENSP00000257600 | DTX1 | 0.5789 |
| ENSP00000383178 | DIAPH3 | 0.5789 |
| ENSP00000362687 | DDX50 | 0.5789 |
| ENSP00000418191 | CTNNA2 | 0.5789 |
| ENSP00000297848 | COL14A1 | 0.5789 |
| ENSP00000448012 | CEP290 | 0.5789 |
| ENSP00000360532 | CDC5L | 0.5789 |
| ENSP00000405726 | CDC45 | 0.5789 |
| ENSP00000339191 | CAV1 | 0.5789 |
| ENSP00000311032 | CASP3 | 0.5789 |
| ENSP00000381412 | CAMK2A | 0.5789 |

| | | |
|---|---|---|
| ENSP00000216911 | AURKA | 0.5789 |
| ENSP00000262030 | ATP5B | 0.5789 |
| ENSP00000364839 | ASXL1 | 0.5789 |
| ENSP00000262942 | ARPC1A | 0.5789 |
| ENSP00000354837 | ARHGEF2 | 0.5789 |
| ENSP00000255194 | AP3B1 | 0.5789 |
| ENSP00000264448 | ALMS1 | 0.5789 |
| ENSP00000262713 | AJUBA | 0.5789 |
| ENSP00000224764 | BMPR1A | 0.5789 |
| ENSP00000265428 | WWP1 | 0.5763 |
| ENSP00000440586 | USO1 | 0.5763 |
| ENSP00000247668 | TRAF2 | 0.5763 |
| ENSP00000286827 | TIAM1 | 0.5763 |
| ENSP00000364519 | TFDP1 | 0.5763 |
| ENSP00000296702 | TCERG1 | 0.5763 |
| ENSP00000343515 | TCEA2 | 0.5763 |
| ENSP00000247219 | TBPL2 | 0.5763 |
| ENSP00000348128 | SVIL | 0.5763 |
| ENSP00000368831 | SNX2 | 0.5763 |
| ENSP00000298532 | SNAPC4 | 0.5763 |
| ENSP00000262158 | SMAD7 | 0.5763 |
| ENSP00000298428 | SEC61A2 | 0.5763 |
| ENSP00000464383 | RP11-599B13.6 | 0.5763 |
| ENSP00000220751 | RIPK2 | 0.5763 |
| ENSP00000311747 | RBM14 | 0.5763 |
| ENSP00000247470 | PYCARD | 0.5763 |
| ENSP00000228307 | PXN | 0.5763 |
| ENSP00000381504 | PRPS2 | 0.5763 |
| ENSP00000351686 | PRDM10 | 0.5763 |
| ENSP00000221859 | POLR2I | 0.5763 |
| ENSP00000262741 | PIK3R3 | 0.5763 |
| ENSP00000420294 | PHC3 | 0.5763 |
| ENSP00000252891 | NUMBL | 0.5763 |
| ENSP00000385142 | NRXN1 | 0.5763 |
| ENSP00000312988 | NFKBIB | 0.5763 |
| ENSP00000355966 | NEK2 | 0.5763 |
| ENSP00000380334 | NCF4 | 0.5763 |
| ENSP00000354554 | MT-CYB | 0.5763 |
| ENSP00000233146 | MSH2 | 0.5763 |
| ENSP00000305682 | MRPL39 | 0.5763 |
| ENSP00000298048 | MELK | 0.5763 |
| ENSP00000333685 | MAPK11 | 0.5763 |
| ENSP00000351908 | MAP3K5 | 0.5763 |
| ENSP00000366330 | KLF9 | 0.5763 |
| ENSP00000441691 | ITGAM | 0.5763 |
| ENSP00000404121 | ILF3 | 0.5763 |
| ENSP00000347184 | HTT | 0.5763 |
| ENSP00000225648 | HOXB6 | 0.5763 |
| ENSP00000319690 | HNRNPC | 0.5763 |
| ENSP00000216117 | HMOX1 | 0.5763 |
| ENSP00000369871 | HAUS6 | 0.5763 |
| ENSP00000302728 | GUSB | 0.5763 |
| ENSP00000362924 | GSN | 0.5763 |

| | | |
|---|---|---|
| ENSP00000238558 | GSC | 0.5763 |
| ENSP00000323880 | FOXJ1 | 0.5763 |
| ENSP00000311697 | FGF5 | 0.5763 |
| ENSP00000262464 | FBN2 | 0.5763 |
| ENSP00000333640 | EYA2 | 0.5763 |
| ENSP00000234198 | DLX2 | 0.5763 |
| ENSP00000417078 | DHX36 | 0.5763 |
| ENSP00000236671 | CTSD | 0.5763 |
| ENSP00000361290 | COL4A6 | 0.5763 |
| ENSP00000356623 | CITED2 | 0.5763 |
| ENSP00000262127 | CEP76 | 0.5763 |
| ENSP00000354782 | CD247 | 0.5763 |
| ENSP00000349238 | CCDC88B | 0.5763 |
| ENSP00000384490 | CBX6 | 0.5763 |
| ENSP00000209875 | CBX5 | 0.5763 |
| ENSP00000319060 | CAMK2G | 0.5763 |
| ENSP00000287598 | BUB1B | 0.5763 |
| ENSP00000267953 | BCL2A1 | 0.5763 |
| ENSP00000396954 | ATF5 | 0.5763 |
| ENSP00000252725 | ARPC1B | 0.5763 |
| ENSP00000362442 | ARHGAP40 | 0.5763 |
| ENSP00000378152 | AKTIP | 0.5763 |
| ENSP00000410186 | AK9 | 0.5763 |
| ENSP00000340361 | ACVR2B | 0.5763 |
| ENSP00000351777 | VCP | 0.5737 |
| ENSP00000261965 | TUBGCP3 | 0.5737 |
| ENSP00000343764 | TTN | 0.5737 |
| ENSP00000254436 | TRIM21 | 0.5737 |
| ENSP00000356286 | TNNT2 | 0.5737 |
| ENSP00000398698 | TNF | 0.5737 |
| ENSP00000355471 | TFB2M | 0.5737 |
| ENSP00000431376 | TATDN3 | 0.5737 |
| ENSP00000265112 | TARS | 0.5737 |
| ENSP00000372547 | SRY | 0.5737 |
| ENSP00000270142 | SOD1 | 0.5737 |
| ENSP00000243563 | SNRPA | 0.5737 |
| ENSP00000370083 | SMN1 | 0.5737 |
| ENSP00000254480 | SMARCC1 | 0.5737 |
| ENSP00000331019 | RPS27L | 0.5737 |
| ENSP00000359685 | PTP4A1 | 0.5737 |
| ENSP00000359719 | PRKACB | 0.5737 |
| ENSP00000378306 | PPP3CB | 0.5737 |
| ENSP00000362329 | PPA1 | 0.5737 |
| ENSP00000362103 | POU3F1 | 0.5737 |
| ENSP00000339826 | PIK3AP1 | 0.5737 |
| ENSP00000313490 | PFAS | 0.5737 |
| ENSP00000253754 | PDLIM4 | 0.5737 |
| ENSP00000368438 | PCNA | 0.5737 |
| ENSP00000325421 | PARL | 0.5737 |
| ENSP00000217073 | PABPC1L | 0.5737 |
| ENSP00000305503 | NUP62 | 0.5737 |
| ENSP00000240423 | NCAPH | 0.5737 |
| ENSP00000261681 | MPP5 | 0.5737 |

| | | |
|---|---|---|
| ENSP00000203630 | MLF2 | 0.5737 |
| ENSP00000229794 | MAPK14 | 0.5737 |
| ENSP00000381066 | MAP2K7 | 0.5737 |
| ENSP00000292599 | MAML1 | 0.5737 |
| ENSP00000333298 | LAMP1 | 0.5737 |
| ENSP00000324532 | LAMA3 | 0.5737 |
| ENSP00000321584 | IMPDH2 | 0.5737 |
| ENSP00000299421 | ILK | 0.5737 |
| ENSP00000264563 | IL11 | 0.5737 |
| ENSP00000349437 | IGF2R | 0.5737 |
| ENSP00000367972 | ID4 | 0.5737 |
| ENSP00000364114 | HLA-DRB5 | 0.5737 |
| ENSP00000260983 | HECW2 | 0.5737 |
| ENSP00000216341 | GZMB | 0.5737 |
| ENSP00000251337 | GNAT2 | 0.5737 |
| ENSP00000314508 | GBA | 0.5737 |
| ENSP00000372326 | FECH | 0.5737 |
| ENSP00000353564 | EXOC8 | 0.5737 |
| ENSP00000272427 | EXOC6B | 0.5737 |
| ENSP00000323377 | EXOC3 | 0.5737 |
| ENSP00000451080 | ERH | 0.5737 |
| ENSP00000265720 | DUS4L | 0.5737 |
| ENSP00000309539 | DPYSL2 | 0.5737 |
| ENSP00000333363 | DNAH8 | 0.5737 |
| ENSP00000276297 | DLC1 | 0.5737 |
| ENSP00000321711 | DIS3L | 0.5737 |
| ENSP00000356520 | DHX9 | 0.5737 |
| ENSP00000389140 | DCC | 0.5737 |
| ENSP00000350359 | CREB5 | 0.5737 |
| ENSP00000393912 | COPG2 | 0.5737 |
| ENSP00000253233 | C12orf65 | 0.5737 |
| ENSP00000305714 | BMP1 | 0.5737 |
| ENSP00000301633 | BIRC5 | 0.5737 |
| ENSP00000353878 | BAK1 | 0.5737 |
| ENSP00000260952 | ASNSD1 | 0.5737 |
| ENSP00000000233 | ARF5 | 0.5737 |
| ENSP00000263270 | AP2S1 | 0.5737 |
| ENSP00000388996 | AP1M1 | 0.5737 |
| ENSP00000382120 | AIFM3 | 0.5737 |
| ENSP00000261017 | ABI2 | 0.5737 |
| ENSP00000306983 | YEATS2 | 0.5711 |
| ENSP00000352257 | XRCC6 | 0.5711 |
| ENSP00000353847 | WWTR1 | 0.5711 |
| ENSP00000369531 | UBQLNL | 0.5711 |
| ENSP00000376500 | TRAF3 | 0.5711 |
| ENSP00000362994 | TRAF1 | 0.5711 |
| ENSP00000349705 | TOP3B | 0.5711 |
| ENSP00000321636 | TOP3A | 0.5711 |
| ENSP00000438262 | TJP2 | 0.5711 |
| ENSP00000365534 | THNSL1 | 0.5711 |
| ENSP00000221930 | TGFB1 | 0.5711 |
| ENSP00000356134 | TFB1M | 0.5711 |
| ENSP00000313752 | SSNA1 | 0.5711 |

| | | |
|---|---|---|
| ENSP00000362900 | SRSF4 | 0.5711 |
| ENSP00000216373 | SOS2 | 0.5711 |
| ENSP00000355136 | SORBS1 | 0.5711 |
| ENSP00000377409 | SFTPB | 0.5711 |
| ENSP00000383145 | SFI1 | 0.5711 |
| ENSP00000246868 | SBDS | 0.5711 |
| ENSP00000225577 | RPS6KB1 | 0.5711 |
| ENSP00000296674 | RPS23 | 0.5711 |
| ENSP00000339027 | RPLP0 | 0.5711 |
| ENSP00000338671 | RHOBTB1 | 0.5711 |
| ENSP00000362592 | RBBP4 | 0.5711 |
| ENSP00000304447 | PTCRA | 0.5711 |
| ENSP00000353415 | PRKAR1B | 0.5711 |
| ENSP00000012443 | PPP5C | 0.5711 |
| ENSP00000221138 | PPP2CB | 0.5711 |
| ENSP00000303754 | PPID | 0.5711 |
| ENSP00000310928 | PPARD | 0.5711 |
| ENSP00000281321 | POU4F2 | 0.5711 |
| ENSP00000347345 | POLR3H | 0.5711 |
| ENSP00000368645 | POLR2J3 | 0.5711 |
| ENSP00000348163 | PLS3 | 0.5711 |
| ENSP00000392812 | PIK3R5 | 0.5711 |
| ENSP00000262971 | PIAS4 | 0.5711 |
| ENSP00000451040 | PGF | 0.5711 |
| ENSP00000257770 | NT5E | 0.5711 |
| ENSP00000300659 | NFATC3 | 0.5711 |
| ENSP00000242462 | NEUROG3 | 0.5711 |
| ENSP00000318472 | NCAM1 | 0.5711 |
| ENSP00000341992 | NANOS3 | 0.5711 |
| ENSP00000377867 | MST4 | 0.5711 |
| ENSP00000453793 | MEIS2 | 0.5711 |
| ENSP00000382423 | MAP3K1 | 0.5711 |
| ENSP00000302830 | MAEA | 0.5711 |
| ENSP00000359573 | LMO4 | 0.5711 |
| ENSP00000366915 | KLF5 | 0.5711 |
| ENSP00000266041 | ITIH4 | 0.5711 |
| ENSP00000303242 | ITGB2 | 0.5711 |
| ENSP00000231449 | IL4 | 0.5711 |
| ENSP00000431512 | HSF1 | 0.5711 |
| ENSP00000320176 | HCLS1 | 0.5711 |
| ENSP00000309555 | HCFC1 | 0.5711 |
| ENSP00000230771 | HARS2 | 0.5711 |
| ENSP00000352565 | HAND2 | 0.5711 |
| ENSP00000370532 | GTF3A | 0.5711 |
| ENSP00000341247 | GSPT2 | 0.5711 |
| ENSP00000381793 | GRB10 | 0.5711 |
| ENSP00000301838 | FADD | 0.5711 |
| ENSP00000367992 | ESD | 0.5711 |
| ENSP00000355890 | EPRS | 0.5711 |
| ENSP00000242577 | DYNLL1 | 0.5711 |
| ENSP00000346120 | DDX21 | 0.5711 |
| ENSP00000255448 | DCLK1 | 0.5711 |
| ENSP00000358695 | CYB5R4 | 0.5711 |

| | | |
|---|---|---|
| ENSP00000255641 | CSNK1G2 | 0.5711 |
| ENSP00000342056 | CS | 0.5711 |
| ENSP00000271889 | CREB3L4 | 0.5711 |
| ENSP00000305613 | CPLX1 | 0.5711 |
| ENSP00000240316 | COIL | 0.5711 |
| ENSP00000294053 | CLPB | 0.5711 |
| ENSP00000299198 | CKB | 0.5711 |
| ENSP00000380661 | CEP250 | 0.5711 |
| ENSP00000226279 | CD38 | 0.5711 |
| ENSP00000282356 | CAMK4 | 0.5711 |
| ENSP00000250244 | AP1M2 | 0.5711 |
| ENSP00000331268 | ANKRD45 | 0.5711 |
| ENSP00000217426 | AHCY | 0.5711 |
| ENSP00000368678 | AGRN | 0.5711 |
| ENSP00000349297 | ACADVL | 0.5711 |
| ENSP00000354945 | MCM10 | 0.5711 |
| ENSP00000365514 | ZIC2 | 0.5684 |
| ENSP00000284273 | UBASH3B | 0.5684 |
| ENSP00000354340 | UBA3 | 0.5684 |
| ENSP00000336712 | TNPO1 | 0.5684 |
| ENSP00000365048 | TNFSF13B | 0.5684 |
| ENSP00000233156 | TFPI | 0.5684 |
| ENSP00000204517 | TFAP4 | 0.5684 |
| ENSP00000237316 | TCF21 | 0.5684 |
| ENSP00000261716 | TAOK1 | 0.5684 |
| ENSP00000286332 | TAB2 | 0.5684 |
| ENSP00000261205 | SYT1 | 0.5684 |
| ENSP00000222812 | STX1A | 0.5684 |
| ENSP00000328023 | SRPR | 0.5684 |
| ENSP00000354476 | SREBF2 | 0.5684 |
| ENSP00000343785 | SPRY1 | 0.5684 |
| ENSP00000265773 | SMARCA2 | 0.5684 |
| ENSP00000416293 | SLC2A1 | 0.5684 |
| ENSP00000228318 | SLC25A3 | 0.5684 |
| ENSP00000345492 | SH2B3 | 0.5684 |
| ENSP00000340608 | SGK2 | 0.5684 |
| ENSP00000386759 | SETD2 | 0.5684 |
| ENSP00000243253 | SEC61A1 | 0.5684 |
| ENSP00000262544 | SEC23B | 0.5684 |
| ENSP00000346598 | RPSAP58 | 0.5684 |
| ENSP00000311028 | RPS14 | 0.5684 |
| ENSP00000261523 | RORA | 0.5684 |
| ENSP00000342502 | PYCR2 | 0.5684 |
| ENSP00000310129 | PSMD2 | 0.5684 |
| ENSP00000229328 | PRKAB1 | 0.5684 |
| ENSP00000419970 | POR | 0.5684 |
| ENSP00000264708 | POMC | 0.5684 |
| ENSP00000243924 | PI3 | 0.5684 |
| ENSP00000352438 | PCBP2 | 0.5684 |
| ENSP00000363921 | PARD3 | 0.5684 |
| ENSP00000302886 | PA2G4 | 0.5684 |
| ENSP00000458954 | NUP88 | 0.5684 |
| ENSP00000242210 | NT5C3A | 0.5684 |

| | | |
|---|---|---|
| ENSP00000274137 | NDUFS6 | 0.5684 |
| ENSP00000345892 | NDE1 | 0.5684 |
| ENSP00000222553 | NAMPT | 0.5684 |
| ENSP00000326424 | NADSYN1 | 0.5684 |
| ENSP00000354499 | MT-CO1 | 0.5684 |
| ENSP00000354927 | MAP3K3 | 0.5684 |
| ENSP00000345083 | MAP2K3 | 0.5684 |
| ENSP00000421180 | MAML3 | 0.5684 |
| ENSP00000360525 | MAGOH | 0.5684 |
| ENSP00000310596 | LSM1 | 0.5684 |
| ENSP00000263816 | LRP2 | 0.5684 |
| ENSP00000273317 | LIMD1 | 0.5684 |
| ENSP00000332455 | KPNA2 | 0.5684 |
| ENSP00000297792 | KDM1B | 0.5684 |
| ENSP00000331608 | IRX3 | 0.5684 |
| ENSP00000305973 | HOXC4 | 0.5684 |
| ENSP00000221419 | HNRNPL | 0.5684 |
| ENSP00000296503 | HMGB2 | 0.5684 |
| ENSP00000419038 | GFM1 | 0.5684 |
| ENSP00000299293 | FRS2 | 0.5684 |
| ENSP00000371138 | FKBP1A | 0.5684 |
| ENSP00000370270 | ESRRB | 0.5684 |
| ENSP00000256797 | ERN2 | 0.5684 |
| ENSP00000364119 | EIF2S2 | 0.5684 |
| ENSP00000239223 | DUSP1 | 0.5684 |
| ENSP00000341680 | DTNBP1 | 0.5684 |
| ENSP00000330460 | DDX59 | 0.5684 |
| ENSP00000233084 | DDX1 | 0.5684 |
| ENSP00000305651 | CXCL10 | 0.5684 |
| ENSP00000008527 | CRY1 | 0.5684 |
| ENSP00000434939 | CREB3L1 | 0.5684 |
| ENSP00000304102 | COPS6 | 0.5684 |
| ENSP00000429824 | CLINT1 | 0.5684 |
| ENSP00000312455 | CFLAR | 0.5684 |
| ENSP00000256442 | CCNB1 | 0.5684 |
| ENSP00000225245 | CCL3 | 0.5684 |
| ENSP00000269397 | CBX4 | 0.5684 |
| ENSP00000302530 | BUB1 | 0.5684 |
| ENSP00000307208 | BPTF | 0.5684 |
| ENSP00000357625 | BNIP3 | 0.5684 |
| ENSP00000367705 | BCOR | 0.5684 |
| ENSP00000302898 | AURKC | 0.5684 |
| ENSP00000463999 | AURKB | 0.5684 |
| ENSP00000234396 | ATP6V1B1 | 0.5684 |
| ENSP00000391447 | ASXL2 | 0.5684 |
| ENSP00000228825 | ARPC3 | 0.5684 |
| ENSP00000349250 | ARL4A | 0.5684 |
| ENSP00000392760 | ARHGAP11B | 0.5684 |
| ENSP00000367615 | APRT | 0.5684 |
| ENSP00000349588 | ANK2 | 0.5684 |
| ENSP00000241416 | ACVR2A | 0.5684 |
| ENSP00000248673 | ZFP36 | 0.5658 |
| ENSP00000403397 | ZDHHC17 | 0.5658 |

| | | |
|---|---|---|
| ENSP00000161863 | YTHDC2 | 0.5658 |
| ENSP00000335055 | WASF3 | 0.5658 |
| ENSP00000441000 | VARS2 | 0.5658 |
| ENSP00000324897 | UBE2I | 0.5658 |
| ENSP00000381717 | UBE2D2 | 0.5658 |
| ENSP00000388107 | UBA52 | 0.5658 |
| ENSP00000252936 | TUBGCP2 | 0.5658 |
| ENSP00000251968 | TSG101 | 0.5658 |
| ENSP00000313877 | TSFM | 0.5658 |
| ENSP00000321810 | TRIT1 | 0.5658 |
| ENSP00000355208 | TPTE | 0.5658 |
| ENSP00000358854 | TAF5 | 0.5658 |
| ENSP00000343023 | SP100 | 0.5658 |
| ENSP00000283131 | SMARCA5 | 0.5658 |
| ENSP00000328596 | SIX6 | 0.5658 |
| ENSP00000353622 | SIN3A | 0.5658 |
| ENSP00000318868 | SHMT1 | 0.5658 |
| ENSP00000326477 | SETDB2 | 0.5658 |
| ENSP00000331736 | SELE | 0.5658 |
| ENSP00000427926 | RHOBTB2 | 0.5658 |
| ENSP00000306124 | PRKCE | 0.5658 |
| ENSP00000378323 | PPP3CA | 0.5658 |
| ENSP00000360305 | PDLIM1 | 0.5658 |
| ENSP00000381740 | PARP3 | 0.5658 |
| ENSP00000281589 | PABPC3 | 0.5658 |
| ENSP00000340523 | NMNAT3 | 0.5658 |
| ENSP00000383199 | NEDD4L | 0.5658 |
| ENSP00000261839 | MYO5C | 0.5658 |
| ENSP00000354558 | MTOR | 0.5658 |
| ENSP00000308845 | MRPS12 | 0.5658 |
| ENSP00000306548 | MRPL13 | 0.5658 |
| ENSP00000282486 | MBNL1 | 0.5658 |
| ENSP00000263025 | MAPK3 | 0.5658 |
| ENSP00000468348 | MAP2K6 | 0.5658 |
| ENSP00000268607 | MAP1LC3B | 0.5658 |
| ENSP00000360811 | LHX3 | 0.5658 |
| ENSP00000299601 | LEO1 | 0.5658 |
| ENSP00000252999 | LAMA5 | 0.5658 |
| ENSP00000328169 | JAG2 | 0.5658 |
| ENSP00000303351 | ITGB1 | 0.5658 |
| ENSP00000293379 | ITGA5 | 0.5658 |
| ENSP00000340737 | INTS12 | 0.5658 |
| ENSP00000231454 | IL5 | 0.5658 |
| ENSP00000358622 | IKBKG | 0.5658 |
| ENSP00000366005 | HLA-A | 0.5658 |
| ENSP00000385571 | HIPK2 | 0.5658 |
| ENSP00000296518 | GUCY1A3 | 0.5658 |
| ENSP00000222330 | GSK3A | 0.5658 |
| ENSP00000381098 | GRIP1 | 0.5658 |
| ENSP00000416097 | GOLGA2 | 0.5658 |
| ENSP00000341344 | GGA1 | 0.5658 |
| ENSP00000223366 | GCK | 0.5658 |
| ENSP00000359000 | GBF1 | 0.5658 |

| | | |
|---|---|---|
| ENSP00000451792 | GALT | 0.5658 |
| ENSP00000324573 | FLII | 0.5658 |
| ENSP00000380453 | ERMN | 0.5658 |
| ENSP00000350265 | ENPP3 | 0.5658 |
| ENSP00000263791 | EIF2AK4 | 0.5658 |
| ENSP00000232905 | EIF1B | 0.5658 |
| ENSP00000350876 | DNM3 | 0.5658 |
| ENSP00000450399 | DNM1L | 0.5658 |
| ENSP00000379140 | CXCL12 | 0.5658 |
| ENSP00000345344 | CTSL1 | 0.5658 |
| ENSP00000298248 | CRYL1 | 0.5658 |
| ENSP00000308741 | CLOCK | 0.5658 |
| ENSP00000295887 | CDS1 | 0.5658 |
| ENSP00000261211 | CDK17 | 0.5658 |
| ENSP00000264012 | CDH3 | 0.5658 |
| ENSP00000257857 | CD63 | 0.5658 |
| ENSP00000342681 | CD5 | 0.5658 |
| ENSP00000357858 | BUB3 | 0.5658 |
| ENSP00000357255 | BGLAP | 0.5658 |
| ENSP00000284727 | ATP5G3 | 0.5658 |
| ENSP00000327214 | ATP13A2 | 0.5658 |
| ENSP00000281419 | ASAP2 | 0.5658 |
| ENSP00000307479 | ARNT2 | 0.5658 |
| ENSP00000337722 | ARL6 | 0.5658 |
| ENSP00000385720 | ARHGAP35 | 0.5658 |
| ENSP00000432016 | ANKHD1-EIF4EBP3 | 0.5658 |
| ENSP00000378191 | AIMP1 | 0.5658 |
| ENSP00000272065 | ACP1 | 0.5658 |
| ENSP00000354487 | ZEB1 | 0.5632 |
| ENSP00000268616 | ZCCHC14 | 0.5632 |
| ENSP00000306330 | YWHAG | 0.5632 |
| ENSP00000282441 | YAP1 | 0.5632 |
| ENSP00000270288 | WTIP | 0.5632 |
| ENSP00000224237 | VIM | 0.5632 |
| ENSP00000306397 | UQCRFS1 | 0.5632 |
| ENSP00000316176 | UBE2N | 0.5632 |
| ENSP00000262629 | TYROBP | 0.5632 |
| ENSP00000336799 | TUBA1B | 0.5632 |
| ENSP00000318944 | TRAF7 | 0.5632 |
| ENSP00000261652 | TNFRSF13B | 0.5632 |
| ENSP00000262306 | TCEB2 | 0.5632 |
| ENSP00000177694 | TBX21 | 0.5632 |
| ENSP00000361824 | SPTAN1 | 0.5632 |
| ENSP00000218867 | SGCG | 0.5632 |
| ENSP00000410715 | SFRP4 | 0.5632 |
| ENSP00000350894 | SERPINH1 | 0.5632 |
| ENSP00000347329 | SEC31A | 0.5632 |
| ENSP00000252785 | SCO2 | 0.5632 |
| ENSP00000369757 | RPS6 | 0.5632 |
| ENSP00000429374 | RPS20 | 0.5632 |
| ENSP00000389103 | RPL23A | 0.5632 |
| ENSP00000420321 | ROBO1 | 0.5632 |
| ENSP00000310406 | RIN1 | 0.5632 |

| | | |
|---|---|---|
| ENSP00000323050 | RBBP8 | 0.5632 |
| ENSP00000419471 | RAD51B | 0.5632 |
| ENSP00000328858 | PYCR1 | 0.5632 |
| ENSP00000365687 | PTF1A | 0.5632 |
| ENSP00000346255 | PPTC7 | 0.5632 |
| ENSP00000336591 | PPP2R2B | 0.5632 |
| ENSP00000375557 | POU5F1B | 0.5632 |
| ENSP00000355001 | POU3F3 | 0.5632 |
| ENSP00000377061 | PLXNA1 | 0.5632 |
| ENSP00000262304 | PKD1 | 0.5632 |
| ENSP00000261313 | PEBP1 | 0.5632 |
| ENSP00000313007 | PABPC1 | 0.5632 |
| ENSP00000196371 | OXCT1 | 0.5632 |
| ENSP00000282549 | OTX1 | 0.5632 |
| ENSP00000262551 | OGN | 0.5632 |
| ENSP00000344314 | OFD1 | 0.5632 |
| ENSP00000254508 | NUP210 | 0.5632 |
| ENSP00000349275 | NRG1 | 0.5632 |
| ENSP00000357982 | NR2E1 | 0.5632 |
| ENSP00000300589 | NOD2 | 0.5632 |
| ENSP00000222823 | NOD1 | 0.5632 |
| ENSP00000287713 | NMNAT2 | 0.5632 |
| ENSP00000268668 | NDUFB10 | 0.5632 |
| ENSP00000256854 | NARS | 0.5632 |
| ENSP00000333657 | MX2 | 0.5632 |
| ENSP00000236826 | MMP8 | 0.5632 |
| ENSP00000262041 | MEOX2 | 0.5632 |
| ENSP00000261267 | LYZ | 0.5632 |
| ENSP00000294304 | LRP5 | 0.5632 |
| ENSP00000347684 | LMX1B | 0.5632 |
| ENSP00000348384 | LAMB3 | 0.5632 |
| ENSP00000290158 | KPNB1 | 0.5632 |
| ENSP00000264690 | KLKB1 | 0.5632 |
| ENSP00000439065 | KLC1 | 0.5632 |
| ENSP00000408979 | KIF5A | 0.5632 |
| ENSP00000264712 | KIF3C | 0.5632 |
| ENSP00000367203 | KDM6A | 0.5632 |
| ENSP00000264741 | ITGA9 | 0.5632 |
| ENSP00000307006 | IRX2 | 0.5632 |
| ENSP00000263341 | IL1B | 0.5632 |
| ENSP00000360252 | HOOK1 | 0.5632 |
| ENSP00000363745 | HNRNPR | 0.5632 |
| ENSP00000284154 | GRAP | 0.5632 |
| ENSP00000337972 | GDPD5 | 0.5632 |
| ENSP00000373918 | GARS | 0.5632 |
| ENSP00000306866 | GABARAP | 0.5632 |
| ENSP00000373572 | FOXI2 | 0.5632 |
| ENSP00000327145 | FLNC | 0.5632 |
| ENSP00000274625 | FGF18 | 0.5632 |
| ENSP00000324105 | ENO3 | 0.5632 |
| ENSP00000348965 | DYNC1H1 | 0.5632 |
| ENSP00000316054 | DVL3 | 0.5632 |
| ENSP00000363081 | DKK1 | 0.5632 |

| | | |
|---|---|---|
| ENSP00000321348 | DIAPH2 | 0.5632 |
| ENSP00000219240 | DHODH | 0.5632 |
| ENSP00000220764 | DECR1 | 0.5632 |
| ENSP00000358716 | DDX20 | 0.5632 |
| ENSP00000364188 | DDOST | 0.5632 |
| ENSP00000312435 | DAG1 | 0.5632 |
| ENSP00000362849 | CTNNA3 | 0.5632 |
| ENSP00000446743 | CNPY2 | 0.5632 |
| ENSP00000432768 | CKAP5 | 0.5632 |
| ENSP00000455307 | CHD9 | 0.5632 |
| ENSP00000362330 | CHD6 | 0.5632 |
| ENSP00000349508 | CHD4 | 0.5632 |
| ENSP00000318486 | CDK5R1 | 0.5632 |
| ENSP00000027335 | CDH17 | 0.5632 |
| ENSP00000420514 | BAG1 | 0.5632 |
| ENSP00000295685 | ARPC2 | 0.5632 |
| ENSP00000335044 | ARID2 | 0.5632 |
| ENSP00000272102 | ARF1 | 0.5632 |
| ENSP00000261722 | AP3B2 | 0.5632 |
| ENSP00000394107 | AP000304.12 | 0.5632 |
| ENSP00000396527 | AC068533.7 | 0.5632 |
| ENSP00000323929 | A2M | 0.5632 |
| ENSP00000358202 | EMX2 | 0.5632 |
| ENSP00000322915 | ZMYM4 | 0.5605 |
| ENSP00000254037 | ZCCHC9 | 0.5605 |
| ENSP00000348129 | WDR88 | 0.5605 |
| ENSP00000216639 | VRK1 | 0.5605 |
| ENSP00000356515 | UTRN | 0.5605 |
| ENSP00000249042 | TST | 0.5605 |
| ENSP00000314733 | TOLLIP | 0.5605 |
| ENSP00000278317 | TNNT3 | 0.5605 |
| ENSP00000237289 | TNFAIP3 | 0.5605 |
| ENSP00000316029 | TLN1 | 0.5605 |
| ENSP00000237264 | TBPL1 | 0.5605 |
| ENSP00000340271 | TAF3 | 0.5605 |
| ENSP00000404029 | SUPT5H | 0.5605 |
| ENSP00000263904 | STAM2 | 0.5605 |
| ENSP00000351480 | SPECC1L | 0.5605 |
| ENSP00000373964 | SMTNL2 | 0.5605 |
| ENSP00000329466 | SIRT7 | 0.5605 |
| ENSP00000349156 | SIAH1 | 0.5605 |
| ENSP00000415786 | SERPINE2 | 0.5605 |
| ENSP00000239944 | SERP1 | 0.5605 |
| ENSP00000361818 | SDC4 | 0.5605 |
| ENSP00000307046 | SDC2 | 0.5605 |
| ENSP00000217086 | SALL4 | 0.5605 |
| ENSP00000270225 | SAE1 | 0.5605 |
| ENSP00000318297 | RUVBL1 | 0.5605 |
| ENSP00000348944 | RSPO1 | 0.5605 |
| ENSP00000261991 | RPS6KA5 | 0.5605 |
| ENSP00000333896 | RPS6KA4 | 0.5605 |
| ENSP00000363021 | RPA2 | 0.5605 |
| ENSP00000254719 | RPA1 | 0.5605 |

| | | |
|---|---|---|
| ENSP00000327025 | RORC | 0.5605 |
| ENSP00000265080 | RASGRF2 | 0.5605 |
| ENSP00000222008 | RABAC1 | 0.5605 |
| ENSP00000307567 | QARS | 0.5605 |
| ENSP00000264639 | PSMD3 | 0.5605 |
| ENSP00000414359 | PSMA1 | 0.5605 |
| ENSP00000293860 | POLR3K | 0.5605 |
| ENSP00000263088 | PLD2 | 0.5605 |
| ENSP00000342793 | PLD1 | 0.5605 |
| ENSP00000260402 | PLCB2 | 0.5605 |
| ENSP00000320171 | PKM | 0.5605 |
| ENSP00000358417 | PHGDH | 0.5605 |
| ENSP00000219406 | PDIA2 | 0.5605 |
| ENSP00000401721 | PAN2 | 0.5605 |
| ENSP00000347710 | OPHN1 | 0.5605 |
| ENSP00000371785 | OLIG1 | 0.5605 |
| ENSP00000397297 | NTF3 | 0.5605 |
| ENSP00000381293 | NSF | 0.5605 |
| ENSP00000278886 | NINL | 0.5605 |
| ENSP00000317333 | NEUROG2 | 0.5605 |
| ENSP00000470609 | NDUFA7 | 0.5605 |
| ENSP00000364289 | NCBP1 | 0.5605 |
| ENSP00000325017 | NCAPD2 | 0.5605 |
| ENSP00000363571 | MUSK | 0.5605 |
| ENSP00000368790 | MTRF1 | 0.5605 |
| ENSP00000382250 | MRPS6 | 0.5605 |
| ENSP00000361206 | MRPS18A | 0.5605 |
| ENSP00000359818 | MMS19 | 0.5605 |
| ENSP00000211287 | MAPK13 | 0.5605 |
| ENSP00000314363 | MAP4K4 | 0.5605 |
| ENSP00000368020 | KIF3A | 0.5605 |
| ENSP00000366271 | KDM2B | 0.5605 |
| ENSP00000337746 | ISYNA1 | 0.5605 |
| ENSP00000345096 | IMPDH1 | 0.5605 |
| ENSP00000278200 | IMMP1L | 0.5605 |
| ENSP00000340019 | HSPD1 | 0.5605 |
| ENSP00000343040 | HMGB1 | 0.5605 |
| ENSP00000080059 | HDAC7 | 0.5605 |
| ENSP00000230990 | HBEGF | 0.5605 |
| ENSP00000313432 | GRHPR | 0.5605 |
| ENSP00000245541 | GGA3 | 0.5605 |
| ENSP00000365145 | FOXS1 | 0.5605 |
| ENSP00000296641 | F2RL2 | 0.5605 |
| ENSP00000346173 | ETFB | 0.5605 |
| ENSP00000363763 | EPHB2 | 0.5605 |
| ENSP00000381097 | EPHB1 | 0.5605 |
| ENSP00000360250 | ENTPD1 | 0.5605 |
| ENSP00000265753 | EIF4H | 0.5605 |
| ENSP00000370936 | E2F6 | 0.5605 |
| ENSP00000342105 | DYRK2 | 0.5605 |
| ENSP00000216024 | DMC1 | 0.5605 |
| ENSP00000366997 | DIS3 | 0.5605 |
| ENSP00000353401 | CUX1 | 0.5605 |

| | | |
|---|---|---|
| ENSP00000216336 | CTSG | 0.5605 |
| ENSP00000227266 | CTSC | 0.5605 |
| ENSP00000262506 | CSNK2A2 | 0.5605 |
| ENSP00000345412 | CPSF7 | 0.5605 |
| ENSP00000457881 | CPEB1 | 0.5605 |
| ENSP00000261951 | CNOT6 | 0.5605 |
| ENSP00000340954 | CNBD2 | 0.5605 |
| ENSP00000256722 | CMPK2 | 0.5605 |
| ENSP00000358262 | CHD1L | 0.5605 |
| ENSP00000273853 | CENPC1 | 0.5605 |
| ENSP00000261769 | CDH1 | 0.5605 |
| ENSP00000209728 | CDC6 | 0.5605 |
| ENSP00000312027 | CD7 | 0.5605 |
| ENSP00000250092 | CD68 | 0.5605 |
| ENSP00000299300 | CCT2 | 0.5605 |
| ENSP00000291295 | CALM3 | 0.5605 |
| ENSP00000369965 | BTF3 | 0.5605 |
| ENSP00000448323 | BRF1 | 0.5605 |
| ENSP00000216267 | BRD1 | 0.5605 |
| ENSP00000417132 | BAP1 | 0.5605 |
| ENSP00000338967 | ARHGAP6 | 0.5605 |
| ENSP00000306010 | ARF4 | 0.5605 |
| ENSP00000256658 | AP4B1 | 0.5605 |
| ENSP00000377148 | AP1G1 | 0.5605 |
| ENSP00000315931 | AHCYL2 | 0.5605 |
| ENSP00000367934 | UQCRQ | 0.5579 |
| ENSP00000284440 | UCHL1 | 0.5579 |
| ENSP00000263798 | TYRO3 | 0.5579 |
| ENSP00000259818 | TUBB2B | 0.5579 |
| ENSP00000339001 | TUBB | 0.5579 |
| ENSP00000372437 | TPTE2 | 0.5579 |
| ENSP00000367013 | TNFRSF25 | 0.5579 |
| ENSP00000291527 | TFF1 | 0.5579 |
| ENSP00000257915 | TFCP2 | 0.5579 |
| ENSP00000361057 | SURF4 | 0.5579 |
| ENSP00000390500 | STK3 | 0.5579 |
| ENSP00000362931 | SRPK1 | 0.5579 |
| ENSP00000453785 | SNX1 | 0.5579 |
| ENSP00000369154 | SMAD9 | 0.5579 |
| ENSP00000220772 | SFRP1 | 0.5579 |
| ENSP00000221494 | SF3A2 | 0.5579 |
| ENSP00000346067 | RPSA | 0.5579 |
| ENSP00000467676 | RPS15 | 0.5579 |
| ENSP00000352401 | RPE | 0.5579 |
| ENSP00000353030 | PTPRF | 0.5579 |
| ENSP00000271308 | PSMA5 | 0.5579 |
| ENSP00000295797 | PRKCI | 0.5579 |
| ENSP00000327255 | PPM1A | 0.5579 |
| ENSP00000431603 | POU2F2 | 0.5579 |
| ENSP00000228347 | POLR3B | 0.5579 |
| ENSP00000370377 | PITRM1 | 0.5579 |
| ENSP00000216727 | PABPN1 | 0.5579 |
| ENSP00000285848 | OXA1L | 0.5579 |

| | | |
|---|---|---|
| ENSP00000361882 | ODF2 | 0.5579 |
| ENSP00000347169 | NUMB | 0.5579 |
| ENSP00000310006 | NR1D2 | 0.5579 |
| ENSP00000366410 | NMNAT1 | 0.5579 |
| ENSP00000456832 | NDUFB8 | 0.5579 |
| ENSP00000237889 | NDUFB3 | 0.5579 |
| ENSP00000335636 | NAA20 | 0.5579 |
| ENSP00000365152 | MYLK2 | 0.5579 |
| ENSP00000383023 | MYL5 | 0.5579 |
| ENSP00000321445 | MSC | 0.5579 |
| ENSP00000361405 | MMP9 | 0.5579 |
| ENSP00000279068 | LSM14B | 0.5579 |
| ENSP00000261349 | LRP6 | 0.5579 |
| ENSP00000254457 | LHX1 | 0.5579 |
| ENSP00000445175 | LDHA | 0.5579 |
| ENSP00000293308 | KRT8 | 0.5579 |
| ENSP00000355124 | KRT19 | 0.5579 |
| ENSP00000364864 | KIF3B | 0.5579 |
| ENSP00000306565 | ISG20 | 0.5579 |
| ENSP00000377218 | IRF2 | 0.5579 |
| ENSP00000255078 | IGHMBP2 | 0.5579 |
| ENSP00000302665 | IGF1 | 0.5579 |
| ENSP00000332773 | IGDCC3 | 0.5579 |
| ENSP00000369554 | IFNA2 | 0.5579 |
| ENSP00000264832 | ICAM1 | 0.5579 |
| ENSP00000364794 | IARS | 0.5579 |
| ENSP00000450527 | HOPX | 0.5579 |
| ENSP00000316772 | HMHA1 | 0.5579 |
| ENSP00000351524 | HELZ | 0.5579 |
| ENSP00000298068 | HECTD2 | 0.5579 |
| ENSP00000325136 | HADHB | 0.5579 |
| ENSP00000370023 | HADHA | 0.5579 |
| ENSP00000296417 | H2AFZ | 0.5579 |
| ENSP00000319118 | GSX2 | 0.5579 |
| ENSP00000349305 | GOLGA4 | 0.5579 |
| ENSP00000378338 | GIT1 | 0.5579 |
| ENSP00000340698 | GIPC1 | 0.5579 |
| ENSP00000394008 | GABARAPL3 | 0.5579 |
| ENSP00000305480 | FEN1 | 0.5579 |
| ENSP00000320543 | EPN2 | 0.5579 |
| ENSP00000263277 | EHD2 | 0.5579 |
| ENSP00000245323 | EFNB2 | 0.5579 |
| ENSP00000288699 | DPYSL5 | 0.5579 |
| ENSP00000353104 | DHDDS | 0.5579 |
| ENSP00000379475 | DDX39B | 0.5579 |
| ENSP00000354791 | DCTN1 | 0.5579 |
| ENSP00000264414 | CUL3 | 0.5579 |
| ENSP00000390011 | CTU1 | 0.5579 |
| ENSP00000362063 | CSTF2 | 0.5579 |
| ENSP00000402608 | CPS1 | 0.5579 |
| ENSP00000242285 | CLTA | 0.5579 |
| ENSP00000357907 | CDK19 | 0.5579 |
| ENSP00000219789 | CDIPT | 0.5579 |

| | | |
|---|---|---|
| ENSP00000331172 | CD8B | 0.5579 |
| ENSP00000228434 | CD69 | 0.5579 |
| ENSP00000262262 | CD33 | 0.5579 |
| ENSP00000370989 | CD274 | 0.5579 |
| ENSP00000361878 | CAP1 | 0.5579 |
| ENSP00000315299 | CALML3 | 0.5579 |
| ENSP00000225698 | C1QBP | 0.5579 |
| ENSP00000358081 | BAG3 | 0.5579 |
| ENSP00000292401 | AZGP1 | 0.5579 |
| ENSP00000242839 | ATP7B | 0.5579 |
| ENSP00000431605 | ATP6V0B | 0.5579 |
| ENSP00000344546 | ARID1B | 0.5579 |
| ENSP00000357066 | ARG1 | 0.5579 |
| ENSP00000256682 | ARF3 | 0.5579 |
| ENSP00000302895 | ARAP2 | 0.5579 |
| ENSP00000358921 | ACTR1A | 0.5579 |
| ENSP00000407761 | ACOXL | 0.5579 |
| ENSP00000387356 | ACAN | 0.5579 |
| ENSP00000302501 | ZEB2 | 0.5553 |
| ENSP00000299138 | VPS35 | 0.5553 |
| ENSP00000342812 | USP9Y | 0.5553 |
| ENSP00000356425 | UCHL5 | 0.5553 |
| ENSP00000264552 | UBE2S | 0.5553 |
| ENSP00000283645 | TUBGCP5 | 0.5553 |
| ENSP00000264071 | TUBB4A | 0.5553 |
| ENSP00000298552 | TSC1 | 0.5553 |
| ENSP00000341268 | TRADD | 0.5553 |
| ENSP00000332326 | TNFAIP2 | 0.5553 |
| ENSP00000280605 | TKTL2 | 0.5553 |
| ENSP00000282111 | TCF7L1 | 0.5553 |
| ENSP00000331791 | TBX1 | 0.5553 |
| ENSP00000287652 | TATDN2 | 0.5553 |
| ENSP00000278968 | TAGLN | 0.5553 |
| ENSP00000265073 | SUB1 | 0.5553 |
| ENSP00000317714 | STX4 | 0.5553 |
| ENSP00000229812 | STK38 | 0.5553 |
| ENSP00000341815 | SOX18 | 0.5553 |
| ENSP00000357674 | SNAPIN | 0.5553 |
| ENSP00000244050 | SNAI1 | 0.5553 |
| ENSP00000340989 | SFN | 0.5553 |
| ENSP00000337053 | SEL1L | 0.5553 |
| ENSP00000359370 | SEC31B | 0.5553 |
| ENSP00000468633 | SEC11C | 0.5553 |
| ENSP00000260926 | SATB2 | 0.5553 |
| ENSP00000341885 | RPS2 | 0.5553 |
| ENSP00000393241 | RPS18 | 0.5553 |
| ENSP00000332454 | RIPK4 | 0.5553 |
| ENSP00000408295 | RFC5 | 0.5553 |
| ENSP00000370151 | RAD17 | 0.5553 |
| ENSP00000365837 | PUS1 | 0.5553 |
| ENSP00000349932 | PTPRS | 0.5553 |
| ENSP00000406162 | PRMT1 | 0.5553 |
| ENSP00000298510 | PRDX3 | 0.5553 |

| | | |
|---|---|---|
| ENSP00000263857 | POLR1A | 0.5553 |
| ENSP00000322570 | POLE | 0.5553 |
| ENSP00000354532 | PNP | 0.5553 |
| ENSP00000334105 | PLCB4 | 0.5553 |
| ENSP00000339933 | PKLR | 0.5553 |
| ENSP00000356155 | PIK3C2B | 0.5553 |
| ENSP00000348099 | PDLIM7 | 0.5553 |
| ENSP00000255266 | PDE6A | 0.5553 |
| ENSP00000380378 | PAFAH1B1 | 0.5553 |
| ENSP00000333275 | NR2C1 | 0.5553 |
| ENSP00000215565 | NDUFB7 | 0.5553 |
| ENSP00000289473 | NCF1 | 0.5553 |
| ENSP00000309597 | MAP3K11 | 0.5553 |
| ENSP00000222725 | LFNG | 0.5553 |
| ENSP00000306772 | LDB2 | 0.5553 |
| ENSP00000386918 | KDELR3 | 0.5553 |
| ENSP00000225916 | KAT2A | 0.5553 |
| ENSP00000369456 | ITPA | 0.5553 |
| ENSP00000274364 | IQGAP2 | 0.5553 |
| ENSP00000227752 | IL10RA | 0.5553 |
| ENSP00000369581 | IFNB1 | 0.5553 |
| ENSP00000334061 | HDAC6 | 0.5553 |
| ENSP00000216271 | HDAC10 | 0.5553 |
| ENSP00000308405 | H2AFV | 0.5553 |
| ENSP00000377836 | GPC3 | 0.5553 |
| ENSP00000327589 | GLUD2 | 0.5553 |
| ENSP00000296875 | GDF9 | 0.5553 |
| ENSP00000215631 | GADD45B | 0.5553 |
| ENSP00000434034 | FZD4 | 0.5553 |
| ENSP00000226247 | FOXN1 | 0.5553 |
| ENSP00000362107 | FHL3 | 0.5553 |
| ENSP00000306361 | FGA | 0.5553 |
| ENSP00000221801 | FBL | 0.5553 |
| ENSP00000359356 | EVI5 | 0.5553 |
| ENSP00000362299 | ENG | 0.5553 |
| ENSP00000263360 | EED | 0.5553 |
| ENSP00000303515 | DUS1L | 0.5553 |
| ENSP00000372005 | DNAJC19 | 0.5553 |
| ENSP00000222598 | DLX5 | 0.5553 |
| ENSP00000340292 | DLK1 | 0.5553 |
| ENSP00000381565 | DIAPH1 | 0.5553 |
| ENSP00000382840 | DDX3X | 0.5553 |
| ENSP00000355086 | DARS2 | 0.5553 |
| ENSP00000264613 | CP | 0.5553 |
| ENSP00000355500 | CEP170 | 0.5553 |
| ENSP00000357025 | CD48 | 0.5553 |
| ENSP00000367605 | CCL3L3 | 0.5553 |
| ENSP00000408984 | CCL3L1 | 0.5553 |
| ENSP00000341940 | CAV3 | 0.5553 |
| ENSP00000309595 | C10orf2 | 0.5553 |
| ENSP00000268057 | BBS4 | 0.5553 |
| ENSP00000260947 | BARD1 | 0.5553 |
| ENSP00000345728 | ATP7A | 0.5553 |

| | | |
|---|---|---|
| ENSP00000377878 | ATP5G2 | 0.5553 |
| ENSP00000322159 | ATG4C | 0.5553 |
| ENSP00000375057 | ARL4C | 0.5553 |
| ENSP00000380635 | ARL16 | 0.5553 |
| ENSP00000355090 | ARHGAP11A | 0.5553 |
| ENSP00000360985 | ARFGEF2 | 0.5553 |
| ENSP00000379891 | AP1S3 | 0.5553 |
| ENSP00000265709 | ANK1 | 0.5553 |
| ENSP00000221496 | AMH | 0.5553 |
| ENSP00000357459 | ADAR | 0.5553 |
| ENSP00000260408 | ADAM10 | 0.5553 |
| ENSP00000265724 | ABCB1 | 0.5553 |
| ENSP00000261772 | AARS | 0.5553 |
| ENSP00000360277 | DAB1 | 0.5553 |
| ENSP00000384179 | ZFPM2 | 0.5526 |
| ENSP00000262630 | ZBTB32 | 0.5526 |
| ENSP00000452549 | YJEFN3 | 0.5526 |
| ENSP00000367756 | UNC13B | 0.5526 |
| ENSP00000456648 | TUBGCP4 | 0.5526 |
| ENSP00000301071 | TUBA1A | 0.5526 |
| ENSP00000253024 | TRIM28 | 0.5526 |
| ENSP00000396704 | TOP2B | 0.5526 |
| ENSP00000355566 | TOMM20 | 0.5526 |
| ENSP00000353874 | TLR9 | 0.5526 |
| ENSP00000260010 | TLR2 | 0.5526 |
| ENSP00000385461 | TFDP3 | 0.5526 |
| ENSP00000296145 | TDGF1 | 0.5526 |
| ENSP00000338093 | TARSL2 | 0.5526 |
| ENSP00000356033 | TAGAP | 0.5526 |
| ENSP00000201586 | SULT2B1 | 0.5526 |
| ENSP00000267814 | SORD | 0.5526 |
| ENSP00000429276 | SNTG1 | 0.5526 |
| ENSP00000370808 | SLC25A6 | 0.5526 |
| ENSP00000251020 | SALL1 | 0.5526 |
| ENSP00000346050 | RPS3A | 0.5526 |
| ENSP00000367439 | RPP38 | 0.5526 |
| ENSP00000351132 | RHOT1 | 0.5526 |
| ENSP00000450244 | RASAL1 | 0.5526 |
| ENSP00000373864 | RAPGEF3 | 0.5526 |
| ENSP00000319169 | PRMT5 | 0.5526 |
| ENSP00000263431 | PRKCG | 0.5526 |
| ENSP00000408695 | PRKCA | 0.5526 |
| ENSP00000260643 | PREB | 0.5526 |
| ENSP00000262746 | PRDX1 | 0.5526 |
| ENSP00000381726 | PRAME | 0.5526 |
| ENSP00000328412 | PDE6G | 0.5526 |
| ENSP00000354033 | PCGF2 | 0.5526 |
| ENSP00000362621 | PABPC1L2B | 0.5526 |
| ENSP00000274276 | OSMR | 0.5526 |
| ENSP00000373674 | NT5M | 0.5526 |
| ENSP00000337459 | NOS1 | 0.5526 |
| ENSP00000328181 | NOG | 0.5526 |
| ENSP00000413572 | NLE1 | 0.5526 |

| | | |
|---|---|---|
| ENSP00000387219 | NFU1 | 0.5526 |
| ENSP00000325663 | NFKBIZ | 0.5526 |
| ENSP00000351015 | NF1 | 0.5526 |
| ENSP00000341021 | NANOS2 | 0.5526 |
| ENSP00000228641 | MYF6 | 0.5526 |
| ENSP00000356202 | MTRF1L | 0.5526 |
| ENSP00000264995 | MRPL3 | 0.5526 |
| ENSP00000370410 | MPDZ | 0.5526 |
| ENSP00000350028 | MOV10 | 0.5526 |
| ENSP00000438144 | MDH1 | 0.5526 |
| ENSP00000299766 | MC4R | 0.5526 |
| ENSP00000340820 | MAPT | 0.5526 |
| ENSP00000215659 | MAPK12 | 0.5526 |
| ENSP00000380066 | MAP4K1 | 0.5526 |
| ENSP00000231004 | LOX | 0.5526 |
| ENSP00000330393 | LEPR | 0.5526 |
| ENSP00000363435 | ITPR3 | 0.5526 |
| ENSP00000354451 | IQGAP3 | 0.5526 |
| ENSP00000250971 | INS | 0.5526 |
| ENSP00000170630 | IL4R | 0.5526 |
| ENSP00000296545 | IL15 | 0.5526 |
| ENSP00000231228 | IL12B | 0.5526 |
| ENSP00000303279 | IARS2 | 0.5526 |
| ENSP00000296464 | HSPA4L | 0.5526 |
| ENSP00000249440 | HOXD3 | 0.5526 |
| ENSP00000268097 | HEXA | 0.5526 |
| ENSP00000367065 | HES2 | 0.5526 |
| ENSP00000359859 | GTPBP5 | 0.5526 |
| ENSP00000222511 | GTPBP10 | 0.5526 |
| ENSP00000344173 | GRM8 | 0.5526 |
| ENSP00000251808 | GRHL2 | 0.5526 |
| ENSP00000329380 | GP1BA | 0.5526 |
| ENSP00000265070 | GOLPH3 | 0.5526 |
| ENSP00000305260 | GNB2 | 0.5526 |
| ENSP00000334051 | GNAL | 0.5526 |
| ENSP00000275364 | GNA12 | 0.5526 |
| ENSP00000276533 | GINS4 | 0.5526 |
| ENSP00000345785 | FZD9 | 0.5526 |
| ENSP00000250113 | FXR2 | 0.5526 |
| ENSP00000001008 | FKBP4 | 0.5526 |
| ENSP00000222157 | FGF21 | 0.5526 |
| ENSP00000281092 | FER | 0.5526 |
| ENSP00000357097 | FCER1A | 0.5526 |
| ENSP00000274680 | FARS2 | 0.5526 |
| ENSP00000337445 | ELAC2 | 0.5526 |
| ENSP00000368169 | DVL1 | 0.5526 |
| ENSP00000263035 | DHTKD1 | 0.5526 |
| ENSP00000265036 | DEPDC1B | 0.5526 |
| ENSP00000373715 | DCP2 | 0.5526 |
| ENSP00000350785 | DAPK1 | 0.5526 |
| ENSP00000354398 | CYTH1 | 0.5526 |
| ENSP00000259470 | CTSL2 | 0.5526 |
| ENSP00000279804 | CTF1 | 0.5526 |

| | | |
|---|---|---|
| ENSP00000369126 | CSNK1A1L | 0.5526 |
| ENSP00000246554 | COX6B1 | 0.5526 |
| ENSP00000379823 | COL4A3 | 0.5526 |
| ENSP00000347665 | COL18A1 | 0.5526 |
| ENSP00000003084 | CFTR | 0.5526 |
| ENSP00000389951 | CELF2 | 0.5526 |
| ENSP00000303706 | CDC25A | 0.5526 |
| ENSP00000011653 | CD4 | 0.5526 |
| ENSP00000324890 | CD28 | 0.5526 |
| ENSP00000292303 | CCR5 | 0.5526 |
| ENSP00000380256 | CCNF | 0.5526 |
| ENSP00000225831 | CCL2 | 0.5526 |
| ENSP00000368124 | CAMK1D | 0.5526 |
| ENSP00000432472 | BRK1 | 0.5526 |
| ENSP00000293805 | BCL6B | 0.5526 |
| ENSP00000301887 | BATF2 | 0.5526 |
| ENSP00000349142 | ATP5C1 | 0.5526 |
| ENSP00000356379 | ASPM | 0.5526 |
| ENSP00000261636 | ARL1 | 0.5526 |
| ENSP00000313506 | ARHGAP28 | 0.5526 |
| ENSP00000357218 | APOA1BP | 0.5526 |
| ENSP00000345848 | ANP32B | 0.5526 |
| ENSP00000417864 | ANP32A | 0.5526 |
| ENSP00000264276 | ALS2 | 0.5526 |
| ENSP00000360268 | ALDH18A1 | 0.5526 |
| ENSP00000346921 | AK2 | 0.5526 |
| ENSP00000355627 | AGT | 0.5526 |
| ENSP00000355493 | ADSS | 0.5526 |
| ENSP00000347498 | ZFYVE19 | 0.5500 |
| ENSP00000345633 | ZCCHC13 | 0.5500 |
| ENSP00000389381 | YY2 | 0.5500 |
| ENSP00000347858 | XIAP | 0.5500 |
| ENSP00000265107 | WDR70 | 0.5500 |
| ENSP00000325377 | WDR33 | 0.5500 |
| ENSP00000341289 | TUBB4B | 0.5500 |
| ENSP00000369703 | TUBB2A | 0.5500 |
| ENSP00000438349 | TRMT112 | 0.5500 |
| ENSP00000231524 | TRIM23 | 0.5500 |
| ENSP00000401371 | TIA1 | 0.5500 |
| ENSP00000370571 | TH | 0.5500 |
| ENSP00000296096 | TCF23 | 0.5500 |
| ENSP00000284811 | TCEB1 | 0.5500 |
| ENSP00000419325 | SUCLG2 | 0.5500 |
| ENSP00000377446 | SUCLG1 | 0.5500 |
| ENSP00000305958 | STIP1 | 0.5500 |
| ENSP00000363727 | STARD8 | 0.5500 |
| ENSP00000338785 | STARD13 | 0.5500 |
| ENSP00000418401 | SRPRB | 0.5500 |
| ENSP00000305133 | SOCS5 | 0.5500 |
| ENSP00000262188 | SMARCD3 | 0.5500 |
| ENSP00000297151 | SLU7 | 0.5500 |
| ENSP00000369131 | SIN3B | 0.5500 |
| ENSP00000356671 | SERPINC1 | 0.5500 |

| | | |
|---|---|---|
| ENSP00000236147 | SELL | 0.5500 |
| ENSP00000223641 | SEC61B | 0.5500 |
| ENSP00000364699 | SDHD | 0.5500 |
| ENSP00000251453 | RPS16 | 0.5500 |
| ENSP00000453089 | RP11-468E2.6 | 0.5500 |
| ENSP00000364860 | ROR2 | 0.5500 |
| ENSP00000306080 | RNMTL1 | 0.5500 |
| ENSP00000262940 | RASA4 | 0.5500 |
| ENSP00000414624 | PRPSAP1 | 0.5500 |
| ENSP00000227524 | PRPF19 | 0.5500 |
| ENSP00000372067 | PRMT8 | 0.5500 |
| ENSP00000377793 | PRC1 | 0.5500 |
| ENSP00000418603 | POLR2J2 | 0.5500 |
| ENSP00000462664 | POLI | 0.5500 |
| ENSP00000350132 | PLN | 0.5500 |
| ENSP00000458238 | PI4KA | 0.5500 |
| ENSP00000225655 | PFN1 | 0.5500 |
| ENSP00000340278 | PARK7 | 0.5500 |
| ENSP00000341390 | NR3C2 | 0.5500 |
| ENSP00000320447 | NR2C2 | 0.5500 |
| ENSP00000276062 | NDUFB11 | 0.5500 |
| ENSP00000360576 | NDOR1 | 0.5500 |
| ENSP00000333982 | NDEL1 | 0.5500 |
| ENSP00000314620 | MS4A1 | 0.5500 |
| ENSP00000053468 | MRPS10 | 0.5500 |
| ENSP00000322788 | MMP1 | 0.5500 |
| ENSP00000348020 | MLH3 | 0.5500 |
| ENSP00000261537 | MIB1 | 0.5500 |
| ENSP00000372025 | MBP | 0.5500 |
| ENSP00000364721 | MAPRE1 | 0.5500 |
| ENSP00000419370 | MALSU1 | 0.5500 |
| ENSP00000243077 | LRP1 | 0.5500 |
| ENSP00000261366 | LMNB1 | 0.5500 |
| ENSP00000263274 | LIG1 | 0.5500 |
| ENSP00000314837 | KLC2 | 0.5500 |
| ENSP00000381216 | KHSRP | 0.5500 |
| ENSP00000245121 | KATNAL2 | 0.5500 |
| ENSP00000306253 | ITPR1 | 0.5500 |
| ENSP00000310127 | IRF3 | 0.5500 |
| ENSP00000355011 | ILF2 | 0.5500 |
| ENSP00000247207 | HSPA2 | 0.5500 |
| ENSP00000318631 | HSD17B6 | 0.5500 |
| ENSP00000328598 | HOXD1 | 0.5500 |
| ENSP00000261416 | HEXB | 0.5500 |
| ENSP00000239027 | HELLS | 0.5500 |
| ENSP00000376609 | GRK5 | 0.5500 |
| ENSP00000354677 | GPX7 | 0.5500 |
| ENSP00000259727 | GMPR | 0.5500 |
| ENSP00000331745 | GDF3 | 0.5500 |
| ENSP00000462795 | FOXD1 | 0.5500 |
| ENSP00000331544 | FBLN1 | 0.5500 |
| ENSP00000261942 | FAF2 | 0.5500 |
| ENSP00000386171 | ESRRG | 0.5500 |

| | | |
|---|---|---|
| ENSP00000295743 | EOMES | 0.5500 |
| ENSP00000256383 | EIF2S1 | 0.5500 |
| ENSP00000351769 | DUS2L | 0.5500 |
| ENSP00000359211 | DPYD | 0.5500 |
| ENSP00000360644 | DPM1 | 0.5500 |
| ENSP00000354478 | DLX1 | 0.5500 |
| ENSP00000345731 | DLG1 | 0.5500 |
| ENSP00000355593 | DISC1 | 0.5500 |
| ENSP00000315569 | DIS3L2 | 0.5500 |
| ENSP00000233078 | DAZAP1 | 0.5500 |
| ENSP00000317159 | CYC1 | 0.5500 |
| ENSP00000367851 | CYBB | 0.5500 |
| ENSP00000295890 | COX18 | 0.5500 |
| ENSP00000264903 | CNOT6L | 0.5500 |
| ENSP00000309415 | CLTB | 0.5500 |
| ENSP00000263710 | CLASP1 | 0.5500 |
| ENSP00000388648 | CHEK1 | 0.5500 |
| ENSP00000308165 | CD36 | 0.5500 |
| ENSP00000261900 | CCNT1 | 0.5500 |
| ENSP00000288207 | CCNB2 | 0.5500 |
| ENSP00000420194 | CASR | 0.5500 |
| ENSP00000304643 | CALML6 | 0.5500 |
| ENSP00000265990 | BTAF1 | 0.5500 |
| ENSP00000379204 | BMP7 | 0.5500 |
| ENSP00000440045 | ATP2A2 | 0.5500 |
| ENSP00000352918 | ARPC5 | 0.5500 |
| ENSP00000351333 | ARHGAP19 | 0.5500 |
| ENSP00000352603 | AP4M1 | 0.5500 |
| ENSP00000285093 | ACAA2 | 0.5500 |
| ENSP00000275189 | ARHGAP18 | 0.5500 |
| ENSP00000339484 | ZFAND4 | 0.5474 |
| ENSP00000298139 | WRN | 0.5474 |
| ENSP00000351446 | WDR5 | 0.5474 |
| ENSP00000298125 | WDFY2 | 0.5474 |
| ENSP00000254950 | VPS4A | 0.5474 |
| ENSP00000414922 | USP41 | 0.5474 |
| ENSP00000294383 | USP24 | 0.5474 |
| ENSP00000246337 | UROD | 0.5474 |
| ENSP00000358813 | TTK | 0.5474 |
| ENSP00000333934 | TRMT11 | 0.5474 |
| ENSP00000368538 | TNFRSF4 | 0.5474 |
| ENSP00000414334 | TK2 | 0.5474 |
| ENSP00000338562 | STX3 | 0.5474 |
| ENSP00000359988 | SRSF11 | 0.5474 |
| ENSP00000262518 | SRCAP | 0.5474 |
| ENSP00000313681 | SPHK1 | 0.5474 |
| ENSP00000240123 | SORBS3 | 0.5474 |
| ENSP00000267064 | SMARCC2 | 0.5474 |
| ENSP00000359729 | SLC9A6 | 0.5474 |
| ENSP00000281154 | SLC25A31 | 0.5474 |
| ENSP00000348215 | SH3PXD2A | 0.5474 |
| ENSP00000274063 | SFRP2 | 0.5474 |
| ENSP00000228463 | SELPLG | 0.5474 |

| | | |
|---|---|---|
| ENSP00000381823 | SEC24A | 0.5474 |
| ENSP00000348918 | SAA1 | 0.5474 |
| ENSP00000358059 | RTN4IP1 | 0.5474 |
| ENSP00000353770 | RRM2 | 0.5474 |
| ENSP00000379888 | RPS8 | 0.5474 |
| ENSP00000321971 | RHOT2 | 0.5474 |
| ENSP00000311816 | REST | 0.5474 |
| ENSP00000459789 | RCOR1 | 0.5474 |
| ENSP00000466834 | RAD51D | 0.5474 |
| ENSP00000336701 | RAD51C | 0.5474 |
| ENSP00000372155 | PSME1 | 0.5474 |
| ENSP00000440485 | PSMD9 | 0.5474 |
| ENSP00000359910 | PSMA7 | 0.5474 |
| ENSP00000240139 | PPP3CC | 0.5474 |
| ENSP00000361446 | POLR3A | 0.5474 |
| ENSP00000262039 | PIK3C3 | 0.5474 |
| ENSP00000420295 | PDE6B | 0.5474 |
| ENSP00000258742 | NUPL2 | 0.5474 |
| ENSP00000362057 | NOX1 | 0.5474 |
| ENSP00000295886 | NKX6-1 | 0.5474 |
| ENSP00000272425 | NAT8 | 0.5474 |
| ENSP00000380444 | MYO9B | 0.5474 |
| ENSP00000228841 | MYL2 | 0.5474 |
| ENSP00000370930 | MRPL23 | 0.5474 |
| ENSP00000358547 | MPP1 | 0.5474 |
| ENSP00000308208 | MMP14 | 0.5474 |
| ENSP00000370557 | MIS12 | 0.5474 |
| ENSP00000413632 | MCMDC2 | 0.5474 |
| ENSP00000451605 | MC1R | 0.5474 |
| ENSP00000337354 | LIPA | 0.5474 |
| ENSP00000401437 | LDB3 | 0.5474 |
| ENSP00000265023 | KNG1 | 0.5474 |
| ENSP00000369442 | KL | 0.5474 |
| ENSP00000368699 | ISG15 | 0.5474 |
| ENSP00000378132 | IRX5 | 0.5474 |
| ENSP00000259206 | IL1RN | 0.5474 |
| ENSP00000364801 | HSPA1B | 0.5474 |
| ENSP00000325376 | HNRNPM | 0.5474 |
| ENSP00000365370 | HNRNPCL1 | 0.5474 |
| ENSP00000382034 | HLA-DQB1 | 0.5474 |
| ENSP00000384774 | HK1 | 0.5474 |
| ENSP00000272937 | HES6 | 0.5474 |
| ENSP00000379228 | HECW1 | 0.5474 |
| ENSP00000431245 | GUCY1A2 | 0.5474 |
| ENSP00000053469 | GUCA1A | 0.5474 |
| ENSP00000316598 | GTPBP6 | 0.5474 |
| ENSP00000381339 | GNAT3 | 0.5474 |
| ENSP00000347464 | GIT2 | 0.5474 |
| ENSP00000312673 | GH1 | 0.5474 |
| ENSP00000229416 | GCLC | 0.5474 |
| ENSP00000425845 | FYB | 0.5474 |
| ENSP00000341961 | FOXD4L3 | 0.5474 |
| ENSP00000216330 | FKBP3 | 0.5474 |

| | | |
|---|---|---|
| ENSP00000263621 | ELANE | 0.5474 |
| ENSP00000416250 | EIF2B1 | 0.5474 |
| ENSP00000417980 | EHMT1 | 0.5474 |
| ENSP00000301729 | ECI1 | 0.5474 |
| ENSP00000451995 | DUX4L5 | 0.5474 |
| ENSP00000362014 | DNM1 | 0.5474 |
| ENSP00000264065 | DNAJC10 | 0.5474 |
| ENSP00000199320 | DIMT1 | 0.5474 |
| ENSP00000424838 | DDX4 | 0.5474 |
| ENSP00000336725 | DDX3Y | 0.5474 |
| ENSP00000242776 | DDX39A | 0.5474 |
| ENSP00000421689 | CSNK1A1 | 0.5474 |
| ENSP00000321606 | CRMP1 | 0.5474 |
| ENSP00000356024 | CR2 | 0.5474 |
| ENSP00000264463 | CDH10 | 0.5474 |
| ENSP00000309181 | CCNE2 | 0.5474 |
| ENSP00000389009 | CARNS1 | 0.5474 |
| ENSP00000266376 | CACNA1C | 0.5474 |
| ENSP00000363773 | C1QA | 0.5474 |
| ENSP00000310697 | BRSK2 | 0.5474 |
| ENSP00000318822 | BID | 0.5474 |
| ENSP00000362993 | BICC1 | 0.5474 |
| ENSP00000273398 | ATP6V1A | 0.5474 |
| ENSP00000389649 | ATP5J | 0.5474 |
| ENSP00000350310 | ATP2B4 | 0.5474 |
| ENSP00000261173 | ATP2B1 | 0.5474 |
| ENSP00000353072 | ATP2A3 | 0.5474 |
| ENSP00000253004 | ASS1 | 0.5474 |
| ENSP00000377380 | ARHGAP9 | 0.5474 |
| ENSP00000295897 | ALB | 0.5474 |
| ENSP00000333019 | ADSSL1 | 0.5474 |
| ENSP00000297323 | ADCY1 | 0.5474 |
| ENSP00000216254 | ACO2 | 0.5474 |
| ENSP00000262710 | ACIN1 | 0.5474 |
| ENSP00000363868 | ABCA1 | 0.5474 |
| ENSP00000221167 | TUSC3 | 0.5474 |
| ENSP00000363055 | ZWINT | 0.5447 |
| ENSP00000265351 | XPO5 | 0.5447 |
| ENSP00000219473 | USP10 | 0.5447 |
| ENSP00000268379 | UQCRC2 | 0.5447 |
| ENSP00000429562 | UNC13A | 0.5447 |
| ENSP00000304697 | UBB | 0.5447 |
| ENSP00000263321 | TYR | 0.5447 |
| ENSP00000369081 | TXNDC5 | 0.5447 |
| ENSP00000361186 | TP53RK | 0.5447 |
| ENSP00000371475 | TP53BP1 | 0.5447 |
| ENSP00000311579 | TNKS | 0.5447 |
| ENSP00000399511 | TNIK | 0.5447 |
| ENSP00000405455 | TKT | 0.5447 |
| ENSP00000262768 | TIMP2 | 0.5447 |
| ENSP00000260129 | TGS1 | 0.5447 |
| ENSP00000345772 | TEAD3 | 0.5447 |
| ENSP00000390621 | TDRD12 | 0.5447 |

| | | |
|---|---|---|
| ENSP00000217964 | TBL1X | 0.5447 |
| ENSP00000329967 | TBK1 | 0.5447 |
| ENSP00000336724 | TBC1D22A | 0.5447 |
| ENSP00000350719 | SYNE2 | 0.5447 |
| ENSP00000348069 | SREBF1 | 0.5447 |
| ENSP00000230085 | SNX3 | 0.5447 |
| ENSP00000317123 | SNRNP200 | 0.5447 |
| ENSP00000249647 | SNAP23 | 0.5447 |
| ENSP00000355924 | SMYD2 | 0.5447 |
| ENSP00000341382 | SMC4 | 0.5447 |
| ENSP00000253270 | SLC35D2 | 0.5447 |
| ENSP00000360181 | SH2D1A | 0.5447 |
| ENSP00000221566 | SGTA | 0.5447 |
| ENSP00000266066 | SFRP5 | 0.5447 |
| ENSP00000360034 | SERBP1 | 0.5447 |
| ENSP00000251900 | SCML2 | 0.5447 |
| ENSP00000472465 | SARS2 | 0.5447 |
| ENSP00000379339 | RPS29 | 0.5447 |
| ENSP00000363676 | RPL11 | 0.5447 |
| ENSP00000298283 | RPL10L | 0.5447 |
| ENSP00000216274 | RIPK3 | 0.5447 |
| ENSP00000313890 | RBM15B | 0.5447 |
| ENSP00000316589 | RANBP10 | 0.5447 |
| ENSP00000372088 | RAD51 | 0.5447 |
| ENSP00000323867 | PRKAG1 | 0.5447 |
| ENSP00000343190 | PPCDC | 0.5447 |
| ENSP00000403852 | POLR2F | 0.5447 |
| ENSP00000265849 | PMS2 | 0.5447 |
| ENSP00000268261 | PMM2 | 0.5447 |
| ENSP00000323194 | PLXNA4 | 0.5447 |
| ENSP00000282903 | PLOD2 | 0.5447 |
| ENSP00000260766 | PLCE1 | 0.5447 |
| ENSP00000337797 | PIDD | 0.5447 |
| ENSP00000271657 | PI4KB | 0.5447 |
| ENSP00000332116 | PDE4B | 0.5447 |
| ENSP00000233630 | PCGF1 | 0.5447 |
| ENSP00000298281 | PCF11 | 0.5447 |
| ENSP00000322957 | PAK7 | 0.5447 |
| ENSP00000362618 | PABPC1L2A | 0.5447 |
| ENSP00000264151 | OSGEPL1 | 0.5447 |
| ENSP00000360621 | ORC1 | 0.5447 |
| ENSP00000357838 | OAT | 0.5447 |
| ENSP00000261396 | NUP133 | 0.5447 |
| ENSP00000301490 | NUDT8 | 0.5447 |
| ENSP00000274606 | NHP2 | 0.5447 |
| ENSP00000368856 | NFX1 | 0.5447 |
| ENSP00000357206 | NES | 0.5447 |
| ENSP00000347988 | NDUFA5 | 0.5447 |
| ENSP00000252102 | NDUFA2 | 0.5447 |
| ENSP00000294785 | NCSTN | 0.5447 |
| ENSP00000293373 | NCKAP1L | 0.5447 |
| ENSP00000265433 | NBN | 0.5447 |
| ENSP00000205890 | MYO15A | 0.5447 |

| | | |
|---|---|---|
| ENSP00000420714 | MRPS14 | 0.5447 |
| ENSP00000373404 | MRPL2 | 0.5447 |
| ENSP00000233616 | MOGS | 0.5447 |
| ENSP00000267273 | METTL21C | 0.5447 |
| ENSP00000323720 | MED14 | 0.5447 |
| ENSP00000262027 | MARS | 0.5447 |
| ENSP00000263925 | LNX1 | 0.5447 |
| ENSP00000294638 | LHX8 | 0.5447 |
| ENSP00000267436 | L2HGDH | 0.5447 |
| ENSP00000322791 | KIF1A | 0.5447 |
| ENSP00000369989 | KATNAL1 | 0.5447 |
| ENSP00000287239 | KAT6B | 0.5447 |
| ENSP00000355988 | IRF6 | 0.5447 |
| ENSP00000369312 | IL15RA | 0.5447 |
| ENSP00000370473 | IGFBP3 | 0.5447 |
| ENSP00000229135 | IFNG | 0.5447 |
| ENSP00000217901 | IDH3G | 0.5447 |
| ENSP00000219439 | HSDL1 | 0.5447 |
| ENSP00000309336 | HOXC5 | 0.5447 |
| ENSP00000338477 | HNRNPF | 0.5447 |
| ENSP00000314080 | HIC1 | 0.5447 |
| ENSP00000374353 | HAGHL | 0.5447 |
| ENSP00000377969 | GTF2F1 | 0.5447 |
| ENSP00000225567 | GOSR2 | 0.5447 |
| ENSP00000248572 | GNGT1 | 0.5447 |
| ENSP00000248996 | GNAZ | 0.5447 |
| ENSP00000232461 | GNAT1 | 0.5447 |
| ENSP00000277865 | GLUD1 | 0.5447 |
| ENSP00000252506 | GADD45G | 0.5447 |
| ENSP00000180166 | FGF20 | 0.5447 |
| ENSP00000424243 | EYS | 0.5447 |
| ENSP00000312385 | EVX2 | 0.5447 |
| ENSP00000376684 | EPHB6 | 0.5447 |
| ENSP00000301825 | ENTPD3 | 0.5447 |
| ENSP00000438513 | DUX4L4 | 0.5447 |
| ENSP00000365272 | DLG2 | 0.5447 |
| ENSP00000210060 | DHPS | 0.5447 |
| ENSP00000319170 | DHFRL1 | 0.5447 |
| ENSP00000360828 | DDX27 | 0.5447 |
| ENSP00000304994 | DACH1 | 0.5447 |
| ENSP00000355013 | CTR9 | 0.5447 |
| ENSP00000307134 | CTNND2 | 0.5447 |
| ENSP00000319343 | CPT1C | 0.5447 |
| ENSP00000292476 | CPSF4 | 0.5447 |
| ENSP00000300107 | CLPX | 0.5447 |
| ENSP00000262662 | CDKN2C | 0.5447 |
| ENSP00000217372 | CDK5RAP1 | 0.5447 |
| ENSP00000354826 | CALD1 | 0.5447 |
| ENSP00000360664 | BTF3L4 | 0.5447 |
| ENSP00000375082 | BORA | 0.5447 |
| ENSP00000346437 | ATG7 | 0.5447 |
| ENSP00000339016 | ATAD1 | 0.5447 |
| ENSP00000380427 | ARPC4-TTLL3 | 0.5447 |

| | | |
|---|---|---|
| ENSP00000411459 | ARHGEF28 | 0.5447 |
| ENSP00000216714 | APEX1 | 0.5447 |
| ENSP00000296511 | ANXA5 | 0.5447 |
| ENSP00000256578 | AMPD2 | 0.5447 |
| ENSP00000384881 | ABCB5 | 0.5447 |
| ENSP00000228251 | YBX3 | 0.5421 |
| ENSP00000349658 | XPNPEP3 | 0.5421 |
| ENSP00000342381 | VRK2 | 0.5421 |
| ENSP00000361635 | VDAC2 | 0.5421 |
| ENSP00000339957 | USP47 | 0.5421 |
| ENSP00000309565 | UQCRH | 0.5421 |
| ENSP00000296328 | UBXN7 | 0.5421 |
| ENSP00000356243 | UBE2T | 0.5421 |
| ENSP00000315644 | TYMS | 0.5421 |
| ENSP00000370034 | TLR7 | 0.5421 |
| ENSP00000276692 | TATDN1 | 0.5421 |
| ENSP00000409667 | SYNJ1 | 0.5421 |
| ENSP00000258301 | STX6 | 0.5421 |
| ENSP00000365730 | STK24 | 0.5421 |
| ENSP00000278412 | SSRP1 | 0.5421 |
| ENSP00000420195 | SRSF10 | 0.5421 |
| ENSP00000266735 | SNRPF | 0.5421 |
| ENSP00000255175 | SERINC3 | 0.5421 |
| ENSP00000345445 | SAMM50 | 0.5421 |
| ENSP00000359035 | RTEL1 | 0.5421 |
| ENSP00000272190 | REN | 0.5421 |
| ENSP00000321239 | RCHY1 | 0.5421 |
| ENSP00000384192 | RASGRP3 | 0.5421 |
| ENSP00000386520 | RAD54L2 | 0.5421 |
| ENSP00000264025 | PVRL1 | 0.5421 |
| ENSP00000292644 | PSMC2 | 0.5421 |
| ENSP00000363993 | PSMB9 | 0.5421 |
| ENSP00000216455 | PSMA3 | 0.5421 |
| ENSP00000325074 | PPP2R2A | 0.5421 |
| ENSP00000230732 | POU4F3 | 0.5421 |
| ENSP00000342889 | POLR2K | 0.5421 |
| ENSP00000219252 | POLR2C | 0.5421 |
| ENSP00000405950 | PNCK | 0.5421 |
| ENSP00000361275 | PLK3 | 0.5421 |
| ENSP00000338185 | PLCB1 | 0.5421 |
| ENSP00000471914 | PIK3R2 | 0.5421 |
| ENSP00000360473 | PFDN4 | 0.5421 |
| ENSP00000313851 | PDS5B | 0.5421 |
| ENSP00000339906 | PAX4 | 0.5421 |
| ENSP00000353864 | PAK3 | 0.5421 |
| ENSP00000244458 | PACSIN1 | 0.5421 |
| ENSP00000343819 | OTX2 | 0.5421 |
| ENSP00000339479 | NT5C2 | 0.5421 |
| ENSP00000433415 | NT5C1B-RDH14 | 0.5421 |
| ENSP00000220931 | NCALD | 0.5421 |
| ENSP00000348349 | MYO9A | 0.5421 |
| ENSP00000386331 | MYO7A | 0.5421 |
| ENSP00000358994 | MYO6 | 0.5421 |

| | | |
|---|---|---|
| ENSP00000207437 | MYL6B | 0.5421 |
| ENSP00000446714 | MYL6 | 0.5421 |
| ENSP00000435591 | MUC5AC | 0.5421 |
| ENSP00000376800 | MTPN | 0.5421 |
| ENSP00000333633 | MTA1 | 0.5421 |
| ENSP00000296473 | MON1A | 0.5421 |
| ENSP00000257745 | MLL5 | 0.5421 |
| ENSP00000281453 | MLF1IP | 0.5421 |
| ENSP00000364946 | MKX | 0.5421 |
| ENSP00000351664 | MICAL1 | 0.5421 |
| ENSP00000228938 | MGP | 0.5421 |
| ENSP00000447378 | MGAM | 0.5421 |
| ENSP00000230321 | MDFI | 0.5421 |
| ENSP00000314505 | MCM9 | 0.5421 |
| ENSP00000367309 | MAOB | 0.5421 |
| ENSP00000400365 | LAMA2 | 0.5421 |
| ENSP00000290759 | ISL2 | 0.5421 |
| ENSP00000290552 | IRX6 | 0.5421 |
| ENSP00000303231 | IL12A | 0.5421 |
| ENSP00000299518 | IDH3A | 0.5421 |
| ENSP00000233893 | HSPE1 | 0.5421 |
| ENSP00000357440 | HSF2 | 0.5421 |
| ENSP00000259667 | HINT2 | 0.5421 |
| ENSP00000393198 | HES4 | 0.5421 |
| ENSP00000261195 | GYS2 | 0.5421 |
| ENSP00000452454 | GTF2A1 | 0.5421 |
| ENSP00000304331 | GSX1 | 0.5421 |
| ENSP00000359864 | GPC4 | 0.5421 |
| ENSP00000341848 | GOLGB1 | 0.5421 |
| ENSP00000232564 | GNB4 | 0.5421 |
| ENSP00000370737 | GLDC | 0.5421 |
| ENSP00000368226 | GK | 0.5421 |
| ENSP00000410833 | GBE1 | 0.5421 |
| ENSP00000299162 | FOXN4 | 0.5421 |
| ENSP00000370373 | FKBP1B | 0.5421 |
| ENSP00000237837 | FGF23 | 0.5421 |
| ENSP00000276326 | FBXO25 | 0.5421 |
| ENSP00000431822 | FAU | 0.5421 |
| ENSP00000359939 | EXOSC1 | 0.5421 |
| ENSP00000370330 | ERBB2IP | 0.5421 |
| ENSP00000268933 | EPN3 | 0.5421 |
| ENSP00000430269 | EPHX2 | 0.5421 |
| ENSP00000338481 | EPB41L2 | 0.5421 |
| ENSP00000234590 | ENO1 | 0.5421 |
| ENSP00000348842 | EML6 | 0.5421 |
| ENSP00000289371 | EIF5B | 0.5421 |
| ENSP00000425561 | EIF4E | 0.5421 |
| ENSP00000429931 | EIF3H | 0.5421 |
| ENSP00000332604 | EIF3C | 0.5421 |
| ENSP00000312789 | DYRK1B | 0.5421 |
| ENSP00000451304 | DUX4 | 0.5421 |
| ENSP00000225171 | DNAJC12 | 0.5421 |
| ENSP00000256996 | DDB2 | 0.5421 |

| | | |
|---|---|---|
| ENSP00000264161 | DARS | 0.5421 |
| ENSP00000386884 | CXCR4 | 0.5421 |
| ENSP00000353904 | CSNK1G3 | 0.5421 |
| ENSP00000298875 | CPSF2 | 0.5421 |
| ENSP00000006101 | COPZ2 | 0.5421 |
| ENSP00000299259 | COPS2 | 0.5421 |
| ENSP00000357048 | COPA | 0.5421 |
| ENSP00000340937 | COL17A1 | 0.5421 |
| ENSP00000464814 | COASY | 0.5421 |
| ENSP00000265689 | CHKA | 0.5421 |
| ENSP00000262608 | CECR2 | 0.5421 |
| ENSP00000286788 | CCT8 | 0.5421 |
| ENSP00000256897 | CCNH | 0.5421 |
| ENSP00000247461 | CANX | 0.5421 |
| ENSP00000343741 | ATR | 0.5421 |
| ENSP00000340896 | ASH2L | 0.5421 |
| ENSP00000365411 | APBB1IP | 0.5421 |
| ENSP00000263640 | ACVR1 | 0.5421 |
| ENSP00000344789 | ACACA | 0.5421 |
| ENSP00000379847 | ZNF143 | 0.5395 |
| ENSP00000426964 | YJEFN3 | 0.5395 |
| ENSP00000362677 | WDR38 | 0.5395 |
| ENSP00000309457 | VPS41 | 0.5395 |
| ENSP00000353422 | VPS13A | 0.5395 |
| ENSP00000287022 | UQCRB | 0.5395 |
| ENSP00000332887 | UQCR10 | 0.5395 |
| ENSP00000348708 | UPF2 | 0.5395 |
| ENSP00000242576 | UNG | 0.5395 |
| ENSP00000346155 | UCKL1 | 0.5395 |
| ENSP00000363019 | UBE2D1 | 0.5395 |
| ENSP00000320295 | TUBB3 | 0.5395 |
| ENSP00000301072 | TUBA1C | 0.5395 |
| ENSP00000267890 | TTBK2 | 0.5395 |
| ENSP00000341838 | TNNI3 | 0.5395 |
| ENSP00000265131 | TNC | 0.5395 |
| ENSP00000327315 | TIAM2 | 0.5395 |
| ENSP00000383892 | TFIP11 | 0.5395 |
| ENSP00000240328 | TBX2 | 0.5395 |
| ENSP00000358060 | TARS2 | 0.5395 |
| ENSP00000330219 | SYN3 | 0.5395 |
| ENSP00000295709 | STK36 | 0.5395 |
| ENSP00000360992 | STAMBPL1 | 0.5395 |
| ENSP00000263736 | SRBD1 | 0.5395 |
| ENSP00000325785 | SPECC1L | 0.5395 |
| ENSP00000381034 | SOCS6 | 0.5395 |
| ENSP00000342374 | SNRPD2 | 0.5395 |
| ENSP00000215730 | SNAP29 | 0.5395 |
| ENSP00000402515 | SMG1 | 0.5395 |
| ENSP00000249396 | SIRT2 | 0.5395 |
| ENSP00000370839 | SGCB | 0.5395 |
| ENSP00000362110 | SF3A3 | 0.5395 |
| ENSP00000303540 | SCN1A | 0.5395 |
| ENSP00000254663 | SCLY | 0.5395 |

| | | |
|---|---|---|
| ENSP00000386444 | SAG | 0.5395 |
| ENSP00000260443 | RSL24D1 | 0.5395 |
| ENSP00000258955 | RSAD1 | 0.5395 |
| ENSP00000377865 | RPL23 | 0.5395 |
| ENSP00000363018 | RPL10A | 0.5395 |
| ENSP00000301730 | RNPS1 | 0.5395 |
| ENSP00000338864 | RASGRP2 | 0.5395 |
| ENSP00000335029 | RASA3 | 0.5395 |
| ENSP00000290101 | RAP1GAP | 0.5395 |
| ENSP00000220966 | PYCRL | 0.5395 |
| ENSP00000396937 | PSMD13 | 0.5395 |
| ENSP00000222266 | PSENEN | 0.5395 |
| ENSP00000326366 | PSEN1 | 0.5395 |
| ENSP00000363313 | PRPF4 | 0.5395 |
| ENSP00000235372 | PRDM2 | 0.5395 |
| ENSP00000324124 | POLR2L | 0.5395 |
| ENSP00000473036 | PNMAL2 | 0.5395 |
| ENSP00000376765 | PIAS3 | 0.5395 |
| ENSP00000363003 | PHF19 | 0.5395 |
| ENSP00000263666 | PDZRN3 | 0.5395 |
| ENSP00000291539 | PDE9A | 0.5395 |
| ENSP00000270474 | PDE4A | 0.5395 |
| ENSP00000331487 | NPLOC4 | 0.5395 |
| ENSP00000219302 | NME3 | 0.5395 |
| ENSP00000264187 | NID1 | 0.5395 |
| ENSP00000263774 | NDUFS3 | 0.5395 |
| ENSP00000286794 | NAA11 | 0.5395 |
| ENSP00000324527 | MYO1D | 0.5395 |
| ENSP00000292327 | MYL3 | 0.5395 |
| ENSP00000359321 | MTF2 | 0.5395 |
| ENSP00000244051 | MOCS3 | 0.5395 |
| ENSP00000380157 | MOCS2 | 0.5395 |
| ENSP00000162023 | MEF2BNB-MEF2B | 0.5395 |
| ENSP00000314776 | MBLAC2 | 0.5395 |
| ENSP00000354573 | MACF1 | 0.5395 |
| ENSP00000431284 | LPXN | 0.5395 |
| ENSP00000264144 | LAMC2 | 0.5395 |
| ENSP00000222399 | LAMB1 | 0.5395 |
| ENSP00000343701 | KPNA1 | 0.5395 |
| ENSP00000334140 | KLHDC10 | 0.5395 |
| ENSP00000348108 | KHDRBS3 | 0.5395 |
| ENSP00000311202 | KCTD13 | 0.5395 |
| ENSP00000386896 | ITGA6 | 0.5395 |
| ENSP00000231357 | IRX4 | 0.5395 |
| ENSP00000226284 | IBSP | 0.5395 |
| ENSP00000262854 | HUWE1 | 0.5395 |
| ENSP00000357980 | HTRA1 | 0.5395 |
| ENSP00000264009 | HSF4 | 0.5395 |
| ENSP00000375863 | HNRNPUL1 | 0.5395 |
| ENSP00000316042 | HNRNPA0 | 0.5395 |
| ENSP00000379701 | HNF4G | 0.5395 |
| ENSP00000346643 | HKDC1 | 0.5395 |
| ENSP00000290573 | HK2 | 0.5395 |

| | | |
|---|---|---|
| ENSP00000368066 | HAO1 | 0.5395 |
| ENSP00000319799 | H1FOO | 0.5395 |
| ENSP00000379839 | GNE | 0.5395 |
| ENSP00000328570 | GLRX5 | 0.5395 |
| ENSP00000358866 | FLNA | 0.5395 |
| ENSP00000342087 | FHIT | 0.5395 |
| ENSP00000359635 | FGF13 | 0.5395 |
| ENSP00000293829 | FGF11 | 0.5395 |
| ENSP00000252771 | FCHO1 | 0.5395 |
| ENSP00000289779 | F11R | 0.5395 |
| ENSP00000254928 | ERAL1 | 0.5395 |
| ENSP00000374323 | EPHA6 | 0.5395 |
| ENSP00000275815 | EPHA1 | 0.5395 |
| ENSP00000253039 | EIF2S3 | 0.5395 |
| ENSP00000250457 | EGLN3 | 0.5395 |
| ENSP00000358596 | DUSP5 | 0.5395 |
| ENSP00000270223 | DMWD | 0.5395 |
| ENSP00000369213 | DDX58 | 0.5395 |
| ENSP00000303356 | DCXR | 0.5395 |
| ENSP00000408910 | DCTN2 | 0.5395 |
| ENSP00000349576 | DCTD | 0.5395 |
| ENSP00000342070 | CTSB | 0.5395 |
| ENSP00000394227 | CSF2RA | 0.5395 |
| ENSP00000317780 | COX5A | 0.5395 |
| ENSP00000261070 | COX17 | 0.5395 |
| ENSP00000288532 | COQ5 | 0.5395 |
| ENSP00000329419 | COPB2 | 0.5395 |
| ENSP00000379866 | COL4A4 | 0.5395 |
| ENSP00000295522 | CLDN1 | 0.5395 |
| ENSP00000220913 | CHRAC1 | 0.5395 |
| ENSP00000350386 | CHM | 0.5395 |
| ENSP00000342510 | CEP97 | 0.5395 |
| ENSP00000274170 | CDH18 | 0.5395 |
| ENSP00000276014 | CCNB3 | 0.5395 |
| ENSP00000405533 | CCL4L1 | 0.5395 |
| ENSP00000336687 | CBX3 | 0.5395 |
| ENSP00000420381 | CALU | 0.5395 |
| ENSP00000307508 | CALB2 | 0.5395 |
| ENSP00000259008 | BRIP1 | 0.5395 |
| ENSP00000370003 | BNIP3L | 0.5395 |
| ENSP00000267859 | BNIP2 | 0.5395 |
| ENSP00000447537 | BLOC1S1 | 0.5395 |
| ENSP00000392330 | BCAP31 | 0.5395 |
| ENSP00000301587 | ATP5H | 0.5395 |
| ENSP00000243997 | ATP5E | 0.5395 |
| ENSP00000339182 | ATP13A4 | 0.5395 |
| ENSP00000289968 | ARHGAP17 | 0.5395 |
| ENSP00000314615 | ARFGAP1 | 0.5395 |
| ENSP00000239440 | ARAP3 | 0.5395 |
| ENSP00000364126 | APEX2 | 0.5395 |
| ENSP00000346032 | ANXA2 | 0.5395 |
| ENSP00000332369 | ALAS2 | 0.5395 |
| ENSP00000352584 | AKR1B10 | 0.5395 |

| | | |
|---|---|---|
| ENSP00000362300 | AGO1 | 0.5395 |
| ENSP00000305372 | ADRB2 | 0.5395 |
| ENSP00000358301 | ADRB1 | 0.5395 |
| ENSP00000442656 | ACVR1B | 0.5395 |
| ENSP00000224784 | ACTA2 | 0.5395 |
| ENSP00000303211 | ACHE | 0.5395 |
| ENSP00000356015 | ACAT2 | 0.5395 |
| ENSP00000357873 | ACADSB | 0.5395 |
| ENSP00000244571 | AARS2 | 0.5395 |
| ENSP00000450811 | ENSG00000259131 | 0.5395 |
| ENSP00000302790 | XPO6 | 0.5368 |
| ENSP00000427772 | WWC1 | 0.5368 |
| ENSP00000293879 | WDR90 | 0.5368 |
| ENSP00000330381 | WDR5B | 0.5368 |
| ENSP00000263559 | VPS26A | 0.5368 |
| ENSP00000307863 | U2AF2 | 0.5368 |
| ENSP00000416959 | TRA2B | 0.5368 |
| ENSP00000263525 | TNR | 0.5368 |
| ENSP00000226225 | TNFAIP1 | 0.5368 |
| ENSP00000406293 | TCEA3 | 0.5368 |
| ENSP00000299328 | TAZ | 0.5368 |
| ENSP00000240185 | TARDBP | 0.5368 |
| ENSP00000294179 | STX5 | 0.5368 |
| ENSP00000336655 | STRADA | 0.5368 |
| ENSP00000361892 | STK4 | 0.5368 |
| ENSP00000472998 | SNRNP70 | 0.5368 |
| ENSP00000254976 | SNAP25 | 0.5368 |
| ENSP00000360163 | SMARCA1 | 0.5368 |
| ENSP00000288840 | SMAD6 | 0.5368 |
| ENSP00000316152 | SFTPC | 0.5368 |
| ENSP00000305790 | SF3B3 | 0.5368 |
| ENSP00000356953 | SDHC | 0.5368 |
| ENSP00000264932 | SDHA | 0.5368 |
| ENSP00000371973 | SAP18 | 0.5368 |
| ENSP00000311430 | RPL4 | 0.5368 |
| ENSP00000309334 | RPL15 | 0.5368 |
| ENSP00000253303 | RGN | 0.5368 |
| ENSP00000294904 | RBMS1 | 0.5368 |
| ENSP00000230419 | PTK7 | 0.5368 |
| ENSP00000348442 | PSMD12 | 0.5368 |
| ENSP00000259457 | PSMB7 | 0.5368 |
| ENSP00000270586 | PSMB6 | 0.5368 |
| ENSP00000044462 | PSMA4 | 0.5368 |
| ENSP00000392262 | PPP5D1 | 0.5368 |
| ENSP00000306682 | PPM1D | 0.5368 |
| ENSP00000306614 | PPIH | 0.5368 |
| ENSP00000308938 | PLG | 0.5368 |
| ENSP00000430344 | PLCD1 | 0.5368 |
| ENSP00000361850 | PLAU | 0.5368 |
| ENSP00000333024 | PHF7 | 0.5368 |
| ENSP00000216252 | PHF5A | 0.5368 |
| ENSP00000370517 | PFKP | 0.5368 |
| ENSP00000269848 | PFKL | 0.5368 |

| | | |
|---|---|---|
| ENSP00000307259 | P2RY12 | 0.5368 |
| ENSP00000234296 | ORC2 | 0.5368 |
| ENSP00000231498 | NUP155 | 0.5368 |
| ENSP00000282516 | NIPBL | 0.5368 |
| ENSP00000380505 | NEB | 0.5368 |
| ENSP00000255120 | NASP | 0.5368 |
| ENSP00000303584 | N6AMT1 | 0.5368 |
| ENSP00000306382 | MYO1B | 0.5368 |
| ENSP00000301012 | MVD | 0.5368 |
| ENSP00000257068 | MTNR1B | 0.5368 |
| ENSP00000356290 | MTHFD1L | 0.5368 |
| ENSP00000354982 | MT-CO3 | 0.5368 |
| ENSP00000377486 | MRPL19 | 0.5368 |
| ENSP00000262966 | MPND | 0.5368 |
| ENSP00000355775 | MIXL1 | 0.5368 |
| ENSP00000321684 | MEOX1 | 0.5368 |
| ENSP00000307288 | MCM7 | 0.5368 |
| ENSP00000282276 | MARS2 | 0.5368 |
| ENSP00000261845 | MAPK6 | 0.5368 |
| ENSP00000266718 | LUM | 0.5368 |
| ENSP00000367498 | LRRC47 | 0.5368 |
| ENSP00000285737 | LONP2 | 0.5368 |
| ENSP00000259708 | KLC4 | 0.5368 |
| ENSP00000247986 | KIF17 | 0.5368 |
| ENSP00000323659 | KDM3A | 0.5368 |
| ENSP00000406037 | KAT8 | 0.5368 |
| ENSP00000323580 | IFT88 | 0.5368 |
| ENSP00000384144 | HYOU1 | 0.5368 |
| ENSP00000258080 | HTRA2 | 0.5368 |
| ENSP00000303394 | HSPB3 | 0.5368 |
| ENSP00000254963 | HSPA12B | 0.5368 |
| ENSP00000381785 | HSDL2 | 0.5368 |
| ENSP00000278353 | HSD17B12 | 0.5368 |
| ENSP00000331741 | HOXB2 | 0.5368 |
| ENSP00000222718 | HOXA2 | 0.5368 |
| ENSP00000281543 | GUF1 | 0.5368 |
| ENSP00000301149 | GPD1 | 0.5368 |
| ENSP00000330836 | GLRX3 | 0.5368 |
| ENSP00000282841 | GGPS1 | 0.5368 |
| ENSP00000252809 | GDF15 | 0.5368 |
| ENSP00000343633 | FOXN2 | 0.5368 |
| ENSP00000354620 | FOXJ3 | 0.5368 |
| ENSP00000252037 | FKBP6 | 0.5368 |
| ENSP00000353741 | ETF1 | 0.5368 |
| ENSP00000278927 | ESAM | 0.5368 |
| ENSP00000354238 | ENPP1 | 0.5368 |
| ENSP00000363559 | EIF6 | 0.5368 |
| ENSP00000216554 | EIF5 | 0.5368 |
| ENSP00000262056 | EIF4B | 0.5368 |
| ENSP00000253108 | EIF3G | 0.5368 |
| ENSP00000353575 | EIF2B3 | 0.5368 |
| ENSP00000266126 | EIF2B2 | 0.5368 |
| ENSP00000368683 | EDN1 | 0.5368 |

| | | |
|---|---|---|
| ENSP00000362850 | DSN1 | 0.5368 |
| ENSP00000359290 | DR1 | 0.5368 |
| ENSP00000396308 | DHFR | 0.5368 |
| ENSP00000337697 | DCX | 0.5368 |
| ENSP00000259632 | DCTN3 | 0.5368 |
| ENSP00000384573 | DAZ1 | 0.5368 |
| ENSP00000221996 | CRX | 0.5368 |
| ENSP00000238112 | CPSF3 | 0.5368 |
| ENSP00000355180 | COL6A1 | 0.5368 |
| ENSP00000351682 | CNDP1 | 0.5368 |
| ENSP00000405575 | CHMP3 | 0.5368 |
| ENSP00000317902 | CEP57 | 0.5368 |
| ENSP00000360540 | CEP55 | 0.5368 |
| ENSP00000299564 | CDYL2 | 0.5368 |
| ENSP00000274695 | CDKAL1 | 0.5368 |
| ENSP00000304370 | CDC40 | 0.5368 |
| ENSP00000354916 | CDC14A | 0.5368 |
| ENSP00000009180 | CD9 | 0.5368 |
| ENSP00000315477 | CD209 | 0.5368 |
| ENSP00000292314 | CCDC12 | 0.5368 |
| ENSP00000279247 | CAPN1 | 0.5368 |
| ENSP00000355192 | CACNA1S | 0.5368 |
| ENSP00000350267 | BRPF3 | 0.5368 |
| ENSP00000220659 | BRF2 | 0.5368 |
| ENSP00000320509 | BANK1 | 0.5368 |
| ENSP00000307875 | B3GAT1 | 0.5368 |
| ENSP00000301178 | AXL | 0.5368 |
| ENSP00000378917 | ATXN2L | 0.5368 |
| ENSP00000349595 | ATP2A1 | 0.5368 |
| ENSP00000357060 | ATP1A4 | 0.5368 |
| ENSP00000349877 | ATP13A1 | 0.5368 |
| ENSP00000425107 | ATG12 | 0.5368 |
| ENSP00000263207 | ARVCF | 0.5368 |
| ENSP00000261842 | AP4E1 | 0.5368 |
| ENSP00000312442 | AP1G2 | 0.5368 |
| ENSP00000280772 | ANK3 | 0.5368 |
| ENSP00000358814 | AHCYL1 | 0.5368 |
| ENSP00000254235 | ADCY7 | 0.5368 |
| ENSP00000237612 | ABCG2 | 0.5368 |
| ENSP00000384863 | XPO1 | 0.5342 |
| ENSP00000406084 | WDR20 | 0.5342 |
| ENSP00000365891 | WAS | 0.5342 |
| ENSP00000265333 | VDAC1 | 0.5342 |
| ENSP00000265077 | VCAN | 0.5342 |
| ENSP00000364815 | VARS | 0.5342 |
| ENSP00000258399 | USP37 | 0.5342 |
| ENSP00000364448 | UPF3A | 0.5342 |
| ENSP00000217133 | TUBB1 | 0.5342 |
| ENSP00000248437 | TUBA4A | 0.5342 |
| ENSP00000298746 | TRUB1 | 0.5342 |
| ENSP00000283943 | TRIP12 | 0.5342 |
| ENSP00000295756 | TRAT1 | 0.5342 |
| ENSP00000302783 | TRAPPC1 | 0.5342 |

| | | |
|---|---|---|
| ENSP00000318820 | TPO | 0.5342 |
| ENSP00000328835 | TOP1MT | 0.5342 |
| ENSP00000371341 | TNK2 | 0.5342 |
| ENSP00000263932 | TNFRSF8 | 0.5342 |
| ENSP00000465500 | TNFRSF11A | 0.5342 |
| ENSP00000303145 | TMED10 | 0.5342 |
| ENSP00000386341 | TICAM2 | 0.5342 |
| ENSP00000260356 | THBS1 | 0.5342 |
| ENSP00000362979 | TGIF2 | 0.5342 |
| ENSP00000367406 | TAF2 | 0.5342 |
| ENSP00000367208 | SUGT1 | 0.5342 |
| ENSP00000367923 | SUCLA2 | 0.5342 |
| ENSP00000360310 | SPO11 | 0.5342 |
| ENSP00000245222 | SPHK2 | 0.5342 |
| ENSP00000445829 | SLC39A11 | 0.5342 |
| ENSP00000333667 | SHMT2 | 0.5342 |
| ENSP00000345193 | SHANK2 | 0.5342 |
| ENSP00000373354 | SETMAR | 0.5342 |
| ENSP00000399518 | SATB1 | 0.5342 |
| ENSP00000346294 | S100A4 | 0.5342 |
| ENSP00000296084 | RYK | 0.5342 |
| ENSP00000457868 | RTEL1-TNFRSF6B | 0.5342 |
| ENSP00000217260 | RSPO4 | 0.5342 |
| ENSP00000300738 | RRM1 | 0.5342 |
| ENSP00000373331 | RPUSD3 | 0.5342 |
| ENSP00000470004 | RPS19 | 0.5342 |
| ENSP00000237530 | RPN2 | 0.5342 |
| ENSP00000386717 | RPL31 | 0.5342 |
| ENSP00000417335 | ROBO2 | 0.5342 |
| ENSP00000221486 | RNASEH2A | 0.5342 |
| ENSP00000365926 | RFK | 0.5342 |
| ENSP00000254901 | REEP2 | 0.5342 |
| ENSP00000371169 | RCL1 | 0.5342 |
| ENSP00000391723 | PUM1 | 0.5342 |
| ENSP00000352833 | PTPN22 | 0.5342 |
| ENSP00000356438 | PTGS2 | 0.5342 |
| ENSP00000334499 | PTBP3 | 0.5342 |
| ENSP00000365175 | PRRC2A | 0.5342 |
| ENSP00000366488 | PRKACG | 0.5342 |
| ENSP00000362803 | PPIL1 | 0.5342 |
| ENSP00000264220 | PPAT | 0.5342 |
| ENSP00000263331 | POLR1B | 0.5342 |
| ENSP00000442563 | POLG2 | 0.5342 |
| ENSP00000271715 | POGZ | 0.5342 |
| ENSP00000356000 | PLXNA2 | 0.5342 |
| ENSP00000196061 | PLOD1 | 0.5342 |
| ENSP00000359019 | PITX3 | 0.5342 |
| ENSP00000360124 | PGM1 | 0.5342 |
| ENSP00000322170 | PFN4 | 0.5342 |
| ENSP00000286091 | PDIA4 | 0.5342 |
| ENSP00000360502 | PDE6C | 0.5342 |
| ENSP00000347046 | PDE5A | 0.5342 |
| ENSP00000216277 | PAPOLA | 0.5342 |

| | | |
|---|---|---|
| ENSP00000230859 | PAPD7 | 0.5342 |
| ENSP00000308012 | PABPC5 | 0.5342 |
| ENSP00000462986 | PABPC4L | 0.5342 |
| ENSP00000304858 | ORMDL3 | 0.5342 |
| ENSP00000264279 | NOP58 | 0.5342 |
| ENSP00000199447 | NME8 | 0.5342 |
| ENSP00000363205 | NFS1 | 0.5342 |
| ENSP00000306754 | NEUROD2 | 0.5342 |
| ENSP00000261908 | NEO1 | 0.5342 |
| ENSP00000391942 | NECAP2 | 0.5342 |
| ENSP00000369176 | NDUFB6 | 0.5342 |
| ENSP00000265944 | MYO3A | 0.5342 |
| ENSP00000264668 | MTRR | 0.5342 |
| ENSP00000450560 | MTHFD1 | 0.5342 |
| ENSP00000263187 | MSH4 | 0.5342 |
| ENSP00000253099 | MRPL4 | 0.5342 |
| ENSP00000260227 | MMP7 | 0.5342 |
| ENSP00000223215 | MEST | 0.5342 |
| ENSP00000344223 | MBTPS1 | 0.5342 |
| ENSP00000303147 | MAT2A | 0.5342 |
| ENSP00000337691 | MAPK15 | 0.5342 |
| ENSP00000345629 | MAP3K15 | 0.5342 |
| ENSP00000253055 | MAP3K10 | 0.5342 |
| ENSP00000432799 | MANF | 0.5342 |
| ENSP00000413964 | LSM14A | 0.5342 |
| ENSP00000373600 | LRRK1 | 0.5342 |
| ENSP00000422533 | LPHN3 | 0.5342 |
| ENSP00000342071 | LIPT1 | 0.5342 |
| ENSP00000280704 | LDHC | 0.5342 |
| ENSP00000229319 | LDHB | 0.5342 |
| ENSP00000432695 | KIAA1456 | 0.5342 |
| ENSP00000228495 | KCTD10 | 0.5342 |
| ENSP00000259021 | KAT7 | 0.5342 |
| ENSP00000340330 | KAT5 | 0.5342 |
| ENSP00000347244 | ITSN2 | 0.5342 |
| ENSP00000367316 | ITGA8 | 0.5342 |
| ENSP00000428220 | IRGM | 0.5342 |
| ENSP00000258886 | IREB2 | 0.5342 |
| ENSP00000330825 | IFITM1 | 0.5342 |
| ENSP00000370223 | IDH3B | 0.5342 |
| ENSP00000358211 | HSPA12A | 0.5342 |
| ENSP00000352706 | HIBCH | 0.5342 |
| ENSP00000356811 | HBS1L | 0.5342 |
| ENSP00000222115 | HAS1 | 0.5342 |
| ENSP00000292377 | GPC2 | 0.5342 |
| ENSP00000248114 | GFER | 0.5342 |
| ENSP00000302251 | GBX2 | 0.5342 |
| ENSP00000314806 | FOXR1 | 0.5342 |
| ENSP00000215530 | FGF22 | 0.5342 |
| ENSP00000377862 | FAT4 | 0.5342 |
| ENSP00000289081 | FANCC | 0.5342 |
| ENSP00000323046 | EXOSC3 | 0.5342 |
| ENSP00000445077 | EIF2S3L | 0.5342 |

| | | |
|---|---|---|
| ENSP00000204961 | EFNB1 | 0.5342 |
| ENSP00000343690 | DPYSL3 | 0.5342 |
| ENSP00000309690 | DMXL1 | 0.5342 |
| ENSP00000405620 | DHX30 | 0.5342 |
| ENSP00000307305 | DDIT4 | 0.5342 |
| ENSP00000303887 | DCLK2 | 0.5342 |
| ENSP00000371802 | DAZ3 | 0.5342 |
| ENSP00000261623 | CYBA | 0.5342 |
| ENSP00000295683 | CXCR1 | 0.5342 |
| ENSP00000352222 | CTPS2 | 0.5342 |
| ENSP00000305777 | CSNK1G1 | 0.5342 |
| ENSP00000445508 | CNOT4 | 0.5342 |
| ENSP00000394734 | CLK1 | 0.5342 |
| ENSP00000269141 | CDH2 | 0.5342 |
| ENSP00000275603 | CCT6A | 0.5342 |
| ENSP00000344635 | CCNG1 | 0.5342 |
| ENSP00000250151 | CCL4 | 0.5342 |
| ENSP00000302234 | CCL11 | 0.5342 |
| ENSP00000309052 | CATSPER1 | 0.5342 |
| ENSP00000458075 | BOLA2B | 0.5342 |
| ENSP00000264568 | BMPR1B | 0.5342 |
| ENSP00000317469 | BBS1 | 0.5342 |
| ENSP00000262429 | ATP2C2 | 0.5342 |
| ENSP00000310561 | ARHGAP32 | 0.5342 |
| ENSP00000297991 | AQP3 | 0.5342 |
| ENSP00000366927 | ALDH1B1 | 0.5342 |
| ENSP00000362249 | AK1 | 0.5342 |
| ENSP00000252071 | ACTR3C | 0.5342 |
| ENSP00000301956 | ACSM1 | 0.5342 |
| ENSP00000300441 | ACSF2 | 0.5342 |
| ENSP00000409612 | ACADM | 0.5342 |
| ENSP00000389813 | ACAD10 | 0.5342 |
| ENSP00000472299 | AC013449.1 | 0.5342 |
| ENSP00000472933 | AC012493.2 | 0.5342 |
| ENSP00000323670 | ZBTB7A | 0.5316 |
| ENSP00000348283 | WWP2 | 0.5316 |
| ENSP00000286574 | WIF1 | 0.5316 |
| ENSP00000233055 | WDFY1 | 0.5316 |
| ENSP00000401191 | WBSCR22 | 0.5316 |
| ENSP00000235521 | WARS2 | 0.5316 |
| ENSP00000238497 | VPS4B | 0.5316 |
| ENSP00000281187 | VPS26B | 0.5316 |
| ENSP00000378426 | VKORC1 | 0.5316 |
| ENSP00000316357 | USP9X | 0.5316 |
| ENSP00000276201 | UPF3B | 0.5316 |
| ENSP00000344259 | UBE2L3 | 0.5316 |
| ENSP00000303709 | UBE2E1 | 0.5316 |
| ENSP00000216185 | TXN2 | 0.5316 |
| ENSP00000357651 | TUBE1 | 0.5316 |
| ENSP00000298832 | TTLL5 | 0.5316 |
| ENSP00000259750 | TTBK1 | 0.5316 |
| ENSP00000273482 | TRPC1 | 0.5316 |
| ENSP00000250018 | TPH1 | 0.5316 |

| | | |
|---|---|---|
| ENSP00000221132 | TNFRSF10A | 0.5316 |
| ENSP00000262225 | TMED2 | 0.5316 |
| ENSP00000351905 | TGFBR2 | 0.5316 |
| ENSP00000374205 | TBR1 | 0.5316 |
| ENSP00000330343 | SUMO3 | 0.5316 |
| ENSP00000356540 | STX11 | 0.5316 |
| ENSP00000372689 | STAG1 | 0.5316 |
| ENSP00000265729 | SRI | 0.5316 |
| ENSP00000374455 | SQSTM1 | 0.5316 |
| ENSP00000323300 | SPAG5 | 0.5316 |
| ENSP00000293894 | SOX8 | 0.5316 |
| ENSP00000330218 | SOX1 | 0.5316 |
| ENSP00000304429 | SNX7 | 0.5316 |
| ENSP00000295951 | SLMAP | 0.5316 |
| ENSP00000337332 | SIRT6 | 0.5316 |
| ENSP00000348307 | SIRPA | 0.5316 |
| ENSP00000376793 | SERPINA3 | 0.5316 |
| ENSP00000362335 | SAR1A | 0.5316 |
| ENSP00000389182 | RPP30 | 0.5316 |
| ENSP00000328977 | RPH3AL | 0.5316 |
| ENSP00000386655 | RP1-164F3.9 | 0.5316 |
| ENSP00000392995 | RNF103-CHMP3 | 0.5316 |
| ENSP00000363787 | RING1 | 0.5316 |
| ENSP00000025008 | RB1CC1 | 0.5316 |
| ENSP00000349428 | PTBP1 | 0.5316 |
| ENSP00000386541 | PSMD14 | 0.5316 |
| ENSP00000261479 | PSMA6 | 0.5316 |
| ENSP00000457299 | PSMA1 | 0.5316 |
| ENSP00000301522 | PRDX2 | 0.5316 |
| ENSP00000261461 | PPP2R5A | 0.5316 |
| ENSP00000361918 | PPIE | 0.5316 |
| ENSP00000450710 | PPAN | 0.5316 |
| ENSP00000216180 | PNPLA3 | 0.5316 |
| ENSP00000249269 | PMPCB | 0.5316 |
| ENSP00000343325 | PKN1 | 0.5316 |
| ENSP00000237596 | PKD2 | 0.5316 |
| ENSP00000266095 | PISD | 0.5316 |
| ENSP00000362773 | PIN4 | 0.5316 |
| ENSP00000226382 | PHOX2B | 0.5316 |
| ENSP00000232375 | PFKFB4 | 0.5316 |
| ENSP00000417214 | PARP15 | 0.5316 |
| ENSP00000345395 | PAPLN | 0.5316 |
| ENSP00000302630 | ONECUT1 | 0.5316 |
| ENSP00000347626 | NUDCD3 | 0.5316 |
| ENSP00000159060 | NOX3 | 0.5316 |
| ENSP00000355133 | NOS1AP | 0.5316 |
| ENSP00000366347 | NKX2-2 | 0.5316 |
| ENSP00000341737 | NECAP1 | 0.5316 |
| ENSP00000319977 | NDRG1 | 0.5316 |
| ENSP00000439182 | MYO1H | 0.5316 |
| ENSP00000274643 | MYLK4 | 0.5316 |
| ENSP00000237500 | MYL12B | 0.5316 |
| ENSP00000263063 | MTPAP | 0.5316 |

| | | |
|---|---|---|
| ENSP00000385045 | MTA3 | 0.5316 |
| ENSP00000308897 | MRPL11 | 0.5316 |
| ENSP00000380313 | MLST8 | 0.5316 |
| ENSP00000282892 | MED21 | 0.5316 |
| ENSP00000380053 | LEUTX | 0.5316 |
| ENSP00000252242 | KRT5 | 0.5316 |
| ENSP00000424198 | KLHL2 | 0.5316 |
| ENSP00000248071 | KLF2 | 0.5316 |
| ENSP00000367828 | ITM2B | 0.5316 |
| ENSP00000349252 | ITGAL | 0.5316 |
| ENSP00000361418 | IPO13 | 0.5316 |
| ENSP00000238628 | IFT43 | 0.5316 |
| ENSP00000270139 | IFNAR1 | 0.5316 |
| ENSP00000430505 | IDO1 | 0.5316 |
| ENSP00000302961 | HSPA4 | 0.5316 |
| ENSP00000364802 | HSPA1A | 0.5316 |
| ENSP00000302548 | HOXD4 | 0.5316 |
| ENSP00000222728 | HOXA6 | 0.5316 |
| ENSP00000292432 | HK3 | 0.5316 |
| ENSP00000358571 | HIPK1 | 0.5316 |
| ENSP00000296575 | HHIP | 0.5316 |
| ENSP00000378624 | HERC4 | 0.5316 |
| ENSP00000264345 | HERC3 | 0.5316 |
| ENSP00000232854 | HEMK1 | 0.5316 |
| ENSP00000425634 | HARS | 0.5316 |
| ENSP00000364398 | HABP4 | 0.5316 |
| ENSP00000348510 | GTF3C1 | 0.5316 |
| ENSP00000156109 | GPKOW | 0.5316 |
| ENSP00000313869 | GORASP1 | 0.5316 |
| ENSP00000395772 | GNL3 | 0.5316 |
| ENSP00000363489 | GDF5 | 0.5316 |
| ENSP00000222214 | GCDH | 0.5316 |
| ENSP00000348578 | G3BP1 | 0.5316 |
| ENSP00000371940 | FOXD4 | 0.5316 |
| ENSP00000355518 | FH | 0.5316 |
| ENSP00000228837 | FGF6 | 0.5316 |
| ENSP00000283268 | FEZF2 | 0.5316 |
| ENSP00000393776 | FCHO2 | 0.5316 |
| ENSP00000428205 | FBXO32 | 0.5316 |
| ENSP00000315476 | EXOSC4 | 0.5316 |
| ENSP00000337451 | EPHA3 | 0.5316 |
| ENSP00000435619 | EID3 | 0.5316 |
| ENSP00000380308 | DYNC1I2 | 0.5316 |
| ENSP00000276420 | DOK2 | 0.5316 |
| ENSP00000368523 | DNAJC15 | 0.5316 |
| ENSP00000369127 | DNAJA1 | 0.5316 |
| ENSP00000382133 | DNA2 | 0.5316 |
| ENSP00000398495 | DIABLO | 0.5316 |
| ENSP00000268482 | DHX38 | 0.5316 |
| ENSP00000310723 | DDX23 | 0.5316 |
| ENSP00000363840 | COL11A2 | 0.5316 |
| ENSP00000305449 | CNNM3 | 0.5316 |
| ENSP00000303585 | CLIP1 | 0.5316 |

| | | |
|---|---|---|
| ENSP00000392028 | CHD7 | 0.5316 |
| ENSP00000338673 | CDK10 | 0.5316 |
| ENSP00000364212 | CDA | 0.5316 |
| ENSP00000264246 | CD80 | 0.5316 |
| ENSP00000359866 | BMP5 | 0.5316 |
| ENSP00000393596 | BIRC6 | 0.5316 |
| ENSP00000352219 | BCS1L | 0.5316 |
| ENSP00000309103 | BAD | 0.5316 |
| ENSP00000276390 | ATP6V1B2 | 0.5316 |
| ENSP00000300688 | ATP5L | 0.5316 |
| ENSP00000324172 | ATP2B2 | 0.5316 |
| ENSP00000261879 | APH1B | 0.5316 |
| ENSP00000257497 | ANXA1 | 0.5316 |
| ENSP00000324074 | ANP32E | 0.5316 |
| ENSP00000377083 | ALDH1L1 | 0.5316 |
| ENSP00000285930 | AKR1B1 | 0.5316 |
| ENSP00000231420 | AGXT2 | 0.5316 |
| ENSP00000333664 | ACAA1 | 0.5316 |
| ENSP00000225941 | ABI3 | 0.5316 |
| ENSP00000374467 | ABCC8 | 0.5316 |
| ENSP00000265723 | ABCB4 | 0.5316 |
| ENSP00000338160 | FKBP5 | 0.5316 |
| ENSP00000227322 | ZNF259 | 0.5289 |
| ENSP00000280193 | VEGFC | 0.5289 |
| ENSP00000340596 | UBE3B | 0.5289 |
| ENSP00000340305 | UBE2V1 | 0.5289 |
| ENSP00000349722 | UBE2D3 | 0.5289 |
| ENSP00000264029 | TREH | 0.5289 |
| ENSP00000238788 | TMEM214 | 0.5289 |
| ENSP00000354932 | TLR1 | 0.5289 |
| ENSP00000380969 | THOC5 | 0.5289 |
| ENSP00000265097 | THOC3 | 0.5289 |
| ENSP00000307260 | TBL2 | 0.5289 |
| ENSP00000346139 | TBC1D20 | 0.5289 |
| ENSP00000347792 | SYNJ2 | 0.5289 |
| ENSP00000376178 | STX2 | 0.5289 |
| ENSP00000215095 | STX1B | 0.5289 |
| ENSP00000282908 | STK32B | 0.5289 |
| ENSP00000322977 | SSBP2 | 0.5289 |
| ENSP00000309945 | SPNS1 | 0.5289 |
| ENSP00000371542 | SPATA13 | 0.5289 |
| ENSP00000430333 | SLIT3 | 0.5289 |
| ENSP00000222248 | SLC5A5 | 0.5289 |
| ENSP00000371535 | SEPSECS | 0.5289 |
| ENSP00000396320 | SCN4A | 0.5289 |
| ENSP00000310448 | SART1 | 0.5289 |
| ENSP00000352608 | RYR1 | 0.5289 |
| ENSP00000419494 | RYBP | 0.5289 |
| ENSP00000345957 | RPS21 | 0.5289 |
| ENSP00000346022 | RPL9 | 0.5289 |
| ENSP00000294189 | RPL29 | 0.5289 |
| ENSP00000466829 | RP11-318A15.7 | 0.5289 |
| ENSP00000342755 | RNF41 | 0.5289 |

| | | |
|---|---|---|
| ENSP00000296271 | RHO | 0.5289 |
| ENSP00000269051 | RHBDL3 | 0.5289 |
| ENSP00000289248 | RHBDL2 | 0.5289 |
| ENSP00000262316 | RHBDF1 | 0.5289 |
| ENSP00000386810 | RGPD4 | 0.5289 |
| ENSP00000253363 | RBM39 | 0.5289 |
| ENSP00000366829 | RBM10 | 0.5289 |
| ENSP00000393262 | RBFOX3 | 0.5289 |
| ENSP00000259569 | RANBP6 | 0.5289 |
| ENSP00000351284 | RAD52 | 0.5289 |
| ENSP00000350708 | RAD23B | 0.5289 |
| ENSP00000378313 | RAB3IL1 | 0.5289 |
| ENSP00000377047 | PTPRZ1 | 0.5289 |
| ENSP00000400010 | PTPRJ | 0.5289 |
| ENSP00000418112 | PTPRG | 0.5289 |
| ENSP00000303424 | PTGDR | 0.5289 |
| ENSP00000351314 | PSMB10 | 0.5289 |
| ENSP00000262848 | PRKX | 0.5289 |
| ENSP00000345064 | PRICKLE1 | 0.5289 |
| ENSP00000234310 | PPP3R1 | 0.5289 |
| ENSP00000362296 | POU3F4 | 0.5289 |
| ENSP00000301788 | POLR2G | 0.5289 |
| ENSP00000216259 | PMM1 | 0.5289 |
| ENSP00000366977 | PLEKHG5 | 0.5289 |
| ENSP00000220809 | PLAT | 0.5289 |
| ENSP00000244137 | PEPD | 0.5289 |
| ENSP00000365388 | PDSS1 | 0.5289 |
| ENSP00000360327 | PARS2 | 0.5289 |
| ENSP00000354681 | OPA1 | 0.5289 |
| ENSP00000388001 | OAS1 | 0.5289 |
| ENSP00000285968 | NUP205 | 0.5289 |
| ENSP00000358019 | NSMCE4A | 0.5289 |
| ENSP00000355944 | NSL1 | 0.5289 |
| ENSP00000348460 | NPAS3 | 0.5289 |
| ENSP00000370215 | NOXRED1 | 0.5289 |
| ENSP00000013034 | NME1 | 0.5289 |
| ENSP00000268459 | NKD1 | 0.5289 |
| ENSP00000431482 | NEDD8-MDP1 | 0.5289 |
| ENSP00000432614 | NDUFC2-KCTD14 | 0.5289 |
| ENSP00000299166 | NDUFB8 | 0.5289 |
| ENSP00000281038 | NARS2 | 0.5289 |
| ENSP00000301972 | MYRIP | 0.5289 |
| ENSP00000081029 | MRPS35 | 0.5289 |
| ENSP00000372093 | MRPL36 | 0.5289 |
| ENSP00000315017 | MRPL1 | 0.5289 |
| ENSP00000264605 | MLPH | 0.5289 |
| ENSP00000347427 | MINK1 | 0.5289 |
| ENSP00000215957 | MICALL1 | 0.5289 |
| ENSP00000456533 | MFRP | 0.5289 |
| ENSP00000342392 | MESP2 | 0.5289 |
| ENSP00000363193 | MED12 | 0.5289 |
| ENSP00000401980 | MAVS | 0.5289 |
| ENSP00000226578 | MANBA | 0.5289 |

| | | |
|---|---|---|
| ENSP00000267978 | MAN2C1 | 0.5289 |
| ENSP00000319240 | MAGOHB | 0.5289 |
| ENSP00000298910 | LRRK2 | 0.5289 |
| ENSP00000377086 | LONRF2 | 0.5289 |
| ENSP00000261434 | LIAS | 0.5289 |
| ENSP00000263726 | LHX4 | 0.5289 |
| ENSP00000262776 | LGALS3BP | 0.5289 |
| ENSP00000253339 | LATS1 | 0.5289 |
| ENSP00000408411 | LAMP2 | 0.5289 |
| ENSP00000318016 | KIAA0196 | 0.5289 |
| ENSP00000432786 | KDM2A | 0.5289 |
| ENSP00000366682 | IRG1 | 0.5289 |
| ENSP00000297185 | HSPA9 | 0.5289 |
| ENSP00000348170 | HP | 0.5289 |
| ENSP00000365402 | HLA-C | 0.5289 |
| ENSP00000399168 | HLA-B | 0.5289 |
| ENSP00000264346 | HERC6 | 0.5289 |
| ENSP00000229330 | HCFC2 | 0.5289 |
| ENSP00000306991 | HAS2 | 0.5289 |
| ENSP00000373477 | GPX3 | 0.5289 |
| ENSP00000245206 | GOT2 | 0.5289 |
| ENSP00000222286 | GAPDHS | 0.5289 |
| ENSP00000340466 | GANAB | 0.5289 |
| ENSP00000296839 | FOXQ1 | 0.5289 |
| ENSP00000366637 | FOXD4L5 | 0.5289 |
| ENSP00000297904 | FIGF | 0.5289 |
| ENSP00000304592 | FASN | 0.5289 |
| ENSP00000221233 | EXOSC5 | 0.5289 |
| ENSP00000358309 | EPHA7 | 0.5289 |
| ENSP00000229277 | ENO2 | 0.5289 |
| ENSP00000338020 | EIF4G1 | 0.5289 |
| ENSP00000273783 | EIF2B5 | 0.5289 |
| ENSP00000398124 | E2F5 | 0.5289 |
| ENSP00000404079 | DNAJB5 | 0.5289 |
| ENSP00000312697 | DMAP1 | 0.5289 |
| ENSP00000377344 | DDX60 | 0.5289 |
| ENSP00000250863 | DAZL | 0.5289 |
| ENSP00000287490 | COX6A2 | 0.5289 |
| ENSP00000285896 | CNOT8 | 0.5289 |
| ENSP00000272133 | CNIH3 | 0.5289 |
| ENSP00000271636 | CGN | 0.5289 |
| ENSP00000215980 | CENPM | 0.5289 |
| ENSP00000380718 | CDYL | 0.5289 |
| ENSP00000464036 | CDK11B | 0.5289 |
| ENSP00000353656 | CDH4 | 0.5289 |
| ENSP00000363836 | CCNY | 0.5289 |
| ENSP00000344549 | CARD14 | 0.5289 |
| ENSP00000296452 | BSN | 0.5289 |
| ENSP00000314792 | BOLL | 0.5289 |
| ENSP00000252677 | BMP15 | 0.5289 |
| ENSP00000263182 | BBOX1 | 0.5289 |
| ENSP00000295598 | ATP1A1 | 0.5289 |
| ENSP00000175506 | ASNS | 0.5289 |

| | | |
|---|---|---|
| ENSP00000347602 | ARID4A | 0.5289 |
| ENSP00000236850 | APOA1 | 0.5289 |
| ENSP00000233607 | APC2 | 0.5289 |
| ENSP00000261558 | AP5M1 | 0.5289 |
| ENSP00000297183 | ANKHD1 | 0.5289 |
| ENSP00000261819 | ANAPC5 | 0.5289 |
| ENSP00000262374 | ALG1 | 0.5289 |
| ENSP00000302620 | AGXT | 0.5289 |
| ENSP00000312126 | ADCY4 | 0.5289 |
| ENSP00000316924 | ACSS1 | 0.5289 |
| ENSP00000293217 | ACOX1 | 0.5289 |
| ENSP00000341044 | ACACB | 0.5289 |
| ENSP00000222388 | ABCF2 | 0.5289 |
| ENSP00000380795 | VPS29 | 0.5289 |
| ENSP00000402084 | WEE1 | 0.5263 |
| ENSP00000363308 | WDR31 | 0.5263 |
| ENSP00000428845 | VDAC3 | 0.5263 |
| ENSP00000215794 | USP18 | 0.5263 |
| ENSP00000261601 | USP14 | 0.5263 |
| ENSP00000319501 | UGDH | 0.5263 |
| ENSP00000313454 | UBA6 | 0.5263 |
| ENSP00000287078 | TYSND1 | 0.5263 |
| ENSP00000318697 | TUBB6 | 0.5263 |
| ENSP00000381003 | TTC28 | 0.5263 |
| ENSP00000360552 | TSHZ2 | 0.5263 |
| ENSP00000260810 | TOPBP1 | 0.5263 |
| ENSP00000413697 | TOM1 | 0.5263 |
| ENSP00000276431 | TNFRSF10B | 0.5263 |
| ENSP00000266085 | TIMP3 | 0.5263 |
| ENSP00000218388 | TIMP1 | 0.5263 |
| ENSP00000438455 | TIMM8B | 0.5263 |
| ENSP00000245838 | THOC2 | 0.5263 |
| ENSP00000346691 | TBC1D3 | 0.5263 |
| ENSP00000346997 | SUV39H2 | 0.5263 |
| ENSP00000344742 | STAMBP | 0.5263 |
| ENSP00000360371 | SSBP3 | 0.5263 |
| ENSP00000382518 | SRL | 0.5263 |
| ENSP00000263672 | SPCS2 | 0.5263 |
| ENSP00000376024 | SNX9 | 0.5263 |
| ENSP00000254193 | SNRPA1 | 0.5263 |
| ENSP00000304360 | SMYD4 | 0.5263 |
| ENSP00000266058 | SLIT1 | 0.5263 |
| ENSP00000345580 | SLC25A10 | 0.5263 |
| ENSP00000368552 | SIRT5 | 0.5263 |
| ENSP00000293441 | SHANK1 | 0.5263 |
| ENSP00000302913 | SH3D19 | 0.5263 |
| ENSP00000321221 | SH2B1 | 0.5263 |
| ENSP00000301037 | SGK494 | 0.5263 |
| ENSP00000263686 | SELP | 0.5263 |
| ENSP00000254351 | SDC1 | 0.5263 |
| ENSP00000360938 | SARDH | 0.5263 |
| ENSP00000196551 | RPS5 | 0.5263 |
| ENSP00000450730 | RP11-934B9.3 | 0.5263 |

| | | |
|---|---|---|
| ENSP00000268125 | RLBP1 | 0.5263 |
| ENSP00000369318 | RHOBTB3 | 0.5263 |
| ENSP00000343054 | RBM5 | 0.5263 |
| ENSP00000360286 | RAE1 | 0.5263 |
| ENSP00000216962 | PYGB | 0.5263 |
| ENSP00000227474 | PUS3 | 0.5263 |
| ENSP00000283228 | PTPRR | 0.5263 |
| ENSP00000298852 | PSMC3 | 0.5263 |
| ENSP00000261303 | PSMC1 | 0.5263 |
| ENSP00000261454 | PROX1 | 0.5263 |
| ENSP00000333551 | PROSC | 0.5263 |
| ENSP00000343924 | PRELP | 0.5263 |
| ENSP00000265462 | PRDX5 | 0.5263 |
| ENSP00000368646 | PRDX4 | 0.5263 |
| ENSP00000329867 | PPME1 | 0.5263 |
| ENSP00000282412 | PPM1B | 0.5263 |
| ENSP00000292614 | POLR2J | 0.5263 |
| ENSP00000361465 | POLR1C | 0.5263 |
| ENSP00000360782 | PMPCA | 0.5263 |
| ENSP00000339328 | PLAUR | 0.5263 |
| ENSP00000298282 | PKNOX2 | 0.5263 |
| ENSP00000371393 | PGM2 | 0.5263 |
| ENSP00000363734 | PFDN6 | 0.5263 |
| ENSP00000272227 | PDIA6 | 0.5263 |
| ENSP00000227868 | PDHX | 0.5263 |
| ENSP00000264933 | PDCD6 | 0.5263 |
| ENSP00000358812 | PDCD11 | 0.5263 |
| ENSP00000193322 | OSTM1 | 0.5263 |
| ENSP00000224950 | OBFC1 | 0.5263 |
| ENSP00000342262 | NUP43 | 0.5263 |
| ENSP00000300291 | NUDT21 | 0.5263 |
| ENSP00000235628 | NT5C1A | 0.5263 |
| ENSP00000354929 | NOTCH2NL | 0.5263 |
| ENSP00000301457 | NDUFA7 | 0.5263 |
| ENSP00000423673 | NDUFA13 | 0.5263 |
| ENSP00000401508 | NAPRT1 | 0.5263 |
| ENSP00000258787 | MYO1G | 0.5263 |
| ENSP00000217652 | MYL12A | 0.5263 |
| ENSP00000262101 | MSR1 | 0.5263 |
| ENSP00000337907 | MPP7 | 0.5263 |
| ENSP00000387278 | MPP4 | 0.5263 |
| ENSP00000269095 | MPP2 | 0.5263 |
| ENSP00000452854 | MEIS3 | 0.5263 |
| ENSP00000350639 | MAPKAPK3 | 0.5263 |
| ENSP00000265960 | MAPKAP1 | 0.5263 |
| ENSP00000375986 | MAP3K4 | 0.5263 |
| ENSP00000285928 | LRGUK | 0.5263 |
| ENSP00000309757 | LPL | 0.5263 |
| ENSP00000372035 | LATS2 | 0.5263 |
| ENSP00000258341 | LAMC1 | 0.5263 |
| ENSP00000329471 | KDELR1 | 0.5263 |
| ENSP00000355402 | ISLR2 | 0.5263 |
| ENSP00000261574 | IPO5 | 0.5263 |

| | | |
|---|---|---|
| ENSP00000329553 | IMMP2L | 0.5263 |
| ENSP00000264497 | IL21 | 0.5263 |
| ENSP00000403103 | IL12RB1 | 0.5263 |
| ENSP00000457706 | HSPB2-C11orf52 | 0.5263 |
| ENSP00000364412 | HSD17B3 | 0.5263 |
| ENSP00000216027 | HSCB | 0.5263 |
| ENSP00000381237 | HM13 | 0.5263 |
| ENSP00000332194 | HIST2H2AC | 0.5263 |
| ENSP00000413520 | HID1 | 0.5263 |
| ENSP00000317904 | GYS1 | 0.5263 |
| ENSP00000354003 | GYPA | 0.5263 |
| ENSP00000221130 | GSR | 0.5263 |
| ENSP00000374265 | GPX2 | 0.5263 |
| ENSP00000282541 | GPD1L | 0.5263 |
| ENSP00000234160 | GORASP2 | 0.5263 |
| ENSP00000315925 | GMPPA | 0.5263 |
| ENSP00000385721 | GGT2 | 0.5263 |
| ENSP00000226796 | GAR1 | 0.5263 |
| ENSP00000291670 | FTCD | 0.5263 |
| ENSP00000295113 | FRZB | 0.5263 |
| ENSP00000326272 | FOXL1 | 0.5263 |
| ENSP00000259806 | FOXF2 | 0.5263 |
| ENSP00000365301 | FGF14 | 0.5263 |
| ENSP00000366135 | EXOSC10 | 0.5263 |
| ENSP00000323714 | EIF4E1B | 0.5263 |
| ENSP00000309953 | EFEMP2 | 0.5263 |
| ENSP00000380054 | EAF1 | 0.5263 |
| ENSP00000354111 | DNAJC5 | 0.5263 |
| ENSP00000455744 | DKFZP686D09174 | 0.5263 |
| ENSP00000336741 | DHX15 | 0.5263 |
| ENSP00000265022 | DGKG | 0.5263 |
| ENSP00000263209 | DGCR8 | 0.5263 |
| ENSP00000259512 | DERL1 | 0.5263 |
| ENSP00000292782 | DCUN1D1 | 0.5263 |
| ENSP00000394484 | DCLK3 | 0.5263 |
| ENSP00000358903 | CYP17A1 | 0.5263 |
| ENSP00000361562 | CTSA | 0.5263 |
| ENSP00000359976 | CTH | 0.5263 |
| ENSP00000300527 | COL6A2 | 0.5263 |
| ENSP00000303153 | COL22A1 | 0.5263 |
| ENSP00000341882 | CNTN6 | 0.5263 |
| ENSP00000229329 | CMAS | 0.5263 |
| ENSP00000284049 | CHD1 | 0.5263 |
| ENSP00000370297 | CER1 | 0.5263 |
| ENSP00000336868 | CENPA | 0.5263 |
| ENSP00000419879 | CDS2 | 0.5263 |
| ENSP00000355237 | CDC42BPB | 0.5263 |
| ENSP00000363708 | BMPR2 | 0.5263 |
| ENSP00000355231 | BECN1 | 0.5263 |
| ENSP00000368414 | BCAP29 | 0.5263 |
| ENSP00000370718 | ASMTL | 0.5263 |
| ENSP00000328789 | AP1S2 | 0.5263 |
| ENSP00000265132 | AMBP | 0.5263 |

| | | |
|---|---|---|
| ENSP00000336927 | ALDOA | 0.5263 |
| ENSP00000346827 | ALDH9A1 | 0.5263 |
| ENSP00000371230 | AK3 | 0.5263 |
| ENSP00000362306 | AGO4 | 0.5263 |
| ENSP00000342952 | ADCY2 | 0.5263 |
| ENSP00000309477 | ACO1 | 0.5263 |
| ENSP00000222800 | ABHD11 | 0.5263 |
| ENSP00000411471 | ABCF3 | 0.5263 |
| ENSP00000434236 | SUGT1P3 | 0.5263 |
| ENSP00000261980 | VSX2 | 0.5237 |
| ENSP00000379602 | VAMP1 | 0.5237 |
| ENSP00000328939 | UTY | 0.5237 |
| ENSP00000400312 | ULK3 | 0.5237 |
| ENSP00000365105 | TTLL9 | 0.5237 |
| ENSP00000433757 | TTC12 | 0.5237 |
| ENSP00000261740 | TRPV4 | 0.5237 |
| ENSP00000362026 | TRMT2B | 0.5237 |
| ENSP00000261464 | TRAF5 | 0.5237 |
| ENSP00000356966 | TOMM40L | 0.5237 |
| ENSP00000360689 | TNKS2 | 0.5237 |
| ENSP00000297350 | TNFRSF11B | 0.5237 |
| ENSP00000420588 | TFAM | 0.5237 |
| ENSP00000309913 | TBX5 | 0.5237 |
| ENSP00000040877 | TARBP1 | 0.5237 |
| ENSP00000296946 | T | 0.5237 |
| ENSP00000394700 | SYT16 | 0.5237 |
| ENSP00000392270 | STRAP | 0.5237 |
| ENSP00000267540 | STON2 | 0.5237 |
| ENSP00000348886 | STOML2 | 0.5237 |
| ENSP00000410452 | STMN1 | 0.5237 |
| ENSP00000301740 | SRRM2 | 0.5237 |
| ENSP00000260324 | SQRDL | 0.5237 |
| ENSP00000305494 | SPATA5L1 | 0.5237 |
| ENSP00000215829 | SNRPD3 | 0.5237 |
| ENSP00000354720 | SMC3 | 0.5237 |
| ENSP00000306190 | SLAMF1 | 0.5237 |
| ENSP00000262018 | SGCA | 0.5237 |
| ENSP00000391311 | SEPT5 | 0.5237 |
| ENSP00000310521 | SEC22A | 0.5237 |
| ENSP00000355499 | SDCCAG8 | 0.5237 |
| ENSP00000344468 | SDC3 | 0.5237 |
| ENSP00000317224 | SAMD4B | 0.5237 |
| ENSP00000251810 | RRM2B | 0.5237 |
| ENSP00000298317 | RPUSD4 | 0.5237 |
| ENSP00000323288 | RPUSD2 | 0.5237 |
| ENSP00000339095 | RPS7 | 0.5237 |
| ENSP00000339795 | RPL7 | 0.5237 |
| ENSP00000274242 | RPL37 | 0.5237 |
| ENSP00000391266 | RP1-241P17.4 | 0.5237 |
| ENSP00000315212 | RNF4 | 0.5237 |
| ENSP00000428417 | RIMS1 | 0.5237 |
| ENSP00000333194 | RGS19 | 0.5237 |
| ENSP00000368295 | REXO1L1 | 0.5237 |

| | | |
|---|---|---|
| ENSP00000309117 | RBFOX1 | 0.5237 |
| ENSP00000358549 | RARS2 | 0.5237 |
| ENSP00000430128 | RALYL | 0.5237 |
| ENSP00000297338 | RAD21 | 0.5237 |
| ENSP00000355094 | QKI | 0.5237 |
| ENSP00000369756 | PTPRA | 0.5237 |
| ENSP00000248594 | PTPN12 | 0.5237 |
| ENSP00000386621 | PTGES3L-AARSD1 | 0.5237 |
| ENSP00000219313 | PSMD7 | 0.5237 |
| ENSP00000223321 | PSMA2 | 0.5237 |
| ENSP00000234347 | PRTN3 | 0.5237 |
| ENSP00000287878 | PRKAG2 | 0.5237 |
| ENSP00000295902 | PRICKLE2 | 0.5237 |
| ENSP00000335614 | PPFIA3 | 0.5237 |
| ENSP00000377385 | PPAN-P2RY11 | 0.5237 |
| ENSP00000343885 | PPA2 | 0.5237 |
| ENSP00000406046 | POLD1 | 0.5237 |
| ENSP00000273077 | PNKD | 0.5237 |
| ENSP00000406490 | PMS1 | 0.5237 |
| ENSP00000420132 | PLSCR2 | 0.5237 |
| ENSP00000223127 | PLOD3 | 0.5237 |
| ENSP00000262300 | PKMYT1 | 0.5237 |
| ENSP00000264380 | PIKFYVE | 0.5237 |
| ENSP00000329097 | PHF6 | 0.5237 |
| ENSP00000368682 | PHEX | 0.5237 |
| ENSP00000425809 | PGM3 | 0.5237 |
| ENSP00000356989 | PFDN2 | 0.5237 |
| ENSP00000301396 | PELP1 | 0.5237 |
| ENSP00000303427 | PDS5A | 0.5237 |
| ENSP00000384515 | PARVB | 0.5237 |
| ENSP00000360672 | PARD6B | 0.5237 |
| ENSP00000297431 | ORC5 | 0.5237 |
| ENSP00000394624 | OPRM1 | 0.5237 |
| ENSP00000369136 | NWD1 | 0.5237 |
| ENSP00000367721 | NUP160 | 0.5237 |
| ENSP00000258960 | NMT1 | 0.5237 |
| ENSP00000299872 | NGFRAP1 | 0.5237 |
| ENSP00000360231 | NFIA | 0.5237 |
| ENSP00000386461 | MYO7B | 0.5237 |
| ENSP00000415183 | MUC2 | 0.5237 |
| ENSP00000257552 | MSI1 | 0.5237 |
| ENSP00000333837 | MRPL12 | 0.5237 |
| ENSP00000250156 | MRM1 | 0.5237 |
| ENSP00000222644 | MPP6 | 0.5237 |
| ENSP00000381425 | MPP3 | 0.5237 |
| ENSP00000308351 | MLKL | 0.5237 |
| ENSP00000246062 | MKKS | 0.5237 |
| ENSP00000263881 | MAP4K3 | 0.5237 |
| ENSP00000260665 | LRPPRC | 0.5237 |
| ENSP00000264162 | LCT | 0.5237 |
| ENSP00000254043 | KRT15 | 0.5237 |
| ENSP00000388241 | KIF26A | 0.5237 |
| ENSP00000371587 | KCNIP4 | 0.5237 |

| | | |
|---|---|---|
| ENSP00000335106 | KATNA1 | 0.5237 |
| ENSP00000452120 | ITGA7 | 0.5237 |
| ENSP00000364805 | HSPA1L | 0.5237 |
| ENSP00000308944 | HLTF | 0.5237 |
| ENSP00000295488 | HELQ | 0.5237 |
| ENSP00000423563 | H2AFY | 0.5237 |
| ENSP00000276414 | GNRH1 | 0.5237 |
| ENSP00000312999 | GNAI2 | 0.5237 |
| ENSP00000300648 | GCN1L1 | 0.5237 |
| ENSP00000387662 | GCG | 0.5237 |
| ENSP00000225614 | GALK1 | 0.5237 |
| ENSP00000363621 | GALE | 0.5237 |
| ENSP00000354607 | FZD5 | 0.5237 |
| ENSP00000427329 | FOXR2 | 0.5237 |
| ENSP00000302756 | FOXD4L1 | 0.5237 |
| ENSP00000336829 | FGG | 0.5237 |
| ENSP00000287647 | FANCD2 | 0.5237 |
| ENSP00000166244 | EPHA8 | 0.5237 |
| ENSP00000337168 | EPB41L1 | 0.5237 |
| ENSP00000429986 | ENY2 | 0.5237 |
| ENSP00000345555 | ENO4 | 0.5237 |
| ENSP00000220849 | EIF3E | 0.5237 |
| ENSP00000363680 | EDA | 0.5237 |
| ENSP00000311977 | DUS3L | 0.5237 |
| ENSP00000237449 | DNAH6 | 0.5237 |
| ENSP00000416583 | DERA | 0.5237 |
| ENSP00000301264 | DAPK3 | 0.5237 |
| ENSP00000297044 | CYTH3 | 0.5237 |
| ENSP00000308928 | CYLD | 0.5237 |
| ENSP00000324549 | CYFIP1 | 0.5237 |
| ENSP00000286758 | CXCL13 | 0.5237 |
| ENSP00000361699 | CTPS1 | 0.5237 |
| ENSP00000217244 | CSNK2A1 | 0.5237 |
| ENSP00000299529 | CRABP1 | 0.5237 |
| ENSP00000332371 | COL7A1 | 0.5237 |
| ENSP00000419974 | CLASP2 | 0.5237 |
| ENSP00000223500 | CHMP5 | 0.5237 |
| ENSP00000298159 | CFL2 | 0.5237 |
| ENSP00000362018 | CENPI | 0.5237 |
| ENSP00000372319 | CDY2B | 0.5237 |
| ENSP00000302968 | CDY1 | 0.5237 |
| ENSP00000384442 | CDK11A | 0.5237 |
| ENSP00000319166 | CDH7 | 0.5237 |
| ENSP00000368450 | CD83 | 0.5237 |
| ENSP00000300105 | CACNG2 | 0.5237 |
| ENSP00000318128 | BLOC1S4 | 0.5237 |
| ENSP00000326391 | BHLHA15 | 0.5237 |
| ENSP00000343313 | ATG5 | 0.5237 |
| ENSP00000310071 | ANAPC10 | 0.5237 |
| ENSP00000430236 | ALG11 | 0.5237 |
| ENSP00000307634 | AGAP1 | 0.5237 |
| ENSP00000289228 | ACTR1B | 0.5237 |
| ENSP00000160382 | ACTL6B | 0.5237 |

| | | |
|---|---|---|
| ENSP00000313603 | ABCF1 | 0.5237 |
| ENSP00000253699 | ZFYVE20 | 0.5211 |
| ENSP00000343392 | XRCC3 | 0.5211 |
| ENSP00000262887 | XRCC1 | 0.5211 |
| ENSP00000319474 | WSB2 | 0.5211 |
| ENSP00000346829 | WLS | 0.5211 |
| ENSP00000307491 | WDR48 | 0.5211 |
| ENSP00000298767 | WAPAL | 0.5211 |
| ENSP00000345656 | VAPA | 0.5211 |
| ENSP00000381577 | USP34 | 0.5211 |
| ENSP00000324343 | UPB1 | 0.5211 |
| ENSP00000237014 | TTR | 0.5211 |
| ENSP00000347733 | TRRAP | 0.5211 |
| ENSP00000317891 | TNIP1 | 0.5211 |
| ENSP00000301634 | TK1 | 0.5211 |
| ENSP00000361993 | TIMM8A | 0.5211 |
| ENSP00000258975 | TACO1 | 0.5211 |
| ENSP00000366395 | SYVN1 | 0.5211 |
| ENSP00000376472 | STT3A | 0.5211 |
| ENSP00000325748 | STK25 | 0.5211 |
| ENSP00000356591 | SOAT1 | 0.5211 |
| ENSP00000378414 | SMARCD1 | 0.5211 |
| ENSP00000358770 | SLK | 0.5211 |
| ENSP00000422591 | SLIT2 | 0.5211 |
| ENSP00000408005 | SLC9A3R2 | 0.5211 |
| ENSP00000263980 | SLC9A1 | 0.5211 |
| ENSP00000233202 | SLC11A1 | 0.5211 |
| ENSP00000304502 | SIX2 | 0.5211 |
| ENSP00000370395 | SGTB | 0.5211 |
| ENSP00000366604 | SF1 | 0.5211 |
| ENSP00000265175 | SEC24B | 0.5211 |
| ENSP00000283256 | SCN2A | 0.5211 |
| ENSP00000385432 | SAR1B | 0.5211 |
| ENSP00000373713 | SACM1L | 0.5211 |
| ENSP00000291700 | S100B | 0.5211 |
| ENSP00000318646 | RPS15A | 0.5211 |
| ENSP00000404375 | RPL36A | 0.5211 |
| ENSP00000447000 | RP11-762I7.5 | 0.5211 |
| ENSP00000307971 | RFNG | 0.5211 |
| ENSP00000216225 | RBX1 | 0.5211 |
| ENSP00000413035 | RBFOX2 | 0.5211 |
| ENSP00000011619 | RANBP9 | 0.5211 |
| ENSP00000467024 | RAD23A | 0.5211 |
| ENSP00000317473 | RABGGTB | 0.5211 |
| ENSP00000282091 | PTH | 0.5211 |
| ENSP00000261712 | PSMD11 | 0.5211 |
| ENSP00000386458 | PRPF40A | 0.5211 |
| ENSP00000359095 | PRMT6 | 0.5211 |
| ENSP00000350491 | PRIM1 | 0.5211 |
| ENSP00000216367 | POLE2 | 0.5211 |
| ENSP00000316021 | PLSCR3 | 0.5211 |
| ENSP00000386733 | PLEKHG2 | 0.5211 |
| ENSP00000294964 | PKDCC | 0.5211 |

| | | |
|---|---|---|
| ENSP00000265970 | PIK3C2A | 0.5211 |
| ENSP00000226319 | PHF17 | 0.5211 |
| ENSP00000257118 | PHC2 | 0.5211 |
| ENSP00000300408 | PHB | 0.5211 |
| ENSP00000262764 | PGS1 | 0.5211 |
| ENSP00000239940 | PFN2 | 0.5211 |
| ENSP00000310661 | PDE7B | 0.5211 |
| ENSP00000266395 | PDE6H | 0.5211 |
| ENSP00000442050 | PCYT2 | 0.5211 |
| ENSP00000406157 | PAPSS2 | 0.5211 |
| ENSP00000265174 | PAPSS1 | 0.5211 |
| ENSP00000347379 | OCLN | 0.5211 |
| ENSP00000352839 | NPSR1 | 0.5211 |
| ENSP00000370589 | NOP56 | 0.5211 |
| ENSP00000340925 | NADK | 0.5211 |
| ENSP00000260950 | MSTN | 0.5211 |
| ENSP00000314441 | METTL1 | 0.5211 |
| ENSP00000308546 | MEPCE | 0.5211 |
| ENSP00000348982 | MEGF6 | 0.5211 |
| ENSP00000343657 | MCCC2 | 0.5211 |
| ENSP00000355884 | MARK1 | 0.5211 |
| ENSP00000250894 | MAPK8IP3 | 0.5211 |
| ENSP00000298288 | LRR1 | 0.5211 |
| ENSP00000421922 | LRPAP1 | 0.5211 |
| ENSP00000231368 | LNPEP | 0.5211 |
| ENSP00000252244 | KRT1 | 0.5211 |
| ENSP00000361298 | KIF2C | 0.5211 |
| ENSP00000296181 | ITGB5 | 0.5211 |
| ENSP00000378295 | INCENP | 0.5211 |
| ENSP00000296266 | IFT122 | 0.5211 |
| ENSP00000305919 | HTRA4 | 0.5211 |
| ENSP00000366898 | HIF3A | 0.5211 |
| ENSP00000361943 | HEYL | 0.5211 |
| ENSP00000385638 | HADH | 0.5211 |
| ENSP00000262903 | HACE1 | 0.5211 |
| ENSP00000283875 | GTF2E1 | 0.5211 |
| ENSP00000374390 | GPR149 | 0.5211 |
| ENSP00000405573 | GPI | 0.5211 |
| ENSP00000392859 | GMPR2 | 0.5211 |
| ENSP00000351706 | GK2 | 0.5211 |
| ENSP00000265857 | GET4 | 0.5211 |
| ENSP00000394071 | GDI1 | 0.5211 |
| ENSP00000377192 | G6PD | 0.5211 |
| ENSP00000256759 | FST | 0.5211 |
| ENSP00000261302 | FOXN3 | 0.5211 |
| ENSP00000359693 | FKBP1C | 0.5211 |
| ENSP00000260270 | FDX1 | 0.5211 |
| ENSP00000349078 | FDPS | 0.5211 |
| ENSP00000369666 | FBXL19 | 0.5211 |
| ENSP00000416387 | FBLIM1 | 0.5211 |
| ENSP00000267430 | FANCM | 0.5211 |
| ENSP00000033079 | FAM13B | 0.5211 |
| ENSP00000253496 | F12 | 0.5211 |

| | | |
|---|---|---|
| ENSP00000364709 | F10 | 0.5211 |
| ENSP00000361725 | ENDOG | 0.5211 |
| ENSP00000304736 | ELOVL6 | 0.5211 |
| ENSP00000258416 | EIF4E2 | 0.5211 |
| ENSP00000318057 | EGR3 | 0.5211 |
| ENSP00000224073 | EDF1 | 0.5211 |
| ENSP00000357535 | ECHS1 | 0.5211 |
| ENSP00000279488 | DUSP6 | 0.5211 |
| ENSP00000373169 | DRD3 | 0.5211 |
| ENSP00000353731 | DPP4 | 0.5211 |
| ENSP00000262415 | DHX8 | 0.5211 |
| ENSP00000286234 | DEPTOR | 0.5211 |
| ENSP00000280557 | DENR | 0.5211 |
| ENSP00000250498 | DAD1 | 0.5211 |
| ENSP00000398736 | CYR61 | 0.5211 |
| ENSP00000409075 | CYB5RL | 0.5211 |
| ENSP00000262061 | COPZ1 | 0.5211 |
| ENSP00000325002 | COPG1 | 0.5211 |
| ENSP00000380602 | CNTN4 | 0.5211 |
| ENSP00000322887 | CIRBP | 0.5211 |
| ENSP00000256509 | CHL1 | 0.5211 |
| ENSP00000392395 | CHFR | 0.5211 |
| ENSP00000382863 | CHD8 | 0.5211 |
| ENSP00000369716 | CHD3 | 0.5211 |
| ENSP00000250838 | CDY2A | 0.5211 |
| ENSP00000328228 | CDCA2 | 0.5211 |
| ENSP00000280200 | CD226 | 0.5211 |
| ENSP00000280326 | CCT5 | 0.5211 |
| ENSP00000358327 | CASP7 | 0.5211 |
| ENSP00000264645 | CASC3 | 0.5211 |
| ENSP00000458149 | CAMP | 0.5211 |
| ENSP00000009105 | CAMK1G | 0.5211 |
| ENSP00000285379 | CA2 | 0.5211 |
| ENSP00000302397 | ATP1A3 | 0.5211 |
| ENSP00000322628 | ARL4D | 0.5211 |
| ENSP00000263674 | ARHGEF17 | 0.5211 |
| ENSP00000336666 | AP1S1 | 0.5211 |
| ENSP00000362010 | ANXA7 | 0.5211 |
| ENSP00000265748 | ANLN | 0.5211 |
| ENSP00000322175 | AK4 | 0.5211 |
| ENSP00000287295 | AIFM1 | 0.5211 |
| ENSP00000311405 | ADCY6 | 0.5211 |
| ENSP00000286657 | ADAMTS3 | 0.5211 |
| ENSP00000256001 | ACTR3B | 0.5211 |
| ENSP00000346733 | ACAP3 | 0.5211 |
| ENSP00000263817 | ABCB11 | 0.5211 |
| ENSP00000343488 | EARS2 | 0.5211 |
| ENSP00000355317 | WDTC1 | 0.5184 |
| ENSP00000261405 | VWF | 0.5184 |
| ENSP00000261517 | VPS13C | 0.5184 |
| ENSP00000381282 | VIMP | 0.5184 |
| ENSP00000294728 | VCAM1 | 0.5184 |
| ENSP00000310800 | VANGL1 | 0.5184 |

| | | |
|---|---|---|
| ENSP00000324560 | ULK1 | 0.5184 |
| ENSP00000379217 | UBFD1 | 0.5184 |
| ENSP00000347836 | UBE2H | 0.5184 |
| ENSP00000338413 | UBA1 | 0.5184 |
| ENSP00000308000 | TUT1 | 0.5184 |
| ENSP00000451560 | TUBB3 | 0.5184 |
| ENSP00000363095 | TRIM32 | 0.5184 |
| ENSP00000341957 | TP53BP2 | 0.5184 |
| ENSP00000353735 | TOPORS | 0.5184 |
| ENSP00000361636 | TNNC2 | 0.5184 |
| ENSP00000442046 | TMTC1 | 0.5184 |
| ENSP00000433967 | TM9SF1 | 0.5184 |
| ENSP00000215570 | TIMM13 | 0.5184 |
| ENSP00000355330 | TGM2 | 0.5184 |
| ENSP00000454021 | TGIF2-C20orf24 | 0.5184 |
| ENSP00000240335 | TBX4 | 0.5184 |
| ENSP00000328879 | TBL1Y | 0.5184 |
| ENSP00000362399 | STXBP1 | 0.5184 |
| ENSP00000350071 | STRN3 | 0.5184 |
| ENSP00000358810 | STRIP1 | 0.5184 |
| ENSP00000320754 | STK33 | 0.5184 |
| ENSP00000381030 | STK32A | 0.5184 |
| ENSP00000381968 | SPTLC3 | 0.5184 |
| ENSP00000359567 | SOX3 | 0.5184 |
| ENSP00000284776 | SORBS2 | 0.5184 |
| ENSP00000300413 | SNRPD1 | 0.5184 |
| ENSP00000281456 | SLC25A4 | 0.5184 |
| ENSP00000378364 | SLC11A2 | 0.5184 |
| ENSP00000353157 | SEPT2 | 0.5184 |
| ENSP00000359380 | SCD | 0.5184 |
| ENSP00000357799 | S100A10 | 0.5184 |
| ENSP00000355533 | RYR2 | 0.5184 |
| ENSP00000043402 | RTN4R | 0.5184 |
| ENSP00000349131 | RSPO3 | 0.5184 |
| ENSP00000288666 | RPS4Y2 | 0.5184 |
| ENSP00000362744 | RPS4X | 0.5184 |
| ENSP00000418082 | RPL37A | 0.5184 |
| ENSP00000362131 | RPA4 | 0.5184 |
| ENSP00000462196 | RP11-403P17.5 | 0.5184 |
| ENSP00000366093 | RORB | 0.5184 |
| ENSP00000351697 | REV3L | 0.5184 |
| ENSP00000262031 | RBMS2 | 0.5184 |
| ENSP00000348577 | RANGAP1 | 0.5184 |
| ENSP00000019317 | RALBP1 | 0.5184 |
| ENSP00000351832 | RAB3GAP2 | 0.5184 |
| ENSP00000411418 | RAB3GAP1 | 0.5184 |
| ENSP00000402060 | PVR | 0.5184 |
| ENSP00000354612 | PTGS1 | 0.5184 |
| ENSP00000216802 | PSME2 | 0.5184 |
| ENSP00000310572 | PSMC5 | 0.5184 |
| ENSP00000311121 | PSMA8 | 0.5184 |
| ENSP00000311290 | PROP1 | 0.5184 |
| ENSP00000351885 | PPP2R4 | 0.5184 |

| | | |
|---|---|---|
| ENSP00000311344 | PPP2R1B | 0.5184 |
| ENSP00000335084 | PPP1CC | 0.5184 |
| ENSP00000264714 | PPM1G | 0.5184 |
| ENSP00000300026 | PPIB | 0.5184 |
| ENSP00000419425 | PPIA | 0.5184 |
| ENSP00000312649 | PPARGC1B | 0.5184 |
| ENSP00000402065 | POLR2J2 | 0.5184 |
| ENSP00000264233 | POLQ | 0.5184 |
| ENSP00000300055 | PLIN1 | 0.5184 |
| ENSP00000279230 | PLCB3 | 0.5184 |
| ENSP00000359552 | PKN2 | 0.5184 |
| ENSP00000266497 | PIK3C2G | 0.5184 |
| ENSP00000341805 | PHF10 | 0.5184 |
| ENSP00000334910 | PDE2A | 0.5184 |
| ENSP00000381499 | PAX1 | 0.5184 |
| ENSP00000351049 | PAK4 | 0.5184 |
| ENSP00000219097 | ORC6 | 0.5184 |
| ENSP00000229179 | NUP107 | 0.5184 |
| ENSP00000339503 | NUDT1 | 0.5184 |
| ENSP00000281081 | NUBPL | 0.5184 |
| ENSP00000309126 | NSUN5 | 0.5184 |
| ENSP00000317992 | NOC2L | 0.5184 |
| ENSP00000356785 | NME7 | 0.5184 |
| ENSP00000386394 | NIF3L1 | 0.5184 |
| ENSP00000324792 | NFATC2IP | 0.5184 |
| ENSP00000266544 | NDUFA9 | 0.5184 |
| ENSP00000428657 | NAIP | 0.5184 |
| ENSP00000417763 | NAA10 | 0.5184 |
| ENSP00000418734 | MYSM1 | 0.5184 |
| ENSP00000355536 | MTR | 0.5184 |
| ENSP00000377617 | MTHFD2 | 0.5184 |
| ENSP00000320567 | MRPS33 | 0.5184 |
| ENSP00000356001 | MRPL18 | 0.5184 |
| ENSP00000343463 | MAP3K2 | 0.5184 |
| ENSP00000451511 | LY75 | 0.5184 |
| ENSP00000312652 | LEP | 0.5184 |
| ENSP00000314311 | LARP7 | 0.5184 |
| ENSP00000374309 | LAMA1 | 0.5184 |
| ENSP00000295225 | KCNIP3 | 0.5184 |
| ENSP00000267082 | ITGB7 | 0.5184 |
| ENSP00000310623 | ISCU | 0.5184 |
| ENSP00000360730 | IL13RA1 | 0.5184 |
| ENSP00000348784 | IGBP1 | 0.5184 |
| ENSP00000367623 | HSPA14 | 0.5184 |
| ENSP00000351108 | HNRNPAB | 0.5184 |
| ENSP00000408146 | HLA-DPB1 | 0.5184 |
| ENSP00000291823 | HIPK4 | 0.5184 |
| ENSP00000382269 | HECTD1 | 0.5184 |
| ENSP00000354581 | GPX6 | 0.5184 |
| ENSP00000366267 | GPC5 | 0.5184 |
| ENSP00000461460 | GOSR2 | 0.5184 |
| ENSP00000248564 | GNG11 | 0.5184 |
| ENSP00000331313 | GNB1L | 0.5184 |

| | | |
|---|---|---|
| ENSP00000306920 | GLB1 | 0.5184 |
| ENSP00000354347 | GFPT1 | 0.5184 |
| ENSP00000220429 | GABPB1 | 0.5184 |
| ENSP00000366814 | FOXD4L2 | 0.5184 |
| ENSP00000292180 | FLAD1 | 0.5184 |
| ENSP00000326022 | FHL5 | 0.5184 |
| ENSP00000306099 | FGB | 0.5184 |
| ENSP00000401445 | ERN1 | 0.5184 |
| ENSP00000233057 | EIF2AK2 | 0.5184 |
| ENSP00000267803 | DUOXA1 | 0.5184 |
| ENSP00000322885 | DTX2 | 0.5184 |
| ENSP00000265104 | DNAH5 | 0.5184 |
| ENSP00000371711 | DMRT1 | 0.5184 |
| ENSP00000353132 | DMBX1 | 0.5184 |
| ENSP00000389870 | DLX3 | 0.5184 |
| ENSP00000350616 | DDC | 0.5184 |
| ENSP00000301764 | DDB1 | 0.5184 |
| ENSP00000376808 | CUL5 | 0.5184 |
| ENSP00000355050 | CTNNBL1 | 0.5184 |
| ENSP00000273179 | CTDSPL | 0.5184 |
| ENSP00000273857 | CORIN | 0.5184 |
| ENSP00000366275 | CNNM4 | 0.5184 |
| ENSP00000266546 | CLSTN3 | 0.5184 |
| ENSP00000418287 | CIAO1 | 0.5184 |
| ENSP00000301280 | CHAF1A | 0.5184 |
| ENSP00000364737 | CENPP | 0.5184 |
| ENSP00000303178 | CDY1B | 0.5184 |
| ENSP00000271324 | CD53 | 0.5184 |
| ENSP00000295688 | CCT3 | 0.5184 |
| ENSP00000295055 | CAPN13 | 0.5184 |
| ENSP00000263119 | CABIN1 | 0.5184 |
| ENSP00000358474 | BRCC3 | 0.5184 |
| ENSP00000269980 | BCKDHA | 0.5184 |
| ENSP00000443459 | BCAT1 | 0.5184 |
| ENSP00000310275 | BANF1 | 0.5184 |
| ENSP00000238789 | ATAD2B | 0.5184 |
| ENSP00000371517 | AMACR | 0.5184 |
| ENSP00000375881 | ALPP | 0.5184 |
| ENSP00000249750 | ALDH1A2 | 0.5184 |
| ENSP00000356825 | ADCY10 | 0.5184 |
| ENSP00000309968 | ADAM17 | 0.5184 |
| ENSP00000397552 | ACTL6A | 0.5184 |
| ENSP00000218758 | ACP5 | 0.5184 |
| ENSP00000260645 | ABCG5 | 0.5184 |
| ENSP00000432545 | NANOGP1 | 0.5184 |
| ENSP00000262383 | ZNF423 | 0.5158 |
| ENSP00000360017 | ZNF280C | 0.5158 |
| ENSP00000288828 | WIPI2 | 0.5158 |
| ENSP00000311127 | VEGFB | 0.5158 |
| ENSP00000263864 | VAMP8 | 0.5158 |
| ENSP00000262803 | UPF1 | 0.5158 |
| ENSP00000302640 | UBTF | 0.5158 |
| ENSP00000376215 | UBE2E3 | 0.5158 |

| | | |
|---|---|---|
| ENSP00000383365 | TXNRD2 | 0.5158 |
| ENSP00000392549 | TTLL3 | 0.5158 |
| ENSP00000406407 | TSPY10 | 0.5158 |
| ENSP00000339299 | TRIO | 0.5158 |
| ENSP00000460823 | TOM1L1 | 0.5158 |
| ENSP00000360483 | TMEM48 | 0.5158 |
| ENSP00000389414 | TBXAS1 | 0.5158 |
| ENSP00000362068 | TAF8 | 0.5158 |
| ENSP00000418379 | TAF1L | 0.5158 |
| ENSP00000308022 | TADA2B | 0.5158 |
| ENSP00000377262 | SRPK2 | 0.5158 |
| ENSP00000337513 | SRA1 | 0.5158 |
| ENSP00000330221 | SPSB1 | 0.5158 |
| ENSP00000355217 | SNX6 | 0.5158 |
| ENSP00000374152 | SMYD5 | 0.5158 |
| ENSP00000370990 | SHOX | 0.5158 |
| ENSP00000418744 | SH3GLB1 | 0.5158 |
| ENSP00000268220 | SEC11A | 0.5158 |
| ENSP00000225430 | RPL19 | 0.5158 |
| ENSP00000354739 | RPL12 | 0.5158 |
| ENSP00000341730 | RPL10 | 0.5158 |
| ENSP00000414068 | RP4-559A3.7 | 0.5158 |
| ENSP00000461295 | RP11-343C2.3 | 0.5158 |
| ENSP00000290524 | RFX5 | 0.5158 |
| ENSP00000317636 | RECQL5 | 0.5158 |
| ENSP00000359645 | RBMX | 0.5158 |
| ENSP00000333001 | RBM8A | 0.5158 |
| ENSP00000264431 | RAPGEF2 | 0.5158 |
| ENSP00000394415 | PTGES3L | 0.5158 |
| ENSP00000343966 | PSPC1 | 0.5158 |
| ENSP00000290541 | PSMB4 | 0.5158 |
| ENSP00000355747 | PSEN2 | 0.5158 |
| ENSP00000234071 | PROC | 0.5158 |
| ENSP00000305355 | PRKCB | 0.5158 |
| ENSP00000233944 | PRKAG3 | 0.5158 |
| ENSP00000331065 | PPP1R27 | 0.5158 |
| ENSP00000200453 | PPP1R15A | 0.5158 |
| ENSP00000253329 | PPIL4 | 0.5158 |
| ENSP00000215587 | POLR2E | 0.5158 |
| ENSP00000323856 | PLEC | 0.5158 |
| ENSP00000348888 | PIGR | 0.5158 |
| ENSP00000384197 | PDZRN4 | 0.5158 |
| ENSP00000300289 | PDIA3 | 0.5158 |
| ENSP00000369134 | PDHA1 | 0.5158 |
| ENSP00000422464 | PDGFC | 0.5158 |
| ENSP00000285083 | OXNAD1 | 0.5158 |
| ENSP00000285420 | OTUD6B | 0.5158 |
| ENSP00000284719 | OLA1 | 0.5158 |
| ENSP00000294172 | NXF1 | 0.5158 |
| ENSP00000419628 | NUDT5 | 0.5158 |
| ENSP00000262302 | NUBP2 | 0.5158 |
| ENSP00000223190 | NRF1 | 0.5158 |
| ENSP00000242152 | NPY | 0.5158 |

| | | |
|---|---|---|
| ENSP00000359024 | NOLC1 | 0.5158 |
| ENSP00000419457 | NOBOX | 0.5158 |
| ENSP00000265191 | NME5 | 0.5158 |
| ENSP00000388910 | NFATC4 | 0.5158 |
| ENSP00000251588 | NARFL | 0.5158 |
| ENSP00000407952 | NAMPTL | 0.5158 |
| ENSP00000307280 | MYL1 | 0.5158 |
| ENSP00000220058 | MTFMT | 0.5158 |
| ENSP00000278823 | MTA2 | 0.5158 |
| ENSP00000225969 | MRPL27 | 0.5158 |
| ENSP00000297508 | MICALL2 | 0.5158 |
| ENSP00000235329 | MFN2 | 0.5158 |
| ENSP00000386951 | MED26 | 0.5158 |
| ENSP00000000412 | M6PR | 0.5158 |
| ENSP00000303634 | LRP8 | 0.5158 |
| ENSP00000273261 | LRIG1 | 0.5158 |
| ENSP00000321537 | LLGL1 | 0.5158 |
| ENSP00000367787 | LIG3 | 0.5158 |
| ENSP00000377854 | LHX6 | 0.5158 |
| ENSP00000329243 | KRT7 | 0.5158 |
| ENSP00000330878 | KPNA7 | 0.5158 |
| ENSP00000334373 | KPNA4 | 0.5158 |
| ENSP00000259335 | KIAA0368 | 0.5158 |
| ENSP00000370744 | ITPR2 | 0.5158 |
| ENSP00000268296 | ITGAX | 0.5158 |
| ENSP00000331103 | IP6K2 | 0.5158 |
| ENSP00000243786 | INHA | 0.5158 |
| ENSP00000320566 | ING3 | 0.5158 |
| ENSP00000244174 | IL9R | 0.5158 |
| ENSP00000256452 | IL5RA | 0.5158 |
| ENSP00000363779 | IKBKAP | 0.5158 |
| ENSP00000290341 | IGF2BP1 | 0.5158 |
| ENSP00000343957 | IFNAR2 | 0.5158 |
| ENSP00000276927 | IFNA1 | 0.5158 |
| ENSP00000298556 | HPRT1 | 0.5158 |
| ENSP00000359680 | HOGA1 | 0.5158 |
| ENSP00000264350 | HERC5 | 0.5158 |
| ENSP00000254854 | GUCY2D | 0.5158 |
| ENSP00000306138 | GRM5 | 0.5158 |
| ENSP00000392398 | GPX5 | 0.5158 |
| ENSP00000359539 | GOT1 | 0.5158 |
| ENSP00000472249 | GMFG | 0.5158 |
| ENSP00000322716 | GLYR1 | 0.5158 |
| ENSP00000362463 | GLO1 | 0.5158 |
| ENSP00000418001 | GK5 | 0.5158 |
| ENSP00000248923 | GGT1 | 0.5158 |
| ENSP00000311962 | GGA2 | 0.5158 |
| ENSP00000253408 | GFAP | 0.5158 |
| ENSP00000318977 | GEN1 | 0.5158 |
| ENSP00000366630 | FOXD4L4 | 0.5158 |
| ENSP00000369335 | FBXO18 | 0.5158 |
| ENSP00000347834 | FBXL3 | 0.5158 |
| ENSP00000364731 | F7 | 0.5158 |

| | | |
|---|---|---|
| ENSP00000262455 | ERP44 | 0.5158 |
| ENSP00000237853 | ELL2 | 0.5158 |
| ENSP00000349594 | ELAVL4 | 0.5158 |
| ENSP00000354722 | EIF1AY | 0.5158 |
| ENSP00000357392 | EFNA1 | 0.5158 |
| ENSP00000331901 | EEF1G | 0.5158 |
| ENSP00000240343 | DYNLL2 | 0.5158 |
| ENSP00000262177 | DNAJB6 | 0.5158 |
| ENSP00000404381 | DNAJB14 | 0.5158 |
| ENSP00000330671 | DNAH11 | 0.5158 |
| ENSP00000363480 | DLG3 | 0.5158 |
| ENSP00000216500 | DHRS7 | 0.5158 |
| ENSP00000362217 | DACH2 | 0.5158 |
| ENSP00000408236 | CYTH2 | 0.5158 |
| ENSP00000354468 | CYB5R3 | 0.5158 |
| ENSP00000341625 | CYB5A | 0.5158 |
| ENSP00000220166 | CTSH | 0.5158 |
| ENSP00000466514 | CTB-54O9.9 | 0.5158 |
| ENSP00000291568 | CSTB | 0.5158 |
| ENSP00000308236 | COMMD1 | 0.5158 |
| ENSP00000360882 | COL5A1 | 0.5158 |
| ENSP00000362776 | COL16A1 | 0.5158 |
| ENSP00000264638 | CNTNAP1 | 0.5158 |
| ENSP00000064724 | CLDN11 | 0.5158 |
| ENSP00000300283 | CKMT1B | 0.5158 |
| ENSP00000262315 | CHTF18 | 0.5158 |
| ENSP00000341159 | CERKL | 0.5158 |
| ENSP00000265071 | CDH6 | 0.5158 |
| ENSP00000356405 | CDC73 | 0.5158 |
| ENSP00000353362 | CACNA1A | 0.5158 |
| ENSP00000245907 | C3 | 0.5158 |
| ENSP00000348107 | C1D | 0.5158 |
| ENSP00000378181 | BRD7 | 0.5158 |
| ENSP00000372210 | BET1L | 0.5158 |
| ENSP00000222547 | BET1 | 0.5158 |
| ENSP00000364883 | AUH | 0.5158 |
| ENSP00000306003 | ATP5I | 0.5158 |
| ENSP00000370414 | ATP5EP2 | 0.5158 |
| ENSP00000466174 | ATF7 | 0.5158 |
| ENSP00000262215 | ARFGEF1 | 0.5158 |
| ENSP00000264028 | ARCN1 | 0.5158 |
| ENSP00000233242 | APOB | 0.5158 |
| ENSP00000263574 | APLP2 | 0.5158 |
| ENSP00000174653 | AP3M2 | 0.5158 |
| ENSP00000377170 | ANKRD39 | 0.5158 |
| ENSP00000332256 | ALDH1A3 | 0.5158 |
| ENSP00000355465 | AHCTF1 | 0.5158 |
| ENSP00000310978 | AGXT2L2 | 0.5158 |
| ENSP00000368766 | ADRA1D | 0.5158 |
| ENSP00000355739 | ADCK3 | 0.5158 |
| ENSP00000251582 | ADAMTS2 | 0.5158 |
| ENSP00000414558 | ABHD4 | 0.5158 |
| ENSP00000359233 | ABCD3 | 0.5158 |

| | | |
|---|---|---|
| ENSP00000225402 | AATF | 0.5158 |
| ENSP00000353586 | ZNF280B | 0.5132 |
| ENSP00000440847 | ZNF185 | 0.5132 |
| ENSP00000322845 | ZMYM3 | 0.5132 |
| ENSP00000251038 | ZC3H14 | 0.5132 |
| ENSP00000329614 | YBEY | 0.5132 |
| ENSP00000313059 | WNK1 | 0.5132 |
| ENSP00000267199 | VPS33A | 0.5132 |
| ENSP00000258123 | USP15 | 0.5132 |
| ENSP00000338703 | UGP2 | 0.5132 |
| ENSP00000428209 | UBE2V2 | 0.5132 |
| ENSP00000373570 | TYRP1 | 0.5132 |
| ENSP00000290846 | TRMU | 0.5132 |
| ENSP00000456272 | TRIM59 | 0.5132 |
| ENSP00000367030 | TRDMT1 | 0.5132 |
| ENSP00000450635 | TMEM189 | 0.5132 |
| ENSP00000233638 | TLX2 | 0.5132 |
| ENSP00000295899 | THOC7 | 0.5132 |
| ENSP00000354588 | TEAD1 | 0.5132 |
| ENSP00000395574 | TCEB3 | 0.5132 |
| ENSP00000312624 | TCAP | 0.5132 |
| ENSP00000362590 | TBC1D22B | 0.5132 |
| ENSP00000448182 | TBC1D15 | 0.5132 |
| ENSP00000346206 | TAP1 | 0.5132 |
| ENSP00000258281 | TAF5L | 0.5132 |
| ENSP00000356224 | SYNE1 | 0.5132 |
| ENSP00000295770 | STT3B | 0.5132 |
| ENSP00000310969 | STON1 | 0.5132 |
| ENSP00000368952 | STOML3 | 0.5132 |
| ENSP00000349259 | SPTBN1 | 0.5132 |
| ENSP00000378517 | SPP1 | 0.5132 |
| ENSP00000311684 | SPEG | 0.5132 |
| ENSP00000256637 | SORT1 | 0.5132 |
| ENSP00000371554 | SOD3 | 0.5132 |
| ENSP00000370119 | SMN2 | 0.5132 |
| ENSP00000323967 | SMARCE1 | 0.5132 |
| ENSP00000450909 | SLIRP | 0.5132 |
| ENSP00000230671 | SLC6A7 | 0.5132 |
| ENSP00000261707 | SLC6A4 | 0.5132 |
| ENSP00000355893 | SLC30A10 | 0.5132 |
| ENSP00000383212 | SGSM1 | 0.5132 |
| ENSP00000350447 | SGOL2 | 0.5132 |
| ENSP00000372221 | SERPINB5 | 0.5132 |
| ENSP00000341524 | SEPT6 | 0.5132 |
| ENSP00000368572 | SAT1 | 0.5132 |
| ENSP00000337838 | RTN4 | 0.5132 |
| ENSP00000355315 | RPL39 | 0.5132 |
| ENSP00000346001 | RPL3 | 0.5132 |
| ENSP00000375730 | RPL13A | 0.5132 |
| ENSP00000304670 | RNFT1 | 0.5132 |
| ENSP00000324956 | RNF14 | 0.5132 |
| ENSP00000373277 | RBMS3 | 0.5132 |
| ENSP00000358042 | QRSL1 | 0.5132 |

| | | |
|---|---|---|
| ENSP00000216392 | PYGL | 0.5132 |
| ENSP00000344909 | PTP4A2 | 0.5132 |
| ENSP00000379213 | PTHLH | 0.5132 |
| ENSP00000244043 | PTGIS | 0.5132 |
| ENSP00000349003 | PTGER3 | 0.5132 |
| ENSP00000217958 | PSMD10 | 0.5132 |
| ENSP00000401802 | PSMC6 | 0.5132 |
| ENSP00000365494 | PRICKLE3 | 0.5132 |
| ENSP00000296122 | PPP1CB | 0.5132 |
| ENSP00000286175 | PPIL3 | 0.5132 |
| ENSP00000354871 | PPEF1 | 0.5132 |
| ENSP00000369071 | POSTN | 0.5132 |
| ENSP00000331678 | PKP3 | 0.5132 |
| ENSP00000357883 | PIP5K1A | 0.5132 |
| ENSP00000262719 | PHLPP1 | 0.5132 |
| ENSP00000305995 | PGK2 | 0.5132 |
| ENSP00000417587 | PEG10 | 0.5132 |
| ENSP00000358033 | PDSS2 | 0.5132 |
| ENSP00000288050 | PDPR | 0.5132 |
| ENSP00000449770 | PA2G4 | 0.5132 |
| ENSP00000257570 | OASL | 0.5132 |
| ENSP00000230792 | NUDT12 | 0.5132 |
| ENSP00000310205 | NRIP3 | 0.5132 |
| ENSP00000346196 | NDUFV3 | 0.5132 |
| ENSP00000276689 | NDUFB9 | 0.5132 |
| ENSP00000261597 | NDC80 | 0.5132 |
| ENSP00000354152 | MTHFSD | 0.5132 |
| ENSP00000354632 | MT-ATP6 | 0.5132 |
| ENSP00000261507 | MSMO1 | 0.5132 |
| ENSP00000391664 | MFRP | 0.5132 |
| ENSP00000325312 | METAP2 | 0.5132 |
| ENSP00000215555 | MARCH2 | 0.5132 |
| ENSP00000300249 | MAPRE2 | 0.5132 |
| ENSP00000304604 | MAGI3 | 0.5132 |
| ENSP00000374135 | LRP1B | 0.5132 |
| ENSP00000261596 | LPIN2 | 0.5132 |
| ENSP00000340688 | LPHN1 | 0.5132 |
| ENSP00000377900 | LIPF | 0.5132 |
| ENSP00000341947 | LIN54 | 0.5132 |
| ENSP00000261731 | LHX5 | 0.5132 |
| ENSP00000300051 | LDHD | 0.5132 |
| ENSP00000302393 | LDHAL6B | 0.5132 |
| ENSP00000375810 | KLC3 | 0.5132 |
| ENSP00000341466 | KIF18B | 0.5132 |
| ENSP00000258739 | KDELR2 | 0.5132 |
| ENSP00000345708 | KCNJ11 | 0.5132 |
| ENSP00000262644 | IMPAD1 | 0.5132 |
| ENSP00000321345 | IL23R | 0.5132 |
| ENSP00000233946 | IL1R1 | 0.5132 |
| ENSP00000280357 | IL18 | 0.5132 |
| ENSP00000371634 | IGF2BP2 | 0.5132 |
| ENSP00000388223 | IFNAR2 | 0.5132 |
| ENSP00000287936 | HMGCR | 0.5132 |

| | | |
|---|---|---|
| ENSP00000364310 | H2AFX | 0.5132 |
| ENSP00000311469 | GSTM1 | 0.5132 |
| ENSP00000338573 | GNL3L | 0.5132 |
| ENSP00000307900 | GLUL | 0.5132 |
| ENSP00000241124 | GJB6 | 0.5132 |
| ENSP00000262460 | GINS1 | 0.5132 |
| ENSP00000307939 | GCC2 | 0.5132 |
| ENSP00000366525 | FTL | 0.5132 |
| ENSP00000162391 | FOXJ2 | 0.5132 |
| ENSP00000366693 | FOXD4L6 | 0.5132 |
| ENSP00000071281 | FHL1 | 0.5132 |
| ENSP00000364277 | FGD1 | 0.5132 |
| ENSP00000243167 | FAAH | 0.5132 |
| ENSP00000419945 | ERVW-1 | 0.5132 |
| ENSP00000341138 | EPB41L3 | 0.5132 |
| ENSP00000261868 | EIF3J | 0.5132 |
| ENSP00000221418 | ECH1 | 0.5132 |
| ENSP00000361667 | DOLK | 0.5132 |
| ENSP00000441858 | DMXL2 | 0.5132 |
| ENSP00000247191 | DLGAP5 | 0.5132 |
| ENSP00000301180 | DIP2B | 0.5132 |
| ENSP00000264093 | DGUOK | 0.5132 |
| ENSP00000260184 | DDX60L | 0.5132 |
| ENSP00000368667 | DDX53 | 0.5132 |
| ENSP00000401363 | DAZ4 | 0.5132 |
| ENSP00000247170 | DAAM1 | 0.5132 |
| ENSP00000370460 | CWC27 | 0.5132 |
| ENSP00000381148 | CTDSP2 | 0.5132 |
| ENSP00000332444 | CSTF2T | 0.5132 |
| ENSP00000280527 | CRIM1 | 0.5132 |
| ENSP00000181383 | CPB2 | 0.5132 |
| ENSP00000261643 | COX10 | 0.5132 |
| ENSP00000309338 | CLCF1 | 0.5132 |
| ENSP00000364976 | CKS2 | 0.5132 |
| ENSP00000306490 | CHRM1 | 0.5132 |
| ENSP00000355511 | CHML | 0.5132 |
| ENSP00000283882 | CFDP1 | 0.5132 |
| ENSP00000381768 | CDH23 | 0.5132 |
| ENSP00000370781 | CDC20B | 0.5132 |
| ENSP00000259633 | CD72 | 0.5132 |
| ENSP00000296140 | CCR1 | 0.5132 |
| ENSP00000374529 | CCNK | 0.5132 |
| ENSP00000295006 | CAPN2 | 0.5132 |
| ENSP00000312741 | CAMKK2 | 0.5132 |
| ENSP00000256460 | CAMK1 | 0.5132 |
| ENSP00000269127 | C17orf64 | 0.5132 |
| ENSP00000376914 | ATP2C1 | 0.5132 |
| ENSP00000349325 | ATP10A | 0.5132 |
| ENSP00000369217 | ARIH1 | 0.5132 |
| ENSP00000318775 | ANAPC4 | 0.5132 |
| ENSP00000430075 | AMPD1 | 0.5132 |
| ENSP00000226253 | ALDOC | 0.5132 |
| ENSP00000267584 | AK7 | 0.5132 |

| | | |
|---|---|---|
| ENSP00000349525 | AHSA2 | 0.5132 |
| ENSP00000269143 | AFG3L2 | 0.5132 |
| ENSP00000381840 | AEBP2 | 0.5132 |
| ENSP00000286621 | ADK | 0.5132 |
| ENSP00000320709 | ADIPOQ | 0.5132 |
| ENSP00000336842 | ACTR8 | 0.5132 |
| ENSP00000331514 | ACTG1 | 0.5132 |
| ENSP00000217455 | ACOT8 | 0.5132 |
| ENSP00000265838 | ACAT1 | 0.5132 |
| ENSP00000264990 | ACAD11 | 0.5132 |
| ENSP00000296577 | ABCE1 | 0.5132 |
| ENSP00000377040 | AASS | 0.5132 |
| ENSP00000361280 | MAT1A | 0.5132 |
| ENSP00000360817 | ZNFX1 | 0.5105 |
| ENSP00000351137 | XAB2 | 0.5105 |
| ENSP00000350854 | VPS13D | 0.5105 |
| ENSP00000359869 | USP26 | 0.5105 |
| ENSP00000401197 | USP19 | 0.5105 |
| ENSP00000203407 | UQCRC1 | 0.5105 |
| ENSP00000364403 | UBR4 | 0.5105 |
| ENSP00000222402 | UBE2D4 | 0.5105 |
| ENSP00000246548 | UBA2 | 0.5105 |
| ENSP00000360329 | TTC4 | 0.5105 |
| ENSP00000233623 | TTC31 | 0.5105 |
| ENSP00000403304 | TSPY1 | 0.5105 |
| ENSP00000340913 | TRPC6 | 0.5105 |
| ENSP00000251607 | TRNT1 | 0.5105 |
| ENSP00000252136 | TRMT2A | 0.5105 |
| ENSP00000381240 | TRDN | 0.5105 |
| ENSP00000262709 | TOX4 | 0.5105 |
| ENSP00000405926 | TMED7 | 0.5105 |
| ENSP00000218032 | TLR8 | 0.5105 |
| ENSP00000355119 | TGIF2LX | 0.5105 |
| ENSP00000276603 | TERF1 | 0.5105 |
| ENSP00000370607 | TECRL | 0.5105 |
| ENSP00000215567 | TECR | 0.5105 |
| ENSP00000234827 | TCEANC2 | 0.5105 |
| ENSP00000393333 | TBXA2R | 0.5105 |
| ENSP00000356795 | TBX19 | 0.5105 |
| ENSP00000276072 | TAF1 | 0.5105 |
| ENSP00000263918 | STRN | 0.5105 |
| ENSP00000348278 | STK39 | 0.5105 |
| ENSP00000379964 | STK16 | 0.5105 |
| ENSP00000262554 | SPTLC1 | 0.5105 |
| ENSP00000268704 | SPG7 | 0.5105 |
| ENSP00000309710 | SLC39A1 | 0.5105 |
| ENSP00000075120 | SLC2A3 | 0.5105 |
| ENSP00000360671 | SLC25A5 | 0.5105 |
| ENSP00000221485 | SLC17A7 | 0.5105 |
| ENSP00000316842 | SIX5 | 0.5105 |
| ENSP00000459962 | SHPK | 0.5105 |
| ENSP00000358464 | SHOC2 | 0.5105 |
| ENSP00000369816 | SHBG | 0.5105 |

| | | |
|---|---|---|
| ENSP00000309714 | SH3PXD2B | 0.5105 |
| ENSP00000332513 | SH3BGR | 0.5105 |
| ENSP00000338343 | SGCD | 0.5105 |
| ENSP00000390783 | SCFD1 | 0.5105 |
| ENSP00000264027 | SC5D | 0.5105 |
| ENSP00000303248 | RXFP1 | 0.5105 |
| ENSP00000302896 | RPS9 | 0.5105 |
| ENSP00000346027 | RPL21 | 0.5105 |
| ENSP00000359688 | RPF1 | 0.5105 |
| ENSP00000385916 | RP11-145E5.5 | 0.5105 |
| ENSP00000362937 | RCC1 | 0.5105 |
| ENSP00000340176 | RBPMS | 0.5105 |
| ENSP00000316543 | RAPH1 | 0.5105 |
| ENSP00000466399 | RAD51D | 0.5105 |
| ENSP00000347839 | RAB11FIP2 | 0.5105 |
| ENSP00000304336 | PYDC1 | 0.5105 |
| ENSP00000384211 | PSME4 | 0.5105 |
| ENSP00000357879 | PSMD4 | 0.5105 |
| ENSP00000355325 | PSMB5 | 0.5105 |
| ENSP00000266079 | PRPF6 | 0.5105 |
| ENSP00000333568 | PRKD1 | 0.5105 |
| ENSP00000252115 | POLDIP3 | 0.5105 |
| ENSP00000219345 | PLA2G15 | 0.5105 |
| ENSP00000215904 | PDXP | 0.5105 |
| ENSP00000355245 | PAX9 | 0.5105 |
| ENSP00000448059 | PAH | 0.5105 |
| ENSP00000234111 | ODC1 | 0.5105 |
| ENSP00000310668 | NUP93 | 0.5105 |
| ENSP00000245544 | NUP85 | 0.5105 |
| ENSP00000353582 | NRP2 | 0.5105 |
| ENSP00000258317 | NPL | 0.5105 |
| ENSP00000368190 | NPHS1 | 0.5105 |
| ENSP00000337383 | NLRP3 | 0.5105 |
| ENSP00000342828 | NKX2-3 | 0.5105 |
| ENSP00000232978 | NKTR | 0.5105 |
| ENSP00000298310 | NEMF | 0.5105 |
| ENSP00000362702 | NEK6 | 0.5105 |
| ENSP00000339720 | NDUFA4 | 0.5105 |
| ENSP00000348657 | NCAPG2 | 0.5105 |
| ENSP00000251496 | NCAPG | 0.5105 |
| ENSP00000378288 | MYLK3 | 0.5105 |
| ENSP00000355206 | MT-ND3 | 0.5105 |
| ENSP00000355046 | MT-ND2 | 0.5105 |
| ENSP00000354525 | MRPL24 | 0.5105 |
| ENSP00000248248 | MON1B | 0.5105 |
| ENSP00000256194 | MICAL2 | 0.5105 |
| ENSP00000243911 | MC3R | 0.5105 |
| ENSP00000353508 | MAP2 | 0.5105 |
| ENSP00000256720 | LPIN1 | 0.5105 |
| ENSP00000266674 | LGR5 | 0.5105 |
| ENSP00000276590 | LACTB2 | 0.5105 |
| ENSP00000273963 | KLHL8 | 0.5105 |
| ENSP00000268919 | KIF2B | 0.5105 |

| | | |
|---|---|---|
| ENSP00000324020 | KIF15 | 0.5105 |
| ENSP00000281156 | KHDRBS2 | 0.5105 |
| ENSP00000266719 | KERA | 0.5105 |
| ENSP00000361473 | KDM4A | 0.5105 |
| ENSP00000288309 | KCNJ6 | 0.5105 |
| ENSP00000256861 | ITIH5 | 0.5105 |
| ENSP00000312778 | IFT80 | 0.5105 |
| ENSP00000355672 | IBA57 | 0.5105 |
| ENSP00000261609 | HERC2 | 0.5105 |
| ENSP00000361245 | HECTD3 | 0.5105 |
| ENSP00000325290 | GSG2 | 0.5105 |
| ENSP00000329558 | GRPEL2 | 0.5105 |
| ENSP00000407375 | GPX1 | 0.5105 |
| ENSP00000355556 | GNG4 | 0.5105 |
| ENSP00000323901 | FZD2 | 0.5105 |
| ENSP00000351602 | FUT4 | 0.5105 |
| ENSP00000326948 | FTSJ1 | 0.5105 |
| ENSP00000216187 | FOXRED2 | 0.5105 |
| ENSP00000223136 | FIS1 | 0.5105 |
| ENSP00000413496 | FGF12 | 0.5105 |
| ENSP00000437464 | FBXL17 | 0.5105 |
| ENSP00000310842 | FANCI | 0.5105 |
| ENSP00000404658 | EZH1 | 0.5105 |
| ENSP00000334675 | ERCC6L | 0.5105 |
| ENSP00000356489 | EPM2A | 0.5105 |
| ENSP00000272167 | EPHX1 | 0.5105 |
| ENSP00000393324 | EIF4E3 | 0.5105 |
| ENSP00000307080 | EGLN2 | 0.5105 |
| ENSP00000355601 | EGLN1 | 0.5105 |
| ENSP00000381167 | DYNC2H1 | 0.5105 |
| ENSP00000369129 | DSP | 0.5105 |
| ENSP00000339850 | DPYSL4 | 0.5105 |
| ENSP00000254322 | DNAJB1 | 0.5105 |
| ENSP00000335304 | DLST | 0.5105 |
| ENSP00000332258 | DGAT1 | 0.5105 |
| ENSP00000308344 | DCAF7 | 0.5105 |
| ENSP00000376776 | DBH | 0.5105 |
| ENSP00000301141 | CYP2A6 | 0.5105 |
| ENSP00000224356 | CYP26A1 | 0.5105 |
| ENSP00000457656 | CYB5RL | 0.5105 |
| ENSP00000463376 | CTC-479C5.12 | 0.5105 |
| ENSP00000378736 | CRCP | 0.5105 |
| ENSP00000265394 | CPVL | 0.5105 |
| ENSP00000265641 | CPT1A | 0.5105 |
| ENSP00000229379 | COX6A1 | 0.5105 |
| ENSP00000355629 | COG2 | 0.5105 |
| ENSP00000355279 | CNOT7 | 0.5105 |
| ENSP00000358894 | CNNM2 | 0.5105 |
| ENSP00000327009 | CLIP4 | 0.5105 |
| ENSP00000254035 | CKMT2 | 0.5105 |
| ENSP00000322343 | CDK20 | 0.5105 |
| ENSP00000215574 | CDC34 | 0.5105 |
| ENSP00000246657 | CCR7 | 0.5105 |

| | | |
|---|---|---|
| ENSP00000315743 | CCNG2 | 0.5105 |
| ENSP00000367832 | CCL7 | 0.5105 |
| ENSP00000251973 | CARD10 | 0.5105 |
| ENSP00000367608 | CA9 | 0.5105 |
| ENSP00000415941 | C4B | 0.5105 |
| ENSP00000302079 | C3AR1 | 0.5105 |
| ENSP00000327336 | BGN | 0.5105 |
| ENSP00000362441 | ATRX | 0.5105 |
| ENSP00000285393 | ATP6V0D2 | 0.5105 |
| ENSP00000329757 | ATP6V0C | 0.5105 |
| ENSP00000283290 | ATG3 | 0.5105 |
| ENSP00000311538 | ARR3 | 0.5105 |
| ENSP00000266503 | ARNTL2 | 0.5105 |
| ENSP00000264183 | ARID4B | 0.5105 |
| ENSP00000386911 | ARHGAP25 | 0.5105 |
| ENSP00000300060 | ANPEP | 0.5105 |
| ENSP00000303518 | ANKRD49 | 0.5105 |
| ENSP00000349957 | ANAPC11 | 0.5105 |
| ENSP00000273588 | AMT | 0.5105 |
| ENSP00000252505 | ALLC | 0.5105 |
| ENSP00000314649 | ALDH5A1 | 0.5105 |
| ENSP00000337736 | AKAP1 | 0.5105 |
| ENSP00000216479 | AHSA1 | 0.5105 |
| ENSP00000264167 | AGPS | 0.5105 |
| ENSP00000294724 | AGL | 0.5105 |
| ENSP00000296412 | ADH5 | 0.5105 |
| ENSP00000209668 | ADH1A | 0.5105 |
| ENSP00000286355 | ADCY8 | 0.5105 |
| ENSP00000260600 | ADCY3 | 0.5105 |
| ENSP00000449535 | ACSS3 | 0.5105 |
| ENSP00000307697 | ACOX2 | 0.5105 |
| ENSP00000355637 | ABCB10 | 0.5105 |
| ENSP00000338190 | ABLIM1 | 0.5105 |
| ENSP00000338397 | NUDT2 | 0.5105 |
| ENSP00000311778 | ZMIZ2 | 0.5079 |
| ENSP00000364895 | ZBTB17 | 0.5079 |
| ENSP00000223369 | YKT6 | 0.5079 |
| ENSP00000301765 | VPS37C | 0.5079 |
| ENSP00000220509 | VPS18 | 0.5079 |
| ENSP00000442436 | URI1 | 0.5079 |
| ENSP00000466420 | UQCR11 | 0.5079 |
| ENSP00000355343 | UBAP2L | 0.5079 |
| ENSP00000248846 | TUBGCP6 | 0.5079 |
| ENSP00000368591 | TTLL10 | 0.5079 |
| ENSP00000300181 | TSC22D4 | 0.5079 |
| ENSP00000379065 | TRPS1 | 0.5079 |
| ENSP00000219746 | TOX3 | 0.5079 |
| ENSP00000336783 | TNRC6C | 0.5079 |
| ENSP00000330945 | TMED9 | 0.5079 |
| ENSP00000257245 | TIMM10 | 0.5079 |
| ENSP00000358089 | TIAL1 | 0.5079 |
| ENSP00000284240 | THY1 | 0.5079 |
| ENSP00000420616 | TFDP2 | 0.5079 |

| | | |
|---|---|---|
| ENSP00000444788 | TDO2 | 0.5079 |
| ENSP00000226444 | SULT1E1 | 0.5079 |
| ENSP00000075503 | STYK1 | 0.5079 |
| ENSP00000278742 | ST14 | 0.5079 |
| ENSP00000325905 | SRSF7 | 0.5079 |
| ENSP00000359119 | SRPK3 | 0.5079 |
| ENSP00000374372 | SPTB | 0.5079 |
| ENSP00000353238 | SPN | 0.5079 |
| ENSP00000320885 | SPAST | 0.5079 |
| ENSP00000282470 | SPARCL1 | 0.5079 |
| ENSP00000421380 | SNF8 | 0.5079 |
| ENSP00000349823 | SMARCAL1 | 0.5079 |
| ENSP00000330320 | SLC9A7 | 0.5079 |
| ENSP00000217420 | SLC32A1 | 0.5079 |
| ENSP00000264784 | SLC2A9 | 0.5079 |
| ENSP00000364543 | SKIV2L | 0.5079 |
| ENSP00000285116 | SKA1 | 0.5079 |
| ENSP00000356906 | SH2D1B | 0.5079 |
| ENSP00000321853 | SERPINF2 | 0.5079 |
| ENSP00000333203 | SERPINA5 | 0.5079 |
| ENSP00000348822 | SEMA4D | 0.5079 |
| ENSP00000382779 | SEH1L | 0.5079 |
| ENSP00000456434 | SDHD | 0.5079 |
| ENSP00000262878 | SAMHD1 | 0.5079 |
| ENSP00000298386 | RXFP2 | 0.5079 |
| ENSP00000250784 | RPS4Y1 | 0.5079 |
| ENSP00000202773 | RPL6 | 0.5079 |
| ENSP00000454786 | RP11-152F13.10 | 0.5079 |
| ENSP00000362578 | RNF8 | 0.5079 |
| ENSP00000360497 | RNF113A | 0.5079 |
| ENSP00000289013 | RHPN1 | 0.5079 |
| ENSP00000235382 | RGS2 | 0.5079 |
| ENSP00000295802 | RETSAT | 0.5079 |
| ENSP00000302088 | RER1 | 0.5079 |
| ENSP00000392423 | RELN | 0.5079 |
| ENSP00000257895 | RDH5 | 0.5079 |
| ENSP00000425446 | RBM4 | 0.5079 |
| ENSP00000265562 | PTPN23 | 0.5079 |
| ENSP00000394794 | PTPN13 | 0.5079 |
| ENSP00000210313 | PSMD5 | 0.5079 |
| ENSP00000364016 | PSMB8 | 0.5079 |
| ENSP00000225174 | PPIF | 0.5079 |
| ENSP00000393953 | PNPT1 | 0.5079 |
| ENSP00000326119 | PMAIP1 | 0.5079 |
| ENSP00000266505 | PLCZ1 | 0.5079 |
| ENSP00000380460 | PLAA | 0.5079 |
| ENSP00000356436 | PLA2G4A | 0.5079 |
| ENSP00000335333 | PIP5K1C | 0.5079 |
| ENSP00000268043 | PIF1 | 0.5079 |
| ENSP00000379678 | PGM5 | 0.5079 |
| ENSP00000295266 | PDHA2 | 0.5079 |
| ENSP00000337500 | PCGF5 | 0.5079 |
| ENSP00000391453 | PARVG | 0.5079 |

| | | |
|---|---|---|
| ENSP00000371419 | PARP4 | 0.5079 |
| ENSP00000260404 | PAK6 | 0.5079 |
| ENSP00000264883 | NUP54 | 0.5079 |
| ENSP00000219066 | NTHL1 | 0.5079 |
| ENSP00000354910 | NRG2 | 0.5079 |
| ENSP00000367407 | NMT2 | 0.5079 |
| ENSP00000376886 | NME1-NME2 | 0.5079 |
| ENSP00000259037 | NDUFB5 | 0.5079 |
| ENSP00000249299 | NAA38 | 0.5079 |
| ENSP00000288235 | MYO1E | 0.5079 |
| ENSP00000347055 | MYL4 | 0.5079 |
| ENSP00000278888 | MS4A2 | 0.5079 |
| ENSP00000279242 | MRPL49 | 0.5079 |
| ENSP00000380318 | MPST | 0.5079 |
| ENSP00000285814 | MKI67IP | 0.5079 |
| ENSP00000274473 | MEGF10 | 0.5079 |
| ENSP00000258648 | MED4 | 0.5079 |
| ENSP00000365107 | MASTL | 0.5079 |
| ENSP00000350195 | MAP3K6 | 0.5079 |
| ENSP00000260382 | LRRC49 | 0.5079 |
| ENSP00000244289 | LIPE | 0.5079 |
| ENSP00000344401 | LIN28B | 0.5079 |
| ENSP00000349393 | LIG4 | 0.5079 |
| ENSP00000215909 | LGALS1 | 0.5079 |
| ENSP00000272163 | LBR | 0.5079 |
| ENSP00000424183 | LAMTOR3 | 0.5079 |
| ENSP00000362728 | KPNA6 | 0.5079 |
| ENSP00000355517 | KMO | 0.5079 |
| ENSP00000356230 | KLHL12 | 0.5079 |
| ENSP00000385000 | KIF2A | 0.5079 |
| ENSP00000263934 | KIF1B | 0.5079 |
| ENSP00000367323 | KCNAB2 | 0.5079 |
| ENSP00000394085 | JMJD6 | 0.5079 |
| ENSP00000373854 | ITGAD | 0.5079 |
| ENSP00000304915 | IL13 | 0.5079 |
| ENSP00000259874 | IER3 | 0.5079 |
| ENSP00000303766 | HTRA3 | 0.5079 |
| ENSP00000218364 | HTATSF1 | 0.5079 |
| ENSP00000295757 | HDAC11 | 0.5079 |
| ENSP00000423822 | GPX8 | 0.5079 |
| ENSP00000261837 | GNB5 | 0.5079 |
| ENSP00000271348 | GJA5 | 0.5079 |
| ENSP00000253462 | GINS2 | 0.5079 |
| ENSP00000261386 | GDE1 | 0.5079 |
| ENSP00000318177 | FUBP3 | 0.5079 |
| ENSP00000302707 | FPR1 | 0.5079 |
| ENSP00000450895 | FPGT-TNNI3K | 0.5079 |
| ENSP00000335677 | FOXK2 | 0.5079 |
| ENSP00000335493 | FOXD2 | 0.5079 |
| ENSP00000304004 | FOXA3 | 0.5079 |
| ENSP00000310935 | FKBP2 | 0.5079 |
| ENSP00000399324 | FGF16 | 0.5079 |
| ENSP00000462972 | FDXR | 0.5079 |

| | | |
|---|---|---|
| ENSP00000463576 | FAM230A | 0.5079 |
| ENSP00000264344 | FAM13A | 0.5079 |
| ENSP00000256104 | FABP4 | 0.5079 |
| ENSP00000374354 | EXOSC8 | 0.5079 |
| ENSP00000265564 | EXOSC7 | 0.5079 |
| ENSP00000384524 | ETV2 | 0.5079 |
| ENSP00000362139 | EPHA10 | 0.5079 |
| ENSP00000307235 | EIF2AK3 | 0.5079 |
| ENSP00000368927 | EIF1AX | 0.5079 |
| ENSP00000268206 | EFTUD1 | 0.5079 |
| ENSP00000215368 | EFNA2 | 0.5079 |
| ENSP00000254730 | EEFSEC | 0.5079 |
| ENSP00000263026 | EEF2K | 0.5079 |
| ENSP00000382064 | DTNA | 0.5079 |
| ENSP00000306129 | DRD5 | 0.5079 |
| ENSP00000436901 | DPF2 | 0.5079 |
| ENSP00000262375 | DNAJA3 | 0.5079 |
| ENSP00000365625 | DHX16 | 0.5079 |
| ENSP00000311899 | DAW1 | 0.5079 |
| ENSP00000230895 | DAP | 0.5079 |
| ENSP00000299498 | CYB5R2 | 0.5079 |
| ENSP00000308430 | CYB5B | 0.5079 |
| ENSP00000292174 | CXCR5 | 0.5079 |
| ENSP00000352544 | CPLX2 | 0.5079 |
| ENSP00000222271 | COMP | 0.5079 |
| ENSP00000348385 | COL27A1 | 0.5079 |
| ENSP00000364140 | COL15A1 | 0.5079 |
| ENSP00000325506 | CLVS1 | 0.5079 |
| ENSP00000406577 | CKMT1A | 0.5079 |
| ENSP00000262570 | CHCHD3 | 0.5079 |
| ENSP00000359300 | CETN2 | 0.5079 |
| ENSP00000283006 | CENPH | 0.5079 |
| ENSP00000337358 | CECR5 | 0.5079 |
| ENSP00000234626 | CDC7 | 0.5079 |
| ENSP00000269385 | CBX8 | 0.5079 |
| ENSP00000376849 | CASP5 | 0.5079 |
| ENSP00000358147 | CACUL1 | 0.5079 |
| ENSP00000260210 | BUD13 | 0.5079 |
| ENSP00000306752 | BOLA2B | 0.5079 |
| ENSP00000250405 | BCL2L2 | 0.5079 |
| ENSP00000376534 | BAZ2B | 0.5079 |
| ENSP00000253413 | ATP6V1E1 | 0.5079 |
| ENSP00000318846 | ASCL3 | 0.5079 |
| ENSP00000368332 | ARX | 0.5079 |
| ENSP00000366487 | ARL5B | 0.5079 |
| ENSP00000323847 | ARL14 | 0.5079 |
| ENSP00000279873 | ARID5B | 0.5079 |
| ENSP00000336923 | ARHGAP10 | 0.5079 |
| ENSP00000356969 | APOA2 | 0.5079 |
| ENSP00000347408 | AP3M1 | 0.5079 |
| ENSP00000369579 | ANKDD1A | 0.5079 |
| ENSP00000394394 | ANAPC7 | 0.5079 |
| ENSP00000294517 | ADC | 0.5079 |

| | | |
|---|---|---|
| ENSP00000349396 | ABCD4 | 0.5079 |
| ENSP00000402835 | AARSD1 | 0.5079 |
| ENSP00000334474 | ZMIZ1 | 0.5053 |
| ENSP00000384434 | ZGLP1 | 0.5053 |
| ENSP00000261353 | YPEL5 | 0.5053 |
| ENSP00000362576 | YARS | 0.5053 |
| ENSP00000366396 | XRN2 | 0.5053 |
| ENSP00000328671 | WDR4 | 0.5053 |
| ENSP00000326534 | VPS39 | 0.5053 |
| ENSP00000318629 | VPS37A | 0.5053 |
| ENSP00000312029 | UCP2 | 0.5053 |
| ENSP00000363338 | UBXN11 | 0.5053 |
| ENSP00000380178 | UBE2G1 | 0.5053 |
| ENSP00000348838 | UBE2C | 0.5053 |
| ENSP00000239587 | TTLL2 | 0.5053 |
| ENSP00000303437 | TSNARE1 | 0.5053 |
| ENSP00000355991 | TRAF3IP3 | 0.5053 |
| ENSP00000216034 | TOMM22 | 0.5053 |
| ENSP00000376445 | TIRAP | 0.5053 |
| ENSP00000265686 | TCIRG1 | 0.5053 |
| ENSP00000402935 | TBC1D5 | 0.5053 |
| ENSP00000348234 | TAT | 0.5053 |
| ENSP00000217893 | TAF9 | 0.5053 |
| ENSP00000321106 | TAC1 | 0.5053 |
| ENSP00000216297 | SUPT16H | 0.5053 |
| ENSP00000363054 | STX12 | 0.5053 |
| ENSP00000194530 | STRADB | 0.5053 |
| ENSP00000378965 | SNTB1 | 0.5053 |
| ENSP00000272348 | SNRPG | 0.5053 |
| ENSP00000244520 | SNRPC | 0.5053 |
| ENSP00000406485 | SMTNL1 | 0.5053 |
| ENSP00000351947 | SMARCAD1 | 0.5053 |
| ENSP00000415011 | SLC30A8 | 0.5053 |
| ENSP00000355968 | SLC30A1 | 0.5053 |
| ENSP00000230640 | SKIV2L2 | 0.5053 |
| ENSP00000296257 | SENP2 | 0.5053 |
| ENSP00000260130 | SDCBP | 0.5053 |
| ENSP00000360569 | SCP2 | 0.5053 |
| ENSP00000316329 | SCD5 | 0.5053 |
| ENSP00000441823 | SALL3 | 0.5053 |
| ENSP00000273064 | RQCD1 | 0.5053 |
| ENSP00000287038 | RPL30 | 0.5053 |
| ENSP00000283646 | RPIA | 0.5053 |
| ENSP00000380903 | ROBO3 | 0.5053 |
| ENSP00000360441 | RHOXF2 | 0.5053 |
| ENSP00000219551 | RHBDL1 | 0.5053 |
| ENSP00000365946 | RBM3 | 0.5053 |
| ENSP00000369218 | RBM17 | 0.5053 |
| ENSP00000264515 | RBBP5 | 0.5053 |
| ENSP00000338173 | PUM2 | 0.5053 |
| ENSP00000324122 | PRPF31 | 0.5053 |
| ENSP00000279387 | PPP4C | 0.5053 |
| ENSP00000260970 | PPIG | 0.5053 |

| | | |
|---|---|---|
| ENSP00000313731 | PLCD3 | 0.5053 |
| ENSP00000316809 | PITPNA | 0.5053 |
| ENSP00000298231 | PHOX2A | 0.5053 |
| ENSP00000356047 | PFKFB2 | 0.5053 |
| ENSP00000379503 | PDP1 | 0.5053 |
| ENSP00000345502 | PDE4D | 0.5053 |
| ENSP00000347689 | PDE4C | 0.5053 |
| ENSP00000286063 | PDE11A | 0.5053 |
| ENSP00000384700 | PAPOLB | 0.5053 |
| ENSP00000298838 | PACSIN3 | 0.5053 |
| ENSP00000361914 | OXCT2 | 0.5053 |
| ENSP00000039007 | OTC | 0.5053 |
| ENSP00000378504 | NXF3 | 0.5053 |
| ENSP00000281701 | NVL | 0.5053 |
| ENSP00000318986 | NSUN3 | 0.5053 |
| ENSP00000345147 | NKX2-4 | 0.5053 |
| ENSP00000380781 | NFIX | 0.5053 |
| ENSP00000356972 | NDUFS2 | 0.5053 |
| ENSP00000360492 | NDUFA1 | 0.5053 |
| ENSP00000263354 | NAPA | 0.5053 |
| ENSP00000367428 | MS4A13 | 0.5053 |
| ENSP00000260302 | MMP13 | 0.5053 |
| ENSP00000279441 | MMP10 | 0.5053 |
| ENSP00000281928 | MED13L | 0.5053 |
| ENSP00000385751 | MARK2 | 0.5053 |
| ENSP00000013125 | MAP4K5 | 0.5053 |
| ENSP00000451263 | MAP3K9 | 0.5053 |
| ENSP00000346151 | MAGI2 | 0.5053 |
| ENSP00000231751 | LTF | 0.5053 |
| ENSP00000353826 | LONP1 | 0.5053 |
| ENSP00000393379 | KIF5C | 0.5053 |
| ENSP00000363524 | KIF4A | 0.5053 |
| ENSP00000160827 | KIF22 | 0.5053 |
| ENSP00000382770 | KCNU1 | 0.5053 |
| ENSP00000369042 | IPO7 | 0.5053 |
| ENSP00000380024 | ING4 | 0.5053 |
| ENSP00000326981 | IMP3 | 0.5053 |
| ENSP00000391826 | IGF2 | 0.5053 |
| ENSP00000390158 | HERC1 | 0.5053 |
| ENSP00000282058 | HAUS1 | 0.5053 |
| ENSP00000355316 | GRM3 | 0.5053 |
| ENSP00000360608 | GRIN1 | 0.5053 |
| ENSP00000366246 | GPC6 | 0.5053 |
| ENSP00000311876 | GNPDA1 | 0.5053 |
| ENSP00000363411 | GNG10 | 0.5053 |
| ENSP00000308622 | GLE1 | 0.5053 |
| ENSP00000384363 | GDPD3 | 0.5053 |
| ENSP00000359497 | GBP2 | 0.5053 |
| ENSP00000453129 | GALK2 | 0.5053 |
| ENSP00000262426 | FOXF1 | 0.5053 |
| ENSP00000377311 | FDX1L | 0.5053 |
| ENSP00000304108 | FAM171B | 0.5053 |
| ENSP00000219368 | FA2H | 0.5053 |

| | | |
|---|---|---|
| ENSP00000264870 | F13A1 | 0.5053 |
| ENSP00000252482 | EXOC3L2 | 0.5053 |
| ENSP00000426638 | ETFDH | 0.5053 |
| ENSP00000414378 | EFNA4 | 0.5053 |
| ENSP00000364028 | ECE1 | 0.5053 |
| ENSP00000354859 | DRD2 | 0.5053 |
| ENSP00000377523 | DQX1 | 0.5053 |
| ENSP00000276651 | DPYS | 0.5053 |
| ENSP00000285311 | DKK2 | 0.5053 |
| ENSP00000331907 | DHX34 | 0.5053 |
| ENSP00000347717 | DHCR7 | 0.5053 |
| ENSP00000360316 | DHCR24 | 0.5053 |
| ENSP00000241051 | DEPDC7 | 0.5053 |
| ENSP00000392762 | DCT | 0.5053 |
| ENSP00000366466 | CTNNBIP1 | 0.5053 |
| ENSP00000198765 | CPNE3 | 0.5053 |
| ENSP00000258654 | COG3 | 0.5053 |
| ENSP00000279463 | CNTN5 | 0.5053 |
| ENSP00000302569 | CLEC7A | 0.5053 |
| ENSP00000288861 | CIB4 | 0.5053 |
| ENSP00000261007 | CHRNA1 | 0.5053 |
| ENSP00000251363 | CERS4 | 0.5053 |
| ENSP00000388566 | CASP4 | 0.5053 |
| ENSP00000360797 | CARD9 | 0.5053 |
| ENSP00000347005 | C7orf55-LUC7L2 | 0.5053 |
| ENSP00000313967 | C1QB | 0.5053 |
| ENSP00000306477 | BTD | 0.5053 |
| ENSP00000283147 | BMP6 | 0.5053 |
| ENSP00000318799 | BHLHE22 | 0.5053 |
| ENSP00000323099 | ATRIP | 0.5053 |
| ENSP00000292475 | ATP5J2 | 0.5053 |
| ENSP00000358737 | ATP5F1 | 0.5053 |
| ENSP00000263519 | ATP2B3 | 0.5053 |
| ENSP00000358159 | ASCC3 | 0.5053 |
| ENSP00000368994 | ARHGAP44 | 0.5053 |
| ENSP00000361027 | AMOT | 0.5053 |
| ENSP00000329117 | AMER1 | 0.5053 |
| ENSP00000295463 | ALPI | 0.5053 |
| ENSP00000255084 | ALDH3B2 | 0.5053 |
| ENSP00000225740 | ALDH3A1 | 0.5053 |
| ENSP00000309259 | ALAS1 | 0.5053 |
| ENSP00000386284 | ALAD | 0.5053 |
| ENSP00000229335 | AICDA | 0.5053 |
| ENSP00000301732 | ABCA3 | 0.5053 |
| ENSP00000443824 | ZNRF3 | 0.5026 |
| ENSP00000400113 | ZDHHC13 | 0.5026 |
| ENSP00000338788 | ZC3H15 | 0.5026 |
| ENSP00000282007 | ZC3H13 | 0.5026 |
| ENSP00000366098 | YIF1A | 0.5026 |
| ENSP00000401859 | YAF2 | 0.5026 |
| ENSP00000360147 | XPNPEP2 | 0.5026 |
| ENSP00000296490 | WDR82 | 0.5026 |
| ENSP00000324636 | VRK3 | 0.5026 |

| | | |
|---|---|---|
| ENSP00000313811 | USP20 | 0.5026 |
| ENSP00000263966 | USP13 | 0.5026 |
| ENSP00000354201 | UBE2Z | 0.5026 |
| ENSP00000366249 | UBD | 0.5026 |
| ENSP00000364591 | TSPYL2 | 0.5026 |
| ENSP00000339804 | TSLP | 0.5026 |
| ENSP00000302801 | TRMT61B | 0.5026 |
| ENSP00000366729 | TNFRSF9 | 0.5026 |
| ENSP00000350965 | TMBIM4 | 0.5026 |
| ENSP00000450607 | TIMELESS | 0.5026 |
| ENSP00000355896 | TGFB2 | 0.5026 |
| ENSP00000262715 | TEP1 | 0.5026 |
| ENSP00000313886 | TCEANC | 0.5026 |
| ENSP00000298630 | STK32C | 0.5026 |
| ENSP00000360922 | STAU1 | 0.5026 |
| ENSP00000305230 | SRP9 | 0.5026 |
| ENSP00000295050 | SPRTN | 0.5026 |
| ENSP00000310620 | SNX32 | 0.5026 |
| ENSP00000363349 | SNX30 | 0.5026 |
| ENSP00000311837 | SNTG2 | 0.5026 |
| ENSP00000246071 | SNRPB2 | 0.5026 |
| ENSP00000412566 | SNRPB | 0.5026 |
| ENSP00000346762 | SND1 | 0.5026 |
| ENSP00000338606 | SMUG1 | 0.5026 |
| ENSP00000286398 | SMC2 | 0.5026 |
| ENSP00000298681 | SLC39A2 | 0.5026 |
| ENSP00000461324 | SLC25A10 | 0.5026 |
| ENSP00000270162 | SIK1 | 0.5026 |
| ENSP00000442518 | SHANK3 | 0.5026 |
| ENSP00000422168 | SH3BP2 | 0.5026 |
| ENSP00000381992 | SEPT7 | 0.5026 |
| ENSP00000296504 | SAP30 | 0.5026 |
| ENSP00000346045 | RPS17 | 0.5026 |
| ENSP00000270625 | RPS11 | 0.5026 |
| ENSP00000346012 | RPL36AL | 0.5026 |
| ENSP00000393393 | RPL35A | 0.5026 |
| ENSP00000378160 | RPL34 | 0.5026 |
| ENSP00000378782 | QPRT | 0.5026 |
| ENSP00000434359 | PUF60 | 0.5026 |
| ENSP00000412788 | PTBP2 | 0.5026 |
| ENSP00000377384 | PSMC3IP | 0.5026 |
| ENSP00000318602 | PRTFDC1 | 0.5026 |
| ENSP00000296223 | POLR2H | 0.5026 |
| ENSP00000308937 | PMP22 | 0.5026 |
| ENSP00000358696 | PLXNA3 | 0.5026 |
| ENSP00000364252 | PLA2G2A | 0.5026 |
| ENSP00000245255 | PIWIL1 | 0.5026 |
| ENSP00000359665 | PI4K2A | 0.5026 |
| ENSP00000330918 | PGP | 0.5026 |
| ENSP00000270776 | PGD | 0.5026 |
| ENSP00000309548 | PDP2 | 0.5026 |
| ENSP00000334319 | PCLO | 0.5026 |
| ENSP00000334008 | PARVA | 0.5026 |

| | | |
|---|---|---|
| ENSP00000453403 | NUSAP1 | 0.5026 |
| ENSP00000357480 | NUS1 | 0.5026 |
| ENSP00000420267 | NR6A1 | 0.5026 |
| ENSP00000382392 | NOP2 | 0.5026 |
| ENSP00000332198 | NOP10 | 0.5026 |
| ENSP00000296684 | NDUFS4 | 0.5026 |
| ENSP00000330737 | NDUFA12 | 0.5026 |
| ENSP00000366225 | NAPB | 0.5026 |
| ENSP00000325239 | MYLPF | 0.5026 |
| ENSP00000402038 | MTO1 | 0.5026 |
| ENSP00000223324 | MRPL32 | 0.5026 |
| ENSP00000377252 | MON2 | 0.5026 |
| ENSP00000370880 | MLANA | 0.5026 |
| ENSP00000267812 | MFAP1 | 0.5026 |
| ENSP00000255764 | MED10 | 0.5026 |
| ENSP00000403946 | MBTD1 | 0.5026 |
| ENSP00000310933 | MADD | 0.5026 |
| ENSP00000348762 | LSS | 0.5026 |
| ENSP00000410758 | LSM5 | 0.5026 |
| ENSP00000355963 | LPGAT1 | 0.5026 |
| ENSP00000356257 | LMOD1 | 0.5026 |
| ENSP00000155840 | KCNQ1 | 0.5026 |
| ENSP00000263087 | ITGAE | 0.5026 |
| ENSP00000249842 | ISLR | 0.5026 |
| ENSP00000364929 | ING1 | 0.5026 |
| ENSP00000262345 | IL12RB2 | 0.5026 |
| ENSP00000412237 | IL10 | 0.5026 |
| ENSP00000317980 | IGSF9B | 0.5026 |
| ENSP00000356713 | IFNGR1 | 0.5026 |
| ENSP00000263043 | ICK | 0.5026 |
| ENSP00000160262 | ICAM3 | 0.5026 |
| ENSP00000285667 | HSPA13 | 0.5026 |
| ENSP00000297679 | HSD3B7 | 0.5026 |
| ENSP00000304226 | HIPK3 | 0.5026 |
| ENSP00000312042 | HDLBP | 0.5026 |
| ENSP00000366620 | H6PD | 0.5026 |
| ENSP00000373730 | H2AFJ | 0.5026 |
| ENSP00000053867 | GRN | 0.5026 |
| ENSP00000264426 | GRIA2 | 0.5026 |
| ENSP00000263915 | GRB14 | 0.5026 |
| ENSP00000442770 | GPN3 | 0.5026 |
| ENSP00000226413 | GNRHR | 0.5026 |
| ENSP00000309092 | GMPPB | 0.5026 |
| ENSP00000321706 | GEMIN4 | 0.5026 |
| ENSP00000378890 | GCH1 | 0.5026 |
| ENSP00000371110 | GCAT | 0.5026 |
| ENSP00000350928 | GAD1 | 0.5026 |
| ENSP00000357158 | FCRL1 | 0.5026 |
| ENSP00000298047 | FAT3 | 0.5026 |
| ENSP00000218099 | F9 | 0.5026 |
| ENSP00000398597 | EXOSC6 | 0.5026 |
| ENSP00000225371 | EPX | 0.5026 |
| ENSP00000438095 | ELOVL5 | 0.5026 |

| | | |
|---|---|---|
| ENSP00000216190 | EIF3D | 0.5026 |
| ENSP00000356076 | DYRK3 | 0.5026 |
| ENSP00000288943 | DUSP2 | 0.5026 |
| ENSP00000384084 | DTNB | 0.5026 |
| ENSP00000346142 | DPAGT1 | 0.5026 |
| ENSP00000221403 | DHDH | 0.5026 |
| ENSP00000374574 | DDX42 | 0.5026 |
| ENSP00000344989 | DALRD3 | 0.5026 |
| ENSP00000369050 | CYP1A1 | 0.5026 |
| ENSP00000444856 | CUL2 | 0.5026 |
| ENSP00000217131 | CTSZ | 0.5026 |
| ENSP00000356954 | CTGF | 0.5026 |
| ENSP00000356016 | CR1 | 0.5026 |
| ENSP00000386104 | CPE | 0.5026 |
| ENSP00000330633 | CNTN2 | 0.5026 |
| ENSP00000312995 | CLSPN | 0.5026 |
| ENSP00000376306 | CIT | 0.5026 |
| ENSP00000246006 | CD93 | 0.5026 |
| ENSP00000227155 | CD82 | 0.5026 |
| ENSP00000317214 | CAPN6 | 0.5026 |
| ENSP00000365441 | CACNA1F | 0.5026 |
| ENSP00000358146 | BOLA1 | 0.5026 |
| ENSP00000376946 | BBS10 | 0.5026 |
| ENSP00000415822 | ATXN1L | 0.5026 |
| ENSP00000417378 | ATP6V1F | 0.5026 |
| ENSP00000256031 | ATP13A3 | 0.5026 |
| ENSP00000311766 | ATAD3B | 0.5026 |
| ENSP00000356992 | ARHGAP30 | 0.5026 |
| ENSP00000254584 | ARFIP2 | 0.5026 |
| ENSP00000261733 | ALDH2 | 0.5026 |
| ENSP00000379138 | AFP | 0.5026 |
| ENSP00000294016 | ADCY9 | 0.5026 |
| ENSP00000419361 | ADCY5 | 0.5026 |
| ENSP00000237283 | ADAT2 | 0.5026 |
| ENSP00000271643 | ADAMTSL4 | 0.5026 |
| ENSP00000243349 | ACVR1C | 0.5026 |
| ENSP00000188312 | ACTR6 | 0.5026 |
| ENSP00000348775 | ACOX3 | 0.5026 |
| ENSP00000281182 | ACAD8 | 0.5026 |
| ENSP00000365725 | ABHD12 | 0.5026 |
| ENSP00000273359 | ABHD10 | 0.5026 |
| ENSP00000205557 | ABCC6 | 0.5026 |
| ENSP00000359478 | ABCC2 | 0.5026 |
| ENSP00000265316 | ABCB6 | 0.5026 |
| ENSP00000268251 | ABAT | 0.5026 |
| ENSP00000387841 | MRRFP1 | 0.5026 |
| ENSP00000421566 | XPNPEP1 | 0.5000 |
| ENSP00000262144 | WDR59 | 0.5000 |
| ENSP00000376792 | VTI1A | 0.5000 |
| ENSP00000311245 | USMG5 | 0.5000 |
| ENSP00000276893 | UHRF2 | 0.5000 |
| ENSP00000263228 | UBE2R2 | 0.5000 |
| ENSP00000323687 | UBE2O | 0.5000 |

| | | |
|---|---|---|
| ENSP00000338348 | UBE2G2 | 0.5000 |
| ENSP00000269601 | TXNL4A | 0.5000 |
| ENSP00000363641 | TXN | 0.5000 |
| ENSP00000367743 | TSPAN7 | 0.5000 |
| ENSP00000262213 | TRAM1 | 0.5000 |
| ENSP00000431872 | TPT1 | 0.5000 |
| ENSP00000354842 | TOX | 0.5000 |
| ENSP00000347948 | TNFRSF14 | 0.5000 |
| ENSP00000297632 | TMEM65 | 0.5000 |
| ENSP00000258412 | TMBIM1 | 0.5000 |
| ENSP00000270538 | TIMM44 | 0.5000 |
| ENSP00000416330 | TGFBI | 0.5000 |
| ENSP00000430690 | TFF3 | 0.5000 |
| ENSP00000387303 | TDRD9 | 0.5000 |
| ENSP00000321259 | TALDO1 | 0.5000 |
| ENSP00000263033 | SYTL4 | 0.5000 |
| ENSP00000221283 | STXBP2 | 0.5000 |
| ENSP00000356918 | STX7 | 0.5000 |
| ENSP00000320679 | STX19 | 0.5000 |
| ENSP00000375777 | STRN4 | 0.5000 |
| ENSP00000291386 | SSU72 | 0.5000 |
| ENSP00000424870 | SRP19 | 0.5000 |
| ENSP00000267884 | SRP14 | 0.5000 |
| ENSP00000311489 | SPTBN2 | 0.5000 |
| ENSP00000355354 | SOX15 | 0.5000 |
| ENSP00000306223 | SNRPN | 0.5000 |
| ENSP00000337125 | SMEK1 | 0.5000 |
| ENSP00000306627 | SLC9C1 | 0.5000 |
| ENSP00000379834 | SLC2A14 | 0.5000 |
| ENSP00000377771 | SIPA1 | 0.5000 |
| ENSP00000398704 | SHOX2 | 0.5000 |
| ENSP00000403712 | SENP3 | 0.5000 |
| ENSP00000265362 | SEMA3A | 0.5000 |
| ENSP00000251170 | SEC14L5 | 0.5000 |
| ENSP00000368411 | SCNN1D | 0.5000 |
| ENSP00000357708 | S100A6 | 0.5000 |
| ENSP00000301336 | RILP | 0.5000 |
| ENSP00000451030 | RGS6 | 0.5000 |
| ENSP00000319308 | RGS5 | 0.5000 |
| ENSP00000356430 | RGS18 | 0.5000 |
| ENSP00000265881 | REXO2 | 0.5000 |
| ENSP00000261973 | RBM25 | 0.5000 |
| ENSP00000361043 | RAD54L | 0.5000 |
| ENSP00000281273 | QTRTD1 | 0.5000 |
| ENSP00000295718 | PTPRN | 0.5000 |
| ENSP00000298942 | PTER | 0.5000 |
| ENSP00000329127 | PRKCH | 0.5000 |
| ENSP00000417963 | PPP2R5D | 0.5000 |
| ENSP00000164133 | PPP2R5B | 0.5000 |
| ENSP00000202556 | PPP1R13B | 0.5000 |
| ENSP00000286719 | PPEF2 | 0.5000 |
| ENSP00000265465 | POLA2 | 0.5000 |
| ENSP00000346810 | PLAGL1 | 0.5000 |

| | | |
|---|---|---|
| ENSP00000291906 | PKN3 | 0.5000 |
| ENSP00000349465 | PICK1 | 0.5000 |
| ENSP00000243052 | PDE1B | 0.5000 |
| ENSP00000335062 | PDCD1 | 0.5000 |
| ENSP00000292823 | PCYT1A | 0.5000 |
| ENSP00000365462 | PCCA | 0.5000 |
| ENSP00000396995 | PAPD5 | 0.5000 |
| ENSP00000234961 | OPRD1 | 0.5000 |
| ENSP00000426978 | NXF5 | 0.5000 |
| ENSP00000345895 | NUP50 | 0.5000 |
| ENSP00000268533 | NUDT7 | 0.5000 |
| ENSP00000337773 | NQO2 | 0.5000 |
| ENSP00000382834 | NPRL3 | 0.5000 |
| ENSP00000264663 | NNT | 0.5000 |
| ENSP00000442308 | NKRF | 0.5000 |
| ENSP00000215956 | NHP2L1 | 0.5000 |
| ENSP00000344786 | NFASC | 0.5000 |
| ENSP00000309899 | NARF | 0.5000 |
| ENSP00000210444 | NANS | 0.5000 |
| ENSP00000437073 | MYO18A | 0.5000 |
| ENSP00000316950 | MYEF2 | 0.5000 |
| ENSP00000354813 | MT-ND5 | 0.5000 |
| ENSP00000354687 | MT-ND1 | 0.5000 |
| ENSP00000355692 | MRPL55 | 0.5000 |
| ENSP00000300151 | MRPL16 | 0.5000 |
| ENSP00000362282 | MOCS1 | 0.5000 |
| ENSP00000379364 | MOB1A | 0.5000 |
| ENSP00000261245 | MNAT1 | 0.5000 |
| ENSP00000361064 | MINPP1 | 0.5000 |
| ENSP00000315152 | METAP1D | 0.5000 |
| ENSP00000296411 | METAP1 | 0.5000 |
| ENSP00000356070 | MAPKAPK2 | 0.5000 |
| ENSP00000294066 | MAP4K2 | 0.5000 |
| ENSP00000261921 | LOXL1 | 0.5000 |
| ENSP00000365159 | ISCA1 | 0.5000 |
| ENSP00000242208 | INHBA | 0.5000 |
| ENSP00000387262 | IMMT | 0.5000 |
| ENSP00000396301 | IK | 0.5000 |
| ENSP00000384886 | IFI30 | 0.5000 |
| ENSP00000303599 | HSFY1 | 0.5000 |
| ENSP00000305632 | HOMER2 | 0.5000 |
| ENSP00000265866 | HNRNPH3 | 0.5000 |
| ENSP00000381654 | HMGCLL1 | 0.5000 |
| ENSP00000338387 | HLCS | 0.5000 |
| ENSP00000353472 | HLA-G | 0.5000 |
| ENSP00000277903 | HABP2 | 0.5000 |
| ENSP00000381607 | GSTP1 | 0.5000 |
| ENSP00000362656 | GOLGA1 | 0.5000 |
| ENSP00000295448 | GNPDA2 | 0.5000 |
| ENSP00000361894 | GNMT | 0.5000 |
| ENSP00000248150 | GNG13 | 0.5000 |
| ENSP00000266659 | GLIPR1 | 0.5000 |
| ENSP00000372295 | GJB2 | 0.5000 |

| | | |
|---|---|---|
| ENSP00000338964 | GGT7 | 0.5000 |
| ENSP00000359258 | GCLM | 0.5000 |
| ENSP00000359804 | FUBP1 | 0.5000 |
| ENSP00000329219 | FMNL1 | 0.5000 |
| ENSP00000364475 | FBP1 | 0.5000 |
| ENSP00000361433 | EXOSC2 | 0.5000 |
| ENSP00000379042 | ERO1L | 0.5000 |
| ENSP00000276461 | ERLIN2 | 0.5000 |
| ENSP00000185150 | ERLEC1 | 0.5000 |
| ENSP00000332118 | EPHB3 | 0.5000 |
| ENSP00000354125 | EIF3B | 0.5000 |
| ENSP00000327116 | EHD3 | 0.5000 |
| ENSP00000256497 | EDEM1 | 0.5000 |
| ENSP00000258443 | EDAR | 0.5000 |
| ENSP00000356130 | DSTYK | 0.5000 |
| ENSP00000273075 | DNPEP | 0.5000 |
| ENSP00000406463 | DNAJC7 | 0.5000 |
| ENSP00000378605 | DNAJC30 | 0.5000 |
| ENSP00000366800 | DNAJC11 | 0.5000 |
| ENSP00000366179 | DNAJC1 | 0.5000 |
| ENSP00000359799 | DNAJB4 | 0.5000 |
| ENSP00000386770 | DNAH10 | 0.5000 |
| ENSP00000266070 | DIDO1 | 0.5000 |
| ENSP00000251241 | DHX40 | 0.5000 |
| ENSP00000251636 | DHX29 | 0.5000 |
| ENSP00000288490 | DGKI | 0.5000 |
| ENSP00000328405 | DGKA | 0.5000 |
| ENSP00000359361 | DDX43 | 0.5000 |
| ENSP00000355185 | DCLRE1A | 0.5000 |
| ENSP00000390475 | CXXC1 | 0.5000 |
| ENSP00000354901 | CXCL9 | 0.5000 |
| ENSP00000364589 | CUL4A | 0.5000 |
| ENSP00000321732 | CTDNEP1 | 0.5000 |
| ENSP00000343746 | CORO2A | 0.5000 |
| ENSP00000261401 | CORO1C | 0.5000 |
| ENSP00000333946 | COQ6 | 0.5000 |
| ENSP00000263960 | COQ10B | 0.5000 |
| ENSP00000295550 | COL6A3 | 0.5000 |
| ENSP00000325146 | COL12A1 | 0.5000 |
| ENSP00000387122 | CLEC16A | 0.5000 |
| ENSP00000317468 | CHMP6 | 0.5000 |
| ENSP00000256443 | CDK7 | 0.5000 |
| ENSP00000462701 | CDH8 | 0.5000 |
| ENSP00000267383 | CDH24 | 0.5000 |
| ENSP00000256447 | CD180 | 0.5000 |
| ENSP00000377958 | CCT4 | 0.5000 |
| ENSP00000282572 | CCNO | 0.5000 |
| ENSP00000004921 | CCL18 | 0.5000 |
| ENSP00000265164 | CASP6 | 0.5000 |
| ENSP00000424038 | CASP12 | 0.5000 |
| ENSP00000310649 | BRSK1 | 0.5000 |
| ENSP00000217169 | BIRC7 | 0.5000 |
| ENSP00000245157 | BBS2 | 0.5000 |

| | | |
|---|---|---|
| ENSP00000332247 | ATP6V0A2 | 0.5000 |
| ENSP00000316854 | ATOX1 | 0.5000 |
| ENSP00000329212 | ATF7 | 0.5000 |
| ENSP00000368030 | ATAD3A | 0.5000 |
| ENSP00000353210 | ARL9 | 0.5000 |
| ENSP00000260746 | ARL3 | 0.5000 |
| ENSP00000282032 | ARL14EP | 0.5000 |
| ENSP00000276211 | ARHGAP36 | 0.5000 |
| ENSP00000263245 | ARFGAP3 | 0.5000 |
| ENSP00000434442 | ARFGAP2 | 0.5000 |
| ENSP00000199280 | AQP2 | 0.5000 |
| ENSP00000083182 | APPBP2 | 0.5000 |
| ENSP00000265381 | APBA1 | 0.5000 |
| ENSP00000448165 | APAF1 | 0.5000 |
| ENSP00000344055 | AP3D1 | 0.5000 |
| ENSP00000341662 | AGMO | 0.5000 |
| ENSP00000364986 | AGMAT | 0.5000 |
| ENSP00000312262 | ADRBK1 | 0.5000 |
| ENSP00000443189 | AC073610.5 | 0.5000 |
| ENSP00000310688 | ABCD2 | 0.5000 |
| ENSP00000327652 | DRD1 | 0.5000 |
| ENSP00000331111 | ZNRD1 | 0.4974 |
| ENSP00000311679 | ZNF483 | 0.4974 |
| ENSP00000343557 | ZCCHC17 | 0.4974 |
| ENSP00000326813 | YOD1 | 0.4974 |
| ENSP00000274496 | YIPF5 | 0.4974 |
| ENSP00000320658 | YARS2 | 0.4974 |
| ENSP00000235090 | WDR77 | 0.4974 |
| ENSP00000339449 | WDR16 | 0.4974 |
| ENSP00000347495 | WARS | 0.4974 |
| ENSP00000357887 | VPS72 | 0.4974 |
| ENSP00000440594 | VNN3 | 0.4974 |
| ENSP00000322276 | VNN2 | 0.4974 |
| ENSP00000415434 | USP40 | 0.4974 |
| ENSP00000363397 | UGCG | 0.4974 |
| ENSP00000296786 | UBLCP1 | 0.4974 |
| ENSP00000451261 | UBE2J1 | 0.4974 |
| ENSP00000281741 | TXNDC16 | 0.4974 |
| ENSP00000369784 | TUBAL3 | 0.4974 |
| ENSP00000392252 | TTLL8 | 0.4974 |
| ENSP00000333920 | TTF1 | 0.4974 |
| ENSP00000247829 | TSPAN8 | 0.4974 |
| ENSP00000362936 | TRNAU1AP | 0.4974 |
| ENSP00000252487 | TOMM40 | 0.4974 |
| ENSP00000368818 | TOM1L2 | 0.4974 |
| ENSP00000252898 | TNNI2 | 0.4974 |
| ENSP00000248244 | TICAM1 | 0.4974 |
| ENSP00000355751 | THBS2 | 0.4974 |
| ENSP00000367440 | TDP2 | 0.4974 |
| ENSP00000326550 | TACC3 | 0.4974 |
| ENSP00000319104 | SUPT6H | 0.4974 |
| ENSP00000305810 | STX18 | 0.4974 |
| ENSP00000373684 | STK38L | 0.4974 |

| | | |
|---|---|---|
| ENSP00000276449 | STAR | 0.4974 |
| ENSP00000274008 | SPATA5 | 0.4974 |
| ENSP00000348984 | SON | 0.4974 |
| ENSP00000262418 | SLC4A1 | 0.4974 |
| ENSP00000359174 | SLC35A3 | 0.4974 |
| ENSP00000348019 | SLC17A5 | 0.4974 |
| ENSP00000216513 | SIX4 | 0.4974 |
| ENSP00000292055 | SIK3 | 0.4974 |
| ENSP00000246104 | SCRT2 | 0.4974 |
| ENSP00000255390 | SCO1 | 0.4974 |
| ENSP00000328968 | SCN5A | 0.4974 |
| ENSP00000228284 | SART3 | 0.4974 |
| ENSP00000358423 | RRAGD | 0.4974 |
| ENSP00000307272 | RPTOR | 0.4974 |
| ENSP00000472469 | RPS28 | 0.4974 |
| ENSP00000346046 | RPS17L | 0.4974 |
| ENSP00000296277 | RPL39L | 0.4974 |
| ENSP00000346015 | RPL27A | 0.4974 |
| ENSP00000257575 | RNFT2 | 0.4974 |
| ENSP00000417895 | RASA4B | 0.4974 |
| ENSP00000246194 | RALY | 0.4974 |
| ENSP00000250237 | QTRT1 | 0.4974 |
| ENSP00000254667 | PTPRE | 0.4974 |
| ENSP00000293362 | PSME3 | 0.4974 |
| ENSP00000417659 | PPM1L | 0.4974 |
| ENSP00000392257 | PPIL6 | 0.4974 |
| ENSP00000390427 | PPIL2 | 0.4974 |
| ENSP00000241436 | POLK | 0.4974 |
| ENSP00000458021 | PMF1 | 0.4974 |
| ENSP00000305152 | PLP1 | 0.4974 |
| ENSP00000466248 | PLK5 | 0.4974 |
| ENSP00000274289 | PLK2 | 0.4974 |
| ENSP00000297540 | PHAX | 0.4974 |
| ENSP00000292357 | PEAR1 | 0.4974 |
| ENSP00000323313 | PDIA5 | 0.4974 |
| ENSP00000376506 | PDCD10 | 0.4974 |
| ENSP00000263556 | P4HA1 | 0.4974 |
| ENSP00000419740 | NSUN4 | 0.4974 |
| ENSP00000376889 | NME2 | 0.4974 |
| ENSP00000307525 | NMD3 | 0.4974 |
| ENSP00000356988 | NIT1 | 0.4974 |
| ENSP00000362058 | NDUFS5 | 0.4974 |
| ENSP00000293404 | NAGS | 0.4974 |
| ENSP00000037502 | MYOC | 0.4974 |
| ENSP00000331152 | MXI1 | 0.4974 |
| ENSP00000436812 | MUC5B | 0.4974 |
| ENSP00000370508 | MTIF3 | 0.4974 |
| ENSP00000364855 | MSH5 | 0.4974 |
| ENSP00000276585 | MRPS28 | 0.4974 |
| ENSP00000252602 | MRPL34 | 0.4974 |
| ENSP00000355583 | MLK4 | 0.4974 |
| ENSP00000342924 | MCPH1 | 0.4974 |
| ENSP00000383234 | MAPK4 | 0.4974 |

| | | |
|---|---|---|
| ENSP00000385450 | MAGI1 | 0.4974 |
| ENSP00000376048 | MAG | 0.4974 |
| ENSP00000302160 | LSM3 | 0.4974 |
| ENSP00000362354 | LPIN3 | 0.4974 |
| ENSP00000329102 | LIN9 | 0.4974 |
| ENSP00000336740 | LIMK1 | 0.4974 |
| ENSP00000226299 | LAP3 | 0.4974 |
| ENSP00000310878 | KLF14 | 0.4974 |
| ENSP00000264501 | KIAA1109 | 0.4974 |
| ENSP00000302719 | KCNAB3 | 0.4974 |
| ENSP00000419952 | KCNAB1 | 0.4974 |
| ENSP00000351190 | ITIH2 | 0.4974 |
| ENSP00000307183 | ING2 | 0.4974 |
| ENSP00000295666 | IGFBP7 | 0.4974 |
| ENSP00000232003 | HRG | 0.4974 |
| ENSP00000380785 | HOOK2 | 0.4974 |
| ENSP00000349430 | HIST1H2BK | 0.4974 |
| ENSP00000362674 | HDAC8 | 0.4974 |
| ENSP00000222574 | HBP1 | 0.4974 |
| ENSP00000338082 | HBG2 | 0.4974 |
| ENSP00000333994 | HBB | 0.4974 |
| ENSP00000304440 | HAS3 | 0.4974 |
| ENSP00000216410 | GNPNAT1 | 0.4974 |
| ENSP00000377665 | GAPVD1 | 0.4974 |
| ENSP00000274547 | GABRB2 | 0.4974 |
| ENSP00000350170 | FXR1 | 0.4974 |
| ENSP00000347041 | FMOD | 0.4974 |
| ENSP00000230124 | FIG4 | 0.4974 |
| ENSP00000394487 | FGD4 | 0.4974 |
| ENSP00000356936 | FCRLA | 0.4974 |
| ENSP00000281623 | FBXO4 | 0.4974 |
| ENSP00000405738 | ESRP1 | 0.4974 |
| ENSP00000222329 | ERF | 0.4974 |
| ENSP00000350896 | EPHB4 | 0.4974 |
| ENSP00000231887 | EHHADH | 0.4974 |
| ENSP00000226091 | EFNB3 | 0.4974 |
| ENSP00000360577 | ECHDC2 | 0.4974 |
| ENSP00000323275 | DYX1C1 | 0.4974 |
| ENSP00000302936 | DYNLRB2 | 0.4974 |
| ENSP00000452398 | DUXA | 0.4974 |
| ENSP00000355866 | DUSP10 | 0.4974 |
| ENSP00000383303 | DSCAM | 0.4974 |
| ENSP00000382104 | DOPEY2 | 0.4974 |
| ENSP00000256935 | DOCK2 | 0.4974 |
| ENSP00000382260 | DGKB | 0.4974 |
| ENSP00000158771 | DERL2 | 0.4974 |
| ENSP00000417748 | DDI2 | 0.4974 |
| ENSP00000358576 | DCLRE1B | 0.4974 |
| ENSP00000299441 | DCHS1 | 0.4974 |
| ENSP00000341504 | DCAKD | 0.4974 |
| ENSP00000292427 | CYP11B1 | 0.4974 |
| ENSP00000306884 | CXCL11 | 0.4974 |
| ENSP00000417656 | COX7B | 0.4974 |

| | | |
|---|---|---|
| ENSP00000310873 | COQ2 | 0.4974 |
| ENSP00000354511 | COMT | 0.4974 |
| ENSP00000369862 | COL4A3BP | 0.4974 |
| ENSP00000407546 | CHRNA7 | 0.4974 |
| ENSP00000378350 | CDC23 | 0.4974 |
| ENSP00000348554 | CDC16 | 0.4974 |
| ENSP00000357153 | CD1D | 0.4974 |
| ENSP00000264157 | CCNT2 | 0.4974 |
| ENSP00000368005 | CCNI2 | 0.4974 |
| ENSP00000381795 | CCDC33 | 0.4974 |
| ENSP00000265872 | CCAR1 | 0.4974 |
| ENSP00000264202 | CAPZB | 0.4974 |
| ENSP00000375844 | CAPN10 | 0.4974 |
| ENSP00000158166 | CAMKK1 | 0.4974 |
| ENSP00000267103 | C12orf10 | 0.4974 |
| ENSP00000334836 | BMP2K | 0.4974 |
| ENSP00000412957 | BIRC8 | 0.4974 |
| ENSP00000351575 | BDP1 | 0.4974 |
| ENSP00000451320 | BCL2L2-PABPN1 | 0.4974 |
| ENSP00000364683 | BAMBI | 0.4974 |
| ENSP00000375872 | ATG16L1 | 0.4974 |
| ENSP00000349887 | ASNA1 | 0.4974 |
| ENSP00000307188 | ASL | 0.4974 |
| ENSP00000378611 | ARHGAP24 | 0.4974 |
| ENSP00000348714 | AREL1 | 0.4974 |
| ENSP00000325369 | AP3S1 | 0.4974 |
| ENSP00000335287 | ANKRD46 | 0.4974 |
| ENSP00000312606 | AKR1A1 | 0.4974 |
| ENSP00000332448 | ADAT3 | 0.4974 |
| ENSP00000390849 | ABHD5 | 0.4974 |
| ENSP00000351717 | ABCB8 | 0.4974 |
| ENSP00000328808 | TUBB8 | 0.4974 |
| ENSP00000403936 | ENSG00000232856 | 0.4974 |
| ENSP00000311429 | ZWILCH | 0.4947 |
| ENSP00000303015 | ZRSR2 | 0.4947 |
| ENSP00000219593 | ZP2 | 0.4947 |
| ENSP00000274712 | ZMAT2 | 0.4947 |
| ENSP00000282928 | ZIC1 | 0.4947 |
| ENSP00000257177 | ZCCHC11 | 0.4947 |
| ENSP00000362135 | YRDC | 0.4947 |
| ENSP00000318480 | YME1L1 | 0.4947 |
| ENSP00000332772 | YIPF7 | 0.4947 |
| ENSP00000370150 | WRNIP1 | 0.4947 |
| ENSP00000450731 | VTI1B | 0.4947 |
| ENSP00000351492 | UBL5 | 0.4947 |
| ENSP00000309198 | UBE3C | 0.4947 |
| ENSP00000333326 | TUBA8 | 0.4947 |
| ENSP00000362255 | TSPO2 | 0.4947 |
| ENSP00000286349 | TRIM42 | 0.4947 |
| ENSP00000232975 | TNNC1 | 0.4947 |
| ENSP00000299705 | TMED3 | 0.4947 |
| ENSP00000354911 | TIFA | 0.4947 |
| ENSP00000379612 | TAX1BP1 | 0.4947 |

| | | |
|---|---|---|
| ENSP00000307684 | TADA3 | 0.4947 |
| ENSP00000341489 | SYDE1 | 0.4947 |
| ENSP00000360183 | STX16 | 0.4947 |
| ENSP00000455047 | SQRDL | 0.4947 |
| ENSP00000296695 | SPINK1 | 0.4947 |
| ENSP00000366988 | SNX5 | 0.4947 |
| ENSP00000327968 | SNAI3 | 0.4947 |
| ENSP00000358605 | SMNDC1 | 0.4947 |
| ENSP00000363229 | SLC18A3 | 0.4947 |
| ENSP00000372191 | SIRT3 | 0.4947 |
| ENSP00000354829 | SGMS1 | 0.4947 |
| ENSP00000356541 | SF3B5 | 0.4947 |
| ENSP00000318861 | SF3B2 | 0.4947 |
| ENSP00000224140 | SETX | 0.4947 |
| ENSP00000316203 | SEC14L2 | 0.4947 |
| ENSP00000278282 | SCGB1A1 | 0.4947 |
| ENSP00000357336 | RUSC1 | 0.4947 |
| ENSP00000295930 | RSRC1 | 0.4947 |
| ENSP00000291536 | RSPH1 | 0.4947 |
| ENSP00000435096 | RPS25 | 0.4947 |
| ENSP00000359345 | RPL5 | 0.4947 |
| ENSP00000307889 | RPL13 | 0.4947 |
| ENSP00000305071 | RFXANK | 0.4947 |
| ENSP00000395449 | RECQL | 0.4947 |
| ENSP00000320768 | RCAN1 | 0.4947 |
| ENSP00000317872 | RBBP6 | 0.4947 |
| ENSP00000356231 | RABIF | 0.4947 |
| ENSP00000175756 | PTPN18 | 0.4947 |
| ENSP00000309474 | PSMD1 | 0.4947 |
| ENSP00000378394 | PSAP | 0.4947 |
| ENSP00000254079 | PPP1R1B | 0.4947 |
| ENSP00000384496 | PPP1R12B | 0.4947 |
| ENSP00000263212 | PPM1F | 0.4947 |
| ENSP00000366828 | POLR3F | 0.4947 |
| ENSP00000361310 | POLH | 0.4947 |
| ENSP00000337701 | PNPLA2 | 0.4947 |
| ENSP00000369820 | PIGA | 0.4947 |
| ENSP00000305465 | PDILT | 0.4947 |
| ENSP00000385632 | PDE7A | 0.4947 |
| ENSP00000351957 | PDE3A | 0.4947 |
| ENSP00000438284 | PDE10A | 0.4947 |
| ENSP00000387911 | PARN | 0.4947 |
| ENSP00000219169 | NUTF2 | 0.4947 |
| ENSP00000251074 | NUP37 | 0.4947 |
| ENSP00000173229 | NTN1 | 0.4947 |
| ENSP00000359297 | NSDHL | 0.4947 |
| ENSP00000263317 | NOX4 | 0.4947 |
| ENSP00000243346 | NMI | 0.4947 |
| ENSP00000219479 | NME4 | 0.4947 |
| ENSP00000377696 | NIT2 | 0.4947 |
| ENSP00000362873 | NDUFA8 | 0.4947 |
| ENSP00000326806 | NCBP2 | 0.4947 |
| ENSP00000350467 | NAT9 | 0.4947 |

| | | |
|---|---|---|
| ENSP00000362617 | NAP1L6 | 0.4947 |
| ENSP00000320488 | NAP1L5 | 0.4947 |
| ENSP00000386213 | MYO3B | 0.4947 |
| ENSP00000421280 | MYO10 | 0.4947 |
| ENSP00000223364 | MYL7 | 0.4947 |
| ENSP00000354665 | MT-ND6 | 0.4947 |
| ENSP00000347324 | MSRB3 | 0.4947 |
| ENSP00000313921 | MSRA | 0.4947 |
| ENSP00000364320 | MRTO4 | 0.4947 |
| ENSP00000353643 | MORF4L2 | 0.4947 |
| ENSP00000257979 | MIP | 0.4947 |
| ENSP00000426103 | MIB2 | 0.4947 |
| ENSP00000302111 | MGMT | 0.4947 |
| ENSP00000219596 | MEFV | 0.4947 |
| ENSP00000377686 | MED24 | 0.4947 |
| ENSP00000354346 | MATR3 | 0.4947 |
| ENSP00000411397 | MARK3 | 0.4947 |
| ENSP00000228918 | LTBR | 0.4947 |
| ENSP00000368516 | LGR4 | 0.4947 |
| ENSP00000354526 | L3MBTL3 | 0.4947 |
| ENSP00000398516 | L3MBTL1 | 0.4947 |
| ENSP00000167586 | KRT14 | 0.4947 |
| ENSP00000261667 | KPNA3 | 0.4947 |
| ENSP00000368976 | KIFC3 | 0.4947 |
| ENSP00000301738 | KCTD5 | 0.4947 |
| ENSP00000325448 | KARS | 0.4947 |
| ENSP00000283249 | ITGB6 | 0.4947 |
| ENSP00000228534 | IL23A | 0.4947 |
| ENSP00000233813 | IGFBP5 | 0.4947 |
| ENSP00000360874 | IFIT1B | 0.4947 |
| ENSP00000261465 | HSD11B1 | 0.4947 |
| ENSP00000295470 | HNRPDL | 0.4947 |
| ENSP00000377492 | HMMR | 0.4947 |
| ENSP00000358414 | HMGCS2 | 0.4947 |
| ENSP00000295934 | HESX1 | 0.4947 |
| ENSP00000330721 | HDDC3 | 0.4947 |
| ENSP00000329002 | HAX1 | 0.4947 |
| ENSP00000316339 | HAO2 | 0.4947 |
| ENSP00000323811 | HACL1 | 0.4947 |
| ENSP00000348168 | GTF2E2 | 0.4947 |
| ENSP00000264954 | GRPEL1 | 0.4947 |
| ENSP00000264039 | GPC1 | 0.4947 |
| ENSP00000360021 | GNG12 | 0.4947 |
| ENSP00000356410 | GLRX2 | 0.4947 |
| ENSP00000387170 | GIGYF2 | 0.4947 |
| ENSP00000285873 | GEMIN5 | 0.4947 |
| ENSP00000297537 | GBX1 | 0.4947 |
| ENSP00000351605 | FZD6 | 0.4947 |
| ENSP00000349356 | FIGNL1 | 0.4947 |
| ENSP00000373952 | FANCA | 0.4947 |
| ENSP00000273854 | EPHA5 | 0.4947 |
| ENSP00000281821 | EPHA4 | 0.4947 |
| ENSP00000456711 | ENSG00000259753 | 0.4947 |

| | | |
|---|---|---|
| ENSP00000243878 | ENKD1 | 0.4947 |
| ENSP00000346693 | ELOVL2 | 0.4947 |
| ENSP00000320503 | EDC3 | 0.4947 |
| ENSP00000257192 | DSG1 | 0.4947 |
| ENSP00000401653 | DRGX | 0.4947 |
| ENSP00000176183 | DRD4 | 0.4947 |
| ENSP00000319651 | DMRTA1 | 0.4947 |
| ENSP00000349893 | DLK2 | 0.4947 |
| ENSP00000358563 | DKC1 | 0.4947 |
| ENSP00000392066 | DIP2A | 0.4947 |
| ENSP00000228027 | DGAT2 | 0.4947 |
| ENSP00000306117 | DDX19A | 0.4947 |
| ENSP00000217109 | CSTF1 | 0.4947 |
| ENSP00000262982 | CSE1L | 0.4947 |
| ENSP00000339353 | CPSF1 | 0.4947 |
| ENSP00000344562 | CORO6 | 0.4947 |
| ENSP00000454783 | CORO2B | 0.4947 |
| ENSP00000364000 | COL5A2 | 0.4947 |
| ENSP00000402460 | CLSTN2 | 0.4947 |
| ENSP00000363500 | CLIC4 | 0.4947 |
| ENSP00000326699 | CLGN | 0.4947 |
| ENSP00000388548 | CITED1 | 0.4947 |
| ENSP00000258930 | CIB2 | 0.4947 |
| ENSP00000265148 | CENPE | 0.4947 |
| ENSP00000301019 | CDT1 | 0.4947 |
| ENSP00000335357 | CDKN3 | 0.4947 |
| ENSP00000263645 | CD81 | 0.4947 |
| ENSP00000355361 | CD47 | 0.4947 |
| ENSP00000325708 | CD37 | 0.4947 |
| ENSP00000428982 | CCNC | 0.4947 |
| ENSP00000369689 | CALML5 | 0.4947 |
| ENSP00000360406 | CACNA1B | 0.4947 |
| ENSP00000256119 | CA1 | 0.4947 |
| ENSP00000373340 | BRPF1 | 0.4947 |
| ENSP00000274353 | BHMT | 0.4947 |
| ENSP00000219794 | BCKDK | 0.4947 |
| ENSP00000331210 | BCAN | 0.4947 |
| ENSP00000287322 | BAG4 | 0.4947 |
| ENSP00000319979 | B3GNTL1 | 0.4947 |
| ENSP00000253856 | ATP6V0A4 | 0.4947 |
| ENSP00000354490 | ATP1A2 | 0.4947 |
| ENSP00000265138 | ARRDC3 | 0.4947 |
| ENSP00000295087 | ARL5A | 0.4947 |
| ENSP00000308496 | ARL10 | 0.4947 |
| ENSP00000366121 | ARHGAP27 | 0.4947 |
| ENSP00000335578 | ARGFX | 0.4947 |
| ENSP00000308583 | APITD1 | 0.4947 |
| ENSP00000299402 | APBB1 | 0.4947 |
| ENSP00000257863 | AMHR2 | 0.4947 |
| ENSP00000290649 | AMFR | 0.4947 |
| ENSP00000258494 | ALDH1L2 | 0.4947 |
| ENSP00000235835 | AKR7A2 | 0.4947 |
| ENSP00000378359 | ADH6 | 0.4947 |

| | | |
|---|---|---|
| ENSP00000329468 | ADAP2 | 0.4947 |
| ENSP00000295137 | ACTG2 | 0.4947 |
| ENSP00000290378 | ACTC1 | 0.4947 |
| ENSP00000158762 | ACAP1 | 0.4947 |
| ENSP00000250615 | AANAT | 0.4947 |
| ENSP00000304593 | ZNHIT1 | 0.4921 |
| ENSP00000267807 | ZNF280D | 0.4921 |
| ENSP00000450742 | ZFYVE1 | 0.4921 |
| ENSP00000384716 | ZDHHC8 | 0.4921 |
| ENSP00000380675 | WEE2 | 0.4921 |
| ENSP00000468969 | WDR83OS | 0.4921 |
| ENSP00000387982 | WDR73 | 0.4921 |
| ENSP00000297873 | WBSCR27 | 0.4921 |
| ENSP00000377944 | UTP14A | 0.4921 |
| ENSP00000218348 | USP11 | 0.4921 |
| ENSP00000005226 | USH1C | 0.4921 |
| ENSP00000361289 | UCK1 | 0.4921 |
| ENSP00000360613 | UBE2A | 0.4921 |
| ENSP00000333266 | UBA7 | 0.4921 |
| ENSP00000256592 | TSHB | 0.4921 |
| ENSP00000283351 | TRAPPC8 | 0.4921 |
| ENSP00000362261 | TRAPPC3 | 0.4921 |
| ENSP00000300403 | TPX2 | 0.4921 |
| ENSP00000361900 | TOMM34 | 0.4921 |
| ENSP00000359928 | TNNI3K | 0.4921 |
| ENSP00000365435 | TNFRSF1B | 0.4921 |
| ENSP00000254616 | TIMM10B | 0.4921 |
| ENSP00000361158 | TESK2 | 0.4921 |
| ENSP00000364034 | TAP2 | 0.4921 |
| ENSP00000259075 | TANK | 0.4921 |
| ENSP00000286713 | STOM | 0.4921 |
| ENSP00000263373 | SPTBN4 | 0.4921 |
| ENSP00000363149 | SPDEF | 0.4921 |
| ENSP00000427463 | SPCS3 | 0.4921 |
| ENSP00000451145 | SOX7 | 0.4921 |
| ENSP00000251775 | SNX4 | 0.4921 |
| ENSP00000380336 | SMU1 | 0.4921 |
| ENSP00000323421 | SMC1A | 0.4921 |
| ENSP00000247138 | SLC35A2 | 0.4921 |
| ENSP00000252595 | SLC27A1 | 0.4921 |
| ENSP00000326305 | SLC25A20 | 0.4921 |
| ENSP00000225519 | SHPK | 0.4921 |
| ENSP00000371512 | SGCZ | 0.4921 |
| ENSP00000335321 | SF3B1 | 0.4921 |
| ENSP00000307599 | SCN11A | 0.4921 |
| ENSP00000252542 | SAFB2 | 0.4921 |
| ENSP00000246115 | S1PR4 | 0.4921 |
| ENSP00000317895 | RPTN | 0.4921 |
| ENSP00000309830 | RPL38 | 0.4921 |
| ENSP00000252543 | RPL36 | 0.4921 |
| ENSP00000401450 | RPL28 | 0.4921 |
| ENSP00000447001 | RPL18 | 0.4921 |
| ENSP00000467396 | RP11-886H22.1 | 0.4921 |

| | | |
|---|---|---|
| ENSP00000251507 | RABGAP1L | 0.4921 |
| ENSP00000308720 | PRSS1 | 0.4921 |
| ENSP00000349577 | PRODH | 0.4921 |
| ENSP00000368752 | PRNP | 0.4921 |
| ENSP00000264399 | PRKG2 | 0.4921 |
| ENSP00000276594 | PRDM14 | 0.4921 |
| ENSP00000292539 | PPP1R16A | 0.4921 |
| ENSP00000304169 | PITX2 | 0.4921 |
| ENSP00000288022 | PDF | 0.4921 |
| ENSP00000418194 | PARP14 | 0.4921 |
| ENSP00000238714 | PAPOLG | 0.4921 |
| ENSP00000262890 | PAFAH1B3 | 0.4921 |
| ENSP00000311665 | PAAF1 | 0.4921 |
| ENSP00000311713 | OXSR1 | 0.4921 |
| ENSP00000340836 | OSTF1 | 0.4921 |
| ENSP00000220514 | OIP5 | 0.4921 |
| ENSP00000361835 | NXF2B | 0.4921 |
| ENSP00000331471 | NXF2 | 0.4921 |
| ENSP00000263273 | NUCB1 | 0.4921 |
| ENSP00000283027 | NUBP1 | 0.4921 |
| ENSP00000371201 | NSUN7 | 0.4921 |
| ENSP00000418529 | NSFL1C | 0.4921 |
| ENSP00000265074 | NPR3 | 0.4921 |
| ENSP00000416658 | NME6 | 0.4921 |
| ENSP00000254940 | NIP7 | 0.4921 |
| ENSP00000362171 | NAP1L3 | 0.4921 |
| ENSP00000463645 | MYO15B | 0.4921 |
| ENSP00000274813 | MUT | 0.4921 |
| ENSP00000263629 | MTIF2 | 0.4921 |
| ENSP00000417871 | MSH5-SAPCD1 | 0.4921 |
| ENSP00000360498 | MRPL41 | 0.4921 |
| ENSP00000262794 | MOV10L1 | 0.4921 |
| ENSP00000310189 | MOB1B | 0.4921 |
| ENSP00000286317 | MED7 | 0.4921 |
| ENSP00000380888 | MED13 | 0.4921 |
| ENSP00000352852 | MDK | 0.4921 |
| ENSP00000365588 | MDC1 | 0.4921 |
| ENSP00000349640 | MCRS1 | 0.4921 |
| ENSP00000249016 | MCHR1 | 0.4921 |
| ENSP00000440374 | MCF2L | 0.4921 |
| ENSP00000265594 | MCCC1 | 0.4921 |
| ENSP00000356247 | LGR6 | 0.4921 |
| ENSP00000264005 | LCAT | 0.4921 |
| ENSP00000284898 | L3MBTL4 | 0.4921 |
| ENSP00000264170 | KYNU | 0.4921 |
| ENSP00000229214 | KRR1 | 0.4921 |
| ENSP00000265529 | KIF9 | 0.4921 |
| ENSP00000347409 | KEL | 0.4921 |
| ENSP00000385806 | KCNMA1 | 0.4921 |
| ENSP00000360133 | ITGB3BP | 0.4921 |
| ENSP00000282588 | ITGA1 | 0.4921 |
| ENSP00000360200 | INADL | 0.4921 |
| ENSP00000264538 | IFT57 | 0.4921 |

| | | |
|---|---|---|
| ENSP00000260570 | IFT172 | 0.4921 |
| ENSP00000306549 | HSFY2 | 0.4921 |
| ENSP00000254878 | HRSP12 | 0.4921 |
| ENSP00000441828 | HPR | 0.4921 |
| ENSP00000362566 | HPCA | 0.4921 |
| ENSP00000438468 | HHAT | 0.4921 |
| ENSP00000430620 | HEPH | 0.4921 |
| ENSP00000361219 | GTF3C4 | 0.4921 |
| ENSP00000379372 | GTF2A2 | 0.4921 |
| ENSP00000300177 | GREM1 | 0.4921 |
| ENSP00000264718 | GPN1 | 0.4921 |
| ENSP00000225724 | GOSR1 | 0.4921 |
| ENSP00000371594 | GNG7 | 0.4921 |
| ENSP00000414019 | GDPD2 | 0.4921 |
| ENSP00000247005 | GDF1 | 0.4921 |
| ENSP00000366482 | FXN | 0.4921 |
| ENSP00000328720 | FOXK1 | 0.4921 |
| ENSP00000222308 | FKBP8 | 0.4921 |
| ENSP00000354691 | FCRL5 | 0.4921 |
| ENSP00000357167 | FCRL3 | 0.4921 |
| ENSP00000313034 | FBXW5 | 0.4921 |
| ENSP00000346240 | FBXO2 | 0.4921 |
| ENSP00000330098 | FBXL6 | 0.4921 |
| ENSP00000445905 | EVI5L | 0.4921 |
| ENSP00000310520 | ERCC4 | 0.4921 |
| ENSP00000347213 | ENTPD2 | 0.4921 |
| ENSP00000361536 | ELOVL1 | 0.4921 |
| ENSP00000369460 | ELAVL2 | 0.4921 |
| ENSP00000328777 | EFNA5 | 0.4921 |
| ENSP00000436585 | ECHDC1 | 0.4921 |
| ENSP00000349679 | DYNLRB1 | 0.4921 |
| ENSP00000260605 | DYNC2LI1 | 0.4921 |
| ENSP00000227451 | DTX4 | 0.4921 |
| ENSP00000250937 | DOHH | 0.4921 |
| ENSP00000262442 | DNAH9 | 0.4921 |
| ENSP00000205402 | DLD | 0.4921 |
| ENSP00000346236 | DDX46 | 0.4921 |
| ENSP00000384703 | DDX11 | 0.4921 |
| ENSP00000302805 | DDI1 | 0.4921 |
| ENSP00000349768 | DCHS2 | 0.4921 |
| ENSP00000221700 | CYP4F2 | 0.4921 |
| ENSP00000273062 | CTDSP1 | 0.4921 |
| ENSP00000299543 | CTDP1 | 0.4921 |
| ENSP00000327647 | CRADD | 0.4921 |
| ENSP00000219150 | CORO1A | 0.4921 |
| ENSP00000262812 | COPE | 0.4921 |
| ENSP00000396755 | CNTD2 | 0.4921 |
| ENSP00000378505 | CLK3 | 0.4921 |
| ENSP00000296130 | CLEC3B | 0.4921 |
| ENSP00000216492 | CHGA | 0.4921 |
| ENSP00000416561 | CFB | 0.4921 |
| ENSP00000219172 | CENPT | 0.4921 |
| ENSP00000260662 | CENPO | 0.4921 |

| | | |
|---|---|---|
| ENSP00000355731 | CDC42BPA | 0.4921 |
| ENSP00000311984 | CARKD | 0.4921 |
| ENSP00000263168 | CAPZA1 | 0.4921 |
| ENSP00000269881 | CALR3 | 0.4921 |
| ENSP00000356652 | CACYBP | 0.4921 |
| ENSP00000270458 | CACNG8 | 0.4921 |
| ENSP00000377840 | CACNB1 | 0.4921 |
| ENSP00000443246 | BCKDHA | 0.4921 |
| ENSP00000357475 | ATP8B2 | 0.4921 |
| ENSP00000290299 | ATP5O | 0.4921 |
| ENSP00000348205 | ATP5G1 | 0.4921 |
| ENSP00000215375 | ATP5D | 0.4921 |
| ENSP00000263382 | ASF1B | 0.4921 |
| ENSP00000419199 | ASB14 | 0.4921 |
| ENSP00000391751 | ARL17B | 0.4921 |
| ENSP00000263620 | ARID3A | 0.4921 |
| ENSP00000379709 | ARHGAP21 | 0.4921 |
| ENSP00000386502 | ANKRD61 | 0.4921 |
| ENSP00000377051 | ANKRD42 | 0.4921 |
| ENSP00000304292 | ANKRD27 | 0.4921 |
| ENSP00000265512 | ADH4 | 0.4921 |
| ENSP00000320646 | ACSF3 | 0.4921 |
| ENSP00000216139 | ACR | 0.4921 |
| ENSP00000354995 | ABCG1 | 0.4921 |
| ENSP00000360215 | ZBP1 | 0.4895 |
| ENSP00000343435 | YIF1B | 0.4895 |
| ENSP00000352271 | XRCC2 | 0.4895 |
| ENSP00000353793 | WDHD1 | 0.4895 |
| ENSP00000417185 | VWA1 | 0.4895 |
| ENSP00000267202 | VPS37B | 0.4895 |
| ENSP00000327246 | VIPR1 | 0.4895 |
| ENSP00000308332 | UTP23 | 0.4895 |
| ENSP00000285199 | USP43 | 0.4895 |
| ENSP00000003302 | USP28 | 0.4895 |
| ENSP00000305941 | USH2A | 0.4895 |
| ENSP00000260323 | UNC13C | 0.4895 |
| ENSP00000311648 | UGT8 | 0.4895 |
| ENSP00000263202 | UFD1L | 0.4895 |
| ENSP00000379931 | UBE2E2 | 0.4895 |
| ENSP00000252029 | TYMP | 0.4895 |
| ENSP00000266254 | TTLL1 | 0.4895 |
| ENSP00000261647 | TTC19 | 0.4895 |
| ENSP00000262294 | TRIM37 | 0.4895 |
| ENSP00000357750 | TRAF3IP2 | 0.4895 |
| ENSP00000297071 | TRA2A | 0.4895 |
| ENSP00000344215 | TP53INP1 | 0.4895 |
| ENSP00000238721 | TP53I3 | 0.4895 |
| ENSP00000337022 | TNNI1 | 0.4895 |
| ENSP00000303476 | TLN2 | 0.4895 |
| ENSP00000352926 | TEAD4 | 0.4895 |
| ENSP00000300584 | TBC1D2B | 0.4895 |
| ENSP00000245934 | SYMPK | 0.4895 |
| ENSP00000340279 | SSX2IP | 0.4895 |

| | | |
|---|---|---|
| ENSP00000311609 | SPSB4 | 0.4895 |
| ENSP00000363391 | SPAG4 | 0.4895 |
| ENSP00000320246 | SLC9A9 | 0.4895 |
| ENSP00000354966 | SLC9A8 | 0.4895 |
| ENSP00000370128 | SLC7A1 | 0.4895 |
| ENSP00000370192 | SLC46A3 | 0.4895 |
| ENSP00000377401 | SLC35B2 | 0.4895 |
| ENSP00000323568 | SLC2A2 | 0.4895 |
| ENSP00000267842 | SLC27A2 | 0.4895 |
| ENSP00000200652 | SLC22A4 | 0.4895 |
| ENSP00000254722 | SERPINF1 | 0.4895 |
| ENSP00000331692 | SCRT1 | 0.4895 |
| ENSP00000262288 | SCPEP1 | 0.4895 |
| ENSP00000261693 | SCARB1 | 0.4895 |
| ENSP00000466933 | S1PR2 | 0.4895 |
| ENSP00000198767 | RRN3 | 0.4895 |
| ENSP00000348849 | RPS26 | 0.4895 |
| ENSP00000465737 | RP11-729L2.2 | 0.4895 |
| ENSP00000309431 | RP11-683L23.1 | 0.4895 |
| ENSP00000373772 | RNF20 | 0.4895 |
| ENSP00000358297 | RNF115 | 0.4895 |
| ENSP00000370750 | RDH11 | 0.4895 |
| ENSP00000252483 | PVRL2 | 0.4895 |
| ENSP00000351342 | PTPN5 | 0.4895 |
| ENSP00000341170 | PTN | 0.4895 |
| ENSP00000157812 | PSMC4 | 0.4895 |
| ENSP00000365773 | PSAT1 | 0.4895 |
| ENSP00000347906 | PRMT2 | 0.4895 |
| ENSP00000263433 | PPP1R12C | 0.4895 |
| ENSP00000253925 | PPFIA1 | 0.4895 |
| ENSP00000367029 | POLR1E | 0.4895 |
| ENSP00000386105 | POLD2 | 0.4895 |
| ENSP00000388631 | PLCD4 | 0.4895 |
| ENSP00000265382 | PIP5K1B | 0.4895 |
| ENSP00000252603 | PGLS | 0.4895 |
| ENSP00000359991 | PGAM1 | 0.4895 |
| ENSP00000369100 | PFKFB3 | 0.4895 |
| ENSP00000261813 | PFDN1 | 0.4895 |
| ENSP00000419975 | PEX5L | 0.4895 |
| ENSP00000419027 | PCCB | 0.4895 |
| ENSP00000234040 | PASK | 0.4895 |
| ENSP00000271452 | NUF2 | 0.4895 |
| ENSP00000376349 | NUDT14 | 0.4895 |
| ENSP00000263257 | NOVA2 | 0.4895 |
| ENSP00000354541 | NLGN1 | 0.4895 |
| ENSP00000322087 | NHLH2 | 0.4895 |
| ENSP00000409936 | MYO19 | 0.4895 |
| ENSP00000273353 | MYH15 | 0.4895 |
| ENSP00000370968 | MYBBP1A | 0.4895 |
| ENSP00000333401 | MRPL40 | 0.4895 |
| ENSP00000315702 | MOB4 | 0.4895 |
| ENSP00000328251 | MINA | 0.4895 |
| ENSP00000366939 | MGME1 | 0.4895 |

| | | |
|---|---|---|
| ENSP00000219905 | MGA | 0.4895 |
| ENSP00000352657 | ME3 | 0.4895 |
| ENSP00000262891 | MARK4 | 0.4895 |
| ENSP00000364813 | LSM2 | 0.4895 |
| ENSP00000359990 | LRRC40 | 0.4895 |
| ENSP00000280706 | LDHAL6A | 0.4895 |
| ENSP00000310551 | LCLAT1 | 0.4895 |
| ENSP00000370671 | KLHL42 | 0.4895 |
| ENSP00000232766 | KLHL18 | 0.4895 |
| ENSP00000346401 | KIF25 | 0.4895 |
| ENSP00000295101 | KCNJ3 | 0.4895 |
| ENSP00000222573 | ITGB8 | 0.4895 |
| ENSP00000380227 | ITGA4 | 0.4895 |
| ENSP00000387347 | IQCA1 | 0.4895 |
| ENSP00000274520 | IL9 | 0.4895 |
| ENSP00000375795 | IL24 | 0.4895 |
| ENSP00000406012 | IFT140 | 0.4895 |
| ENSP00000360860 | IFIT5 | 0.4895 |
| ENSP00000362352 | H2AFY2 | 0.4895 |
| ENSP00000248935 | GSTT1 | 0.4895 |
| ENSP00000257248 | GIF | 0.4895 |
| ENSP00000257868 | GDF11 | 0.4895 |
| ENSP00000421725 | GC | 0.4895 |
| ENSP00000272252 | GALM | 0.4895 |
| ENSP00000259271 | GAD2 | 0.4895 |
| ENSP00000229030 | FZD10 | 0.4895 |
| ENSP00000287934 | FZD1 | 0.4895 |
| ENSP00000418823 | FTO | 0.4895 |
| ENSP00000242209 | FKBP9 | 0.4895 |
| ENSP00000333097 | FIGLA | 0.4895 |
| ENSP00000358165 | FCGR1A | 0.4895 |
| ENSP00000310332 | FBXO45 | 0.4895 |
| ENSP00000281828 | FARSB | 0.4895 |
| ENSP00000264042 | FARP2 | 0.4895 |
| ENSP00000308541 | F2 | 0.4895 |
| ENSP00000257934 | ESPL1 | 0.4895 |
| ENSP00000288221 | ERC2 | 0.4895 |
| ENSP00000248342 | EIF3K | 0.4895 |
| ENSP00000417229 | EIF2A | 0.4895 |
| ENSP00000312224 | DTD2 | 0.4895 |
| ENSP00000261590 | DSG2 | 0.4895 |
| ENSP00000404179 | DOCK4 | 0.4895 |
| ENSP00000276570 | DNAJC5B | 0.4895 |
| ENSP00000368565 | DNAJC2 | 0.4895 |
| ENSP00000220496 | DNAJC17 | 0.4895 |
| ENSP00000265028 | DNAJB11 | 0.4895 |
| ENSP00000314030 | DNAJA2 | 0.4895 |
| ENSP00000255189 | DMGDH | 0.4895 |
| ENSP00000249749 | DLL4 | 0.4895 |
| ENSP00000263576 | DDX25 | 0.4895 |
| ENSP00000288071 | DDX19B | 0.4895 |
| ENSP00000356218 | CYB5R1 | 0.4895 |
| ENSP00000264474 | CSTA | 0.4895 |

| | | |
|---|---|---|
| ENSP00000227251 | CRYAB | 0.4895 |
| ENSP00000312189 | CPT1B | 0.4895 |
| ENSP00000320672 | COX6B2 | 0.4895 |
| ENSP00000340211 | CORO1B | 0.4895 |
| ENSP00000268717 | COPS3 | 0.4895 |
| ENSP00000273308 | CNPY2 | 0.4895 |
| ENSP00000349147 | CNNM1 | 0.4895 |
| ENSP00000337056 | CDKN2D | 0.4895 |
| ENSP00000327191 | CCT6B | 0.4895 |
| ENSP00000344460 | CBS | 0.4895 |
| ENSP00000372169 | CACNA2D4 | 0.4895 |
| ENSP00000288139 | CACNA1D | 0.4895 |
| ENSP00000312054 | C7orf10 | 0.4895 |
| ENSP00000347197 | C5AR1 | 0.4895 |
| ENSP00000256319 | C14orf1 | 0.4895 |
| ENSP00000179259 | C12orf5 | 0.4895 |
| ENSP00000331369 | BOLA3 | 0.4895 |
| ENSP00000259407 | BAAT | 0.4895 |
| ENSP00000381590 | ATXN7 | 0.4895 |
| ENSP00000264649 | ATP6V0A1 | 0.4895 |
| ENSP00000229595 | ASF1A | 0.4895 |
| ENSP00000269586 | ARL5C | 0.4895 |
| ENSP00000337478 | ARL17A | 0.4895 |
| ENSP00000282026 | ARL11 | 0.4895 |
| ENSP00000374423 | ARHGAP8 | 0.4895 |
| ENSP00000260283 | ARHGAP20 | 0.4895 |
| ENSP00000255040 | APCS | 0.4895 |
| ENSP00000306185 | ANTXR2 | 0.4895 |
| ENSP00000378260 | ALG13 | 0.4895 |
| ENSP00000293350 | ALDH16A1 | 0.4895 |
| ENSP00000370129 | AKR1C2 | 0.4895 |
| ENSP00000223029 | AIMP2 | 0.4895 |
| ENSP00000407436 | AGAP10 | 0.4895 |
| ENSP00000420269 | ADH7 | 0.4895 |
| ENSP00000413074 | ADAL | 0.4895 |
| ENSP00000373574 | ACVRL1 | 0.4895 |
| ENSP00000290866 | ACE | 0.4895 |
| ENSP00000304111 | ABCG4 | 0.4895 |
| ENSP00000304308 | ZNF217 | 0.4868 |
| ENSP00000368727 | XDH | 0.4868 |
| ENSP00000334186 | VWCE | 0.4868 |
| ENSP00000358207 | VAX1 | 0.4868 |
| ENSP00000236192 | VAMP4 | 0.4868 |
| ENSP00000261497 | USP22 | 0.4868 |
| ENSP00000357775 | UROS | 0.4868 |
| ENSP00000314480 | UNCX | 0.4868 |
| ENSP00000262999 | UCP1 | 0.4868 |
| ENSP00000360116 | UBE2U | 0.4868 |
| ENSP00000358323 | TXNIP | 0.4868 |
| ENSP00000264255 | TXNDC9 | 0.4868 |
| ENSP00000318197 | TUBA3E | 0.4868 |
| ENSP00000326042 | TUBA3D | 0.4868 |
| ENSP00000260116 | TTPA | 0.4868 |

| | | |
|---|---|---|
| ENSP00000323889 | TRIM25 | 0.4868 |
| ENSP00000436005 | TRAPPC4 | 0.4868 |
| ENSP00000266712 | TMTC3 | 0.4868 |
| ENSP00000335416 | TMEM68 | 0.4868 |
| ENSP00000454670 | TMBIM4 | 0.4868 |
| ENSP00000356256 | TIMM17A | 0.4868 |
| ENSP00000357362 | THBS3 | 0.4868 |
| ENSP00000273368 | TAGLN3 | 0.4868 |
| ENSP00000263846 | SYT7 | 0.4868 |
| ENSP00000247001 | SUGP1 | 0.4868 |
| ENSP00000339435 | SRR | 0.4868 |
| ENSP00000349677 | SPOCK3 | 0.4868 |
| ENSP00000365811 | SPAG6 | 0.4868 |
| ENSP00000251809 | SPAG1 | 0.4868 |
| ENSP00000301466 | SOAT2 | 0.4868 |
| ENSP00000403310 | SNRNP35 | 0.4868 |
| ENSP00000393453 | SMYD1 | 0.4868 |
| ENSP00000357650 | SLC27A3 | 0.4868 |
| ENSP00000398930 | SGCE | 0.4868 |
| ENSP00000341117 | SDSL | 0.4868 |
| ENSP00000272091 | SDE2 | 0.4868 |
| ENSP00000390600 | SCN10A | 0.4868 |
| ENSP00000373884 | RYR3 | 0.4868 |
| ENSP00000007264 | RPUSD1 | 0.4868 |
| ENSP00000265100 | RPL26L1 | 0.4868 |
| ENSP00000456318 | RP11-234B24.6 | 0.4868 |
| ENSP00000381214 | RGPD2 | 0.4868 |
| ENSP00000381253 | RGPD1 | 0.4868 |
| ENSP00000170168 | REXO1 | 0.4868 |
| ENSP00000336606 | RAD54B | 0.4868 |
| ENSP00000326003 | PUS10 | 0.4868 |
| ENSP00000327704 | PSMF1 | 0.4868 |
| ENSP00000363939 | PPP3R2 | 0.4868 |
| ENSP00000261207 | PPP1R12A | 0.4868 |
| ENSP00000370222 | POMP | 0.4868 |
| ENSP00000396045 | PLA2G4B | 0.4868 |
| ENSP00000374409 | PKP4 | 0.4868 |
| ENSP00000348611 | PHLPP2 | 0.4868 |
| ENSP00000455607 | PHKG2 | 0.4868 |
| ENSP00000300056 | PEX11A | 0.4868 |
| ENSP00000263985 | PET112 | 0.4868 |
| ENSP00000397157 | PERP | 0.4868 |
| ENSP00000319814 | PCK1 | 0.4868 |
| ENSP00000384048 | PAXIP1 | 0.4868 |
| ENSP00000263650 | OSBPL5 | 0.4868 |
| ENSP00000380544 | OGFOD2 | 0.4868 |
| ENSP00000359532 | NTSR1 | 0.4868 |
| ENSP00000447149 | NR1H4 | 0.4868 |
| ENSP00000322450 | NDUFV1 | 0.4868 |
| ENSP00000379430 | NDUFAF6 | 0.4868 |
| ENSP00000252575 | NCAN | 0.4868 |
| ENSP00000261182 | NAP1L1 | 0.4868 |
| ENSP00000290810 | NAE1 | 0.4868 |

| | | |
|---|---|---|
| ENSP00000252172 | MYH13 | 0.4868 |
| ENSP00000265052 | MGLL | 0.4868 |
| ENSP00000362166 | MEAF6 | 0.4868 |
| ENSP00000290429 | MCAT | 0.4868 |
| ENSP00000309141 | MARCH3 | 0.4868 |
| ENSP00000293872 | LUC7L | 0.4868 |
| ENSP00000376333 | LLGL2 | 0.4868 |
| ENSP00000373487 | KRT18 | 0.4868 |
| ENSP00000308452 | KRT17 | 0.4868 |
| ENSP00000352138 | KIRREL | 0.4868 |
| ENSP00000301332 | KIFC2 | 0.4868 |
| ENSP00000320821 | KIF1C | 0.4868 |
| ENSP00000007722 | ITGA3 | 0.4868 |
| ENSP00000452007 | ISCA2 | 0.4868 |
| ENSP00000310260 | INTS6 | 0.4868 |
| ENSP00000413937 | INPP5K | 0.4868 |
| ENSP00000384534 | INPP5J | 0.4868 |
| ENSP00000168216 | HSD17B10 | 0.4868 |
| ENSP00000265983 | HPX | 0.4868 |
| ENSP00000334382 | HOMER1 | 0.4868 |
| ENSP00000339398 | HLA-DQA1 | 0.4868 |
| ENSP00000267845 | HDC | 0.4868 |
| ENSP00000380514 | HAGH | 0.4868 |
| ENSP00000351644 | GTPBP3 | 0.4868 |
| ENSP00000350348 | GRM7 | 0.4868 |
| ENSP00000231188 | GRM6 | 0.4868 |
| ENSP00000285900 | GRIA1 | 0.4868 |
| ENSP00000303077 | GOT1L1 | 0.4868 |
| ENSP00000318821 | GCC1 | 0.4868 |
| ENSP00000363826 | FZD8 | 0.4868 |
| ENSP00000286201 | FZD7 | 0.4868 |
| ENSP00000346901 | FMO1 | 0.4868 |
| ENSP00000357789 | FLG | 0.4868 |
| ENSP00000333836 | FIGN | 0.4868 |
| ENSP00000344393 | FCF1 | 0.4868 |
| ENSP00000345008 | FBLN5 | 0.4868 |
| ENSP00000261800 | FAT2 | 0.4868 |
| ENSP00000452762 | ETFA | 0.4868 |
| ENSP00000266397 | ERP27 | 0.4868 |
| ENSP00000256398 | ELP3 | 0.4868 |
| ENSP00000364687 | EHMT2 | 0.4868 |
| ENSP00000263991 | EHBP1 | 0.4868 |
| ENSP00000263083 | DPH1 | 0.4868 |
| ENSP00000280346 | DLAT | 0.4868 |
| ENSP00000311135 | DHX37 | 0.4868 |
| ENSP00000367527 | DCLRE1C | 0.4868 |
| ENSP00000227256 | DBX1 | 0.4868 |
| ENSP00000371870 | DAZ2 | 0.4868 |
| ENSP00000259371 | DAB2IP | 0.4868 |
| ENSP00000268053 | CYP11A1 | 0.4868 |
| ENSP00000267085 | CSAD | 0.4868 |
| ENSP00000366557 | CRNKL1 | 0.4868 |
| ENSP00000365243 | COX4I2 | 0.4868 |

| | | |
|---|---|---|
| ENSP00000016171 | COX15 | 0.4868 |
| ENSP00000354778 | CNTNAP2 | 0.4868 |
| ENSP00000366513 | CLSTN1 | 0.4868 |
| ENSP00000442057 | CINP | 0.4868 |
| ENSP00000377747 | CHD2 | 0.4868 |
| ENSP00000337103 | CHAT | 0.4868 |
| ENSP00000315700 | CHAF1B | 0.4868 |
| ENSP00000309629 | CFL1 | 0.4868 |
| ENSP00000377007 | CENPN | 0.4868 |
| ENSP00000242872 | CENPK | 0.4868 |
| ENSP00000262738 | CELSR1 | 0.4868 |
| ENSP00000237654 | CCNI | 0.4868 |
| ENSP00000331636 | CAPN12 | 0.4868 |
| ENSP00000265431 | CALB1 | 0.4868 |
| ENSP00000320025 | CACNB2 | 0.4868 |
| ENSP00000363135 | C6orf106 | 0.4868 |
| ENSP00000282701 | BMP3 | 0.4868 |
| ENSP00000359727 | BAG2 | 0.4868 |
| ENSP00000284509 | ATP8B4 | 0.4868 |
| ENSP00000379203 | ATP6V1C1 | 0.4868 |
| ENSP00000341942 | ATP13A5 | 0.4868 |
| ENSP00000345808 | ARHGAP12 | 0.4868 |
| ENSP00000219919 | AQP9 | 0.4868 |
| ENSP00000252486 | APOE | 0.4868 |
| ENSP00000445688 | ANKRD65 | 0.4868 |
| ENSP00000351416 | ANKRD17 | 0.4868 |
| ENSP00000471417 | ANKDD1B | 0.4868 |
| ENSP00000380793 | ALG3 | 0.4868 |
| ENSP00000387123 | ALDH7A1 | 0.4868 |
| ENSP00000273430 | AGTR1 | 0.4868 |
| ENSP00000362287 | AGO3 | 0.4868 |
| ENSP00000362888 | ADO | 0.4868 |
| ENSP00000284984 | ADAMTS1 | 0.4868 |
| ENSP00000289416 | ACSM3 | 0.4868 |
| ENSP00000386456 | AAK1 | 0.4868 |
| ENSP00000415398 | ENSG00000236954 | 0.4868 |
| ENSP00000302855 | ZNF280A | 0.4842 |
| ENSP00000441410 | ZNF135 | 0.4842 |
| ENSP00000265069 | ZFR | 0.4842 |
| ENSP00000261749 | ZFAND6 | 0.4842 |
| ENSP00000314619 | ZFAND2A | 0.4842 |
| ENSP00000342011 | XRCC4 | 0.4842 |
| ENSP00000384290 | WDR60 | 0.4842 |
| ENSP00000314444 | WDR35 | 0.4842 |
| ENSP00000358126 | VPS45 | 0.4842 |
| ENSP00000327650 | VPS33B | 0.4842 |
| ENSP00000351346 | VPS13B | 0.4842 |
| ENSP00000248444 | VIL1 | 0.4842 |
| ENSP00000260187 | USP2 | 0.4842 |
| ENSP00000330032 | UPP1 | 0.4842 |
| ENSP00000420270 | UHMK1 | 0.4842 |
| ENSP00000265339 | UBE2B | 0.4842 |
| ENSP00000217515 | TXNL1 | 0.4842 |

| | | |
|---|---|---|
| ENSP00000417919 | TSPYL6 | 0.4842 |
| ENSP00000357597 | TSPYL1 | 0.4842 |
| ENSP00000363390 | TRIM63 | 0.4842 |
| ENSP00000392495 | TRAPPC2 | 0.4842 |
| ENSP00000407182 | TNPO2 | 0.4842 |
| ENSP00000260867 | TIMM23 | 0.4842 |
| ENSP00000318502 | TGIF2LY | 0.4842 |
| ENSP00000338127 | TESK1 | 0.4842 |
| ENSP00000454836 | TBL3 | 0.4842 |
| ENSP00000343610 | SYNRG | 0.4842 |
| ENSP00000266971 | SUOX | 0.4842 |
| ENSP00000318635 | SUMO4 | 0.4842 |
| ENSP00000259400 | STX17 | 0.4842 |
| ENSP00000466298 | STX10 | 0.4842 |
| ENSP00000300737 | STIM1 | 0.4842 |
| ENSP00000216484 | SPTLC2 | 0.4842 |
| ENSP00000317790 | SPTBN5 | 0.4842 |
| ENSP00000217381 | SNTA1 | 0.4842 |
| ENSP00000270349 | SLC6A3 | 0.4842 |
| ENSP00000298472 | SLC18A2 | 0.4842 |
| ENSP00000202967 | SIRT4 | 0.4842 |
| ENSP00000366936 | SHB | 0.4842 |
| ENSP00000415332 | SESTD1 | 0.4842 |
| ENSP00000402527 | SENP6 | 0.4842 |
| ENSP00000300175 | SCG5 | 0.4842 |
| ENSP00000356146 | SCAF8 | 0.4842 |
| ENSP00000262584 | RPL8 | 0.4842 |
| ENSP00000361076 | RPL7A | 0.4842 |
| ENSP00000454454 | RP11-512M8.5 | 0.4842 |
| ENSP00000304051 | RNF139 | 0.4842 |
| ENSP00000422846 | RNASET2 | 0.4842 |
| ENSP00000331871 | RHD | 0.4842 |
| ENSP00000259406 | RGS3 | 0.4842 |
| ENSP00000297313 | RGS20 | 0.4842 |
| ENSP00000330842 | RGPD6 | 0.4842 |
| ENSP00000368664 | RGCC | 0.4842 |
| ENSP00000254325 | RFX1 | 0.4842 |
| ENSP00000226193 | RCVRN | 0.4842 |
| ENSP00000386226 | RBM34 | 0.4842 |
| ENSP00000352956 | RBM23 | 0.4842 |
| ENSP00000327583 | RANBP1 | 0.4842 |
| ENSP00000353198 | PYY | 0.4842 |
| ENSP00000418661 | PTRH1 | 0.4842 |
| ENSP00000334928 | PTPRB | 0.4842 |
| ENSP00000450995 | PTCD1 | 0.4842 |
| ENSP00000339764 | PRDM8 | 0.4842 |
| ENSP00000261475 | PPP2R3C | 0.4842 |
| ENSP00000375080 | PPP2R3B | 0.4842 |
| ENSP00000361642 | PPCS | 0.4842 |
| ENSP00000296028 | PPBP | 0.4842 |
| ENSP00000350249 | POT1 | 0.4842 |
| ENSP00000363284 | POLE3 | 0.4842 |
| ENSP00000216177 | PNPLA5 | 0.4842 |

| | | |
|---|---|---|
| ENSP00000424417 | PLRG1 | 0.4842 |
| ENSP00000333142 | PLA2G6 | 0.4842 |
| ENSP00000313504 | PHKB | 0.4842 |
| ENSP00000218343 | PHF16 | 0.4842 |
| ENSP00000251757 | PHC1 | 0.4842 |
| ENSP00000297283 | PGAM2 | 0.4842 |
| ENSP00000387536 | PDK3 | 0.4842 |
| ENSP00000251630 | PDGFRL | 0.4842 |
| ENSP00000368439 | PCYT1B | 0.4842 |
| ENSP00000356348 | PCMT1 | 0.4842 |
| ENSP00000300146 | PATL1 | 0.4842 |
| ENSP00000353512 | PARP9 | 0.4842 |
| ENSP00000343144 | PARD6G | 0.4842 |
| ENSP00000296783 | PAPD4 | 0.4842 |
| ENSP00000310305 | P2RY2 | 0.4842 |
| ENSP00000360270 | OMA1 | 0.4842 |
| ENSP00000319664 | NUDC | 0.4842 |
| ENSP00000319788 | NQO1 | 0.4842 |
| ENSP00000365663 | NPPA | 0.4842 |
| ENSP00000405290 | NPAS1 | 0.4842 |
| ENSP00000287139 | NODAL | 0.4842 |
| ENSP00000383210 | NEK3 | 0.4842 |
| ENSP00000327268 | NDUFV2 | 0.4842 |
| ENSP00000315774 | NDUFS8 | 0.4842 |
| ENSP00000290231 | NCOA5 | 0.4842 |
| ENSP00000379680 | NAGA | 0.4842 |
| ENSP00000261435 | N4BP2 | 0.4842 |
| ENSP00000302811 | MTNR1A | 0.4842 |
| ENSP00000354728 | MT-ND4L | 0.4842 |
| ENSP00000379108 | MTHFD2L | 0.4842 |
| ENSP00000295491 | MRPS18C | 0.4842 |
| ENSP00000229238 | MRPL51 | 0.4842 |
| ENSP00000312311 | MRPL46 | 0.4842 |
| ENSP00000260102 | MRPL15 | 0.4842 |
| ENSP00000372445 | METTL17 | 0.4842 |
| ENSP00000384369 | METTL15 | 0.4842 |
| ENSP00000290663 | MED8 | 0.4842 |
| ENSP00000256379 | MED6 | 0.4842 |
| ENSP00000291688 | MCM3AP | 0.4842 |
| ENSP00000244217 | MCEE | 0.4842 |
| ENSP00000156825 | MBD3 | 0.4842 |
| ENSP00000326817 | LRRC57 | 0.4842 |
| ENSP00000366886 | KLHL21 | 0.4842 |
| ENSP00000356319 | KIF14 | 0.4842 |
| ENSP00000309501 | KIAA1239 | 0.4842 |
| ENSP00000305824 | KCNS3 | 0.4842 |
| ENSP00000385478 | ISPD | 0.4842 |
| ENSP00000327889 | INTS5 | 0.4842 |
| ENSP00000344192 | IL17A | 0.4842 |
| ENSP00000392985 | HTATIP2 | 0.4842 |
| ENSP00000358421 | HSD3B1 | 0.4842 |
| ENSP00000336856 | HMG20A | 0.4842 |
| ENSP00000299163 | HIF1AN | 0.4842 |

| | | |
|---|---|---|
| ENSP00000265395 | HIBADH | 0.4842 |
| ENSP00000261047 | GUCA1C | 0.4842 |
| ENSP00000216951 | GSS | 0.4842 |
| ENSP00000324693 | GRHL1 | 0.4842 |
| ENSP00000346103 | GPX4 | 0.4842 |
| ENSP00000355146 | GPRASP1 | 0.4842 |
| ENSP00000383382 | GP1BB | 0.4842 |
| ENSP00000354900 | GJB1 | 0.4842 |
| ENSP00000401018 | GINS3 | 0.4842 |
| ENSP00000320815 | GDPD4 | 0.4842 |
| ENSP00000288985 | ERCC6L2 | 0.4842 |
| ENSP00000259486 | ENPP2 | 0.4842 |
| ENSP00000295066 | DPY30 | 0.4842 |
| ENSP00000347716 | DPF1 | 0.4842 |
| ENSP00000365007 | DNAJC16 | 0.4842 |
| ENSP00000260818 | DNAJC13 | 0.4842 |
| ENSP00000373825 | DNAH2 | 0.4842 |
| ENSP00000327975 | DHRS7C | 0.4842 |
| ENSP00000328036 | DGAT2L6 | 0.4842 |
| ENSP00000306528 | DESI2 | 0.4842 |
| ENSP00000307126 | DEGS2 | 0.4842 |
| ENSP00000263579 | DCPS | 0.4842 |
| ENSP00000423602 | DAPP1 | 0.4842 |
| ENSP00000221307 | CYP4F3 | 0.4842 |
| ENSP00000261726 | CUX2 | 0.4842 |
| ENSP00000350911 | CTSE | 0.4842 |
| ENSP00000368140 | CRLS1 | 0.4842 |
| ENSP00000315013 | CRAT | 0.4842 |
| ENSP00000359603 | COL24A1 | 0.4842 |
| ENSP00000375069 | COL23A1 | 0.4842 |
| ENSP00000310003 | CNIH2 | 0.4842 |
| ENSP00000471505 | CKS1B | 0.4842 |
| ENSP00000221476 | CKM | 0.4842 |
| ENSP00000333873 | CIB1 | 0.4842 |
| ENSP00000283122 | CETN3 | 0.4842 |
| ENSP00000251108 | CDADC1 | 0.4842 |
| ENSP00000304236 | CD14 | 0.4842 |
| ENSP00000225603 | CBX1 | 0.4842 |
| ENSP00000352561 | CALCR | 0.4842 |
| ENSP00000295379 | BMP10 | 0.4842 |
| ENSP00000347232 | BLM | 0.4842 |
| ENSP00000318351 | BCKDHB | 0.4842 |
| ENSP00000358554 | BCAS2 | 0.4842 |
| ENSP00000264499 | BBS7 | 0.4842 |
| ENSP00000292095 | BACE1 | 0.4842 |
| ENSP00000306410 | ARMC4 | 0.4842 |
| ENSP00000377769 | ARL13B | 0.4842 |
| ENSP00000320038 | ARHGAP33 | 0.4842 |
| ENSP00000156471 | AQR | 0.4842 |
| ENSP00000297988 | AQP7 | 0.4842 |
| ENSP00000301455 | ANGPTL4 | 0.4842 |
| ENSP00000360149 | ALG6 | 0.4842 |
| ENSP00000340009 | ALG1L | 0.4842 |

| | | |
|---|---|---|
| ENSP00000470087 | AC006486.9 | 0.4842 |
| ENSP00000400587 | AC005008.2 | 0.4842 |
| ENSP00000280560 | ABCB9 | 0.4842 |
| ENSP00000354518 | ZNF830 | 0.4816 |
| ENSP00000287727 | ZFYVE9 | 0.4816 |
| ENSP00000296127 | ZDHHC3 | 0.4816 |
| ENSP00000262577 | ZC3H3 | 0.4816 |
| ENSP00000246914 | WNK4 | 0.4816 |
| ENSP00000355510 | WDR64 | 0.4816 |
| ENSP00000423067 | WDR36 | 0.4816 |
| ENSP00000409952 | VPS52 | 0.4816 |
| ENSP00000320957 | VGLL2 | 0.4816 |
| ENSP00000417175 | VAPB | 0.4816 |
| ENSP00000289865 | USP21 | 0.4816 |
| ENSP00000287156 | UBE2L6 | 0.4816 |
| ENSP00000434516 | TXNRD1 | 0.4816 |
| ENSP00000262120 | TWSG1 | 0.4816 |
| ENSP00000362386 | TTC16 | 0.4816 |
| ENSP00000355621 | TTC13 | 0.4816 |
| ENSP00000374399 | TRMT61A | 0.4816 |
| ENSP00000401946 | TNRC6B | 0.4816 |
| ENSP00000296861 | TNFRSF21 | 0.4816 |
| ENSP00000417517 | TLR9 | 0.4816 |
| ENSP00000340089 | TLR5 | 0.4816 |
| ENSP00000378332 | TGFB1I1 | 0.4816 |
| ENSP00000365431 | TCF19 | 0.4816 |
| ENSP00000246080 | TCF15 | 0.4816 |
| ENSP00000225504 | SUPT4H1 | 0.4816 |
| ENSP00000385616 | SUN2 | 0.4816 |
| ENSP00000457522 | STX16-NPEPL1 | 0.4816 |
| ENSP00000217961 | STS | 0.4816 |
| ENSP00000218089 | STAG2 | 0.4816 |
| ENSP00000295702 | SSR2 | 0.4816 |
| ENSP00000280098 | SPOPL | 0.4816 |
| ENSP00000302812 | SP7 | 0.4816 |
| ENSP00000385746 | SMS | 0.4816 |
| ENSP00000340409 | SMPD1 | 0.4816 |
| ENSP00000367123 | SLC3A2 | 0.4816 |
| ENSP00000368981 | SLC35B3 | 0.4816 |
| ENSP00000352456 | SLC33A1 | 0.4816 |
| ENSP00000307859 | SLC22A18 | 0.4816 |
| ENSP00000419362 | SHOX2 | 0.4816 |
| ENSP00000215793 | SF3A1 | 0.4816 |
| ENSP00000284951 | SEL1L2 | 0.4816 |
| ENSP00000353292 | SCNN1A | 0.4816 |
| ENSP00000344106 | RTN3 | 0.4816 |
| ENSP00000260563 | RTCA | 0.4816 |
| ENSP00000468690 | RP11-618P17.4 | 0.4816 |
| ENSP00000452401 | RP11-47I22.4 | 0.4816 |
| ENSP00000220676 | RP1 | 0.4816 |
| ENSP00000278833 | ROM1 | 0.4816 |
| ENSP00000386588 | RGPD3 | 0.4816 |
| ENSP00000368959 | REEP5 | 0.4816 |

| | | |
|---|---|---|
| ENSP00000303482 | REEP4 | 0.4816 |
| ENSP00000382239 | RBM12B | 0.4816 |
| ENSP00000348632 | RBCK1 | 0.4816 |
| ENSP00000386414 | RAD21L1 | 0.4816 |
| ENSP00000309116 | PTPN7 | 0.4816 |
| ENSP00000332274 | PTP4A3 | 0.4816 |
| ENSP00000361547 | PRRX2 | 0.4816 |
| ENSP00000399970 | PPP2R2D | 0.4816 |
| ENSP00000234038 | PPP1R7 | 0.4816 |
| ENSP00000350098 | POP5 | 0.4816 |
| ENSP00000339529 | POP1 | 0.4816 |
| ENSP00000303088 | POLR3D | 0.4816 |
| ENSP00000368349 | POLA1 | 0.4816 |
| ENSP00000263657 | PNO1 | 0.4816 |
| ENSP00000070846 | PKP2 | 0.4816 |
| ENSP00000322218 | PITPNM2 | 0.4816 |
| ENSP00000359683 | PI4K2A | 0.4816 |
| ENSP00000329933 | PHF12 | 0.4816 |
| ENSP00000298198 | PGM2L1 | 0.4816 |
| ENSP00000404676 | PGGT1B | 0.4816 |
| ENSP00000364145 | PFKFB1 | 0.4816 |
| ENSP00000346725 | PES1 | 0.4816 |
| ENSP00000282493 | PDZD2 | 0.4816 |
| ENSP00000312634 | PDLIM2 | 0.4816 |
| ENSP00000307241 | PDHB | 0.4816 |
| ENSP00000305556 | PCBP1 | 0.4816 |
| ENSP00000221265 | PAF1 | 0.4816 |
| ENSP00000007414 | OSBPL7 | 0.4816 |
| ENSP00000320291 | OSBPL1A | 0.4816 |
| ENSP00000330862 | OPCML | 0.4816 |
| ENSP00000366519 | NSUN6 | 0.4816 |
| ENSP00000438378 | NRK | 0.4816 |
| ENSP00000356355 | NEK7 | 0.4816 |
| ENSP00000311997 | NEFH | 0.4816 |
| ENSP00000233627 | NDUFS7 | 0.4816 |
| ENSP00000362616 | NAP1L2 | 0.4816 |
| ENSP00000249442 | MTX2 | 0.4816 |
| ENSP00000312244 | MSL3 | 0.4816 |
| ENSP00000390331 | MRPS17 | 0.4816 |
| ENSP00000339844 | MRPL43 | 0.4816 |
| ENSP00000361084 | MRPL14 | 0.4816 |
| ENSP00000331310 | MORF4L1 | 0.4816 |
| ENSP00000299855 | MMP3 | 0.4816 |
| ENSP00000261758 | MESDC2 | 0.4816 |
| ENSP00000251871 | MED17 | 0.4816 |
| ENSP00000233121 | MAPRE3 | 0.4816 |
| ENSP00000241014 | MAPK8IP1 | 0.4816 |
| ENSP00000240304 | LUC7L3 | 0.4816 |
| ENSP00000270115 | LRRC56 | 0.4816 |
| ENSP00000291592 | LRRC3 | 0.4816 |
| ENSP00000384665 | LPAR2 | 0.4816 |
| ENSP00000351755 | LPAR1 | 0.4816 |
| ENSP00000310431 | LMAN1L | 0.4816 |

| | | |
|---|---|---|
| ENSP00000361007 | LIPJ | 0.4816 |
| ENSP00000359691 | LGSN | 0.4816 |
| ENSP00000454071 | LDLR | 0.4816 |
| ENSP00000321997 | LARP1B | 0.4816 |
| ENSP00000348704 | KPNA5 | 0.4816 |
| ENSP00000393963 | KIFC1 | 0.4816 |
| ENSP00000387875 | KIF4B | 0.4816 |
| ENSP00000363232 | KIF12 | 0.4816 |
| ENSP00000194118 | KIAA0141 | 0.4816 |
| ENSP00000216039 | JOSD1 | 0.4816 |
| ENSP00000380692 | JHDM1D | 0.4816 |
| ENSP00000341280 | JARID2 | 0.4816 |
| ENSP00000418397 | IVD | 0.4816 |
| ENSP00000266646 | INHBE | 0.4816 |
| ENSP00000416683 | IGFALS | 0.4816 |
| ENSP00000319623 | IGDCC4 | 0.4816 |
| ENSP00000362121 | IFT52 | 0.4816 |
| ENSP00000263642 | IFIH1 | 0.4816 |
| ENSP00000265986 | IDE | 0.4816 |
| ENSP00000358424 | HSD3B2 | 0.4816 |
| ENSP00000312625 | HP1BP3 | 0.4816 |
| ENSP00000278715 | HMBS | 0.4816 |
| ENSP00000230361 | GUCA1B | 0.4816 |
| ENSP00000303997 | GTPBP2 | 0.4816 |
| ENSP00000288955 | GRHL3 | 0.4816 |
| ENSP00000334952 | GPR125 | 0.4816 |
| ENSP00000203556 | GMIP | 0.4816 |
| ENSP00000350757 | GMFB | 0.4816 |
| ENSP00000368119 | GALT | 0.4816 |
| ENSP00000352738 | G3BP2 | 0.4816 |
| ENSP00000274963 | FGD2 | 0.4816 |
| ENSP00000244426 | FBXO9 | 0.4816 |
| ENSP00000229758 | FBXO5 | 0.4816 |
| ENSP00000406229 | FAT1 | 0.4816 |
| ENSP00000368984 | EXOSC9 | 0.4816 |
| ENSP00000369409 | EXOC3L4 | 0.4816 |
| ENSP00000325674 | EXOC3L1 | 0.4816 |
| ENSP00000311873 | EXO1 | 0.4816 |
| ENSP00000266517 | ETNK1 | 0.4816 |
| ENSP00000306999 | ESCO2 | 0.4816 |
| ENSP00000269214 | ESCO1 | 0.4816 |
| ENSP00000375809 | ERCC2 | 0.4816 |
| ENSP00000013807 | ERCC1 | 0.4816 |
| ENSP00000263735 | EPCAM | 0.4816 |
| ENSP00000352162 | ELAVL3 | 0.4816 |
| ENSP00000436049 | EIF3M | 0.4816 |
| ENSP00000370258 | EIF3CL | 0.4816 |
| ENSP00000346964 | EGFLAM | 0.4816 |
| ENSP00000301727 | E4F1 | 0.4816 |
| ENSP00000258198 | DYNC1LI2 | 0.4816 |
| ENSP00000451253 | DUX4L6 | 0.4816 |
| ENSP00000240100 | DUSP4 | 0.4816 |
| ENSP00000280904 | DSC2 | 0.4816 |

| | | |
|---|---|---|
| ENSP00000307197 | DNAJB7 | 0.4816 |
| ENSP00000280886 | DIP2C | 0.4816 |
| ENSP00000225296 | DHX33 | 0.4816 |
| ENSP00000362795 | CXCR3 | 0.4816 |
| ENSP00000291554 | CRYAA | 0.4816 |
| ENSP00000276571 | CRH | 0.4816 |
| ENSP00000315775 | COG4 | 0.4816 |
| ENSP00000360939 | CMPK1 | 0.4816 |
| ENSP00000221455 | CLASRP | 0.4816 |
| ENSP00000443140 | CHURC1-FNTB | 0.4816 |
| ENSP00000297265 | CHMP4C | 0.4816 |
| ENSP00000326238 | CAPZA3 | 0.4816 |
| ENSP00000380349 | CAPN3 | 0.4816 |
| ENSP00000262138 | CACNG4 | 0.4816 |
| ENSP00000328173 | C1S | 0.4816 |
| ENSP00000361577 | C10orf11 | 0.4816 |
| ENSP00000252593 | BST2 | 0.4816 |
| ENSP00000362372 | BRWD3 | 0.4816 |
| ENSP00000314132 | BOK | 0.4816 |
| ENSP00000393840 | BLOC1S3 | 0.4816 |
| ENSP00000353458 | BAZ1A | 0.4816 |
| ENSP00000321195 | ATP11B | 0.4816 |
| ENSP00000269197 | ASXL3 | 0.4816 |
| ENSP00000364694 | ASPN | 0.4816 |
| ENSP00000264607 | ASB1 | 0.4816 |
| ENSP00000355060 | ARHGEF10L | 0.4816 |
| ENSP00000296456 | APEH | 0.4816 |
| ENSP00000379802 | AMPD3 | 0.4816 |
| ENSP00000387739 | AMOTL1 | 0.4816 |
| ENSP00000296486 | AGXT2L1 | 0.4816 |
| ENSP00000331944 | AEN | 0.4816 |
| ENSP00000238561 | ADCK1 | 0.4816 |
| ENSP00000359958 | ZRANB2 | 0.4789 |
| ENSP00000282388 | ZFP36L2 | 0.4789 |
| ENSP00000337393 | UXT | 0.4789 |
| ENSP00000207549 | UNC13D | 0.4789 |
| ENSP00000356982 | UFC1 | 0.4789 |
| ENSP00000429084 | UBR5 | 0.4789 |
| ENSP00000359525 | UBE2NL | 0.4789 |
| ENSP00000253023 | UBE2M | 0.4789 |
| ENSP00000283033 | TXNDC11 | 0.4789 |
| ENSP00000231238 | TTC1 | 0.4789 |
| ENSP00000322802 | TSPYL5 | 0.4789 |
| ENSP00000410943 | TSPYL4 | 0.4789 |
| ENSP00000399668 | TSPY4 | 0.4789 |
| ENSP00000309036 | TSPAN17 | 0.4789 |
| ENSP00000379144 | TNRC6A | 0.4789 |
| ENSP00000265388 | TNPO3 | 0.4789 |
| ENSP00000294543 | TMCO4 | 0.4789 |
| ENSP00000356807 | TIPRL | 0.4789 |
| ENSP00000379999 | TIMM17B | 0.4789 |
| ENSP00000317334 | TCP1 | 0.4789 |
| ENSP00000355560 | TBCE | 0.4789 |

| | | |
|---|---|---|
| ENSP00000221855 | TBCB | 0.4789 |
| ENSP00000386921 | TBC1D14 | 0.4789 |
| ENSP00000310094 | TAOK2 | 0.4789 |
| ENSP00000362403 | TACR2 | 0.4789 |
| ENSP00000288422 | TAB3 | 0.4789 |
| ENSP00000263688 | SUCO | 0.4789 |
| ENSP00000263955 | STK17B | 0.4789 |
| ENSP00000302978 | SPRY3 | 0.4789 |
| ENSP00000321108 | SPOCK2 | 0.4789 |
| ENSP00000263694 | SNRNP40 | 0.4789 |
| ENSP00000371297 | SNPH | 0.4789 |
| ENSP00000366641 | SLC2A5 | 0.4789 |
| ENSP00000305529 | SIRPG | 0.4789 |
| ENSP00000263593 | SIAE | 0.4789 |
| ENSP00000278407 | SERPING1 | 0.4789 |
| ENSP00000330812 | SDR42E2 | 0.4789 |
| ENSP00000462116 | SAP30BP | 0.4789 |
| ENSP00000305416 | S1PR1 | 0.4789 |
| ENSP00000267484 | RTN1 | 0.4789 |
| ENSP00000223862 | RLN1 | 0.4789 |
| ENSP00000356429 | RGS1 | 0.4789 |
| ENSP00000452126 | RGMA | 0.4789 |
| ENSP00000306335 | RFX2 | 0.4789 |
| ENSP00000221515 | RETN | 0.4789 |
| ENSP00000418070 | PVRL3 | 0.4789 |
| ENSP00000355923 | PTPN14 | 0.4789 |
| ENSP00000295901 | PSMD6 | 0.4789 |
| ENSP00000257181 | PRPF38A | 0.4789 |
| ENSP00000302114 | PRELID1 | 0.4789 |
| ENSP00000335083 | PPP2R2C | 0.4789 |
| ENSP00000343943 | PPOX | 0.4789 |
| ENSP00000299853 | POLR3E | 0.4789 |
| ENSP00000356104 | PM20D1 | 0.4789 |
| ENSP00000263946 | PKP1 | 0.4789 |
| ENSP00000350676 | PHF8 | 0.4789 |
| ENSP00000378784 | PHF20L1 | 0.4789 |
| ENSP00000315680 | PEX7 | 0.4789 |
| ENSP00000005178 | PDK4 | 0.4789 |
| ENSP00000466214 | PDCD5 | 0.4789 |
| ENSP00000358862 | PCGF6 | 0.4789 |
| ENSP00000257789 | ORC3 | 0.4789 |
| ENSP00000464146 | OAZ1 | 0.4789 |
| ENSP00000254998 | NXT1 | 0.4789 |
| ENSP00000361658 | NUP188 | 0.4789 |
| ENSP00000365160 | NUDT11 | 0.4789 |
| ENSP00000301411 | NTF4 | 0.4789 |
| ENSP00000320089 | NKX2-6 | 0.4789 |
| ENSP00000238616 | NEK9 | 0.4789 |
| ENSP00000218652 | NDFIP2 | 0.4789 |
| ENSP00000369915 | NAP1L4 | 0.4789 |
| ENSP00000468991 | MTRNR2L12 | 0.4789 |
| ENSP00000354961 | MT-ND4 | 0.4789 |
| ENSP00000255087 | MTL5 | 0.4789 |

| | | |
|---|---|---|
| ENSP00000414287 | MST1 | 0.4789 |
| ENSP00000285298 | MRPS17 | 0.4789 |
| ENSP00000417602 | MRPL47 | 0.4789 |
| ENSP00000233545 | MPV17 | 0.4789 |
| ENSP00000416015 | MICAL3 | 0.4789 |
| ENSP00000297347 | MED30 | 0.4789 |
| ENSP00000357047 | MED23 | 0.4789 |
| ENSP00000325612 | MED16 | 0.4789 |
| ENSP00000308383 | LSP1 | 0.4789 |
| ENSP00000250173 | LRRC6 | 0.4789 |
| ENSP00000283415 | LPCAT1 | 0.4789 |
| ENSP00000301159 | LIN37 | 0.4789 |
| ENSP00000339916 | LIMK2 | 0.4789 |
| ENSP00000355785 | LEFTY2 | 0.4789 |
| ENSP00000363944 | LAS1L | 0.4789 |
| ENSP00000359074 | L1CAM | 0.4789 |
| ENSP00000287152 | KIF6 | 0.4789 |
| ENSP00000272117 | ITPKB | 0.4789 |
| ENSP00000273283 | ITIH1 | 0.4789 |
| ENSP00000318641 | INTS3 | 0.4789 |
| ENSP00000385722 | INTS1 | 0.4789 |
| ENSP00000233331 | INO80B | 0.4789 |
| ENSP00000284202 | IMPACT | 0.4789 |
| ENSP00000005558 | IFRD1 | 0.4789 |
| ENSP00000262626 | HPN | 0.4789 |
| ENSP00000295256 | HPGDS | 0.4789 |
| ENSP00000398890 | HLA-DMB | 0.4789 |
| ENSP00000328484 | HIST1H2AJ | 0.4789 |
| ENSP00000359454 | HFM1 | 0.4789 |
| ENSP00000361180 | GTF3C5 | 0.4789 |
| ENSP00000314380 | GRINA | 0.4789 |
| ENSP00000332549 | GRIN2A | 0.4789 |
| ENSP00000397351 | GRID2IP | 0.4789 |
| ENSP00000319286 | GP5 | 0.4789 |
| ENSP00000284116 | GDPD1 | 0.4789 |
| ENSP00000358451 | GDAP2 | 0.4789 |
| ENSP00000282538 | GADL1 | 0.4789 |
| ENSP00000305692 | GAA | 0.4789 |
| ENSP00000305603 | FUT3 | 0.4789 |
| ENSP00000375748 | FUT2 | 0.4789 |
| ENSP00000396673 | FTSJ3 | 0.4789 |
| ENSP00000295633 | FSTL1 | 0.4789 |
| ENSP00000254122 | FSHB | 0.4789 |
| ENSP00000220584 | FDFT1 | 0.4789 |
| ENSP00000377280 | FBXO8 | 0.4789 |
| ENSP00000364486 | FBP2 | 0.4789 |
| ENSP00000321951 | FAIM2 | 0.4789 |
| ENSP00000321326 | F2R | 0.4789 |
| ENSP00000379032 | EXT2 | 0.4789 |
| ENSP00000341743 | ENSA | 0.4789 |
| ENSP00000345974 | ENOSF1 | 0.4789 |
| ENSP00000426741 | EFNA3 | 0.4789 |
| ENSP00000347596 | EFEMP1 | 0.4789 |

| | | |
|---|---|---|
| ENSP00000380597 | EEF1E1-MUTED | 0.4789 |
| ENSP00000356920 | DUSP12 | 0.4789 |
| ENSP00000332151 | DSE | 0.4789 |
| ENSP00000353608 | DSC3 | 0.4789 |
| ENSP00000362041 | DNAJC9 | 0.4789 |
| ENSP00000296097 | DNAJC5G | 0.4789 |
| ENSP00000396896 | DNAJC4 | 0.4789 |
| ENSP00000344431 | DNAJB13 | 0.4789 |
| ENSP00000252011 | DHX35 | 0.4789 |
| ENSP00000297436 | DEFA6 | 0.4789 |
| ENSP00000359151 | DBT | 0.4789 |
| ENSP00000260327 | CTDSPL2 | 0.4789 |
| ENSP00000316416 | CSH1 | 0.4789 |
| ENSP00000381333 | CRHR1 | 0.4789 |
| ENSP00000264828 | COL5A3 | 0.4789 |
| ENSP00000259938 | CLPS | 0.4789 |
| ENSP00000290332 | CLIC6 | 0.4789 |
| ENSP00000432279 | CHMP1B | 0.4789 |
| ENSP00000358595 | CGA | 0.4789 |
| ENSP00000284382 | CERS3 | 0.4789 |
| ENSP00000271332 | CELSR2 | 0.4789 |
| ENSP00000398880 | CDK12 | 0.4789 |
| ENSP00000371689 | CDH12 | 0.4789 |
| ENSP00000339393 | CCR6 | 0.4789 |
| ENSP00000259607 | CCL21 | 0.4789 |
| ENSP00000221554 | CCDC130 | 0.4789 |
| ENSP00000386959 | CAPS2 | 0.4789 |
| ENSP00000335320 | BCL9L | 0.4789 |
| ENSP00000357748 | BCCIP | 0.4789 |
| ENSP00000300404 | B4GALNT2 | 0.4789 |
| ENSP00000376965 | ATXN3 | 0.4789 |
| ENSP00000290949 | ATP6V0D1 | 0.4789 |
| ENSP00000321388 | ASB4 | 0.4789 |
| ENSP00000311165 | AQP1 | 0.4789 |
| ENSP00000338777 | AP3S2 | 0.4789 |
| ENSP00000339109 | ANAPC1 | 0.4789 |
| ENSP00000239891 | ALG5 | 0.4789 |
| ENSP00000345774 | ALDH3A2 | 0.4789 |
| ENSP00000393887 | AHSG | 0.4789 |
| ENSP00000314036 | AGPAT4 | 0.4789 |
| ENSP00000304501 | ADORA2B | 0.4789 |
| ENSP00000278175 | ADM | 0.4789 |
| ENSP00000379865 | ADHFE1 | 0.4789 |
| ENSP00000219054 | ACSM2A | 0.4789 |
| ENSP00000323036 | ACPP | 0.4789 |
| ENSP00000367086 | ACOT7 | 0.4789 |
| ENSP00000366084 | ABCC4 | 0.4789 |
| ENSP00000324842 | AACS | 0.4789 |
| ENSP00000348419 | ZNF44 | 0.4763 |
| ENSP00000337159 | ZFYVE16 | 0.4763 |
| ENSP00000261015 | WDR12 | 0.4763 |
| ENSP00000387230 | UPP2 | 0.4763 |
| ENSP00000323740 | UCP3 | 0.4763 |

| | | |
|---|---|---|
| ENSP00000382982 | TUBA3C | 0.4763 |
| ENSP00000398803 | TSTA3 | 0.4763 |
| ENSP00000252015 | TRPC4AP | 0.4763 |
| ENSP00000261884 | TRIP4 | 0.4763 |
| ENSP00000442778 | TRAPPC2P1 | 0.4763 |
| ENSP00000299608 | TMX3 | 0.4763 |
| ENSP00000371376 | TLR6 | 0.4763 |
| ENSP00000326531 | THOC6 | 0.4763 |
| ENSP00000364037 | TEX10 | 0.4763 |
| ENSP00000221543 | TBC1D17 | 0.4763 |
| ENSP00000357075 | TAGLN2 | 0.4763 |
| ENSP00000225396 | TADA2A | 0.4763 |
| ENSP00000357307 | SYT11 | 0.4763 |
| ENSP00000233025 | SPCS1 | 0.4763 |
| ENSP00000323439 | SMC6 | 0.4763 |
| ENSP00000354957 | SMC5 | 0.4763 |
| ENSP00000392617 | SMARCD2 | 0.4763 |
| ENSP00000328940 | SLX1B | 0.4763 |
| ENSP00000417654 | SLC35A5 | 0.4763 |
| ENSP00000366797 | SLC22A12 | 0.4763 |
| ENSP00000340465 | SLC20A2 | 0.4763 |
| ENSP00000248566 | SHFM1 | 0.4763 |
| ENSP00000248929 | SGSM3 | 0.4763 |
| ENSP00000361366 | SFTPD | 0.4763 |
| ENSP00000321674 | SEPT4 | 0.4763 |
| ENSP00000264454 | SEC22C | 0.4763 |
| ENSP00000255858 | SEC14L4 | 0.4763 |
| ENSP00000257549 | SDS | 0.4763 |
| ENSP00000300061 | SCNN1G | 0.4763 |
| ENSP00000346534 | SCN8A | 0.4763 |
| ENSP00000371471 | RSAD2 | 0.4763 |
| ENSP00000362092 | RRAGC | 0.4763 |
| ENSP00000262850 | RRAGB | 0.4763 |
| ENSP00000230050 | RPS12 | 0.4763 |
| ENSP00000450353 | RP11-162P23.2 | 0.4763 |
| ENSP00000380876 | RGS11 | 0.4763 |
| ENSP00000303192 | RGL1 | 0.4763 |
| ENSP00000371321 | RFC1 | 0.4763 |
| ENSP00000308699 | REC8 | 0.4763 |
| ENSP00000391121 | RDH13 | 0.4763 |
| ENSP00000364582 | RCC2 | 0.4763 |
| ENSP00000374069 | PTPRN2 | 0.4763 |
| ENSP00000321999 | PTH1R | 0.4763 |
| ENSP00000318176 | PRKRA | 0.4763 |
| ENSP00000363092 | PRKG1 | 0.4763 |
| ENSP00000394863 | PPT1 | 0.4763 |
| ENSP00000311677 | PPP1R8 | 0.4763 |
| ENSP00000450337 | PPFIA2 | 0.4763 |
| ENSP00000215885 | PLA2G3 | 0.4763 |
| ENSP00000312286 | PLA2G1B | 0.4763 |
| ENSP00000349208 | PIWIL2 | 0.4763 |
| ENSP00000364204 | PINK1 | 0.4763 |
| ENSP00000359848 | PIGK | 0.4763 |

| | | |
|---|---|---|
| ENSP00000345771 | PFKM | 0.4763 |
| ENSP00000331574 | PDE1A | 0.4763 |
| ENSP00000411825 | PDCD6IP | 0.4763 |
| ENSP00000302814 | OTP | 0.4763 |
| ENSP00000379804 | OSBPL10 | 0.4763 |
| ENSP00000342278 | OAS2 | 0.4763 |
| ENSP00000264670 | NSUN2 | 0.4763 |
| ENSP00000338766 | NPHP3 | 0.4763 |
| ENSP00000261797 | NDST1 | 0.4763 |
| ENSP00000393275 | NANOS1 | 0.4763 |
| ENSP00000302021 | MUC7 | 0.4763 |
| ENSP00000281047 | MSGN1 | 0.4763 |
| ENSP00000308275 | MRPL38 | 0.4763 |
| ENSP00000318318 | MPI | 0.4763 |
| ENSP00000198801 | MOGAT2 | 0.4763 |
| ENSP00000262430 | MLYCD | 0.4763 |
| ENSP00000352455 | MFAP5 | 0.4763 |
| ENSP00000337340 | MED19 | 0.4763 |
| ENSP00000263702 | MECR | 0.4763 |
| ENSP00000358400 | MDN1 | 0.4763 |
| ENSP00000430487 | MATN2 | 0.4763 |
| ENSP00000360690 | LONRF3 | 0.4763 |
| ENSP00000249075 | LIF | 0.4763 |
| ENSP00000308369 | LEMD3 | 0.4763 |
| ENSP00000304422 | KREMEN2 | 0.4763 |
| ENSP00000209884 | KLHL20 | 0.4763 |
| ENSP00000352282 | KLHDC1 | 0.4763 |
| ENSP00000307023 | KLF11 | 0.4763 |
| ENSP00000420040 | KCNIP2 | 0.4763 |
| ENSP00000295321 | IWS1 | 0.4763 |
| ENSP00000311492 | ISX | 0.4763 |
| ENSP00000323424 | ISG20L2 | 0.4763 |
| ENSP00000273221 | IQSEC1 | 0.4763 |
| ENSP00000355205 | INO80 | 0.4763 |
| ENSP00000329384 | IL22 | 0.4763 |
| ENSP00000258774 | HUS1 | 0.4763 |
| ENSP00000321389 | HIST1H2AC | 0.4763 |
| ENSP00000340736 | GYG1 | 0.4763 |
| ENSP00000218006 | GUCY2F | 0.4763 |
| ENSP00000279593 | GRIN2B | 0.4763 |
| ENSP00000230056 | GMNN | 0.4763 |
| ENSP00000240093 | FZD3 | 0.4763 |
| ENSP00000384823 | FBXO11 | 0.4763 |
| ENSP00000320604 | FAXDC2 | 0.4763 |
| ENSP00000322229 | FADS1 | 0.4763 |
| ENSP00000301607 | EVPL | 0.4763 |
| ENSP00000357175 | ETV3 | 0.4763 |
| ENSP00000296741 | ENPP6 | 0.4763 |
| ENSP00000248378 | EMC6 | 0.4763 |
| ENSP00000220325 | EHD4 | 0.4763 |
| ENSP00000273668 | EAF2 | 0.4763 |
| ENSP00000010132 | DYRK4 | 0.4763 |
| ENSP00000451700 | DUX4L7 | 0.4763 |

| | | |
|---|---|---|
| ENSP00000451798 | DUX4L3 | 0.4763 |
| ENSP00000346160 | DUSP19 | 0.4763 |
| ENSP00000320650 | DNAJC25 | 0.4763 |
| ENSP00000378508 | DNAJC22 | 0.4763 |
| ENSP00000249356 | DNAJB9 | 0.4763 |
| ENSP00000378324 | DNAJA4 | 0.4763 |
| ENSP00000311273 | DNAH7 | 0.4763 |
| ENSP00000401514 | DNAH1 | 0.4763 |
| ENSP00000240306 | DLX4 | 0.4763 |
| ENSP00000400258 | DLGAP2 | 0.4763 |
| ENSP00000295373 | DHX57 | 0.4763 |
| ENSP00000368173 | DHRS13 | 0.4763 |
| ENSP00000280665 | DCP1B | 0.4763 |
| ENSP00000360958 | CYP4A22 | 0.4763 |
| ENSP00000275016 | CYP39A1 | 0.4763 |
| ENSP00000346340 | COPS8 | 0.4763 |
| ENSP00000349790 | COL9A1 | 0.4763 |
| ENSP00000221232 | CNOT3 | 0.4763 |
| ENSP00000250378 | CMA1 | 0.4763 |
| ENSP00000353475 | CLDN7 | 0.4763 |
| ENSP00000183605 | CLDN18 | 0.4763 |
| ENSP00000311083 | CKS1B | 0.4763 |
| ENSP00000315602 | CHRNA3 | 0.4763 |
| ENSP00000255409 | CHI3L1 | 0.4763 |
| ENSP00000258969 | CHAD | 0.4763 |
| ENSP00000319052 | CETN1 | 0.4763 |
| ENSP00000271688 | CERS2 | 0.4763 |
| ENSP00000303058 | CEP120 | 0.4763 |
| ENSP00000348527 | CENPL | 0.4763 |
| ENSP00000293275 | CCL16 | 0.4763 |
| ENSP00000316114 | CCDC101 | 0.4763 |
| ENSP00000354812 | C9orf114 | 0.4763 |
| ENSP00000363768 | C1QC | 0.4763 |
| ENSP00000361915 | BMP8B | 0.4763 |
| ENSP00000375067 | BCAS3 | 0.4763 |
| ENSP00000404503 | BBC3 | 0.4763 |
| ENSP00000342434 | BAZ1B | 0.4763 |
| ENSP00000265605 | ALDH8A1 | 0.4763 |
| ENSP00000450436 | ALDH6A1 | 0.4763 |
| ENSP00000290597 | ALDH4A1 | 0.4763 |
| ENSP00000233710 | ACADL | 0.4763 |
| ENSP00000341237 | AC008132.1 | 0.4763 |
| ENSP00000382957 | ZNF705D | 0.4737 |
| ENSP00000264447 | ZNF638 | 0.4737 |
| ENSP00000362527 | ZNF362 | 0.4737 |
| ENSP00000268489 | ZFHX3 | 0.4737 |
| ENSP00000285021 | XPC | 0.4737 |
| ENSP00000226760 | WFS1 | 0.4737 |
| ENSP00000054666 | VAMP3 | 0.4737 |
| ENSP00000262946 | UQCR11 | 0.4737 |
| ENSP00000366819 | UCHL3 | 0.4737 |
| ENSP00000343001 | UBE4B | 0.4737 |
| ENSP00000261427 | UBE2K | 0.4737 |

| | | |
|---|---|---|
| ENSP00000430031 | TXNRD3 | 0.4737 |
| ENSP00000380552 | TSPAN4 | 0.4737 |
| ENSP00000356476 | TRMT1L | 0.4737 |
| ENSP00000006275 | TRAPPC6A | 0.4737 |
| ENSP00000416037 | TMPRSS4 | 0.4737 |
| ENSP00000259365 | TMOD1 | 0.4737 |
| ENSP00000261881 | TIPIN | 0.4737 |
| ENSP00000409195 | TBC1D3H | 0.4737 |
| ENSP00000346576 | SYTL2 | 0.4737 |
| ENSP00000249344 | STRIP2 | 0.4737 |
| ENSP00000429243 | STMN2 | 0.4737 |
| ENSP00000370891 | STK35 | 0.4737 |
| ENSP00000260956 | SSB | 0.4737 |
| ENSP00000296215 | SNIP1 | 0.4737 |
| ENSP00000342082 | SLPI | 0.4737 |
| ENSP00000287766 | SLC6A1 | 0.4737 |
| ENSP00000445340 | SLC5A8 | 0.4737 |
| ENSP00000470652 | SLC35E1 | 0.4737 |
| ENSP00000235345 | SLC35D1 | 0.4737 |
| ENSP00000416240 | SEBOX | 0.4737 |
| ENSP00000265565 | SCAP | 0.4737 |
| ENSP00000350261 | SAMD4A | 0.4737 |
| ENSP00000357726 | S100A12 | 0.4737 |
| ENSP00000268661 | RPL3L | 0.4737 |
| ENSP00000306637 | RGPD8 | 0.4737 |
| ENSP00000258349 | RC3H1 | 0.4737 |
| ENSP00000334813 | RAX | 0.4737 |
| ENSP00000344936 | PTTG1 | 0.4737 |
| ENSP00000280362 | PTS | 0.4737 |
| ENSP00000302846 | PTGER4 | 0.4737 |
| ENSP00000325919 | PSMG2 | 0.4737 |
| ENSP00000349856 | PRRC2B | 0.4737 |
| ENSP00000315379 | PRPF3 | 0.4737 |
| ENSP00000377783 | PROS1 | 0.4737 |
| ENSP00000302478 | POLR1D | 0.4737 |
| ENSP00000372316 | POLN | 0.4737 |
| ENSP00000364902 | POFUT1 | 0.4737 |
| ENSP00000347038 | PLSCR4 | 0.4737 |
| ENSP00000417003 | PLEKHM3 | 0.4737 |
| ENSP00000299001 | PIWIL4 | 0.4737 |
| ENSP00000264864 | PI4K2B | 0.4737 |
| ENSP00000216780 | PCK2 | 0.4737 |
| ENSP00000396040 | PCED1B | 0.4737 |
| ENSP00000377527 | PC | 0.4737 |
| ENSP00000354676 | OSTC | 0.4737 |
| ENSP00000263847 | OSBP | 0.4737 |
| ENSP00000306561 | OGG1 | 0.4737 |
| ENSP00000295119 | NUP35 | 0.4737 |
| ENSP00000355587 | NTPCR | 0.4737 |
| ENSP00000337501 | NRIP2 | 0.4737 |
| ENSP00000373810 | NOP16 | 0.4737 |
| ENSP00000264230 | NOA1 | 0.4737 |
| ENSP00000364578 | NELFE | 0.4737 |

| | | |
|---|---|---|
| ENSP00000389160 | NDUFA11 | 0.4737 |
| ENSP00000433681 | NCAPD3 | 0.4737 |
| ENSP00000389087 | NAGK | 0.4737 |
| ENSP00000371221 | MTMR6 | 0.4737 |
| ENSP00000431040 | MRPL22 | 0.4737 |
| ENSP00000040663 | MRI1 | 0.4737 |
| ENSP00000306697 | MRAP | 0.4737 |
| ENSP00000223114 | MOGAT3 | 0.4737 |
| ENSP00000263969 | MFN1 | 0.4737 |
| ENSP00000386908 | MEGF11 | 0.4737 |
| ENSP00000225728 | MED31 | 0.4737 |
| ENSP00000417235 | MED12L | 0.4737 |
| ENSP00000007969 | LRRC23 | 0.4737 |
| ENSP00000322270 | LPHN2 | 0.4737 |
| ENSP00000433352 | LMO7 | 0.4737 |
| ENSP00000261438 | KLF3 | 0.4737 |
| ENSP00000297814 | KIF27 | 0.4737 |
| ENSP00000260363 | KIF23 | 0.4737 |
| ENSP00000263181 | KIF18A | 0.4737 |
| ENSP00000394033 | KCNK2 | 0.4737 |
| ENSP00000360777 | INPP5E | 0.4737 |
| ENSP00000290200 | IL10RB | 0.4737 |
| ENSP00000240652 | IAPP | 0.4737 |
| ENSP00000429214 | HMGB4 | 0.4737 |
| ENSP00000352442 | HIST1H2BM | 0.4737 |
| ENSP00000366618 | HIST1H2BL | 0.4737 |
| ENSP00000341094 | HIST1H2AD | 0.4737 |
| ENSP00000355541 | HEATR1 | 0.4737 |
| ENSP00000355778 | H3F3A | 0.4737 |
| ENSP00000370555 | GYG2 | 0.4737 |
| ENSP00000340823 | GTF2F2 | 0.4737 |
| ENSP00000282753 | GRM1 | 0.4737 |
| ENSP00000368305 | GPCPD1 | 0.4737 |
| ENSP00000343676 | GJA4 | 0.4737 |
| ENSP00000237527 | GHRH | 0.4737 |
| ENSP00000253778 | GFPT2 | 0.4737 |
| ENSP00000449751 | FKBP11 | 0.4737 |
| ENSP00000403293 | FBXO43 | 0.4737 |
| ENSP00000349970 | ERGIC3 | 0.4737 |
| ENSP00000377374 | ERGIC1 | 0.4737 |
| ENSP00000365812 | EP400NL | 0.4737 |
| ENSP00000364073 | EIF4G3 | 0.4737 |
| ENSP00000340281 | EIF4G2 | 0.4737 |
| ENSP00000361668 | EDN2 | 0.4737 |
| ENSP00000389585 | DUX4L2 | 0.4737 |
| ENSP00000363412 | DNAJC25-GNG10 | 0.4737 |
| ENSP00000338019 | DNAJB2 | 0.4737 |
| ENSP00000312554 | DNAH12 | 0.4737 |
| ENSP00000316377 | DLGAP1 | 0.4737 |
| ENSP00000273814 | DGKQ | 0.4737 |
| ENSP00000331681 | DGCR6 | 0.4737 |
| ENSP00000323858 | DDX54 | 0.4737 |
| ENSP00000387006 | CWC22 | 0.4737 |

| | | |
|---|---|---|
| ENSP00000321260 | COX8A | 0.4737 |
| ENSP00000404078 | COX6A1P2 | 0.4737 |
| ENSP00000264389 | COPS4 | 0.4737 |
| ENSP00000243222 | COL10A1 | 0.4737 |
| ENSP00000353698 | CNPPD1 | 0.4737 |
| ENSP00000306579 | CERS6 | 0.4737 |
| ENSP00000216264 | CERK | 0.4737 |
| ENSP00000262607 | CECR1 | 0.4737 |
| ENSP00000363322 | CDC26 | 0.4737 |
| ENSP00000354947 | CAPZA2 | 0.4737 |
| ENSP00000343412 | BRE | 0.4737 |
| ENSP00000321507 | AZIN1 | 0.4737 |
| ENSP00000283558 | ATP11A | 0.4737 |
| ENSP00000387185 | ATHL1 | 0.4737 |
| ENSP00000261168 | ATF7IP | 0.4737 |
| ENSP00000328327 | ASB7 | 0.4737 |
| ENSP00000249822 | ARPP19 | 0.4737 |
| ENSP00000353796 | ANKRD53 | 0.4737 |
| ENSP00000249883 | AMOTL2 | 0.4737 |
| ENSP00000358807 | ALX3 | 0.4737 |
| ENSP00000374219 | ALKBH8 | 0.4737 |
| ENSP00000242375 | AKR1D1 | 0.4737 |
| ENSP00000380184 | AGPAT6 | 0.4737 |
| ENSP00000337463 | AGPAT1 | 0.4737 |
| ENSP00000343782 | ADRB3 | 0.4737 |
| ENSP00000336630 | ADORA2A | 0.4737 |
| ENSP00000310547 | ADCK5 | 0.4737 |
| ENSP00000361965 | ADA | 0.4737 |
| ENSP00000327453 | ACSM2B | 0.4737 |
| ENSP00000305804 | ZNF131 | 0.4711 |
| ENSP00000273320 | ZKSCAN7 | 0.4711 |
| ENSP00000417573 | YIPF6 | 0.4711 |
| ENSP00000364270 | XPA | 0.4711 |
| ENSP00000262139 | WIPI1 | 0.4711 |
| ENSP00000356433 | UST | 0.4711 |
| ENSP00000434676 | USP50 | 0.4711 |
| ENSP00000407487 | UNC45A | 0.4711 |
| ENSP00000354877 | ULK2 | 0.4711 |
| ENSP00000314655 | TSC22D3 | 0.4711 |
| ENSP00000296098 | TRIM54 | 0.4711 |
| ENSP00000284320 | TOMM70A | 0.4711 |
| ENSP00000419704 | TIMM10B | 0.4711 |
| ENSP00000339532 | THUMPD3 | 0.4711 |
| ENSP00000261600 | THOC1 | 0.4711 |
| ENSP00000364207 | TBC1D2 | 0.4711 |
| ENSP00000376317 | TAOK3 | 0.4711 |
| ENSP00000332287 | SYNGR1 | 0.4711 |
| ENSP00000305899 | SUV420H1 | 0.4711 |
| ENSP00000333292 | SPNS2 | 0.4711 |
| ENSP00000260197 | SORL1 | 0.4711 |
| ENSP00000339769 | SMEK2 | 0.4711 |
| ENSP00000350036 | SMC1B | 0.4711 |
| ENSP00000299798 | SLC9A5 | 0.4711 |

| | | |
|---|---|---|
| ENSP00000383698 | SLC2A7 | 0.4711 |
| ENSP00000272542 | SLC20A1 | 0.4711 |
| ENSP00000315137 | SGPP2 | 0.4711 |
| ENSP00000367991 | SEPT8 | 0.4711 |
| ENSP00000385695 | SEC14L6 | 0.4711 |
| ENSP00000301761 | SDHAF2 | 0.4711 |
| ENSP00000283254 | SCN3A | 0.4711 |
| ENSP00000287482 | SASS6 | 0.4711 |
| ENSP00000223129 | RPA3 | 0.4711 |
| ENSP00000391432 | RNPC3 | 0.4711 |
| ENSP00000357297 | RNF146 | 0.4711 |
| ENSP00000253571 | RLIM | 0.4711 |
| ENSP00000016946 | RGPD5 | 0.4711 |
| ENSP00000438346 | REEP1 | 0.4711 |
| ENSP00000267502 | RDH12 | 0.4711 |
| ENSP00000306817 | RBKS | 0.4711 |
| ENSP00000291294 | PTGIR | 0.4711 |
| ENSP00000335579 | PPP4R1L | 0.4711 |
| ENSP00000387176 | POTEJ | 0.4711 |
| ENSP00000352288 | PLXNB2 | 0.4711 |
| ENSP00000348772 | PITPNM1 | 0.4711 |
| ENSP00000269554 | PIP4K2B | 0.4711 |
| ENSP00000318966 | PINX1 | 0.4711 |
| ENSP00000357051 | PEX19 | 0.4711 |
| ENSP00000439467 | PDCD2 | 0.4711 |
| ENSP00000354724 | PCGF3 | 0.4711 |
| ENSP00000304767 | P2RY1 | 0.4711 |
| ENSP00000415904 | OAZ3 | 0.4711 |
| ENSP00000361544 | NUP62CL | 0.4711 |
| ENSP00000341083 | NPR2 | 0.4711 |
| ENSP00000268605 | NOL3 | 0.4711 |
| ENSP00000339495 | NELFB | 0.4711 |
| ENSP00000221166 | NEFM | 0.4711 |
| ENSP00000002125 | NDUFAF7 | 0.4711 |
| ENSP00000367346 | NDUFAF5 | 0.4711 |
| ENSP00000264363 | NDST4 | 0.4711 |
| ENSP00000362181 | NCR2 | 0.4711 |
| ENSP00000257829 | NAT10 | 0.4711 |
| ENSP00000255416 | MYBPH | 0.4711 |
| ENSP00000361170 | MUTYH | 0.4711 |
| ENSP00000367637 | MRS2 | 0.4711 |
| ENSP00000253686 | MRPS25 | 0.4711 |
| ENSP00000354086 | MRPL37 | 0.4711 |
| ENSP00000296102 | MRPL33 | 0.4711 |
| ENSP00000288937 | MRPL17 | 0.4711 |
| ENSP00000269202 | MEP1B | 0.4711 |
| ENSP00000292035 | MED27 | 0.4711 |
| ENSP00000326767 | MED25 | 0.4711 |
| ENSP00000358719 | ME1 | 0.4711 |
| ENSP00000343164 | MATN4 | 0.4711 |
| ENSP00000319341 | LSM10 | 0.4711 |
| ENSP00000327875 | LPAR5 | 0.4711 |
| ENSP00000383900 | LIPK | 0.4711 |

| | | |
|---|---|---|
| ENSP00000349477 | KLRG1 | 0.4711 |
| ENSP00000312397 | KLHL3 | 0.4711 |
| ENSP00000331682 | KLHL22 | 0.4711 |
| ENSP00000374566 | KIF19 | 0.4711 |
| ENSP00000394390 | KCTD15 | 0.4711 |
| ENSP00000271751 | KCNH1 | 0.4711 |
| ENSP00000328511 | KCNA4 | 0.4711 |
| ENSP00000322730 | ITM2C | 0.4711 |
| ENSP00000273541 | ISY1 | 0.4711 |
| ENSP00000322142 | ING5 | 0.4711 |
| ENSP00000296980 | IL22RA2 | 0.4711 |
| ENSP00000382707 | IFITM3 | 0.4711 |
| ENSP00000360876 | IFIT3 | 0.4711 |
| ENSP00000298902 | IFI27 | 0.4711 |
| ENSP00000255631 | HSPBP1 | 0.4711 |
| ENSP00000431063 | HSFX2 | 0.4711 |
| ENSP00000254521 | HSD17B7 | 0.4711 |
| ENSP00000289004 | HPD | 0.4711 |
| ENSP00000310749 | HPCAL1 | 0.4711 |
| ENSP00000225893 | HNF1B | 0.4711 |
| ENSP00000436714 | HCAR3 | 0.4711 |
| ENSP00000274306 | GZMA | 0.4711 |
| ENSP00000230036 | GPLD1 | 0.4711 |
| ENSP00000383431 | GAB4 | 0.4711 |
| ENSP00000246166 | FNTB | 0.4711 |
| ENSP00000297267 | FNDC1 | 0.4711 |
| ENSP00000364298 | FKBPL | 0.4711 |
| ENSP00000310841 | FBXO31 | 0.4711 |
| ENSP00000418748 | ESRP2 | 0.4711 |
| ENSP00000374212 | EP400 | 0.4711 |
| ENSP00000320346 | ELL3 | 0.4711 |
| ENSP00000262103 | EFCAB1 | 0.4711 |
| ENSP00000296161 | DTX3L | 0.4711 |
| ENSP00000244364 | DST | 0.4711 |
| ENSP00000359127 | DPH5 | 0.4711 |
| ENSP00000329890 | DEFA5 | 0.4711 |
| ENSP00000332340 | DDX28 | 0.4711 |
| ENSP00000221114 | DCTN6 | 0.4711 |
| ENSP00000311095 | CYP4A11 | 0.4711 |
| ENSP00000293778 | CXCL16 | 0.4711 |
| ENSP00000384109 | CUL4B | 0.4711 |
| ENSP00000320434 | CTNNAL1 | 0.4711 |
| ENSP00000254759 | COQ3 | 0.4711 |
| ENSP00000399013 | CNTNAP5 | 0.4711 |
| ENSP00000325548 | CNDP2 | 0.4711 |
| ENSP00000217402 | CHMP4B | 0.4711 |
| ENSP00000353720 | CES1 | 0.4711 |
| ENSP00000435926 | CELF1 | 0.4711 |
| ENSP00000442068 | CDCA3 | 0.4711 |
| ENSP00000258091 | CCT7 | 0.4711 |
| ENSP00000308750 | CBX2 | 0.4711 |
| ENSP00000246533 | CAPNS1 | 0.4711 |
| ENSP00000222969 | BUD31 | 0.4711 |

| | | |
|---|---|---|
| ENSP00000330753 | BRWD1 | 0.4711 |
| ENSP00000263080 | ASPA | 0.4711 |
| ENSP00000366522 | ARHGAP39 | 0.4711 |
| ENSP00000293599 | AQP5 | 0.4711 |
| ENSP00000453144 | APBA2 | 0.4711 |
| ENSP00000216366 | AP4S1 | 0.4711 |
| ENSP00000299626 | ALG8 | 0.4711 |
| ENSP00000363988 | ALDOB | 0.4711 |
| ENSP00000264409 | AGPAT9 | 0.4711 |
| ENSP00000333666 | ADI1 | 0.4711 |
| ENSP00000356975 | ADAMTS4 | 0.4711 |
| ENSP00000253382 | ACSS2 | 0.4711 |
| ENSP00000327916 | ACSM5 | 0.4711 |
| ENSP00000230048 | ACOT13 | 0.4711 |
| ENSP00000301452 | ACER1 | 0.4711 |
| ENSP00000324287 | ACAP2 | 0.4711 |
| ENSP00000439425 | AC025287.1 | 0.4711 |
| ENSP00000393511 | ABLIM2 | 0.4711 |
| ENSP00000382342 | ABCC1 | 0.4711 |
| ENSP00000319829 | ZNF687 | 0.4684 |
| ENSP00000411409 | ZNF598 | 0.4684 |
| ENSP00000377080 | ZFP1 | 0.4684 |
| ENSP00000369810 | VPS16 | 0.4684 |
| ENSP00000356905 | VNN1 | 0.4684 |
| ENSP00000339122 | VIPAS39 | 0.4684 |
| ENSP00000286428 | VBP1 | 0.4684 |
| ENSP00000261776 | VAC14 | 0.4684 |
| ENSP00000261637 | UTP20 | 0.4684 |
| ENSP00000254181 | USP29 | 0.4684 |
| ENSP00000285679 | USP25 | 0.4684 |
| ENSP00000356853 | UCK2 | 0.4684 |
| ENSP00000358674 | UBL4A | 0.4684 |
| ENSP00000363634 | TXNDC8 | 0.4684 |
| ENSP00000222567 | TWISTNB | 0.4684 |
| ENSP00000262605 | TTPAL | 0.4684 |
| ENSP00000377043 | TTLL6 | 0.4684 |
| ENSP00000382659 | TRPV1 | 0.4684 |
| ENSP00000368966 | TRPC3 | 0.4684 |
| ENSP00000261249 | TRMT5 | 0.4684 |
| ENSP00000369373 | TRIM5 | 0.4684 |
| ENSP00000295033 | TRIM17 | 0.4684 |
| ENSP00000239462 | TNN | 0.4684 |
| ENSP00000243347 | TNFAIP6 | 0.4684 |
| ENSP00000291532 | TMPRSS3 | 0.4684 |
| ENSP00000414786 | TMEM64 | 0.4684 |
| ENSP00000378588 | TIMM9 | 0.4684 |
| ENSP00000266304 | TEF | 0.4684 |
| ENSP00000310701 | TEAD2 | 0.4684 |
| ENSP00000440190 | TDRD3 | 0.4684 |
| ENSP00000381781 | TBC1D3B | 0.4684 |
| ENSP00000300504 | TBC1D21 | 0.4684 |
| ENSP00000266987 | TARBP2 | 0.4684 |
| ENSP00000299424 | TAF10 | 0.4684 |

| | | |
|---|---|---|
| ENSP00000340594 | SYDE2 | 0.4684 |
| ENSP00000332565 | SULT4A1 | 0.4684 |
| ENSP00000260128 | SULF1 | 0.4684 |
| ENSP00000305255 | STX8 | 0.4684 |
| ENSP00000366156 | SRM | 0.4684 |
| ENSP00000334538 | SREK1 | 0.4684 |
| ENSP00000345405 | SPRED3 | 0.4684 |
| ENSP00000264938 | SLC9A3 | 0.4684 |
| ENSP00000264047 | SLC5A7 | 0.4684 |
| ENSP00000304783 | SLC26A5 | 0.4684 |
| ENSP00000263753 | SGOL1 | 0.4684 |
| ENSP00000367893 | SEPHS1 | 0.4684 |
| ENSP00000263071 | SCARF1 | 0.4684 |
| ENSP00000369270 | RREB1 | 0.4684 |
| ENSP00000346063 | RPL7L1 | 0.4684 |
| ENSP00000389465 | RPL17-C18orf32 | 0.4684 |
| ENSP00000420449 | RP11-1220K2.2 | 0.4684 |
| ENSP00000434797 | RNF170 | 0.4684 |
| ENSP00000243326 | RIF1 | 0.4684 |
| ENSP00000262406 | RGS9 | 0.4684 |
| ENSP00000356641 | RFWD2 | 0.4684 |
| ENSP00000362751 | RABGAP1 | 0.4684 |
| ENSP00000216200 | PVALB | 0.4684 |
| ENSP00000331418 | PTPRM | 0.4684 |
| ENSP00000365528 | PTPRH | 0.4684 |
| ENSP00000020673 | PSD | 0.4684 |
| ENSP00000343629 | PRRC2C | 0.4684 |
| ENSP00000343103 | PRMT7 | 0.4684 |
| ENSP00000335185 | PRICKLE4 | 0.4684 |
| ENSP00000337641 | PPP2R5E | 0.4684 |
| ENSP00000299824 | PPP1R16B | 0.4684 |
| ENSP00000304353 | POP7 | 0.4684 |
| ENSP00000262483 | PITPNM3 | 0.4684 |
| ENSP00000347032 | PIP4K2C | 0.4684 |
| ENSP00000078527 | PIGV | 0.4684 |
| ENSP00000350263 | PIGN | 0.4684 |
| ENSP00000412189 | PGAM4 | 0.4684 |
| ENSP00000248633 | PEX1 | 0.4684 |
| ENSP00000322341 | PCBP4 | 0.4684 |
| ENSP00000422375 | NUDT16 | 0.4684 |
| ENSP00000356587 | NPHS2 | 0.4684 |
| ENSP00000438875 | NOVA1 | 0.4684 |
| ENSP00000365775 | MTHFR | 0.4684 |
| ENSP00000341082 | MRPL20 | 0.4684 |
| ENSP00000219271 | MMP15 | 0.4684 |
| ENSP00000325562 | MINOS1 | 0.4684 |
| ENSP00000349490 | MFNG | 0.4684 |
| ENSP00000364685 | MFAP2 | 0.4684 |
| ENSP00000230588 | MEP1A | 0.4684 |
| ENSP00000295065 | MEMO1 | 0.4684 |
| ENSP00000362948 | MED18 | 0.4684 |
| ENSP00000249910 | MBD4 | 0.4684 |
| ENSP00000331983 | LRRC16A | 0.4684 |

| | | |
|---|---|---|
| ENSP00000299022 | LIPC | 0.4684 |
| ENSP00000357835 | LHPP | 0.4684 |
| ENSP00000334052 | LGMN | 0.4684 |
| ENSP00000363458 | LDLRAP1 | 0.4684 |
| ENSP00000354360 | LAMC3 | 0.4684 |
| ENSP00000205386 | LAMB4 | 0.4684 |
| ENSP00000357973 | LACE1 | 0.4684 |
| ENSP00000298307 | KLHDC2 | 0.4684 |
| ENSP00000384164 | KIF16B | 0.4684 |
| ENSP00000260731 | KIF11 | 0.4684 |
| ENSP00000415769 | ITIH3 | 0.4684 |
| ENSP00000074304 | INPP4A | 0.4684 |
| ENSP00000313243 | HSF5 | 0.4684 |
| ENSP00000350275 | HIST1H3A | 0.4684 |
| ENSP00000289316 | HIST1H2BD | 0.4684 |
| ENSP00000313699 | HEPHL1 | 0.4684 |
| ENSP00000369654 | HBD | 0.4684 |
| ENSP00000364580 | GRTP1 | 0.4684 |
| ENSP00000344659 | GLB1L2 | 0.4684 |
| ENSP00000467494 | GIPR | 0.4684 |
| ENSP00000362344 | FPGS | 0.4684 |
| ENSP00000291744 | FCN2 | 0.4684 |
| ENSP00000320828 | EPS8L2 | 0.4684 |
| ENSP00000345259 | EPB41 | 0.4684 |
| ENSP00000414851 | ELP2 | 0.4684 |
| ENSP00000402634 | ELOVL7 | 0.4684 |
| ENSP00000269466 | ELAC1 | 0.4684 |
| ENSP00000362314 | EIF4EBP2 | 0.4684 |
| ENSP00000369038 | EEF1E1 | 0.4684 |
| ENSP00000376855 | DPP10 | 0.4684 |
| ENSP00000361625 | DOLPP1 | 0.4684 |
| ENSP00000276440 | DOCK5 | 0.4684 |
| ENSP00000374490 | DNAH17 | 0.4684 |
| ENSP00000428480 | DLX6 | 0.4684 |
| ENSP00000300087 | DCTN5 | 0.4684 |
| ENSP00000301645 | CYP7A1 | 0.4684 |
| ENSP00000324648 | CYP2B6 | 0.4684 |
| ENSP00000258415 | CYP27A1 | 0.4684 |
| ENSP00000407724 | CSDE1 | 0.4684 |
| ENSP00000302485 | CSDC2 | 0.4684 |
| ENSP00000360541 | CPT2 | 0.4684 |
| ENSP00000316948 | CLK4 | 0.4684 |
| ENSP00000223398 | CLIP2 | 0.4684 |
| ENSP00000324205 | CHMP4A | 0.4684 |
| ENSP00000361151 | CEL | 0.4684 |
| ENSP00000234170 | CEBPZ | 0.4684 |
| ENSP00000383611 | CCNL2 | 0.4684 |
| ENSP00000311847 | CARHSP1 | 0.4684 |
| ENSP00000258418 | CAB39 | 0.4684 |
| ENSP00000331127 | BOLA2 | 0.4684 |
| ENSP00000322991 | BCAT2 | 0.4684 |
| ENSP00000369055 | B4GALT1 | 0.4684 |
| ENSP00000335203 | ATPIF1 | 0.4684 |

| | | |
|---|---|---|
| ENSP00000358777 | ATP6AP1 | 0.4684 |
| ENSP00000385057 | APOBEC3G | 0.4684 |
| ENSP00000310146 | AP000769.1 | 0.4684 |
| ENSP00000253799 | AOC2 | 0.4684 |
| ENSP00000314004 | ANAPC2 | 0.4684 |
| ENSP00000297785 | ALDH1A1 | 0.4684 |
| ENSP00000315615 | AKAP5 | 0.4684 |
| ENSP00000312370 | AIFM2 | 0.4684 |
| ENSP00000285518 | AGPAT5 | 0.4684 |
| ENSP00000380413 | AGAP3 | 0.4684 |
| ENSP00000306606 | ADH1B | 0.4684 |
| ENSP00000264758 | ADD1 | 0.4684 |
| ENSP00000265846 | ADAP1 | 0.4684 |
| ENSP00000333037 | ACTRT3 | 0.4684 |
| ENSP00000354841 | ABHD14B | 0.4684 |
| ENSP00000362174 | ZC3H12A | 0.4684 |
| ENSP00000420227 | RPS4XP21 | 0.4684 |
| ENSP00000200135 | ZW10 | 0.4658 |
| ENSP00000352233 | ZNF705A | 0.4658 |
| ENSP00000331462 | ZNF704 | 0.4658 |
| ENSP00000237937 | ZFAND5 | 0.4658 |
| ENSP00000341681 | ZDHHC7 | 0.4658 |
| ENSP00000365130 | ZCCHC6 | 0.4658 |
| ENSP00000253048 | ZC3H4 | 0.4658 |
| ENSP00000384222 | WWC2 | 0.4658 |
| ENSP00000267973 | WDR61 | 0.4658 |
| ENSP00000371532 | VLDLR | 0.4658 |
| ENSP00000387019 | UXS1 | 0.4658 |
| ENSP00000312981 | USP39 | 0.4658 |
| ENSP00000301831 | ULK4 | 0.4658 |
| ENSP00000290650 | UBR1 | 0.4658 |
| ENSP00000386935 | UAP1L1 | 0.4658 |
| ENSP00000182527 | TRAM2 | 0.4658 |
| ENSP00000344724 | TOX2 | 0.4658 |
| ENSP00000281834 | TNFSF4 | 0.4658 |
| ENSP00000331288 | TMEM173 | 0.4658 |
| ENSP00000359305 | TMED5 | 0.4658 |
| ENSP00000339730 | THBS4 | 0.4658 |
| ENSP00000238682 | TGFB3 | 0.4658 |
| ENSP00000334785 | TESC | 0.4658 |
| ENSP00000350937 | TES | 0.4658 |
| ENSP00000365962 | TBC1D25 | 0.4658 |
| ENSP00000303522 | TACR1 | 0.4658 |
| ENSP00000316130 | SYNE4 | 0.4658 |
| ENSP00000384015 | SUN1 | 0.4658 |
| ENSP00000341938 | SUMF2 | 0.4658 |
| ENSP00000365530 | STXBP4 | 0.4658 |
| ENSP00000330138 | SSTR3 | 0.4658 |
| ENSP00000338191 | SNTB2 | 0.4658 |
| ENSP00000356687 | SLC9C2 | 0.4658 |
| ENSP00000280612 | SLC7A11 | 0.4658 |
| ENSP00000310208 | SLC5A6 | 0.4658 |
| ENSP00000273063 | SLC4A3 | 0.4658 |

| | | |
|---|---|---|
| ENSP00000234800 | SLC35E2B | 0.4658 |
| ENSP00000381399 | SLC2A11 | 0.4658 |
| ENSP00000345873 | SLC26A3 | 0.4658 |
| ENSP00000275233 | SHPRH | 0.4658 |
| ENSP00000361640 | SH3GLB2 | 0.4658 |
| ENSP00000362298 | SGPL1 | 0.4658 |
| ENSP00000397082 | SFTPA1 | 0.4658 |
| ENSP00000233468 | SF3B14 | 0.4658 |
| ENSP00000426234 | SEPHS2 | 0.4658 |
| ENSP00000370288 | SELO | 0.4658 |
| ENSP00000019103 | SCTR | 0.4658 |
| ENSP00000369899 | RRAGA | 0.4658 |
| ENSP00000362532 | RPRD1B | 0.4658 |
| ENSP00000463847 | RPL26 | 0.4658 |
| ENSP00000359368 | RPAP2 | 0.4658 |
| ENSP00000273480 | RNF7 | 0.4658 |
| ENSP00000325677 | RNF40 | 0.4658 |
| ENSP00000254260 | RHPN2 | 0.4658 |
| ENSP00000217999 | RHOXF1 | 0.4658 |
| ENSP00000343656 | RAPGEF5 | 0.4658 |
| ENSP00000254695 | RAP1GAP2 | 0.4658 |
| ENSP00000350094 | QRICH1 | 0.4658 |
| ENSP00000363667 | PTPN3 | 0.4658 |
| ENSP00000254630 | PTCD3 | 0.4658 |
| ENSP00000452746 | PSTPIP1 | 0.4658 |
| ENSP00000215071 | PSMD8 | 0.4658 |
| ENSP00000373937 | PRTG | 0.4658 |
| ENSP00000367835 | PRPF18 | 0.4658 |
| ENSP00000331879 | PRMT3 | 0.4658 |
| ENSP00000303057 | PPIC | 0.4658 |
| ENSP00000350052 | POTEF | 0.4658 |
| ENSP00000299443 | POTED | 0.4658 |
| ENSP00000334564 | POLR3C | 0.4658 |
| ENSP00000358223 | PNLIP | 0.4658 |
| ENSP00000332225 | PMCH | 0.4658 |
| ENSP00000390111 | PLSCR5 | 0.4658 |
| ENSP00000382434 | PLA2G4E | 0.4658 |
| ENSP00000415203 | PIGG | 0.4658 |
| ENSP00000263038 | PHYH | 0.4658 |
| ENSP00000297373 | PHKG1 | 0.4658 |
| ENSP00000324248 | PENK | 0.4658 |
| ENSP00000342033 | PDC | 0.4658 |
| ENSP00000364597 | PADI4 | 0.4658 |
| ENSP00000320376 | P2RY13 | 0.4658 |
| ENSP00000280701 | OXSM | 0.4658 |
| ENSP00000349874 | NUDT13 | 0.4658 |
| ENSP00000287437 | NSMCE2 | 0.4658 |
| ENSP00000278612 | NPAT | 0.4658 |
| ENSP00000354387 | NMRK1 | 0.4658 |
| ENSP00000342300 | NELFCD | 0.4658 |
| ENSP00000392709 | NDUFS1 | 0.4658 |
| ENSP00000184266 | NDUFB4 | 0.4658 |
| ENSP00000296499 | NDST3 | 0.4658 |

| | | |
|---|---|---|
| ENSP00000310657 | NDST2 | 0.4658 |
| ENSP00000361454 | NCBP2L | 0.4658 |
| ENSP00000302441 | NANP | 0.4658 |
| ENSP00000452521 | NAA30 | 0.4658 |
| ENSP00000354849 | MYBPC1 | 0.4658 |
| ENSP00000310785 | MRPS22 | 0.4658 |
| ENSP00000312395 | MRPS21 | 0.4658 |
| ENSP00000275053 | MMS22L | 0.4658 |
| ENSP00000313881 | MKRN3 | 0.4658 |
| ENSP00000321070 | ME2 | 0.4658 |
| ENSP00000256178 | LYVE1 | 0.4658 |
| ENSP00000312001 | LRRN3 | 0.4658 |
| ENSP00000344364 | LRRC29 | 0.4658 |
| ENSP00000222307 | KXD1 | 0.4658 |
| ENSP00000330101 | KRT76 | 0.4658 |
| ENSP00000328236 | KNTC1 | 0.4658 |
| ENSP00000249776 | KNSTRN | 0.4658 |
| ENSP00000338130 | KLRD1 | 0.4658 |
| ENSP00000326159 | KLK4 | 0.4658 |
| ENSP00000385545 | KIF26B | 0.4658 |
| ENSP00000378356 | KIF20A | 0.4658 |
| ENSP00000339960 | KCNJ5 | 0.4658 |
| ENSP00000245787 | INSIG2 | 0.4658 |
| ENSP00000263383 | ILVBL | 0.4658 |
| ENSP00000330959 | IL1R2 | 0.4658 |
| ENSP00000302476 | HSPB2 | 0.4658 |
| ENSP00000335060 | HPDL | 0.4658 |
| ENSP00000361935 | HPCAL4 | 0.4658 |
| ENSP00000342886 | HIST1H2BJ | 0.4658 |
| ENSP00000244573 | HIST1H1A | 0.4658 |
| ENSP00000274787 | HIGD2A | 0.4658 |
| ENSP00000290765 | GSTT2B | 0.4658 |
| ENSP00000343428 | GPR18 | 0.4658 |
| ENSP00000363250 | GPN2 | 0.4658 |
| ENSP00000347206 | GPAA1 | 0.4658 |
| ENSP00000300873 | GNG8 | 0.4658 |
| ENSP00000229264 | GNB3 | 0.4658 |
| ENSP00000317379 | GLS | 0.4658 |
| ENSP00000411593 | GLRA1 | 0.4658 |
| ENSP00000362460 | GJB3 | 0.4658 |
| ENSP00000336914 | FGD3 | 0.4658 |
| ENSP00000441093 | FDX1L | 0.4658 |
| ENSP00000294800 | FCGR3B | 0.4658 |
| ENSP00000344866 | FBXL5 | 0.4658 |
| ENSP00000224862 | FBXL15 | 0.4658 |
| ENSP00000188790 | FAP | 0.4658 |
| ENSP00000402825 | FAM197Y1 | 0.4658 |
| ENSP00000360843 | FAF1 | 0.4658 |
| ENSP00000287675 | EXOG | 0.4658 |
| ENSP00000436337 | EPPK1 | 0.4658 |
| ENSP00000317431 | ENTHD1 | 0.4658 |
| ENSP00000230565 | ENPP5 | 0.4658 |
| ENSP00000265162 | ENPEP | 0.4658 |

| | | |
|---|---|---|
| ENSP00000358831 | ELOVL4 | 0.4658 |
| ENSP00000470059 | ECH1 | 0.4658 |
| ENSP00000356056 | DYNLT1 | 0.4658 |
| ENSP00000367001 | DPP6 | 0.4658 |
| ENSP00000357384 | DPM3 | 0.4658 |
| ENSP00000263697 | DNAJC8 | 0.4658 |
| ENSP00000316053 | DNAJB8 | 0.4658 |
| ENSP00000251642 | DHX58 | 0.4658 |
| ENSP00000350698 | DDX47 | 0.4658 |
| ENSP00000454699 | DDX19B | 0.4658 |
| ENSP00000216862 | CYP24A1 | 0.4658 |
| ENSP00000458537 | CTRL | 0.4658 |
| ENSP00000470389 | CTD-2287O16.3 | 0.4658 |
| ENSP00000243914 | CTCFL | 0.4658 |
| ENSP00000346805 | CRYBA4 | 0.4658 |
| ENSP00000358460 | CLIC2 | 0.4658 |
| ENSP00000355922 | CENPF | 0.4658 |
| ENSP00000280614 | CCRN4L | 0.4658 |
| ENSP00000302227 | CCBL1 | 0.4658 |
| ENSP00000290354 | CBR3 | 0.4658 |
| ENSP00000395359 | CADM1 | 0.4658 |
| ENSP00000349320 | CACNA2D1 | 0.4658 |
| ENSP00000352011 | CACNA1G | 0.4658 |
| ENSP00000419765 | BRD9 | 0.4658 |
| ENSP00000403524 | BRAP | 0.4658 |
| ENSP00000308334 | ATP5S | 0.4658 |
| ENSP00000332756 | ATP11C | 0.4658 |
| ENSP00000257254 | APLNR | 0.4658 |
| ENSP00000359096 | AMY1C | 0.4658 |
| ENSP00000315417 | ALX1 | 0.4658 |
| ENSP00000290953 | AGRP | 0.4658 |
| ENSP00000449241 | AGAP2 | 0.4658 |
| ENSP00000264436 | ADD2 | 0.4658 |
| ENSP00000342609 | ACER2 | 0.4658 |
| ENSP00000261377 | AC004381.6 | 0.4658 |
| ENSP00000295962 | ABHD6 | 0.4658 |
| ENSP00000218104 | ABCD1 | 0.4658 |
| ENSP00000290575 | C1R | 0.4658 |
| ENSP00000306381 | SMGA | 0.4658 |
| ENSP00000361845 | ZMPSTE24 | 0.4632 |
| ENSP00000324274 | ZCCHC10 | 0.4632 |
| ENSP00000367299 | VPS36 | 0.4632 |
| ENSP00000357040 | VANGL2 | 0.4632 |
| ENSP00000296792 | UTP15 | 0.4632 |
| ENSP00000300896 | USP32 | 0.4632 |
| ENSP00000192788 | UHRF1BP1 | 0.4632 |
| ENSP00000334044 | UBL4B | 0.4632 |
| ENSP00000355599 | TSNAX | 0.4632 |
| ENSP00000397435 | TSC22D1 | 0.4632 |
| ENSP00000390478 | TREX1 | 0.4632 |
| ENSP00000353165 | TPK1 | 0.4632 |
| ENSP00000229270 | TPI1 | 0.4632 |
| ENSP00000206765 | TGM1 | 0.4632 |

| | | |
|---|---|---|
| ENSP00000391806 | TAP2 | 0.4632 |
| ENSP00000352678 | SUPV3L1 | 0.4632 |
| ENSP00000319323 | STOML1 | 0.4632 |
| ENSP00000342181 | SRP72 | 0.4632 |
| ENSP00000312066 | SRP68 | 0.4632 |
| ENSP00000359484 | SMAP1 | 0.4632 |
| ENSP00000344648 | SLC37A1 | 0.4632 |
| ENSP00000313318 | SLC35C1 | 0.4632 |
| ENSP00000262462 | SLC27A6 | 0.4632 |
| ENSP00000360076 | SGIP1 | 0.4632 |
| ENSP00000264893 | SEPT11 | 0.4632 |
| ENSP00000332407 | SDR42E1 | 0.4632 |
| ENSP00000286835 | SCAF4 | 0.4632 |
| ENSP00000354045 | RRBP1 | 0.4632 |
| ENSP00000358497 | RNGTT | 0.4632 |
| ENSP00000322242 | RNF10 | 0.4632 |
| ENSP00000397181 | RGS4 | 0.4632 |
| ENSP00000262633 | RBM42 | 0.4632 |
| ENSP00000450687 | RAX2 | 0.4632 |
| ENSP00000264926 | RAD18 | 0.4632 |
| ENSP00000348722 | PUS7 | 0.4632 |
| ENSP00000376758 | PTRH2 | 0.4632 |
| ENSP00000225426 | PSMB3 | 0.4632 |
| ENSP00000276616 | PSKH2 | 0.4632 |
| ENSP00000369634 | PRPF40B | 0.4632 |
| ENSP00000348010 | PRPF39 | 0.4632 |
| ENSP00000412324 | PPP2R5C | 0.4632 |
| ENSP00000264977 | PPP2R3A | 0.4632 |
| ENSP00000295908 | PPM1K | 0.4632 |
| ENSP00000361302 | POMT1 | 0.4632 |
| ENSP00000420176 | POLE4 | 0.4632 |
| ENSP00000296484 | POC1A | 0.4632 |
| ENSP00000279036 | PIGT | 0.4632 |
| ENSP00000378451 | PHF15 | 0.4632 |
| ENSP00000438465 | PGAM5 | 0.4632 |
| ENSP00000407401 | PEX5 | 0.4632 |
| ENSP00000358312 | PEX11B | 0.4632 |
| ENSP00000326581 | PEG3 | 0.4632 |
| ENSP00000284770 | PDLIM3 | 0.4632 |
| ENSP00000223061 | PCOLCE | 0.4632 |
| ENSP00000315693 | PAM16 | 0.4632 |
| ENSP00000359085 | NTNG1 | 0.4632 |
| ENSP00000340998 | NTN4 | 0.4632 |
| ENSP00000357669 | NPR1 | 0.4632 |
| ENSP00000319377 | NLRP12 | 0.4632 |
| ENSP00000258829 | NKX2-8 | 0.4632 |
| ENSP00000367301 | NDP | 0.4632 |
| ENSP00000406861 | MUC6 | 0.4632 |
| ENSP00000355388 | MPHOSPH8 | 0.4632 |
| ENSP00000328694 | MOB2 | 0.4632 |
| ENSP00000456163 | MIR3654 | 0.4632 |
| ENSP00000378957 | MFAP4 | 0.4632 |
| ENSP00000385481 | METTL21A | 0.4632 |

| | | |
|---|---|---|
| ENSP00000268711 | MED9 | 0.4632 |
| ENSP00000265350 | MED20 | 0.4632 |
| ENSP00000293777 | MED11 | 0.4632 |
| ENSP00000256429 | MBD2 | 0.4632 |
| ENSP00000219782 | MAZ | 0.4632 |
| ENSP00000383690 | MASP2 | 0.4632 |
| ENSP00000252622 | LSM7 | 0.4632 |
| ENSP00000386357 | LRRTM4 | 0.4632 |
| ENSP00000326870 | LRRC48 | 0.4632 |
| ENSP00000363275 | LRRC18 | 0.4632 |
| ENSP00000381490 | LARP4 | 0.4632 |
| ENSP00000354878 | KIF21A | 0.4632 |
| ENSP00000297404 | KCNV1 | 0.4632 |
| ENSP00000257981 | KCNH3 | 0.4632 |
| ENSP00000252321 | KCNA5 | 0.4632 |
| ENSP00000284262 | JPH3 | 0.4632 |
| ENSP00000314807 | IL1RAP | 0.4632 |
| ENSP00000431254 | IGLL5 | 0.4632 |
| ENSP00000329991 | IFNL1 | 0.4632 |
| ENSP00000359444 | HSFX1 | 0.4632 |
| ENSP00000316786 | HSD11B2 | 0.4632 |
| ENSP00000296522 | HPGD | 0.4632 |
| ENSP00000361927 | HNRNPH2 | 0.4632 |
| ENSP00000330074 | HIST1H1B | 0.4632 |
| ENSP00000246551 | HCST | 0.4632 |
| ENSP00000381666 | GXYLT1 | 0.4632 |
| ENSP00000274400 | GTF2H2 | 0.4632 |
| ENSP00000347251 | GPS1 | 0.4632 |
| ENSP00000287020 | GDF6 | 0.4632 |
| ENSP00000318690 | GCFC2 | 0.4632 |
| ENSP00000377372 | GAP43 | 0.4632 |
| ENSP00000413152 | FKBP7 | 0.4632 |
| ENSP00000342858 | FERMT2 | 0.4632 |
| ENSP00000266087 | FBXO7 | 0.4632 |
| ENSP00000454380 | FAM173A | 0.4632 |
| ENSP00000356771 | F5 | 0.4632 |
| ENSP00000366702 | ERRFI1 | 0.4632 |
| ENSP00000318066 | ENPP4 | 0.4632 |
| ENSP00000262726 | EFCAB6 | 0.4632 |
| ENSP00000366416 | EDNRB | 0.4632 |
| ENSP00000430241 | EBF2 | 0.4632 |
| ENSP00000226004 | DUSP3 | 0.4632 |
| ENSP00000377910 | DUSP14 | 0.4632 |
| ENSP00000417548 | DNAJC24 | 0.4632 |
| ENSP00000190165 | DMRT3 | 0.4632 |
| ENSP00000378887 | DHRS7B | 0.4632 |
| ENSP00000380495 | DDX51 | 0.4632 |
| ENSP00000356814 | DCAF6 | 0.4632 |
| ENSP00000323663 | DBF4B | 0.4632 |
| ENSP00000413493 | CPSF3L | 0.4632 |
| ENSP00000312587 | COQ10A | 0.4632 |
| ENSP00000229251 | COPS7A | 0.4632 |
| ENSP00000361834 | COL9A2 | 0.4632 |

| | | |
|---|---|---|
| ENSP00000229195 | CNOT2 | 0.4632 |
| ENSP00000252456 | CNN1 | 0.4632 |
| ENSP00000354609 | CNKSR1 | 0.4632 |
| ENSP00000409378 | CHRM4 | 0.4632 |
| ENSP00000380998 | CHMP1A | 0.4632 |
| ENSP00000295767 | CHCHD4 | 0.4632 |
| ENSP00000325485 | CERS5 | 0.4632 |
| ENSP00000268383 | CDR2 | 0.4632 |
| ENSP00000181839 | CDK13 | 0.4632 |
| ENSP00000304903 | CD2BP2 | 0.4632 |
| ENSP00000290349 | CBR1 | 0.4632 |
| ENSP00000258947 | CALCOCO2 | 0.4632 |
| ENSP00000396688 | C4A | 0.4632 |
| ENSP00000466121 | C19orf40 | 0.4632 |
| ENSP00000380598 | BLOC1S5 | 0.4632 |
| ENSP00000398076 | ATP9B | 0.4632 |
| ENSP00000356789 | ATP1B1 | 0.4632 |
| ENSP00000287394 | ATAD2 | 0.4632 |
| ENSP00000352995 | ARHGEF18 | 0.4632 |
| ENSP00000355026 | ARHGEF15 | 0.4632 |
| ENSP00000217456 | APMAP | 0.4632 |
| ENSP00000265140 | ANKRD32 | 0.4632 |
| ENSP00000459775 | ANKFY1 | 0.4632 |
| ENSP00000330484 | AMY1B | 0.4632 |
| ENSP00000262844 | AMMECR1 | 0.4632 |
| ENSP00000363512 | ALOX5 | 0.4632 |
| ENSP00000360761 | AGPAT2 | 0.4632 |
| ENSP00000328938 | AFMID | 0.4632 |
| ENSP00000420487 | ABHD14A-ACY1 | 0.4632 |
| ENSP00000202788 | MAPKAPK5 | 0.4632 |
| ENSP00000415634 | ENSG00000248354 | 0.4632 |
| ENSP00000382987 | ZNF705B | 0.4605 |
| ENSP00000311596 | ZNF699 | 0.4605 |
| ENSP00000386488 | ZC3H8 | 0.4605 |
| ENSP00000007699 | YBX2 | 0.4605 |
| ENSP00000334808 | USP16 | 0.4605 |
| ENSP00000293218 | UNK | 0.4605 |
| ENSP00000406022 | UNC5C | 0.4605 |
| ENSP00000464265 | UBBP4 | 0.4605 |
| ENSP00000356903 | UAP1 | 0.4605 |
| ENSP00000225576 | TVP23C | 0.4605 |
| ENSP00000370007 | TMSB4X | 0.4605 |
| ENSP00000404042 | TMED4 | 0.4605 |
| ENSP00000279263 | TM7SF2 | 0.4605 |
| ENSP00000254942 | TERF2 | 0.4605 |
| ENSP00000354633 | TAF11 | 0.4605 |
| ENSP00000356236 | SYT2 | 0.4605 |
| ENSP00000332818 | SV2B | 0.4605 |
| ENSP00000319192 | STK17A | 0.4605 |
| ENSP00000301463 | SPRYD3 | 0.4605 |
| ENSP00000332488 | SPPL2C | 0.4605 |
| ENSP00000322568 | SOX11 | 0.4605 |
| ENSP00000222990 | SNX8 | 0.4605 |

| | | |
|---|---|---|
| ENSP00000229729 | SLC44A4 | 0.4605 |
| ENSP00000355920 | SLC22A2 | 0.4605 |
| ENSP00000321735 | SLC16A8 | 0.4605 |
| ENSP00000305976 | SIK2 | 0.4605 |
| ENSP00000373627 | SEPT14 | 0.4605 |
| ENSP00000305675 | SDPR | 0.4605 |
| ENSP00000333537 | SALL2 | 0.4605 |
| ENSP00000357301 | RXFP4 | 0.4605 |
| ENSP00000274811 | RNF44 | 0.4605 |
| ENSP00000313350 | RNASEH1 | 0.4605 |
| ENSP00000232219 | RBP1 | 0.4605 |
| ENSP00000288976 | PTPDC1 | 0.4605 |
| ENSP00000275605 | PSPH | 0.4605 |
| ENSP00000384848 | PRR5 | 0.4605 |
| ENSP00000359042 | PRPF38B | 0.4605 |
| ENSP00000252455 | PRKCSH | 0.4605 |
| ENSP00000228437 | PRDM4 | 0.4605 |
| ENSP00000278070 | PPRC1 | 0.4605 |
| ENSP00000238994 | PPP1R3C | 0.4605 |
| ENSP00000393845 | PPIAL4G | 0.4605 |
| ENSP00000242248 | POLM | 0.4605 |
| ENSP00000299206 | POLL | 0.4605 |
| ENSP00000265421 | POLB | 0.4605 |
| ENSP00000330634 | PKD1P1 | 0.4605 |
| ENSP00000365757 | PIP4K2A | 0.4605 |
| ENSP00000296029 | PF4 | 0.4605 |
| ENSP00000274311 | PELO | 0.4605 |
| ENSP00000420927 | PDK2 | 0.4605 |
| ENSP00000370345 | PAN3 | 0.4605 |
| ENSP00000442349 | P2RX7 | 0.4605 |
| ENSP00000339389 | OTUD6A | 0.4605 |
| ENSP00000265572 | OPRK1 | 0.4605 |
| ENSP00000361141 | OPN4 | 0.4605 |
| ENSP00000356708 | OLIG3 | 0.4605 |
| ENSP00000318029 | ODF3L2 | 0.4605 |
| ENSP00000331843 | NPIP | 0.4605 |
| ENSP00000360412 | NOC3L | 0.4605 |
| ENSP00000343479 | NBR1 | 0.4605 |
| ENSP00000442795 | MYBPC3 | 0.4605 |
| ENSP00000245185 | MT2A | 0.4605 |
| ENSP00000431538 | MPZ | 0.4605 |
| ENSP00000445920 | MMAB | 0.4605 |
| ENSP00000314343 | MED29 | 0.4605 |
| ENSP00000359925 | LRRC1 | 0.4605 |
| ENSP00000262290 | LPO | 0.4605 |
| ENSP00000357731 | LOR | 0.4605 |
| ENSP00000261292 | LIPG | 0.4605 |
| ENSP00000318423 | LINS | 0.4605 |
| ENSP00000378400 | LIMA1 | 0.4605 |
| ENSP00000221421 | LHB | 0.4605 |
| ENSP00000371886 | JMJD7-PLA2G4B | 0.4605 |
| ENSP00000260386 | ITPKA | 0.4605 |
| ENSP00000259239 | IMP4 | 0.4605 |

| | | |
|---|---|---|
| ENSP00000360891 | IFIT2 | 0.4605 |
| ENSP00000370293 | HUS1B | 0.4605 |
| ENSP00000387564 | HMCN2 | 0.4605 |
| ENSP00000358155 | HIST2H2AA4 | 0.4605 |
| ENSP00000274764 | HIST1H2BA | 0.4605 |
| ENSP00000366679 | HIST1H2AH | 0.4605 |
| ENSP00000215780 | GSTT2 | 0.4605 |
| ENSP00000370104 | GPS2 | 0.4605 |
| ENSP00000303942 | GP9 | 0.4605 |
| ENSP00000258145 | GNS | 0.4605 |
| ENSP00000355607 | GNPAT | 0.4605 |
| ENSP00000310447 | GLS2 | 0.4605 |
| ENSP00000268797 | GFOD2 | 0.4605 |
| ENSP00000368589 | GFOD1 | 0.4605 |
| ENSP00000319531 | GCSH | 0.4605 |
| ENSP00000265643 | GAL | 0.4605 |
| ENSP00000292079 | FXYD2 | 0.4605 |
| ENSP00000309823 | FOXP4 | 0.4605 |
| ENSP00000256999 | FOLH1 | 0.4605 |
| ENSP00000254090 | FMO5 | 0.4605 |
| ENSP00000387471 | FAM96B | 0.4605 |
| ENSP00000250056 | FAM64A | 0.4605 |
| ENSP00000295834 | FABP1 | 0.4605 |
| ENSP00000356170 | ETNK2 | 0.4605 |
| ENSP00000347978 | ERCC5 | 0.4605 |
| ENSP00000351811 | EDC4 | 0.4605 |
| ENSP00000340742 | DOCK7 | 0.4605 |
| ENSP00000308312 | DNAI2 | 0.4605 |
| ENSP00000251312 | DHRS11 | 0.4605 |
| ENSP00000363071 | DES | 0.4605 |
| ENSP00000362727 | DENND1A | 0.4605 |
| ENSP00000003100 | CYP51A1 | 0.4605 |
| ENSP00000321821 | CYP4F12 | 0.4605 |
| ENSP00000376623 | CSH2 | 0.4605 |
| ENSP00000370966 | CRYZL1 | 0.4605 |
| ENSP00000325660 | CNTN1 | 0.4605 |
| ENSP00000317439 | CNPY1 | 0.4605 |
| ENSP00000286808 | CLDN17 | 0.4605 |
| ENSP00000310440 | CHMP2A | 0.4605 |
| ENSP00000337289 | CENPQ | 0.4605 |
| ENSP00000164024 | CELSR3 | 0.4605 |
| ENSP00000308815 | CCL19 | 0.4605 |
| ENSP00000325355 | CASKIN2 | 0.4605 |
| ENSP00000340329 | CAPRIN1 | 0.4605 |
| ENSP00000261669 | CAB39L | 0.4605 |
| ENSP00000342032 | BPGM | 0.4605 |
| ENSP00000267012 | BIN2 | 0.4605 |
| ENSP00000281474 | BICD1 | 0.4605 |
| ENSP00000309132 | BCL2L14 | 0.4605 |
| ENSP00000439128 | BANF2 | 0.4605 |
| ENSP00000359630 | BAI3 | 0.4605 |
| ENSP00000313171 | ATAD5 | 0.4605 |
| ENSP00000450040 | ASPG | 0.4605 |

| | | |
|---|---|---|
| ENSP00000264245 | ARHGAP31 | 0.4605 |
| ENSP00000261783 | ARG2 | 0.4605 |
| ENSP00000205948 | APOH | 0.4605 |
| ENSP00000355929 | ANGEL2 | 0.4605 |
| ENSP00000351327 | AKAP4 | 0.4605 |
| ENSP00000265602 | AHI1 | 0.4605 |
| ENSP00000369921 | ADAMTSL1 | 0.4605 |
| ENSP00000416706 | ACTBL2 | 0.4605 |
| ENSP00000409267 | AC006156.1 | 0.4605 |
| ENSP00000359245 | ABCA4 | 0.4605 |
| ENSP00000313674 | AAR2 | 0.4605 |
| ENSP00000373698 | FBXW4 | 0.4605 |
| ENSP00000325018 | ZNF569 | 0.4579 |
| ENSP00000321209 | ZNF274 | 0.4579 |
| ENSP00000333253 | ZC3H11A | 0.4579 |
| ENSP00000323678 | ZADH2 | 0.4579 |
| ENSP00000253031 | YIPF2 | 0.4579 |
| ENSP00000427687 | WDR1 | 0.4579 |
| ENSP00000356602 | VTA1 | 0.4579 |
| ENSP00000253794 | VPS25 | 0.4579 |
| ENSP00000348455 | UVRAG | 0.4579 |
| ENSP00000350009 | USP33 | 0.4579 |
| ENSP00000304811 | UGT2B7 | 0.4579 |
| ENSP00000259253 | UGGT1 | 0.4579 |
| ENSP00000262209 | TRPA1 | 0.4579 |
| ENSP00000345719 | TOR1A | 0.4579 |
| ENSP00000268957 | TOB1 | 0.4579 |
| ENSP00000266732 | TMPO | 0.4579 |
| ENSP00000272521 | TMEM177 | 0.4579 |
| ENSP00000389399 | TMCO3 | 0.4579 |
| ENSP00000318115 | TIMM50 | 0.4579 |
| ENSP00000349781 | TBC1D8B | 0.4579 |
| ENSP00000263974 | TAF12 | 0.4579 |
| ENSP00000246895 | STATH | 0.4579 |
| ENSP00000364665 | SSX2B | 0.4579 |
| ENSP00000287641 | SST | 0.4579 |
| ENSP00000352936 | SPINK5 | 0.4579 |
| ENSP00000465075 | SPC24 | 0.4579 |
| ENSP00000370200 | SNAPC3 | 0.4579 |
| ENSP00000441269 | SLCO1B3 | 0.4579 |
| ENSP00000372390 | SLC7A4 | 0.4579 |
| ENSP00000429200 | SLC25A37 | 0.4579 |
| ENSP00000361666 | SLC22A7 | 0.4579 |
| ENSP00000357844 | SLC16A10 | 0.4579 |
| ENSP00000262461 | SLC12A2 | 0.4579 |
| ENSP00000216540 | SLC10A1 | 0.4579 |
| ENSP00000264382 | SI | 0.4579 |
| ENSP00000299502 | SERPINB2 | 0.4579 |
| ENSP00000371936 | SEMA5A | 0.4579 |
| ENSP00000341538 | SEC61G | 0.4579 |
| ENSP00000245923 | RTN2 | 0.4579 |
| ENSP00000276659 | RSPO2 | 0.4579 |
| ENSP00000232888 | RRP9 | 0.4579 |

| | | |
|---|---|---|
| ENSP00000417464 | RRP1 | 0.4579 |
| ENSP00000347271 | RPS10 | 0.4579 |
| ENSP00000293842 | RPL26 | 0.4579 |
| ENSP00000431165 | RP11-192H23.4 | 0.4579 |
| ENSP00000342121 | RNF6 | 0.4579 |
| ENSP00000302324 | RNASE3 | 0.4579 |
| ENSP00000362863 | REEP3 | 0.4579 |
| ENSP00000339090 | RBMX2 | 0.4579 |
| ENSP00000362283 | PTPRT | 0.4579 |
| ENSP00000357196 | PTPRK | 0.4579 |
| ENSP00000225992 | PPY | 0.4579 |
| ENSP00000312411 | PPM1E | 0.4579 |
| ENSP00000439444 | PPIAL4D | 0.4579 |
| ENSP00000334840 | PNRC2 | 0.4579 |
| ENSP00000323511 | PNKP | 0.4579 |
| ENSP00000290472 | PLA2G4D | 0.4579 |
| ENSP00000398824 | PHF21A | 0.4579 |
| ENSP00000258662 | NUDT15 | 0.4579 |
| ENSP00000296802 | NSA2 | 0.4579 |
| ENSP00000361214 | NRG3 | 0.4579 |
| ENSP00000349041 | NRARP | 0.4579 |
| ENSP00000264218 | NMU | 0.4579 |
| ENSP00000252711 | NDUFA10 | 0.4579 |
| ENSP00000239926 | MYOT | 0.4579 |
| ENSP00000417498 | MUC4 | 0.4579 |
| ENSP00000237380 | MED28 | 0.4579 |
| ENSP00000318604 | MAF1 | 0.4579 |
| ENSP00000235310 | MAD2L2 | 0.4579 |
| ENSP00000365040 | LY6G5B | 0.4579 |
| ENSP00000302456 | KLF13 | 0.4579 |
| ENSP00000259711 | KIF13A | 0.4579 |
| ENSP00000370710 | KDM4C | 0.4579 |
| ENSP00000355580 | KCNK1 | 0.4579 |
| ENSP00000376432 | KCNJ1 | 0.4579 |
| ENSP00000463533 | KCNH6 | 0.4579 |
| ENSP00000346453 | IPO4 | 0.4579 |
| ENSP00000408526 | IMPA1 | 0.4579 |
| ENSP00000360869 | IFIT1 | 0.4579 |
| ENSP00000359393 | HMGB3 | 0.4579 |
| ENSP00000378786 | HLA-DRA | 0.4579 |
| ENSP00000350580 | HIST1H2BB | 0.4579 |
| ENSP00000420168 | GSTA2 | 0.4579 |
| ENSP00000378492 | GRM2 | 0.4579 |
| ENSP00000222803 | FKBP14 | 0.4579 |
| ENSP00000361669 | ESX1 | 0.4579 |
| ENSP00000353270 | ERGIC2 | 0.4579 |
| ENSP00000308472 | EIF4EBP3 | 0.4579 |
| ENSP00000394869 | EIF2B4 | 0.4579 |
| ENSP00000370614 | DPF3 | 0.4579 |
| ENSP00000266037 | DOCK3 | 0.4579 |
| ENSP00000365991 | DNAJC3 | 0.4579 |
| ENSP00000238146 | DDX55 | 0.4579 |
| ENSP00000297579 | DCAF13 | 0.4579 |

| | | |
|---|---|---|
| ENSP00000337065 | CXCL14 | 0.4579 |
| ENSP00000367064 | CUBN | 0.4579 |
| ENSP00000312506 | CSPG4 | 0.4579 |
| ENSP00000397441 | COG6 | 0.4579 |
| ENSP00000355572 | COA6 | 0.4579 |
| ENSP00000315130 | CLU | 0.4579 |
| ENSP00000354856 | CLK2 | 0.4579 |
| ENSP00000185206 | CLIC5 | 0.4579 |
| ENSP00000319984 | CHRM2 | 0.4579 |
| ENSP00000263780 | CHMP2B | 0.4579 |
| ENSP00000340210 | CD59 | 0.4579 |
| ENSP00000292301 | CCR2 | 0.4579 |
| ENSP00000303525 | CBR4 | 0.4579 |
| ENSP00000298892 | CAPRIN2 | 0.4579 |
| ENSP00000419195 | CAMKV | 0.4579 |
| ENSP00000331746 | CALCA | 0.4579 |
| ENSP00000324960 | CABP4 | 0.4579 |
| ENSP00000294288 | CABP2 | 0.4579 |
| ENSP00000327440 | BMP8A | 0.4579 |
| ENSP00000363162 | ATP6V1G1 | 0.4579 |
| ENSP00000355173 | ATG9A | 0.4579 |
| ENSP00000370526 | ARSE | 0.4579 |
| ENSP00000472409 | ARL14EPL | 0.4579 |
| ENSP00000219660 | AQP8 | 0.4579 |
| ENSP00000318355 | AQP10 | 0.4579 |
| ENSP00000379133 | APIP | 0.4579 |
| ENSP00000454303 | AP5B1 | 0.4579 |
| ENSP00000360170 | ANGPTL3 | 0.4579 |
| ENSP00000291572 | AGPAT3 | 0.4579 |
| ENSP00000348381 | ADD3 | 0.4579 |
| ENSP00000296513 | ADAD1 | 0.4579 |
| ENSP00000254286 | ACTR10 | 0.4579 |
| ENSP00000252519 | ACE2 | 0.4579 |
| ENSP00000399696 | AC037459.4 | 0.4579 |
| ENSP00000272286 | ABCG8 | 0.4579 |
| ENSP00000352498 | WASH6P | 0.4579 |
| ENSP00000448888 | ENSG00000258210 | 0.4579 |
| ENSP00000378201 | ZBTB1 | 0.4553 |
| ENSP00000242796 | WDR83 | 0.4553 |
| ENSP00000320563 | WDFY4 | 0.4553 |
| ENSP00000272322 | VPS54 | 0.4553 |
| ENSP00000262291 | VMP1 | 0.4553 |
| ENSP00000227471 | UNC93B1 | 0.4553 |
| ENSP00000358478 | TTF2 | 0.4553 |
| ENSP00000357219 | TTC24 | 0.4553 |
| ENSP00000398163 | TSPY3 | 0.4553 |
| ENSP00000342222 | TRPV2 | 0.4553 |
| ENSP00000354204 | TOMM20L | 0.4553 |
| ENSP00000356298 | TMEM9 | 0.4553 |
| ENSP00000423933 | THUMPD2 | 0.4553 |
| ENSP00000255224 | SYT4 | 0.4553 |
| ENSP00000293695 | SYCE2 | 0.4553 |
| ENSP00000343645 | SULT1A3 | 0.4553 |

| | | |
|---|---|---|
| ENSP00000264228 | SRD5A3 | 0.4553 |
| ENSP00000282223 | SPOCK1 | 0.4553 |
| ENSP00000347314 | SPG20 | 0.4553 |
| ENSP00000219611 | SOLH | 0.4553 |
| ENSP00000355261 | SMG5 | 0.4553 |
| ENSP00000354574 | SLC9B2 | 0.4553 |
| ENSP00000261867 | SLC30A4 | 0.4553 |
| ENSP00000378756 | SLC16A9 | 0.4553 |
| ENSP00000319991 | SLC16A6 | 0.4553 |
| ENSP00000338171 | SKAP1 | 0.4553 |
| ENSP00000361400 | SFTPA2 | 0.4553 |
| ENSP00000345751 | SCNN1B | 0.4553 |
| ENSP00000381824 | SAC3D1 | 0.4553 |
| ENSP00000375853 | RGS13 | 0.4553 |
| ENSP00000370648 | RDH14 | 0.4553 |
| ENSP00000054950 | RCN1 | 0.4553 |
| ENSP00000341483 | RANBP3 | 0.4553 |
| ENSP00000311360 | RAD9A | 0.4553 |
| ENSP00000216840 | RABGGTA | 0.4553 |
| ENSP00000350934 | RABEP2 | 0.4553 |
| ENSP00000302327 | PYGO1 | 0.4553 |
| ENSP00000348472 | PXK | 0.4553 |
| ENSP00000387365 | PWP1 | 0.4553 |
| ENSP00000266688 | PTPRQ | 0.4553 |
| ENSP00000301974 | PTAFR | 0.4553 |
| ENSP00000295083 | PQLC3 | 0.4553 |
| ENSP00000377390 | PPP6R3 | 0.4553 |
| ENSP00000439146 | PPIAL4A | 0.4553 |
| ENSP00000329697 | PPAP2C | 0.4553 |
| ENSP00000392718 | POTEI | 0.4553 |
| ENSP00000439189 | POTEE | 0.4553 |
| ENSP00000457689 | POTEB | 0.4553 |
| ENSP00000402758 | PMEL | 0.4553 |
| ENSP00000247665 | PHPT1 | 0.4553 |
| ENSP00000311453 | PDE8A | 0.4553 |
| ENSP00000354950 | PCDH15 | 0.4553 |
| ENSP00000296220 | OSBPL11 | 0.4553 |
| ENSP00000264169 | ORC4 | 0.4553 |
| ENSP00000228928 | OAS3 | 0.4553 |
| ENSP00000368459 | NUFIP1 | 0.4553 |
| ENSP00000303575 | NUDT9 | 0.4553 |
| ENSP00000336528 | NR1I2 | 0.4553 |
| ENSP00000370883 | NOMO2 | 0.4553 |
| ENSP00000297990 | NOL6 | 0.4553 |
| ENSP00000351591 | NLGN3 | 0.4553 |
| ENSP00000396103 | MYT1L | 0.4553 |
| ENSP00000348821 | MYOM1 | 0.4553 |
| ENSP00000349977 | MVP | 0.4553 |
| ENSP00000258105 | MRPL53 | 0.4553 |
| ENSP00000354580 | MRPL21 | 0.4553 |
| ENSP00000302177 | MBOAT2 | 0.4553 |
| ENSP00000352835 | MB | 0.4553 |
| ENSP00000403208 | MAP10 | 0.4553 |

| | | |
|---|---|---|
| ENSP00000228740 | LTA4H | 0.4553 |
| ENSP00000321334 | LPA | 0.4553 |
| ENSP00000462730 | LMNB2 | 0.4553 |
| ENSP00000402301 | LAPTM4B | 0.4553 |
| ENSP00000377582 | KRTAP4-12 | 0.4553 |
| ENSP00000395323 | KCNIP1 | 0.4553 |
| ENSP00000328813 | KCNH8 | 0.4553 |
| ENSP00000262186 | KCNH2 | 0.4553 |
| ENSP00000348648 | JPH4 | 0.4553 |
| ENSP00000420419 | JAM2 | 0.4553 |
| ENSP00000354742 | IPO9 | 0.4553 |
| ENSP00000429065 | INTS9 | 0.4553 |
| ENSP00000380247 | HRH1 | 0.4553 |
| ENSP00000322706 | HMGCS1 | 0.4553 |
| ENSP00000355656 | HIST3H2A | 0.4553 |
| ENSP00000344504 | H1F0 | 0.4553 |
| ENSP00000216044 | GTPBP1 | 0.4553 |
| ENSP00000345282 | GPT2 | 0.4553 |
| ENSP00000392828 | GPSM1 | 0.4553 |
| ENSP00000339057 | GPRASP2 | 0.4553 |
| ENSP00000229314 | GOLT1B | 0.4553 |
| ENSP00000381340 | GGT5 | 0.4553 |
| ENSP00000359504 | GBP1 | 0.4553 |
| ENSP00000331358 | GAST | 0.4553 |
| ENSP00000254466 | GAS2L2 | 0.4553 |
| ENSP00000242257 | FTSJ2 | 0.4553 |
| ENSP00000340191 | FPR2 | 0.4553 |
| ENSP00000288670 | FMNL2 | 0.4553 |
| ENSP00000317232 | FKBP10 | 0.4553 |
| ENSP00000411145 | FEZF1 | 0.4553 |
| ENSP00000365944 | FBXO6 | 0.4553 |
| ENSP00000364742 | FBXO42 | 0.4553 |
| ENSP00000229971 | FBXL4 | 0.4553 |
| ENSP00000337477 | FAM60A | 0.4553 |
| ENSP00000367059 | ESPN | 0.4553 |
| ENSP00000258484 | EPC2 | 0.4553 |
| ENSP00000311545 | EMR1 | 0.4553 |
| ENSP00000273130 | DYNC1LI1 | 0.4553 |
| ENSP00000329539 | DUSP8 | 0.4553 |
| ENSP00000255108 | DPH2 | 0.4553 |
| ENSP00000246949 | DNASE1 | 0.4553 |
| ENSP00000216068 | DNAL4 | 0.4553 |
| ENSP00000284061 | DGKE | 0.4553 |
| ENSP00000265728 | DBF4 | 0.4553 |
| ENSP00000360247 | CYP2J2 | 0.4553 |
| ENSP00000228606 | CYP27B1 | 0.4553 |
| ENSP00000365116 | CTRC | 0.4553 |
| ENSP00000454861 | CTC-554D6.1 | 0.4553 |
| ENSP00000339399 | CRYZ | 0.4553 |
| ENSP00000299335 | COX11 | 0.4553 |
| ENSP00000272995 | COPS7B | 0.4553 |
| ENSP00000381949 | COL13A1 | 0.4553 |
| ENSP00000305459 | COG8 | 0.4553 |

| | | |
|---|---|---|
| ENSP00000328478 | CNGA2 | 0.4553 |
| ENSP00000405635 | CNEP1R1 | 0.4553 |
| ENSP00000305204 | CKAP2L | 0.4553 |
| ENSP00000299847 | CHRFAM7A | 0.4553 |
| ENSP00000229266 | CHPT1 | 0.4553 |
| ENSP00000275517 | CDCA5 | 0.4553 |
| ENSP00000323280 | CD6 | 0.4553 |
| ENSP00000320084 | CD276 | 0.4553 |
| ENSP00000262962 | CCDC94 | 0.4553 |
| ENSP00000216133 | CBX7 | 0.4553 |
| ENSP00000355837 | CAPN8 | 0.4553 |
| ENSP00000278559 | CAPN5 | 0.4553 |
| ENSP00000381758 | CAPN11 | 0.4553 |
| ENSP00000226021 | CACNG1 | 0.4553 |
| ENSP00000223642 | C5 | 0.4553 |
| ENSP00000332979 | BACE2 | 0.4553 |
| ENSP00000352408 | BABAM1 | 0.4553 |
| ENSP00000029410 | B4GALT7 | 0.4553 |
| ENSP00000421076 | ATP5L2 | 0.4553 |
| ENSP00000246747 | ARL2 | 0.4553 |
| ENSP00000264487 | AREG | 0.4553 |
| ENSP00000266594 | ANP32D | 0.4553 |
| ENSP00000296785 | ANKRA2 | 0.4553 |
| ENSP00000417764 | ALG2 | 0.4553 |
| ENSP00000223357 | AEBP1 | 0.4553 |
| ENSP00000253003 | ADRM1 | 0.4553 |
| ENSP00000072869 | ADCK2 | 0.4553 |
| ENSP00000384296 | ACY1 | 0.4553 |
| ENSP00000311224 | ACOT1 | 0.4553 |
| ENSP00000434480 | ACER3 | 0.4553 |
| ENSP00000464149 | ACE | 0.4553 |
| ENSP00000311436 | ABRA | 0.4553 |
| ENSP00000261200 | ABCC9 | 0.4553 |
| ENSP00000282326 | ZSCAN1 | 0.4526 |
| ENSP00000408108 | WDR26 | 0.4526 |
| ENSP00000295156 | VSNL1 | 0.4526 |
| ENSP00000356213 | VIP | 0.4526 |
| ENSP00000334329 | UNC5B | 0.4526 |
| ENSP00000268876 | UNC45B | 0.4526 |
| ENSP00000283628 | UBP1 | 0.4526 |
| ENSP00000387362 | UBE4A | 0.4526 |
| ENSP00000397453 | UBE2W | 0.4526 |
| ENSP00000314077 | TSPY2 | 0.4526 |
| ENSP00000369003 | TRPC4 | 0.4526 |
| ENSP00000350352 | TRMT1 | 0.4526 |
| ENSP00000328203 | TRAIP | 0.4526 |
| ENSP00000351214 | TOMM7 | 0.4526 |
| ENSP00000328207 | TNFRSF18 | 0.4526 |
| ENSP00000230461 | TMEM30A | 0.4526 |
| ENSP00000385995 | THADA | 0.4526 |
| ENSP00000251864 | TDRD1 | 0.4526 |
| ENSP00000347719 | TBCD | 0.4526 |
| ENSP00000345014 | TBC1D3G | 0.4526 |

| | | |
|---|---|---|
| ENSP00000236273 | SYF2 | 0.4526 |
| ENSP00000443988 | SUDS3 | 0.4526 |
| ENSP00000248600 | STYXL1 | 0.4526 |
| ENSP00000265044 | SSR3 | 0.4526 |
| ENSP00000347721 | SPNS3 | 0.4526 |
| ENSP00000366452 | SNX15 | 0.4526 |
| ENSP00000361803 | SMAP2 | 0.4526 |
| ENSP00000251303 | SLX1A | 0.4526 |
| ENSP00000394168 | SLCO1B7 | 0.4526 |
| ENSP00000265113 | SLC1A3 | 0.4526 |
| ENSP00000277010 | SIGMAR1 | 0.4526 |
| ENSP00000306473 | SHCBP1 | 0.4526 |
| ENSP00000362308 | SH3BGRL | 0.4526 |
| ENSP00000347117 | SEMA4A | 0.4526 |
| ENSP00000299333 | SCN3B | 0.4526 |
| ENSP00000324729 | SAV1 | 0.4526 |
| ENSP00000374280 | RTF1 | 0.4526 |
| ENSP00000462023 | RPL17 | 0.4526 |
| ENSP00000306123 | RPAP1 | 0.4526 |
| ENSP00000468280 | RP11-178C3.1 | 0.4526 |
| ENSP00000283109 | RIOK2 | 0.4526 |
| ENSP00000301459 | RCOR2 | 0.4526 |
| ENSP00000222145 | RASIP1 | 0.4526 |
| ENSP00000291041 | PSKH1 | 0.4526 |
| ENSP00000234179 | PRKD3 | 0.4526 |
| ENSP00000315035 | PPP1R42 | 0.4526 |
| ENSP00000301242 | PPP1R14A | 0.4526 |
| ENSP00000465213 | POP4 | 0.4526 |
| ENSP00000317128 | PLXND1 | 0.4526 |
| ENSP00000217971 | PGRMC1 | 0.4526 |
| ENSP00000303511 | PEX6 | 0.4526 |
| ENSP00000408598 | PABPN1L | 0.4526 |
| ENSP00000298440 | OXGR1 | 0.4526 |
| ENSP00000385636 | OBSL1 | 0.4526 |
| ENSP00000225388 | NUFIP2 | 0.4526 |
| ENSP00000368314 | NRCAM | 0.4526 |
| ENSP00000382274 | NOMO3 | 0.4526 |
| ENSP00000265500 | NDUFC1 | 0.4526 |
| ENSP00000247866 | NDUFB2 | 0.4526 |
| ENSP00000323076 | NDUFAF3 | 0.4526 |
| ENSP00000251127 | NALCN | 0.4526 |
| ENSP00000223167 | MYL10 | 0.4526 |
| ENSP00000304147 | MYEOV2 | 0.4526 |
| ENSP00000350332 | MYBPC2 | 0.4526 |
| ENSP00000228510 | MVK | 0.4526 |
| ENSP00000331849 | MRPL54 | 0.4526 |
| ENSP00000363999 | MRPL50 | 0.4526 |
| ENSP00000240488 | MND1 | 0.4526 |
| ENSP00000262065 | MMD | 0.4526 |
| ENSP00000383894 | MATN3 | 0.4526 |
| ENSP00000359240 | LZTS2 | 0.4526 |
| ENSP00000284818 | LY96 | 0.4526 |
| ENSP00000357020 | LY9 | 0.4526 |

| | | |
|---|---|---|
| ENSP00000469468 | LSM4 | 0.4526 |
| ENSP00000295628 | LRRC58 | 0.4526 |
| ENSP00000326759 | LRIG3 | 0.4526 |
| ENSP00000359643 | LPAR3 | 0.4526 |
| ENSP00000216237 | L3MBTL2 | 0.4526 |
| ENSP00000351933 | KLHL9 | 0.4526 |
| ENSP00000343273 | KLHL7 | 0.4526 |
| ENSP00000356990 | KLHDC9 | 0.4526 |
| ENSP00000358784 | KCNA3 | 0.4526 |
| ENSP00000262419 | KANSL1 | 0.4526 |
| ENSP00000269593 | IGFBP4 | 0.4526 |
| ENSP00000316244 | HTR1A | 0.4526 |
| ENSP00000199936 | HSD17B2 | 0.4526 |
| ENSP00000366506 | HRH2 | 0.4526 |
| ENSP00000380177 | HIST1H2BN | 0.4526 |
| ENSP00000352627 | HIST1H2AM | 0.4526 |
| ENSP00000352119 | HIST1H2AG | 0.4526 |
| ENSP00000259791 | HIST1H2AB | 0.4526 |
| ENSP00000297012 | HIST1H2AA | 0.4526 |
| ENSP00000359049 | HENMT1 | 0.4526 |
| ENSP00000293330 | HCRT | 0.4526 |
| ENSP00000261170 | GUCY2C | 0.4526 |
| ENSP00000378408 | GPT | 0.4526 |
| ENSP00000237858 | GLRX | 0.4526 |
| ENSP00000355675 | GJC2 | 0.4526 |
| ENSP00000333157 | GH2 | 0.4526 |
| ENSP00000458307 | GGT6 | 0.4526 |
| ENSP00000272224 | GDF7 | 0.4526 |
| ENSP00000224605 | GDF10 | 0.4526 |
| ENSP00000299267 | GABRB3 | 0.4526 |
| ENSP00000313691 | FTMT | 0.4526 |
| ENSP00000371798 | FSCN1 | 0.4526 |
| ENSP00000350032 | FRMD4A | 0.4526 |
| ENSP00000417257 | FNDC3A | 0.4526 |
| ENSP00000318884 | FMN2 | 0.4526 |
| ENSP00000326570 | FKRP | 0.4526 |
| ENSP00000310686 | FBXW8 | 0.4526 |
| ENSP00000385021 | FANCL | 0.4526 |
| ENSP00000346635 | ERO1LB | 0.4526 |
| ENSP00000348089 | ERCC6 | 0.4526 |
| ENSP00000199389 | EIF2AK1 | 0.4526 |
| ENSP00000357393 | EFNA3 | 0.4526 |
| ENSP00000228862 | DUSP16 | 0.4526 |
| ENSP00000225729 | DRG2 | 0.4526 |
| ENSP00000365838 | DPRX | 0.4526 |
| ENSP00000262960 | DPP9 | 0.4526 |
| ENSP00000296218 | DNALI1 | 0.4526 |
| ENSP00000305785 | DMRT2 | 0.4526 |
| ENSP00000356366 | DENND1B | 0.4526 |
| ENSP00000228476 | DAO | 0.4526 |
| ENSP00000378360 | DAK | 0.4526 |
| ENSP00000265968 | CSRP3 | 0.4526 |
| ENSP00000264193 | CPOX | 0.4526 |

| | | |
|---|---|---|
| ENSP00000296046 | CPA3 | 0.4526 |
| ENSP00000373298 | COLQ | 0.4526 |
| ENSP00000275162 | CLVS2 | 0.4526 |
| ENSP00000353732 | CLIP3 | 0.4526 |
| ENSP00000335632 | CHP1 | 0.4526 |
| ENSP00000344683 | CDK5RAP3 | 0.4526 |
| ENSP00000316121 | CDCA8 | 0.4526 |
| ENSP00000259631 | CCL27 | 0.4526 |
| ENSP00000379157 | CAST | 0.4526 |
| ENSP00000304364 | C1GALT1C1 | 0.4526 |
| ENSP00000396052 | BRMS1 | 0.4526 |
| ENSP00000359474 | BARHL2 | 0.4526 |
| ENSP00000286800 | BACH1 | 0.4526 |
| ENSP00000367697 | ATP6AP2 | 0.4526 |
| ENSP00000286371 | ATP1B3 | 0.4526 |
| ENSP00000250111 | ATP1B2 | 0.4526 |
| ENSP00000384259 | ATG4B | 0.4526 |
| ENSP00000370639 | ASMT | 0.4526 |
| ENSP00000397582 | AMY2A | 0.4526 |
| ENSP00000293761 | ALOX15 | 0.4526 |
| ENSP00000355377 | AKR7A3 | 0.4526 |
| ENSP00000263126 | AKR1C4 | 0.4526 |
| ENSP00000467958 | AC006538.4 | 0.4526 |
| ENSP00000311030 | ABCC12 | 0.4526 |
| ENSP00000375133 | ZRSR1 | 0.4500 |
| ENSP00000383020 | ZNF705G | 0.4500 |
| ENSP00000313258 | ZNF541 | 0.4500 |
| ENSP00000349494 | ZNF254 | 0.4500 |
| ENSP00000353863 | ZNF148 | 0.4500 |
| ENSP00000349708 | ZMYM6 | 0.4500 |
| ENSP00000339030 | ZFP91 | 0.4500 |
| ENSP00000262394 | WSB1 | 0.4500 |
| ENSP00000324203 | WRAP53 | 0.4500 |
| ENSP00000297954 | WNK2 | 0.4500 |
| ENSP00000385361 | VPREB1 | 0.4500 |
| ENSP00000309262 | USP48 | 0.4500 |
| ENSP00000304845 | UGT1A1 | 0.4500 |
| ENSP00000362711 | TXLNA | 0.4500 |
| ENSP00000451131 | TTC6 | 0.4500 |
| ENSP00000327984 | TSPY8 | 0.4500 |
| ENSP00000309402 | TRAM1L1 | 0.4500 |
| ENSP00000329093 | TPH2 | 0.4500 |
| ENSP00000347930 | TMEM30B | 0.4500 |
| ENSP00000344166 | TMEM189-UBE2V1 | 0.4500 |
| ENSP00000267415 | TINF2 | 0.4500 |
| ENSP00000363211 | TIMM23B | 0.4500 |
| ENSP00000295400 | TGFA | 0.4500 |
| ENSP00000223051 | TFR2 | 0.4500 |
| ENSP00000249284 | TAS2R16 | 0.4500 |
| ENSP00000371932 | TAS2R1 | 0.4500 |
| ENSP00000312709 | TAF7 | 0.4500 |
| ENSP00000399982 | TAF6 | 0.4500 |
| ENSP00000272902 | SUMF1 | 0.4500 |

| | | |
|---|---|---|
| ENSP00000366746 | STAM | 0.4500 |
| ENSP00000265437 | ST7 | 0.4500 |
| ENSP00000269033 | SSH2 | 0.4500 |
| ENSP00000265896 | SQLE | 0.4500 |
| ENSP00000365019 | SPIN1 | 0.4500 |
| ENSP00000297316 | SOX17 | 0.4500 |
| ENSP00000261368 | SNCAIP | 0.4500 |
| ENSP00000355110 | SMOC1 | 0.4500 |
| ENSP00000263073 | SMG6 | 0.4500 |
| ENSP00000298085 | SLC7A3 | 0.4500 |
| ENSP00000336888 | SLC44A2 | 0.4500 |
| ENSP00000363807 | SLC39A7 | 0.4500 |
| ENSP00000246421 | SLC35E2 | 0.4500 |
| ENSP00000243896 | SLC35C2 | 0.4500 |
| ENSP00000363394 | SLC30A2 | 0.4500 |
| ENSP00000378920 | SLC26A6 | 0.4500 |
| ENSP00000265715 | SLC26A4 | 0.4500 |
| ENSP00000225665 | SLC25A11 | 0.4500 |
| ENSP00000215882 | SLC25A1 | 0.4500 |
| ENSP00000344322 | SLC23A2 | 0.4500 |
| ENSP00000278379 | SLC1A2 | 0.4500 |
| ENSP00000358640 | SLC16A1 | 0.4500 |
| ENSP00000365686 | SLC15A1 | 0.4500 |
| ENSP00000357022 | SLAMF7 | 0.4500 |
| ENSP00000319417 | SKA3 | 0.4500 |
| ENSP00000262901 | SIM1 | 0.4500 |
| ENSP00000368477 | SERP2 | 0.4500 |
| ENSP00000327197 | SENP5 | 0.4500 |
| ENSP00000270176 | SCYL1 | 0.4500 |
| ENSP00000358016 | SCML4 | 0.4500 |
| ENSP00000326247 | SAMD9L | 0.4500 |
| ENSP00000350878 | S1PR3 | 0.4500 |
| ENSP00000325594 | RUFY1 | 0.4500 |
| ENSP00000222247 | RPL18A | 0.4500 |
| ENSP00000255499 | RNF128 | 0.4500 |
| ENSP00000371434 | RFX3 | 0.4500 |
| ENSP00000233596 | REEP6 | 0.4500 |
| ENSP00000348538 | RBM38 | 0.4500 |
| ENSP00000255688 | RARRES3 | 0.4500 |
| ENSP00000362639 | RABEPK | 0.4500 |
| ENSP00000272198 | PPFIA4 | 0.4500 |
| ENSP00000392553 | PPAPDC1B | 0.4500 |
| ENSP00000400036 | PLA2G4C | 0.4500 |
| ENSP00000335618 | PITPNC1 | 0.4500 |
| ENSP00000334738 | PITPNB | 0.4500 |
| ENSP00000369274 | PHKA2 | 0.4500 |
| ENSP00000362807 | PEF1 | 0.4500 |
| ENSP00000256404 | PEBP4 | 0.4500 |
| ENSP00000321804 | PAQR4 | 0.4500 |
| ENSP00000324270 | OXTR | 0.4500 |
| ENSP00000358967 | OPN1LW | 0.4500 |
| ENSP00000257627 | OCM2 | 0.4500 |
| ENSP00000381105 | NPS | 0.4500 |

| | | |
|---|---|---|
| ENSP00000259526 | NOV | 0.4500 |
| ENSP00000307853 | MUS81 | 0.4500 |
| ENSP00000441929 | MUC12 | 0.4500 |
| ENSP00000300057 | MESP1 | 0.4500 |
| ENSP00000325425 | MAT2B | 0.4500 |
| ENSP00000296280 | MASP1 | 0.4500 |
| ENSP00000255681 | MACROD1 | 0.4500 |
| ENSP00000296581 | LSM6 | 0.4500 |
| ENSP00000346414 | LRRIQ3 | 0.4500 |
| ENSP00000344233 | LRRC43 | 0.4500 |
| ENSP00000310126 | LINGO2 | 0.4500 |
| ENSP00000293760 | LEMD2 | 0.4500 |
| ENSP00000348298 | LCOR | 0.4500 |
| ENSP00000356162 | KISS1 | 0.4500 |
| ENSP00000377934 | KIF7 | 0.4500 |
| ENSP00000328494 | KIF21B | 0.4500 |
| ENSP00000256707 | KIDINS220 | 0.4500 |
| ENSP00000300035 | KIAA0101 | 0.4500 |
| ENSP00000357067 | KCNJ9 | 0.4500 |
| ENSP00000314520 | KCNA2 | 0.4500 |
| ENSP00000395742 | JAM3 | 0.4500 |
| ENSP00000457016 | INO80E | 0.4500 |
| ENSP00000308716 | INHBC | 0.4500 |
| ENSP00000295228 | INHBB | 0.4500 |
| ENSP00000229134 | IL26 | 0.4500 |
| ENSP00000233954 | IL1RL1 | 0.4500 |
| ENSP00000327824 | IFNLR1 | 0.4500 |
| ENSP00000382714 | IFITM2 | 0.4500 |
| ENSP00000337949 | HTR7 | 0.4500 |
| ENSP00000350549 | HMX3 | 0.4500 |
| ENSP00000417404 | HFE | 0.4500 |
| ENSP00000216465 | GSTZ1 | 0.4500 |
| ENSP00000265276 | GPAM | 0.4500 |
| ENSP00000362153 | GNL2 | 0.4500 |
| ENSP00000241502 | FYTTD1 | 0.4500 |
| ENSP00000259748 | FRS3 | 0.4500 |
| ENSP00000379369 | FOXB1 | 0.4500 |
| ENSP00000378368 | FLOT2 | 0.4500 |
| ENSP00000420213 | FLNB | 0.4500 |
| ENSP00000307298 | FEM1B | 0.4500 |
| ENSP00000285398 | ERCC3 | 0.4500 |
| ENSP00000228741 | ELK3 | 0.4500 |
| ENSP00000315011 | EDNRA | 0.4500 |
| ENSP00000345575 | DNAJB12 | 0.4500 |
| ENSP00000361467 | DLG5 | 0.4500 |
| ENSP00000284690 | DHX32 | 0.4500 |
| ENSP00000408994 | DET1 | 0.4500 |
| ENSP00000328524 | DENND5A | 0.4500 |
| ENSP00000357920 | DDO | 0.4500 |
| ENSP00000451235 | DCAF8 | 0.4500 |
| ENSP00000315351 | D2HGDH | 0.4500 |
| ENSP00000248901 | CYTH4 | 0.4500 |
| ENSP00000353820 | CYP2D6 | 0.4500 |

| | | |
|---|---|---|
| ENSP00000260630 | CYP1B1 | 0.4500 |
| ENSP00000366124 | CST3 | 0.4500 |
| ENSP00000342015 | COX19 | 0.4500 |
| ENSP00000261037 | COL8A1 | 0.4500 |
| ENSP00000263665 | CNTN3 | 0.4500 |
| ENSP00000367276 | CKAP2 | 0.4500 |
| ENSP00000319851 | CHDH | 0.4500 |
| ENSP00000356399 | CFH | 0.4500 |
| ENSP00000332139 | CFD | 0.4500 |
| ENSP00000402697 | CERS1 | 0.4500 |
| ENSP00000326432 | CCR8 | 0.4500 |
| ENSP00000441600 | CCR3 | 0.4500 |
| ENSP00000295926 | CCNL1 | 0.4500 |
| ENSP00000378118 | CCL8 | 0.4500 |
| ENSP00000311695 | CCDC85B | 0.4500 |
| ENSP00000239374 | CCDC170 | 0.4500 |
| ENSP00000357057 | CASQ1 | 0.4500 |
| ENSP00000380523 | CAPSL | 0.4500 |
| ENSP00000418081 | CACNA2D2 | 0.4500 |
| ENSP00000345824 | BZRAP1 | 0.4500 |
| ENSP00000446880 | BAZ2A | 0.4500 |
| ENSP00000417190 | ATPAF2 | 0.4500 |
| ENSP00000313600 | ATP10B | 0.4500 |
| ENSP00000284629 | ASZ1 | 0.4500 |
| ENSP00000388169 | ARPC4 | 0.4500 |
| ENSP00000420333 | ARMC8 | 0.4500 |
| ENSP00000326884 | ARFRP1 | 0.4500 |
| ENSP00000320247 | AQP6 | 0.4500 |
| ENSP00000360998 | ANKRD22 | 0.4500 |
| ENSP00000359100 | AMY1A | 0.4500 |
| ENSP00000298375 | AKR1E2 | 0.4500 |
| ENSP00000417935 | AKR1CL1 | 0.4500 |
| ENSP00000279146 | AIP | 0.4500 |
| ENSP00000349436 | ADAM15 | 0.4500 |
| ENSP00000205214 | AASDH | 0.4500 |
| ENSP00000209873 | AAAS | 0.4500 |
| ENSP00000316746 | PRF1 | 0.4500 |
| ENSP00000369774 | ZNF763 | 0.4474 |
| ENSP00000337386 | ZFP36L1 | 0.4474 |
| ENSP00000293883 | WDR24 | 0.4474 |
| ENSP00000333255 | VMA21 | 0.4474 |
| ENSP00000305647 | VAMP5 | 0.4474 |
| ENSP00000366006 | UBIAD1 | 0.4474 |
| ENSP00000260505 | TTLL7 | 0.4474 |
| ENSP00000262839 | TRPC5 | 0.4474 |
| ENSP00000323913 | TRIM55 | 0.4474 |
| ENSP00000316990 | TRAPPC5 | 0.4474 |
| ENSP00000469049 | TNFSF14 | 0.4474 |
| ENSP00000386443 | TGOLN2 | 0.4474 |
| ENSP00000300086 | TERF2IP | 0.4474 |
| ENSP00000440704 | SYTL1 | 0.4474 |
| ENSP00000341282 | SYCE1 | 0.4474 |
| ENSP00000276646 | SYBU | 0.4474 |

| | | |
|---|---|---|
| ENSP00000398789 | SNX13 | 0.4474 |
| ENSP00000256958 | SLCO1B1 | 0.4474 |
| ENSP00000004531 | SLC7A2 | 0.4474 |
| ENSP00000231706 | SLC7A14 | 0.4474 |
| ENSP00000269740 | SLC39A3 | 0.4474 |
| ENSP00000316596 | SLC38A9 | 0.4474 |
| ENSP00000311833 | SLC37A2 | 0.4474 |
| ENSP00000245680 | SLC35F5 | 0.4474 |
| ENSP00000367770 | SLC35B4 | 0.4474 |
| ENSP00000330141 | SLC16A5 | 0.4474 |
| ENSP00000370381 | SLC12A1 | 0.4474 |
| ENSP00000370299 | SHROOM2 | 0.4474 |
| ENSP00000268989 | SGSM2 | 0.4474 |
| ENSP00000376268 | SEC14L1 | 0.4474 |
| ENSP00000345599 | SCXA | 0.4474 |
| ENSP00000386306 | SCN9A | 0.4474 |
| ENSP00000322460 | SCN4B | 0.4474 |
| ENSP00000415178 | RP4-539M6.19 | 0.4474 |
| ENSP00000308219 | RGMB | 0.4474 |
| ENSP00000405038 | REXO1L11P | 0.4474 |
| ENSP00000300069 | RBPMS2 | 0.4474 |
| ENSP00000309166 | RBM4 | 0.4474 |
| ENSP00000298854 | RAPSN | 0.4474 |
| ENSP00000302647 | RALGAPA1 | 0.4474 |
| ENSP00000281171 | PTPRO | 0.4474 |
| ENSP00000263708 | PTPN4 | 0.4474 |
| ENSP00000332812 | PTGDR2 | 0.4474 |
| ENSP00000272645 | POLR2D | 0.4474 |
| ENSP00000402861 | PLCL1 | 0.4474 |
| ENSP00000371833 | PLA2G4F | 0.4474 |
| ENSP00000225609 | PIGL | 0.4474 |
| ENSP00000383168 | PCBP3 | 0.4474 |
| ENSP00000351618 | PARD3B | 0.4474 |
| ENSP00000217386 | OXT | 0.4474 |
| ENSP00000457258 | OGFOD1 | 0.4474 |
| ENSP00000348831 | NUDT10 | 0.4474 |
| ENSP00000261402 | NUAK1 | 0.4474 |
| ENSP00000287667 | NOMO1 | 0.4474 |
| ENSP00000283429 | NMRAL1 | 0.4474 |
| ENSP00000354159 | NLRC4 | 0.4474 |
| ENSP00000253719 | NAPSA | 0.4474 |
| ENSP00000384288 | MYCBP2 | 0.4474 |
| ENSP00000221086 | MTMR9 | 0.4474 |
| ENSP00000325285 | MTMR4 | 0.4474 |
| ENSP00000436767 | MTG1 | 0.4474 |
| ENSP00000347277 | MRPL52 | 0.4474 |
| ENSP00000258383 | MRPL44 | 0.4474 |
| ENSP00000261326 | MOCOS | 0.4474 |
| ENSP00000383820 | MIER1 | 0.4474 |
| ENSP00000262843 | MID2 | 0.4474 |
| ENSP00000296755 | MAP1B | 0.4474 |
| ENSP00000313021 | MAK | 0.4474 |
| ENSP00000264265 | LXN | 0.4474 |

| | | |
|---|---|---|
| ENSP00000356548 | LTV1 | 0.4474 |
| ENSP00000419542 | LRRC55 | 0.4474 |
| ENSP00000446121 | LIMS1 | 0.4474 |
| ENSP00000413479 | KRT3 | 0.4474 |
| ENSP00000285407 | KLF10 | 0.4474 |
| ENSP00000260753 | KIF20B | 0.4474 |
| ENSP00000397239 | KDM4E | 0.4474 |
| ENSP00000391498 | KCNK16 | 0.4474 |
| ENSP00000229447 | IYD | 0.4474 |
| ENSP00000411822 | ISY1-RAB43 | 0.4474 |
| ENSP00000330408 | IST1 | 0.4474 |
| ENSP00000344741 | INSIG1 | 0.4474 |
| ENSP00000269159 | IMPA2 | 0.4474 |
| ENSP00000258729 | IGF2BP3 | 0.4474 |
| ENSP00000296051 | HPS3 | 0.4474 |
| ENSP00000363614 | HMGCL | 0.4474 |
| ENSP00000364076 | HLA-DQA2 | 0.4474 |
| ENSP00000355657 | HIST3H3 | 0.4474 |
| ENSP00000366962 | HIST1H2BI | 0.4474 |
| ENSP00000341214 | HIST1H1T | 0.4474 |
| ENSP00000396452 | HDHD1 | 0.4474 |
| ENSP00000206474 | HAUS4 | 0.4474 |
| ENSP00000264108 | HAT1 | 0.4474 |
| ENSP00000415430 | GTSE1 | 0.4474 |
| ENSP00000363296 | GRM4 | 0.4474 |
| ENSP00000367343 | GBA2 | 0.4474 |
| ENSP00000381549 | FRMD4B | 0.4474 |
| ENSP00000365898 | FOXB2 | 0.4474 |
| ENSP00000356729 | FMO3 | 0.4474 |
| ENSP00000209929 | FMO2 | 0.4474 |
| ENSP00000279227 | FERMT3 | 0.4474 |
| ENSP00000423630 | FBXL7 | 0.4474 |
| ENSP00000417601 | FBXL2 | 0.4474 |
| ENSP00000354497 | FAN1 | 0.4474 |
| ENSP00000244869 | EREG | 0.4474 |
| ENSP00000400376 | ERAP2 | 0.4474 |
| ENSP00000379383 | ENAM | 0.4474 |
| ENSP00000417183 | DUSP7 | 0.4474 |
| ENSP00000355958 | DTL | 0.4474 |
| ENSP00000339208 | DPP8 | 0.4474 |
| ENSP00000452037 | DNAL1 | 0.4474 |
| ENSP00000316670 | DHRS9 | 0.4474 |
| ENSP00000391167 | DENND4A | 0.4474 |
| ENSP00000414906 | DCTN4 | 0.4474 |
| ENSP00000367715 | DCDC2 | 0.4474 |
| ENSP00000261891 | DAPK2 | 0.4474 |
| ENSP00000260682 | CYP2C9 | 0.4474 |
| ENSP00000438788 | CUL7 | 0.4474 |
| ENSP00000470383 | CTC-273B12.7 | 0.4474 |
| ENSP00000389026 | CRISP3 | 0.4474 |
| ENSP00000433459 | CREBZF | 0.4474 |
| ENSP00000410396 | CMSS1 | 0.4474 |
| ENSP00000342991 | CLYBL | 0.4474 |

| | | |
|---|---|---|
| ENSP00000372193 | CLCN7 | 0.4474 |
| ENSP00000269878 | CIB3 | 0.4474 |
| ENSP00000384400 | CHKB | 0.4474 |
| ENSP00000262450 | CHD5 | 0.4474 |
| ENSP00000349696 | CEPT1 | 0.4474 |
| ENSP00000369075 | CENPB | 0.4474 |
| ENSP00000199764 | CEACAM6 | 0.4474 |
| ENSP00000358501 | CD58 | 0.4474 |
| ENSP00000316333 | CD55 | 0.4474 |
| ENSP00000317310 | CABP1 | 0.4474 |
| ENSP00000216115 | BIK | 0.4474 |
| ENSP00000369647 | AVP | 0.4474 |
| ENSP00000397259 | ATXN7L3 | 0.4474 |
| ENSP00000338769 | ASAP3 | 0.4474 |
| ENSP00000268042 | ARRDC4 | 0.4474 |
| ENSP00000249601 | ARHGAP22 | 0.4474 |
| ENSP00000336762 | ANG | 0.4474 |
| ENSP00000403270 | AGAP9 | 0.4474 |
| ENSP00000308549 | ADORA1 | 0.4474 |
| ENSP00000364555 | ACTL8 | 0.4474 |
| ENSP00000303246 | ACOT12 | 0.4474 |
| ENSP00000285238 | ABCC3 | 0.4474 |
| ENSP00000264159 | ZRANB3 | 0.4447 |
| ENSP00000257940 | ZC3H10 | 0.4447 |
| ENSP00000308179 | WDR3 | 0.4447 |
| ENSP00000295888 | WDFY3 | 0.4447 |
| ENSP00000397879 | VPS8 | 0.4447 |
| ENSP00000303129 | VAT1L | 0.4447 |
| ENSP00000369681 | USP3 | 0.4447 |
| ENSP00000351596 | TTC37 | 0.4447 |
| ENSP00000333018 | TTC32 | 0.4447 |
| ENSP00000303452 | TRH | 0.4447 |
| ENSP00000217121 | TPD52L2 | 0.4447 |
| ENSP00000389998 | TMEM67 | 0.4447 |
| ENSP00000411099 | TLK1 | 0.4447 |
| ENSP00000262968 | TJP3 | 0.4447 |
| ENSP00000291526 | TFF2 | 0.4447 |
| ENSP00000008391 | TFAP2D | 0.4447 |
| ENSP00000279386 | TBX6 | 0.4447 |
| ENSP00000321703 | TACC1 | 0.4447 |
| ENSP00000346265 | SYT5 | 0.4447 |
| ENSP00000255613 | SUV420H2 | 0.4447 |
| ENSP00000360515 | SUPT3H | 0.4447 |
| ENSP00000350734 | ST7L | 0.4447 |
| ENSP00000312318 | SSSCA1 | 0.4447 |
| ENSP00000312081 | SSH3 | 0.4447 |
| ENSP00000315713 | SSH1 | 0.4447 |
| ENSP00000261854 | SPPL2A | 0.4447 |
| ENSP00000265295 | SPDL1 | 0.4447 |
| ENSP00000295269 | SLC9A4 | 0.4447 |
| ENSP00000233969 | SLC9A2 | 0.4447 |
| ENSP00000261622 | SLC7A5 | 0.4447 |
| ENSP00000371483 | SLC34A2 | 0.4447 |

| | | |
|---|---|---|
| ENSP00000321424 | SLC34A1 | 0.4447 |
| ENSP00000233535 | SLC30A3 | 0.4447 |
| ENSP00000279178 | SLC22A9 | 0.4447 |
| ENSP00000444408 | SLC1A5 | 0.4447 |
| ENSP00000234256 | SLC1A4 | 0.4447 |
| ENSP00000227880 | SLC15A3 | 0.4447 |
| ENSP00000417085 | SLC15A2 | 0.4447 |
| ENSP00000316905 | SFXN1 | 0.4447 |
| ENSP00000370115 | SERPINB1 | 0.4447 |
| ENSP00000361867 | SEMG1 | 0.4447 |
| ENSP00000418910 | SELT | 0.4447 |
| ENSP00000360593 | SELRC1 | 0.4447 |
| ENSP00000281142 | SCLT1 | 0.4447 |
| ENSP00000350333 | SAP130 | 0.4447 |
| ENSP00000339521 | RSU1 | 0.4447 |
| ENSP00000254605 | RRP8 | 0.4447 |
| ENSP00000322419 | RPLP2 | 0.4447 |
| ENSP00000360120 | ROR1 | 0.4447 |
| ENSP00000444777 | REXO1L10P | 0.4447 |
| ENSP00000258428 | REV1 | 0.4447 |
| ENSP00000413929 | RCOR3 | 0.4447 |
| ENSP00000265271 | RBM27 | 0.4447 |
| ENSP00000368341 | RBM24 | 0.4447 |
| ENSP00000199814 | RBM22 | 0.4447 |
| ENSP00000376440 | RAD9B | 0.4447 |
| ENSP00000340879 | RAD1 | 0.4447 |
| ENSP00000386212 | PSMB11 | 0.4447 |
| ENSP00000358224 | PPIAL4C | 0.4447 |
| ENSP00000443989 | PPIAL4B | 0.4447 |
| ENSP00000215591 | POLRMT | 0.4447 |
| ENSP00000216832 | PNN | 0.4447 |
| ENSP00000358242 | PNISR | 0.4447 |
| ENSP00000276914 | PLIN2 | 0.4447 |
| ENSP00000367391 | PGA4 | 0.4447 |
| ENSP00000322192 | PGA3 | 0.4447 |
| ENSP00000356563 | PEX3 | 0.4447 |
| ENSP00000349016 | PEX14 | 0.4447 |
| ENSP00000280154 | PDCD4 | 0.4447 |
| ENSP00000353739 | PCMTD1 | 0.4447 |
| ENSP00000299299 | PCBD1 | 0.4447 |
| ENSP00000358747 | OVGP1 | 0.4447 |
| ENSP00000272371 | OTOF | 0.4447 |
| ENSP00000261183 | OSBPL8 | 0.4447 |
| ENSP00000354652 | NPY1R | 0.4447 |
| ENSP00000342848 | NOXA1 | 0.4447 |
| ENSP00000242994 | NEUROD4 | 0.4447 |
| ENSP00000349654 | NELL1 | 0.4447 |
| ENSP00000253814 | NDFIP1 | 0.4447 |
| ENSP00000352272 | MYOZ1 | 0.4447 |
| ENSP00000396774 | MUC20 | 0.4447 |
| ENSP00000303222 | MTCH2 | 0.4447 |
| ENSP00000355084 | MSRB1 | 0.4447 |
| ENSP00000318158 | MRPS24 | 0.4447 |

| | | |
|---|---|---|
| ENSP00000320184 | MRPS23 | 0.4447 |
| ENSP00000313437 | MMP19 | 0.4447 |
| ENSP00000215754 | MIF | 0.4447 |
| ENSP00000259050 | MARCH7 | 0.4447 |
| ENSP00000320043 | LYPLA1 | 0.4447 |
| ENSP00000296144 | LRRC2 | 0.4447 |
| ENSP00000262134 | LPCAT2 | 0.4447 |
| ENSP00000175091 | LAPTM4A | 0.4447 |
| ENSP00000272797 | KLHL23 | 0.4447 |
| ENSP00000313995 | KLHDC3 | 0.4447 |
| ENSP00000354560 | KIFAP3 | 0.4447 |
| ENSP00000385215 | KIAA1009 | 0.4447 |
| ENSP00000384391 | KCTD17 | 0.4447 |
| ENSP00000365198 | KAZN | 0.4447 |
| ENSP00000371734 | KANK1 | 0.4447 |
| ENSP00000358310 | ITGA10 | 0.4447 |
| ENSP00000355961 | INTS7 | 0.4447 |
| ENSP00000358865 | INA | 0.4447 |
| ENSP00000328133 | IL20RB | 0.4447 |
| ENSP00000260049 | IL18BP | 0.4447 |
| ENSP00000233809 | IGFBP2 | 0.4447 |
| ENSP00000280097 | HNMT | 0.4447 |
| ENSP00000363976 | HLA-DMA | 0.4447 |
| ENSP00000332790 | HIST2H2AB | 0.4447 |
| ENSP00000321744 | HIST1H2BC | 0.4447 |
| ENSP00000339566 | HIST1H1C | 0.4447 |
| ENSP00000294973 | HAAO | 0.4447 |
| ENSP00000445162 | GTF2H3 | 0.4447 |
| ENSP00000253237 | GRWD1 | 0.4447 |
| ENSP00000256857 | GRP | 0.4447 |
| ENSP00000293190 | GRIN2C | 0.4447 |
| ENSP00000294409 | GMEB1 | 0.4447 |
| ENSP00000371398 | GLIS3 | 0.4447 |
| ENSP00000326227 | GANC | 0.4447 |
| ENSP00000355632 | GALNT2 | 0.4447 |
| ENSP00000360488 | FRA10AC1 | 0.4447 |
| ENSP00000318716 | FAM9B | 0.4447 |
| ENSP00000362409 | FAM129B | 0.4447 |
| ENSP00000328103 | EIF4ENIF1 | 0.4447 |
| ENSP00000355480 | EFCAB2 | 0.4447 |
| ENSP00000270517 | ECSIT | 0.4447 |
| ENSP00000367841 | DYNLT3 | 0.4447 |
| ENSP00000412178 | DGKZ | 0.4447 |
| ENSP00000412292 | DEPDC1 | 0.4447 |
| ENSP00000448051 | DAZAP2 | 0.4447 |
| ENSP00000310721 | CYP7B1 | 0.4447 |
| ENSP00000360968 | CYP4X1 | 0.4447 |
| ENSP00000337915 | CYP3A4 | 0.4447 |
| ENSP00000360317 | CYP2C8 | 0.4447 |
| ENSP00000357204 | CRABP2 | 0.4447 |
| ENSP00000366787 | CNTNAP3B | 0.4447 |
| ENSP00000388872 | CLUH | 0.4447 |
| ENSP00000433919 | CLNS1A | 0.4447 |

| | | |
|---|---|---|
| ENSP00000353073 | CLN3 | 0.4447 |
| ENSP00000405577 | CLMP | 0.4447 |
| ENSP00000247153 | CFP | 0.4447 |
| ENSP00000355089 | CELF4 | 0.4447 |
| ENSP00000253693 | CAPN7 | 0.4447 |
| ENSP00000438949 | CACNB4 | 0.4447 |
| ENSP00000385019 | CACNA1I | 0.4447 |
| ENSP00000334198 | CACNA1H | 0.4447 |
| ENSP00000293255 | CABP5 | 0.4447 |
| ENSP00000256925 | CABLES1 | 0.4447 |
| ENSP00000310696 | B4GALT2 | 0.4447 |
| ENSP00000272238 | ATP6V1C2 | 0.4447 |
| ENSP00000364349 | ATF6B | 0.4447 |
| ENSP00000222250 | ARRDC2 | 0.4447 |
| ENSP00000363832 | AOX1 | 0.4447 |
| ENSP00000370254 | AKR1C1 | 0.4447 |
| ENSP00000379514 | ADCYAP1R1 | 0.4447 |
| ENSP00000382349 | ACSM4 | 0.4447 |
| ENSP00000238651 | ACOT2 | 0.4447 |
| ENSP00000411096 | ABCA13 | 0.4447 |
| ENSP00000429734 | 61E3.4 | 0.4447 |
| ENSP00000299927 | ZNF592 | 0.4421 |
| ENSP00000340796 | ZNF326 | 0.4421 |
| ENSP00000278856 | WDR74 | 0.4421 |
| ENSP00000261167 | WBP11 | 0.4421 |
| ENSP00000376504 | VWA5A | 0.4421 |
| ENSP00000301962 | USP42 | 0.4421 |
| ENSP00000294119 | UBXN1 | 0.4421 |
| ENSP00000217173 | UBOX5 | 0.4421 |
| ENSP00000305654 | TVP23B | 0.4421 |
| ENSP00000370031 | TTC8 | 0.4421 |
| ENSP00000295314 | TMOD4 | 0.4421 |
| ENSP00000288025 | TMED6 | 0.4421 |
| ENSP00000370867 | TGM3 | 0.4421 |
| ENSP00000207157 | TBX15 | 0.4421 |
| ENSP00000294168 | TAF6L | 0.4421 |
| ENSP00000258052 | SMPD2 | 0.4421 |
| ENSP00000296422 | SLC9B1 | 0.4421 |
| ENSP00000379326 | SLC5A12 | 0.4421 |
| ENSP00000363891 | SLC38A10 | 0.4421 |
| ENSP00000345528 | SLC35F6 | 0.4421 |
| ENSP00000339626 | SLC35E4 | 0.4421 |
| ENSP00000350278 | SLC30A7 | 0.4421 |
| ENSP00000263093 | SLC27A5 | 0.4421 |
| ENSP00000286298 | SLC26A2 | 0.4421 |
| ENSP00000400101 | SLC25A13 | 0.4421 |
| ENSP00000302851 | SLC23A1 | 0.4421 |
| ENSP00000290399 | SIM2 | 0.4421 |
| ENSP00000223095 | SERPINE1 | 0.4421 |
| ENSP00000338358 | SERPINB6 | 0.4421 |
| ENSP00000292169 | S100A1 | 0.4421 |
| ENSP00000346080 | RPL22L1 | 0.4421 |
| ENSP00000341905 | RASAL3 | 0.4421 |

| | | |
|---|---|---|
| ENSP00000359607 | PYROXD2 | 0.4421 |
| ENSP00000332706 | PURA | 0.4421 |
| ENSP00000334941 | PTPRU | 0.4421 |
| ENSP00000363459 | PTPN20A | 0.4421 |
| ENSP00000360687 | PTGDS | 0.4421 |
| ENSP00000301175 | PRODH2 | 0.4421 |
| ENSP00000408342 | PRAM1 | 0.4421 |
| ENSP00000354218 | PPP1R13L | 0.4421 |
| ENSP00000360296 | PPAP2B | 0.4421 |
| ENSP00000373702 | PP2D1 | 0.4421 |
| ENSP00000349205 | PIK3R4 | 0.4421 |
| ENSP00000339382 | PIGO | 0.4421 |
| ENSP00000410626 | PHRF1 | 0.4421 |
| ENSP00000349543 | PEX2 | 0.4421 |
| ENSP00000402551 | OTUB1 | 0.4421 |
| ENSP00000380193 | NRL | 0.4421 |
| ENSP00000345752 | MTMR2 | 0.4421 |
| ENSP00000355304 | MOK | 0.4421 |
| ENSP00000301327 | MFSD3 | 0.4421 |
| ENSP00000339047 | LRRC61 | 0.4421 |
| ENSP00000302621 | LRG1 | 0.4421 |
| ENSP00000262301 | LMF1 | 0.4421 |
| ENSP00000278193 | LIN7C | 0.4421 |
| ENSP00000428765 | LETM2 | 0.4421 |
| ENSP00000369317 | KRT6A | 0.4421 |
| ENSP00000229402 | KLRB1 | 0.4421 |
| ENSP00000343930 | KLHL17 | 0.4421 |
| ENSP00000344820 | KCNK7 | 0.4421 |
| ENSP00000295082 | KCNF1 | 0.4421 |
| ENSP00000327290 | ITGA11 | 0.4421 |
| ENSP00000270800 | IL22RA1 | 0.4421 |
| ENSP00000302935 | IL16 | 0.4421 |
| ENSP00000343552 | ICMT | 0.4421 |
| ENSP00000314736 | HYDIN | 0.4421 |
| ENSP00000372224 | HGFAC | 0.4421 |
| ENSP00000304185 | GTSF1 | 0.4421 |
| ENSP00000426120 | GRAMD3 | 0.4421 |
| ENSP00000371420 | GPNMB | 0.4421 |
| ENSP00000241125 | GJA3 | 0.4421 |
| ENSP00000350005 | GIP | 0.4421 |
| ENSP00000383558 | GCGR | 0.4421 |
| ENSP00000344012 | GAS2L1 | 0.4421 |
| ENSP00000364150 | GALNT12 | 0.4421 |
| ENSP00000261304 | GALC | 0.4421 |
| ENSP00000236166 | FMO6P | 0.4421 |
| ENSP00000386722 | FCHSD2 | 0.4421 |
| ENSP00000367910 | FANCG | 0.4421 |
| ENSP00000272610 | FAHD2B | 0.4421 |
| ENSP00000296677 | F2RL1 | 0.4421 |
| ENSP00000384957 | F11 | 0.4421 |
| ENSP00000265038 | ERCC8 | 0.4421 |
| ENSP00000416892 | EIF3L | 0.4421 |
| ENSP00000320130 | DYNC1I1 | 0.4421 |

| | | |
|---|---|---|
| ENSP00000357087 | DUSP23 | 0.4421 |
| ENSP00000257198 | DSC1 | 0.4421 |
| ENSP00000315988 | DPY19L2 | 0.4421 |
| ENSP00000294618 | DOCK6 | 0.4421 |
| ENSP00000251473 | DKFZP761J1410 | 0.4421 |
| ENSP00000252137 | DGCR14 | 0.4421 |
| ENSP00000297439 | DEFB1 | 0.4421 |
| ENSP00000415556 | DCAF11 | 0.4421 |
| ENSP00000274867 | DAAM2 | 0.4421 |
| ENSP00000252945 | CYP2E1 | 0.4421 |
| ENSP00000343900 | CPSF4L | 0.4421 |
| ENSP00000234301 | COX7A2L | 0.4421 |
| ENSP00000354762 | COA3 | 0.4421 |
| ENSP00000359285 | CHRNA4 | 0.4421 |
| ENSP00000372750 | CHRM5 | 0.4421 |
| ENSP00000361923 | CHCHD1 | 0.4421 |
| ENSP00000360918 | CH25H | 0.4421 |
| ENSP00000364389 | CDC14B | 0.4421 |
| ENSP00000335657 | CCK | 0.4421 |
| ENSP00000260508 | CCBL2 | 0.4421 |
| ENSP00000398481 | CADPS2 | 0.4421 |
| ENSP00000373215 | CADPS | 0.4421 |
| ENSP00000419101 | CACNA2D3 | 0.4421 |
| ENSP00000289388 | C2orf62 | 0.4421 |
| ENSP00000255192 | BHMT2 | 0.4421 |
| ENSP00000243578 | B9D2 | 0.4421 |
| ENSP00000257861 | AVIL | 0.4421 |
| ENSP00000371070 | ATP8A2 | 0.4421 |
| ENSP00000352522 | ATP6V1H | 0.4421 |
| ENSP00000451575 | ASB2 | 0.4421 |
| ENSP00000259477 | ARPC5L | 0.4421 |
| ENSP00000270747 | ARHGEF19 | 0.4421 |
| ENSP00000221891 | APLP1 | 0.4421 |
| ENSP00000400325 | AP000350.10 | 0.4421 |
| ENSP00000297837 | ANKS6 | 0.4421 |
| ENSP00000369530 | ALOX15B | 0.4421 |
| ENSP00000346577 | AK5 | 0.4421 |
| ENSP00000369960 | ADRA1A | 0.4421 |
| ENSP00000381036 | ADGB | 0.4421 |
| ENSP00000357668 | ADAM12 | 0.4421 |
| ENSP00000360165 | ACTRT1 | 0.4421 |
| ENSP00000363799 | ACTL7B | 0.4421 |
| ENSP00000454833 | AC025263.3 | 0.4421 |
| ENSP00000352676 | ZRANB1 | 0.4395 |
| ENSP00000262096 | ZDHHC2 | 0.4395 |
| ENSP00000327821 | XPOT | 0.4395 |
| ENSP00000287380 | WDR67 | 0.4395 |
| ENSP00000382717 | WDR19 | 0.4395 |
| ENSP00000347872 | VAT1 | 0.4395 |
| ENSP00000229268 | USP5 | 0.4395 |
| ENSP00000320401 | UGT2B17 | 0.4395 |
| ENSP00000301281 | UBXN6 | 0.4395 |
| ENSP00000268483 | TXNL4B | 0.4395 |

| | | |
|---|---|---|
| ENSP00000304908 | TXNDC2 | 0.4395 |
| ENSP00000380897 | TRPM1 | 0.4395 |
| ENSP00000426070 | TRPC7 | 0.4395 |
| ENSP00000273962 | TRMT10A | 0.4395 |
| ENSP00000424048 | TRIM39-RPP21 | 0.4395 |
| ENSP00000272424 | TPRKB | 0.4395 |
| ENSP00000233143 | TMSB10 | 0.4395 |
| ENSP00000422473 | TMEM33 | 0.4395 |
| ENSP00000375975 | TMCO1 | 0.4395 |
| ENSP00000287814 | TIMP4 | 0.4395 |
| ENSP00000220616 | TG | 0.4395 |
| ENSP00000343995 | TEKT3 | 0.4395 |
| ENSP00000369736 | TBCA | 0.4395 |
| ENSP00000440207 | TBC1D30 | 0.4395 |
| ENSP00000225235 | TBC1D12 | 0.4395 |
| ENSP00000355051 | TAF13 | 0.4395 |
| ENSP00000358531 | SYCP1 | 0.4395 |
| ENSP00000361042 | SURF1 | 0.4395 |
| ENSP00000353007 | SULF2 | 0.4395 |
| ENSP00000216218 | ST13 | 0.4395 |
| ENSP00000282074 | SPC25 | 0.4395 |
| ENSP00000320378 | SLC7A8 | 0.4395 |
| ENSP00000261024 | SLC40A1 | 0.4395 |
| ENSP00000300456 | SLC27A4 | 0.4395 |
| ENSP00000360855 | SLC16A12 | 0.4395 |
| ENSP00000261994 | SERPINA10 | 0.4395 |
| ENSP00000460441 | SEPT1 | 0.4395 |
| ENSP00000350215 | SEMA5B | 0.4395 |
| ENSP00000358071 | SEC23IP | 0.4395 |
| ENSP00000215812 | SEC14L3 | 0.4395 |
| ENSP00000367197 | SCGN | 0.4395 |
| ENSP00000296370 | S100P | 0.4395 |
| ENSP00000321449 | RRP7A | 0.4395 |
| ENSP00000420821 | RPL41 | 0.4395 |
| ENSP00000367766 | RPGR | 0.4395 |
| ENSP00000346402 | RNH1 | 0.4395 |
| ENSP00000385328 | RNF43 | 0.4395 |
| ENSP00000386229 | RGS14 | 0.4395 |
| ENSP00000309163 | RCE1 | 0.4395 |
| ENSP00000318415 | RBMXL1 | 0.4395 |
| ENSP00000264808 | PRDM5 | 0.4395 |
| ENSP00000383402 | PPP4R1 | 0.4395 |
| ENSP00000382058 | POLR3G | 0.4395 |
| ENSP00000345988 | PLCH1 | 0.4395 |
| ENSP00000393847 | PLA2G10 | 0.4395 |
| ENSP00000354775 | PJA2 | 0.4395 |
| ENSP00000379496 | PDE1C | 0.4395 |
| ENSP00000353868 | PCED1A | 0.4395 |
| ENSP00000412733 | OSBPL9 | 0.4395 |
| ENSP00000304854 | NUDCD2 | 0.4395 |
| ENSP00000313169 | NPHP1 | 0.4395 |
| ENSP00000275857 | NLGN4X | 0.4395 |
| ENSP00000216286 | NID2 | 0.4395 |

| | | |
|---|---|---|
| ENSP00000299821 | NCAPH2 | 0.4395 |
| ENSP00000334563 | MYO18B | 0.4395 |
| ENSP00000180173 | MTMR7 | 0.4395 |
| ENSP00000342343 | MED22 | 0.4395 |
| ENSP00000302537 | MDM1 | 0.4395 |
| ENSP00000324944 | MBOAT1 | 0.4395 |
| ENSP00000351813 | MARCH5 | 0.4395 |
| ENSP00000330572 | MAPK8IP2 | 0.4395 |
| ENSP00000443900 | LYRM4 | 0.4395 |
| ENSP00000373846 | LTN1 | 0.4395 |
| ENSP00000337224 | LRAT | 0.4395 |
| ENSP00000451812 | LIN52 | 0.4395 |
| ENSP00000272134 | LEFTY1 | 0.4395 |
| ENSP00000217407 | LBP | 0.4395 |
| ENSP00000336721 | LARP1 | 0.4395 |
| ENSP00000380982 | KIAA0020 | 0.4395 |
| ENSP00000306497 | KCNJ4 | 0.4395 |
| ENSP00000328150 | KCNJ12 | 0.4395 |
| ENSP00000331727 | KCNH7 | 0.4395 |
| ENSP00000356087 | IKBKE | 0.4395 |
| ENSP00000357113 | IFI16 | 0.4395 |
| ENSP00000370748 | IDI1 | 0.4395 |
| ENSP00000367959 | HTR2A | 0.4395 |
| ENSP00000328269 | HMG20B | 0.4395 |
| ENSP00000303408 | HIST1H2BO | 0.4395 |
| ENSP00000303373 | HIST1H2AE | 0.4395 |
| ENSP00000283871 | HGD | 0.4395 |
| ENSP00000253669 | HAUS8 | 0.4395 |
| ENSP00000263956 | GTF3C3 | 0.4395 |
| ENSP00000433511 | GRK6 | 0.4395 |
| ENSP00000264126 | GPSM2 | 0.4395 |
| ENSP00000468772 | GPS2 | 0.4395 |
| ENSP00000356581 | GPR126 | 0.4395 |
| ENSP00000300406 | GNGT2 | 0.4395 |
| ENSP00000359675 | GNG5 | 0.4395 |
| ENSP00000370194 | GMDS | 0.4395 |
| ENSP00000308533 | GEMIN2 | 0.4395 |
| ENSP00000255759 | FOPNL | 0.4395 |
| ENSP00000303423 | FNTA | 0.4395 |
| ENSP00000359506 | FMR1 | 0.4395 |
| ENSP00000278840 | FADS2 | 0.4395 |
| ENSP00000220562 | EXTL3 | 0.4395 |
| ENSP00000296754 | ERAP1 | 0.4395 |
| ENSP00000332656 | ENPP7 | 0.4395 |
| ENSP00000337128 | EDN3 | 0.4395 |
| ENSP00000314810 | EDDM3B | 0.4395 |
| ENSP00000307850 | DRAP1 | 0.4395 |
| ENSP00000389630 | DPY19L4 | 0.4395 |
| ENSP00000254579 | DNHD1 | 0.4395 |
| ENSP00000288111 | DHRS1 | 0.4395 |
| ENSP00000367454 | DFFB | 0.4395 |
| ENSP00000373734 | DENND5B | 0.4395 |
| ENSP00000359446 | CPN1 | 0.4395 |

| | | |
|---|---|---|
| ENSP00000359225 | CNN3 | 0.4395 |
| ENSP00000419395 | CDNF | 0.4395 |
| ENSP00000370588 | CD99 | 0.4395 |
| ENSP00000298546 | C9orf9 | 0.4395 |
| ENSP00000216038 | C22orf28 | 0.4395 |
| ENSP00000255608 | BTBD2 | 0.4395 |
| ENSP00000350914 | BDH1 | 0.4395 |
| ENSP00000233997 | AZU1 | 0.4395 |
| ENSP00000342481 | ATP9A | 0.4395 |
| ENSP00000311318 | ATG4D | 0.4395 |
| ENSP00000421315 | AQP1 | 0.4395 |
| ENSP00000359224 | ALG14 | 0.4395 |
| ENSP00000310120 | ALG10B | 0.4395 |
| ENSP00000367658 | ACTRT2 | 0.4395 |
| ENSP00000326491 | ABHD1 | 0.4395 |
| ENSP00000349017 | ABCC11 | 0.4395 |
| ENSP00000253577 | ABCB7 | 0.4395 |
| ENSP00000435509 | ZIC4 | 0.4368 |
| ENSP00000294258 | ZFPL1 | 0.4368 |
| ENSP00000324463 | YLPM1 | 0.4368 |
| ENSP00000428619 | UTP14C | 0.4368 |
| ENSP00000297229 | USP49 | 0.4368 |
| ENSP00000263897 | USE1 | 0.4368 |
| ENSP00000312107 | UBXN2A | 0.4368 |
| ENSP00000313953 | TTC27 | 0.4368 |
| ENSP00000329858 | TRMT12 | 0.4368 |
| ENSP00000169551 | TIMM21 | 0.4368 |
| ENSP00000258449 | TGFBRAP1 | 0.4368 |
| ENSP00000366036 | TBC1D8 | 0.4368 |
| ENSP00000390761 | TBC1D3C | 0.4368 |
| ENSP00000395220 | SYTL5 | 0.4368 |
| ENSP00000266743 | SYCP3 | 0.4368 |
| ENSP00000423541 | SV2C | 0.4368 |
| ENSP00000317331 | SSR4 | 0.4368 |
| ENSP00000244763 | SSR1 | 0.4368 |
| ENSP00000274192 | SRD5A1 | 0.4368 |
| ENSP00000301691 | SOST | 0.4368 |
| ENSP00000261196 | SLCO1B3 | 0.4368 |
| ENSP00000331938 | SLC9B1P1 | 0.4368 |
| ENSP00000305302 | SLC6A19 | 0.4368 |
| ENSP00000359892 | SLC44A5 | 0.4368 |
| ENSP00000353557 | SLC35F1 | 0.4368 |
| ENSP00000271628 | SF3B4 | 0.4368 |
| ENSP00000303212 | SEMA3E | 0.4368 |
| ENSP00000382327 | SDR39U1 | 0.4368 |
| ENSP00000244496 | RRP36 | 0.4368 |
| ENSP00000320898 | RNF168 | 0.4368 |
| ENSP00000430955 | RNF145 | 0.4368 |
| ENSP00000358099 | RGS10 | 0.4368 |
| ENSP00000332208 | RFX6 | 0.4368 |
| ENSP00000267229 | RBM26 | 0.4368 |
| ENSP00000291576 | PWP2 | 0.4368 |
| ENSP00000343081 | PUS7L | 0.4368 |

| | | |
|---|---|---|
| ENSP00000418721 | PURG | 0.4368 |
| ENSP00000252329 | PSMG3 | 0.4368 |
| ENSP00000284601 | PPP1R3A | 0.4368 |
| ENSP00000340510 | PPL | 0.4368 |
| ENSP00000380129 | PPIP5K1 | 0.4368 |
| ENSP00000257694 | PNPLA8 | 0.4368 |
| ENSP00000367747 | PLCH2 | 0.4368 |
| ENSP00000346809 | PGAP1 | 0.4368 |
| ENSP00000288774 | PEX10 | 0.4368 |
| ENSP00000351789 | PELI1 | 0.4368 |
| ENSP00000337405 | PCID2 | 0.4368 |
| ENSP00000261584 | PALB2 | 0.4368 |
| ENSP00000364620 | PADI1 | 0.4368 |
| ENSP00000166534 | P4HA2 | 0.4368 |
| ENSP00000326869 | ORMDL1 | 0.4368 |
| ENSP00000279147 | ORAOV1 | 0.4368 |
| ENSP00000256010 | NTS | 0.4368 |
| ENSP00000338349 | NRXN3 | 0.4368 |
| ENSP00000357581 | NKX6-2 | 0.4368 |
| ENSP00000372335 | NELFA | 0.4368 |
| ENSP00000383295 | NBEA | 0.4368 |
| ENSP00000261745 | NAA25 | 0.4368 |
| ENSP00000258874 | MTHFS | 0.4368 |
| ENSP00000361014 | MKNK1 | 0.4368 |
| ENSP00000357643 | MKI67 | 0.4368 |
| ENSP00000290130 | MIS18A | 0.4368 |
| ENSP00000403117 | MFSD1 | 0.4368 |
| ENSP00000333821 | MC2R | 0.4368 |
| ENSP00000354671 | MAST2 | 0.4368 |
| ENSP00000355877 | MARC1 | 0.4368 |
| ENSP00000306524 | LRRC8E | 0.4368 |
| ENSP00000332681 | LRP2BP | 0.4368 |
| ENSP00000369445 | KRTAP10-7 | 0.4368 |
| ENSP00000297625 | KIAA1161 | 0.4368 |
| ENSP00000408405 | KCTD1 | 0.4368 |
| ENSP00000243457 | KCNJ2 | 0.4368 |
| ENSP00000355568 | IRF2BP2 | 0.4368 |
| ENSP00000354519 | INPP5F | 0.4368 |
| ENSP00000262992 | INPP4B | 0.4368 |
| ENSP00000369564 | IFNA16 | 0.4368 |
| ENSP00000375736 | HIST3H2BB | 0.4368 |
| ENSP00000358158 | HIST2H2AA3 | 0.4368 |
| ENSP00000348924 | HIST1H2BE | 0.4368 |
| ENSP00000367714 | HES5 | 0.4368 |
| ENSP00000233099 | HEATR5B | 0.4368 |
| ENSP00000251595 | HBA2 | 0.4368 |
| ENSP00000415032 | GTF2H5 | 0.4368 |
| ENSP00000335620 | GSTA1 | 0.4368 |
| ENSP00000293662 | GRASP | 0.4368 |
| ENSP00000264717 | GCKR | 0.4368 |
| ENSP00000468977 | FLT3LG | 0.4368 |
| ENSP00000320309 | FARSA | 0.4368 |
| ENSP00000384396 | FAM58A | 0.4368 |

| | | |
|---|---|---|
| ENSP00000334145 | F3 | 0.4368 |
| ENSP00000369677 | EMILIN1 | 0.4368 |
| ENSP00000309175 | EIF1AD | 0.4368 |
| ENSP00000401566 | ECD | 0.4368 |
| ENSP00000407323 | EBNA1BP2 | 0.4368 |
| ENSP00000452702 | DUSP13 | 0.4368 |
| ENSP00000311399 | DSCR3 | 0.4368 |
| ENSP00000261383 | DNAH3 | 0.4368 |
| ENSP00000326219 | DHRS4 | 0.4368 |
| ENSP00000337450 | CYP3A7 | 0.4368 |
| ENSP00000222982 | CYP3A5 | 0.4368 |
| ENSP00000325822 | CYP11B2 | 0.4368 |
| ENSP00000006053 | CX3CL1 | 0.4368 |
| ENSP00000319464 | CPN2 | 0.4368 |
| ENSP00000347549 | CPA5 | 0.4368 |
| ENSP00000341640 | COL9A3 | 0.4368 |
| ENSP00000356182 | CNKSR3 | 0.4368 |
| ENSP00000273986 | CISD2 | 0.4368 |
| ENSP00000356579 | CEP350 | 0.4368 |
| ENSP00000416445 | CCDC120 | 0.4368 |
| ENSP00000273145 | CCBP2 | 0.4368 |
| ENSP00000398391 | CC2D2A | 0.4368 |
| ENSP00000259199 | CBWD2 | 0.4368 |
| ENSP00000364859 | CARD17 | 0.4368 |
| ENSP00000222125 | CAPS | 0.4368 |
| ENSP00000400882 | CAPNS2 | 0.4368 |
| ENSP00000271971 | CAPN9 | 0.4368 |
| ENSP00000263867 | CAPG | 0.4368 |
| ENSP00000348300 | BTG4 | 0.4368 |
| ENSP00000261721 | BTBD1 | 0.4368 |
| ENSP00000216807 | BRMS1L | 0.4368 |
| ENSP00000359371 | BHLHE23 | 0.4368 |
| ENSP00000265471 | B3GAT3 | 0.4368 |
| ENSP00000273859 | ATP10D | 0.4368 |
| ENSP00000366475 | ATG2A | 0.4368 |
| ENSP00000345420 | ASCL4 | 0.4368 |
| ENSP00000396747 | ANKRD60 | 0.4368 |
| ENSP00000357112 | AIM2 | 0.4368 |
| ENSP00000317578 | ADRBK2 | 0.4368 |
| ENSP00000315118 | ADCK4 | 0.4368 |
| ENSP00000316674 | ACTL9 | 0.4368 |
| ENSP00000278618 | AASDHPPT | 0.4368 |
| ENSP00000262525 | ZNF629 | 0.4342 |
| ENSP00000309161 | ZNF471 | 0.4342 |
| ENSP00000418210 | ZMYND8 | 0.4342 |
| ENSP00000386764 | ZC3H6 | 0.4342 |
| ENSP00000356792 | XCL1 | 0.4342 |
| ENSP00000386878 | WIPF3 | 0.4342 |
| ENSP00000401435 | VPS53 | 0.4342 |
| ENSP00000343838 | UGT1A10 | 0.4342 |
| ENSP00000348565 | UBA5 | 0.4342 |
| ENSP00000308925 | TLR10 | 0.4342 |
| ENSP00000327323 | THNSL2 | 0.4342 |

| | | |
|---|---|---|
| ENSP00000411197 | TBC1D9 | 0.4342 |
| ENSP00000266556 | TAPBPL | 0.4342 |
| ENSP00000334280 | TACC2 | 0.4342 |
| ENSP00000321988 | SULT1A1 | 0.4342 |
| ENSP00000219334 | SMPD3 | 0.4342 |
| ENSP00000307252 | SMOX | 0.4342 |
| ENSP00000381089 | SLC35E3 | 0.4342 |
| ENSP00000264451 | SLC30A9 | 0.4342 |
| ENSP00000417686 | SLBP | 0.4342 |
| ENSP00000317817 | SH2D3C | 0.4342 |
| ENSP00000342850 | SERPINA6 | 0.4342 |
| ENSP00000382767 | SEL1L3 | 0.4342 |
| ENSP00000340463 | S100A14 | 0.4342 |
| ENSP00000322396 | RRS1 | 0.4342 |
| ENSP00000259469 | RPL35 | 0.4342 |
| ENSP00000440272 | RP13-512J5.1 | 0.4342 |
| ENSP00000242719 | RNF11 | 0.4342 |
| ENSP00000339381 | RGS12 | 0.4342 |
| ENSP00000361010 | REXO4 | 0.4342 |
| ENSP00000352668 | RBM12 | 0.4342 |
| ENSP00000345341 | PTGES2 | 0.4342 |
| ENSP00000245457 | PTGER2 | 0.4342 |
| ENSP00000292513 | PTGER1 | 0.4342 |
| ENSP00000367164 | PRR20A | 0.4342 |
| ENSP00000361009 | PREX1 | 0.4342 |
| ENSP00000397050 | PPM1N | 0.4342 |
| ENSP00000264775 | PPAP2A | 0.4342 |
| ENSP00000325296 | PKD2L1 | 0.4342 |
| ENSP00000309542 | PGA5 | 0.4342 |
| ENSP00000282077 | PDK1 | 0.4342 |
| ENSP00000307854 | PCMTD2 | 0.4342 |
| ENSP00000264360 | PCDH10 | 0.4342 |
| ENSP00000343877 | PAQR5 | 0.4342 |
| ENSP00000337946 | PACRG | 0.4342 |
| ENSP00000363643 | P2RY4 | 0.4342 |
| ENSP00000336764 | OPRL1 | 0.4342 |
| ENSP00000247271 | OMG | 0.4342 |
| ENSP00000346659 | OCA2 | 0.4342 |
| ENSP00000244766 | NRN1 | 0.4342 |
| ENSP00000233557 | NRBP1 | 0.4342 |
| ENSP00000356958 | NR1I3 | 0.4342 |
| ENSP00000168977 | NMRK2 | 0.4342 |
| ENSP00000260361 | NDUFAF1 | 0.4342 |
| ENSP00000296543 | NAA15 | 0.4342 |
| ENSP00000323047 | MTG1 | 0.4342 |
| ENSP00000355265 | MT-ATP8 | 0.4342 |
| ENSP00000239761 | MRC1 | 0.4342 |
| ENSP00000465894 | MPPE1 | 0.4342 |
| ENSP00000250896 | MKNK2 | 0.4342 |
| ENSP00000317087 | MARCH8 | 0.4342 |
| ENSP00000343706 | MAGEH1 | 0.4342 |
| ENSP00000359301 | MAGEA3 | 0.4342 |
| ENSP00000217246 | MACROD2 | 0.4342 |

| | | |
|---|---|---|
| ENSP00000344470 | LRRC39 | 0.4342 |
| ENSP00000383923 | LIPN | 0.4342 |
| ENSP00000447488 | LIN7A | 0.4342 |
| ENSP00000328358 | KRT79 | 0.4342 |
| ENSP00000352064 | KLRC1 | 0.4342 |
| ENSP00000469315 | KLK15 | 0.4342 |
| ENSP00000296343 | KIAA0226 | 0.4342 |
| ENSP00000310568 | KCNK10 | 0.4342 |
| ENSP00000344488 | JPH1 | 0.4342 |
| ENSP00000356468 | IVNS1ABP | 0.4342 |
| ENSP00000254654 | ILKAP | 0.4342 |
| ENSP00000409000 | IFNL3 | 0.4342 |
| ENSP00000290219 | IFNGR2 | 0.4342 |
| ENSP00000411940 | IFNA17 | 0.4342 |
| ENSP00000277517 | IDI2 | 0.4342 |
| ENSP00000258400 | HTR2B | 0.4342 |
| ENSP00000466799 | HSD17B1 | 0.4342 |
| ENSP00000355167 | HORMAD1 | 0.4342 |
| ENSP00000357189 | HDGF | 0.4342 |
| ENSP00000086933 | GSC2 | 0.4342 |
| ENSP00000270257 | GEMIN7 | 0.4342 |
| ENSP00000226798 | FRG1 | 0.4342 |
| ENSP00000260008 | FHDC1 | 0.4342 |
| ENSP00000363036 | FBXW2 | 0.4342 |
| ENSP00000261755 | FAH | 0.4342 |
| ENSP00000306356 | ENC1 | 0.4342 |
| ENSP00000365643 | DOCK9 | 0.4342 |
| ENSP00000383909 | DMRTA2 | 0.4342 |
| ENSP00000205143 | DLL3 | 0.4342 |
| ENSP00000419005 | DIEXF | 0.4342 |
| ENSP00000384744 | DERL3 | 0.4342 |
| ENSP00000322524 | DCTPP1 | 0.4342 |
| ENSP00000318867 | CYP8B1 | 0.4342 |
| ENSP00000368079 | CYP4V2 | 0.4342 |
| ENSP00000160373 | CTTNBP2 | 0.4342 |
| ENSP00000359114 | COL11A1 | 0.4342 |
| ENSP00000377914 | CIAPIN1 | 0.4342 |
| ENSP00000385026 | CHRNA2 | 0.4342 |
| ENSP00000373998 | CCDC64B | 0.4342 |
| ENSP00000222693 | CAV2 | 0.4342 |
| ENSP00000261448 | CASQ2 | 0.4342 |
| ENSP00000301050 | CACNB3 | 0.4342 |
| ENSP00000294360 | C1orf123 | 0.4342 |
| ENSP00000230340 | BYSL | 0.4342 |
| ENSP00000338862 | BRIX1 | 0.4342 |
| ENSP00000304151 | BOP1 | 0.4342 |
| ENSP00000296424 | BDH2 | 0.4342 |
| ENSP00000419371 | AZI2 | 0.4342 |
| ENSP00000299178 | AVPR1A | 0.4342 |
| ENSP00000361306 | ATG4A | 0.4342 |
| ENSP00000343126 | ARID3B | 0.4342 |
| ENSP00000354610 | AMY2B | 0.4342 |
| ENSP00000272647 | AMMECR1L | 0.4342 |

| | | |
|---|---|---|
| ENSP00000357880 | AMD1 | 0.4342 |
| ENSP00000315167 | ALOX12B | 0.4342 |
| ENSP00000284987 | ADAMTS5 | 0.4342 |
| ENSP00000334300 | ACTL7A | 0.4342 |
| ENSP00000268129 | ABHD2 | 0.4342 |
| ENSP00000395902 | CCNYL2 | 0.4342 |
| ENSP00000287538 | ZIC3 | 0.4316 |
| ENSP00000220669 | ZFAND1 | 0.4316 |
| ENSP00000225298 | UTP18 | 0.4316 |
| ENSP00000272638 | UBXN4 | 0.4316 |
| ENSP00000346916 | TRIM6-TRIM34 | 0.4316 |
| ENSP00000261180 | TRHDE | 0.4316 |
| ENSP00000393292 | TOR1AIP1 | 0.4316 |
| ENSP00000310263 | TNFRSF10D | 0.4316 |
| ENSP00000334962 | TMPRSS6 | 0.4316 |
| ENSP00000283916 | TMPRSS11D | 0.4316 |
| ENSP00000187762 | TMEM38A | 0.4316 |
| ENSP00000257262 | TMEM258 | 0.4316 |
| ENSP00000440638 | TMEM216 | 0.4316 |
| ENSP00000304467 | THOP1 | 0.4316 |
| ENSP00000355736 | TCTE3 | 0.4316 |
| ENSP00000339973 | TBC1D28 | 0.4316 |
| ENSP00000455547 | TBC1D24 | 0.4316 |
| ENSP00000403636 | SYNGAP1 | 0.4316 |
| ENSP00000222002 | SULT2A1 | 0.4316 |
| ENSP00000455643 | ST20-MTHFS | 0.4316 |
| ENSP00000346959 | SLC38A6 | 0.4316 |
| ENSP00000321498 | SLC37A3 | 0.4316 |
| ENSP00000390722 | SLC25A17 | 0.4316 |
| ENSP00000310490 | SLC16A11 | 0.4316 |
| ENSP00000402152 | SLC12A3 | 0.4316 |
| ENSP00000370074 | SERPINB9 | 0.4316 |
| ENSP00000283752 | SERPINB3 | 0.4316 |
| ENSP00000298841 | SERPINA4 | 0.4316 |
| ENSP00000266214 | SCARF2 | 0.4316 |
| ENSP00000425421 | SATL1 | 0.4316 |
| ENSP00000328708 | RXFP3 | 0.4316 |
| ENSP00000339145 | RRP1B | 0.4316 |
| ENSP00000360031 | RRP12 | 0.4316 |
| ENSP00000414321 | RPS24 | 0.4316 |
| ENSP00000380156 | RPL32 | 0.4316 |
| ENSP00000455952 | RP13-279N23.2 | 0.4316 |
| ENSP00000416453 | RP11-287D1.3 | 0.4316 |
| ENSP00000303554 | PTPN9 | 0.4316 |
| ENSP00000342385 | PTGES | 0.4316 |
| ENSP00000364697 | PROZ | 0.4316 |
| ENSP00000392147 | PPP6C | 0.4316 |
| ENSP00000330190 | POU6F1 | 0.4316 |
| ENSP00000320337 | PLA2G16 | 0.4316 |
| ENSP00000287600 | PDE6D | 0.4316 |
| ENSP00000380855 | PDCD1LG2 | 0.4316 |
| ENSP00000327557 | PAPL | 0.4316 |
| ENSP00000302108 | PANK1 | 0.4316 |

| | | |
|---|---|---|
| ENSP00000315410 | OSBPL3 | 0.4316 |
| ENSP00000242104 | OCM | 0.4316 |
| ENSP00000387310 | NOTUM | 0.4316 |
| ENSP00000349145 | MYO16 | 0.4316 |
| ENSP00000324834 | MUC3A | 0.4316 |
| ENSP00000360365 | MCTS1 | 0.4316 |
| ENSP00000273067 | MARCH4 | 0.4316 |
| ENSP00000348959 | MAN1A2 | 0.4316 |
| ENSP00000035383 | LRRC7 | 0.4316 |
| ENSP00000294507 | LAPTM5 | 0.4316 |
| ENSP00000252250 | KRT6C | 0.4316 |
| ENSP00000448220 | KRT4 | 0.4316 |
| ENSP00000417303 | KLF8 | 0.4316 |
| ENSP00000334181 | KDM4D | 0.4316 |
| ENSP00000362328 | KCNK17 | 0.4316 |
| ENSP00000312631 | INSM1 | 0.4316 |
| ENSP00000265967 | HPS5 | 0.4316 |
| ENSP00000358151 | HIST2H2BE | 0.4316 |
| ENSP00000352252 | HIST1H3J | 0.4316 |
| ENSP00000348706 | HIST1H2BH | 0.4316 |
| ENSP00000252951 | HBZ | 0.4316 |
| ENSP00000322421 | HBA1 | 0.4316 |
| ENSP00000254810 | H3F3B | 0.4316 |
| ENSP00000333193 | GJC1 | 0.4316 |
| ENSP00000281950 | GEMIN6 | 0.4316 |
| ENSP00000249598 | GDF2 | 0.4316 |
| ENSP00000265012 | GCNT2 | 0.4316 |
| ENSP00000450281 | GATC | 0.4316 |
| ENSP00000363317 | FRMPD2 | 0.4316 |
| ENSP00000356723 | FMO4 | 0.4316 |
| ENSP00000376652 | EVL | 0.4316 |
| ENSP00000429190 | ENDOV | 0.4316 |
| ENSP00000286523 | ELMSAN1 | 0.4316 |
| ENSP00000360129 | EFCAB7 | 0.4316 |
| ENSP00000307143 | DONSON | 0.4316 |
| ENSP00000356985 | DEDD | 0.4316 |
| ENSP00000357103 | DARC | 0.4316 |
| ENSP00000455329 | CRIP1 | 0.4316 |
| ENSP00000362734 | CRB2 | 0.4316 |
| ENSP00000222482 | CPA4 | 0.4316 |
| ENSP00000292907 | COX7A1 | 0.4316 |
| ENSP00000361616 | COMTD1 | 0.4316 |
| ENSP00000272602 | CNGA3 | 0.4316 |
| ENSP00000336571 | CLDN2 | 0.4316 |
| ENSP00000339867 | CLCN1 | 0.4316 |
| ENSP00000276410 | CHRNA6 | 0.4316 |
| ENSP00000299565 | CHRNA5 | 0.4316 |
| ENSP00000361112 | CHRDL1 | 0.4316 |
| ENSP00000353048 | CCT8L2 | 0.4316 |
| ENSP00000221740 | CASP14 | 0.4316 |
| ENSP00000296777 | CARTPT | 0.4316 |
| ENSP00000375767 | CARD8 | 0.4316 |
| ENSP00000005284 | CACNG3 | 0.4316 |

| | | |
|---|---|---|
| ENSP00000320081 | C3orf58 | 0.4316 |
| ENSP00000261714 | BLMH | 0.4316 |
| ENSP00000295240 | BBS5 | 0.4316 |
| ENSP00000299575 | ATMIN | 0.4316 |
| ENSP00000348769 | ARIH2 | 0.4316 |
| ENSP00000350078 | ARID5A | 0.4316 |
| ENSP00000258530 | APPL2 | 0.4316 |
| ENSP00000321617 | ANKZF1 | 0.4316 |
| ENSP00000251535 | ALOX12 | 0.4316 |
| ENSP00000389289 | AKR1B15 | 0.4316 |
| ENSP00000316109 | AGGF1 | 0.4316 |
| ENSP00000280155 | ADRA2A | 0.4316 |
| ENSP00000329647 | ACTL10 | 0.4316 |
| ENSP00000377496 | ACD | 0.4316 |
| ENSP00000450694 | ENSG00000258508 | 0.4316 |
| ENSP00000361499 | YIPF3 | 0.4289 |
| ENSP00000310405 | XCR1 | 0.4289 |
| ENSP00000327716 | WRB | 0.4289 |
| ENSP00000314193 | WDR75 | 0.4289 |
| ENSP00000351100 | WDR55 | 0.4289 |
| ENSP00000407818 | USP46 | 0.4289 |
| ENSP00000362513 | UGT1A5 | 0.4289 |
| ENSP00000216129 | TTLL12 | 0.4289 |
| ENSP00000298355 | TRIM9 | 0.4289 |
| ENSP00000291574 | TRAPPC10 | 0.4289 |
| ENSP00000212355 | TGFBR3 | 0.4289 |
| ENSP00000329256 | TBC1D3F | 0.4289 |
| ENSP00000339917 | TAF9B | 0.4289 |
| ENSP00000290607 | STARD9 | 0.4289 |
| ENSP00000314491 | SRRT | 0.4289 |
| ENSP00000335612 | SPATA21 | 0.4289 |
| ENSP00000347646 | SOX12 | 0.4289 |
| ENSP00000363392 | SNX12 | 0.4289 |
| ENSP00000305974 | SLCO1A2 | 0.4289 |
| ENSP00000434364 | SLC6A5 | 0.4289 |
| ENSP00000295736 | SLC4A7 | 0.4289 |
| ENSP00000219320 | SLC38A7 | 0.4289 |
| ENSP00000327133 | SLC35A4 | 0.4289 |
| ENSP00000352216 | SLC2A10 | 0.4289 |
| ENSP00000377112 | SLC12A8 | 0.4289 |
| ENSP00000264930 | SLC12A7 | 0.4289 |
| ENSP00000342962 | SERINC1 | 0.4289 |
| ENSP00000002829 | SEMA3F | 0.4289 |
| ENSP00000439918 | RBM7 | 0.4289 |
| ENSP00000356621 | RASAL2 | 0.4289 |
| ENSP00000321271 | PXMP2 | 0.4289 |
| ENSP00000381302 | PPAPDC1A | 0.4289 |
| ENSP00000373399 | PIP5KL1 | 0.4289 |
| ENSP00000255389 | PEMT | 0.4289 |
| ENSP00000309142 | PDE12 | 0.4289 |
| ENSP00000368887 | PAK1IP1 | 0.4289 |
| ENSP00000332170 | P4HA3 | 0.4289 |
| ENSP00000396722 | NTM | 0.4289 |

| | | |
|---|---|---|
| ENSP00000339377 | NPY5R | 0.4289 |
| ENSP00000332591 | NPY2R | 0.4289 |
| ENSP00000337889 | MXD4 | 0.4289 |
| ENSP00000363985 | MTMR8 | 0.4289 |
| ENSP00000250124 | MPDU1 | 0.4289 |
| ENSP00000223054 | MOSPD3 | 0.4289 |
| ENSP00000406674 | MOGAT1 | 0.4289 |
| ENSP00000376827 | MKS1 | 0.4289 |
| ENSP00000309790 | MIS18BP1 | 0.4289 |
| ENSP00000371607 | MIPEP | 0.4289 |
| ENSP00000385527 | MFSD2B | 0.4289 |
| ENSP00000323264 | MARVELD2 | 0.4289 |
| ENSP00000353655 | MAN2A2 | 0.4289 |
| ENSP00000387928 | MAGIX | 0.4289 |
| ENSP00000386043 | LTBP1 | 0.4289 |
| ENSP00000353538 | LRRCC1 | 0.4289 |
| ENSP00000304923 | LRRC28 | 0.4289 |
| ENSP00000221459 | LIN7B | 0.4289 |
| ENSP00000378856 | LGALS9 | 0.4289 |
| ENSP00000416696 | KIAA1432 | 0.4289 |
| ENSP00000360626 | KCNG1 | 0.4289 |
| ENSP00000362071 | JPH2 | 0.4289 |
| ENSP00000382204 | JMJD1C | 0.4289 |
| ENSP00000263370 | ITPKC | 0.4289 |
| ENSP00000262457 | INVS | 0.4289 |
| ENSP00000434466 | INTS4 | 0.4289 |
| ENSP00000369553 | IFNA8 | 0.4289 |
| ENSP00000342560 | HRH3 | 0.4289 |
| ENSP00000439493 | HIST1H3C | 0.4289 |
| ENSP00000349873 | HIST1H2AL | 0.4289 |
| ENSP00000302276 | HEXIM2 | 0.4289 |
| ENSP00000350378 | GOLGA7 | 0.4289 |
| ENSP00000262366 | GLIS2 | 0.4289 |
| ENSP00000359490 | GBP4 | 0.4289 |
| ENSP00000269373 | FN3KRP | 0.4289 |
| ENSP00000357429 | FABP7 | 0.4289 |
| ENSP00000364035 | FAAH2 | 0.4289 |
| ENSP00000326267 | EFCAB11 | 0.4289 |
| ENSP00000370022 | EBF4 | 0.4289 |
| ENSP00000222219 | DNASE2 | 0.4289 |
| ENSP00000344674 | DHRS2 | 0.4289 |
| ENSP00000219481 | DECR2 | 0.4289 |
| ENSP00000361232 | DDX31 | 0.4289 |
| ENSP00000351147 | DCAF4 | 0.4289 |
| ENSP00000337439 | DACT1 | 0.4289 |
| ENSP00000360372 | CYP2C19 | 0.4289 |
| ENSP00000301146 | CYP2A7 | 0.4289 |
| ENSP00000216416 | CNIH | 0.4289 |
| ENSP00000328674 | CLDN6 | 0.4289 |
| ENSP00000428780 | CLDN23 | 0.4289 |
| ENSP00000299736 | CENPV | 0.4289 |
| ENSP00000436318 | CCS | 0.4289 |
| ENSP00000342344 | CCNYL1 | 0.4289 |

| | | |
|---|---|---|
| ENSP00000225842 | CCL1 | 0.4289 |
| ENSP00000318429 | CCDC114 | 0.4289 |
| ENSP00000303092 | CACNG5 | 0.4289 |
| ENSP00000365522 | C9orf64 | 0.4289 |
| ENSP00000286122 | BANP | 0.4289 |
| ENSP00000369141 | APTX | 0.4289 |
| ENSP00000371347 | ANGPT4 | 0.4289 |
| ENSP00000316590 | AL445665.1 | 0.4289 |
| ENSP00000363208 | AGAP7 | 0.4289 |
| ENSP00000238855 | AFTPH | 0.4289 |
| ENSP00000312250 | ADPGK | 0.4289 |
| ENSP00000255082 | ACY3 | 0.4289 |
| ENSP00000273596 | ABHD14A | 0.4289 |
| ENSP00000217130 | ZNF512B | 0.4263 |
| ENSP00000378165 | ZNF207 | 0.4263 |
| ENSP00000308759 | ZBTB21 | 0.4263 |
| ENSP00000354822 | XAF1 | 0.4263 |
| ENSP00000340526 | UTS2B | 0.4263 |
| ENSP00000254803 | UTP3 | 0.4263 |
| ENSP00000343526 | USP1 | 0.4263 |
| ENSP00000337040 | UNC119 | 0.4263 |
| ENSP00000366434 | UIMC1 | 0.4263 |
| ENSP00000387888 | UGT2A2 | 0.4263 |
| ENSP00000303174 | UGT1A6 | 0.4263 |
| ENSP00000365938 | UGGT2 | 0.4263 |
| ENSP00000203001 | TRMT6 | 0.4263 |
| ENSP00000432743 | TP53AIP1 | 0.4263 |
| ENSP00000320236 | TIMM22 | 0.4263 |
| ENSP00000247881 | TAS2R4 | 0.4263 |
| ENSP00000269142 | TAF4B | 0.4263 |
| ENSP00000327072 | TAF1A | 0.4263 |
| ENSP00000344041 | SYNGR4 | 0.4263 |
| ENSP00000371388 | SRXN1 | 0.4263 |
| ENSP00000346298 | SLC6A20 | 0.4263 |
| ENSP00000339260 | SLC6A13 | 0.4263 |
| ENSP00000393557 | SLC4A4 | 0.4263 |
| ENSP00000260649 | SLC3A1 | 0.4263 |
| ENSP00000358565 | SLC35A1 | 0.4263 |
| ENSP00000379836 | SLC30A5 | 0.4263 |
| ENSP00000005587 | SKAP2 | 0.4263 |
| ENSP00000343445 | SERPINB4 | 0.4263 |
| ENSP00000324857 | SEMA6D | 0.4263 |
| ENSP00000369380 | SCML1 | 0.4263 |
| ENSP00000370394 | RUFY3 | 0.4263 |
| ENSP00000460871 | RSL1D1 | 0.4263 |
| ENSP00000328340 | RNF135 | 0.4263 |
| ENSP00000369162 | RIOK1 | 0.4263 |
| ENSP00000360455 | RHOXF2B | 0.4263 |
| ENSP00000264400 | RASGEF1B | 0.4263 |
| ENSP00000262305 | RAB11FIP3 | 0.4263 |
| ENSP00000419503 | PTPLAD2 | 0.4263 |
| ENSP00000261875 | PTPLAD1 | 0.4263 |
| ENSP00000258324 | PIGC | 0.4263 |

| | | |
|---|---|---|
| ENSP00000362116 | PGC | 0.4263 |
| ENSP00000225873 | PEX12 | 0.4263 |
| ENSP00000254908 | PCBD2 | 0.4263 |
| ENSP00000325618 | PARP10 | 0.4263 |
| ENSP00000316649 | OSBPL2 | 0.4263 |
| ENSP00000305288 | NLGN2 | 0.4263 |
| ENSP00000396538 | NFAT5 | 0.4263 |
| ENSP00000416341 | NELL2 | 0.4263 |
| ENSP00000281871 | MZT2B | 0.4263 |
| ENSP00000367049 | MZT1 | 0.4263 |
| ENSP00000462945 | MSL1 | 0.4263 |
| ENSP00000365005 | MAP3K19 | 0.4263 |
| ENSP00000215637 | MADCAM1 | 0.4263 |
| ENSP00000369251 | MAB21L1 | 0.4263 |
| ENSP00000309463 | LIPT2 | 0.4263 |
| ENSP00000383901 | LIPM | 0.4263 |
| ENSP00000305653 | LETM1 | 0.4263 |
| ENSP00000252252 | KRT6B | 0.4263 |
| ENSP00000263372 | KCNK6 | 0.4263 |
| ENSP00000240662 | KCNJ8 | 0.4263 |
| ENSP00000333496 | KCND2 | 0.4263 |
| ENSP00000357753 | IVL | 0.4263 |
| ENSP00000379712 | IQSEC2 | 0.4263 |
| ENSP00000381064 | INTS10 | 0.4263 |
| ENSP00000358963 | HTR1B | 0.4263 |
| ENSP00000392896 | HSBP1 | 0.4263 |
| ENSP00000326649 | HPS1 | 0.4263 |
| ENSP00000247815 | HELB | 0.4263 |
| ENSP00000437968 | HEATR5A | 0.4263 |
| ENSP00000199708 | HBQ1 | 0.4263 |
| ENSP00000384597 | GTF2A1L | 0.4263 |
| ENSP00000263269 | GRIN2D | 0.4263 |
| ENSP00000297469 | GPER | 0.4263 |
| ENSP00000351552 | GATAD2A | 0.4263 |
| ENSP00000313309 | FUZ | 0.4263 |
| ENSP00000368207 | FTHL17 | 0.4263 |
| ENSP00000357682 | FANK1 | 0.4263 |
| ENSP00000217429 | FAM83D | 0.4263 |
| ENSP00000335808 | FAM192A | 0.4263 |
| ENSP00000263062 | EPC1 | 0.4263 |
| ENSP00000265800 | EPB49 | 0.4263 |
| ENSP00000333917 | DUSP18 | 0.4263 |
| ENSP00000340609 | DUPD1 | 0.4263 |
| ENSP00000257189 | DSG3 | 0.4263 |
| ENSP00000322181 | DPM2 | 0.4263 |
| ENSP00000316240 | DNAJC14 | 0.4263 |
| ENSP00000365397 | DHRS3 | 0.4263 |
| ENSP00000366237 | DFFA | 0.4263 |
| ENSP00000258772 | DDX56 | 0.4263 |
| ENSP00000269703 | CYP4F22 | 0.4263 |
| ENSP00000222382 | CYP3A43 | 0.4263 |
| ENSP00000001146 | CYP26B1 | 0.4263 |
| ENSP00000342007 | CYP1A2 | 0.4263 |

| | | |
|---|---|---|
| ENSP00000297135 | COG5 | 0.4263 |
| ENSP00000418741 | CNTNAP4 | 0.4263 |
| ENSP00000297668 | CNTNAP3 | 0.4263 |
| ENSP00000261514 | CLCN3 | 0.4263 |
| ENSP00000261751 | CHRNB4 | 0.4263 |
| ENSP00000357461 | CHRNB2 | 0.4263 |
| ENSP00000300113 | CHP2 | 0.4263 |
| ENSP00000403649 | CGB8 | 0.4263 |
| ENSP00000317842 | CES2 | 0.4263 |
| ENSP00000290122 | CELA3A | 0.4263 |
| ENSP00000365075 | CELA2B | 0.4263 |
| ENSP00000371278 | CDC37L1 | 0.4263 |
| ENSP00000384040 | CCDC85A | 0.4263 |
| ENSP00000364464 | C9orf3 | 0.4263 |
| ENSP00000356311 | C1orf106 | 0.4263 |
| ENSP00000297161 | BMPER | 0.4263 |
| ENSP00000307713 | BDKRB2 | 0.4263 |
| ENSP00000435210 | BCLAF1 | 0.4263 |
| ENSP00000283684 | ATP8B1 | 0.4263 |
| ENSP00000304891 | ATP6V1E2 | 0.4263 |
| ENSP00000417914 | ASB11 | 0.4263 |
| ENSP00000368189 | ARID3C | 0.4263 |
| ENSP00000372654 | AQP4 | 0.4263 |
| ENSP00000227667 | APOC3 | 0.4263 |
| ENSP00000409722 | AL592183.1 | 0.4263 |
| ENSP00000364217 | AGER | 0.4263 |
| ENSP00000415452 | AGAP8 | 0.4263 |
| ENSP00000363207 | AGAP5 | 0.4263 |
| ENSP00000341785 | ADIPOR1 | 0.4263 |
| ENSP00000282641 | A1CF | 0.4263 |
| ENSP00000378286 | ZBTB3 | 0.4237 |
| ENSP00000326200 | ZBTB11 | 0.4237 |
| ENSP00000282344 | USP12 | 0.4237 |
| ENSP00000252597 | USHBP1 | 0.4237 |
| ENSP00000346768 | UGT1A9 | 0.4237 |
| ENSP00000418532 | UGT1A3 | 0.4237 |
| ENSP00000351206 | TXLNB | 0.4237 |
| ENSP00000299866 | TVP23A | 0.4237 |
| ENSP00000284995 | TSEN2 | 0.4237 |
| ENSP00000356411 | TROVE2 | 0.4237 |
| ENSP00000302120 | TRIM8 | 0.4237 |
| ENSP00000305161 | TRIM56 | 0.4237 |
| ENSP00000371693 | TNFRSF19 | 0.4237 |
| ENSP00000402698 | TMEM30C | 0.4237 |
| ENSP00000380747 | TMEM209 | 0.4237 |
| ENSP00000261296 | TGDS | 0.4237 |
| ENSP00000295201 | TEKT4 | 0.4237 |
| ENSP00000264037 | TECTA | 0.4237 |
| ENSP00000410111 | TBC1D26 | 0.4237 |
| ENSP00000350162 | SYCP2 | 0.4237 |
| ENSP00000311493 | STON1-GTF2A1L | 0.4237 |
| ENSP00000428756 | STAU2 | 0.4237 |
| ENSP00000350198 | SSTR2 | 0.4237 |

| | | |
|---|---|---|
| ENSP00000364430 | SP5 | 0.4237 |
| ENSP00000216294 | SNAPC1 | 0.4237 |
| ENSP00000247225 | SGPP1 | 0.4237 |
| ENSP00000308339 | SEC16B | 0.4237 |
| ENSP00000256190 | SBF2 | 0.4237 |
| ENSP00000461728 | RP11-212D19.4 | 0.4237 |
| ENSP00000257700 | RINT1 | 0.4237 |
| ENSP00000465742 | RASGRP4 | 0.4237 |
| ENSP00000363350 | PTPN20B | 0.4237 |
| ENSP00000222381 | PON1 | 0.4237 |
| ENSP00000318914 | PNMA1 | 0.4237 |
| ENSP00000291009 | PIP | 0.4237 |
| ENSP00000217446 | PIGU | 0.4237 |
| ENSP00000387966 | PGBD3 | 0.4237 |
| ENSP00000357547 | NUP210L | 0.4237 |
| ENSP00000265459 | NRXN2 | 0.4237 |
| ENSP00000348395 | NPEPL1 | 0.4237 |
| ENSP00000373518 | NOX5 | 0.4237 |
| ENSP00000268802 | NOB1 | 0.4237 |
| ENSP00000337452 | NIPA1 | 0.4237 |
| ENSP00000353375 | MAP4 | 0.4237 |
| ENSP00000387077 | LYPD6B | 0.4237 |
| ENSP00000329932 | LGALS9C | 0.4237 |
| ENSP00000382021 | LCMT1 | 0.4237 |
| ENSP00000256644 | LAMTOR5 | 0.4237 |
| ENSP00000438526 | KLHL35 | 0.4237 |
| ENSP00000368813 | KLHL34 | 0.4237 |
| ENSP00000221200 | KCTD9 | 0.4237 |
| ENSP00000371985 | KCNA1 | 0.4237 |
| ENSP00000218436 | ITIH6 | 0.4237 |
| ENSP00000254801 | IGJ | 0.4237 |
| ENSP00000379908 | ICA1 | 0.4237 |
| ENSP00000338457 | HPS4 | 0.4237 |
| ENSP00000012134 | HIVEP2 | 0.4237 |
| ENSP00000330307 | HIST1H2AK | 0.4237 |
| ENSP00000238379 | HDHD3 | 0.4237 |
| ENSP00000204726 | GOLGA3 | 0.4237 |
| ENSP00000246802 | GLTSCR2 | 0.4237 |
| ENSP00000396615 | GLB1L3 | 0.4237 |
| ENSP00000340811 | GJB5 | 0.4237 |
| ENSP00000365920 | GCNT1 | 0.4237 |
| ENSP00000351631 | FAM76B | 0.4237 |
| ENSP00000267750 | EMC4 | 0.4237 |
| ENSP00000318147 | EDEM3 | 0.4237 |
| ENSP00000366672 | DTD1 | 0.4237 |
| ENSP00000343665 | DOC2B | 0.4237 |
| ENSP00000222122 | DBP | 0.4237 |
| ENSP00000470318 | CTD-3148I10.9 | 0.4237 |
| ENSP00000297405 | CSMD3 | 0.4237 |
| ENSP00000364691 | CROCC | 0.4237 |
| ENSP00000454500 | COG8 | 0.4237 |
| ENSP00000420443 | CNIH4 | 0.4237 |
| ENSP00000311733 | CMKLR1 | 0.4237 |

| | | |
|---|---|---|
| ENSP00000298912 | CLMN | 0.4237 |
| ENSP00000374145 | CHRNG | 0.4237 |
| ENSP00000352841 | CEP57L1 | 0.4237 |
| ENSP00000332504 | CCR10 | 0.4237 |
| ENSP00000390423 | CATSPER4 | 0.4237 |
| ENSP00000222212 | CACNG7 | 0.4237 |
| ENSP00000282059 | C18orf25 | 0.4237 |
| ENSP00000333769 | BSG | 0.4237 |
| ENSP00000335201 | BCDIN3D | 0.4237 |
| ENSP00000297512 | ASIC3 | 0.4237 |
| ENSP00000369855 | ASB9 | 0.4237 |
| ENSP00000273258 | ARL6IP5 | 0.4237 |
| ENSP00000315136 | APBA3 | 0.4237 |
| ENSP00000360762 | ANKRD1 | 0.4237 |
| ENSP00000333813 | ALG12 | 0.4237 |
| ENSP00000266483 | ALG10 | 0.4237 |
| ENSP00000277491 | AL354898.1 | 0.4237 |
| ENSP00000347581 | AGK | 0.4237 |
| ENSP00000306662 | ADRA1B | 0.4237 |
| ENSP00000348912 | ADAM33 | 0.4237 |
| ENSP00000243903 | ACTR5 | 0.4237 |
| ENSP00000333926 | ABCC5 | 0.4237 |
| ENSP00000262095 | ONECUT2 | 0.4237 |
| ENSP00000299218 | CAND1 | 0.4237 |
| ENSP00000359837 | ZZZ3 | 0.4211 |
| ENSP00000363384 | ZNF593 | 0.4211 |
| ENSP00000313582 | ZNF436 | 0.4211 |
| ENSP00000363257 | ZDHHC18 | 0.4211 |
| ENSP00000347999 | ZC3H7A | 0.4211 |
| ENSP00000349503 | ZBTB14 | 0.4211 |
| ENSP00000339245 | YTHDC1 | 0.4211 |
| ENSP00000261381 | XYLT1 | 0.4211 |
| ENSP00000356793 | XCL2 | 0.4211 |
| ENSP00000258499 | USP44 | 0.4211 |
| ENSP00000362525 | UGT1A7 | 0.4211 |
| ENSP00000456827 | UBE2F | 0.4211 |
| ENSP00000429865 | TVP23C-CDRT4 | 0.4211 |
| ENSP00000321346 | TTLL11 | 0.4211 |
| ENSP00000301021 | TRAPPC2L | 0.4211 |
| ENSP00000358765 | TPBG | 0.4211 |
| ENSP00000386239 | TONSL | 0.4211 |
| ENSP00000272771 | TMEFF2 | 0.4211 |
| ENSP00000462799 | TBC1D29 | 0.4211 |
| ENSP00000332592 | SPAG16 | 0.4211 |
| ENSP00000271227 | SLC44A3 | 0.4211 |
| ENSP00000337561 | SLC43A3 | 0.4211 |
| ENSP00000256689 | SLC38A2 | 0.4211 |
| ENSP00000317382 | SLC36A4 | 0.4211 |
| ENSP00000368648 | SLC30A6 | 0.4211 |
| ENSP00000350024 | SLC29A2 | 0.4211 |
| ENSP00000367102 | SLC22A6 | 0.4211 |
| ENSP00000301891 | SLC22A11 | 0.4211 |
| ENSP00000263160 | SLC17A6 | 0.4211 |

| | | |
|---|---|---|
| ENSP00000406220 | SLC13A5 | 0.4211 |
| ENSP00000393762 | SESN1 | 0.4211 |
| ENSP00000329374 | SERPINA7 | 0.4211 |
| ENSP00000394791 | SENP1 | 0.4211 |
| ENSP00000297029 | SCIN | 0.4211 |
| ENSP00000328472 | S1PR5 | 0.4211 |
| ENSP00000357711 | S100A7 | 0.4211 |
| ENSP00000216036 | RTDR1 | 0.4211 |
| ENSP00000355927 | RPS6KC1 | 0.4211 |
| ENSP00000333948 | RPS19BP1 | 0.4211 |
| ENSP00000427123 | RAI14 | 0.4211 |
| ENSP00000274710 | PSD2 | 0.4211 |
| ENSP00000291281 | PRKD2 | 0.4211 |
| ENSP00000254101 | PRKAB2 | 0.4211 |
| ENSP00000384725 | PRDM6 | 0.4211 |
| ENSP00000349124 | PPP4R2 | 0.4211 |
| ENSP00000308318 | PPP1R3B | 0.4211 |
| ENSP00000265322 | PECR | 0.4211 |
| ENSP00000291565 | PDXK | 0.4211 |
| ENSP00000332576 | OSBP2 | 0.4211 |
| ENSP00000332823 | NR2C2AP | 0.4211 |
| ENSP00000365651 | NPPB | 0.4211 |
| ENSP00000320324 | NPEPPS | 0.4211 |
| ENSP00000215057 | MZF1 | 0.4211 |
| ENSP00000351790 | MYPN | 0.4211 |
| ENSP00000287025 | MTERFD1 | 0.4211 |
| ENSP00000248643 | MTERF | 0.4211 |
| ENSP00000290208 | MRPL10 | 0.4211 |
| ENSP00000219431 | MPG | 0.4211 |
| ENSP00000344242 | LRRC17 | 0.4211 |
| ENSP00000317619 | LACC1 | 0.4211 |
| ENSP00000356123 | KLHDC8A | 0.4211 |
| ENSP00000368881 | KIN | 0.4211 |
| ENSP00000159111 | KDM4B | 0.4211 |
| ENSP00000271915 | KCNN3 | 0.4211 |
| ENSP00000264661 | KCNH4 | 0.4211 |
| ENSP00000319591 | KCND3 | 0.4211 |
| ENSP00000315662 | IQSEC3 | 0.4211 |
| ENSP00000361502 | HYI | 0.4211 |
| ENSP00000351035 | HSD17B11 | 0.4211 |
| ENSP00000271588 | HMCN1 | 0.4211 |
| ENSP00000353581 | HIST1H3E | 0.4211 |
| ENSP00000307705 | HIST1H1E | 0.4211 |
| ENSP00000327431 | HBG1 | 0.4211 |
| ENSP00000292896 | HBE1 | 0.4211 |
| ENSP00000352047 | GULP1 | 0.4211 |
| ENSP00000253458 | GSE1 | 0.4211 |
| ENSP00000313756 | GPR75-ASB3 | 0.4211 |
| ENSP00000272644 | GPR17 | 0.4211 |
| ENSP00000308535 | GOLT1A | 0.4211 |
| ENSP00000335074 | GHRL | 0.4211 |
| ENSP00000238018 | GDA | 0.4211 |
| ENSP00000269856 | FEM1A | 0.4211 |

| | | |
|---|---|---|
| ENSP00000355157 | FCRL2 | 0.4211 |
| ENSP00000361592 | ERMAP | 0.4211 |
| ENSP00000323246 | E2F7 | 0.4211 |
| ENSP00000195654 | DOPEY1 | 0.4211 |
| ENSP00000302843 | DNAJC18 | 0.4211 |
| ENSP00000269945 | DMRTC2 | 0.4211 |
| ENSP00000417706 | DCUN1D2 | 0.4211 |
| ENSP00000360991 | CYP4B1 | 0.4211 |
| ENSP00000333212 | CYP2U1 | 0.4211 |
| ENSP00000408860 | CYP21A2 | 0.4211 |
| ENSP00000293230 | CYGB | 0.4211 |
| ENSP00000271277 | CTTNBP2NL | 0.4211 |
| ENSP00000246891 | CSN1S1 | 0.4211 |
| ENSP00000251102 | CNGB1 | 0.4211 |
| ENSP00000370213 | CLCN4 | 0.4211 |
| ENSP00000290583 | CELF3 | 0.4211 |
| ENSP00000383986 | CDCA7L | 0.4211 |
| ENSP00000357012 | CD244 | 0.4211 |
| ENSP00000324101 | CD151 | 0.4211 |
| ENSP00000369853 | C2CD2 | 0.4211 |
| ENSP00000223122 | C1GALT1 | 0.4211 |
| ENSP00000261700 | C14orf166 | 0.4211 |
| ENSP00000254900 | BRD8 | 0.4211 |
| ENSP00000242067 | BBS9 | 0.4211 |
| ENSP00000400168 | ATP5J2-PTCD1 | 0.4211 |
| ENSP00000306678 | ANKK1 | 0.4211 |
| ENSP00000367631 | ANKEF1 | 0.4211 |
| ENSP00000392513 | AGAP4 | 0.4211 |
| ENSP00000360997 | ADAMTS13 | 0.4211 |
| ENSP00000361608 | ABCC10 | 0.4211 |
| ENSP00000430633 | 61E3.4 | 0.4211 |
| ENSP00000253115 | ZNF426 | 0.4184 |
| ENSP00000290974 | ZFYVE28 | 0.4184 |
| ENSP00000304985 | ZFX | 0.4184 |
| ENSP00000300101 | ZBTB39 | 0.4184 |
| ENSP00000262178 | VIPR2 | 0.4184 |
| ENSP00000333329 | USP17L2 | 0.4184 |
| ENSP00000332737 | UNC5A | 0.4184 |
| ENSP00000305221 | UGT2B4 | 0.4184 |
| ENSP00000382507 | UBXN2B | 0.4184 |
| ENSP00000312356 | TRMT10C | 0.4184 |
| ENSP00000373272 | TRIM71 | 0.4184 |
| ENSP00000361162 | TOE1 | 0.4184 |
| ENSP00000307519 | TMPRSS11E | 0.4184 |
| ENSP00000222284 | TMEM147 | 0.4184 |
| ENSP00000384376 | TDRD15 | 0.4184 |
| ENSP00000263346 | TCF25 | 0.4184 |
| ENSP00000334308 | SYNE3 | 0.4184 |
| ENSP00000336750 | SUPT7L | 0.4184 |
| ENSP00000263997 | SLC7A6OS | 0.4184 |
| ENSP00000395653 | SLC46A1 | 0.4184 |
| ENSP00000360966 | SLC2A6 | 0.4184 |
| ENSP00000259608 | SIT1 | 0.4184 |

| | | |
|---|---|---|
| ENSP00000309186 | SH3RF3 | 0.4184 |
| ENSP00000331368 | SERPINB8 | 0.4184 |
| ENSP00000307607 | SDR16C5 | 0.4184 |
| ENSP00000396915 | SCN1B | 0.4184 |
| ENSP00000449223 | RP11-347C12.3 | 0.4184 |
| ENSP00000355523 | RGS7 | 0.4184 |
| ENSP00000360519 | RBP4 | 0.4184 |
| ENSP00000331211 | RBM43 | 0.4184 |
| ENSP00000223073 | RBM28 | 0.4184 |
| ENSP00000261741 | RBM19 | 0.4184 |
| ENSP00000330276 | PTPN21 | 0.4184 |
| ENSP00000263681 | POLD3 | 0.4184 |
| ENSP00000364249 | PLA2G5 | 0.4184 |
| ENSP00000294338 | PDZK1IP1 | 0.4184 |
| ENSP00000328992 | PAXBP1 | 0.4184 |
| ENSP00000328088 | PAWR | 0.4184 |
| ENSP00000309771 | P2RY6 | 0.4184 |
| ENSP00000376921 | NTNG2 | 0.4184 |
| ENSP00000355077 | NSMCE1 | 0.4184 |
| ENSP00000269228 | NPC1 | 0.4184 |
| ENSP00000380109 | NFKBID | 0.4184 |
| ENSP00000355955 | NENF | 0.4184 |
| ENSP00000446132 | NAV3 | 0.4184 |
| ENSP00000403683 | NABP1 | 0.4184 |
| ENSP00000401867 | MXD3 | 0.4184 |
| ENSP00000423600 | MTX3 | 0.4184 |
| ENSP00000328118 | MCF2L2 | 0.4184 |
| ENSP00000304198 | MBD3L1 | 0.4184 |
| ENSP00000274140 | MARCH6 | 0.4184 |
| ENSP00000389128 | LRRC15 | 0.4184 |
| ENSP00000349629 | LRBA | 0.4184 |
| ENSP00000325929 | LNX2 | 0.4184 |
| ENSP00000347088 | LARGE | 0.4184 |
| ENSP00000265598 | LAMP3 | 0.4184 |
| ENSP00000257951 | KRT84 | 0.4184 |
| ENSP00000427900 | KIF13B | 0.4184 |
| ENSP00000262916 | KCNQ4 | 0.4184 |
| ENSP00000378033 | KCNK4 | 0.4184 |
| ENSP00000368982 | KATNB1 | 0.4184 |
| ENSP00000295980 | IL17RE | 0.4184 |
| ENSP00000412897 | IFNA4 | 0.4184 |
| ENSP00000369566 | IFNA10 | 0.4184 |
| ENSP00000395590 | IFI35 | 0.4184 |
| ENSP00000322924 | HTR1F | 0.4184 |
| ENSP00000263278 | HSD17B14 | 0.4184 |
| ENSP00000299238 | HPS6 | 0.4184 |
| ENSP00000264720 | GTF3C2 | 0.4184 |
| ENSP00000265963 | GTF2H1 | 0.4184 |
| ENSP00000282020 | GRID2 | 0.4184 |
| ENSP00000290374 | GJD2 | 0.4184 |
| ENSP00000335592 | GABRA5 | 0.4184 |
| ENSP00000331411 | FBLN7 | 0.4184 |
| ENSP00000347744 | FAM49A | 0.4184 |

| | | |
|---|---|---|
| ENSP00000298937 | ELP4 | 0.4184 |
| ENSP00000312671 | EHBP1L1 | 0.4184 |
| ENSP00000403932 | EFCAB3 | 0.4184 |
| ENSP00000344235 | DUSP28 | 0.4184 |
| ENSP00000276202 | DOCK11 | 0.4184 |
| ENSP00000314910 | DKK3 | 0.4184 |
| ENSP00000268854 | DDX52 | 0.4184 |
| ENSP00000282957 | CPB1 | 0.4184 |
| ENSP00000366248 | CORT | 0.4184 |
| ENSP00000286809 | CLDN8 | 0.4184 |
| ENSP00000243776 | CHPF | 0.4184 |
| ENSP00000292672 | CELF5 | 0.4184 |
| ENSP00000345133 | CDC42BPG | 0.4184 |
| ENSP00000354416 | CCL28 | 0.4184 |
| ENSP00000364858 | CARD16 | 0.4184 |
| ENSP00000248420 | CACTIN | 0.4184 |
| ENSP00000263413 | C6 | 0.4184 |
| ENSP00000363642 | BMS1 | 0.4184 |
| ENSP00000263368 | BLVRB | 0.4184 |
| ENSP00000344087 | AUTS2 | 0.4184 |
| ENSP00000277458 | ASB6 | 0.4184 |
| ENSP00000371152 | ASAH1 | 0.4184 |
| ENSP00000433427 | ARL15 | 0.4184 |
| ENSP00000297562 | AP5Z1 | 0.4184 |
| ENSP00000353518 | ANKS1A | 0.4184 |
| ENSP00000369927 | AKR1C3 | 0.4184 |
| ENSP00000365227 | AIF1 | 0.4184 |
| ENSP00000363168 | AGAP6 | 0.4184 |
| ENSP00000386069 | ADRA2C | 0.4184 |
| ENSP00000349616 | ADIPOR2 | 0.4184 |
| ENSP00000356567 | ACBD6 | 0.4184 |
| ENSP00000303252 | ZNF804A | 0.4158 |
| ENSP00000334394 | ZNF677 | 0.4158 |
| ENSP00000337008 | ZNF644 | 0.4158 |
| ENSP00000337313 | ZCCHC8 | 0.4158 |
| ENSP00000406955 | ZBTB38 | 0.4158 |
| ENSP00000351539 | ZBTB18 | 0.4158 |
| ENSP00000309031 | VCPIP1 | 0.4158 |
| ENSP00000303434 | USP38 | 0.4158 |
| ENSP00000307701 | TSPAN5 | 0.4158 |
| ENSP00000299427 | TPP1 | 0.4158 |
| ENSP00000312599 | TMEM70 | 0.4158 |
| ENSP00000346065 | TDRD6 | 0.4158 |
| ENSP00000421655 | TCOF1 | 0.4158 |
| ENSP00000358677 | TBX18 | 0.4158 |
| ENSP00000321826 | STXBP5 | 0.4158 |
| ENSP00000270061 | SSBP4 | 0.4158 |
| ENSP00000288680 | SPPL3 | 0.4158 |
| ENSP00000386531 | SMPD4 | 0.4158 |
| ENSP00000294008 | SLX4 | 0.4158 |
| ENSP00000219343 | SLC7A6 | 0.4158 |
| ENSP00000266682 | SLC6A15 | 0.4158 |
| ENSP00000393571 | SLC35F2 | 0.4158 |

| | | |
|---|---|---|
| ENSP00000359526 | SLC25A28 | 0.4158 |
| ENSP00000355930 | SLC22A1 | 0.4158 |
| ENSP00000360549 | SLC1A7 | 0.4158 |
| ENSP00000395983 | SLC12A4 | 0.4158 |
| ENSP00000394049 | SLA | 0.4158 |
| ENSP00000341141 | SIGLEC1 | 0.4158 |
| ENSP00000271375 | SFT2D2 | 0.4158 |
| ENSP00000354590 | SFT2D1 | 0.4158 |
| ENSP00000377655 | SENP7 | 0.4158 |
| ENSP00000345512 | SEMA6A | 0.4158 |
| ENSP00000278947 | SCN2B | 0.4158 |
| ENSP00000304133 | SCG2 | 0.4158 |
| ENSP00000346037 | RPLP1 | 0.4158 |
| ENSP00000371923 | RP1L1 | 0.4158 |
| ENSP00000329454 | RCAN2 | 0.4158 |
| ENSP00000379051 | PURB | 0.4158 |
| ENSP00000373153 | PTPLB | 0.4158 |
| ENSP00000341914 | PTCHD4 | 0.4158 |
| ENSP00000216968 | PROCR | 0.4158 |
| ENSP00000261308 | PPWD1 | 0.4158 |
| ENSP00000414202 | PPP6R1 | 0.4158 |
| ENSP00000257622 | POM121 | 0.4158 |
| ENSP00000283426 | PLEKHG4B | 0.4158 |
| ENSP00000389275 | PKD1P1 | 0.4158 |
| ENSP00000317721 | PIPOX | 0.4158 |
| ENSP00000352185 | PHF2 | 0.4158 |
| ENSP00000295030 | PEX13 | 0.4158 |
| ENSP00000221480 | PEX11G | 0.4158 |
| ENSP00000436376 | OMP | 0.4158 |
| ENSP00000378367 | NRG4 | 0.4158 |
| ENSP00000460475 | NLRP1 | 0.4158 |
| ENSP00000436926 | NFRKB | 0.4158 |
| ENSP00000382390 | NEURL4 | 0.4158 |
| ENSP00000361475 | NCS1 | 0.4158 |
| ENSP00000303920 | MZB1 | 0.4158 |
| ENSP00000302716 | MUC17 | 0.4158 |
| ENSP00000215743 | MMP11 | 0.4158 |
| ENSP00000361895 | MFSD2A | 0.4158 |
| ENSP00000267287 | MBNL2 | 0.4158 |
| ENSP00000399718 | MBIP | 0.4158 |
| ENSP00000261483 | MAN2A1 | 0.4158 |
| ENSP00000265801 | LZTS1 | 0.4158 |
| ENSP00000230568 | LY86 | 0.4158 |
| ENSP00000376910 | LRRIQ1 | 0.4158 |
| ENSP00000278198 | LRRC4C | 0.4158 |
| ENSP00000379880 | LRRC3B | 0.4158 |
| ENSP00000355396 | LRIG2 | 0.4158 |
| ENSP00000317300 | LPCAT4 | 0.4158 |
| ENSP00000307214 | LCMT2 | 0.4158 |
| ENSP00000443191 | KLHL13 | 0.4158 |
| ENSP00000234371 | KISS1R | 0.4158 |
| ENSP00000274629 | KCNMB1 | 0.4158 |
| ENSP00000321427 | KCNH5 | 0.4158 |

| | | |
|---|---|---|
| ENSP00000337255 | KCNE1 | 0.4158 |
| ENSP00000265969 | KCNC1 | 0.4158 |
| ENSP00000386992 | IPO11 | 0.4158 |
| ENSP00000239347 | IFNA7 | 0.4158 |
| ENSP00000259555 | IFNA5 | 0.4158 |
| ENSP00000465194 | IER3IP1 | 0.4158 |
| ENSP00000333300 | HSD17B13 | 0.4158 |
| ENSP00000259241 | HS6ST1 | 0.4158 |
| ENSP00000244601 | HIST1H2BG | 0.4158 |
| ENSP00000351589 | HIST1H2AI | 0.4158 |
| ENSP00000358727 | GSTO1 | 0.4158 |
| ENSP00000282499 | GRIA4 | 0.4158 |
| ENSP00000259803 | GCM1 | 0.4158 |
| ENSP00000394842 | GCA | 0.4158 |
| ENSP00000264318 | GABRA4 | 0.4158 |
| ENSP00000265651 | FBXO3 | 0.4158 |
| ENSP00000359778 | ELTD1 | 0.4158 |
| ENSP00000431162 | EID1 | 0.4158 |
| ENSP00000345281 | DUSP22 | 0.4158 |
| ENSP00000317997 | DUOX1 | 0.4158 |
| ENSP00000248041 | CYP4F11 | 0.4158 |
| ENSP00000334128 | CYP27C1 | 0.4158 |
| ENSP00000418348 | CMC1 | 0.4158 |
| ENSP00000384554 | CLDN5 | 0.4158 |
| ENSP00000308870 | CLDN15 | 0.4158 |
| ENSP00000281141 | CDC123 | 0.4158 |
| ENSP00000405708 | CCDC39 | 0.4158 |
| ENSP00000348915 | CBWD1 | 0.4158 |
| ENSP00000361524 | C9orf78 | 0.4158 |
| ENSP00000340296 | C10orf129 | 0.4158 |
| ENSP00000346697 | BMF | 0.4158 |
| ENSP00000371084 | ATP8A1 | 0.4158 |
| ENSP00000281087 | ATP6V1G3 | 0.4158 |
| ENSP00000252491 | APOC1 | 0.4158 |
| ENSP00000264908 | ANXA3 | 0.4158 |
| ENSP00000346987 | ANAPC13 | 0.4158 |
| ENSP00000307199 | AHSP | 0.4158 |
| ENSP00000387281 | ADRA2B | 0.4158 |
| ENSP00000411658 | ADCYAP1 | 0.4158 |
| ENSP00000419446 | ADAM9 | 0.4158 |
| ENSP00000322376 | U2SURP | 0.4158 |
| ENSP00000352614 | ZSWIM5 | 0.4132 |
| ENSP00000295131 | ZSWIM2 | 0.4132 |
| ENSP00000371905 | ZDHHC20 | 0.4132 |
| ENSP00000243344 | TTC21B | 0.4132 |
| ENSP00000301364 | TSR1 | 0.4132 |
| ENSP00000449795 | TRIAP1 | 0.4132 |
| ENSP00000381567 | TMF1 | 0.4132 |
| ENSP00000376553 | TMEM119 | 0.4132 |
| ENSP00000417050 | TM4SF2 | 0.4132 |
| ENSP00000347538 | SYT17 | 0.4132 |
| ENSP00000242465 | SRGN | 0.4132 |
| ENSP00000353670 | SPOCD1 | 0.4132 |

| | | |
|---|---|---|
| ENSP00000308057 | SNCB | 0.4132 |
| ENSP00000266088 | SLC5A1 | 0.4132 |
| ENSP00000275230 | SLC2A12 | 0.4132 |
| ENSP00000355384 | SLC26A11 | 0.4132 |
| ENSP00000388658 | SLC25A12 | 0.4132 |
| ENSP00000381698 | SHARPIN | 0.4132 |
| ENSP00000348471 | SH3YL1 | 0.4132 |
| ENSP00000331376 | SERHL2 | 0.4132 |
| ENSP00000333956 | SELV | 0.4132 |
| ENSP00000342868 | SCXB | 0.4132 |
| ENSP00000272430 | RTKN | 0.4132 |
| ENSP00000330188 | RSRC2 | 0.4132 |
| ENSP00000449334 | RP11-345J4.3 | 0.4132 |
| ENSP00000446654 | RP11-176H8.1 | 0.4132 |
| ENSP00000270357 | RNPEPL1 | 0.4132 |
| ENSP00000262173 | RNMT | 0.4132 |
| ENSP00000315112 | RNF31 | 0.4132 |
| ENSP00000342667 | RNF19A | 0.4132 |
| ENSP00000306906 | RNF181 | 0.4132 |
| ENSP00000269439 | RNF165 | 0.4132 |
| ENSP00000292363 | RNF126 | 0.4132 |
| ENSP00000350136 | RIMKLB | 0.4132 |
| ENSP00000240285 | RDH10 | 0.4132 |
| ENSP00000356991 | PVRL4 | 0.4132 |
| ENSP00000349541 | PTRF | 0.4132 |
| ENSP00000230582 | PRSS16 | 0.4132 |
| ENSP00000356156 | PPP1R15B | 0.4132 |
| ENSP00000314724 | PPFIBP1 | 0.4132 |
| ENSP00000330442 | PLB1 | 0.4132 |
| ENSP00000217305 | PDYN | 0.4132 |
| ENSP00000218004 | NXT2 | 0.4132 |
| ENSP00000356125 | NUAK2 | 0.4132 |
| ENSP00000358783 | NPBWR2 | 0.4132 |
| ENSP00000360464 | NKAP | 0.4132 |
| ENSP00000298352 | NGB | 0.4132 |
| ENSP00000324628 | NAPG | 0.4132 |
| ENSP00000267023 | NABP2 | 0.4132 |
| ENSP00000264444 | MXD1 | 0.4132 |
| ENSP00000357823 | MRPL9 | 0.4132 |
| ENSP00000258169 | MPHOSPH6 | 0.4132 |
| ENSP00000286614 | MMP16 | 0.4132 |
| ENSP00000312678 | MID1 | 0.4132 |
| ENSP00000296135 | LZTFL1 | 0.4132 |
| ENSP00000340118 | LITAF | 0.4132 |
| ENSP00000365637 | KIAA1217 | 0.4132 |
| ENSP00000352527 | KCNK5 | 0.4132 |
| ENSP00000334650 | KCNK18 | 0.4132 |
| ENSP00000357068 | KCNJ10 | 0.4132 |
| ENSP00000449253 | KCNC2 | 0.4132 |
| ENSP00000379739 | IPP | 0.4132 |
| ENSP00000314976 | IL20RA | 0.4132 |
| ENSP00000387941 | IFFO2 | 0.4132 |
| ENSP00000256433 | IER3IP1 | 0.4132 |

| | | |
|---|---|---|
| ENSP00000384474 | HYPK | 0.4132 |
| ENSP00000407154 | HSD11B1L | 0.4132 |
| ENSP00000387088 | HNRPLL | 0.4132 |
| ENSP00000393566 | HLA-DPA1 | 0.4132 |
| ENSP00000244661 | HIST1H3B | 0.4132 |
| ENSP00000359171 | HIAT1 | 0.4132 |
| ENSP00000014930 | HEBP1 | 0.4132 |
| ENSP00000326579 | HCCS | 0.4132 |
| ENSP00000243706 | HAUS3 | 0.4132 |
| ENSP00000334002 | HAP1 | 0.4132 |
| ENSP00000355155 | GRIN3A | 0.4132 |
| ENSP00000403536 | GAMT | 0.4132 |
| ENSP00000253801 | G6PC | 0.4132 |
| ENSP00000376945 | FBXW9 | 0.4132 |
| ENSP00000292853 | FBXO27 | 0.4132 |
| ENSP00000252338 | FAM155B | 0.4132 |
| ENSP00000261172 | EPYC | 0.4132 |
| ENSP00000381247 | EFCAB9 | 0.4132 |
| ENSP00000250024 | E2F8 | 0.4132 |
| ENSP00000256261 | DUSP26 | 0.4132 |
| ENSP00000408464 | DOCK8 | 0.4132 |
| ENSP00000270172 | DNMT3L | 0.4132 |
| ENSP00000360500 | DMRTB1 | 0.4132 |
| ENSP00000247003 | DDX49 | 0.4132 |
| ENSP00000334625 | DCUN1D4 | 0.4132 |
| ENSP00000308032 | CYP2S1 | 0.4132 |
| ENSP00000333534 | CYP2F1 | 0.4132 |
| ENSP00000469517 | CTB-167G5.5 | 0.4132 |
| ENSP00000225387 | CRYBA1 | 0.4132 |
| ENSP00000321856 | CRELD1 | 0.4132 |
| ENSP00000231948 | CRBN | 0.4132 |
| ENSP00000222481 | CPA2 | 0.4132 |
| ENSP00000011292 | CPA1 | 0.4132 |
| ENSP00000287916 | CLDN12 | 0.4132 |
| ENSP00000320209 | CIDEA | 0.4132 |
| ENSP00000264993 | CDV3 | 0.4132 |
| ENSP00000350256 | CCR9 | 0.4132 |
| ENSP00000225844 | CCL13 | 0.4132 |
| ENSP00000312706 | CCDC65 | 0.4132 |
| ENSP00000385247 | CAPN14 | 0.4132 |
| ENSP00000333919 | BTLA | 0.4132 |
| ENSP00000349351 | BICD2 | 0.4132 |
| ENSP00000365131 | BAG6 | 0.4132 |
| ENSP00000421172 | AWAT2 | 0.4132 |
| ENSP00000302194 | ATP6V1G2 | 0.4132 |
| ENSP00000225737 | AKAP10 | 0.4132 |
| ENSP00000373640 | AGPHD1 | 0.4132 |
| ENSP00000370713 | ADARB2 | 0.4132 |
| ENSP00000355777 | ACBD3 | 0.4132 |
| ENSP00000471024 | AC093917.1 | 0.4132 |
| ENSP00000299989 | CATSPER2 | 0.4132 |
| ENSP00000310042 | ZNF622 | 0.4105 |
| ENSP00000379363 | ZNF501 | 0.4105 |

| | | |
|---|---|---|
| ENSP00000251119 | ZFYVE26 | 0.4105 |
| ENSP00000289528 | ZFAND2B | 0.4105 |
| ENSP00000417677 | ZBTB8OS | 0.4105 |
| ENSP00000329803 | ZAR1 | 0.4105 |
| ENSP00000342832 | YPEL1 | 0.4105 |
| ENSP00000362549 | UGT1A8 | 0.4105 |
| ENSP00000362508 | UGT1A4 | 0.4105 |
| ENSP00000359904 | TYW3 | 0.4105 |
| ENSP00000352358 | TRPV6 | 0.4105 |
| ENSP00000343709 | SNX10 | 0.4105 |
| ENSP00000285850 | SLC7A7 | 0.4105 |
| ENSP00000243389 | SLC36A1 | 0.4105 |
| ENSP00000387694 | SLC12A5 | 0.4105 |
| ENSP00000313079 | SHISA2 | 0.4105 |
| ENSP00000327436 | SETD3 | 0.4105 |
| ENSP00000379704 | SEPT3 | 0.4105 |
| ENSP00000361855 | SEMG2 | 0.4105 |
| ENSP00000337688 | SEC62 | 0.4105 |
| ENSP00000349486 | SCRIB | 0.4105 |
| ENSP00000301904 | SCARA3 | 0.4105 |
| ENSP00000297109 | SAP30L | 0.4105 |
| ENSP00000430237 | RNF130 | 0.4105 |
| ENSP00000267291 | RNF113B | 0.4105 |
| ENSP00000324127 | PSD3 | 0.4105 |
| ENSP00000360035 | PPP1R3D | 0.4105 |
| ENSP00000260264 | POU2F3 | 0.4105 |
| ENSP00000442736 | PLXNB3 | 0.4105 |
| ENSP00000026218 | PIGQ | 0.4105 |
| ENSP00000225538 | P2RX1 | 0.4105 |
| ENSP00000367930 | OXER1 | 0.4105 |
| ENSP00000371155 | NUPL1 | 0.4105 |
| ENSP00000246912 | MLX | 0.4105 |
| ENSP00000345270 | MGAT3 | 0.4105 |
| ENSP00000373300 | METTL6 | 0.4105 |
| ENSP00000374443 | LYST | 0.4105 |
| ENSP00000359609 | LMBRD1 | 0.4105 |
| ENSP00000326888 | LIMS2 | 0.4105 |
| ENSP00000431443 | KRTAP5-9 | 0.4105 |
| ENSP00000287777 | KLHL40 | 0.4105 |
| ENSP00000310670 | KIAA1967 | 0.4105 |
| ENSP00000297591 | KIAA1429 | 0.4105 |
| ENSP00000352035 | KCNQ2 | 0.4105 |
| ENSP00000315654 | KCNG2 | 0.4105 |
| ENSP00000337432 | IL17F | 0.4105 |
| ENSP00000369558 | IFNA6 | 0.4105 |
| ENSP00000194214 | HSPB11 | 0.4105 |
| ENSP00000383516 | HMX1 | 0.4105 |
| ENSP00000274341 | HAPLN1 | 0.4105 |
| ENSP00000362183 | GRIK3 | 0.4105 |
| ENSP00000309493 | GPRC6A | 0.4105 |
| ENSP00000365806 | GNL1 | 0.4105 |
| ENSP00000359385 | GLMN | 0.4105 |
| ENSP00000205061 | GLG1 | 0.4105 |

| | | |
|---|---|---|
| ENSP00000248272 | GAN | 0.4105 |
| ENSP00000376570 | GALNT13 | 0.4105 |
| ENSP00000278882 | FRG1B | 0.4105 |
| ENSP00000300784 | FN3K | 0.4105 |
| ENSP00000248076 | F2RL3 | 0.4105 |
| ENSP00000260585 | EPT1 | 0.4105 |
| ENSP00000358045 | ECM1 | 0.4105 |
| ENSP00000356791 | DPT | 0.4105 |
| ENSP00000334113 | DHRSX | 0.4105 |
| ENSP00000334246 | CYP4Z1 | 0.4105 |
| ENSP00000261835 | CYP46A1 | 0.4105 |
| ENSP00000359507 | CUTC | 0.4105 |
| ENSP00000358511 | CNR1 | 0.4105 |
| ENSP00000384264 | CNGA1 | 0.4105 |
| ENSP00000316228 | CLEC4M | 0.4105 |
| ENSP00000353500 | CLEC4C | 0.4105 |
| ENSP00000357683 | CHTOP | 0.4105 |
| ENSP00000302629 | CHSY3 | 0.4105 |
| ENSP00000375086 | CCL25 | 0.4105 |
| ENSP00000293280 | CCL23 | 0.4105 |
| ENSP00000219244 | CCL17 | 0.4105 |
| ENSP00000436691 | CARD18 | 0.4105 |
| ENSP00000244513 | BTN1A1 | 0.4105 |
| ENSP00000399709 | BEST1 | 0.4105 |
| ENSP00000261499 | B9D1 | 0.4105 |
| ENSP00000360475 | ARRDC1 | 0.4105 |
| ENSP00000466775 | APOC2 | 0.4105 |
| ENSP00000301030 | ANKRD11 | 0.4105 |
| ENSP00000344241 | ZMAT5 | 0.4079 |
| ENSP00000364840 | VWA7 | 0.4079 |
| ENSP00000366565 | VPS28 | 0.4079 |
| ENSP00000387683 | UGT2B11 | 0.4079 |
| ENSP00000358283 | UFL1 | 0.4079 |
| ENSP00000345294 | TTLL13 | 0.4079 |
| ENSP00000265310 | TRPV5 | 0.4079 |
| ENSP00000362205 | TREM2 | 0.4079 |
| ENSP00000356599 | TOR3A | 0.4079 |
| ENSP00000284856 | TMSB4Y | 0.4079 |
| ENSP00000299596 | TMEM41B | 0.4079 |
| ENSP00000061240 | TLL1 | 0.4079 |
| ENSP00000340995 | TEX11 | 0.4079 |
| ENSP00000293970 | TBC1D24 | 0.4079 |
| ENSP00000309794 | TBC1D16 | 0.4079 |
| ENSP00000303325 | TACR3 | 0.4079 |
| ENSP00000293897 | SSTR5 | 0.4079 |
| ENSP00000344967 | SPRY4 | 0.4079 |
| ENSP00000348753 | SPRED2 | 0.4079 |
| ENSP00000335628 | SPDYA | 0.4079 |
| ENSP00000319597 | SNAPC5 | 0.4079 |
| ENSP00000425133 | SMG7 | 0.4079 |
| ENSP00000319574 | SLC25A19 | 0.4079 |
| ENSP00000265870 | SLC25A16 | 0.4079 |
| ENSP00000262352 | SLC1A1 | 0.4079 |

| | | |
|---|---|---|
| ENSP00000380250 | SLC17A3 | 0.4079 |
| ENSP00000358541 | SIKE1 | 0.4079 |
| ENSP00000291842 | SHKBP1 | 0.4079 |
| ENSP00000373301 | SH3BP5 | 0.4079 |
| ENSP00000350789 | SFMBT1 | 0.4079 |
| ENSP00000264896 | SCARB2 | 0.4079 |
| ENSP00000406738 | SARM1 | 0.4079 |
| ENSP00000349955 | RPRD1A | 0.4079 |
| ENSP00000345156 | RPL14 | 0.4079 |
| ENSP00000426906 | RP11-514O12.4 | 0.4079 |
| ENSP00000283632 | RMND5A | 0.4079 |
| ENSP00000361768 | RIMS3 | 0.4079 |
| ENSP00000401536 | RFX8 | 0.4079 |
| ENSP00000357442 | PYGO2 | 0.4079 |
| ENSP00000267568 | PTGR2 | 0.4079 |
| ENSP00000430548 | PTDSS1 | 0.4079 |
| ENSP00000337194 | PRPF4B | 0.4079 |
| ENSP00000269844 | PRDM15 | 0.4079 |
| ENSP00000323302 | POC1B | 0.4079 |
| ENSP00000347883 | PLEKHA7 | 0.4079 |
| ENSP00000348901 | PLD3 | 0.4079 |
| ENSP00000309430 | PIGS | 0.4079 |
| ENSP00000308361 | P2RY14 | 0.4079 |
| ENSP00000429064 | MTSS1 | 0.4079 |
| ENSP00000359518 | MRGBP | 0.4079 |
| ENSP00000225275 | MPO | 0.4079 |
| ENSP00000318077 | MC5R | 0.4079 |
| ENSP00000368798 | MBTPS2 | 0.4079 |
| ENSP00000359890 | MBNL3 | 0.4079 |
| ENSP00000345917 | LYAR | 0.4079 |
| ENSP00000274711 | LRRTM2 | 0.4079 |
| ENSP00000366950 | KRTAP9-2 | 0.4079 |
| ENSP00000366629 | KM-PA-2 | 0.4079 |
| ENSP00000336800 | KLHL25 | 0.4079 |
| ENSP00000257408 | KLB | 0.4079 |
| ENSP00000307694 | KCNS1 | 0.4079 |
| ENSP00000262888 | KCNN4 | 0.4079 |
| ENSP00000304127 | KCNG3 | 0.4079 |
| ENSP00000362935 | ITGB1BP2 | 0.4079 |
| ENSP00000414237 | INTS2 | 0.4079 |
| ENSP00000301464 | IGFBP6 | 0.4079 |
| ENSP00000333277 | HIST2H3D | 0.4079 |
| ENSP00000264357 | GRIA3 | 0.4079 |
| ENSP00000376384 | GPR123 | 0.4079 |
| ENSP00000280187 | GPM6A | 0.4079 |
| ENSP00000261858 | GLCE | 0.4079 |
| ENSP00000295759 | GLB1L | 0.4079 |
| ENSP00000363603 | FUCA1 | 0.4079 |
| ENSP00000285071 | FLCN | 0.4079 |
| ENSP00000316491 | FCAMR | 0.4079 |
| ENSP00000292147 | ETHE1 | 0.4079 |
| ENSP00000261735 | ERP29 | 0.4079 |
| ENSP00000359022 | ELOVL3 | 0.4079 |

| | | |
|---|---|---|
| ENSP00000345853 | DUSP9 | 0.4079 |
| ENSP00000337572 | DGKH | 0.4079 |
| ENSP00000360652 | CXorf56 | 0.4079 |
| ENSP00000284878 | CXADR | 0.4079 |
| ENSP00000365025 | CSNK2B | 0.4079 |
| ENSP00000341030 | CSN2 | 0.4079 |
| ENSP00000460885 | CORO7-PAM16 | 0.4079 |
| ENSP00000262507 | COQ9 | 0.4079 |
| ENSP00000305913 | COL8A2 | 0.4079 |
| ENSP00000382083 | COL25A1 | 0.4079 |
| ENSP00000244728 | COL21A1 | 0.4079 |
| ENSP00000305442 | COG7 | 0.4079 |
| ENSP00000336587 | CDR2L | 0.4079 |
| ENSP00000370503 | CCM2 | 0.4079 |
| ENSP00000404220 | CCDC155 | 0.4079 |
| ENSP00000378690 | CALN1 | 0.4079 |
| ENSP00000419081 | CALML4 | 0.4079 |
| ENSP00000216144 | CABP7 | 0.4079 |
| ENSP00000263408 | C9 | 0.4079 |
| ENSP00000313046 | BAI1 | 0.4079 |
| ENSP00000311336 | ATP8B3 | 0.4079 |
| ENSP00000455561 | ATP6C | 0.4079 |
| ENSP00000334216 | ATP4B | 0.4079 |
| ENSP00000387435 | ARTN | 0.4079 |
| ENSP00000348828 | ARHGEF26 | 0.4079 |
| ENSP00000307004 | APLF | 0.4079 |
| ENSP00000246041 | AP5S1 | 0.4079 |
| ENSP00000265447 | ANXA11 | 0.4079 |
| ENSP00000369434 | ANKRD16 | 0.4079 |
| ENSP00000388275 | AGBL3 | 0.4079 |
| ENSP00000369099 | ADPRM | 0.4079 |
| ENSP00000262198 | ADNP2 | 0.4079 |
| ENSP00000373472 | ADAMTS7 | 0.4079 |
| ENSP00000370443 | ADAMTS6 | 0.4079 |
| ENSP00000323071 | ACOT4 | 0.4079 |
| ENSP00000269080 | ABCA8 | 0.4079 |
| ENSP00000232892 | AADAC | 0.4079 |
| ENSP00000442633 | NPIPL3 | 0.4079 |
| ENSP00000358590 | ZNF292 | 0.4053 |
| ENSP00000292579 | ZNF250 | 0.4053 |
| ENSP00000362918 | YTHDF2 | 0.4053 |
| ENSP00000305906 | UTF1 | 0.4053 |
| ENSP00000431876 | USP35 | 0.4053 |
| ENSP00000354039 | UBAP2 | 0.4053 |
| ENSP00000320239 | TRPM7 | 0.4053 |
| ENSP00000343129 | TRIM29 | 0.4053 |
| ENSP00000343505 | TNFSF13 | 0.4053 |
| ENSP00000275780 | TLK2 | 0.4053 |
| ENSP00000370741 | THUMPD1 | 0.4053 |
| ENSP00000343199 | TC2N | 0.4053 |
| ENSP00000364520 | TAS1R2 | 0.4053 |
| ENSP00000404833 | TAPBP | 0.4053 |
| ENSP00000384917 | SVEP1 | 0.4053 |

| | | |
|---|---|---|
| ENSP00000227135 | SPA17 | 0.4053 |
| ENSP00000222584 | SP4 | 0.4053 |
| ENSP00000253188 | SLC7A10 | 0.4053 |
| ENSP00000333433 | SKA2 | 0.4053 |
| ENSP00000450832 | SIPA1L1 | 0.4053 |
| ENSP00000350018 | SH3BP1 | 0.4053 |
| ENSP00000329189 | SETD4 | 0.4053 |
| ENSP00000380824 | SEPT10 | 0.4053 |
| ENSP00000229554 | RSPH4A | 0.4053 |
| ENSP00000311513 | RSF1 | 0.4053 |
| ENSP00000454445 | RP11-463D19.2 | 0.4053 |
| ENSP00000258646 | RCBTB1 | 0.4053 |
| ENSP00000232217 | RBP2 | 0.4053 |
| ENSP00000368684 | RASSF2 | 0.4053 |
| ENSP00000202677 | RALGAPA2 | 0.4053 |
| ENSP00000329915 | PSMG1 | 0.4053 |
| ENSP00000268124 | POLG | 0.4053 |
| ENSP00000311368 | POLD4 | 0.4053 |
| ENSP00000351318 | PLEKHG1 | 0.4053 |
| ENSP00000307822 | NPFFR2 | 0.4053 |
| ENSP00000360660 | NPDC1 | 0.4053 |
| ENSP00000345464 | NHLRC1 | 0.4053 |
| ENSP00000330694 | NDNL2 | 0.4053 |
| ENSP00000442464 | NCEH1 | 0.4053 |
| ENSP00000359423 | MTM1 | 0.4053 |
| ENSP00000365693 | MSRB2 | 0.4053 |
| ENSP00000339086 | MKL2 | 0.4053 |
| ENSP00000340900 | MIA3 | 0.4053 |
| ENSP00000260953 | METTL5 | 0.4053 |
| ENSP00000354341 | MEPE | 0.4053 |
| ENSP00000333181 | MARCH11 | 0.4053 |
| ENSP00000265245 | LSG1 | 0.4053 |
| ENSP00000295057 | LRRTM1 | 0.4053 |
| ENSP00000434849 | LRRC24 | 0.4053 |
| ENSP00000296388 | LEPRE1 | 0.4053 |
| ENSP00000358861 | LCA5 | 0.4053 |
| ENSP00000358786 | KCNA10 | 0.4053 |
| ENSP00000369402 | IFT74 | 0.4053 |
| ENSP00000369574 | IFNA21 | 0.4053 |
| ENSP00000307766 | HTR1E | 0.4053 |
| ENSP00000252825 | HRC | 0.4053 |
| ENSP00000244534 | HIST1H1D | 0.4053 |
| ENSP00000375066 | HCAR2 | 0.4053 |
| ENSP00000339835 | H3F3C | 0.4053 |
| ENSP00000373176 | GTPBP8 | 0.4053 |
| ENSP00000434045 | GPR68 | 0.4053 |
| ENSP00000362353 | GLP1R | 0.4053 |
| ENSP00000336832 | GJD3 | 0.4053 |
| ENSP00000376465 | GALNT3 | 0.4053 |
| ENSP00000321320 | FAM203A | 0.4053 |
| ENSP00000233379 | FAHD2A | 0.4053 |
| ENSP00000367446 | EXT1 | 0.4053 |
| ENSP00000242108 | EEPD1 | 0.4053 |

| | | |
|---|---|---|
| ENSP00000242317 | DNAI1 | 0.4053 |
| ENSP00000318227 | DCAF8 | 0.4053 |
| ENSP00000300452 | COQ4 | 0.4053 |
| ENSP00000330060 | CNOT10 | 0.4053 |
| ENSP00000263097 | CNN2 | 0.4053 |
| ENSP00000328182 | CLN8 | 0.4053 |
| ENSP00000324491 | CHMP7 | 0.4053 |
| ENSP00000457031 | CHKB-CPT1B | 0.4053 |
| ENSP00000291577 | C21orf33 | 0.4053 |
| ENSP00000401770 | C14orf2 | 0.4053 |
| ENSP00000262865 | BPI | 0.4053 |
| ENSP00000357591 | BET3L | 0.4053 |
| ENSP00000364092 | ASIP | 0.4053 |
| ENSP00000227665 | APOA5 | 0.4053 |
| ENSP00000346550 | ANXA6 | 0.4053 |
| ENSP00000358730 | ADORA3 | 0.4053 |
| ENSP00000418735 | ADAMTS9 | 0.4053 |
| ENSP00000265708 | ADAM2 | 0.4053 |
| ENSP00000310309 | ABLIM3 | 0.4053 |
| ENSP00000263094 | ABCA7 | 0.4053 |
| ENSP00000344155 | ABCA2 | 0.4053 |
| ENSP00000433773 | ZNF585B | 0.4026 |
| ENSP00000287218 | ZFAND3 | 0.4026 |
| ENSP00000306864 | VASN | 0.4026 |
| ENSP00000239878 | UFM1 | 0.4026 |
| ENSP00000361990 | UBR2 | 0.4026 |
| ENSP00000355613 | TRIM67 | 0.4026 |
| ENSP00000438204 | TOMM5 | 0.4026 |
| ENSP00000381104 | TM9SF4 | 0.4026 |
| ENSP00000256246 | TEX15 | 0.4026 |
| ENSP00000417569 | STIM2 | 0.4026 |
| ENSP00000234454 | SPR | 0.4026 |
| ENSP00000352175 | SNX11 | 0.4026 |
| ENSP00000341550 | SLC24A5 | 0.4026 |
| ENSP00000383256 | SIAH3 | 0.4026 |
| ENSP00000351981 | SGMS2 | 0.4026 |
| ENSP00000337212 | SERPINB7 | 0.4026 |
| ENSP00000335024 | SERPINA11 | 0.4026 |
| ENSP00000314173 | SAMD12 | 0.4026 |
| ENSP00000379090 | PPP6R2 | 0.4026 |
| ENSP00000305924 | PPP4R4 | 0.4026 |
| ENSP00000384004 | POU6F2 | 0.4026 |
| ENSP00000309509 | PLAC8 | 0.4026 |
| ENSP00000243501 | PLA2G12A | 0.4026 |
| ENSP00000360158 | PKHD1 | 0.4026 |
| ENSP00000385795 | PHF14 | 0.4026 |
| ENSP00000429301 | PGRMC2 | 0.4026 |
| ENSP00000278060 | PAOX | 0.4026 |
| ENSP00000302768 | PAIP1 | 0.4026 |
| ENSP00000376293 | OSBPL6 | 0.4026 |
| ENSP00000341987 | OR51I2 | 0.4026 |
| ENSP00000293973 | NTN3 | 0.4026 |
| ENSP00000330284 | NPBWR1 | 0.4026 |

| | | |
|---|---|---|
| ENSP00000328854 | NOC4L | 0.4026 |
| ENSP00000350364 | NEGR1 | 0.4026 |
| ENSP00000340093 | NAPEPLD | 0.4026 |
| ENSP00000369682 | MRPS26 | 0.4026 |
| ENSP00000354335 | MPEG1 | 0.4026 |
| ENSP00000369860 | MOSPD2 | 0.4026 |
| ENSP00000367668 | MMEL1 | 0.4026 |
| ENSP00000281317 | MMAA | 0.4026 |
| ENSP00000310375 | MLC1 | 0.4026 |
| ENSP00000363079 | MBL2 | 0.4026 |
| ENSP00000266643 | MARCH9 | 0.4026 |
| ENSP00000274056 | MARCH1 | 0.4026 |
| ENSP00000357453 | MAN1A1 | 0.4026 |
| ENSP00000433433 | MAGEL2 | 0.4026 |
| ENSP00000324701 | MAB21L2 | 0.4026 |
| ENSP00000292616 | LRWD1 | 0.4026 |
| ENSP00000361177 | LRIT1 | 0.4026 |
| ENSP00000347451 | LINGO1 | 0.4026 |
| ENSP00000295682 | KRTCAP2 | 0.4026 |
| ENSP00000246662 | KRT9 | 0.4026 |
| ENSP00000362206 | KLHL4 | 0.4026 |
| ENSP00000328062 | KIAA1033 | 0.4026 |
| ENSP00000371514 | KCNV2 | 0.4026 |
| ENSP00000287042 | KCNS2 | 0.4026 |
| ENSP00000218176 | KCND1 | 0.4026 |
| ENSP00000388723 | KBTBD13 | 0.4026 |
| ENSP00000343000 | IL19 | 0.4026 |
| ENSP00000359787 | IFI44L | 0.4026 |
| ENSP00000287907 | HTR5A | 0.4026 |
| ENSP00000336984 | HORMAD2 | 0.4026 |
| ENSP00000244537 | HIST1H4G | 0.4026 |
| ENSP00000353074 | HIST1H2BF | 0.4026 |
| ENSP00000310621 | HHATL | 0.4026 |
| ENSP00000367196 | GLIPR2 | 0.4026 |
| ENSP00000345868 | GJB4 | 0.4026 |
| ENSP00000319254 | GIPC3 | 0.4026 |
| ENSP00000233838 | GGCX | 0.4026 |
| ENSP00000379895 | GATM | 0.4026 |
| ENSP00000259455 | GABBR2 | 0.4026 |
| ENSP00000274457 | FEM1C | 0.4026 |
| ENSP00000292852 | FBXO17 | 0.4026 |
| ENSP00000393154 | FBXO15 | 0.4026 |
| ENSP00000369857 | FAM175A | 0.4026 |
| ENSP00000332111 | EFCAB13 | 0.4026 |
| ENSP00000384223 | ECE2 | 0.4026 |
| ENSP00000337759 | DOM3Z | 0.4026 |
| ENSP00000383737 | DNAH14 | 0.4026 |
| ENSP00000298292 | DNAAF2 | 0.4026 |
| ENSP00000355114 | DCAF12 | 0.4026 |
| ENSP00000310149 | CYP2W1 | 0.4026 |
| ENSP00000304414 | CXCR6 | 0.4026 |
| ENSP00000278816 | CSRP2BP | 0.4026 |
| ENSP00000305964 | COX7B2 | 0.4026 |

| | | |
|---|---|---|
| ENSP00000370984 | COMMD8 | 0.4026 |
| ENSP00000316030 | COL19A1 | 0.4026 |
| ENSP00000216361 | COCH | 0.4026 |
| ENSP00000378130 | CFI | 0.4026 |
| ENSP00000428864 | CES5A | 0.4026 |
| ENSP00000306968 | CDCA7 | 0.4026 |
| ENSP00000332659 | CCR4 | 0.4026 |
| ENSP00000353295 | CBWD3 | 0.4026 |
| ENSP00000290039 | CACHD1 | 0.4026 |
| ENSP00000327541 | BLZF1 | 0.4026 |
| ENSP00000258168 | BCMO1 | 0.4026 |
| ENSP00000363645 | AWAT1 | 0.4026 |
| ENSP00000432412 | ATG13 | 0.4026 |
| ENSP00000425658 | ANKRD50 | 0.4026 |
| ENSP00000335147 | ANKRD37 | 0.4026 |
| ENSP00000382379 | ANKRD28 | 0.4026 |
| ENSP00000306163 | ANKRD2 | 0.4026 |
| ENSP00000265742 | ANKIB1 | 0.4026 |
| ENSP00000353920 | ADARB1 | 0.4026 |
| ENSP00000263776 | ACCS | 0.4026 |
| ENSP00000431116 | AC133919.6 | 0.4026 |
| ENSP00000226840 | AADAT | 0.4026 |
| ENSP00000303696 | ZNF57 | 0.4000 |
| ENSP00000216923 | ZFP64 | 0.4000 |
| ENSP00000252512 | XPO7 | 0.4000 |
| ENSP00000351141 | WTAP | 0.4000 |
| ENSP00000386156 | WIBG | 0.4000 |
| ENSP00000272930 | UBE2F | 0.4000 |
| ENSP00000305524 | TSEN34 | 0.4000 |
| ENSP00000354006 | TRPM6 | 0.4000 |
| ENSP00000330774 | TMEM106A | 0.4000 |
| ENSP00000350718 | TMCC2 | 0.4000 |
| ENSP00000273037 | TAMM41 | 0.4000 |
| ENSP00000243918 | SYS1 | 0.4000 |
| ENSP00000348496 | SUN5 | 0.4000 |
| ENSP00000346599 | STYX | 0.4000 |
| ENSP00000428338 | SPSB2 | 0.4000 |
| ENSP00000295872 | SPICE1 | 0.4000 |
| ENSP00000309831 | SNUPN | 0.4000 |
| ENSP00000369446 | SMAGP | 0.4000 |
| ENSP00000253122 | SLC6A8 | 0.4000 |
| ENSP00000363852 | SLC44A1 | 0.4000 |
| ENSP00000360773 | SLC29A1 | 0.4000 |
| ENSP00000362159 | SLC25A25 | 0.4000 |
| ENSP00000275730 | SLC12A9 | 0.4000 |
| ENSP00000329213 | SIVA1 | 0.4000 |
| ENSP00000325827 | SEC16A | 0.4000 |
| ENSP00000271638 | S100A11 | 0.4000 |
| ENSP00000364235 | RNF5 | 0.4000 |
| ENSP00000317039 | RMI1 | 0.4000 |
| ENSP00000171214 | RDH8 | 0.4000 |
| ENSP00000228843 | RAD51AP1 | 0.4000 |
| ENSP00000310088 | PTMS | 0.4000 |

| | | |
|---|---|---|
| ENSP00000328178 | PPP1R2 | 0.4000 |
| ENSP00000323927 | PLXDC1 | 0.4000 |
| ENSP00000361337 | PLAC9 | 0.4000 |
| ENSP00000364257 | PLA2G2E | 0.4000 |
| ENSP00000355014 | PJA1 | 0.4000 |
| ENSP00000330031 | PIWIL3 | 0.4000 |
| ENSP00000262265 | PIH1D1 | 0.4000 |
| ENSP00000267460 | PELI2 | 0.4000 |
| ENSP00000277508 | PAEP | 0.4000 |
| ENSP00000343339 | P2RX2 | 0.4000 |
| ENSP00000309673 | OR52W1 | 0.4000 |
| ENSP00000219022 | OLFM4 | 0.4000 |
| ENSP00000287706 | NTAN1 | 0.4000 |
| ENSP00000301295 | NLRP4 | 0.4000 |
| ENSP00000313088 | NETO1 | 0.4000 |
| ENSP00000368716 | NAA16 | 0.4000 |
| ENSP00000311500 | MZT2A | 0.4000 |
| ENSP00000259873 | MRPS18B | 0.4000 |
| ENSP00000339881 | MIOS | 0.4000 |
| ENSP00000354850 | MGEA5 | 0.4000 |
| ENSP00000262432 | METTL2B | 0.4000 |
| ENSP00000364847 | MAGED1 | 0.4000 |
| ENSP00000261978 | LTBP2 | 0.4000 |
| ENSP00000403495 | LTA | 0.4000 |
| ENSP00000293406 | LSM12 | 0.4000 |
| ENSP00000326128 | LARP4B | 0.4000 |
| ENSP00000397598 | KY | 0.4000 |
| ENSP00000310861 | KRT2 | 0.4000 |
| ENSP00000344668 | KRIT1 | 0.4000 |
| ENSP00000279544 | KLRF1 | 0.4000 |
| ENSP00000250916 | KLF16 | 0.4000 |
| ENSP00000307265 | IRF2BP1 | 0.4000 |
| ENSP00000325423 | INPP1 | 0.4000 |
| ENSP00000376410 | INF2 | 0.4000 |
| ENSP00000384432 | ICOSLG | 0.4000 |
| ENSP00000445831 | HIST2H2BF | 0.4000 |
| ENSP00000444823 | HIST1H3F | 0.4000 |
| ENSP00000261917 | HCN4 | 0.4000 |
| ENSP00000222304 | HAMP | 0.4000 |
| ENSP00000397026 | GRIK2 | 0.4000 |
| ENSP00000375893 | GPR55 | 0.4000 |
| ENSP00000262441 | GLP2R | 0.4000 |
| ENSP00000266069 | GID8 | 0.4000 |
| ENSP00000415635 | GGTLC3 | 0.4000 |
| ENSP00000278765 | GGTLC1 | 0.4000 |
| ENSP00000275428 | GGCT | 0.4000 |
| ENSP00000299727 | GALR1 | 0.4000 |
| ENSP00000323065 | GADD45GIP1 | 0.4000 |
| ENSP00000341821 | FPR3 | 0.4000 |
| ENSP00000229769 | FANCE | 0.4000 |
| ENSP00000443411 | FAM92B | 0.4000 |
| ENSP00000366623 | FAM203B | 0.4000 |
| ENSP00000373808 | FAM169A | 0.4000 |

| | | |
|---|---|---|
| ENSP00000300215 | EPB42 | 0.4000 |
| ENSP00000220853 | EMC2 | 0.4000 |
| ENSP00000307843 | EGFL7 | 0.4000 |
| ENSP00000343244 | DUSP21 | 0.4000 |
| ENSP00000344277 | DOK4 | 0.4000 |
| ENSP00000470082 | DEDD2 | 0.4000 |
| ENSP00000321573 | DCBLD2 | 0.4000 |
| ENSP00000341351 | DCAF5 | 0.4000 |
| ENSP00000246012 | CST8 | 0.4000 |
| ENSP00000413575 | CROT | 0.4000 |
| ENSP00000394770 | CMYA5 | 0.4000 |
| ENSP00000454253 | CLN3 | 0.4000 |
| ENSP00000339292 | CLDN14 | 0.4000 |
| ENSP00000259216 | CFC1 | 0.4000 |
| ENSP00000313875 | CD46 | 0.4000 |
| ENSP00000357152 | CD1C | 0.4000 |
| ENSP00000219235 | CCL22 | 0.4000 |
| ENSP00000300900 | CA4 | 0.4000 |
| ENSP00000298295 | C10orf10 | 0.4000 |
| ENSP00000231668 | BNIP1 | 0.4000 |
| ENSP00000256496 | ARL8B | 0.4000 |
| ENSP00000468354 | ANKRD29 | 0.4000 |
| ENSP00000270593 | ACPT | 0.4000 |
| ENSP00000360366 | ACOT11 | 0.4000 |
| ENSP00000354193 | ABP1 | 0.4000 |
| ENSP00000261332 | ZNF24 | 0.3974 |
| ENSP00000362465 | ZDHHC15 | 0.3974 |
| ENSP00000337839 | ZCCHC7 | 0.3974 |
| ENSP00000381821 | YPEL3 | 0.3974 |
| ENSP00000207870 | XYLB | 0.3974 |
| ENSP00000310590 | USP36 | 0.3974 |
| ENSP00000412922 | URM1 | 0.3974 |
| ENSP00000356308 | ULBP3 | 0.3974 |
| ENSP00000384312 | TRIOBP | 0.3974 |
| ENSP00000397073 | TRIM10 | 0.3974 |
| ENSP00000356584 | TOR1AIP2 | 0.3974 |
| ENSP00000457601 | TMSB15B | 0.3974 |
| ENSP00000282382 | TMED7-TICAM2 | 0.3974 |
| ENSP00000334294 | TCFL5 | 0.3974 |
| ENSP00000344411 | TAS1R3 | 0.3974 |
| ENSP00000361998 | TAF7L | 0.3974 |
| ENSP00000314971 | TAF1D | 0.3974 |
| ENSP00000324419 | SYT9 | 0.3974 |
| ENSP00000011691 | SS18L2 | 0.3974 |
| ENSP00000298130 | SPTSSA | 0.3974 |
| ENSP00000289431 | SPATA2 | 0.3974 |
| ENSP00000304930 | SOSTDC1 | 0.3974 |
| ENSP00000336543 | SNURF | 0.3974 |
| ENSP00000221573 | SNAPC2 | 0.3974 |
| ENSP00000405600 | SLC4A2 | 0.3974 |
| ENSP00000354721 | SLC26A1 | 0.3974 |
| ENSP00000253063 | SESN2 | 0.3974 |
| ENSP00000356437 | SASH1 | 0.3974 |

| | | |
|---|---|---|
| ENSP00000259030 | RTP4 | 0.3974 |
| ENSP00000451484 | RP5-1021I20.4 | 0.3974 |
| ENSP00000382101 | RP11-166B2.1 | 0.3974 |
| ENSP00000454746 | RP11-1035H13.3 | 0.3974 |
| ENSP00000338293 | ROMO1 | 0.3974 |
| ENSP00000295640 | RNPEP | 0.3974 |
| ENSP00000237455 | RNF103 | 0.3974 |
| ENSP00000356529 | RGS16 | 0.3974 |
| ENSP00000417291 | REPIN1 | 0.3974 |
| ENSP00000377801 | RCCD1 | 0.3974 |
| ENSP00000349783 | RC3H2 | 0.3974 |
| ENSP00000226105 | RANGRF | 0.3974 |
| ENSP00000344829 | QPCT | 0.3974 |
| ENSP00000252804 | PXDN | 0.3974 |
| ENSP00000359793 | PTGFR | 0.3974 |
| ENSP00000261534 | POMT2 | 0.3974 |
| ENSP00000376372 | PLD4 | 0.3974 |
| ENSP00000409637 | PLCL2 | 0.3974 |
| ENSP00000367096 | PCDH9 | 0.3974 |
| ENSP00000249389 | OPN1SW | 0.3974 |
| ENSP00000379401 | ODAM | 0.3974 |
| ENSP00000463013 | OAZ2 | 0.3974 |
| ENSP00000277942 | NPFFR1 | 0.3974 |
| ENSP00000315674 | NOP14 | 0.3974 |
| ENSP00000349313 | NHEJ1 | 0.3974 |
| ENSP00000307449 | NECAB2 | 0.3974 |
| ENSP00000330787 | NDUFB1 | 0.3974 |
| ENSP00000372313 | MSLN | 0.3974 |
| ENSP00000338057 | MRPL30 | 0.3974 |
| ENSP00000317271 | MCFD2 | 0.3974 |
| ENSP00000369395 | LRRC19 | 0.3974 |
| ENSP00000369252 | LRIT3 | 0.3974 |
| ENSP00000357288 | LAMTOR2 | 0.3974 |
| ENSP00000278671 | LAMTOR1 | 0.3974 |
| ENSP00000379434 | KLHL28 | 0.3974 |
| ENSP00000368464 | KIF24 | 0.3974 |
| ENSP00000313885 | KIAA0195 | 0.3974 |
| ENSP00000430338 | INTS8 | 0.3974 |
| ENSP00000357583 | INPP5A | 0.3974 |
| ENSP00000329312 | IGLL1 | 0.3974 |
| ENSP00000264020 | IFT46 | 0.3974 |
| ENSP00000333639 | IFNL2 | 0.3974 |
| ENSP00000276943 | IFNK | 0.3974 |
| ENSP00000359783 | IFI44 | 0.3974 |
| ENSP00000339801 | IDS | 0.3974 |
| ENSP00000261374 | HS3ST2 | 0.3974 |
| ENSP00000358160 | HIST1H3H | 0.3974 |
| ENSP00000439660 | HIST1H3G | 0.3974 |
| ENSP00000347119 | H2BFM | 0.3974 |
| ENSP00000256594 | GSTM3 | 0.3974 |
| ENSP00000359998 | GSTA4 | 0.3974 |
| ENSP00000455469 | GJE1 | 0.3974 |
| ENSP00000320180 | GHRHR | 0.3974 |

| | | |
|---|---|---|
| ENSP00000249041 | GALR3 | 0.3974 |
| ENSP00000296591 | EDIL3 | 0.3974 |
| ENSP00000315465 | DSCAML1 | 0.3974 |
| ENSP00000371846 | DEAF1 | 0.3974 |
| ENSP00000386870 | DCDC2B | 0.3974 |
| ENSP00000351059 | CX3CR1 | 0.3974 |
| ENSP00000217423 | CST4 | 0.3974 |
| ENSP00000304822 | CSN3 | 0.3974 |
| ENSP00000441462 | CSMD1 | 0.3974 |
| ENSP00000255030 | CRP | 0.3974 |
| ENSP00000339155 | CRISP2 | 0.3974 |
| ENSP00000351310 | COL6A6 | 0.3974 |
| ENSP00000460850 | CLUAP1 | 0.3974 |
| ENSP00000234488 | CLCN6 | 0.3974 |
| ENSP00000263671 | CHRDL2 | 0.3974 |
| ENSP00000200676 | CETP | 0.3974 |
| ENSP00000354458 | C8A | 0.3974 |
| ENSP00000358575 | C6orf165 | 0.3974 |
| ENSP00000334130 | C11orf30 | 0.3974 |
| ENSP00000306822 | AVEN | 0.3974 |
| ENSP00000410759 | ATXN7L1 | 0.3974 |
| ENSP00000247178 | ATG14 | 0.3974 |
| ENSP00000383059 | ARGLU1 | 0.3974 |
| ENSP00000350541 | AQPEP | 0.3974 |
| ENSP00000341674 | ANXA8 | 0.3974 |
| ENSP00000257527 | ADAM19 | 0.3974 |
| ENSP00000248450 | AAMP | 0.3974 |
| ENSP00000346572 | 61E3.4 | 0.3974 |
| ENSP00000451628 | ENSG00000258447 | 0.3974 |
| ENSP00000318373 | ZNF513 | 0.3947 |
| ENSP00000418259 | ZFP57 | 0.3947 |
| ENSP00000352821 | ZDHHC14 | 0.3947 |
| ENSP00000419153 | ZBTB20 | 0.3947 |
| ENSP00000312272 | YPEL2 | 0.3947 |
| ENSP00000402219 | VSIG1 | 0.3947 |
| ENSP00000219689 | USP31 | 0.3947 |
| ENSP00000257548 | USP30 | 0.3947 |
| ENSP00000391879 | TRIM26 | 0.3947 |
| ENSP00000369299 | TRIM22 | 0.3947 |
| ENSP00000299045 | TCP11L2 | 0.3947 |
| ENSP00000367802 | TAF1C | 0.3947 |
| ENSP00000443168 | STH | 0.3947 |
| ENSP00000319678 | ST5 | 0.3947 |
| ENSP00000307340 | SPRR1A | 0.3947 |
| ENSP00000023064 | SLC7A9 | 0.3947 |
| ENSP00000330199 | SLC6A17 | 0.3947 |
| ENSP00000254488 | SLC6A11 | 0.3947 |
| ENSP00000296589 | SLC45A2 | 0.3947 |
| ENSP00000400932 | SLC35G1 | 0.3947 |
| ENSP00000280871 | SLC2A13 | 0.3947 |
| ENSP00000314606 | SGSH | 0.3947 |
| ENSP00000310803 | SFT2D3 | 0.3947 |
| ENSP00000265361 | SEMA3C | 0.3947 |

| | | |
|---|---|---|
| ENSP00000296694 | SCGB3A2 | 0.3947 |
| ENSP00000395259 | SCAND3 | 0.3947 |
| ENSP00000253788 | RPL27 | 0.3947 |
| ENSP00000432029 | RP11-719K4.2 | 0.3947 |
| ENSP00000435325 | RP11-1212A22.2 | 0.3947 |
| ENSP00000256257 | RNF122 | 0.3947 |
| ENSP00000337623 | RNASEH2B | 0.3947 |
| ENSP00000414330 | RIMKLA | 0.3947 |
| ENSP00000376583 | RIC8B | 0.3947 |
| ENSP00000258302 | RGS8 | 0.3947 |
| ENSP00000363516 | RCAN3 | 0.3947 |
| ENSP00000282884 | RASSF8 | 0.3947 |
| ENSP00000344547 | PTMA | 0.3947 |
| ENSP00000272847 | PTH2R | 0.3947 |
| ENSP00000452296 | POTEM | 0.3947 |
| ENSP00000429344 | PNMA2 | 0.3947 |
| ENSP00000274793 | PLA2G7 | 0.3947 |
| ENSP00000433597 | PKD1P1 | 0.3947 |
| ENSP00000363124 | PHF20 | 0.3947 |
| ENSP00000326519 | PAGR1 | 0.3947 |
| ENSP00000316454 | PACS1 | 0.3947 |
| ENSP00000247026 | NSRP1 | 0.3947 |
| ENSP00000370372 | NLN | 0.3947 |
| ENSP00000303580 | NKIRAS2 | 0.3947 |
| ENSP00000392057 | MTUS2 | 0.3947 |
| ENSP00000338235 | MTDH | 0.3947 |
| ENSP00000447547 | MRPL42 | 0.3947 |
| ENSP00000264790 | MMRN1 | 0.3947 |
| ENSP00000246186 | MMP24 | 0.3947 |
| ENSP00000170447 | MKRN2 | 0.3947 |
| ENSP00000292596 | LTC4S | 0.3947 |
| ENSP00000301873 | LTBP3 | 0.3947 |
| ENSP00000259324 | LRRC8A | 0.3947 |
| ENSP00000269025 | LRRC46 | 0.3947 |
| ENSP00000335223 | LCE4A | 0.3947 |
| ENSP00000381009 | KRTAP10-9 | 0.3947 |
| ENSP00000375682 | KLK8 | 0.3947 |
| ENSP00000386389 | KLHL30 | 0.3947 |
| ENSP00000367075 | KLHL1 | 0.3947 |
| ENSP00000312129 | KCNG4 | 0.3947 |
| ENSP00000262648 | KAL1 | 0.3947 |
| ENSP00000264260 | IL18RAP | 0.3947 |
| ENSP00000369571 | IFNA14 | 0.3947 |
| ENSP00000429473 | HS6ST2 | 0.3947 |
| ENSP00000308107 | HPSE | 0.3947 |
| ENSP00000414109 | HJURP | 0.3947 |
| ENSP00000358154 | HIST2H3C | 0.3947 |
| ENSP00000397566 | GTF2IRD1 | 0.3947 |
| ENSP00000243673 | GPR83 | 0.3947 |
| ENSP00000406367 | GPR124 | 0.3947 |
| ENSP00000271732 | GOLPH3L | 0.3947 |
| ENSP00000266068 | GMEB2 | 0.3947 |
| ENSP00000282570 | GMCL1 | 0.3947 |

| | | |
|---|---|---|
| ENSP00000358358 | GJA10 | 0.3947 |
| ENSP00000419751 | GGTLC2 | 0.3947 |
| ENSP00000369600 | FRY | 0.3947 |
| ENSP00000400223 | FJX1 | 0.3947 |
| ENSP00000359375 | FATE1 | 0.3947 |
| ENSP00000330875 | FANCF | 0.3947 |
| ENSP00000384880 | FAM49B | 0.3947 |
| ENSP00000382646 | FAM166B | 0.3947 |
| ENSP00000355204 | FAM110B | 0.3947 |
| ENSP00000352540 | EMP2 | 0.3947 |
| ENSP00000317564 | EID2B | 0.3947 |
| ENSP00000253392 | EDA2R | 0.3947 |
| ENSP00000271385 | DUSP27 | 0.3947 |
| ENSP00000322180 | DSCC1 | 0.3947 |
| ENSP00000371451 | DNAJC21 | 0.3947 |
| ENSP00000280056 | DHRS12 | 0.3947 |
| ENSP00000316476 | DEGS1 | 0.3947 |
| ENSP00000323155 | DAND5 | 0.3947 |
| ENSP00000285949 | CYP26C1 | 0.3947 |
| ENSP00000273153 | CSRNP1 | 0.3947 |
| ENSP00000338276 | CRISP1 | 0.3947 |
| ENSP00000220763 | CPQ | 0.3947 |
| ENSP00000396163 | CLEC2A | 0.3947 |
| ENSP00000362207 | CHST3 | 0.3947 |
| ENSP00000255380 | CHRM3 | 0.3947 |
| ENSP00000439856 | CHERP | 0.3947 |
| ENSP00000293636 | CELA1 | 0.3947 |
| ENSP00000250535 | CDO1 | 0.3947 |
| ENSP00000242786 | CD97 | 0.3947 |
| ENSP00000283285 | CD96 | 0.3947 |
| ENSP00000356545 | CACNA1E | 0.3947 |
| ENSP00000361005 | ATPAF1 | 0.3947 |
| ENSP00000427941 | ATP6V0E1 | 0.3947 |
| ENSP00000367638 | ARHGEF39 | 0.3947 |
| ENSP00000350425 | APOA4 | 0.3947 |
| ENSP00000298861 | AGBL2 | 0.3947 |
| ENSP00000284425 | ABCA6 | 0.3947 |
| ENSP00000272895 | ABCA12 | 0.3947 |
| ENSP00000404439 | 61E3.4 | 0.3947 |
| ENSP00000430853 | 61E3.4 | 0.3947 |
| ENSP00000297857 | ZHX1 | 0.3921 |
| ENSP00000370303 | ZDHHC21 | 0.3921 |
| ENSP00000279281 | VPS51 | 0.3921 |
| ENSP00000314556 | UACA | 0.3921 |
| ENSP00000362546 | TTI1 | 0.3921 |
| ENSP00000422496 | TTC39B | 0.3921 |
| ENSP00000173898 | TRO | 0.3921 |
| ENSP00000362013 | TRERF1 | 0.3921 |
| ENSP00000330289 | TRAPPC6B | 0.3921 |
| ENSP00000352413 | TMC3 | 0.3921 |
| ENSP00000317170 | TIGD2 | 0.3921 |
| ENSP00000406052 | TDRD5 | 0.3921 |
| ENSP00000342098 | SPINT1 | 0.3921 |

| | | |
|---|---|---|
| ENSP00000375899 | SP140 | 0.3921 |
| ENSP00000233575 | SNX17 | 0.3921 |
| ENSP00000308893 | SNED1 | 0.3921 |
| ENSP00000270066 | SMG9 | 0.3921 |
| ENSP00000349174 | SLC39A8 | 0.3921 |
| ENSP00000381634 | SLC38A1 | 0.3921 |
| ENSP00000334223 | SLC36A2 | 0.3921 |
| ENSP00000333591 | SLC35D3 | 0.3921 |
| ENSP00000362469 | SLC2A8 | 0.3921 |
| ENSP00000297578 | SLC25A32 | 0.3921 |
| ENSP00000391249 | SEPT9 | 0.3921 |
| ENSP00000306844 | SEMA4C | 0.3921 |
| ENSP00000429969 | SCUBE2 | 0.3921 |
| ENSP00000370196 | SBF1 | 0.3921 |
| ENSP00000431814 | RP11-467M13.1 | 0.3921 |
| ENSP00000415840 | RP11-295K3.1 | 0.3921 |
| ENSP00000295317 | RNF149 | 0.3921 |
| ENSP00000341874 | RIOK3 | 0.3921 |
| ENSP00000294413 | RHCE | 0.3921 |
| ENSP00000295971 | RBM47 | 0.3921 |
| ENSP00000362123 | PLA2G12B | 0.3921 |
| ENSP00000376865 | PDGFD | 0.3921 |
| ENSP00000328216 | ORAI1 | 0.3921 |
| ENSP00000340328 | NYX | 0.3921 |
| ENSP00000330070 | NPW | 0.3921 |
| ENSP00000292199 | NLRX1 | 0.3921 |
| ENSP00000216121 | NIPSNAP1 | 0.3921 |
| ENSP00000268766 | NEK8 | 0.3921 |
| ENSP00000311364 | MUCL1 | 0.3921 |
| ENSP00000261413 | MRPS27 | 0.3921 |
| ENSP00000244230 | MPHOSPH10 | 0.3921 |
| ENSP00000306220 | MMGT1 | 0.3921 |
| ENSP00000262370 | MGRN1 | 0.3921 |
| ENSP00000296350 | MFI2 | 0.3921 |
| ENSP00000276282 | MFHAS1 | 0.3921 |
| ENSP00000268150 | MFGE8 | 0.3921 |
| ENSP00000376127 | MAU2 | 0.3921 |
| ENSP00000285879 | MAGEC1 | 0.3921 |
| ENSP00000337733 | KLK5 | 0.3921 |
| ENSP00000301420 | KLK1 | 0.3921 |
| ENSP00000327468 | KLHDC8B | 0.3921 |
| ENSP00000354848 | KIAA1279 | 0.3921 |
| ENSP00000280684 | KCNA6 | 0.3921 |
| ENSP00000233957 | IL18R1 | 0.3921 |
| ENSP00000316664 | IGSF8 | 0.3921 |
| ENSP00000347754 | HTR3A | 0.3921 |
| ENSP00000384889 | HIC2 | 0.3921 |
| ENSP00000300605 | HDHD2 | 0.3921 |
| ENSP00000358452 | H2AFB3 | 0.3921 |
| ENSP00000345023 | GSTO2 | 0.3921 |
| ENSP00000431049 | GSTK1 | 0.3921 |
| ENSP00000417161 | GPR143 | 0.3921 |
| ENSP00000267731 | GOLGA8B | 0.3921 |

| | | |
|---|---|---|
| ENSP00000350415 | GJA9 | 0.3921 |
| ENSP00000241256 | GHSR | 0.3921 |
| ENSP00000357914 | GABPB2 | 0.3921 |
| ENSP00000246841 | FLRT1 | 0.3921 |
| ENSP00000251547 | FBXO44 | 0.3921 |
| ENSP00000371805 | FBXL18 | 0.3921 |
| ENSP00000377549 | FABP6 | 0.3921 |
| ENSP00000363616 | EDEM2 | 0.3921 |
| ENSP00000302051 | ECEL1 | 0.3921 |
| ENSP00000341658 | DUSP15 | 0.3921 |
| ENSP00000450980 | DIO2 | 0.3921 |
| ENSP00000275884 | DENND2A | 0.3921 |
| ENSP00000334592 | CYP2R1 | 0.3921 |
| ENSP00000282251 | CWF19L2 | 0.3921 |
| ENSP00000317257 | CPNE1 | 0.3921 |
| ENSP00000330730 | COA5 | 0.3921 |
| ENSP00000368824 | CNKSR2 | 0.3921 |
| ENSP00000318113 | CLDN22 | 0.3921 |
| ENSP00000363041 | CISD1 | 0.3921 |
| ENSP00000254190 | CHSY1 | 0.3921 |
| ENSP00000035307 | CHPF2 | 0.3921 |
| ENSP00000329507 | CD300C | 0.3921 |
| ENSP00000299367 | C2 | 0.3921 |
| ENSP00000435634 | C1orf94 | 0.3921 |
| ENSP00000381377 | C15orf38-AP3S2 | 0.3921 |
| ENSP00000265016 | BST1 | 0.3921 |
| ENSP00000338814 | BAG5 | 0.3921 |
| ENSP00000312457 | BAALC | 0.3921 |
| ENSP00000339404 | ASCC1 | 0.3921 |
| ENSP00000391137 | ASB10 | 0.3921 |
| ENSP00000312458 | ARV1 | 0.3921 |
| ENSP00000345179 | APOD | 0.3921 |
| ENSP00000321679 | ANKRD23 | 0.3921 |
| ENSP00000382218 | ZSWIM7 | 0.3895 |
| ENSP00000307746 | ZNF608 | 0.3895 |
| ENSP00000387091 | ZNF365 | 0.3895 |
| ENSP00000368801 | WBP4 | 0.3895 |
| ENSP00000303908 | TWF2 | 0.3895 |
| ENSP00000325266 | TTC9C | 0.3895 |
| ENSP00000374332 | TSN | 0.3895 |
| ENSP00000349324 | TNFRSF10C | 0.3895 |
| ENSP00000374484 | TMEM87A | 0.3895 |
| ENSP00000222543 | TFPI2 | 0.3895 |
| ENSP00000399753 | TEX13A | 0.3895 |
| ENSP00000349291 | TBC1D9B | 0.3895 |
| ENSP00000331867 | TAS1R1 | 0.3895 |
| ENSP00000408792 | SP8 | 0.3895 |
| ENSP00000323549 | SLC6A18 | 0.3895 |
| ENSP00000202831 | SLC24A6 | 0.3895 |
| ENSP00000406546 | SLC23A3 | 0.3895 |
| ENSP00000337335 | SLC22A8 | 0.3895 |
| ENSP00000245407 | SLC22A5 | 0.3895 |
| ENSP00000396586 | SLC22A24 | 0.3895 |

| | | |
|---|---|---|
| ENSP00000279477 | SIRPB1 | 0.3895 |
| ENSP00000264677 | SERPINI2 | 0.3895 |
| ENSP00000328729 | SEP15 | 0.3895 |
| ENSP00000437133 | RP11-719K4.1 | 0.3895 |
| ENSP00000355308 | PTPLA | 0.3895 |
| ENSP00000365963 | PPP1R11 | 0.3895 |
| ENSP00000386971 | POTEG | 0.3895 |
| ENSP00000301908 | PNOC | 0.3895 |
| ENSP00000361508 | PLTP | 0.3895 |
| ENSP00000364243 | PLA2G2F | 0.3895 |
| ENSP00000357069 | PIGM | 0.3895 |
| ENSP00000362456 | PBDC1 | 0.3895 |
| ENSP00000336607 | P2RX4 | 0.3895 |
| ENSP00000353078 | NRAP | 0.3895 |
| ENSP00000367398 | NPHP4 | 0.3895 |
| ENSP00000296597 | NDUFAF2 | 0.3895 |
| ENSP00000354223 | MPC1 | 0.3895 |
| ENSP00000391227 | MGAT5B | 0.3895 |
| ENSP00000307423 | MGAT2 | 0.3895 |
| ENSP00000251472 | MAST1 | 0.3895 |
| ENSP00000325313 | MAP1S | 0.3895 |
| ENSP00000349085 | MAGEA1 | 0.3895 |
| ENSP00000307445 | LTB4R | 0.3895 |
| ENSP00000454748 | LRRC9 | 0.3895 |
| ENSP00000332674 | LRRC8B | 0.3895 |
| ENSP00000249363 | LRRC4 | 0.3895 |
| ENSP00000375478 | KRTAP10-3 | 0.3895 |
| ENSP00000369349 | KRT81 | 0.3895 |
| ENSP00000293303 | KLHL10 | 0.3895 |
| ENSP00000272748 | KIAA1715 | 0.3895 |
| ENSP00000260598 | KHK | 0.3895 |
| ENSP00000331698 | KCNJ15 | 0.3895 |
| ENSP00000233826 | KCNJ13 | 0.3895 |
| ENSP00000267615 | ITPK1 | 0.3895 |
| ENSP00000356063 | IL20 | 0.3895 |
| ENSP00000282466 | IGSF10 | 0.3895 |
| ENSP00000394494 | IFNA13 | 0.3895 |
| ENSP00000292433 | IER2 | 0.3895 |
| ENSP00000302562 | HSPBAP1 | 0.3895 |
| ENSP00000376162 | HOMER3 | 0.3895 |
| ENSP00000349478 | HCAR1 | 0.3895 |
| ENSP00000211122 | GSTA3 | 0.3895 |
| ENSP00000254799 | GRSF1 | 0.3895 |
| ENSP00000404063 | GRIK4 | 0.3895 |
| ENSP00000352111 | GOLGA8A | 0.3895 |
| ENSP00000268719 | GID4 | 0.3895 |
| ENSP00000318142 | FUT7 | 0.3895 |
| ENSP00000312021 | FUT1 | 0.3895 |
| ENSP00000288078 | FUK | 0.3895 |
| ENSP00000263578 | FOXRED1 | 0.3895 |
| ENSP00000257209 | FHOD3 | 0.3895 |
| ENSP00000264072 | FCER2 | 0.3895 |
| ENSP00000262365 | FAM86B2 | 0.3895 |

| | | |
|---|---|---|
| ENSP00000358458 | FAM46C | 0.3895 |
| ENSP00000356058 | FAIM3 | 0.3895 |
| ENSP00000202816 | ESF1 | 0.3895 |
| ENSP00000318982 | EPSTI1 | 0.3895 |
| ENSP00000346412 | ELP5 | 0.3895 |
| ENSP00000269445 | DYM | 0.3895 |
| ENSP00000360635 | DPP7 | 0.3895 |
| ENSP00000328690 | DDX24 | 0.3895 |
| ENSP00000440698 | DBI | 0.3895 |
| ENSP00000362401 | CYSLTR1 | 0.3895 |
| ENSP00000388320 | CTU2 | 0.3895 |
| ENSP00000241305 | CPXM2 | 0.3895 |
| ENSP00000369979 | CPXM1 | 0.3895 |
| ENSP00000351767 | COL20A1 | 0.3895 |
| ENSP00000365256 | CLCN5 | 0.3895 |
| ENSP00000258711 | CHST12 | 0.3895 |
| ENSP00000287202 | CELF6 | 0.3895 |
| ENSP00000256343 | CATSPERB | 0.3895 |
| ENSP00000344566 | C21orf2 | 0.3895 |
| ENSP00000216442 | ATP6V1D | 0.3895 |
| ENSP00000218008 | ATP1B4 | 0.3895 |
| ENSP00000368062 | ATAD3C | 0.3895 |
| ENSP00000368767 | ASPH | 0.3895 |
| ENSP00000320893 | ASB8 | 0.3895 |
| ENSP00000299381 | ANAPC16 | 0.3895 |
| ENSP00000264595 | AGA | 0.3895 |
| ENSP00000379086 | ADM2 | 0.3895 |
| ENSP00000468389 | AC002398.9 | 0.3895 |
| ENSP00000456021 | ENSG00000260527 | 0.3895 |
| ENSP00000405289 | ZNF778 | 0.3868 |
| ENSP00000155093 | ZFY | 0.3868 |
| ENSP00000317686 | ZFP42 | 0.3868 |
| ENSP00000413418 | ZFHX2 | 0.3868 |
| ENSP00000349689 | ZDHHC9 | 0.3868 |
| ENSP00000287387 | WDYHV1 | 0.3868 |
| ENSP00000366314 | TRPM3 | 0.3868 |
| ENSP00000363942 | TP53INP2 | 0.3868 |
| ENSP00000245817 | TNFSF9 | 0.3868 |
| ENSP00000363157 | TNFSF15 | 0.3868 |
| ENSP00000363189 | TMEM222 | 0.3868 |
| ENSP00000357804 | THEM4 | 0.3868 |
| ENSP00000366307 | THBD | 0.3868 |
| ENSP00000306015 | SYNPO2 | 0.3868 |
| ENSP00000312329 | STAG3L3 | 0.3868 |
| ENSP00000368808 | SMPX | 0.3868 |
| ENSP00000357754 | SMCP | 0.3868 |
| ENSP00000366942 | SLC36A3 | 0.3868 |
| ENSP00000295777 | SERPINI1 | 0.3868 |
| ENSP00000330631 | RTN4RL1 | 0.3868 |
| ENSP00000262340 | RPE65 | 0.3868 |
| ENSP00000420037 | PIGP | 0.3868 |
| ENSP00000300658 | PGAP3 | 0.3868 |
| ENSP00000263549 | PARP12 | 0.3868 |

| | | |
|---|---|---|
| ENSP00000203664 | OTUB2 | 0.3868 |
| ENSP00000259396 | ORM1 | 0.3868 |
| ENSP00000436455 | NUCB2 | 0.3868 |
| ENSP00000346890 | NRD1 | 0.3868 |
| ENSP00000366061 | NMS | 0.3868 |
| ENSP00000246117 | NCLN | 0.3868 |
| ENSP00000443194 | NAT1 | 0.3868 |
| ENSP00000357360 | MTX1 | 0.3868 |
| ENSP00000265517 | MTTP | 0.3868 |
| ENSP00000356552 | MR1 | 0.3868 |
| ENSP00000281806 | MCHR2 | 0.3868 |
| ENSP00000262811 | MAST3 | 0.3868 |
| ENSP00000364777 | MAGED4 | 0.3868 |
| ENSP00000305742 | LRTOMT | 0.3868 |
| ENSP00000300417 | LRSAM1 | 0.3868 |
| ENSP00000400803 | LRRC69 | 0.3868 |
| ENSP00000374447 | LRCH1 | 0.3868 |
| ENSP00000238875 | LGALSL | 0.3868 |
| ENSP00000371661 | LCORL | 0.3868 |
| ENSP00000341342 | KLHL6 | 0.3868 |
| ENSP00000359942 | KLHL31 | 0.3868 |
| ENSP00000312814 | KCTD2 | 0.3868 |
| ENSP00000319370 | KCNMB3 | 0.3868 |
| ENSP00000396749 | KANSL3 | 0.3868 |
| ENSP00000369578 | IFNW1 | 0.3868 |
| ENSP00000342513 | IFI6 | 0.3868 |
| ENSP00000305721 | IBTK | 0.3868 |
| ENSP00000281938 | HSPB8 | 0.3868 |
| ENSP00000366999 | HIST1H3D | 0.3868 |
| ENSP00000362810 | HCRTR1 | 0.3868 |
| ENSP00000349270 | HBM | 0.3868 |
| ENSP00000359239 | HAUS7 | 0.3868 |
| ENSP00000354723 | H2BFWT | 0.3868 |
| ENSP00000344260 | GALNT15 | 0.3868 |
| ENSP00000288988 | GALNT14 | 0.3868 |
| ENSP00000353910 | FUT8 | 0.3868 |
| ENSP00000002165 | FUCA2 | 0.3868 |
| ENSP00000351113 | FRYL | 0.3868 |
| ENSP00000386881 | DYSF | 0.3868 |
| ENSP00000373691 | DUOX2 | 0.3868 |
| ENSP00000349727 | DOK3 | 0.3868 |
| ENSP00000264057 | DGKD | 0.3868 |
| ENSP00000387426 | CYHR1 | 0.3868 |
| ENSP00000322316 | COQ7 | 0.3868 |
| ENSP00000289382 | CNOT11 | 0.3868 |
| ENSP00000364831 | CLCNKB | 0.3868 |
| ENSP00000327179 | CIRH1A | 0.3868 |
| ENSP00000339723 | CIR1 | 0.3868 |
| ENSP00000341828 | CHIA | 0.3868 |
| ENSP00000334229 | C2CD5 | 0.3868 |
| ENSP00000260502 | BCAR3 | 0.3868 |
| ENSP00000352319 | ARSF | 0.3868 |
| ENSP00000306788 | ARL6IP1 | 0.3868 |

| | | |
|---|---|---|
| ENSP00000370521 | AIPL1 | 0.3868 |
| ENSP00000362799 | ACRC | 0.3868 |
| ENSP00000250974 | ABHD17A | 0.3868 |
| ENSP00000325634 | ZNF639 | 0.3842 |
| ENSP00000305077 | ZNF439 | 0.3842 |
| ENSP00000300022 | YPEL4 | 0.3842 |
| ENSP00000328079 | WDR53 | 0.3842 |
| ENSP00000251289 | WDR18 | 0.3842 |
| ENSP00000462337 | VEZF1 | 0.3842 |
| ENSP00000396068 | UBR3 | 0.3842 |
| ENSP00000369465 | TXLNG | 0.3842 |
| ENSP00000386181 | TTC30B | 0.3842 |
| ENSP00000365924 | TRIM31 | 0.3842 |
| ENSP00000362424 | TRAF3IP1 | 0.3842 |
| ENSP00000223795 | TNFSF8 | 0.3842 |
| ENSP00000433361 | TMEM9B | 0.3842 |
| ENSP00000300128 | TMEM194A | 0.3842 |
| ENSP00000268674 | TIGD7 | 0.3842 |
| ENSP00000263707 | TFCP2L1 | 0.3842 |
| ENSP00000280358 | TEX12 | 0.3842 |
| ENSP00000331302 | TCEB3B | 0.3842 |
| ENSP00000371394 | SYT8 | 0.3842 |
| ENSP00000319318 | STAG3 | 0.3842 |
| ENSP00000332931 | SLC8A1 | 0.3842 |
| ENSP00000289952 | SLC39A14 | 0.3842 |
| ENSP00000362285 | SLC29A3 | 0.3842 |
| ENSP00000355141 | SEPN1 | 0.3842 |
| ENSP00000301293 | SEMA6B | 0.3842 |
| ENSP00000350547 | SEMA4F | 0.3842 |
| ENSP00000293502 | SDR9C7 | 0.3842 |
| ENSP00000274938 | SCUBE3 | 0.3842 |
| ENSP00000357697 | S100A2 | 0.3842 |
| ENSP00000369257 | RPGRIP1L | 0.3842 |
| ENSP00000360217 | RHAG | 0.3842 |
| ENSP00000428343 | RGS21 | 0.3842 |
| ENSP00000258062 | REPS1 | 0.3842 |
| ENSP00000262879 | RALGAPB | 0.3842 |
| ENSP00000311572 | PTGR1 | 0.3842 |
| ENSP00000225573 | PNPO | 0.3842 |
| ENSP00000317177 | PLD6 | 0.3842 |
| ENSP00000247992 | PLA2G2C | 0.3842 |
| ENSP00000241041 | PEX16 | 0.3842 |
| ENSP00000297565 | OSR2 | 0.3842 |
| ENSP00000455169 | NETO2 | 0.3842 |
| ENSP00000284503 | NEIL2 | 0.3842 |
| ENSP00000380702 | MYCBP | 0.3842 |
| ENSP00000362718 | MTCH1 | 0.3842 |
| ENSP00000373261 | MOBP | 0.3842 |
| ENSP00000298717 | METTL3 | 0.3842 |
| ENSP00000353098 | MCMBP | 0.3842 |
| ENSP00000363452 | MAN1C1 | 0.3842 |
| ENSP00000364467 | LYZL2 | 0.3842 |
| ENSP00000364650 | LYZL1 | 0.3842 |

| | | |
|---|---|---|
| ENSP00000354575 | LSR | 0.3842 |
| ENSP00000375857 | LRRFIP1 | 0.3842 |
| ENSP00000240618 | KLRK1 | 0.3842 |
| ENSP00000321475 | KLHL38 | 0.3842 |
| ENSP00000290310 | KCNE2 | 0.3842 |
| ENSP00000395650 | KANK2 | 0.3842 |
| ENSP00000384198 | INO80D | 0.3842 |
| ENSP00000313661 | HTR1D | 0.3842 |
| ENSP00000365895 | HS6ST3 | 0.3842 |
| ENSP00000341108 | HMX2 | 0.3842 |
| ENSP00000297440 | HEATR2 | 0.3842 |
| ENSP00000307342 | HCN1 | 0.3842 |
| ENSP00000261208 | HAL | 0.3842 |
| ENSP00000204679 | GNPTG | 0.3842 |
| ENSP00000331831 | GAS6 | 0.3842 |
| ENSP00000410732 | GABRG2 | 0.3842 |
| ENSP00000399588 | GAB3 | 0.3842 |
| ENSP00000269097 | G6PC3 | 0.3842 |
| ENSP00000280481 | FREM2 | 0.3842 |
| ENSP00000435061 | FNTA | 0.3842 |
| ENSP00000335655 | FMNL3 | 0.3842 |
| ENSP00000339912 | FLRT3 | 0.3842 |
| ENSP00000217289 | FERMT1 | 0.3842 |
| ENSP00000326819 | FANCB | 0.3842 |
| ENSP00000354669 | FAM63A | 0.3842 |
| ENSP00000410603 | FAM131B | 0.3842 |
| ENSP00000394352 | DIXDC1 | 0.3842 |
| ENSP00000334801 | DHRS4L2 | 0.3842 |
| ENSP00000380352 | DDHD2 | 0.3842 |
| ENSP00000285979 | CYP2C18 | 0.3842 |
| ENSP00000361628 | CXorf57 | 0.3842 |
| ENSP00000381273 | CRYBB2 | 0.3842 |
| ENSP00000340943 | CRHR2 | 0.3842 |
| ENSP00000383938 | CRELD2 | 0.3842 |
| ENSP00000290776 | CPNE2 | 0.3842 |
| ENSP00000359098 | COX7A2 | 0.3842 |
| ENSP00000299886 | COG1 | 0.3842 |
| ENSP00000314544 | CCZ1B | 0.3842 |
| ENSP00000240079 | CCDC53 | 0.3842 |
| ENSP00000252729 | CACNG6 | 0.3842 |
| ENSP00000412388 | C9orf172 | 0.3842 |
| ENSP00000379092 | BTC | 0.3842 |
| ENSP00000354032 | BSCL2 | 0.3842 |
| ENSP00000265523 | BLVRA | 0.3842 |
| ENSP00000264381 | BCHE | 0.3842 |
| ENSP00000320965 | B4GALT3 | 0.3842 |
| ENSP00000315568 | AVL9 | 0.3842 |
| ENSP00000351155 | ATL1 | 0.3842 |
| ENSP00000378897 | ASAH2 | 0.3842 |
| ENSP00000335560 | ARHGEF25 | 0.3842 |
| ENSP00000391596 | AMDHD2 | 0.3842 |
| ENSP00000318313 | AMBRA1 | 0.3842 |
| ENSP00000433931 | AIM1L | 0.3842 |

| | | |
|---|---|---|
| ENSP00000365251 | AGTPBP1 | 0.3842 |
| ENSP00000358413 | ZDHHC6 | 0.3816 |
| ENSP00000301011 | ZC3H18 | 0.3816 |
| ENSP00000423333 | USP51 | 0.3816 |
| ENSP00000449428 | TWF1 | 0.3816 |
| ENSP00000328875 | TRAK2 | 0.3816 |
| ENSP00000350990 | TNKS1BP1 | 0.3816 |
| ENSP00000308753 | TMOD3 | 0.3816 |
| ENSP00000262817 | TMEM59L | 0.3816 |
| ENSP00000281017 | TMEM18 | 0.3816 |
| ENSP00000360184 | TM9SF3 | 0.3816 |
| ENSP00000357631 | TCERG1L | 0.3816 |
| ENSP00000386170 | TBX20 | 0.3816 |
| ENSP00000361731 | TBC1D13 | 0.3816 |
| ENSP00000248121 | SYNGR3 | 0.3816 |
| ENSP00000337926 | SUGP2 | 0.3816 |
| ENSP00000356172 | SOX13 | 0.3816 |
| ENSP00000372482 | SNRNP25 | 0.3816 |
| ENSP00000353791 | SLC6A9 | 0.3816 |
| ENSP00000360967 | SLC6A14 | 0.3816 |
| ENSP00000352702 | SLC6A12 | 0.3816 |
| ENSP00000266579 | SLC38A4 | 0.3816 |
| ENSP00000309504 | SLC26A7 | 0.3816 |
| ENSP00000279027 | SLC13A3 | 0.3816 |
| ENSP00000262866 | SLA2 | 0.3816 |
| ENSP00000390084 | SHISA6 | 0.3816 |
| ENSP00000265965 | SERGEF | 0.3816 |
| ENSP00000349929 | SDCCAG3 | 0.3816 |
| ENSP00000358374 | SCAF11 | 0.3816 |
| ENSP00000467041 | RP11-322E11.6 | 0.3816 |
| ENSP00000259939 | RNF144B | 0.3816 |
| ENSP00000377055 | PRCP | 0.3816 |
| ENSP00000420826 | PRB1 | 0.3816 |
| ENSP00000257905 | PPP1R1A | 0.3816 |
| ENSP00000322373 | PLEKHF2 | 0.3816 |
| ENSP00000239231 | PANK3 | 0.3816 |
| ENSP00000361558 | NTMT1 | 0.3816 |
| ENSP00000371101 | NOL10 | 0.3816 |
| ENSP00000371577 | MTMR12 | 0.3816 |
| ENSP00000402537 | MTMR10 | 0.3816 |
| ENSP00000377995 | MT1X | 0.3816 |
| ENSP00000307636 | MORN4 | 0.3816 |
| ENSP00000356859 | MGST3 | 0.3816 |
| ENSP00000264036 | MCAM | 0.3816 |
| ENSP00000362870 | MATN1 | 0.3816 |
| ENSP00000215739 | LZTR1 | 0.3816 |
| ENSP00000355187 | LRRTM3 | 0.3816 |
| ENSP00000312535 | LRFN4 | 0.3816 |
| ENSP00000357778 | LCE3E | 0.3816 |
| ENSP00000284669 | KLHL41 | 0.3816 |
| ENSP00000270583 | KLHDC4 | 0.3816 |
| ENSP00000379654 | KIAA0430 | 0.3816 |
| ENSP00000259154 | KCTD3 | 0.3816 |

| | | |
|---|---|---|
| ENSP00000443432 | IDO2 | 0.3816 |
| ENSP00000256906 | HRH4 | 0.3816 |
| ENSP00000263208 | HIRA | 0.3816 |
| ENSP00000439056 | HAUS5 | 0.3816 |
| ENSP00000329662 | H1FX | 0.3816 |
| ENSP00000330148 | GRID1 | 0.3816 |
| ENSP00000306449 | GPR37 | 0.3816 |
| ENSP00000366157 | GPR180 | 0.3816 |
| ENSP00000313050 | GBAS | 0.3816 |
| ENSP00000258201 | FHOD1 | 0.3816 |
| ENSP00000221466 | FCGRT | 0.3816 |
| ENSP00000369625 | FAM50B | 0.3816 |
| ENSP00000265840 | ELMOD1 | 0.3816 |
| ENSP00000445626 | EGR4 | 0.3816 |
| ENSP00000353701 | DPP3 | 0.3816 |
| ENSP00000417182 | DPCR1 | 0.3816 |
| ENSP00000261811 | CYSTM1 | 0.3816 |
| ENSP00000373244 | CSPG5 | 0.3816 |
| ENSP00000363590 | CSGALNACT2 | 0.3816 |
| ENSP00000323696 | CRTAP | 0.3816 |
| ENSP00000265379 | COL6A5 | 0.3816 |
| ENSP00000356133 | CLDN20 | 0.3816 |
| ENSP00000265593 | CLCN2 | 0.3816 |
| ENSP00000269967 | CCDC97 | 0.3816 |
| ENSP00000376177 | CALCRL | 0.3816 |
| ENSP00000346017 | CALCB | 0.3816 |
| ENSP00000366030 | C9orf41 | 0.3816 |
| ENSP00000243611 | C4BPB | 0.3816 |
| ENSP00000356037 | C4BPA | 0.3816 |
| ENSP00000299578 | C16orf46 | 0.3816 |
| ENSP00000264914 | ARSB | 0.3816 |
| ENSP00000187397 | ARPP21 | 0.3816 |
| ENSP00000354432 | ANKRD30A | 0.3816 |
| ENSP00000269701 | AKAP8 | 0.3816 |
| ENSP00000324827 | AKAP17A | 0.3816 |
| ENSP00000413001 | AGBL1 | 0.3816 |
| ENSP00000453302 | ADAM8 | 0.3816 |
| ENSP00000368109 | ACCSL | 0.3816 |
| ENSP00000469958 | ZNF626 | 0.3789 |
| ENSP00000340171 | ZNF573 | 0.3789 |
| ENSP00000458062 | ZNF286A | 0.3789 |
| ENSP00000364220 | VWA5B1 | 0.3789 |
| ENSP00000255304 | USPL1 | 0.3789 |
| ENSP00000372199 | URB1 | 0.3789 |
| ENSP00000356320 | ULBP2 | 0.3789 |
| ENSP00000267938 | UBE2Q2 | 0.3789 |
| ENSP00000292879 | U2AF1L4 | 0.3789 |
| ENSP00000300482 | TRPM2 | 0.3789 |
| ENSP00000326737 | TNFRSF12A | 0.3789 |
| ENSP00000319992 | TMEM11 | 0.3789 |
| ENSP00000216468 | TMED8 | 0.3789 |
| ENSP00000281030 | THRSP | 0.3789 |
| ENSP00000304689 | THAP11 | 0.3789 |

| | | |
|---|---|---|
| ENSP00000324323 | TCTEX1D2 | 0.3789 |
| ENSP00000357794 | TCHH | 0.3789 |
| ENSP00000297239 | SYTL3 | 0.3789 |
| ENSP00000267377 | SSTR1 | 0.3789 |
| ENSP00000457386 | SMIM1 | 0.3789 |
| ENSP00000354107 | SLC6A6 | 0.3789 |
| ENSP00000327943 | SLC5A2 | 0.3789 |
| ENSP00000367950 | SHROOM1 | 0.3789 |
| ENSP00000358853 | SH3BGRL2 | 0.3789 |
| ENSP00000402338 | RPF2 | 0.3789 |
| ENSP00000347548 | RNF220 | 0.3789 |
| ENSP00000217740 | RNF125 | 0.3789 |
| ENSP00000454919 | RNF112 | 0.3789 |
| ENSP00000320623 | RMND5B | 0.3789 |
| ENSP00000381206 | RDH16 | 0.3789 |
| ENSP00000273968 | PYURF | 0.3789 |
| ENSP00000446916 | PRSS58 | 0.3789 |
| ENSP00000327386 | PRSS57 | 0.3789 |
| ENSP00000354280 | PRSS3 | 0.3789 |
| ENSP00000275034 | PHIP | 0.3789 |
| ENSP00000367727 | PANK4 | 0.3789 |
| ENSP00000284292 | NRGN | 0.3789 |
| ENSP00000311196 | NPAS4 | 0.3789 |
| ENSP00000339958 | NISCH | 0.3789 |
| ENSP00000371362 | NADKD1 | 0.3789 |
| ENSP00000306997 | MYOZ2 | 0.3789 |
| ENSP00000359417 | MTMR1 | 0.3789 |
| ENSP00000308901 | MRPL45 | 0.3789 |
| ENSP00000199706 | MRPL28 | 0.3789 |
| ENSP00000331664 | MGAT4C | 0.3789 |
| ENSP00000307077 | METTL18 | 0.3789 |
| ENSP00000370800 | MBD3L2 | 0.3789 |
| ENSP00000355880 | MARC2 | 0.3789 |
| ENSP00000395473 | MAN2B1 | 0.3789 |
| ENSP00000367315 | LRRC10B | 0.3789 |
| ENSP00000344353 | LPAR6 | 0.3789 |
| ENSP00000355302 | KRTAP1-5 | 0.3789 |
| ENSP00000246635 | KRT13 | 0.3789 |
| ENSP00000242810 | KLHL24 | 0.3789 |
| ENSP00000338013 | KIAA1984 | 0.3789 |
| ENSP00000456200 | IST1 | 0.3789 |
| ENSP00000263379 | IL27RA | 0.3789 |
| ENSP00000359583 | HPSE2 | 0.3789 |
| ENSP00000390431 | HLA-DQB2 | 0.3789 |
| ENSP00000284562 | GSTA5 | 0.3789 |
| ENSP00000382791 | GRIK1 | 0.3789 |
| ENSP00000389175 | GLYCTK | 0.3789 |
| ENSP00000309653 | GLIS1 | 0.3789 |
| ENSP00000360060 | FRAT1 | 0.3789 |
| ENSP00000371397 | FAM43A | 0.3789 |
| ENSP00000300255 | EVA1C | 0.3789 |
| ENSP00000375073 | EID2 | 0.3789 |
| ENSP00000216733 | EFS | 0.3789 |

| | | |
|---|---|---|
| ENSP00000345229 | DNER | 0.3789 |
| ENSP00000311401 | DENND6A | 0.3789 |
| ENSP00000358953 | CUEDC2 | 0.3789 |
| ENSP00000310832 | CTSF | 0.3789 |
| ENSP00000457718 | COG8 | 0.3789 |
| ENSP00000352639 | CELA2A | 0.3789 |
| ENSP00000261692 | CDK2AP1 | 0.3789 |
| ENSP00000432034 | CCL15-CCL14 | 0.3789 |
| ENSP00000335544 | CCKBR | 0.3789 |
| ENSP00000366612 | CBWD5 | 0.3789 |
| ENSP00000372955 | C3orf37 | 0.3789 |
| ENSP00000040738 | BOD1L1 | 0.3789 |
| ENSP00000353010 | ATG2B | 0.3789 |
| ENSP00000225823 | ASIC2 | 0.3789 |
| ENSP00000431391 | API5 | 0.3789 |
| ENSP00000377833 | ANXA4 | 0.3789 |
| ENSP00000159087 | ANO8 | 0.3789 |
| ENSP00000292246 | ANO10 | 0.3789 |
| ENSP00000313670 | ALS2CL | 0.3789 |
| ENSP00000229243 | ACRBP | 0.3789 |
| ENSP00000370531 | ACOT6 | 0.3789 |
| ENSP00000294353 | ZYG11B | 0.3763 |
| ENSP00000252463 | ZSCAN10 | 0.3763 |
| ENSP00000311521 | ZNF490 | 0.3763 |
| ENSP00000367069 | ZFAT | 0.3763 |
| ENSP00000370616 | ZBED1 | 0.3763 |
| ENSP00000255305 | XPO4 | 0.3763 |
| ENSP00000341819 | VWC2 | 0.3763 |
| ENSP00000383719 | UBE2J2 | 0.3763 |
| ENSP00000346627 | TYW5 | 0.3763 |
| ENSP00000332152 | TRIM52 | 0.3763 |
| ENSP00000451451 | TNFSF12 | 0.3763 |
| ENSP00000386210 | TAS2R40 | 0.3763 |
| ENSP00000300108 | TAC3 | 0.3763 |
| ENSP00000275216 | TAAR1 | 0.3763 |
| ENSP00000355156 | SUCNR1 | 0.3763 |
| ENSP00000321347 | STRBP | 0.3763 |
| ENSP00000306461 | SPRR1B | 0.3763 |
| ENSP00000315006 | SLC28A2 | 0.3763 |
| ENSP00000342267 | SLC25A15 | 0.3763 |
| ENSP00000266771 | SLC15A4 | 0.3763 |
| ENSP00000362814 | SERINC2 | 0.3763 |
| ENSP00000005386 | RPAP3 | 0.3763 |
| ENSP00000462747 | RP11-343C2.7 | 0.3763 |
| ENSP00000354571 | RNF121 | 0.3763 |
| ENSP00000293677 | RAVER1 | 0.3763 |
| ENSP00000312837 | RAB11FIP4 | 0.3763 |
| ENSP00000269582 | PNMT | 0.3763 |
| ENSP00000369785 | PIR | 0.3763 |
| ENSP00000280350 | PIH1D2 | 0.3763 |
| ENSP00000306670 | NUDT16L1 | 0.3763 |
| ENSP00000290401 | NPTN | 0.3763 |
| ENSP00000375726 | NOSIP | 0.3763 |

| | | |
|---|---|---|
| ENSP00000386134 | NGDN | 0.3763 |
| ENSP00000300131 | NAB2 | 0.3763 |
| ENSP00000354972 | NAA35 | 0.3763 |
| ENSP00000354772 | MVB12B | 0.3763 |
| ENSP00000300209 | METTL21B | 0.3763 |
| ENSP00000363040 | MEGF9 | 0.3763 |
| ENSP00000357624 | MARCKS | 0.3763 |
| ENSP00000433290 | LTB4R2 | 0.3763 |
| ENSP00000367157 | LRRC3C | 0.3763 |
| ENSP00000348043 | LRRC20 | 0.3763 |
| ENSP00000297293 | LMTK2 | 0.3763 |
| ENSP00000221973 | LIM2 | 0.3763 |
| ENSP00000383226 | KRTAP10-1 | 0.3763 |
| ENSP00000257974 | KRT82 | 0.3763 |
| ENSP00000295746 | KIAA1524 | 0.3763 |
| ENSP00000434241 | KCNC3 | 0.3763 |
| ENSP00000360806 | KCNB1 | 0.3763 |
| ENSP00000368797 | KBTBD7 | 0.3763 |
| ENSP00000368799 | KBTBD6 | 0.3763 |
| ENSP00000334003 | INTU | 0.3763 |
| ENSP00000377370 | IGSF11 | 0.3763 |
| ENSP00000319476 | ICOS | 0.3763 |
| ENSP00000388666 | ICAM2 | 0.3763 |
| ENSP00000289753 | HTR6 | 0.3763 |
| ENSP00000301785 | HNRNPUL2 | 0.3763 |
| ENSP00000385479 | HIST2H3A | 0.3763 |
| ENSP00000318085 | HINFP | 0.3763 |
| ENSP00000346450 | H2AFB1 | 0.3763 |
| ENSP00000358851 | GSTM4 | 0.3763 |
| ENSP00000218316 | GPR50 | 0.3763 |
| ENSP00000428824 | GPBAR1 | 0.3763 |
| ENSP00000325775 | GJC3 | 0.3763 |
| ENSP00000359485 | GBP6 | 0.3763 |
| ENSP00000471397 | GBA3 | 0.3763 |
| ENSP00000278919 | FEZ1 | 0.3763 |
| ENSP00000399259 | FCHSD1 | 0.3763 |
| ENSP00000432931 | FAM86B1 | 0.3763 |
| ENSP00000273920 | ENOPH1 | 0.3763 |
| ENSP00000345997 | DMPK | 0.3763 |
| ENSP00000340935 | DMP1 | 0.3763 |
| ENSP00000235180 | DLGAP3 | 0.3763 |
| ENSP00000260803 | DBR1 | 0.3763 |
| ENSP00000265997 | CPEB3 | 0.3763 |
| ENSP00000297770 | CPA6 | 0.3763 |
| ENSP00000264734 | CLDN16 | 0.3763 |
| ENSP00000357311 | CENPW | 0.3763 |
| ENSP00000369325 | CDKL5 | 0.3763 |
| ENSP00000249887 | CCRL1 | 0.3763 |
| ENSP00000300213 | CCNDBP1 | 0.3763 |
| ENSP00000366608 | CBWD7 | 0.3763 |
| ENSP00000323339 | C2CD3 | 0.3763 |
| ENSP00000218147 | BCORL1 | 0.3763 |
| ENSP00000263610 | BARHL1 | 0.3763 |

| | | |
|---|---|---|
| ENSP00000333395 | ARSI | 0.3763 |
| ENSP00000391490 | AGR2 | 0.3763 |
| ENSP00000265727 | ADAM22 | 0.3763 |
| ENSP00000342216 | ABCA9 | 0.3763 |
| ENSP00000365395 | AADACL4 | 0.3763 |
| ENSP00000378326 | ZP3 | 0.3737 |
| ENSP00000314709 | ZHX2 | 0.3737 |
| ENSP00000331933 | WSCD2 | 0.3737 |
| ENSP00000263795 | WDR76 | 0.3737 |
| ENSP00000384302 | WDR43 | 0.3737 |
| ENSP00000333799 | WBSCR16 | 0.3737 |
| ENSP00000362105 | UTP11L | 0.3737 |
| ENSP00000305426 | TUB | 0.3737 |
| ENSP00000388293 | TSTD1 | 0.3737 |
| ENSP00000011898 | TSPAN9 | 0.3737 |
| ENSP00000332668 | TSKU | 0.3737 |
| ENSP00000376930 | TMCC1 | 0.3737 |
| ENSP00000360560 | TCTE1 | 0.3737 |
| ENSP00000247883 | TAS2R5 | 0.3737 |
| ENSP00000297325 | SUN3 | 0.3737 |
| ENSP00000302951 | STRA13 | 0.3737 |
| ENSP00000379353 | ST8SIA1 | 0.3737 |
| ENSP00000345849 | SPAM1 | 0.3737 |
| ENSP00000341682 | SLC26A9 | 0.3737 |
| ENSP00000413049 | SLC25A48 | 0.3737 |
| ENSP00000316909 | SLC17A8 | 0.3737 |
| ENSP00000452776 | SLC12A6 | 0.3737 |
| ENSP00000296043 | SHROOM3 | 0.3737 |
| ENSP00000403476 | SH3D21 | 0.3737 |
| ENSP00000443130 | RP11-286N22.8 | 0.3737 |
| ENSP00000259605 | RNF38 | 0.3737 |
| ENSP00000262482 | RNF167 | 0.3737 |
| ENSP00000425840 | RNF150 | 0.3737 |
| ENSP00000367202 | RECK | 0.3737 |
| ENSP00000245796 | PSD4 | 0.3737 |
| ENSP00000357452 | PMVK | 0.3737 |
| ENSP00000406933 | NT5DC2 | 0.3737 |
| ENSP00000360530 | NSMF | 0.3737 |
| ENSP00000366934 | NOL9 | 0.3737 |
| ENSP00000299964 | NNMT | 0.3737 |
| ENSP00000373411 | NKIRAS1 | 0.3737 |
| ENSP00000296849 | NKD2 | 0.3737 |
| ENSP00000280700 | NGLY1 | 0.3737 |
| ENSP00000424757 | NEK1 | 0.3737 |
| ENSP00000381008 | MUC16 | 0.3737 |
| ENSP00000338487 | MGAT4B | 0.3737 |
| ENSP00000218439 | MAGED2 | 0.3737 |
| ENSP00000356846 | MAEL | 0.3737 |
| ENSP00000309689 | LRCH4 | 0.3737 |
| ENSP00000375683 | KLK7 | 0.3737 |
| ENSP00000332791 | KLHL15 | 0.3737 |
| ENSP00000345055 | KCNQ5 | 0.3737 |
| ENSP00000361952 | KCNK15 | 0.3737 |

| | | |
|---|---|---|
| ENSP00000334714 | IGFN1 | 0.3737 |
| ENSP00000353915 | HTR4 | 0.3737 |
| ENSP00000390354 | HS3ST6 | 0.3737 |
| ENSP00000427888 | HS3ST5 | 0.3737 |
| ENSP00000365817 | HLA-E | 0.3737 |
| ENSP00000308733 | GPR151 | 0.3737 |
| ENSP00000274093 | GLRA3 | 0.3737 |
| ENSP00000324292 | FBF1 | 0.3737 |
| ENSP00000377225 | FAM50A | 0.3737 |
| ENSP00000374565 | FAM168B | 0.3737 |
| ENSP00000261488 | ENOX1 | 0.3737 |
| ENSP00000270221 | EMP3 | 0.3737 |
| ENSP00000370889 | DENND1C | 0.3737 |
| ENSP00000332679 | CYP2A13 | 0.3737 |
| ENSP00000226432 | CWH43 | 0.3737 |
| ENSP00000354294 | CTRB1 | 0.3737 |
| ENSP00000333833 | CMTM4 | 0.3737 |
| ENSP00000317404 | CHST13 | 0.3737 |
| ENSP00000293780 | CHRNE | 0.3737 |
| ENSP00000338369 | CELA3B | 0.3737 |
| ENSP00000353942 | CCDC135 | 0.3737 |
| ENSP00000366677 | CBWD6 | 0.3737 |
| ENSP00000279101 | CABLES2 | 0.3737 |
| ENSP00000400815 | C16orf62 | 0.3737 |
| ENSP00000411672 | ATP6V0E2 | 0.3737 |
| ENSP00000370522 | ARSH | 0.3737 |
| ENSP00000352101 | ANXA8L1 | 0.3737 |
| ENSP00000314048 | ANO2 | 0.3737 |
| ENSP00000280979 | AKAP6 | 0.3737 |
| ENSP00000229583 | ADTRP | 0.3737 |
| ENSP00000352268 | AADACL3 | 0.3737 |
| ENSP00000348911 | AADACL2 | 0.3737 |
| ENSP00000368732 | DGAT2L7P | 0.3737 |
| ENSP00000466795 | ENSG00000267149 | 0.3737 |
| ENSP00000276123 | ZNF711 | 0.3711 |
| ENSP00000394757 | ZNF516 | 0.3711 |
| ENSP00000371003 | ZMYND11 | 0.3711 |
| ENSP00000267294 | ZIC5 | 0.3711 |
| ENSP00000270001 | ZFP14 | 0.3711 |
| ENSP00000430497 | ZFHX4 | 0.3711 |
| ENSP00000318222 | ZDHHC22 | 0.3711 |
| ENSP00000404403 | ZBTB22 | 0.3711 |
| ENSP00000263381 | WIZ | 0.3711 |
| ENSP00000385143 | UNC5D | 0.3711 |
| ENSP00000155858 | TRPM5 | 0.3711 |
| ENSP00000257909 | TROAP | 0.3711 |
| ENSP00000285419 | TMEM55A | 0.3711 |
| ENSP00000214869 | TMED1 | 0.3711 |
| ENSP00000305852 | TM4SF4 | 0.3711 |
| ENSP00000262319 | TELO2 | 0.3711 |
| ENSP00000257264 | TCN1 | 0.3711 |
| ENSP00000310008 | SWSAP1 | 0.3711 |
| ENSP00000361092 | SURF6 | 0.3711 |

| | | |
|---|---|---|
| ENSP00000169298 | ST6GAL1 | 0.3711 |
| ENSP00000373454 | SRRM3 | 0.3711 |
| ENSP00000361087 | SNCG | 0.3711 |
| ENSP00000369399 | SLC4A11 | 0.3711 |
| ENSP00000322020 | SLC25A22 | 0.3711 |
| ENSP00000327569 | SLC22A10 | 0.3711 |
| ENSP00000245312 | SLC10A2 | 0.3711 |
| ENSP00000210633 | SEMA4G | 0.3711 |
| ENSP00000176195 | SCT | 0.3711 |
| ENSP00000388550 | RNF24 | 0.3711 |
| ENSP00000206262 | RGS17 | 0.3711 |
| ENSP00000344226 | RASSF7 | 0.3711 |
| ENSP00000336616 | RASSF3 | 0.3711 |
| ENSP00000336931 | PNRC1 | 0.3711 |
| ENSP00000266671 | PHLDA1 | 0.3711 |
| ENSP00000227638 | PANX1 | 0.3711 |
| ENSP00000171757 | P2RY10 | 0.3711 |
| ENSP00000359121 | OLFM3 | 0.3711 |
| ENSP00000303686 | NTSR2 | 0.3711 |
| ENSP00000411012 | NSMAF | 0.3711 |
| ENSP00000389252 | NPNT | 0.3711 |
| ENSP00000381486 | NOTO | 0.3711 |
| ENSP00000275820 | NOM1 | 0.3711 |
| ENSP00000262113 | MYOM2 | 0.3711 |
| ENSP00000349298 | MYLIP | 0.3711 |
| ENSP00000312235 | MUC13 | 0.3711 |
| ENSP00000323527 | MKLN1 | 0.3711 |
| ENSP00000362144 | MCU | 0.3711 |
| ENSP00000405268 | MBD1 | 0.3711 |
| ENSP00000342188 | LRRIQ4 | 0.3711 |
| ENSP00000405987 | LRRD1 | 0.3711 |
| ENSP00000372959 | LRRC30 | 0.3711 |
| ENSP00000355166 | LRRC10 | 0.3711 |
| ENSP00000428281 | LEPROTL1 | 0.3711 |
| ENSP00000335565 | KRTAP10-8 | 0.3711 |
| ENSP00000391457 | INO80C | 0.3711 |
| ENSP00000370105 | GTF2H2C | 0.3711 |
| ENSP00000381129 | GRK4 | 0.3711 |
| ENSP00000329684 | GALR2 | 0.3711 |
| ENSP00000296137 | FYCO1 | 0.3711 |
| ENSP00000289166 | FAM46B | 0.3711 |
| ENSP00000274024 | FABP2 | 0.3711 |
| ENSP00000382086 | EVPLL | 0.3711 |
| ENSP00000344758 | ECM2 | 0.3711 |
| ENSP00000357637 | EBF3 | 0.3711 |
| ENSP00000419599 | DPH3 | 0.3711 |
| ENSP00000411010 | DNTTIP2 | 0.3711 |
| ENSP00000284476 | DISP1 | 0.3711 |
| ENSP00000327104 | DDHD1 | 0.3711 |
| ENSP00000357779 | CRCT1 | 0.3711 |
| ENSP00000313854 | CLPTM1L | 0.3711 |
| ENSP00000398017 | CLDN9 | 0.3711 |
| ENSP00000307297 | CHST14 | 0.3711 |

| | | |
|---|---|---|
| ENSP00000244336 | CEACAM8 | 0.3711 |
| ENSP00000379176 | CDKL1 | 0.3711 |
| ENSP00000386378 | CD207 | 0.3711 |
| ENSP00000434724 | CCDC90B | 0.3711 |
| ENSP00000282611 | CATSPER3 | 0.3711 |
| ENSP00000341213 | C20orf24 | 0.3711 |
| ENSP00000266383 | B4GALNT3 | 0.3711 |
| ENSP00000317674 | APOL1 | 0.3711 |
| ENSP00000345767 | ANKRD6 | 0.3711 |
| ENSP00000391950 | ANKRD36 | 0.3711 |
| ENSP00000314879 | ALOXE3 | 0.3711 |
| ENSP00000369858 | ALOX5AP | 0.3711 |
| ENSP00000292069 | ZNF667 | 0.3684 |
| ENSP00000296091 | ZNF502 | 0.3684 |
| ENSP00000339767 | ZNF433 | 0.3684 |
| ENSP00000356562 | XPR1 | 0.3684 |
| ENSP00000363746 | WDR46 | 0.3684 |
| ENSP00000425375 | USP17L24 | 0.3684 |
| ENSP00000424077 | USP17L23 | 0.3684 |
| ENSP00000423115 | USP17L22 | 0.3684 |
| ENSP00000262376 | UBN1 | 0.3684 |
| ENSP00000317327 | UBASH3A | 0.3684 |
| ENSP00000361229 | TSPAN14 | 0.3684 |
| ENSP00000312675 | TRIM72 | 0.3684 |
| ENSP00000300747 | TRIM68 | 0.3684 |
| ENSP00000384876 | TRIM66 | 0.3684 |
| ENSP00000321203 | TNIP2 | 0.3684 |
| ENSP00000406885 | TMEM41A | 0.3684 |
| ENSP00000266025 | TMEM115 | 0.3684 |
| ENSP00000231198 | THG1L | 0.3684 |
| ENSP00000328232 | TCEB3C | 0.3684 |
| ENSP00000273980 | TBCK | 0.3684 |
| ENSP00000225525 | TAX1BP3 | 0.3684 |
| ENSP00000324302 | STXBP6 | 0.3684 |
| ENSP00000261866 | SPG11 | 0.3684 |
| ENSP00000355721 | SNAP47 | 0.3684 |
| ENSP00000366586 | SLC39A12 | 0.3684 |
| ENSP00000365413 | SLC28A3 | 0.3684 |
| ENSP00000381782 | SLC25A45 | 0.3684 |
| ENSP00000307443 | SLC22A25 | 0.3684 |
| ENSP00000221742 | SLC1A6 | 0.3684 |
| ENSP00000392411 | SLC13A2 | 0.3684 |
| ENSP00000357036 | SLAMF6 | 0.3684 |
| ENSP00000265195 | SIL1 | 0.3684 |
| ENSP00000455510 | SIGLEC5 | 0.3684 |
| ENSP00000270792 | SH3BGRL3 | 0.3684 |
| ENSP00000224807 | SFXN3 | 0.3684 |
| ENSP00000348596 | SDAD1 | 0.3684 |
| ENSP00000450554 | RP11-1012A1.4 | 0.3684 |
| ENSP00000281722 | RBM46 | 0.3684 |
| ENSP00000354608 | PPT2 | 0.3684 |
| ENSP00000299492 | PPFIBP2 | 0.3684 |
| ENSP00000296333 | PIGX | 0.3684 |

| | | |
|---|---|---|
| ENSP00000216452 | PIGH | 0.3684 |
| ENSP00000322532 | PELI3 | 0.3684 |
| ENSP00000362186 | PCDH11X | 0.3684 |
| ENSP00000363414 | PAQR7 | 0.3684 |
| ENSP00000381049 | NSG1 | 0.3684 |
| ENSP00000367752 | NRSN1 | 0.3684 |
| ENSP00000233840 | NEU2 | 0.3684 |
| ENSP00000366326 | NEBL | 0.3684 |
| ENSP00000324810 | MVB12A | 0.3684 |
| ENSP00000264198 | MUL1 | 0.3684 |
| ENSP00000384651 | MTMR3 | 0.3684 |
| ENSP00000337816 | MMP25 | 0.3684 |
| ENSP00000260228 | MMP20 | 0.3684 |
| ENSP00000331787 | METTL7A | 0.3684 |
| ENSP00000314196 | MBOAT4 | 0.3684 |
| ENSP00000318916 | MARCO | 0.3684 |
| ENSP00000354660 | MAGEC2 | 0.3684 |
| ENSP00000225972 | LRRC59 | 0.3684 |
| ENSP00000360104 | LEPROT | 0.3684 |
| ENSP00000252245 | KRT75 | 0.3684 |
| ENSP00000341549 | KLHL33 | 0.3684 |
| ENSP00000359392 | KHDC3L | 0.3684 |
| ENSP00000398410 | KDM8 | 0.3684 |
| ENSP00000358802 | KCNC4 | 0.3684 |
| ENSP00000221444 | KCNA7 | 0.3684 |
| ENSP00000415436 | KANSL2 | 0.3684 |
| ENSP00000360195 | KANK4 | 0.3684 |
| ENSP00000365351 | ITGBL1 | 0.3684 |
| ENSP00000173527 | ISOC1 | 0.3684 |
| ENSP00000310111 | HSPB7 | 0.3684 |
| ENSP00000363794 | HSD17B8 | 0.3684 |
| ENSP00000329554 | HIST1H3I | 0.3684 |
| ENSP00000264553 | GZMM | 0.3684 |
| ENSP00000231009 | GZMK | 0.3684 |
| ENSP00000386772 | GPATCH11 | 0.3684 |
| ENSP00000359795 | GIPC2 | 0.3684 |
| ENSP00000359329 | GABRQ | 0.3684 |
| ENSP00000260282 | FXYD6 | 0.3684 |
| ENSP00000283309 | FRMD1 | 0.3684 |
| ENSP00000358391 | FCGR1B | 0.3684 |
| ENSP00000352182 | FAM86C1 | 0.3684 |
| ENSP00000372112 | FAHD1 | 0.3684 |
| ENSP00000355028 | DZIP3 | 0.3684 |
| ENSP00000308695 | DPY19L1 | 0.3684 |
| ENSP00000260247 | DCUN1D5 | 0.3684 |
| ENSP00000215939 | CRYBB1 | 0.3684 |
| ENSP00000382356 | COL28A1 | 0.3684 |
| ENSP00000254868 | CLEC10A | 0.3684 |
| ENSP00000299339 | CLDN10 | 0.3684 |
| ENSP00000304290 | CHRNB1 | 0.3684 |
| ENSP00000356198 | CHIT1 | 0.3684 |
| ENSP00000368244 | CHGB | 0.3684 |
| ENSP00000410815 | CFB | 0.3684 |

| | | |
|---|---|---|
| ENSP00000351684 | CAMSAP2 | 0.3684 |
| ENSP00000406598 | C4orf27 | 0.3684 |
| ENSP00000270233 | BCAM | 0.3684 |
| ENSP00000366748 | AUP1 | 0.3684 |
| ENSP00000228468 | ASIC1 | 0.3684 |
| ENSP00000369346 | ARSK | 0.3684 |
| ENSP00000398637 | ARL13A | 0.3684 |
| ENSP00000365081 | APOM | 0.3684 |
| ENSP00000338260 | APOL4 | 0.3684 |
| ENSP00000229304 | APOBEC1 | 0.3684 |
| ENSP00000305464 | APLN | 0.3684 |
| ENSP00000225410 | ZNHIT3 | 0.3658 |
| ENSP00000411084 | ZNF799 | 0.3658 |
| ENSP00000325326 | ZNF335 | 0.3658 |
| ENSP00000343443 | ZNF107 | 0.3658 |
| ENSP00000351052 | ZC3HC1 | 0.3658 |
| ENSP00000242351 | ZC3HAV1 | 0.3658 |
| ENSP00000238831 | YIPF4 | 0.3658 |
| ENSP00000384792 | WDR62 | 0.3658 |
| ENSP00000346986 | WAC | 0.3658 |
| ENSP00000363506 | UQCC | 0.3658 |
| ENSP00000284551 | TRIM11 | 0.3658 |
| ENSP00000373979 | TRAPPC9 | 0.3658 |
| ENSP00000439594 | THEMIS | 0.3658 |
| ENSP00000337353 | TDP1 | 0.3658 |
| ENSP00000348813 | TBC1D7 | 0.3658 |
| ENSP00000263233 | SYP | 0.3658 |
| ENSP00000336775 | SYNM | 0.3658 |
| ENSP00000319622 | SULT1C2 | 0.3658 |
| ENSP00000262915 | ST3GAL3 | 0.3658 |
| ENSP00000204566 | SPG21 | 0.3658 |
| ENSP00000378418 | SP9 | 0.3658 |
| ENSP00000219833 | SLC6A2 | 0.3658 |
| ENSP00000457733 | SLC25A24 | 0.3658 |
| ENSP00000343248 | SBK1 | 0.3658 |
| ENSP00000369292 | SAMD9 | 0.3658 |
| ENSP00000361238 | RSPH9 | 0.3658 |
| ENSP00000325941 | RIC8A | 0.3658 |
| ENSP00000377075 | RGL3 | 0.3658 |
| ENSP00000345144 | RCBTB2 | 0.3658 |
| ENSP00000296859 | RAPGEF6 | 0.3658 |
| ENSP00000281243 | QDPR | 0.3658 |
| ENSP00000326598 | PRSS50 | 0.3658 |
| ENSP00000295619 | PROK2 | 0.3658 |
| ENSP00000313070 | PPIP5K2 | 0.3658 |
| ENSP00000322304 | PORCN | 0.3658 |
| ENSP00000262043 | PHF3 | 0.3658 |
| ENSP00000313377 | PANK2 | 0.3658 |
| ENSP00000448689 | MAP3K12 | 0.3658 |
| ENSP00000276344 | MAGEA4 | 0.3658 |
| ENSP00000328737 | LYPD4 | 0.3658 |
| ENSP00000414670 | LMOD3 | 0.3658 |
| ENSP00000261893 | LACTB | 0.3658 |

| | | |
|---|---|---|
| ENSP00000366955 | KRTAP4-2 | 0.3658 |
| ENSP00000418018 | IFNE | 0.3658 |
| ENSP00000002596 | HS3ST1 | 0.3658 |
| ENSP00000334876 | GRK1 | 0.3658 |
| ENSP00000218075 | GLRA2 | 0.3658 |
| ENSP00000295454 | GABRB1 | 0.3658 |
| ENSP00000344254 | FXYD5 | 0.3658 |
| ENSP00000360262 | FGGY | 0.3658 |
| ENSP00000264658 | FBXL20 | 0.3658 |
| ENSP00000346874 | FAR1 | 0.3658 |
| ENSP00000282633 | FAM21A | 0.3658 |
| ENSP00000329735 | FAM212A | 0.3658 |
| ENSP00000284486 | FAM167A | 0.3658 |
| ENSP00000263713 | EPB41L5 | 0.3658 |
| ENSP00000359520 | ENTPD7 | 0.3658 |
| ENSP00000344937 | DPY19L3 | 0.3658 |
| ENSP00000230431 | DNPH1 | 0.3658 |
| ENSP00000357905 | DMBT1 | 0.3658 |
| ENSP00000310891 | CSGALNACT1 | 0.3658 |
| ENSP00000305725 | CHST11 | 0.3658 |
| ENSP00000304782 | CES3 | 0.3658 |
| ENSP00000430897 | CCDC71L | 0.3658 |
| ENSP00000425493 | CAGE1 | 0.3658 |
| ENSP00000424363 | BBS5 | 0.3658 |
| ENSP00000312326 | AOC3 | 0.3658 |
| ENSP00000295453 | ALPPL2 | 0.3658 |
| ENSP00000378161 | ACYP2 | 0.3658 |
| ENSP00000355529 | ZP4 | 0.3632 |
| ENSP00000345479 | ZNF587 | 0.3632 |
| ENSP00000347648 | ZNF512 | 0.3632 |
| ENSP00000359740 | ZNF451 | 0.3632 |
| ENSP00000407262 | ZNF138 | 0.3632 |
| ENSP00000344162 | ZNF136 | 0.3632 |
| ENSP00000332013 | ZGPAT | 0.3632 |
| ENSP00000291900 | ZER1 | 0.3632 |
| ENSP00000328326 | XKR4 | 0.3632 |
| ENSP00000295121 | WDR92 | 0.3632 |
| ENSP00000398688 | VWA5B2 | 0.3632 |
| ENSP00000323516 | UTS2R | 0.3632 |
| ENSP00000423777 | USP17L28 | 0.3632 |
| ENSP00000296099 | UCN | 0.3632 |
| ENSP00000324318 | TRAPPC12 | 0.3632 |
| ENSP00000381856 | TOMM6 | 0.3632 |
| ENSP00000335094 | TMEM17 | 0.3632 |
| ENSP00000335595 | TCP11L1 | 0.3632 |
| ENSP00000455444 | TCF24 | 0.3632 |
| ENSP00000295367 | SPRR3 | 0.3632 |
| ENSP00000369887 | SLTM | 0.3632 |
| ENSP00000297524 | SLC7A13 | 0.3632 |
| ENSP00000268099 | SCAMP2 | 0.3632 |
| ENSP00000338556 | RSPH10B | 0.3632 |
| ENSP00000258098 | RAB11FIP5 | 0.3632 |
| ENSP00000386385 | PXMP4 | 0.3632 |

| | | |
|---|---|---|
| ENSP00000368666 | PTCHD1 | 0.3632 |
| ENSP00000413405 | PIGZ | 0.3632 |
| ENSP00000330658 | PAPPA | 0.3632 |
| ENSP00000337862 | OVOL1 | 0.3632 |
| ENSP00000252854 | OLFM1 | 0.3632 |
| ENSP00000270235 | NTN5 | 0.3632 |
| ENSP00000358272 | NDUFAF4 | 0.3632 |
| ENSP00000320886 | MLXIPL | 0.3632 |
| ENSP00000267984 | MESDC1 | 0.3632 |
| ENSP00000286307 | LSM11 | 0.3632 |
| ENSP00000367888 | LRP4 | 0.3632 |
| ENSP00000346442 | LRMP | 0.3632 |
| ENSP00000362398 | LPAR4 | 0.3632 |
| ENSP00000373783 | LOXL2 | 0.3632 |
| ENSP00000411932 | LMOD2 | 0.3632 |
| ENSP00000316881 | LEPREL1 | 0.3632 |
| ENSP00000371595 | KRTAP5-6 | 0.3632 |
| ENSP00000383223 | KRTAP10-5 | 0.3632 |
| ENSP00000373648 | KCNQ3 | 0.3632 |
| ENSP00000430846 | KCNB2 | 0.3632 |
| ENSP00000375691 | JOSD2 | 0.3632 |
| ENSP00000369962 | IGSF5 | 0.3632 |
| ENSP00000359940 | IGSF1 | 0.3632 |
| ENSP00000390020 | HLA-DOB | 0.3632 |
| ENSP00000241337 | GSTM2 | 0.3632 |
| ENSP00000245983 | GNRH2 | 0.3632 |
| ENSP00000223528 | FKTN | 0.3632 |
| ENSP00000360871 | FCN1 | 0.3632 |
| ENSP00000338050 | DTX3 | 0.3632 |
| ENSP00000445366 | DND1 | 0.3632 |
| ENSP00000352928 | DDA1 | 0.3632 |
| ENSP00000215855 | CRYBB3 | 0.3632 |
| ENSP00000238892 | CRIPT | 0.3632 |
| ENSP00000339157 | CPM | 0.3632 |
| ENSP00000261340 | CLEC2D | 0.3632 |
| ENSP00000332771 | CLCNKA | 0.3632 |
| ENSP00000351118 | CDHR5 | 0.3632 |
| ENSP00000308117 | CD248 | 0.3632 |
| ENSP00000216029 | CBY1 | 0.3632 |
| ENSP00000371037 | CATSPERD | 0.3632 |
| ENSP00000333283 | C17orf70 | 0.3632 |
| ENSP00000338990 | C12orf44 | 0.3632 |
| ENSP00000355195 | ASB12 | 0.3632 |
| ENSP00000468236 | APOC4 | 0.3632 |
| ENSP00000215941 | ANKRD54 | 0.3632 |
| ENSP00000267116 | ANKRD52 | 0.3632 |
| ENSP00000227618 | ANAPC15 | 0.3632 |
| ENSP00000336693 | ABHD12B | 0.3632 |
| ENSP00000270459 | ZNF787 | 0.3605 |
| ENSP00000252797 | ZNF764 | 0.3605 |
| ENSP00000294753 | ZNF496 | 0.3605 |
| ENSP00000277225 | ZNF462 | 0.3605 |
| ENSP00000276816 | ZNF16 | 0.3605 |

| | | |
|---|---|---|
| ENSP00000292176 | ZBTB7B | 0.3605 |
| ENSP00000072644 | YIPF1 | 0.3605 |
| ENSP00000288912 | WDR66 | 0.3605 |
| ENSP00000234392 | VAX2 | 0.3605 |
| ENSP00000333376 | USP45 | 0.3605 |
| ENSP00000422969 | USP17L29 | 0.3605 |
| ENSP00000423211 | USP17L27 | 0.3605 |
| ENSP00000427366 | USP17L26 | 0.3605 |
| ENSP00000422097 | USP17L25 | 0.3605 |
| ENSP00000441024 | UBAP1 | 0.3605 |
| ENSP00000007390 | TSR3 | 0.3605 |
| ENSP00000323926 | TRPM8 | 0.3605 |
| ENSP00000381184 | TMEM231 | 0.3605 |
| ENSP00000379531 | TMC5 | 0.3605 |
| ENSP00000362332 | TFAP2E | 0.3605 |
| ENSP00000403954 | TENM1 | 0.3605 |
| ENSP00000404923 | TECPR1 | 0.3605 |
| ENSP00000377700 | TBC1D23 | 0.3605 |
| ENSP00000228567 | SYT10 | 0.3605 |
| ENSP00000377789 | SYNPO | 0.3605 |
| ENSP00000337446 | STARD3 | 0.3605 |
| ENSP00000227495 | ST3GAL4 | 0.3605 |
| ENSP00000299084 | SPRED1 | 0.3605 |
| ENSP00000317332 | SNX18 | 0.3605 |
| ENSP00000269187 | SLC39A6 | 0.3605 |
| ENSP00000301305 | SLC39A4 | 0.3605 |
| ENSP00000313740 | SLC38A5 | 0.3605 |
| ENSP00000355353 | SLC34A3 | 0.3605 |
| ENSP00000454014 | SHISA9 | 0.3605 |
| ENSP00000332204 | SEMA4B | 0.3605 |
| ENSP00000386796 | SCN7A | 0.3605 |
| ENSP00000355387 | SCAMP5 | 0.3605 |
| ENSP00000362555 | RNF19B | 0.3605 |
| ENSP00000391564 | RIMBP3 | 0.3605 |
| ENSP00000444196 | RIBC2 | 0.3605 |
| ENSP00000326170 | RFFL | 0.3605 |
| ENSP00000261336 | PZP | 0.3605 |
| ENSP00000217270 | PROKR2 | 0.3605 |
| ENSP00000358232 | PNLIPRP3 | 0.3605 |
| ENSP00000425556 | PALLD | 0.3605 |
| ENSP00000367462 | OLAH | 0.3605 |
| ENSP00000380450 | NOXO1 | 0.3605 |
| ENSP00000337618 | NIPA2 | 0.3605 |
| ENSP00000348394 | NCDN | 0.3605 |
| ENSP00000362869 | MORN5 | 0.3605 |
| ENSP00000215862 | MORC2 | 0.3605 |
| ENSP00000370611 | MIER3 | 0.3605 |
| ENSP00000281923 | MGAT5 | 0.3605 |
| ENSP00000257724 | MDFIC | 0.3605 |
| ENSP00000264079 | MCOLN1 | 0.3605 |
| ENSP00000360645 | MAN1B1 | 0.3605 |
| ENSP00000354912 | MAGEE1 | 0.3605 |
| ENSP00000363638 | LYPLA2 | 0.3605 |

| | | |
|---|---|---|
| ENSP00000414635 | LRRC34 | 0.3605 |
| ENSP00000359557 | LDOC1 | 0.3605 |
| ENSP00000360411 | LDLRAD1 | 0.3605 |
| ENSP00000385597 | IQCE | 0.3605 |
| ENSP00000363046 | IPMK | 0.3605 |
| ENSP00000412457 | HLA-DMB | 0.3605 |
| ENSP00000346509 | H2AFB2 | 0.3605 |
| ENSP00000315070 | GJD4 | 0.3605 |
| ENSP00000260118 | GGH | 0.3605 |
| ENSP00000269195 | GALNT1 | 0.3605 |
| ENSP00000343314 | FXYD1 | 0.3605 |
| ENSP00000302599 | FUT9 | 0.3605 |
| ENSP00000357594 | FAM26F | 0.3605 |
| ENSP00000247270 | EVI2A | 0.3605 |
| ENSP00000264059 | EFHD1 | 0.3605 |
| ENSP00000360107 | EFHC1 | 0.3605 |
| ENSP00000295824 | EFHB | 0.3605 |
| ENSP00000320813 | DEFB104A | 0.3605 |
| ENSP00000303963 | CTRB2 | 0.3605 |
| ENSP00000326052 | COX14 | 0.3605 |
| ENSP00000363596 | CNR2 | 0.3605 |
| ENSP00000296387 | CLDN19 | 0.3605 |
| ENSP00000290913 | CHCHD6 | 0.3605 |
| ENSP00000304250 | CDK5R2 | 0.3605 |
| ENSP00000301458 | CD320 | 0.3605 |
| ENSP00000316237 | CCDC102B | 0.3605 |
| ENSP00000366221 | CASZ1 | 0.3605 |
| ENSP00000367808 | C1orf86 | 0.3605 |
| ENSP00000228136 | C11orf58 | 0.3605 |
| ENSP00000326340 | ATG16L2 | 0.3605 |
| ENSP00000276569 | ARMC1 | 0.3605 |
| ENSP00000272217 | ARL8A | 0.3605 |
| ENSP00000367355 | ARHGEF35 | 0.3605 |
| ENSP00000347454 | ANO1 | 0.3605 |
| ENSP00000449629 | ANKS1B | 0.3605 |
| ENSP00000257787 | AKIRIN2 | 0.3605 |
| ENSP00000378430 | TMSB4XP4 | 0.3605 |
| ENSP00000240499 | ZNF141 | 0.3579 |
| ENSP00000231749 | ZMYND10 | 0.3579 |
| ENSP00000261683 | ZBTB25 | 0.3579 |
| ENSP00000017003 | XYLT2 | 0.3579 |
| ENSP00000311184 | WFIKKN2 | 0.3579 |
| ENSP00000363869 | VSIG4 | 0.3579 |
| ENSP00000373873 | UNKL | 0.3579 |
| ENSP00000341045 | UGT2B15 | 0.3579 |
| ENSP00000251566 | UGT2A3 | 0.3579 |
| ENSP00000418648 | UBN2 | 0.3579 |
| ENSP00000419279 | TTC26 | 0.3579 |
| ENSP00000369440 | TRIM6 | 0.3579 |
| ENSP00000290942 | TPPP3 | 0.3579 |
| ENSP00000386292 | TMEM194B | 0.3579 |
| ENSP00000327724 | TAS2R60 | 0.3579 |
| ENSP00000327124 | SMCR7L | 0.3579 |

| | | |
|---|---|---|
| ENSP00000405812 | SLC4A8 | 0.3579 |
| ENSP00000386272 | SLC38A11 | 0.3579 |
| ENSP00000222345 | SIPA1L3 | 0.3579 |
| ENSP00000333656 | SIGIRR | 0.3579 |
| ENSP00000268231 | SEPT12 | 0.3579 |
| ENSP00000357861 | SELENBP1 | 0.3579 |
| ENSP00000456615 | RP11-697E2.6 | 0.3579 |
| ENSP00000361857 | RLF | 0.3579 |
| ENSP00000354884 | RASSF9 | 0.3579 |
| ENSP00000409279 | OTUD4 | 0.3579 |
| ENSP00000264833 | OLFM2 | 0.3579 |
| ENSP00000285402 | ODF1 | 0.3579 |
| ENSP00000326858 | NT5DC1 | 0.3579 |
| ENSP00000267425 | NOP9 | 0.3579 |
| ENSP00000292431 | NACC1 | 0.3579 |
| ENSP00000416753 | MUC15 | 0.3579 |
| ENSP00000385610 | MEX3C | 0.3579 |
| ENSP00000314901 | LRRN1 | 0.3579 |
| ENSP00000260061 | LRRC32 | 0.3579 |
| ENSP00000360597 | LRRC26 | 0.3579 |
| ENSP00000334644 | LCE3C | 0.3579 |
| ENSP00000349923 | LAGE3 | 0.3579 |
| ENSP00000320936 | IL17RA | 0.3579 |
| ENSP00000229829 | HLA-DOA | 0.3579 |
| ENSP00000369018 | GPR56 | 0.3579 |
| ENSP00000220940 | GML | 0.3579 |
| ENSP00000315835 | GALNT11 | 0.3579 |
| ENSP00000412673 | GABRR1 | 0.3579 |
| ENSP00000023897 | GABRA1 | 0.3579 |
| ENSP00000252675 | FUT5 | 0.3579 |
| ENSP00000367284 | FUNDC1 | 0.3579 |
| ENSP00000270509 | FBN3 | 0.3579 |
| ENSP00000318298 | FAM46A | 0.3579 |
| ENSP00000422131 | FAM193B | 0.3579 |
| ENSP00000250263 | ERI1 | 0.3579 |
| ENSP00000272444 | DUSP11 | 0.3579 |
| ENSP00000315659 | DNMBP | 0.3579 |
| ENSP00000293371 | DCD | 0.3579 |
| ENSP00000369592 | CCDC85C | 0.3579 |
| ENSP00000380679 | CCDC40 | 0.3579 |
| ENSP00000219299 | CCDC113 | 0.3579 |
| ENSP00000285381 | CA3 | 0.3579 |
| ENSP00000328277 | B4GALNT4 | 0.3579 |
| ENSP00000309096 | B3GNT1 | 0.3579 |
| ENSP00000274361 | ANKRD31 | 0.3579 |
| ENSP00000296721 | AFAP1L1 | 0.3579 |
| ENSP00000366527 | ZSCAN16 | 0.3553 |
| ENSP00000337475 | ZDHHC4 | 0.3553 |
| ENSP00000266529 | ZCRB1 | 0.3553 |
| ENSP00000356375 | ZBTB41 | 0.3553 |
| ENSP00000219454 | WFDC1 | 0.3553 |
| ENSP00000422887 | USP17L30 | 0.3553 |
| ENSP00000422216 | USP17L21 | 0.3553 |

| | | |
|---|---|---|
| ENSP00000427264 | USP17L20 | 0.3553 |
| ENSP00000422621 | USP17L17 | 0.3553 |
| ENSP00000229708 | ULBP1 | 0.3553 |
| ENSP00000364293 | TSR2 | 0.3553 |
| ENSP00000222747 | TSPAN12 | 0.3553 |
| ENSP00000297994 | TRMT10B | 0.3553 |
| ENSP00000326063 | TMEM39A | 0.3553 |
| ENSP00000240361 | TEX14 | 0.3553 |
| ENSP00000341346 | TEKT1 | 0.3553 |
| ENSP00000352510 | TECPR2 | 0.3553 |
| ENSP00000263635 | TANC1 | 0.3553 |
| ENSP00000356848 | TADA1 | 0.3553 |
| ENSP00000360269 | TACSTD2 | 0.3553 |
| ENSP00000296632 | STARD4 | 0.3553 |
| ENSP00000255008 | SSTR4 | 0.3553 |
| ENSP00000397911 | SP140L | 0.3553 |
| ENSP00000326603 | SMCHD1 | 0.3553 |
| ENSP00000326693 | SLC25A42 | 0.3553 |
| ENSP00000297282 | SLC13A4 | 0.3553 |
| ENSP00000231721 | SEMA3G | 0.3553 |
| ENSP00000371800 | RNF215 | 0.3553 |
| ENSP00000005995 | PRSS21 | 0.3553 |
| ENSP00000412064 | PRR13 | 0.3553 |
| ENSP00000365570 | PRAF2 | 0.3553 |
| ENSP00000290294 | PRAC | 0.3553 |
| ENSP00000339613 | POFUT2 | 0.3553 |
| ENSP00000347560 | PLEKHA5 | 0.3553 |
| ENSP00000322579 | PHF23 | 0.3553 |
| ENSP00000253812 | PCDHGA3 | 0.3553 |
| ENSP00000295290 | PACRGL | 0.3553 |
| ENSP00000272223 | OSR1 | 0.3553 |
| ENSP00000355512 | OPN3 | 0.3553 |
| ENSP00000325868 | ODF3 | 0.3553 |
| ENSP00000302648 | NRTN | 0.3553 |
| ENSP00000232501 | NPRL2 | 0.3553 |
| ENSP00000289547 | NPC1L1 | 0.3553 |
| ENSP00000277554 | NACC2 | 0.3553 |
| ENSP00000338389 | MRPL35 | 0.3553 |
| ENSP00000367792 | MORN1 | 0.3553 |
| ENSP00000311888 | MGAT1 | 0.3553 |
| ENSP00000314543 | MAGEB18 | 0.3553 |
| ENSP00000355895 | LYPLAL1 | 0.3553 |
| ENSP00000253193 | LRP3 | 0.3553 |
| ENSP00000298119 | LRFN5 | 0.3553 |
| ENSP00000313571 | LGALS7B | 0.3553 |
| ENSP00000343490 | LCTL | 0.3553 |
| ENSP00000371327 | KLRC2 | 0.3553 |
| ENSP00000454657 | KIAA0391 | 0.3553 |
| ENSP00000258111 | KCNMB4 | 0.3553 |
| ENSP00000302166 | KCNK9 | 0.3553 |
| ENSP00000341479 | KCNJ14 | 0.3553 |
| ENSP00000328923 | KANK3 | 0.3553 |
| ENSP00000321724 | INSL3 | 0.3553 |

| | | |
|---|---|---|
| ENSP00000246532 | IGFLR1 | 0.3553 |
| ENSP00000359579 | HS2ST1 | 0.3553 |
| ENSP00000360299 | GPR110 | 0.3553 |
| ENSP00000301671 | GHDC | 0.3553 |
| ENSP00000252318 | GALNT8 | 0.3553 |
| ENSP00000452885 | FAM63B | 0.3553 |
| ENSP00000348852 | FAM168A | 0.3553 |
| ENSP00000420608 | EMC1 | 0.3553 |
| ENSP00000367815 | DNAAF1 | 0.3553 |
| ENSP00000220812 | DKK4 | 0.3553 |
| ENSP00000215773 | DDT | 0.3553 |
| ENSP00000414904 | CTSO | 0.3553 |
| ENSP00000438400 | CLDN24 | 0.3553 |
| ENSP00000349954 | CGB | 0.3553 |
| ENSP00000312367 | CD84 | 0.3553 |
| ENSP00000378167 | CCDC66 | 0.3553 |
| ENSP00000307666 | CCDC132 | 0.3553 |
| ENSP00000314407 | CA8 | 0.3553 |
| ENSP00000431184 | C6orf141 | 0.3553 |
| ENSP00000290390 | C2orf81 | 0.3553 |
| ENSP00000420959 | C2orf15 | 0.3553 |
| ENSP00000381132 | C12orf66 | 0.3553 |
| ENSP00000252934 | ATXN10 | 0.3553 |
| ENSP00000469019 | ASCL5 | 0.3553 |
| ENSP00000296525 | ASB5 | 0.3553 |
| ENSP00000253332 | AKAP12 | 0.3553 |
| ENSP00000362273 | ADPRHL2 | 0.3553 |
| ENSP00000270328 | ADAMTS10 | 0.3553 |
| ENSP00000419512 | BTNL2 | 0.3553 |
| ENSP00000425257 | hCG_33128 | 0.3553 |
| ENSP00000379702 | ZNF665 | 0.3526 |
| ENSP00000311319 | ZNF417 | 0.3526 |
| ENSP00000296326 | ZDHHC19 | 0.3526 |
| ENSP00000359364 | YTHDF1 | 0.3526 |
| ENSP00000425582 | USP17L19 | 0.3526 |
| ENSP00000423503 | USP17L18 | 0.3526 |
| ENSP00000410621 | USP17L15 | 0.3526 |
| ENSP00000390759 | USP17L13 | 0.3526 |
| ENSP00000389443 | USP17L12 | 0.3526 |
| ENSP00000400880 | USP17L11 | 0.3526 |
| ENSP00000355437 | TRIM58 | 0.3526 |
| ENSP00000230099 | TRIM38 | 0.3526 |
| ENSP00000365884 | TRIM15 | 0.3526 |
| ENSP00000263663 | TAF1B | 0.3526 |
| ENSP00000268164 | ST8SIA2 | 0.3526 |
| ENSP00000300917 | SMG8 | 0.3526 |
| ENSP00000286749 | SLC28A1 | 0.3526 |
| ENSP00000329452 | SLC25A21 | 0.3526 |
| ENSP00000244527 | SLC17A1 | 0.3526 |
| ENSP00000215727 | SERPIND1 | 0.3526 |
| ENSP00000337133 | SERPINA9 | 0.3526 |
| ENSP00000329008 | S100A7A | 0.3526 |
| ENSP00000421307 | RP11-455G16.1 | 0.3526 |

| | | |
|---|---|---|
| ENSP00000269391 | RNF157 | 0.3526 |
| ENSP00000328325 | PTTG1IP | 0.3526 |
| ENSP00000275072 | PM20D2 | 0.3526 |
| ENSP00000337757 | PIH1D3 | 0.3526 |
| ENSP00000282096 | PDE3B | 0.3526 |
| ENSP00000259467 | PDCL | 0.3526 |
| ENSP00000338330 | PAQR6 | 0.3526 |
| ENSP00000341422 | P4HTM | 0.3526 |
| ENSP00000342535 | NLGN4Y | 0.3526 |
| ENSP00000415034 | NBEAL2 | 0.3526 |
| ENSP00000413064 | NAT8L | 0.3526 |
| ENSP00000261520 | NARG2 | 0.3526 |
| ENSP00000225927 | NAGLU | 0.3526 |
| ENSP00000274054 | NAF1 | 0.3526 |
| ENSP00000260229 | MMP27 | 0.3526 |
| ENSP00000228506 | MLEC | 0.3526 |
| ENSP00000255977 | MKRN1 | 0.3526 |
| ENSP00000265498 | MGST2 | 0.3526 |
| ENSP00000408058 | METTL11B | 0.3526 |
| ENSP00000311496 | MARCH10 | 0.3526 |
| ENSP00000272462 | MALL | 0.3526 |
| ENSP00000368273 | MAGEB2 | 0.3526 |
| ENSP00000328625 | LRRC33 | 0.3526 |
| ENSP00000326604 | LMBR1 | 0.3526 |
| ENSP00000303366 | LMAN2 | 0.3526 |
| ENSP00000283936 | KCNJ16 | 0.3526 |
| ENSP00000432163 | KBTBD3 | 0.3526 |
| ENSP00000302586 | KBTBD2 | 0.3526 |
| ENSP00000323780 | IP6K1 | 0.3526 |
| ENSP00000421191 | IGSF22 | 0.3526 |
| ENSP00000284110 | HS3ST3A1 | 0.3526 |
| ENSP00000284674 | GPR26 | 0.3526 |
| ENSP00000274545 | GABRA6 | 0.3526 |
| ENSP00000398502 | FAM86A | 0.3526 |
| ENSP00000356382 | F13B | 0.3526 |
| ENSP00000348380 | CYP20A1 | 0.3526 |
| ENSP00000272852 | CPO | 0.3526 |
| ENSP00000398105 | CHAC1 | 0.3526 |
| ENSP00000385739 | CEACAM21 | 0.3526 |
| ENSP00000354504 | ASTN2 | 0.3526 |
| ENSP00000301776 | ASRGL1 | 0.3526 |
| ENSP00000326627 | ASIC4 | 0.3526 |
| ENSP00000269299 | ASGR1 | 0.3526 |
| ENSP00000350331 | ASB13 | 0.3526 |
| ENSP00000370546 | ARSD | 0.3526 |
| ENSP00000216124 | ARSA | 0.3526 |
| ENSP00000371200 | ARRDC5 | 0.3526 |
| ENSP00000362268 | APOOL | 0.3526 |
| ENSP00000265224 | ANKRD7 | 0.3526 |
| ENSP00000261739 | ANKRD13A | 0.3526 |
| ENSP00000305988 | ALCAM | 0.3526 |
| ENSP00000323816 | AHRR | 0.3526 |
| ENSP00000472696 | AD000671.6 | 0.3526 |

| | | |
|---|---|---|
| ENSP00000348459 | ACMSD | 0.3526 |
| ENSP00000269081 | ABCA10 | 0.3526 |
| ENSP00000005905 | KIAA0100 | 0.3526 |
| ENSP00000371051 | ZZEF1 | 0.3500 |
| ENSP00000364023 | ZXDB | 0.3500 |
| ENSP00000335384 | ZPBP2 | 0.3500 |
| ENSP00000278853 | ZP1 | 0.3500 |
| ENSP00000309606 | ZNF408 | 0.3500 |
| ENSP00000303468 | ZCCHC4 | 0.3500 |
| ENSP00000362758 | ZBTB26 | 0.3500 |
| ENSP00000306279 | UMOD | 0.3500 |
| ENSP00000360688 | TXNDC12 | 0.3500 |
| ENSP00000320869 | TRIM41 | 0.3500 |
| ENSP00000309818 | TRHR | 0.3500 |
| ENSP00000289373 | TMSB15A | 0.3500 |
| ENSP00000365567 | TM9SF2 | 0.3500 |
| ENSP00000386186 | TIGD1 | 0.3500 |
| ENSP00000352424 | TIAF1 | 0.3500 |
| ENSP00000283025 | TEKT5 | 0.3500 |
| ENSP00000384996 | TBC1D10A | 0.3500 |
| ENSP00000431719 | TAS2R43 | 0.3500 |
| ENSP00000376822 | STEAP3 | 0.3500 |
| ENSP00000338030 | STARD7 | 0.3500 |
| ENSP00000320634 | SLCO3A1 | 0.3500 |
| ENSP00000370964 | SLCO1C1 | 0.3500 |
| ENSP00000301454 | SLC25A23 | 0.3500 |
| ENSP00000329033 | SLC25A18 | 0.3500 |
| ENSP00000247020 | SDF2 | 0.3500 |
| ENSP00000354061 | SCYL2 | 0.3500 |
| ENSP00000221538 | RSPH6A | 0.3500 |
| ENSP00000473200 | RP11-295P9.3 | 0.3500 |
| ENSP00000315950 | RNF32 | 0.3500 |
| ENSP00000270631 | PTH2 | 0.3500 |
| ENSP00000370430 | PNPLA4 | 0.3500 |
| ENSP00000221957 | PLIN3 | 0.3500 |
| ENSP00000432688 | PIGY | 0.3500 |
| ENSP00000164305 | PIGB | 0.3500 |
| ENSP00000364635 | PADI2 | 0.3500 |
| ENSP00000393559 | PACS2 | 0.3500 |
| ENSP00000263314 | P2RX3 | 0.3500 |
| ENSP00000278780 | OVOL2 | 0.3500 |
| ENSP00000394051 | NOSTRIN | 0.3500 |
| ENSP00000305877 | NMUR1 | 0.3500 |
| ENSP00000345292 | NDRG3 | 0.3500 |
| ENSP00000348050 | MXRA7 | 0.3500 |
| ENSP00000296003 | MTMR14 | 0.3500 |
| ENSP00000281416 | MFSD6 | 0.3500 |
| ENSP00000384115 | MEI1 | 0.3500 |
| ENSP00000261407 | LPCAT3 | 0.3500 |
| ENSP00000315564 | LGALS9B | 0.3500 |
| ENSP00000296233 | KLF15 | 0.3500 |
| ENSP00000353331 | KIRREL2 | 0.3500 |
| ENSP00000432561 | HTN3 | 0.3500 |

| | | |
|---|---|---|
| ENSP00000364493 | HIATL1 | 0.3500 |
| ENSP00000262895 | GRIK5 | 0.3500 |
| ENSP00000318650 | GREM2 | 0.3500 |
| ENSP00000340200 | GLYAT | 0.3500 |
| ENSP00000271234 | FNBP1L | 0.3500 |
| ENSP00000374218 | ESYT3 | 0.3500 |
| ENSP00000256951 | EMP1 | 0.3500 |
| ENSP00000333234 | DEFB108B | 0.3500 |
| ENSP00000376188 | CRLF1 | 0.3500 |
| ENSP00000411904 | CPNE4 | 0.3500 |
| ENSP00000366673 | CLN5 | 0.3500 |
| ENSP00000258385 | CHRND | 0.3500 |
| ENSP00000161559 | CEACAM1 | 0.3500 |
| ENSP00000368080 | CDKL4 | 0.3500 |
| ENSP00000363330 | CD52 | 0.3500 |
| ENSP00000253968 | BARX1 | 0.3500 |
| ENSP00000358886 | ATXN7L2 | 0.3500 |
| ENSP00000386398 | ANKRD30BL | 0.3500 |
| ENSP00000319713 | AGTRAP | 0.3500 |
| ENSP00000375599 | DNAAF3 | 0.3500 |
| ENSP00000349401 | ZNF708 | 0.3474 |
| ENSP00000393876 | ZNF655 | 0.3474 |
| ENSP00000343617 | ZNF606 | 0.3474 |
| ENSP00000327314 | ZNF283 | 0.3474 |
| ENSP00000282308 | ZNF256 | 0.3474 |
| ENSP00000383563 | ZG16 | 0.3474 |
| ENSP00000362556 | ZBTB43 | 0.3474 |
| ENSP00000254029 | WDR44 | 0.3474 |
| ENSP00000263150 | WDR37 | 0.3474 |
| ENSP00000308976 | VWC2L | 0.3474 |
| ENSP00000345216 | USP54 | 0.3474 |
| ENSP00000274278 | UGT3A1 | 0.3474 |
| ENSP00000306344 | TMEM135 | 0.3474 |
| ENSP00000389277 | TMBIM6 | 0.3474 |
| ENSP00000308708 | TCP11 | 0.3474 |
| ENSP00000244625 | TBCC | 0.3474 |
| ENSP00000258034 | TAAR5 | 0.3474 |
| ENSP00000312284 | SPESP1 | 0.3474 |
| ENSP00000352167 | SLC25A29 | 0.3474 |
| ENSP00000239451 | SLC25A2 | 0.3474 |
| ENSP00000236137 | SLC19A2 | 0.3474 |
| ENSP00000291707 | SIGLEC12 | 0.3474 |
| ENSP00000341584 | SERPINB13 | 0.3474 |
| ENSP00000342109 | SERPINA12 | 0.3474 |
| ENSP00000317691 | RPP25 | 0.3474 |
| ENSP00000274134 | ROPN1L | 0.3474 |
| ENSP00000252998 | RBBP8NL | 0.3474 |
| ENSP00000330389 | PTRHD1 | 0.3474 |
| ENSP00000262293 | PRR11 | 0.3474 |
| ENSP00000408288 | PPP1R3E | 0.3474 |
| ENSP00000011684 | PLEKHG6 | 0.3474 |
| ENSP00000246229 | PLAGL2 | 0.3474 |
| ENSP00000352782 | PHOSPHO2 | 0.3474 |

| | | |
|---|---|---|
| ENSP00000364609 | PADI3 | 0.3474 |
| ENSP00000451112 | NPC2 | 0.3474 |
| ENSP00000294064 | NEU3 | 0.3474 |
| ENSP00000307887 | MXRA8 | 0.3474 |
| ENSP00000217939 | MXRA5 | 0.3474 |
| ENSP00000395253 | MUC19 | 0.3474 |
| ENSP00000391668 | MTMR11 | 0.3474 |
| ENSP00000379669 | MPV17L | 0.3474 |
| ENSP00000350353 | METTL20 | 0.3474 |
| ENSP00000345985 | LRFN2 | 0.3474 |
| ENSP00000296603 | LMBRD2 | 0.3474 |
| ENSP00000366280 | LMAN2L | 0.3474 |
| ENSP00000384979 | LINGO3 | 0.3474 |
| ENSP00000318374 | LENG8 | 0.3474 |
| ENSP00000473047 | KLK11 | 0.3474 |
| ENSP00000309570 | KLF7 | 0.3474 |
| ENSP00000357881 | IKZF5 | 0.3474 |
| ENSP00000234389 | GRIN3B | 0.3474 |
| ENSP00000314223 | GPR88 | 0.3474 |
| ENSP00000267549 | GPR65 | 0.3474 |
| ENSP00000352547 | GPAT2 | 0.3474 |
| ENSP00000361700 | GLRA4 | 0.3474 |
| ENSP00000259056 | GALNT5 | 0.3474 |
| ENSP00000436604 | GALNT4 | 0.3474 |
| ENSP00000344729 | FAM166A | 0.3474 |
| ENSP00000424176 | EPPIN-WFDC6 | 0.3474 |
| ENSP00000452085 | EPPIN | 0.3474 |
| ENSP00000216799 | EMC9 | 0.3474 |
| ENSP00000257312 | DZIP1 | 0.3474 |
| ENSP00000363023 | DLGAP4 | 0.3474 |
| ENSP00000354623 | DFNB31 | 0.3474 |
| ENSP00000376957 | DENND2C | 0.3474 |
| ENSP00000225428 | CWC25 | 0.3474 |
| ENSP00000366208 | CST11 | 0.3474 |
| ENSP00000369614 | CNTROB | 0.3474 |
| ENSP00000412365 | CDKL2 | 0.3474 |
| ENSP00000227520 | CCDC86 | 0.3474 |
| ENSP00000453019 | C9orf69 | 0.3474 |
| ENSP00000319914 | C8orf4 | 0.3474 |
| ENSP00000360281 | C8B | 0.3474 |
| ENSP00000357922 | C1orf56 | 0.3474 |
| ENSP00000303740 | B3GALT1 | 0.3474 |
| ENSP00000386532 | ASB18 | 0.3474 |
| ENSP00000358896 | AS3MT | 0.3474 |
| ENSP00000365255 | ANKRD26 | 0.3474 |
| ENSP00000367263 | AHNAK | 0.3474 |
| ENSP00000380999 | ZNF846 | 0.3447 |
| ENSP00000379387 | ZNF445 | 0.3447 |
| ENSP00000338572 | ZNF212 | 0.3447 |
| ENSP00000317542 | WASH4P | 0.3447 |
| ENSP00000425955 | USP17L5 | 0.3447 |
| ENSP00000264689 | UFSP2 | 0.3447 |
| ENSP00000374387 | TRMT44 | 0.3447 |

| | | |
|---|---|---|
| ENSP00000332288 | TRIM61 | 0.3447 |
| ENSP00000348216 | TRIM4 | 0.3447 |
| ENSP00000283206 | TMEM87B | 0.3447 |
| ENSP00000381102 | TMEM55B | 0.3447 |
| ENSP00000363478 | TMEM50A | 0.3447 |
| ENSP00000386264 | TMEM237 | 0.3447 |
| ENSP00000282670 | TCTEX1D1 | 0.3447 |
| ENSP00000247879 | TAS2R3 | 0.3447 |
| ENSP00000362095 | SRPX2 | 0.3447 |
| ENSP00000217159 | SLCO4A1 | 0.3447 |
| ENSP00000258538 | SLC41A2 | 0.3447 |
| ENSP00000261918 | SEMA7A | 0.3447 |
| ENSP00000409799 | RPP21 | 0.3447 |
| ENSP00000404011 | RP11-13B9.3 | 0.3447 |
| ENSP00000328287 | RNF123 | 0.3447 |
| ENSP00000349824 | REPS2 | 0.3447 |
| ENSP00000304311 | REG3A | 0.3447 |
| ENSP00000348645 | PXDNL | 0.3447 |
| ENSP00000230381 | PRPH2 | 0.3447 |
| ENSP00000362125 | PCDH19 | 0.3447 |
| ENSP00000341564 | PAQR9 | 0.3447 |
| ENSP00000421981 | PAQR3 | 0.3447 |
| ENSP00000310337 | OR4S2 | 0.3447 |
| ENSP00000355435 | OR2L2 | 0.3447 |
| ENSP00000289820 | NPM2 | 0.3447 |
| ENSP00000404705 | NAALADL2 | 0.3447 |
| ENSP00000326240 | MYNN | 0.3447 |
| ENSP00000424328 | MRPS30 | 0.3447 |
| ENSP00000244711 | MEA1 | 0.3447 |
| ENSP00000359307 | MAGEA2 | 0.3447 |
| ENSP00000309576 | LGALS8 | 0.3447 |
| ENSP00000383505 | KLHDC7A | 0.3447 |
| ENSP00000296121 | KIAA1143 | 0.3447 |
| ENSP00000264773 | KCNN2 | 0.3447 |
| ENSP00000259895 | GTF2H4 | 0.3447 |
| ENSP00000356658 | GPR52 | 0.3447 |
| ENSP00000296882 | GJB7 | 0.3447 |
| ENSP00000368805 | GCM2 | 0.3447 |
| ENSP00000348668 | GALNT6 | 0.3447 |
| ENSP00000295452 | GABRG1 | 0.3447 |
| ENSP00000384900 | ERLIN1 | 0.3447 |
| ENSP00000282478 | DSPP | 0.3447 |
| ENSP00000403984 | DCDC2C | 0.3447 |
| ENSP00000366953 | DCAF10 | 0.3447 |
| ENSP00000252050 | CUL9 | 0.3447 |
| ENSP00000356370 | CRB1 | 0.3447 |
| ENSP00000443985 | CPEB2 | 0.3447 |
| ENSP00000289957 | CHRNB3 | 0.3447 |
| ENSP00000418428 | CHCHD10 | 0.3447 |
| ENSP00000380690 | CCDC64 | 0.3447 |
| ENSP00000425166 | C1orf216 | 0.3447 |
| ENSP00000278483 | C11orf73 | 0.3447 |
| ENSP00000216629 | BDKRB1 | 0.3447 |

| | | |
|---|---|---|
| ENSP00000303042 | AFAP1L2 | 0.3447 |
| ENSP00000274849 | ABT1 | 0.3447 |
| ENSP00000376443 | ABCA5 | 0.3447 |
| ENSP00000438833 | MST1L | 0.3447 |
| ENSP00000417470 | ZNF786 | 0.3421 |
| ENSP00000382513 | ZNF594 | 0.3421 |
| ENSP00000292562 | ZNF251 | 0.3421 |
| ENSP00000340299 | ZDHHC1 | 0.3421 |
| ENSP00000362763 | ZBTB6 | 0.3421 |
| ENSP00000403760 | USP17L10 | 0.3421 |
| ENSP00000320076 | USH1G | 0.3421 |
| ENSP00000258821 | TTC5 | 0.3421 |
| ENSP00000327487 | TSEN54 | 0.3421 |
| ENSP00000274773 | TRIM7 | 0.3421 |
| ENSP00000343765 | TRIM60 | 0.3421 |
| ENSP00000308310 | TRIM40 | 0.3421 |
| ENSP00000186436 | TMEM131 | 0.3421 |
| ENSP00000299134 | SVOP | 0.3421 |
| ENSP00000348314 | SPEF2 | 0.3421 |
| ENSP00000340799 | SP6 | 0.3421 |
| ENSP00000311427 | SNX33 | 0.3421 |
| ENSP00000244227 | SNRNP27 | 0.3421 |
| ENSP00000370543 | SLC5A3 | 0.3421 |
| ENSP00000356105 | SLC41A1 | 0.3421 |
| ENSP00000275300 | SLC22A3 | 0.3421 |
| ENSP00000248958 | SDF2L1 | 0.3421 |
| ENSP00000357692 | S100A16 | 0.3421 |
| ENSP00000376258 | RNF34 | 0.3421 |
| ENSP00000364263 | RNF186 | 0.3421 |
| ENSP00000320508 | RNF185 | 0.3421 |
| ENSP00000347443 | RASSF5 | 0.3421 |
| ENSP00000264160 | R3HDM1 | 0.3421 |
| ENSP00000271331 | PROK1 | 0.3421 |
| ENSP00000383587 | PNRC2 | 0.3421 |
| ENSP00000366460 | PLXDC2 | 0.3421 |
| ENSP00000331106 | PEX26 | 0.3421 |
| ENSP00000308466 | PBLD | 0.3421 |
| ENSP00000365318 | NFKBIL1 | 0.3421 |
| ENSP00000351484 | NAALADL1 | 0.3421 |
| ENSP00000432481 | NAALAD2 | 0.3421 |
| ENSP00000365473 | MUC21 | 0.3421 |
| ENSP00000271373 | MPC2 | 0.3421 |
| ENSP00000438205 | MANSC1 | 0.3421 |
| ENSP00000333487 | MAGEA2B | 0.3421 |
| ENSP00000340983 | LRRC25 | 0.3421 |
| ENSP00000375829 | LAD1 | 0.3421 |
| ENSP00000366894 | KLF12 | 0.3421 |
| ENSP00000386738 | KCMF1 | 0.3421 |
| ENSP00000362395 | ITM2A | 0.3421 |
| ENSP00000354213 | HS3ST3B1 | 0.3421 |
| ENSP00000247584 | HIVEP3 | 0.3421 |
| ENSP00000251287 | HCN2 | 0.3421 |
| ENSP00000259254 | GYPC | 0.3421 |

| | | |
|---|---|---|
| ENSP00000359512 | GBP3 | 0.3421 |
| ENSP00000206595 | G2E3 | 0.3421 |
| ENSP00000389770 | FXYD3 | 0.3421 |
| ENSP00000263773 | FNBP4 | 0.3421 |
| ENSP00000354481 | FAM5B | 0.3421 |
| ENSP00000353025 | FAM3C | 0.3421 |
| ENSP00000322323 | FAM20C | 0.3421 |
| ENSP00000335076 | EDARADD | 0.3421 |
| ENSP00000359699 | DNASE2B | 0.3421 |
| ENSP00000308597 | DLEC1 | 0.3421 |
| ENSP00000261038 | DIRC2 | 0.3421 |
| ENSP00000328359 | DEFA3 | 0.3421 |
| ENSP00000262207 | CRISPLD1 | 0.3421 |
| ENSP00000369268 | CNGA4 | 0.3421 |
| ENSP00000366493 | CGB7 | 0.3421 |
| ENSP00000256151 | CCDC59 | 0.3421 |
| ENSP00000440765 | C16orf13 | 0.3421 |
| ENSP00000341562 | B4GALNT1 | 0.3421 |
| ENSP00000343002 | B3GALTL | 0.3421 |
| ENSP00000368496 | B3GALT6 | 0.3421 |
| ENSP00000293414 | ASB16 | 0.3421 |
| ENSP00000407193 | ARSG | 0.3421 |
| ENSP00000382610 | ANKRD18A | 0.3421 |
| ENSP00000228850 | AKAP3 | 0.3421 |
| ENSP00000256997 | ACP2 | 0.3421 |
| ENSP00000337320 | ZNF664 | 0.3395 |
| ENSP00000344129 | ZNF648 | 0.3395 |
| ENSP00000354414 | ZNF627 | 0.3395 |
| ENSP00000288466 | ZNF618 | 0.3395 |
| ENSP00000219091 | ZNF205 | 0.3395 |
| ENSP00000311221 | ZMAT3 | 0.3395 |
| ENSP00000352820 | WDSUB1 | 0.3395 |
| ENSP00000283713 | VILL | 0.3395 |
| ENSP00000054668 | UTS2 | 0.3395 |
| ENSP00000334276 | UGT2B28 | 0.3395 |
| ENSP00000461518 | TRPV3 | 0.3395 |
| ENSP00000335371 | TRAPPC11 | 0.3395 |
| ENSP00000368391 | TPD52 | 0.3395 |
| ENSP00000421848 | TNFAIP8 | 0.3395 |
| ENSP00000284885 | TMPRSS15 | 0.3395 |
| ENSP00000363824 | TMEM38B | 0.3395 |
| ENSP00000370736 | TMEM165 | 0.3395 |
| ENSP00000308540 | TBC1D3 | 0.3395 |
| ENSP00000405095 | TAS2R39 | 0.3395 |
| ENSP00000256366 | SYNJ2BP | 0.3395 |
| ENSP00000308770 | SULT1B1 | 0.3395 |
| ENSP00000337804 | SPAG17 | 0.3395 |
| ENSP00000289932 | SLC5A11 | 0.3395 |
| ENSP00000354689 | SLC39A13 | 0.3395 |
| ENSP00000261892 | SLC24A1 | 0.3395 |
| ENSP00000373420 | RUFY2 | 0.3395 |
| ENSP00000304945 | ROBO4 | 0.3395 |
| ENSP00000321330 | RNF144A | 0.3395 |

| | | |
|---|---|---|
| ENSP00000341361 | RNF13 | 0.3395 |
| ENSP00000261655 | RIMBP2 | 0.3395 |
| ENSP00000064780 | RELT | 0.3395 |
| ENSP00000308258 | PTDSS2 | 0.3395 |
| ENSP00000405514 | PPP1R9A | 0.3395 |
| ENSP00000464890 | POU5F2 | 0.3395 |
| ENSP00000389787 | PLEKHF1 | 0.3395 |
| ENSP00000347931 | PCNXL3 | 0.3395 |
| ENSP00000351153 | PARPBP | 0.3395 |
| ENSP00000243045 | ORMDL2 | 0.3395 |
| ENSP00000328563 | OR4D9 | 0.3395 |
| ENSP00000255262 | NMUR2 | 0.3395 |
| ENSP00000262510 | NLRC5 | 0.3395 |
| ENSP00000357341 | NCOA7 | 0.3395 |
| ENSP00000298894 | MOAP1 | 0.3395 |
| ENSP00000289359 | MITD1 | 0.3395 |
| ENSP00000010404 | MGST1 | 0.3395 |
| ENSP00000309610 | METTL2A | 0.3395 |
| ENSP00000359640 | MCOLN2 | 0.3395 |
| ENSP00000300231 | MAP1A | 0.3395 |
| ENSP00000423463 | LY75-CD302 | 0.3395 |
| ENSP00000341944 | LRRC66 | 0.3395 |
| ENSP00000384291 | LRP5L | 0.3395 |
| ENSP00000326563 | KDM3B | 0.3395 |
| ENSP00000351068 | KCNMB2 | 0.3395 |
| ENSP00000242591 | IFT81 | 0.3395 |
| ENSP00000184956 | HEATR6 | 0.3395 |
| ENSP00000355902 | GPATCH2 | 0.3395 |
| ENSP00000317027 | GCNT4 | 0.3395 |
| ENSP00000357644 | GATAD2B | 0.3395 |
| ENSP00000386029 | GABRR2 | 0.3395 |
| ENSP00000359334 | GABRA3 | 0.3395 |
| ENSP00000264433 | FNIP2 | 0.3395 |
| ENSP00000421985 | FNIP1 | 0.3395 |
| ENSP00000278829 | FADS3 | 0.3395 |
| ENSP00000258534 | DRAM1 | 0.3395 |
| ENSP00000258390 | DOCK10 | 0.3395 |
| ENSP00000324633 | DEFB103B | 0.3395 |
| ENSP00000364404 | DCAF17 | 0.3395 |
| ENSP00000305731 | CST1 | 0.3395 |
| ENSP00000244751 | CPNE5 | 0.3395 |
| ENSP00000396304 | CLDN25 | 0.3395 |
| ENSP00000352071 | CD163 | 0.3395 |
| ENSP00000224181 | C8G | 0.3395 |
| ENSP00000334267 | C5orf38 | 0.3395 |
| ENSP00000258761 | BZW2 | 0.3395 |
| ENSP00000305595 | B3GNT2 | 0.3395 |
| ENSP00000296557 | ARFIP1 | 0.3395 |
| ENSP00000249066 | APOL2 | 0.3395 |
| ENSP00000381250 | APOF | 0.3395 |
| ENSP00000363395 | ANXA8L2 | 0.3395 |
| ENSP00000387233 | ANKRD44 | 0.3395 |
| ENSP00000310015 | ADAT1 | 0.3395 |

| | | |
|---|---|---|
| ENSP00000238618 | ACYP1 | 0.3395 |
| ENSP00000289119 | ABHD3 | 0.3395 |
| ENSP00000379282 | ABHD16A | 0.3395 |
| ENSP00000239730 | SEPT7L | 0.3395 |
| ENSP00000353957 | ZNF567 | 0.3368 |
| ENSP00000362609 | ZBTB8A | 0.3368 |
| ENSP00000376139 | WDR45B | 0.3368 |
| ENSP00000261708 | UTP6 | 0.3368 |
| ENSP00000373073 | UROC1 | 0.3368 |
| ENSP00000244565 | UNC5CL | 0.3368 |
| ENSP00000354883 | UBL7 | 0.3368 |
| ENSP00000229771 | TULP1 | 0.3368 |
| ENSP00000257910 | TSPAN31 | 0.3368 |
| ENSP00000299882 | TMPRSS5 | 0.3368 |
| ENSP00000401338 | TMEM8A | 0.3368 |
| ENSP00000373167 | TIGIT | 0.3368 |
| ENSP00000309007 | THAP6 | 0.3368 |
| ENSP00000361707 | TCEAL1 | 0.3368 |
| ENSP00000334050 | TAS2R42 | 0.3368 |
| ENSP00000352655 | SLC39A10 | 0.3368 |
| ENSP00000289292 | SHROOM4 | 0.3368 |
| ENSP00000351395 | SCGB1D4 | 0.3368 |
| ENSP00000382895 | RPGRIP1 | 0.3368 |
| ENSP00000455258 | RP11-58C22.1 | 0.3368 |
| ENSP00000310356 | RMI2 | 0.3368 |
| ENSP00000303712 | RBMY1A1 | 0.3368 |
| ENSP00000351608 | PRRT2 | 0.3368 |
| ENSP00000296682 | PRDM9 | 0.3368 |
| ENSP00000356849 | POGK | 0.3368 |
| ENSP00000246151 | PITHD1 | 0.3368 |
| ENSP00000264917 | PDE8B | 0.3368 |
| ENSP00000370224 | PARM1 | 0.3368 |
| ENSP00000353953 | PAQR8 | 0.3368 |
| ENSP00000252835 | OR11H1 | 0.3368 |
| ENSP00000309124 | OLR1 | 0.3368 |
| ENSP00000352208 | MYOF | 0.3368 |
| ENSP00000278949 | MPZL3 | 0.3368 |
| ENSP00000300762 | MMP26 | 0.3368 |
| ENSP00000373476 | MEX3D | 0.3368 |
| ENSP00000292494 | LY6E | 0.3368 |
| ENSP00000365253 | LRRC38 | 0.3368 |
| ENSP00000266604 | LLPH | 0.3368 |
| ENSP00000333711 | LL0XNC01-19D8.1 | 0.3368 |
| ENSP00000392879 | LL0XNC01-19D8.1 | 0.3368 |
| ENSP00000251645 | KRT31 | 0.3368 |
| ENSP00000423897 | KLHL5 | 0.3368 |
| ENSP00000355121 | ITPRIPL1 | 0.3368 |
| ENSP00000276198 | HTR2C | 0.3368 |
| ENSP00000355870 | HLX | 0.3368 |
| ENSP00000330601 | HHIPL1 | 0.3368 |
| ENSP00000218516 | GLA | 0.3368 |
| ENSP00000275732 | GIGYF1 | 0.3368 |
| ENSP00000220822 | GDAP1 | 0.3368 |

| | | |
|---|---|---|
| ENSP00000340396 | GBP5 | 0.3368 |
| ENSP00000265029 | FETUB | 0.3368 |
| ENSP00000363398 | EXTL1 | 0.3368 |
| ENSP00000245046 | EMC3 | 0.3368 |
| ENSP00000271835 | CRNN | 0.3368 |
| ENSP00000205636 | CMTM6 | 0.3368 |
| ENSP00000352270 | CMTM5 | 0.3368 |
| ENSP00000276055 | CHST7 | 0.3368 |
| ENSP00000281282 | CGNL1 | 0.3368 |
| ENSP00000337226 | CDCA4 | 0.3368 |
| ENSP00000296449 | CCDC36 | 0.3368 |
| ENSP00000373669 | C17orf59 | 0.3368 |
| ENSP00000224652 | ATE1 | 0.3368 |
| ENSP00000261191 | ASUN | 0.3368 |
| ENSP00000244669 | APOBEC2 | 0.3368 |
| ENSP00000366826 | ANKRD20A2 | 0.3368 |
| ENSP00000358407 | ADAM30 | 0.3368 |
| ENSP00000368605 | ACOT9 | 0.3368 |
| ENSP00000232744 | ABTB1 | 0.3368 |
| ENSP00000263100 | A1BG | 0.3368 |
| ENSP00000304843 | MCOLN3 | 0.3368 |
| ENSP00000369802 | DENND4C | 0.3368 |
| ENSP00000459216 | ZNF578 | 0.3342 |
| ENSP00000340514 | ZNF14 | 0.3342 |
| ENSP00000455911 | ZFP91-CNTF | 0.3342 |
| ENSP00000307858 | ZBTB4 | 0.3342 |
| ENSP00000416707 | XKR6 | 0.3342 |
| ENSP00000258796 | TTYH3 | 0.3342 |
| ENSP00000298772 | TRIM13 | 0.3342 |
| ENSP00000359013 | TNFRSF6B | 0.3342 |
| ENSP00000261862 | THSD4 | 0.3342 |
| ENSP00000357812 | TDRKH | 0.3342 |
| ENSP00000335191 | TBX10 | 0.3342 |
| ENSP00000272452 | SULT1C4 | 0.3342 |
| ENSP00000273666 | STXBP5L | 0.3342 |
| ENSP00000279058 | SPINT4 | 0.3342 |
| ENSP00000365931 | SP2 | 0.3342 |
| ENSP00000431840 | SLC24A4 | 0.3342 |
| ENSP00000194130 | SLC13A1 | 0.3342 |
| ENSP00000347924 | SFXN4 | 0.3342 |
| ENSP00000420357 | RWDD1 | 0.3342 |
| ENSP00000355177 | RUSC2 | 0.3342 |
| ENSP00000369391 | RPP40 | 0.3342 |
| ENSP00000423391 | RFPL4B | 0.3342 |
| ENSP00000392936 | RFPL4A | 0.3342 |
| ENSP00000012049 | QPCTL | 0.3342 |
| ENSP00000317836 | PRR15 | 0.3342 |
| ENSP00000381010 | PRKRIP1 | 0.3342 |
| ENSP00000435300 | PCDHA4 | 0.3342 |
| ENSP00000333735 | NPAP1 | 0.3342 |
| ENSP00000429553 | NKX6-3 | 0.3342 |
| ENSP00000221978 | NKG7 | 0.3342 |
| ENSP00000338648 | MS4A4A | 0.3342 |

| | | |
|---|---|---|
| ENSP00000310726 | MRP63 | 0.3342 |
| ENSP00000353767 | MMP17 | 0.3342 |
| ENSP00000218721 | MLNR | 0.3342 |
| ENSP00000378851 | MAPK1IP1L | 0.3342 |
| ENSP00000368320 | MAGEB6 | 0.3342 |
| ENSP00000368135 | LRRN4 | 0.3342 |
| ENSP00000375629 | LILRB2 | 0.3342 |
| ENSP00000367198 | LECT1 | 0.3342 |
| ENSP00000263598 | LCN1 | 0.3342 |
| ENSP00000353203 | LCE1B | 0.3342 |
| ENSP00000435466 | KIRREL3 | 0.3342 |
| ENSP00000310557 | KCNE3 | 0.3342 |
| ENSP00000306106 | JAGN1 | 0.3342 |
| ENSP00000357863 | GTF3C6 | 0.3342 |
| ENSP00000256593 | GSTM5 | 0.3342 |
| ENSP00000369643 | GRPR | 0.3342 |
| ENSP00000369198 | GPR64 | 0.3342 |
| ENSP00000319744 | GPR4 | 0.3342 |
| ENSP00000328818 | GPR132 | 0.3342 |
| ENSP00000379377 | GCNT3 | 0.3342 |
| ENSP00000348897 | GABRA2 | 0.3342 |
| ENSP00000307833 | FBXO22 | 0.3342 |
| ENSP00000328187 | FBXO21 | 0.3342 |
| ENSP00000308575 | FAM46D | 0.3342 |
| ENSP00000350673 | FAM3B | 0.3342 |
| ENSP00000307181 | FAM103A1 | 0.3342 |
| ENSP00000265843 | EXPH5 | 0.3342 |
| ENSP00000430271 | ETV3L | 0.3342 |
| ENSP00000252445 | ELOF1 | 0.3342 |
| ENSP00000417052 | EBP | 0.3342 |
| ENSP00000221847 | EBI3 | 0.3342 |
| ENSP00000284031 | DDAH1 | 0.3342 |
| ENSP00000311300 | CTSW | 0.3342 |
| ENSP00000345436 | CASKIN1 | 0.3342 |
| ENSP00000297156 | CAMLG | 0.3342 |
| ENSP00000270502 | C19orf52 | 0.3342 |
| ENSP00000402020 | C17orf58 | 0.3342 |
| ENSP00000312158 | BTN2A1 | 0.3342 |
| ENSP00000335615 | BTBD7 | 0.3342 |
| ENSP00000306459 | B4GALT6 | 0.3342 |
| ENSP00000352144 | B4GALT4 | 0.3342 |
| ENSP00000216489 | ALKBH1 | 0.3342 |
| ENSP00000405700 | AL953854.2 | 0.3342 |
| ENSP00000317912 | STAP2 | 0.3342 |
| ENSP00000363064 | ZNF76 | 0.3316 |
| ENSP00000338217 | ZNF532 | 0.3316 |
| ENSP00000467931 | ZNF461 | 0.3316 |
| ENSP00000313158 | ZNF366 | 0.3316 |
| ENSP00000309429 | ZDHHC24 | 0.3316 |
| ENSP00000330485 | ZDHHC23 | 0.3316 |
| ENSP00000292778 | YDJC | 0.3316 |
| ENSP00000335522 | WDR86 | 0.3316 |
| ENSP00000292211 | UBE2Q1 | 0.3316 |

| | | |
|---|---|---|
| ENSP00000207457 | TEKT2 | 0.3316 |
| ENSP00000362390 | TBX22 | 0.3316 |
| ENSP00000386201 | TAS2R41 | 0.3316 |
| ENSP00000218894 | SUPT20H | 0.3316 |
| ENSP00000156626 | ST6GALNAC1 | 0.3316 |
| ENSP00000266086 | SLC5A4 | 0.3316 |
| ENSP00000393066 | SLC4A10 | 0.3316 |
| ENSP00000320217 | SLC26A10 | 0.3316 |
| ENSP00000358909 | SFXN2 | 0.3316 |
| ENSP00000357909 | SEMA6C | 0.3316 |
| ENSP00000388942 | SCRN1 | 0.3316 |
| ENSP00000244930 | SCGB2A1 | 0.3316 |
| ENSP00000345044 | SBK2 | 0.3316 |
| ENSP00000340979 | RNF175 | 0.3316 |
| ENSP00000346342 | RFPL1 | 0.3316 |
| ENSP00000421853 | RANBP3L | 0.3316 |
| ENSP00000349709 | RAET1E | 0.3316 |
| ENSP00000217043 | R3HDML | 0.3316 |
| ENSP00000290431 | PKD2L2 | 0.3316 |
| ENSP00000332313 | PIGW | 0.3316 |
| ENSP00000246535 | PDCD2L | 0.3316 |
| ENSP00000310585 | PCP2 | 0.3316 |
| ENSP00000358131 | OTUD7B | 0.3316 |
| ENSP00000352021 | NOL12 | 0.3316 |
| ENSP00000333938 | NEXN | 0.3316 |
| ENSP00000293201 | MYO15B | 0.3316 |
| ENSP00000248306 | METTL25 | 0.3316 |
| ENSP00000353246 | MAK16 | 0.3316 |
| ENSP00000373853 | LRRC4B | 0.3316 |
| ENSP00000424381 | LMF2 | 0.3316 |
| ENSP00000293670 | KRT83 | 0.3316 |
| ENSP00000307240 | KRT74 | 0.3316 |
| ENSP00000055682 | KIAA2022 | 0.3316 |
| ENSP00000316482 | KCTD14 | 0.3316 |
| ENSP00000415106 | KBTBD4 | 0.3316 |
| ENSP00000247933 | IDUA | 0.3316 |
| ENSP00000350049 | HOMEZ | 0.3316 |
| ENSP00000259456 | HEMGN | 0.3316 |
| ENSP00000378548 | GPR135 | 0.3316 |
| ENSP00000349687 | GM2A | 0.3316 |
| ENSP00000268699 | GAS8 | 0.3316 |
| ENSP00000361932 | FUT11 | 0.3316 |
| ENSP00000360058 | FRAT2 | 0.3316 |
| ENSP00000328230 | FFAR3 | 0.3316 |
| ENSP00000298097 | FBXO33 | 0.3316 |
| ENSP00000362817 | FABP3 | 0.3316 |
| ENSP00000361331 | ERI3 | 0.3316 |
| ENSP00000361705 | DNTTIP1 | 0.3316 |
| ENSP00000319482 | DCUN1D3 | 0.3316 |
| ENSP00000292385 | DBN1 | 0.3316 |
| ENSP00000219599 | CRYM | 0.3316 |
| ENSP00000282141 | CRYGC | 0.3316 |
| ENSP00000318804 | CRLF3 | 0.3316 |

| | | |
|---|---|---|
| ENSP00000225719 | CPD | 0.3316 |
| ENSP00000354960 | COLGALT2 | 0.3316 |
| ENSP00000258807 | CIDEB | 0.3316 |
| ENSP00000333947 | CHST15 | 0.3316 |
| ENSP00000348564 | CDAN1 | 0.3316 |
| ENSP00000313500 | C17orf53 | 0.3316 |
| ENSP00000330200 | BTNL9 | 0.3316 |
| ENSP00000289361 | BTN3A1 | 0.3316 |
| ENSP00000349022 | ARC | 0.3316 |
| ENSP00000427211 | APBB2 | 0.3316 |
| ENSP00000308772 | ANKS4B | 0.3316 |
| ENSP00000302232 | ALKBH3 | 0.3316 |
| ENSP00000342905 | ADNP | 0.3316 |
| ENSP00000381693 | ZSWIM8 | 0.3289 |
| ENSP00000243644 | ZNF350 | 0.3289 |
| ENSP00000262961 | ZFR2 | 0.3289 |
| ENSP00000362551 | ZBTB34 | 0.3289 |
| ENSP00000006526 | WDR54 | 0.3289 |
| ENSP00000248948 | VPREB3 | 0.3289 |
| ENSP00000380321 | TULP3 | 0.3289 |
| ENSP00000339813 | TPST2 | 0.3289 |
| ENSP00000325738 | TNP2 | 0.3289 |
| ENSP00000393316 | TMX1 | 0.3289 |
| ENSP00000335261 | TMLHE | 0.3289 |
| ENSP00000360222 | TM2D1 | 0.3289 |
| ENSP00000363031 | THEMIS2 | 0.3289 |
| ENSP00000429430 | TENM2 | 0.3289 |
| ENSP00000340914 | SYT3 | 0.3289 |
| ENSP00000361032 | SURF2 | 0.3289 |
| ENSP00000265087 | STC2 | 0.3289 |
| ENSP00000329200 | STAC3 | 0.3289 |
| ENSP00000291672 | SPATC1L | 0.3289 |
| ENSP00000301921 | SOX7 | 0.3289 |
| ENSP00000262861 | SIPA1L2 | 0.3289 |
| ENSP00000356744 | SCYL3 | 0.3289 |
| ENSP00000357901 | SCNM1 | 0.3289 |
| ENSP00000296142 | RTP3 | 0.3289 |
| ENSP00000303775 | PROKR1 | 0.3289 |
| ENSP00000260648 | PREPL | 0.3289 |
| ENSP00000358217 | PRDM13 | 0.3289 |
| ENSP00000361052 | POMGNT1 | 0.3289 |
| ENSP00000410216 | POC5 | 0.3289 |
| ENSP00000365505 | PLP2 | 0.3289 |
| ENSP00000263265 | PLEKHA4 | 0.3289 |
| ENSP00000358753 | PIFO | 0.3289 |
| ENSP00000295992 | PCOLCE2 | 0.3289 |
| ENSP00000384408 | PARG | 0.3289 |
| ENSP00000379003 | NUPR1 | 0.3289 |
| ENSP00000332766 | NPB | 0.3289 |
| ENSP00000383840 | MMACHC | 0.3289 |
| ENSP00000388825 | MLN | 0.3289 |
| ENSP00000298296 | MAGEC3 | 0.3289 |
| ENSP00000357762 | KPRP | 0.3289 |

| | | |
|---|---|---|
| ENSP00000385879 | KBTBD12 | 0.3289 |
| ENSP00000333350 | JRKL | 0.3289 |
| ENSP00000348986 | INS-IGF2 | 0.3289 |
| ENSP00000342114 | ICAM4 | 0.3289 |
| ENSP00000253457 | EMC8 | 0.3289 |
| ENSP00000319705 | DUOXA2 | 0.3289 |
| ENSP00000261615 | DPEP1 | 0.3289 |
| ENSP00000371546 | DEPDC5 | 0.3289 |
| ENSP00000353373 | CUEDC1 | 0.3289 |
| ENSP00000295589 | CCKAR | 0.3289 |
| ENSP00000355492 | C1orf101 | 0.3289 |
| ENSP00000253110 | C19orf66 | 0.3289 |
| ENSP00000373657 | C17orf85 | 0.3289 |
| ENSP00000304987 | BFSP2 | 0.3289 |
| ENSP00000360776 | B4GALT5 | 0.3289 |
| ENSP00000390784 | B3GALT4 | 0.3289 |
| ENSP00000347152 | ATRNL1 | 0.3289 |
| ENSP00000347802 | ANKRD35 | 0.3289 |
| ENSP00000359982 | ANKRD13C | 0.3289 |
| ENSP00000267339 | ANKRD10 | 0.3289 |
| ENSP00000226355 | AFM | 0.3289 |
| ENSP00000264377 | ADAM23 | 0.3289 |
| ENSP00000364005 | LPPR1 | 0.3289 |
| ENSP00000046087 | ZPBP | 0.3263 |
| ENSP00000369777 | ZNF768 | 0.3263 |
| ENSP00000294740 | ZNF281 | 0.3263 |
| ENSP00000219069 | ZNF263 | 0.3263 |
| ENSP00000361790 | ZFP69 | 0.3263 |
| ENSP00000387462 | ZBTB10 | 0.3263 |
| ENSP00000252826 | TRPM4 | 0.3263 |
| ENSP00000309792 | TMEM234 | 0.3263 |
| ENSP00000355162 | TIGD4 | 0.3263 |
| ENSP00000304941 | TCTN2 | 0.3263 |
| ENSP00000354777 | TBKBP1 | 0.3263 |
| ENSP00000357836 | SNX27 | 0.3263 |
| ENSP00000266980 | SLC39A5 | 0.3263 |
| ENSP00000347778 | SLC26A8 | 0.3263 |
| ENSP00000269298 | SAT2 | 0.3263 |
| ENSP00000216487 | RIN3 | 0.3263 |
| ENSP00000344779 | RHBDD1 | 0.3263 |
| ENSP00000331342 | RAB11FIP1 | 0.3263 |
| ENSP00000382241 | QSER1 | 0.3263 |
| ENSP00000296666 | PRRC1 | 0.3263 |
| ENSP00000307292 | PRKCDBP | 0.3263 |
| ENSP00000358106 | PREP | 0.3263 |
| ENSP00000264254 | PDCL3 | 0.3263 |
| ENSP00000322249 | ORAI3 | 0.3263 |
| ENSP00000309767 | NLRP6 | 0.3263 |
| ENSP00000345716 | NKAPL | 0.3263 |
| ENSP00000320318 | NEU4 | 0.3263 |
| ENSP00000367024 | NAA40 | 0.3263 |
| ENSP00000315064 | MAGEF1 | 0.3263 |
| ENSP00000294818 | LRRC52 | 0.3263 |

| | | |
|---|---|---|
| ENSP00000371548 | LGI2 | 0.3263 |
| ENSP00000357768 | LCE2A | 0.3263 |
| ENSP00000310216 | KLRC4 | 0.3263 |
| ENSP00000339634 | KIR2DL4 | 0.3263 |
| ENSP00000306275 | KCNK3 | 0.3263 |
| ENSP00000420182 | IQCJ-SCHIP1 | 0.3263 |
| ENSP00000349365 | IL27 | 0.3263 |
| ENSP00000330606 | HS3ST4 | 0.3263 |
| ENSP00000370826 | HR | 0.3263 |
| ENSP00000259951 | HLA-F | 0.3263 |
| ENSP00000368965 | HGSNAT | 0.3263 |
| ENSP00000357342 | HCN3 | 0.3263 |
| ENSP00000299314 | GNPTAB | 0.3263 |
| ENSP00000310770 | GLIPR1L1 | 0.3263 |
| ENSP00000286955 | FUT6 | 0.3263 |
| ENSP00000246549 | FFAR2 | 0.3263 |
| ENSP00000261275 | FAM189A1 | 0.3263 |
| ENSP00000251527 | ESYT2 | 0.3263 |
| ENSP00000253673 | EMR3 | 0.3263 |
| ENSP00000377430 | DPEP2 | 0.3263 |
| ENSP00000319255 | CHORDC1 | 0.3263 |
| ENSP00000378812 | CHCHD2 | 0.3263 |
| ENSP00000444052 | CES4A | 0.3263 |
| ENSP00000325681 | CCZ1 | 0.3263 |
| ENSP00000334996 | CCIN | 0.3263 |
| ENSP00000449960 | CALCOCO1 | 0.3263 |
| ENSP00000448598 | C17orf49 | 0.3263 |
| ENSP00000433157 | BBIP1 | 0.3263 |
| ENSP00000262919 | ATRN | 0.3263 |
| ENSP00000403302 | ANKRD36C | 0.3263 |
| ENSP00000405252 | AKAP7 | 0.3263 |
| ENSP00000268070 | ADAMTS17 | 0.3263 |
| ENSP00000265769 | ADAM28 | 0.3263 |
| ENSP00000221315 | ZNF432 | 0.3237 |
| ENSP00000228289 | ZNF268 | 0.3237 |
| ENSP00000287169 | ZDHHC5 | 0.3237 |
| ENSP00000295237 | XIRP2 | 0.3237 |
| ENSP00000346667 | WNK3 | 0.3237 |
| ENSP00000378857 | WDR6 | 0.3237 |
| ENSP00000254806 | WBP2 | 0.3237 |
| ENSP00000358470 | VTCN1 | 0.3237 |
| ENSP00000258398 | TTLL4 | 0.3237 |
| ENSP00000342499 | TSTD2 | 0.3237 |
| ENSP00000182290 | TSPAN32 | 0.3237 |
| ENSP00000332284 | TRIM69 | 0.3237 |
| ENSP00000215838 | TCN2 | 0.3237 |
| ENSP00000301244 | SPINT2 | 0.3237 |
| ENSP00000355577 | SLC35F3 | 0.3237 |
| ENSP00000356518 | SHCBP1L | 0.3237 |
| ENSP00000350633 | SERTAD1 | 0.3237 |
| ENSP00000284136 | SEMA3D | 0.3237 |
| ENSP00000383411 | SAMSN1 | 0.3237 |
| ENSP00000261441 | RSBN1 | 0.3237 |

| | | |
|---|---|---|
| ENSP00000378349 | RCN2 | 0.3237 |
| ENSP00000323074 | RAI1 | 0.3237 |
| ENSP00000271620 | PRUNE | 0.3237 |
| ENSP00000241808 | PRM2 | 0.3237 |
| ENSP00000282406 | PLEKHH2 | 0.3237 |
| ENSP00000367177 | PCDH8 | 0.3237 |
| ENSP00000304846 | OR2AT4 | 0.3237 |
| ENSP00000457628 | NUB1 | 0.3237 |
| ENSP00000327545 | NPTXR | 0.3237 |
| ENSP00000336894 | NAB1 | 0.3237 |
| ENSP00000278836 | MYRF | 0.3237 |
| ENSP00000247712 | MPV17L2 | 0.3237 |
| ENSP00000357123 | MNDA | 0.3237 |
| ENSP00000348308 | MMP23B | 0.3237 |
| ENSP00000330051 | MFSD6L | 0.3237 |
| ENSP00000373474 | METTL14 | 0.3237 |
| ENSP00000310593 | MAP9 | 0.3237 |
| ENSP00000244096 | MAGEA10 | 0.3237 |
| ENSP00000215886 | LGALS2 | 0.3237 |
| ENSP00000334187 | LCE1F | 0.3237 |
| ENSP00000299213 | LARP6 | 0.3237 |
| ENSP00000320917 | KRI1 | 0.3237 |
| ENSP00000363360 | INIP | 0.3237 |
| ENSP00000367848 | GABRD | 0.3237 |
| ENSP00000335651 | FSD2 | 0.3237 |
| ENSP00000300147 | ELFN2 | 0.3237 |
| ENSP00000365147 | EFHD2 | 0.3237 |
| ENSP00000359629 | CRTAC1 | 0.3237 |
| ENSP00000316605 | CNGB3 | 0.3237 |
| ENSP00000228438 | CLEC2B | 0.3237 |
| ENSP00000299752 | CDH16 | 0.3237 |
| ENSP00000296129 | CDCP1 | 0.3237 |
| ENSP00000426174 | CCDC169 | 0.3237 |
| ENSP00000297273 | CASD1 | 0.3237 |
| ENSP00000366662 | CA6 | 0.3237 |
| ENSP00000369339 | C9orf72 | 0.3237 |
| ENSP00000373509 | C11orf31 | 0.3237 |
| ENSP00000346251 | BPIFA1 | 0.3237 |
| ENSP00000320219 | ARSJ | 0.3237 |
| ENSP00000454770 | ANKRD66 | 0.3237 |
| ENSP00000360905 | AGBL4 | 0.3237 |
| ENSP00000298715 | VWA2 | 0.3237 |
| ENSP00000388902 | CCNJ | 0.3237 |
| ENSP00000301093 | ZNF701 | 0.3211 |
| ENSP00000380351 | ZNF682 | 0.3211 |
| ENSP00000301475 | ZNF558 | 0.3211 |
| ENSP00000470381 | ZNF468 | 0.3211 |
| ENSP00000411514 | ZNF385C | 0.3211 |
| ENSP00000301318 | ZFP28 | 0.3211 |
| ENSP00000368017 | ZFC3H1 | 0.3211 |
| ENSP00000323183 | ZBTB2 | 0.3211 |
| ENSP00000391088 | UNC80 | 0.3211 |
| ENSP00000265403 | UGT2B10 | 0.3211 |

| | | |
|---|---|---|
| ENSP00000358762 | UBE3D | 0.3211 |
| ENSP00000296015 | TTC14 | 0.3211 |
| ENSP00000318615 | TRIM73 | 0.3211 |
| ENSP00000304710 | TMC7 | 0.3211 |
| ENSP00000297784 | TMC1 | 0.3211 |
| ENSP00000341803 | TCTEX1D4 | 0.3211 |
| ENSP00000363441 | SYT15 | 0.3211 |
| ENSP00000348206 | SLMO2 | 0.3211 |
| ENSP00000259392 | SLC31A2 | 0.3211 |
| ENSP00000308895 | SLC19A1 | 0.3211 |
| ENSP00000362650 | SCAI | 0.3211 |
| ENSP00000256733 | SAA2 | 0.3211 |
| ENSP00000344489 | RNF133 | 0.3211 |
| ENSP00000377303 | RENBP | 0.3211 |
| ENSP00000376899 | PTGFRN | 0.3211 |
| ENSP00000310515 | PRM1 | 0.3211 |
| ENSP00000260113 | PI15 | 0.3211 |
| ENSP00000400958 | PHTF2 | 0.3211 |
| ENSP00000400895 | OTOGL | 0.3211 |
| ENSP00000330247 | NT5DC4 | 0.3211 |
| ENSP00000378089 | NMB | 0.3211 |
| ENSP00000369400 | NHS | 0.3211 |
| ENSP00000310998 | NAGPA | 0.3211 |
| ENSP00000382328 | N4BP2L2 | 0.3211 |
| ENSP00000327465 | MYT1 | 0.3211 |
| ENSP00000383333 | MORC3 | 0.3211 |
| ENSP00000358512 | MAB21L3 | 0.3211 |
| ENSP00000356143 | LRRN2 | 0.3211 |
| ENSP00000366028 | KLK9 | 0.3211 |
| ENSP00000351207 | KIAA1671 | 0.3211 |
| ENSP00000221980 | ICAM5 | 0.3211 |
| ENSP00000216106 | HMGXB4 | 0.3211 |
| ENSP00000370125 | HMGN1 | 0.3211 |
| ENSP00000356630 | HECA | 0.3211 |
| ENSP00000365596 | GPR183 | 0.3211 |
| ENSP00000453076 | GPR176 | 0.3211 |
| ENSP00000276077 | GPR174 | 0.3211 |
| ENSP00000265417 | GPR116 | 0.3211 |
| ENSP00000343234 | GAL3ST1 | 0.3211 |
| ENSP00000260257 | FDXACB1 | 0.3211 |
| ENSP00000362978 | EYA3 | 0.3211 |
| ENSP00000326411 | CWF19L1 | 0.3211 |
| ENSP00000245903 | CD70 | 0.3211 |
| ENSP00000312399 | CCDC63 | 0.3211 |
| ENSP00000367755 | CAMKMT | 0.3211 |
| ENSP00000178638 | CA12 | 0.3211 |
| ENSP00000343318 | B3GALT5 | 0.3211 |
| ENSP00000369996 | ATXN3L | 0.3211 |
| ENSP00000282185 | ATG10 | 0.3211 |
| ENSP00000347140 | ASGR2 | 0.3211 |
| ENSP00000346378 | APBB3 | 0.3211 |
| ENSP00000304586 | ANKS3 | 0.3211 |
| ENSP00000321731 | ANKRD24 | 0.3211 |

| | | |
|---|---|---|
| ENSP00000378328 | ANKRD13B | 0.3211 |
| ENSP00000353249 | AGBL5 | 0.3211 |
| ENSP00000286744 | ADAMTSL3 | 0.3211 |
| ENSP00000366240 | ABHD17B | 0.3211 |
| ENSP00000376135 | ZNF605 | 0.3184 |
| ENSP00000421258 | ZNF154 | 0.3184 |
| ENSP00000314153 | ZBTB33 | 0.3184 |
| ENSP00000340797 | TRIM3 | 0.3184 |
| ENSP00000302413 | TPST1 | 0.3184 |
| ENSP00000312304 | TPMT | 0.3184 |
| ENSP00000300433 | TMEM92 | 0.3184 |
| ENSP00000401513 | STRC | 0.3184 |
| ENSP00000315182 | SHQ1 | 0.3184 |
| ENSP00000249007 | RFPL3 | 0.3184 |
| ENSP00000298999 | R3HCC1L | 0.3184 |
| ENSP00000316675 | PVRIG | 0.3184 |
| ENSP00000257860 | PRPH | 0.3184 |
| ENSP00000358320 | POLR3GL | 0.3184 |
| ENSP00000367817 | PODXL | 0.3184 |
| ENSP00000294489 | PDPN | 0.3184 |
| ENSP00000329689 | OR10G7 | 0.3184 |
| ENSP00000267017 | NPFF | 0.3184 |
| ENSP00000358307 | NHLRC2 | 0.3184 |
| ENSP00000286479 | NAT2 | 0.3184 |
| ENSP00000349678 | MYBPHL | 0.3184 |
| ENSP00000412130 | MRVI1 | 0.3184 |
| ENSP00000382178 | MDGA2 | 0.3184 |
| ENSP00000402584 | MDGA1 | 0.3184 |
| ENSP00000368264 | MAGEB1 | 0.3184 |
| ENSP00000248668 | LRFN1 | 0.3184 |
| ENSP00000357773 | LCE2D | 0.3184 |
| ENSP00000257867 | LACRT | 0.3184 |
| ENSP00000306261 | KRT78 | 0.3184 |
| ENSP00000251993 | KIAA0930 | 0.3184 |
| ENSP00000327611 | KCNK12 | 0.3184 |
| ENSP00000244241 | IL17C | 0.3184 |
| ENSP00000410447 | IKZF2 | 0.3184 |
| ENSP00000449784 | HECTD4 | 0.3184 |
| ENSP00000312002 | HAVCR2 | 0.3184 |
| ENSP00000411827 | GPR137 | 0.3184 |
| ENSP00000317385 | GLIPR1L2 | 0.3184 |
| ENSP00000355827 | FBXO28 | 0.3184 |
| ENSP00000265978 | FAM160A2 | 0.3184 |
| ENSP00000342144 | EVC2 | 0.3184 |
| ENSP00000339115 | ESPNL | 0.3184 |
| ENSP00000221730 | EPHX3 | 0.3184 |
| ENSP00000296149 | ELP6 | 0.3184 |
| ENSP00000372160 | DOK6 | 0.3184 |
| ENSP00000331470 | DBX2 | 0.3184 |
| ENSP00000241312 | CSMD2 | 0.3184 |
| ENSP00000295728 | CRYBA2 | 0.3184 |
| ENSP00000329748 | CPNE8 | 0.3184 |
| ENSP00000314299 | CFHR1 | 0.3184 |

| | | |
|---|---|---|
| ENSP00000230053 | B3GAT2 | 0.3184 |
| ENSP00000369513 | ATP6AP1L | 0.3184 |
| ENSP00000355340 | APOBEC3C | 0.3184 |
| ENSP00000328515 | ZNF696 | 0.3158 |
| ENSP00000270617 | ZNF473 | 0.3158 |
| ENSP00000350773 | ZNF329 | 0.3158 |
| ENSP00000364798 | ZBTB40 | 0.3158 |
| ENSP00000336127 | TTC7B | 0.3158 |
| ENSP00000327738 | TRIML1 | 0.3158 |
| ENSP00000402595 | TRIM34 | 0.3158 |
| ENSP00000333441 | TREX2 | 0.3158 |
| ENSP00000261234 | TMEM5 | 0.3158 |
| ENSP00000344503 | TMEM201 | 0.3158 |
| ENSP00000291934 | TMEM190 | 0.3158 |
| ENSP00000254250 | THAP1 | 0.3158 |
| ENSP00000310193 | TBC1D10C | 0.3158 |
| ENSP00000290271 | STC1 | 0.3158 |
| ENSP00000318445 | ST3GAL1 | 0.3158 |
| ENSP00000346537 | SMOC2 | 0.3158 |
| ENSP00000297195 | SLC29A4 | 0.3158 |
| ENSP00000334594 | SLC10A7 | 0.3158 |
| ENSP00000368165 | SDHAF1 | 0.3158 |
| ENSP00000362894 | RTKN2 | 0.3158 |
| ENSP00000355899 | RRP15 | 0.3158 |
| ENSP00000244061 | RNF114 | 0.3158 |
| ENSP00000216085 | RHBDD3 | 0.3158 |
| ENSP00000305696 | RBFA | 0.3158 |
| ENSP00000417658 | PTCHD3 | 0.3158 |
| ENSP00000365694 | PPP1R10 | 0.3158 |
| ENSP00000222572 | PON2 | 0.3158 |
| ENSP00000244221 | PAIP2B | 0.3158 |
| ENSP00000347003 | OR5K4 | 0.3158 |
| ENSP00000277309 | OR1K1 | 0.3158 |
| ENSP00000398290 | NDUFA3 | 0.3158 |
| ENSP00000364407 | METTL8 | 0.3158 |
| ENSP00000351669 | MANEA | 0.3158 |
| ENSP00000347358 | MAGEA11 | 0.3158 |
| ENSP00000263177 | LPPR5 | 0.3158 |
| ENSP00000260702 | LOXL4 | 0.3158 |
| ENSP00000357757 | LCE1C | 0.3158 |
| ENSP00000247194 | L3HYPDH | 0.3158 |
| ENSP00000383672 | KIR3DS1 | 0.3158 |
| ENSP00000401878 | KBTBD8 | 0.3158 |
| ENSP00000238647 | IRF2BPL | 0.3158 |
| ENSP00000342520 | IL17REL | 0.3158 |
| ENSP00000364371 | HIATL2 | 0.3158 |
| ENSP00000342118 | HHIPL2 | 0.3158 |
| ENSP00000216338 | GZMH | 0.3158 |
| ENSP00000361207 | GHITM | 0.3158 |
| ENSP00000337512 | FTSJD1 | 0.3158 |
| ENSP00000319897 | FAM214B | 0.3158 |
| ENSP00000377566 | ELMO3 | 0.3158 |
| ENSP00000262593 | DOK5 | 0.3158 |

| | | |
|---|---|---|
| ENSP00000350266 | DENND2D | 0.3158 |
| ENSP00000330488 | CXorf38 | 0.3158 |
| ENSP00000330523 | CTHRC1 | 0.3158 |
| ENSP00000345079 | CSRP1 | 0.3158 |
| ENSP00000315203 | CREG2 | 0.3158 |
| ENSP00000359540 | CREG1 | 0.3158 |
| ENSP00000354255 | CPZ | 0.3158 |
| ENSP00000252599 | COLGALT1 | 0.3158 |
| ENSP00000327599 | CLEC4G | 0.3158 |
| ENSP00000408631 | CIDEC | 0.3158 |
| ENSP00000307911 | CHST2 | 0.3158 |
| ENSP00000312663 | CHRNA9 | 0.3158 |
| ENSP00000393854 | CENPBD1 | 0.3158 |
| ENSP00000363139 | CD164L2 | 0.3158 |
| ENSP00000387641 | CAND2 | 0.3158 |
| ENSP00000381270 | C6orf170 | 0.3158 |
| ENSP00000274000 | AP1AR | 0.3158 |
| ENSP00000254835 | ANKRD20A3 | 0.3158 |
| ENSP00000215479 | AMELY | 0.3158 |
| ENSP00000422554 | ADAMTS12 | 0.3158 |
| ENSP00000375413 | AC011841.1 | 0.3158 |
| ENSP00000299698 | A2ML1 | 0.3158 |
| ENSP00000223210 | ZNF862 | 0.3132 |
| ENSP00000352444 | ZNF33B | 0.3132 |
| ENSP00000299667 | ZNF3 | 0.3132 |
| ENSP00000263095 | ZNF264 | 0.3132 |
| ENSP00000345809 | ZNF197 | 0.3132 |
| ENSP00000323148 | ZKSCAN1 | 0.3132 |
| ENSP00000370242 | WWC3 | 0.3132 |
| ENSP00000393183 | VPRBP | 0.3132 |
| ENSP00000357556 | VENTX | 0.3132 |
| ENSP00000244709 | TREM1 | 0.3132 |
| ENSP00000343577 | TPSAB1 | 0.3132 |
| ENSP00000294309 | TPCN2 | 0.3132 |
| ENSP00000298351 | TMEM63C | 0.3132 |
| ENSP00000305892 | TMEM208 | 0.3132 |
| ENSP00000347444 | TDRD7 | 0.3132 |
| ENSP00000418994 | SYNPR | 0.3132 |
| ENSP00000338742 | SULT1A2 | 0.3132 |
| ENSP00000359070 | STMN3 | 0.3132 |
| ENSP00000369419 | STEAP4 | 0.3132 |
| ENSP00000270570 | SLC47A1 | 0.3132 |
| ENSP00000363329 | SLC31A1 | 0.3132 |
| ENSP00000460571 | RP1-4G17.5 | 0.3132 |
| ENSP00000255006 | RIN2 | 0.3132 |
| ENSP00000304139 | RBMXL2 | 0.3132 |
| ENSP00000339692 | RASSF4 | 0.3132 |
| ENSP00000333490 | PRHOXNB | 0.3132 |
| ENSP00000397947 | PLEKHA8 | 0.3132 |
| ENSP00000281382 | PIGF | 0.3132 |
| ENSP00000348752 | ORAI2 | 0.3132 |
| ENSP00000329467 | OR4M2 | 0.3132 |
| ENSP00000300127 | OR4D6 | 0.3132 |

| | | |
|---|---|---|
| ENSP00000352717 | OR13G1 | 0.3132 |
| ENSP00000356191 | OPTC | 0.3132 |
| ENSP00000273598 | NICN1 | 0.3132 |
| ENSP00000379394 | NAV2 | 0.3132 |
| ENSP00000347486 | MORN3 | 0.3132 |
| ENSP00000357776 | LCE3D | 0.3132 |
| ENSP00000406970 | LAX1 | 0.3132 |
| ENSP00000360676 | KTI12 | 0.3132 |
| ENSP00000445768 | KIAA1147 | 0.3132 |
| ENSP00000282146 | KCNK13 | 0.3132 |
| ENSP00000323587 | ITLN1 | 0.3132 |
| ENSP00000415900 | IL31RA | 0.3132 |
| ENSP00000359581 | HS2ST1 | 0.3132 |
| ENSP00000344844 | HAVCR1 | 0.3132 |
| ENSP00000355799 | GPR31 | 0.3132 |
| ENSP00000308479 | GPR171 | 0.3132 |
| ENSP00000276218 | GPR119 | 0.3132 |
| ENSP00000369895 | GEMIN8 | 0.3132 |
| ENSP00000265000 | GALNT7 | 0.3132 |
| ENSP00000297107 | GALNT10 | 0.3132 |
| ENSP00000265294 | GABRP | 0.3132 |
| ENSP00000331912 | GABRG3 | 0.3132 |
| ENSP00000037869 | FAM136A | 0.3132 |
| ENSP00000297258 | FABP5 | 0.3132 |
| ENSP00000326342 | ELMOD2 | 0.3132 |
| ENSP00000250454 | EAPP | 0.3132 |
| ENSP00000352785 | DSG4 | 0.3132 |
| ENSP00000371835 | DEFB126 | 0.3132 |
| ENSP00000320951 | DEFB103A | 0.3132 |
| ENSP00000371682 | DCAF16 | 0.3132 |
| ENSP00000349204 | CRB3 | 0.3132 |
| ENSP00000336994 | CLPTM1 | 0.3132 |
| ENSP00000338728 | CCDC88A | 0.3132 |
| ENSP00000084798 | CA11 | 0.3132 |
| ENSP00000272139 | C1orf35 | 0.3132 |
| ENSP00000219139 | C16orf70 | 0.3132 |
| ENSP00000394316 | BZW1 | 0.3132 |
| ENSP00000260442 | BCL2L10 | 0.3132 |
| ENSP00000316173 | B3GNT5 | 0.3132 |
| ENSP00000359660 | AVPI1 | 0.3132 |
| ENSP00000319778 | AURKAIP1 | 0.3132 |
| ENSP00000366697 | ANKRD20A1 | 0.3132 |
| ENSP00000380557 | AKAP8L | 0.3132 |
| ENSP00000325519 | AC020907.1 | 0.3132 |
| ENSP00000327691 | HMGN4 | 0.3132 |
| ENSP00000302222 | ZNF25 | 0.3105 |
| ENSP00000423820 | ZFP62 | 0.3105 |
| ENSP00000371559 | TSSC1 | 0.3105 |
| ENSP00000334657 | TRIM46 | 0.3105 |
| ENSP00000234798 | TPSG1 | 0.3105 |
| ENSP00000259339 | TOR1B | 0.3105 |
| ENSP00000246024 | TMX4 | 0.3105 |
| ENSP00000444565 | TMUB2 | 0.3105 |

| | | |
|---|---|---|
| ENSP00000351737 | TMEM126B | 0.3105 |
| ENSP00000350630 | TLL2 | 0.3105 |
| ENSP00000271064 | TINAGL1 | 0.3105 |
| ENSP00000386538 | TBC1D10B | 0.3105 |
| ENSP00000287908 | STEAP2 | 0.3105 |
| ENSP00000312946 | STAB1 | 0.3105 |
| ENSP00000291839 | ST6GALNAC6 | 0.3105 |
| ENSP00000274565 | SPINK7 | 0.3105 |
| ENSP00000369407 | SLC25A35 | 0.3105 |
| ENSP00000340505 | SENP8 | 0.3105 |
| ENSP00000361557 | RBM41 | 0.3105 |
| ENSP00000360112 | RAVER2 | 0.3105 |
| ENSP00000319730 | PRSS8 | 0.3105 |
| ENSP00000355719 | PRSS38 | 0.3105 |
| ENSP00000356278 | PHLDA3 | 0.3105 |
| ENSP00000278243 | PGAP2 | 0.3105 |
| ENSP00000470539 | PET100 | 0.3105 |
| ENSP00000306688 | OR2M4 | 0.3105 |
| ENSP00000294794 | OLFML2B | 0.3105 |
| ENSP00000351650 | NUDT3 | 0.3105 |
| ENSP00000295461 | NIPAL1 | 0.3105 |
| ENSP00000343847 | NEK10 | 0.3105 |
| ENSP00000240050 | MTERFD3 | 0.3105 |
| ENSP00000311827 | MSL2 | 0.3105 |
| ENSP00000356940 | MOXD1 | 0.3105 |
| ENSP00000347896 | MBD6 | 0.3105 |
| ENSP00000334375 | LRCH3 | 0.3105 |
| ENSP00000352962 | LPPR3 | 0.3105 |
| ENSP00000360472 | LGI1 | 0.3105 |
| ENSP00000379034 | KLHDC7B | 0.3105 |
| ENSP00000324742 | IL32 | 0.3105 |
| ENSP00000299157 | IKBIP | 0.3105 |
| ENSP00000263863 | GNLY | 0.3105 |
| ENSP00000268695 | GALNS | 0.3105 |
| ENSP00000221347 | FCGBP | 0.3105 |
| ENSP00000247977 | FBXL12 | 0.3105 |
| ENSP00000300030 | FAM96A | 0.3105 |
| ENSP00000351259 | FAM21B | 0.3105 |
| ENSP00000298492 | FAM175B | 0.3105 |
| ENSP00000353393 | F8 | 0.3105 |
| ENSP00000359131 | EXTL2 | 0.3105 |
| ENSP00000378254 | EML3 | 0.3105 |
| ENSP00000356898 | DDR2 | 0.3105 |
| ENSP00000373343 | CPNE9 | 0.3105 |
| ENSP00000386962 | CATSPERG | 0.3105 |
| ENSP00000363298 | BSPRY | 0.3105 |
| ENSP00000357927 | BNIPL | 0.3105 |
| ENSP00000287590 | B3GNT7 | 0.3105 |
| ENSP00000321874 | B3GNT3 | 0.3105 |
| ENSP00000275699 | ASB15 | 0.3105 |
| ENSP00000256389 | ADAM20 | 0.3105 |
| ENSP00000414066 | ACN9 | 0.3105 |
| ENSP00000471569 | GABRR3 | 0.3105 |

| | | |
|---|---|---|
| ENSP00000359606 | ZNHIT6 | 0.3079 |
| ENSP00000324598 | ZNF540 | 0.3079 |
| ENSP00000315173 | ZNF41 | 0.3079 |
| ENSP00000397163 | ZNF286A | 0.3079 |
| ENSP00000349796 | ZNF23 | 0.3079 |
| ENSP00000348848 | WDR45 | 0.3079 |
| ENSP00000347915 | TTC30A | 0.3079 |
| ENSP00000339820 | TSPAN18 | 0.3079 |
| ENSP00000362387 | TSPAN15 | 0.3079 |
| ENSP00000236979 | TNP1 | 0.3079 |
| ENSP00000321038 | TMEM86B | 0.3079 |
| ENSP00000234831 | TMEM59 | 0.3079 |
| ENSP00000363463 | TMEM57 | 0.3079 |
| ENSP00000338624 | TASP1 | 0.3079 |
| ENSP00000333310 | SULT1C3 | 0.3079 |
| ENSP00000367139 | SPINK9 | 0.3079 |
| ENSP00000322649 | SLC25A41 | 0.3079 |
| ENSP00000358515 | SLC22A15 | 0.3079 |
| ENSP00000273861 | SLC10A4 | 0.3079 |
| ENSP00000321077 | SIGLEC8 | 0.3079 |
| ENSP00000323328 | SIGLEC7 | 0.3079 |
| ENSP00000265807 | SH2D4A | 0.3079 |
| ENSP00000238508 | SERPINB10 | 0.3079 |
| ENSP00000292641 | SCGB3A1 | 0.3079 |
| ENSP00000388920 | RWDD4 | 0.3079 |
| ENSP00000435342 | RTL1 | 0.3079 |
| ENSP00000358064 | RPRD2 | 0.3079 |
| ENSP00000443772 | RP11-87C12.2 | 0.3079 |
| ENSP00000352734 | RNF217 | 0.3079 |
| ENSP00000299563 | RNF169 | 0.3079 |
| ENSP00000268122 | RHCG | 0.3079 |
| ENSP00000310471 | RBM4B | 0.3079 |
| ENSP00000377948 | QRFPR | 0.3079 |
| ENSP00000345487 | QRFP | 0.3079 |
| ENSP00000325958 | PTPMT1 | 0.3079 |
| ENSP00000353646 | PLEKHG4 | 0.3079 |
| ENSP00000428288 | PCDHGB4 | 0.3079 |
| ENSP00000306100 | PAM | 0.3079 |
| ENSP00000365678 | OTUD1 | 0.3079 |
| ENSP00000332500 | OR4N4 | 0.3079 |
| ENSP00000233027 | NEK4 | 0.3079 |
| ENSP00000250144 | MMP28 | 0.3079 |
| ENSP00000384690 | MMD2 | 0.3079 |
| ENSP00000285599 | MAN2B2 | 0.3079 |
| ENSP00000287748 | LYZL4 | 0.3079 |
| ENSP00000338727 | LRRFIP2 | 0.3079 |
| ENSP00000354001 | KRTAP12-2 | 0.3079 |
| ENSP00000377570 | KRT34 | 0.3079 |
| ENSP00000470555 | KLK13 | 0.3079 |
| ENSP00000262032 | IKZF4 | 0.3079 |
| ENSP00000268389 | IGSF6 | 0.3079 |
| ENSP00000246896 | HTN1 | 0.3079 |
| ENSP00000381220 | HDDC2 | 0.3079 |

| | | |
|---|---|---|
| ENSP00000412315 | GSKIP | 0.3079 |
| ENSP00000353465 | GSDMB | 0.3079 |
| ENSP00000297146 | GPR85 | 0.3079 |
| ENSP00000361110 | GBGT1 | 0.3079 |
| ENSP00000417509 | FAM208A | 0.3079 |
| ENSP00000395249 | FAM134A | 0.3079 |
| ENSP00000277165 | FAM120A | 0.3079 |
| ENSP00000296380 | EXO5 | 0.3079 |
| ENSP00000350336 | DSCR8 | 0.3079 |
| ENSP00000262424 | CRISPLD2 | 0.3079 |
| ENSP00000291495 | CILP2 | 0.3079 |
| ENSP00000281882 | CFC1B | 0.3079 |
| ENSP00000321005 | CD300LG | 0.3079 |
| ENSP00000409197 | CCL14 | 0.3079 |
| ENSP00000400592 | CASP16 | 0.3079 |
| ENSP00000290363 | C1orf51 | 0.3079 |
| ENSP00000244519 | BTN3A3 | 0.3079 |
| ENSP00000261658 | BFAR | 0.3079 |
| ENSP00000319062 | BBS12 | 0.3079 |
| ENSP00000368528 | APOO | 0.3079 |
| ENSP00000196548 | ZNF8 | 0.3053 |
| ENSP00000396857 | ZNF697 | 0.3053 |
| ENSP00000312277 | ZNF507 | 0.3053 |
| ENSP00000219478 | ZNF500 | 0.3053 |
| ENSP00000311183 | ZNF497 | 0.3053 |
| ENSP00000326249 | ZNF454 | 0.3053 |
| ENSP00000252979 | ZNF337 | 0.3053 |
| ENSP00000343140 | XIRP1 | 0.3053 |
| ENSP00000013070 | UBR7 | 0.3053 |
| ENSP00000272395 | TRIM43 | 0.3053 |
| ENSP00000397773 | TMEM50B | 0.3053 |
| ENSP00000253047 | TMEM160 | 0.3053 |
| ENSP00000261226 | TMCC3 | 0.3053 |
| ENSP00000275198 | TAAR6 | 0.3053 |
| ENSP00000346130 | SVIP | 0.3053 |
| ENSP00000417583 | ST6GALNAC5 | 0.3053 |
| ENSP00000237536 | SOGA1 | 0.3053 |
| ENSP00000236495 | SLC5A9 | 0.3053 |
| ENSP00000379008 | SLC5A10 | 0.3053 |
| ENSP00000350475 | SLC4A5 | 0.3053 |
| ENSP00000428316 | SERPINE3 | 0.3053 |
| ENSP00000383155 | SELM | 0.3053 |
| ENSP00000353094 | SDF4 | 0.3053 |
| ENSP00000295704 | RNF25 | 0.3053 |
| ENSP00000006777 | RHBDD2 | 0.3053 |
| ENSP00000340610 | POTEH | 0.3053 |
| ENSP00000308315 | PODN | 0.3053 |
| ENSP00000468678 | PLEKHH3 | 0.3053 |
| ENSP00000429273 | PCDHGB1 | 0.3053 |
| ENSP00000380153 | OR51E2 | 0.3053 |
| ENSP00000290291 | OGFR | 0.3053 |
| ENSP00000423014 | OCIAD2 | 0.3053 |
| ENSP00000363894 | NIPSNAP3B | 0.3053 |

| | | |
|---|---|---|
| ENSP00000309782 | MRGPRF | 0.3053 |
| ENSP00000347821 | MORC4 | 0.3053 |
| ENSP00000265026 | MAP3K13 | 0.3053 |
| ENSP00000301046 | LALBA | 0.3053 |
| ENSP00000203629 | LAG3 | 0.3053 |
| ENSP00000353742 | KRTAP26-1 | 0.3053 |
| ENSP00000250351 | KLK12 | 0.3053 |
| ENSP00000311746 | KLK10 | 0.3053 |
| ENSP00000358265 | KLHL32 | 0.3053 |
| ENSP00000420659 | KLHL29 | 0.3053 |
| ENSP00000257572 | HRK | 0.3053 |
| ENSP00000362746 | GPR21 | 0.3053 |
| ENSP00000273352 | GPR128 | 0.3053 |
| ENSP00000370767 | GP2 | 0.3053 |
| ENSP00000454322 | GOLGA8F | 0.3053 |
| ENSP00000408132 | GOLGA6B | 0.3053 |
| ENSP00000354053 | GLT8D2 | 0.3053 |
| ENSP00000302552 | GLOD5 | 0.3053 |
| ENSP00000334940 | GGN | 0.3053 |
| ENSP00000384742 | GAGE2E | 0.3053 |
| ENSP00000369604 | FBXO16 | 0.3053 |
| ENSP00000394405 | FAM213B | 0.3053 |
| ENSP00000355264 | FAM111A | 0.3053 |
| ENSP00000182096 | CRYBG3 | 0.3053 |
| ENSP00000374562 | COX16 | 0.3053 |
| ENSP00000348054 | COMMD6 | 0.3053 |
| ENSP00000335605 | CMTM7 | 0.3053 |
| ENSP00000249806 | CLN6 | 0.3053 |
| ENSP00000261944 | CDHR2 | 0.3053 |
| ENSP00000291691 | C21orf58 | 0.3053 |
| ENSP00000286732 | C15orf26 | 0.3053 |
| ENSP00000421169 | BEND4 | 0.3053 |
| ENSP00000363575 | FXYD4 | 0.3053 |
| ENSP00000379684 | ZNF813 | 0.3026 |
| ENSP00000301744 | ZNF597 | 0.3026 |
| ENSP00000301399 | ZNF577 | 0.3026 |
| ENSP00000247956 | ZNF317 | 0.3026 |
| ENSP00000337724 | ZNF202 | 0.3026 |
| ENSP00000363344 | ZFP37 | 0.3026 |
| ENSP00000361748 | ZDHHC12 | 0.3026 |
| ENSP00000324763 | WFIKKN1 | 0.3026 |
| ENSP00000254442 | WDR7 | 0.3026 |
| ENSP00000365536 | WDR45 | 0.3026 |
| ENSP00000285805 | TRIM74 | 0.3026 |
| ENSP00000402414 | TRIM48 | 0.3026 |
| ENSP00000376227 | TMEM44 | 0.3026 |
| ENSP00000275767 | TMEM140 | 0.3026 |
| ENSP00000408581 | TMEM132D | 0.3026 |
| ENSP00000416050 | TM2D2 | 0.3026 |
| ENSP00000315630 | SWAP70 | 0.3026 |
| ENSP00000339221 | SULT1A4 | 0.3026 |
| ENSP00000377717 | ST3GAL6 | 0.3026 |
| ENSP00000298396 | SSX3 | 0.3026 |

| | | |
|---|---|---|
| ENSP00000387266 | SPIRE1 | 0.3026 |
| ENSP00000355900 | SPATA17 | 0.3026 |
| ENSP00000329287 | SNN | 0.3026 |
| ENSP00000326671 | SLC47A2 | 0.3026 |
| ENSP00000306328 | SLC25A33 | 0.3026 |
| ENSP00000340402 | SLC15A5 | 0.3026 |
| ENSP00000269491 | SERPINB12 | 0.3026 |
| ENSP00000387187 | SERF2 | 0.3026 |
| ENSP00000377751 | SCOC | 0.3026 |
| ENSP00000307275 | SCAMP3 | 0.3026 |
| ENSP00000344822 | S100A13 | 0.3026 |
| ENSP00000334134 | RGS9BP | 0.3026 |
| ENSP00000224600 | RBP3 | 0.3026 |
| ENSP00000317445 | PSAPL1 | 0.3026 |
| ENSP00000293851 | PRSS33 | 0.3026 |
| ENSP00000344961 | PLEKHG7 | 0.3026 |
| ENSP00000253255 | PKDREJ | 0.3026 |
| ENSP00000362643 | PHKA1 | 0.3026 |
| ENSP00000387523 | OR7E24 | 0.3026 |
| ENSP00000232603 | MORC1 | 0.3026 |
| ENSP00000363348 | MNF1 | 0.3026 |
| ENSP00000393099 | MAPKBP1 | 0.3026 |
| ENSP00000362244 | MAP7D1 | 0.3026 |
| ENSP00000350592 | MAGEA12 | 0.3026 |
| ENSP00000472039 | LSP1 | 0.3026 |
| ENSP00000338887 | LRRC8D | 0.3026 |
| ENSP00000353346 | LHFPL5 | 0.3026 |
| ENSP00000257901 | KRT85 | 0.3026 |
| ENSP00000342710 | KRT77 | 0.3026 |
| ENSP00000314608 | KLHL11 | 0.3026 |
| ENSP00000370842 | IL33 | 0.3026 |
| ENSP00000267015 | GPR84 | 0.3026 |
| ENSP00000458130 | GOLGA8G | 0.3026 |
| ENSP00000403802 | FBXO10 | 0.3026 |
| ENSP00000236980 | FASTKD2 | 0.3026 |
| ENSP00000373360 | FAM131A | 0.3026 |
| ENSP00000334314 | EML1 | 0.3026 |
| ENSP00000376952 | EME1 | 0.3026 |
| ENSP00000303532 | DEFB4A | 0.3026 |
| ENSP00000365492 | DEFB119 | 0.3026 |
| ENSP00000346483 | DDRGK1 | 0.3026 |
| ENSP00000295542 | DCST1 | 0.3026 |
| ENSP00000282018 | CYSLTR2 | 0.3026 |
| ENSP00000278980 | COMMD7 | 0.3026 |
| ENSP00000323782 | CCDC43 | 0.3026 |
| ENSP00000348933 | CCDC25 | 0.3026 |
| ENSP00000350011 | C7orf50 | 0.3026 |
| ENSP00000278174 | BTBD10 | 0.3026 |
| ENSP00000359045 | ARHGAP4 | 0.3026 |
| ENSP00000262219 | ANXA13 | 0.3026 |
| ENSP00000409126 | ANO6 | 0.3026 |
| ENSP00000296657 | ANKRD33B | 0.3026 |
| ENSP00000175238 | ADAM7 | 0.3026 |

| | | |
|---|---|---|
| ENSP00000380254 | AC007390.5 | 0.3026 |
| ENSP00000317614 | ZNF518B | 0.3000 |
| ENSP00000375656 | ZNF321P | 0.3000 |
| ENSP00000278319 | ZNF215 | 0.3000 |
| ENSP00000245663 | ZBTB46 | 0.3000 |
| ENSP00000250101 | TXNDC17 | 0.3000 |
| ENSP00000434279 | TMPRSS13 | 0.3000 |
| ENSP00000274532 | TIMD4 | 0.3000 |
| ENSP00000321343 | ST8SIA5 | 0.3000 |
| ENSP00000320431 | ST8SIA3 | 0.3000 |
| ENSP00000376423 | SPRR2A | 0.3000 |
| ENSP00000264694 | SNX25 | 0.3000 |
| ENSP00000299709 | SLC38A8 | 0.3000 |
| ENSP00000376150 | SLC16A3 | 0.3000 |
| ENSP00000276033 | SLC16A2 | 0.3000 |
| ENSP00000418813 | SELK | 0.3000 |
| ENSP00000342935 | SDCBP2 | 0.3000 |
| ENSP00000354733 | SBNO2 | 0.3000 |
| ENSP00000335397 | RTN4RL2 | 0.3000 |
| ENSP00000308193 | RNASEH2C | 0.3000 |
| ENSP00000356329 | RAET1G | 0.3000 |
| ENSP00000317903 | R3HDM2 | 0.3000 |
| ENSP00000234453 | PLEKHA3 | 0.3000 |
| ENSP00000346802 | PCDHB11 | 0.3000 |
| ENSP00000330384 | OR6A2 | 0.3000 |
| ENSP00000322273 | OLFML3 | 0.3000 |
| ENSP00000308717 | MRPL48 | 0.3000 |
| ENSP00000332124 | MRC1L1 | 0.3000 |
| ENSP00000304553 | MPLKIP | 0.3000 |
| ENSP00000445859 | MPHOSPH9 | 0.3000 |
| ENSP00000245615 | MBOAT7 | 0.3000 |
| ENSP00000395962 | MAGED4B | 0.3000 |
| ENSP00000368266 | MAGEB4 | 0.3000 |
| ENSP00000367891 | LGALS7 | 0.3000 |
| ENSP00000375236 | KRTAP4-7 | 0.3000 |
| ENSP00000331242 | KREMEN1 | 0.3000 |
| ENSP00000386787 | KIAA0922 | 0.3000 |
| ENSP00000379441 | JMY | 0.3000 |
| ENSP00000264952 | GRK7 | 0.3000 |
| ENSP00000335158 | GPR142 | 0.3000 |
| ENSP00000170564 | GPATCH1 | 0.3000 |
| ENSP00000246553 | FFAR1 | 0.3000 |
| ENSP00000337510 | FBXO40 | 0.3000 |
| ENSP00000386895 | FAM124B | 0.3000 |
| ENSP00000472280 | EMR2 | 0.3000 |
| ENSP00000365103 | DAOA | 0.3000 |
| ENSP00000319141 | CYBRD1 | 0.3000 |
| ENSP00000313759 | CTC1 | 0.3000 |
| ENSP00000370021 | CNTLN | 0.3000 |
| ENSP00000206423 | CCDC80 | 0.3000 |
| ENSP00000416797 | CAMSAP3 | 0.3000 |
| ENSP00000297623 | C9orf24 | 0.3000 |
| ENSP00000295898 | C4orf36 | 0.3000 |

| | | |
|---|---|---|
| ENSP00000245957 | C20orf26 | 0.3000 |
| ENSP00000318883 | BCL2L13 | 0.3000 |
| ENSP00000286918 | ANKRD9 | 0.3000 |
| ENSP00000251089 | ANGEL1 | 0.3000 |
| ENSP00000252744 | ZSWIM6 | 0.2974 |
| ENSP00000330838 | ZNF775 | 0.2974 |
| ENSP00000375598 | ZNF628 | 0.2974 |
| ENSP00000306869 | ZNF565 | 0.2974 |
| ENSP00000472802 | ZNF446 | 0.2974 |
| ENSP00000340494 | ZNF395 | 0.2974 |
| ENSP00000252211 | ZKSCAN3 | 0.2974 |
| ENSP00000442373 | ZDHHC11B | 0.2974 |
| ENSP00000339918 | XRRA1 | 0.2974 |
| ENSP00000326379 | VWA9 | 0.2974 |
| ENSP00000374501 | UVSSA | 0.2974 |
| ENSP00000221399 | TULP2 | 0.2974 |
| ENSP00000395086 | TRIM51 | 0.2974 |
| ENSP00000256649 | TRIM45 | 0.2974 |
| ENSP00000339659 | TRIM2 | 0.2974 |
| ENSP00000335144 | TPGS2 | 0.2974 |
| ENSP00000359222 | TMEM56 | 0.2974 |
| ENSP00000358430 | TECTB | 0.2974 |
| ENSP00000366549 | ST3GAL5 | 0.2974 |
| ENSP00000345477 | ST3GAL2 | 0.2974 |
| ENSP00000381480 | SPAG11B | 0.2974 |
| ENSP00000323435 | SNX22 | 0.2974 |
| ENSP00000273158 | SLC25A38 | 0.2974 |
| ENSP00000307369 | SHE | 0.2974 |
| ENSP00000441927 | SESN3 | 0.2974 |
| ENSP00000302579 | SCEL | 0.2974 |
| ENSP00000352427 | RGR | 0.2974 |
| ENSP00000383096 | RFPL2 | 0.2974 |
| ENSP00000270645 | RCN3 | 0.2974 |
| ENSP00000295588 | POGLUT1 | 0.2974 |
| ENSP00000429601 | PCDHGA6 | 0.2974 |
| ENSP00000239450 | PCDHB12 | 0.2974 |
| ENSP00000394936 | ORM2 | 0.2974 |
| ENSP00000376615 | NT5DC3 | 0.2974 |
| ENSP00000259953 | NRM | 0.2974 |
| ENSP00000357798 | MMP21 | 0.2974 |
| ENSP00000273286 | LRTM1 | 0.2974 |
| ENSP00000339621 | LCN6 | 0.2974 |
| ENSP00000261588 | KIAA0556 | 0.2974 |
| ENSP00000258180 | KIAA0513 | 0.2974 |
| ENSP00000345667 | ILDR1 | 0.2974 |
| ENSP00000407107 | HHLA1 | 0.2974 |
| ENSP00000384582 | GPR98 | 0.2974 |
| ENSP00000399563 | GPR89C | 0.2974 |
| ENSP00000378195 | GPR75 | 0.2974 |
| ENSP00000284311 | GPR15 | 0.2974 |
| ENSP00000417354 | GOLIM4 | 0.2974 |
| ENSP00000287957 | GATAD1 | 0.2974 |
| ENSP00000357540 | FUOM | 0.2974 |

| | | |
|---|---|---|
| ENSP00000365569 | FLOT1 | 0.2974 |
| ENSP00000359410 | EPHX4 | 0.2974 |
| ENSP00000306105 | CEP89 | 0.2974 |
| ENSP00000361189 | CDHR1 | 0.2974 |
| ENSP00000309376 | CD164 | 0.2974 |
| ENSP00000306522 | CAMTA1 | 0.2974 |
| ENSP00000262947 | C19orf10 | 0.2974 |
| ENSP00000300091 | C18orf54 | 0.2974 |
| ENSP00000349143 | BTN2A2 | 0.2974 |
| ENSP00000386121 | BTBD9 | 0.2974 |
| ENSP00000253354 | BPIFB1 | 0.2974 |
| ENSP00000319974 | BBX | 0.2974 |
| ENSP00000281437 | BARX2 | 0.2974 |
| ENSP00000472450 | BAGE5 | 0.2974 |
| ENSP00000312700 | B3GNT8 | 0.2974 |
| ENSP00000319636 | B3GNT4 | 0.2974 |
| ENSP00000298815 | ARHGAP42 | 0.2974 |
| ENSP00000301945 | ANTXR1 | 0.2974 |
| ENSP00000342295 | ANKRD55 | 0.2974 |
| ENSP00000351875 | ANKRD30B | 0.2974 |
| ENSP00000343021 | ALKBH2 | 0.2974 |
| ENSP00000258884 | ABHD17C | 0.2974 |
| ENSP00000324450 | ZSCAN20 | 0.2974 |
| ENSP00000319053 | ZNF77 | 0.2947 |
| ENSP00000301480 | ZNF560 | 0.2947 |
| ENSP00000357323 | YY1AP1 | 0.2947 |
| ENSP00000282507 | UGT3A2 | 0.2947 |
| ENSP00000321987 | TSPEAR | 0.2947 |
| ENSP00000267970 | TSPAN3 | 0.2947 |
| ENSP00000282369 | TRIM36 | 0.2947 |
| ENSP00000385470 | TNFSF18 | 0.2947 |
| ENSP00000295694 | TMEM79 | 0.2947 |
| ENSP00000358948 | TEX28P1 | 0.2947 |
| ENSP00000380779 | TCTN1 | 0.2947 |
| ENSP00000352463 | TCF20 | 0.2947 |
| ENSP00000356470 | SWT1 | 0.2947 |
| ENSP00000427401 | SREK1IP1 | 0.2947 |
| ENSP00000365830 | SOWAHC | 0.2947 |
| ENSP00000338988 | SLMO1 | 0.2947 |
| ENSP00000336887 | SLC39A9 | 0.2947 |
| ENSP00000348211 | SLC25A46 | 0.2947 |
| ENSP00000386348 | SAPCD2 | 0.2947 |
| ENSP00000457682 | RP11-458D21.5 | 0.2947 |
| ENSP00000340578 | RASSF6 | 0.2947 |
| ENSP00000306390 | PRSS27 | 0.2947 |
| ENSP00000420686 | PLCXD2 | 0.2947 |
| ENSP00000258042 | NMBR | 0.2947 |
| ENSP00000321976 | NKPD1 | 0.2947 |
| ENSP00000298687 | NDRG2 | 0.2947 |
| ENSP00000363557 | MYOM3 | 0.2947 |
| ENSP00000380531 | MRPS34 | 0.2947 |
| ENSP00000354415 | MICU1 | 0.2947 |
| ENSP00000341543 | METTL23 | 0.2947 |

| | | |
|---|---|---|
| ENSP00000343298 | LRRC41 | 0.2947 |
| ENSP00000339251 | KRTDAP | 0.2947 |
| ENSP00000361373 | KLF17 | 0.2947 |
| ENSP00000281830 | KCNE4 | 0.2947 |
| ENSP00000347504 | ITGB1BP1 | 0.2947 |
| ENSP00000395219 | IGFL2 | 0.2947 |
| ENSP00000357791 | HRNR | 0.2947 |
| ENSP00000359899 | HCRTR2 | 0.2947 |
| ENSP00000308908 | GPR148 | 0.2947 |
| ENSP00000403067 | FRMD5 | 0.2947 |
| ENSP00000321927 | FBXL13 | 0.2947 |
| ENSP00000349805 | FAM212B | 0.2947 |
| ENSP00000358251 | FAM160B1 | 0.2947 |
| ENSP00000350651 | ERI2 | 0.2947 |
| ENSP00000333823 | EFHC2 | 0.2947 |
| ENSP00000364271 | DFNB59 | 0.2947 |
| ENSP00000335382 | DEFB128 | 0.2947 |
| ENSP00000367248 | CD177 | 0.2947 |
| ENSP00000293276 | CCL15 | 0.2947 |
| ENSP00000362704 | CCDC28B | 0.2947 |
| ENSP00000288065 | ARMC12 | 0.2947 |
| ENSP00000282849 | ADAMTS18 | 0.2947 |
| ENSP00000362304 | ADAMTS14 | 0.2947 |
| ENSP00000200557 | ADAM11 | 0.2947 |
| ENSP00000358241 | ACP6 | 0.2947 |
| ENSP00000332595 | ZNF92 | 0.2921 |
| ENSP00000270451 | ZNF581 | 0.2921 |
| ENSP00000363556 | ZNF32 | 0.2921 |
| ENSP00000366902 | ZBTB48 | 0.2921 |
| ENSP00000388299 | TRIM49C | 0.2921 |
| ENSP00000296529 | TMEM144 | 0.2921 |
| ENSP00000400471 | TMEM106C | 0.2921 |
| ENSP00000468046 | TCEB3CL2 | 0.2921 |
| ENSP00000325564 | SYCN | 0.2921 |
| ENSP00000231461 | ST8SIA4 | 0.2921 |
| ENSP00000299977 | SLFN5 | 0.2921 |
| ENSP00000260126 | SLCO5A1 | 0.2921 |
| ENSP00000217909 | SLC25A43 | 0.2921 |
| ENSP00000294454 | SLC25A34 | 0.2921 |
| ENSP00000352028 | SH3RF2 | 0.2921 |
| ENSP00000284637 | SH3RF1 | 0.2921 |
| ENSP00000340237 | SH3BP4 | 0.2921 |
| ENSP00000330930 | SGK223 | 0.2921 |
| ENSP00000387361 | SBNO1 | 0.2921 |
| ENSP00000349359 | SASH3 | 0.2921 |
| ENSP00000297186 | RSPH10B2 | 0.2921 |
| ENSP00000255324 | RNF17 | 0.2921 |
| ENSP00000345151 | RFPL4AL1 | 0.2921 |
| ENSP00000345826 | PMEPA1 | 0.2921 |
| ENSP00000252590 | PLVAP | 0.2921 |
| ENSP00000380155 | OR51E1 | 0.2921 |
| ENSP00000358951 | OPN1MW | 0.2921 |
| ENSP00000338352 | NUDT4 | 0.2921 |

| | | |
|---|---|---|
| ENSP00000399903 | NBEAL1 | 0.2921 |
| ENSP00000356265 | NAV1 | 0.2921 |
| ENSP00000356115 | MFSD4 | 0.2921 |
| ENSP00000354920 | METTL13 | 0.2921 |
| ENSP00000314484 | MEIOB | 0.2921 |
| ENSP00000367486 | MEIG1 | 0.2921 |
| ENSP00000385727 | MAST4 | 0.2921 |
| ENSP00000286482 | MAGEA8 | 0.2921 |
| ENSP00000246529 | LRFN3 | 0.2921 |
| ENSP00000382584 | KRTAP5-5 | 0.2921 |
| ENSP00000375232 | KRTAP4-11 | 0.2921 |
| ENSP00000375475 | KRTAP12-1 | 0.2921 |
| ENSP00000347532 | KIAA1598 | 0.2921 |
| ENSP00000367459 | KIAA0319 | 0.2921 |
| ENSP00000294725 | KCNT2 | 0.2921 |
| ENSP00000319918 | ITFG1 | 0.2921 |
| ENSP00000328111 | IL25 | 0.2921 |
| ENSP00000417492 | FSD1L | 0.2921 |
| ENSP00000373370 | FLG2 | 0.2921 |
| ENSP00000356925 | FCRLB | 0.2921 |
| ENSP00000323635 | FAM210A | 0.2921 |
| ENSP00000310565 | DSEL | 0.2921 |
| ENSP00000427336 | DIO3 | 0.2921 |
| ENSP00000363624 | CUTA | 0.2921 |
| ENSP00000332716 | CCDC28A | 0.2921 |
| ENSP00000362507 | CCDC167 | 0.2921 |
| ENSP00000398526 | BBS1 | 0.2921 |
| ENSP00000367453 | ACBD7 | 0.2921 |
| ENSP00000292450 | ZSCAN21 | 0.2895 |
| ENSP00000416662 | ZNF713 | 0.2895 |
| ENSP00000354686 | ZNF652 | 0.2895 |
| ENSP00000340749 | ZNF124 | 0.2895 |
| ENSP00000283441 | ZDHHC11 | 0.2895 |
| ENSP00000269346 | TTYH2 | 0.2895 |
| ENSP00000360323 | TTC22 | 0.2895 |
| ENSP00000431987 | TRIM64C | 0.2895 |
| ENSP00000327994 | TRIM50 | 0.2895 |
| ENSP00000409231 | TRAPPC13 | 0.2895 |
| ENSP00000057513 | TNIP3 | 0.2895 |
| ENSP00000341364 | TMEM230 | 0.2895 |
| ENSP00000346954 | TMEM205 | 0.2895 |
| ENSP00000304277 | TM4SF1 | 0.2895 |
| ENSP00000441949 | TAS2R14 | 0.2895 |
| ENSP00000258381 | SP110 | 0.2895 |
| ENSP00000276373 | SLC18A1 | 0.2895 |
| ENSP00000401502 | SIGLEC6 | 0.2895 |
| ENSP00000023939 | RTFDC1 | 0.2895 |
| ENSP00000420465 | RNF182 | 0.2895 |
| ENSP00000307096 | RNASE4 | 0.2895 |
| ENSP00000334851 | RGS7BP | 0.2895 |
| ENSP00000233735 | REG1A | 0.2895 |
| ENSP00000294484 | PTCHD2 | 0.2895 |
| ENSP00000359234 | PDZD7 | 0.2895 |

| | | |
|---|---|---|
| ENSP00000228820 | PARP11 | 0.2895 |
| ENSP00000377778 | MIEN1 | 0.2895 |
| ENSP00000343118 | LAMTOR4 | 0.2895 |
| ENSP00000456743 | KLHL36 | 0.2895 |
| ENSP00000085068 | ISOC2 | 0.2895 |
| ENSP00000288167 | IL17RB | 0.2895 |
| ENSP00000454407 | GOLGA6L2 | 0.2895 |
| ENSP00000294671 | GBP7 | 0.2895 |
| ENSP00000309432 | FAM134C | 0.2895 |
| ENSP00000267113 | ESYT1 | 0.2895 |
| ENSP00000267889 | DISP2 | 0.2895 |
| ENSP00000297056 | DAGLB | 0.2895 |
| ENSP00000312099 | CRYGS | 0.2895 |
| ENSP00000265085 | CPEB4 | 0.2895 |
| ENSP00000288040 | CLEC18A | 0.2895 |
| ENSP00000362601 | CHIC1 | 0.2895 |
| ENSP00000353259 | CD300A | 0.2895 |
| ENSP00000384193 | CADM2 | 0.2895 |
| ENSP00000423049 | C5orf27 | 0.2895 |
| ENSP00000333737 | C1QTNF9 | 0.2895 |
| ENSP00000338812 | C1QTNF6 | 0.2895 |
| ENSP00000324510 | BAIAP3 | 0.2895 |
| ENSP00000323479 | B3GALNT1 | 0.2895 |
| ENSP00000406381 | APOC4 | 0.2895 |
| ENSP00000365063 | ABHD13 | 0.2895 |
| ENSP00000429148 | ZNF674 | 0.2868 |
| ENSP00000282286 | ZNF304 | 0.2868 |
| ENSP00000391067 | ZNF302 | 0.2868 |
| ENSP00000395723 | ZNF200 | 0.2868 |
| ENSP00000269834 | ZIM3 | 0.2868 |
| ENSP00000363972 | ZC4H2 | 0.2868 |
| ENSP00000263243 | ZC3H7B | 0.2868 |
| ENSP00000340839 | ZC3H12B | 0.2868 |
| ENSP00000398211 | TTC21A | 0.2868 |
| ENSP00000262067 | TSPAN13 | 0.2868 |
| ENSP00000302144 | TMEM154 | 0.2868 |
| ENSP00000296736 | TIGD6 | 0.2868 |
| ENSP00000020926 | SYT13 | 0.2868 |
| ENSP00000344219 | SUSD4 | 0.2868 |
| ENSP00000168148 | SPP2 | 0.2868 |
| ENSP00000324870 | SPINK6 | 0.2868 |
| ENSP00000341765 | SPATA16 | 0.2868 |
| ENSP00000358715 | SORCS3 | 0.2868 |
| ENSP00000246515 | SLURP1 | 0.2868 |
| ENSP00000369135 | SLCO6A1 | 0.2868 |
| ENSP00000242275 | SLC25A51 | 0.2868 |
| ENSP00000371729 | SACS | 0.2868 |
| ENSP00000297157 | RP9 | 0.2868 |
| ENSP00000357229 | RHBG | 0.2868 |
| ENSP00000357882 | PSTK | 0.2868 |
| ENSP00000245810 | PSPN | 0.2868 |
| ENSP00000300835 | PRR14 | 0.2868 |
| ENSP00000399679 | PRG4 | 0.2868 |

| | | |
|---|---|---|
| ENSP00000318131 | PNMAL1 | 0.2868 |
| ENSP00000358945 | OPN1MW2 | 0.2868 |
| ENSP00000262283 | OC90 | 0.2868 |
| ENSP00000401177 | NOL8 | 0.2868 |
| ENSP00000253247 | NOL11 | 0.2868 |
| ENSP00000386227 | MCC | 0.2868 |
| ENSP00000376246 | MB21D2 | 0.2868 |
| ENSP00000273390 | MAATS1 | 0.2868 |
| ENSP00000380605 | LYPD1 | 0.2868 |
| ENSP00000361185 | LRIT2 | 0.2868 |
| ENSP00000357810 | LINGO4 | 0.2868 |
| ENSP00000315997 | LILRB1 | 0.2868 |
| ENSP00000274507 | LECT2 | 0.2868 |
| ENSP00000305975 | KRTAP1-1 | 0.2868 |
| ENSP00000260191 | HTR3B | 0.2868 |
| ENSP00000446205 | HES7 | 0.2868 |
| ENSP00000234142 | GREB1 | 0.2868 |
| ENSP00000384345 | GPR1 | 0.2868 |
| ENSP00000380037 | FITM2 | 0.2868 |
| ENSP00000270879 | FCN3 | 0.2868 |
| ENSP00000331044 | ENKUR | 0.2868 |
| ENSP00000354579 | CMTM3 | 0.2868 |
| ENSP00000219400 | CMC2 | 0.2868 |
| ENSP00000367265 | CKAP4 | 0.2868 |
| ENSP00000338783 | CHST5 | 0.2868 |
| ENSP00000221992 | CEACAM5 | 0.2868 |
| ENSP00000385576 | CEACAM16 | 0.2868 |
| ENSP00000357150 | CD1B | 0.2868 |
| ENSP00000295117 | CCDC104 | 0.2868 |
| ENSP00000367471 | BTBD3 | 0.2868 |
| ENSP00000256538 | ATPBD4 | 0.2868 |
| ENSP00000442477 | ASIC5 | 0.2868 |
| ENSP00000435517 | ALG9 | 0.2868 |
| ENSP00000267499 | ADAM21 | 0.2868 |
| ENSP00000268624 | ADAD2 | 0.2868 |
| ENSP00000342002 | ZNF93 | 0.2842 |
| ENSP00000301096 | ZNF83 | 0.2842 |
| ENSP00000341151 | ZNF81 | 0.2842 |
| ENSP00000415836 | ZNF276 | 0.2842 |
| ENSP00000344616 | ZAR1L | 0.2842 |
| ENSP00000318684 | VSIG2 | 0.2842 |
| ENSP00000328998 | TRAK1 | 0.2842 |
| ENSP00000249700 | TMOD2 | 0.2842 |
| ENSP00000354441 | TMEM184B | 0.2842 |
| ENSP00000371364 | TMEM156 | 0.2842 |
| ENSP00000429275 | TMEM125 | 0.2842 |
| ENSP00000409932 | TCEB3CL | 0.2842 |
| ENSP00000355273 | ST6GAL2 | 0.2842 |
| ENSP00000345964 | SORCS1 | 0.2842 |
| ENSP00000024061 | SLC45A4 | 0.2842 |
| ENSP00000316007 | SCAMP4 | 0.2842 |
| ENSP00000434508 | RP11-849H4.2 | 0.2842 |
| ENSP00000302046 | RNASE6 | 0.2842 |

| | | |
|---|---|---|
| ENSP00000397644 | RFX7 | 0.2842 |
| ENSP00000424261 | PTTG2 | 0.2842 |
| ENSP00000387261 | PSTPIP2 | 0.2842 |
| ENSP00000401701 | PRSS42 | 0.2842 |
| ENSP00000345359 | PODXL2 | 0.2842 |
| ENSP00000460274 | PCDHGA9 | 0.2842 |
| ENSP00000454411 | OST4 | 0.2842 |
| ENSP00000457071 | MPV17L | 0.2842 |
| ENSP00000368304 | MAGEB10 | 0.2842 |
| ENSP00000299194 | LRTM2 | 0.2842 |
| ENSP00000399441 | LRRC70 | 0.2842 |
| ENSP00000261247 | JKAMP | 0.2842 |
| ENSP00000295981 | IL17RC | 0.2842 |
| ENSP00000321184 | IGSF3 | 0.2842 |
| ENSP00000365601 | IDNK | 0.2842 |
| ENSP00000004982 | HSPB6 | 0.2842 |
| ENSP00000362094 | GTSF1L | 0.2842 |
| ENSP00000295500 | GPR155 | 0.2842 |
| ENSP00000263384 | FAM32A | 0.2842 |
| ENSP00000385158 | FAM161A | 0.2842 |
| ENSP00000257013 | FAM127A | 0.2842 |
| ENSP00000278903 | EI24 | 0.2842 |
| ENSP00000381921 | DSCR4 | 0.2842 |
| ENSP00000360803 | DNLZ | 0.2842 |
| ENSP00000334681 | DEFB107A | 0.2842 |
| ENSP00000316496 | DCAF4L2 | 0.2842 |
| ENSP00000358763 | CHI3L2 | 0.2842 |
| ENSP00000259053 | CD302 | 0.2842 |
| ENSP00000349967 | CCRL2 | 0.2842 |
| ENSP00000372576 | CCDC178 | 0.2842 |
| ENSP00000354769 | C1orf68 | 0.2842 |
| ENSP00000300618 | C17orf78 | 0.2842 |
| ENSP00000344609 | BTG3 | 0.2842 |
| ENSP00000374359 | ZXDC | 0.2816 |
| ENSP00000308548 | ZNHIT2 | 0.2816 |
| ENSP00000405218 | ZNF347 | 0.2816 |
| ENSP00000328732 | ZBTB7C | 0.2816 |
| ENSP00000264234 | UPK1B | 0.2816 |
| ENSP00000361072 | TSPAN1 | 0.2816 |
| ENSP00000211076 | TPSD1 | 0.2816 |
| ENSP00000312309 | TNK1 | 0.2816 |
| ENSP00000331827 | TNFAIP8L1 | 0.2816 |
| ENSP00000301939 | TMEM256 | 0.2816 |
| ENSP00000281924 | TMEM163 | 0.2816 |
| ENSP00000229563 | TMEM14C | 0.2816 |
| ENSP00000266534 | TMEM117 | 0.2816 |
| ENSP00000308354 | TIGD3 | 0.2816 |
| ENSP00000215742 | THAP7 | 0.2816 |
| ENSP00000240619 | TAS2R10 | 0.2816 |
| ENSP00000356908 | TAAR2 | 0.2816 |
| ENSP00000304707 | SLN | 0.2816 |
| ENSP00000355109 | SFMBT2 | 0.2816 |
| ENSP00000364965 | SECISBP2 | 0.2816 |

| | | |
|---|---|---|
| ENSP00000244360 | RNF39 | 0.2816 |
| ENSP00000388207 | RNF148 | 0.2816 |
| ENSP00000390630 | RIMBP3C | 0.2816 |
| ENSP00000255476 | RFXAP | 0.2816 |
| ENSP00000336866 | RBBP9 | 0.2816 |
| ENSP00000430307 | PRSS46 | 0.2816 |
| ENSP00000262731 | PRR5-ARHGAP8 | 0.2816 |
| ENSP00000055335 | PPP1R3F | 0.2816 |
| ENSP00000342143 | PDZK1 | 0.2816 |
| ENSP00000347742 | PDZD3 | 0.2816 |
| ENSP00000202017 | PDRG1 | 0.2816 |
| ENSP00000334456 | OR5D14 | 0.2816 |
| ENSP00000408697 | NUGGC | 0.2816 |
| ENSP00000405674 | NOL7 | 0.2816 |
| ENSP00000370949 | NFXL1 | 0.2816 |
| ENSP00000341637 | NCR3LG1 | 0.2816 |
| ENSP00000281513 | NBAS | 0.2816 |
| ENSP00000362127 | MTF1 | 0.2816 |
| ENSP00000396622 | MROH7 | 0.2816 |
| ENSP00000278937 | MPZL2 | 0.2816 |
| ENSP00000216075 | MIOX | 0.2816 |
| ENSP00000381566 | MFSD12 | 0.2816 |
| ENSP00000294635 | LRRC53 | 0.2816 |
| ENSP00000325091 | LRCH2 | 0.2816 |
| ENSP00000333952 | LCE5A | 0.2816 |
| ENSP00000335358 | LCE3B | 0.2816 |
| ENSP00000288350 | LCA5L | 0.2816 |
| ENSP00000375476 | KRTAP12-4 | 0.2816 |
| ENSP00000300976 | KLHL26 | 0.2816 |
| ENSP00000336769 | KIR2DL1 | 0.2816 |
| ENSP00000374558 | KIAA0226L | 0.2816 |
| ENSP00000456039 | INMT-FAM188B | 0.2816 |
| ENSP00000376403 | GPRC5C | 0.2816 |
| ENSP00000270310 | FXYD7 | 0.2816 |
| ENSP00000334665 | FSCN2 | 0.2816 |
| ENSP00000360538 | FFAR4 | 0.2816 |
| ENSP00000369863 | FAM57B | 0.2816 |
| ENSP00000291634 | FAM207A | 0.2816 |
| ENSP00000452347 | CTB-96E2.2 | 0.2816 |
| ENSP00000419378 | CLIC3 | 0.2816 |
| ENSP00000381290 | CHTF8 | 0.2816 |
| ENSP00000384887 | CEACAM19 | 0.2816 |
| ENSP00000287097 | CD109 | 0.2816 |
| ENSP00000366656 | BX255923.1 | 0.2816 |
| ENSP00000355559 | B3GALNT2 | 0.2816 |
| ENSP00000309749 | APOBEC3F | 0.2816 |
| ENSP00000290943 | ANKRD18B | 0.2816 |
| ENSP00000415273 | AL353608.1 | 0.2816 |
| ENSP00000025301 | AKAP11 | 0.2816 |
| ENSP00000360973 | AGTR2 | 0.2816 |
| ENSP00000334800 | ZSCAN25 | 0.2789 |
| ENSP00000362446 | ZNF79 | 0.2789 |
| ENSP00000304769 | ZNF467 | 0.2789 |

| | | |
|---|---|---|
| ENSP00000337363 | ZNF394 | 0.2789 |
| ENSP00000435550 | ZC2HC1C | 0.2789 |
| ENSP00000343990 | TRIM14 | 0.2789 |
| ENSP00000323455 | TPRX1 | 0.2789 |
| ENSP00000381476 | TMPRSS12 | 0.2789 |
| ENSP00000341657 | THYN1 | 0.2789 |
| ENSP00000387593 | TANC2 | 0.2789 |
| ENSP00000273183 | STAC | 0.2789 |
| ENSP00000336733 | ST6GALNAC4 | 0.2789 |
| ENSP00000329214 | ST6GALNAC3 | 0.2789 |
| ENSP00000369045 | SPINK4 | 0.2789 |
| ENSP00000354090 | SIGLEC14 | 0.2789 |
| ENSP00000412361 | SIGLEC11 | 0.2789 |
| ENSP00000422644 | SEPW1 | 0.2789 |
| ENSP00000260385 | RMDN3 | 0.2789 |
| ENSP00000260045 | PRKRIR | 0.2789 |
| ENSP00000412436 | PRH1 | 0.2789 |
| ENSP00000345175 | PODNL1 | 0.2789 |
| ENSP00000428603 | PCDHGB6 | 0.2789 |
| ENSP00000431083 | PCDHGA1 | 0.2789 |
| ENSP00000367151 | PCDH17 | 0.2789 |
| ENSP00000289619 | PAGE5 | 0.2789 |
| ENSP00000316955 | OR7A5 | 0.2789 |
| ENSP00000319546 | OR2A4 | 0.2789 |
| ENSP00000308082 | OR10P1 | 0.2789 |
| ENSP00000399078 | OR10J1 | 0.2789 |
| ENSP00000264312 | OCIAD1 | 0.2789 |
| ENSP00000277746 | NRBF2 | 0.2789 |
| ENSP00000290705 | MT1A | 0.2789 |
| ENSP00000332646 | MFSD10 | 0.2789 |
| ENSP00000362638 | MARCKSL1 | 0.2789 |
| ENSP00000329199 | MAGEA6 | 0.2789 |
| ENSP00000359483 | LRRC8C | 0.2789 |
| ENSP00000469337 | LRRC63 | 0.2789 |
| ENSP00000358778 | LRIF1 | 0.2789 |
| ENSP00000358966 | IMPG1 | 0.2789 |
| ENSP00000357073 | IGSF9 | 0.2789 |
| ENSP00000276708 | GSDMC | 0.2789 |
| ENSP00000300571 | GPRC5B | 0.2789 |
| ENSP00000358233 | GPR89B | 0.2789 |
| ENSP00000356251 | GPR37L1 | 0.2789 |
| ENSP00000335156 | GPR144 | 0.2789 |
| ENSP00000266754 | GAS2L3 | 0.2789 |
| ENSP00000359353 | GABRE | 0.2789 |
| ENSP00000285046 | FGD5 | 0.2789 |
| ENSP00000377396 | FAM198B | 0.2789 |
| ENSP00000359052 | FAM102B | 0.2789 |
| ENSP00000357409 | DCST2 | 0.2789 |
| ENSP00000446100 | CMIP | 0.2789 |
| ENSP00000234701 | CLCA1 | 0.2789 |
| ENSP00000361721 | CITED4 | 0.2789 |
| ENSP00000309270 | CHST1 | 0.2789 |
| ENSP00000310966 | CD3EAP | 0.2789 |

| | | |
|---|---|---|
| ENSP00000374507 | CCDC88C | 0.2789 |
| ENSP00000350961 | C9orf123 | 0.2789 |
| ENSP00000297534 | C7orf55 | 0.2789 |
| ENSP00000328677 | C2CD4C | 0.2789 |
| ENSP00000354553 | C1orf85 | 0.2789 |
| ENSP00000333845 | C11orf88 | 0.2789 |
| ENSP00000042931 | BEST2 | 0.2789 |
| ENSP00000358878 | AMIGO1 | 0.2789 |
| ENSP00000245812 | ALKBH7 | 0.2789 |
| ENSP00000405969 | ACBD4 | 0.2789 |
| ENSP00000410157 | ABTB2 | 0.2789 |
| ENSP00000395007 | ZNF746 | 0.2763 |
| ENSP00000428878 | ZNF721 | 0.2763 |
| ENSP00000377873 | ZNF358 | 0.2763 |
| ENSP00000461413 | ZNF286B | 0.2763 |
| ENSP00000352920 | ZMYM1 | 0.2763 |
| ENSP00000348593 | ZFYVE27 | 0.2763 |
| ENSP00000359891 | ZDHHC16 | 0.2763 |
| ENSP00000307604 | ZBTB5 | 0.2763 |
| ENSP00000398106 | ZBED5 | 0.2763 |
| ENSP00000258243 | URB2 | 0.2763 |
| ENSP00000398003 | U82695.9 | 0.2763 |
| ENSP00000323584 | TSHZ1 | 0.2763 |
| ENSP00000422581 | TRIML2 | 0.2763 |
| ENSP00000330216 | TRIM49B | 0.2763 |
| ENSP00000349087 | TMEM259 | 0.2763 |
| ENSP00000299308 | TMEM132B | 0.2763 |
| ENSP00000358957 | TEX28P2 | 0.2763 |
| ENSP00000324404 | TCHP | 0.2763 |
| ENSP00000377520 | SYT12 | 0.2763 |
| ENSP00000352097 | SPTSSB | 0.2763 |
| ENSP00000433712 | SPRN | 0.2763 |
| ENSP00000296088 | SNRK | 0.2763 |
| ENSP00000366288 | SLITRK1 | 0.2763 |
| ENSP00000366299 | SLC25A39 | 0.2763 |
| ENSP00000295190 | SLC16A14 | 0.2763 |
| ENSP00000369374 | SKOR1 | 0.2763 |
| ENSP00000261674 | SFSWAP | 0.2763 |
| ENSP00000357706 | S100A5 | 0.2763 |
| ENSP00000314946 | RPRM | 0.2763 |
| ENSP00000364476 | RIBC1 | 0.2763 |
| ENSP00000161006 | PRSS22 | 0.2763 |
| ENSP00000239032 | PRLHR | 0.2763 |
| ENSP00000362942 | PHACTR4 | 0.2763 |
| ENSP00000378077 | PCDHGA2 | 0.2763 |
| ENSP00000431914 | OR10S1 | 0.2763 |
| ENSP00000305631 | NXNL1 | 0.2763 |
| ENSP00000337443 | NXN | 0.2763 |
| ENSP00000318154 | MRFAP1L1 | 0.2763 |
| ENSP00000301920 | MMADHC | 0.2763 |
| ENSP00000263369 | MIA | 0.2763 |
| ENSP00000320234 | MFSD7 | 0.2763 |
| ENSP00000298974 | MAGEA9 | 0.2763 |

| | | |
|---|---|---|
| ENSP00000430025 | LYRM2 | 0.2763 |
| ENSP00000450675 | LIMS3 | 0.2763 |
| ENSP00000457330 | LIMS1 | 0.2763 |
| ENSP00000357412 | LENEP | 0.2763 |
| ENSP00000233714 | LANCL1 | 0.2763 |
| ENSP00000346536 | KRTAP23-1 | 0.2763 |
| ENSP00000307014 | KRT73 | 0.2763 |
| ENSP00000352314 | KLHL14 | 0.2763 |
| ENSP00000356189 | IPCEF1 | 0.2763 |
| ENSP00000239316 | INSL4 | 0.2763 |
| ENSP00000385491 | GTF2IRD2 | 0.2763 |
| ENSP00000275169 | GPR6 | 0.2763 |
| ENSP00000348161 | GPR160 | 0.2763 |
| ENSP00000316861 | GPM6B | 0.2763 |
| ENSP00000371170 | GAGE13 | 0.2763 |
| ENSP00000364512 | G6PC2 | 0.2763 |
| ENSP00000275461 | FERD3L | 0.2763 |
| ENSP00000369618 | FASTKD5 | 0.2763 |
| ENSP00000286544 | FAM161B | 0.2763 |
| ENSP00000398401 | FAM133B | 0.2763 |
| ENSP00000216214 | FAM118A | 0.2763 |
| ENSP00000371643 | DEFB135 | 0.2763 |
| ENSP00000412267 | CXXC4 | 0.2763 |
| ENSP00000264376 | CRYGD | 0.2763 |
| ENSP00000268720 | CPNE7 | 0.2763 |
| ENSP00000267935 | COMMD4 | 0.2763 |
| ENSP00000296658 | CMBL | 0.2763 |
| ENSP00000229332 | CLEC4A | 0.2763 |
| ENSP00000356395 | CFHR3 | 0.2763 |
| ENSP00000329942 | CD300E | 0.2763 |
| ENSP00000309681 | CCDC106 | 0.2763 |
| ENSP00000237822 | C2orf43 | 0.2763 |
| ENSP00000333064 | C22orf39 | 0.2763 |
| ENSP00000256324 | C14orf159 | 0.2763 |
| ENSP00000332413 | BEST3 | 0.2763 |
| ENSP00000387512 | AC139426.2 | 0.2763 |
| ENSP00000387547 | AC016745.1 | 0.2763 |
| ENSP00000408206 | ENSG00000235153 | 0.2763 |
| ENSP00000440053 | NPIPP1 | 0.2763 |
| ENSP00000293771 | ZNF653 | 0.2737 |
| ENSP00000344308 | ZNF341 | 0.2737 |
| ENSP00000354501 | ZNF277 | 0.2737 |
| ENSP00000308921 | ZCCHC12 | 0.2737 |
| ENSP00000409107 | ZBTB42 | 0.2737 |
| ENSP00000267522 | WDR89 | 0.2737 |
| ENSP00000256339 | UNC79 | 0.2737 |
| ENSP00000382713 | UBE2QL1 | 0.2737 |
| ENSP00000383911 | UBAC2 | 0.2737 |
| ENSP00000347161 | TSGA10 | 0.2737 |
| ENSP00000278422 | TMX2 | 0.2737 |
| ENSP00000355800 | TMEM63A | 0.2737 |
| ENSP00000227525 | TMEM109 | 0.2737 |
| ENSP00000264866 | TBC1D19 | 0.2737 |

| | | |
|---|---|---|
| ENSP00000352849 | SIRPB2 | 0.2737 |
| ENSP00000413861 | SIGLEC9 | 0.2737 |
| ENSP00000261593 | RNF138 | 0.2737 |
| ENSP00000295049 | RFTN2 | 0.2737 |
| ENSP00000242249 | RAMP3 | 0.2737 |
| ENSP00000288368 | PREX2 | 0.2737 |
| ENSP00000393832 | PPP1R3G | 0.2737 |
| ENSP00000456953 | POTEB | 0.2737 |
| ENSP00000240617 | PLBD1 | 0.2737 |
| ENSP00000458570 | PCDHGA4 | 0.2737 |
| ENSP00000261888 | PARP16 | 0.2737 |
| ENSP00000239690 | NUDCD1 | 0.2737 |
| ENSP00000364782 | NEU1 | 0.2737 |
| ENSP00000315397 | MRPS31 | 0.2737 |
| ENSP00000368315 | MAGEB5 | 0.2737 |
| ENSP00000316891 | LIMCH1 | 0.2737 |
| ENSP00000335006 | LCE3A | 0.2737 |
| ENSP00000267119 | KRT71 | 0.2737 |
| ENSP00000358955 | KIAA1324 | 0.2737 |
| ENSP00000407353 | IFLTD1 | 0.2737 |
| ENSP00000335447 | HEATR4 | 0.2737 |
| ENSP00000271357 | GPR161 | 0.2737 |
| ENSP00000329266 | GPIHBP1 | 0.2737 |
| ENSP00000356842 | GPA33 | 0.2737 |
| ENSP00000266014 | GLT8D1 | 0.2737 |
| ENSP00000355938 | FLVCR1 | 0.2737 |
| ENSP00000368517 | ECHDC3 | 0.2737 |
| ENSP00000297581 | DCSTAMP | 0.2737 |
| ENSP00000375220 | CTAGE1 | 0.2737 |
| ENSP00000309524 | CSHL1 | 0.2737 |
| ENSP00000465204 | CNTD1 | 0.2737 |
| ENSP00000268595 | CMTM2 | 0.2737 |
| ENSP00000460682 | CLEC3A | 0.2737 |
| ENSP00000391402 | CISD3 | 0.2737 |
| ENSP00000328983 | CHST6 | 0.2737 |
| ENSP00000262150 | CDH19 | 0.2737 |
| ENSP00000376397 | CD300LB | 0.2737 |
| ENSP00000368102 | CCDC3 | 0.2737 |
| ENSP00000431759 | C12ORF55 | 0.2737 |
| ENSP00000329990 | BX072566.1 | 0.2737 |
| ENSP00000341762 | BSPH1 | 0.2737 |
| ENSP00000370047 | BNC2 | 0.2737 |
| ENSP00000361813 | BEX1 | 0.2737 |
| ENSP00000349891 | ANKRD20A4 | 0.2737 |
| ENSP00000335255 | ANGPTL5 | 0.2737 |
| ENSP00000236709 | A4GNT | 0.2737 |
| ENSP00000306756 | ZNF584 | 0.2711 |
| ENSP00000322545 | ZNF543 | 0.2711 |
| ENSP00000298299 | ZNF22 | 0.2711 |
| ENSP00000278314 | ZNF214 | 0.2711 |
| ENSP00000385939 | ZNF12 | 0.2711 |
| ENSP00000331704 | XKR3 | 0.2711 |
| ENSP00000361726 | WFDC10A | 0.2711 |

| | | |
|---|---|---|
| ENSP00000386609 | WDR81 | 0.2711 |
| ENSP00000368612 | VWA8 | 0.2711 |
| ENSP00000350409 | UNC50 | 0.2711 |
| ENSP00000332969 | TRIM64B | 0.2711 |
| ENSP00000257604 | TRAFD1 | 0.2711 |
| ENSP00000330475 | TMPRSS11B | 0.2711 |
| ENSP00000334849 | TMEM104 | 0.2711 |
| ENSP00000395328 | TMEM100 | 0.2711 |
| ENSP00000330433 | TM2D3 | 0.2711 |
| ENSP00000361522 | TJAP1 | 0.2711 |
| ENSP00000258613 | THSD1 | 0.2711 |
| ENSP00000358142 | SV2A | 0.2711 |
| ENSP00000265007 | SOX30 | 0.2711 |
| ENSP00000339324 | SMIM15 | 0.2711 |
| ENSP00000376123 | SH2D2A | 0.2711 |
| ENSP00000312439 | RNF26 | 0.2711 |
| ENSP00000296498 | PRSS12 | 0.2711 |
| ENSP00000378935 | PPHLN1 | 0.2711 |
| ENSP00000351695 | PNLIPRP1 | 0.2711 |
| ENSP00000351379 | PFN3 | 0.2711 |
| ENSP00000429834 | PCDHGA5 | 0.2711 |
| ENSP00000287008 | PCDH1 | 0.2711 |
| ENSP00000405424 | OXR1 | 0.2711 |
| ENSP00000373610 | OTOA | 0.2711 |
| ENSP00000301532 | OR5I1 | 0.2711 |
| ENSP00000332613 | OAF | 0.2711 |
| ENSP00000364595 | NINJ1 | 0.2711 |
| ENSP00000473092 | MUC8 | 0.2711 |
| ENSP00000264819 | MIER2 | 0.2711 |
| ENSP00000382867 | MAGEB16 | 0.2711 |
| ENSP00000296252 | LIPH | 0.2711 |
| ENSP00000250366 | KLK9 | 0.2711 |
| ENSP00000355211 | KIAA1704 | 0.2711 |
| ENSP00000347188 | KCTD6 | 0.2711 |
| ENSP00000287996 | IPPK | 0.2711 |
| ENSP00000263326 | IL37 | 0.2711 |
| ENSP00000386207 | HMSD | 0.2711 |
| ENSP00000310255 | GPR152 | 0.2711 |
| ENSP00000458791 | GPR139 | 0.2711 |
| ENSP00000359634 | GOLGA7B | 0.2711 |
| ENSP00000300515 | GOLGA6L9 | 0.2711 |
| ENSP00000290438 | GOLGA6A | 0.2711 |
| ENSP00000315263 | GLTP | 0.2711 |
| ENSP00000240986 | GJA8 | 0.2711 |
| ENSP00000423313 | GALNTL6 | 0.2711 |
| ENSP00000394249 | FLCN | 0.2711 |
| ENSP00000221665 | FIZ1 | 0.2711 |
| ENSP00000416558 | FBXO24 | 0.2711 |
| ENSP00000238823 | FAM98A | 0.2711 |
| ENSP00000393987 | FAM19A2 | 0.2711 |
| ENSP00000355045 | FAM179B | 0.2711 |
| ENSP00000354270 | FAM107A | 0.2711 |
| ENSP00000352273 | DIO3 | 0.2711 |

| | | |
|---|---|---|
| ENSP00000335307 | DEFB106A | 0.2711 |
| ENSP00000334330 | DEFB105A | 0.2711 |
| ENSP00000390590 | DDN | 0.2711 |
| ENSP00000265922 | DBC1 | 0.2711 |
| ENSP00000371294 | CTNS | 0.2711 |
| ENSP00000328521 | CRIP2 | 0.2711 |
| ENSP00000419475 | COMMD2 | 0.2711 |
| ENSP00000364934 | CLIC1 | 0.2711 |
| ENSP00000449999 | CLEC5A | 0.2711 |
| ENSP00000367476 | CEP104 | 0.2711 |
| ENSP00000289429 | CD1A | 0.2711 |
| ENSP00000365477 | CCDC93 | 0.2711 |
| ENSP00000254691 | CARD6 | 0.2711 |
| ENSP00000369755 | C2CD4B | 0.2711 |
| ENSP00000290155 | C21orf59 | 0.2711 |
| ENSP00000397394 | C19orf25 | 0.2711 |
| ENSP00000307264 | C11orf24 | 0.2711 |
| ENSP00000362637 | BLCAP | 0.2711 |
| ENSP00000246174 | ARMCX5 | 0.2711 |
| ENSP00000253401 | ARHGEF9 | 0.2711 |
| ENSP00000427130 | ANKRD13D | 0.2711 |
| ENSP00000370088 | AMELX | 0.2711 |
| ENSP00000351384 | ANKRD36BP1 | 0.2711 |
| ENSP00000367228 | CMAHP | 0.2711 |
| ENSP00000269973 | ZNF45 | 0.2684 |
| ENSP00000230122 | ZBTB24 | 0.2684 |
| ENSP00000410083 | VEZT | 0.2684 |
| ENSP00000373413 | UFSP1 | 0.2684 |
| ENSP00000360821 | UBAC1 | 0.2684 |
| ENSP00000346791 | TTC3 | 0.2684 |
| ENSP00000372394 | TMEM129 | 0.2684 |
| ENSP00000306887 | TMEM126A | 0.2684 |
| ENSP00000385276 | TENM3 | 0.2684 |
| ENSP00000364621 | SUSD3 | 0.2684 |
| ENSP00000295641 | STK11IP | 0.2684 |
| ENSP00000312402 | SLFN11 | 0.2684 |
| ENSP00000289575 | SLCO2B1 | 0.2684 |
| ENSP00000371872 | SLC35G5 | 0.2684 |
| ENSP00000385028 | SLC22A23 | 0.2684 |
| ENSP00000310241 | SLC22A13 | 0.2684 |
| ENSP00000255226 | SLC14A2 | 0.2684 |
| ENSP00000289707 | SLAMF8 | 0.2684 |
| ENSP00000410862 | RUNDC3A | 0.2684 |
| ENSP00000344193 | RNASE1 | 0.2684 |
| ENSP00000308948 | PTCD2 | 0.2684 |
| ENSP00000371272 | PLIN5 | 0.2684 |
| ENSP00000333751 | PLCXD3 | 0.2684 |
| ENSP00000327763 | NLRP10 | 0.2684 |
| ENSP00000369473 | N4BP2L1 | 0.2684 |
| ENSP00000254770 | LANCL2 | 0.2684 |
| ENSP00000366970 | GPR20 | 0.2684 |
| ENSP00000264080 | GPR108 | 0.2684 |
| ENSP00000467556 | GPATCH8 | 0.2684 |

| | | |
|---|---|---|
| ENSP00000420405 | FSBP | 0.2684 |
| ENSP00000282226 | FAM151B | 0.2684 |
| ENSP00000331915 | FAM101B | 0.2684 |
| ENSP00000321029 | EXD1 | 0.2684 |
| ENSP00000264956 | EVC | 0.2684 |
| ENSP00000337675 | EBAG9 | 0.2684 |
| ENSP00000340017 | DOC2A | 0.2684 |
| ENSP00000216241 | CHADL | 0.2684 |
| ENSP00000327075 | CD300LF | 0.2684 |
| ENSP00000225726 | CCDC47 | 0.2684 |
| ENSP00000355623 | C1orf198 | 0.2684 |
| ENSP00000254336 | C19orf57 | 0.2684 |
| ENSP00000317579 | C16orf58 | 0.2684 |
| ENSP00000358293 | C10orf118 | 0.2684 |
| ENSP00000327459 | APOBEC3B | 0.2684 |
| ENSP00000327608 | ADAMTSL5 | 0.2684 |
| ENSP00000274181 | ADAMTS16 | 0.2684 |
| ENSP00000470123 | ZSCAN18 | 0.2658 |
| ENSP00000305373 | ZNF440 | 0.2658 |
| ENSP00000270708 | WRAP73 | 0.2658 |
| ENSP00000360065 | WDR78 | 0.2658 |
| ENSP00000464258 | VHLL | 0.2658 |
| ENSP00000355299 | TSEN15 | 0.2658 |
| ENSP00000361982 | TRUB2 | 0.2658 |
| ENSP00000297459 | TMEM74 | 0.2658 |
| ENSP00000362021 | TMEM35 | 0.2658 |
| ENSP00000296978 | TMEM200A | 0.2658 |
| ENSP00000301204 | TMEM145 | 0.2658 |
| ENSP00000351380 | TMA16 | 0.2658 |
| ENSP00000248701 | SPINK2 | 0.2658 |
| ENSP00000305343 | SOX14 | 0.2658 |
| ENSP00000278222 | SAA4 | 0.2658 |
| ENSP00000374552 | RNF216 | 0.2658 |
| ENSP00000301258 | PSCA | 0.2658 |
| ENSP00000215912 | PIK3IP1 | 0.2658 |
| ENSP00000375907 | PID1 | 0.2658 |
| ENSP00000008938 | PGLYRP1 | 0.2658 |
| ENSP00000410858 | NHLRC4 | 0.2658 |
| ENSP00000377823 | NDRG4 | 0.2658 |
| ENSP00000262244 | MOB3B | 0.2658 |
| ENSP00000263092 | METTL16 | 0.2658 |
| ENSP00000306760 | LRRC45 | 0.2658 |
| ENSP00000321546 | LOH12CR1 | 0.2658 |
| ENSP00000317141 | KCTD12 | 0.2658 |
| ENSP00000328352 | KCTD11 | 0.2658 |
| ENSP00000356549 | IER5 | 0.2658 |
| ENSP00000367378 | GPR34 | 0.2658 |
| ENSP00000300079 | GLYATL1 | 0.2658 |
| ENSP00000338523 | FNDC3B | 0.2658 |
| ENSP00000339067 | FGFBP3 | 0.2658 |
| ENSP00000339250 | DPPA3 | 0.2658 |
| ENSP00000359170 | DPCD | 0.2658 |
| ENSP00000469533 | CTC-490E21.12 | 0.2658 |

| | | |
|---|---|---|
| ENSP00000360360 | CT47B1 | 0.2658 |
| ENSP00000307540 | CST2 | 0.2658 |
| ENSP00000347919 | COA4 | 0.2658 |
| ENSP00000368814 | CMTM1 | 0.2658 |
| ENSP00000347916 | CLEC12A | 0.2658 |
| ENSP00000357474 | CEP85L | 0.2658 |
| ENSP00000389427 | CEP44 | 0.2658 |
| ENSP00000249064 | CCDC117 | 0.2658 |
| ENSP00000345659 | CA7 | 0.2658 |
| ENSP00000257267 | C5AR2 | 0.2658 |
| ENSP00000280758 | BTBD11 | 0.2658 |
| ENSP00000344285 | BSX | 0.2658 |
| ENSP00000358062 | AIM1 | 0.2658 |
| ENSP00000221204 | FGL1 | 0.2658 |
| ENSP00000319222 | ZNF720 | 0.2632 |
| ENSP00000334853 | ZNF555 | 0.2632 |
| ENSP00000361761 | WFDC2 | 0.2632 |
| ENSP00000263461 | WDR11 | 0.2632 |
| ENSP00000359668 | VGLL1 | 0.2632 |
| ENSP00000358888 | SYPL2 | 0.2632 |
| ENSP00000260637 | SULT6B1 | 0.2632 |
| ENSP00000275764 | STRA8 | 0.2632 |
| ENSP00000366144 | SOX21 | 0.2632 |
| ENSP00000352927 | SOGA2 | 0.2632 |
| ENSP00000436897 | SMIM3 | 0.2632 |
| ENSP00000385814 | RPAIN | 0.2632 |
| ENSP00000468267 | RP11-146D12.2 | 0.2632 |
| ENSP00000326095 | RNF166 | 0.2632 |
| ENSP00000407925 | RIMBP3B | 0.2632 |
| ENSP00000333003 | PRSS55 | 0.2632 |
| ENSP00000314396 | PRMT10 | 0.2632 |
| ENSP00000351856 | POTEC | 0.2632 |
| ENSP00000219207 | PLLP | 0.2632 |
| ENSP00000440896 | PLD5 | 0.2632 |
| ENSP00000433378 | PCDHA6 | 0.2632 |
| ENSP00000363400 | PAFAH2 | 0.2632 |
| ENSP00000325381 | OR8D4 | 0.2632 |
| ENSP00000310488 | OR7D4 | 0.2632 |
| ENSP00000441354 | OR4D2 | 0.2632 |
| ENSP00000306651 | OR4C11 | 0.2632 |
| ENSP00000389625 | OR2M3 | 0.2632 |
| ENSP00000319817 | OPA3 | 0.2632 |
| ENSP00000215061 | OCEL1 | 0.2632 |
| ENSP00000414055 | NRBP2 | 0.2632 |
| ENSP00000359128 | NPM3 | 0.2632 |
| ENSP00000322956 | MFAP3 | 0.2632 |
| ENSP00000377796 | METTL7B | 0.2632 |
| ENSP00000362457 | MAGEE2 | 0.2632 |
| ENSP00000336661 | LRRC71 | 0.2632 |
| ENSP00000357769 | LCE2B | 0.2632 |
| ENSP00000334869 | LCE1A | 0.2632 |
| ENSP00000303549 | GPR82 | 0.2632 |
| ENSP00000359686 | GPR112 | 0.2632 |

| | | |
|---|---|---|
| ENSP00000361483 | GPR107 | 0.2632 |
| ENSP00000264428 | GLRB | 0.2632 |
| ENSP00000355996 | G0S2 | 0.2632 |
| ENSP00000332886 | FREM3 | 0.2632 |
| ENSP00000277632 | FAM188A | 0.2632 |
| ENSP00000359292 | FAM178A | 0.2632 |
| ENSP00000246100 | FAM110A | 0.2632 |
| ENSP00000284274 | FAM105B | 0.2632 |
| ENSP00000334037 | EMC10 | 0.2632 |
| ENSP00000384081 | EFR3B | 0.2632 |
| ENSP00000242827 | EBPL | 0.2632 |
| ENSP00000335306 | DPPA4 | 0.2632 |
| ENSP00000365486 | DEFB123 | 0.2632 |
| ENSP00000472710 | CTD-2192J16.24 | 0.2632 |
| ENSP00000366032 | COMMD3 | 0.2632 |
| ENSP00000339168 | COLEC11 | 0.2632 |
| ENSP00000264001 | CKLF | 0.2632 |
| ENSP00000341206 | CHST4 | 0.2632 |
| ENSP00000264249 | CHST10 | 0.2632 |
| ENSP00000291458 | CCDC58 | 0.2632 |
| ENSP00000261058 | CCDC54 | 0.2632 |
| ENSP00000373566 | CCDC158 | 0.2632 |
| ENSP00000387252 | CCDC103 | 0.2632 |
| ENSP00000356263 | C6orf211 | 0.2632 |
| ENSP00000238044 | C2orf40 | 0.2632 |
| ENSP00000343742 | BROX | 0.2632 |
| ENSP00000354675 | BHLHB9 | 0.2632 |
| ENSP00000266736 | AMDHD1 | 0.2632 |
| ENSP00000339161 | AIDA | 0.2632 |
| ENSP00000402343 | ZNF469 | 0.2605 |
| ENSP00000350869 | ZNF346 | 0.2605 |
| ENSP00000327604 | TRIM49 | 0.2605 |
| ENSP00000362381 | TOR2A | 0.2605 |
| ENSP00000324775 | TMIE | 0.2605 |
| ENSP00000312679 | TESPA1 | 0.2605 |
| ENSP00000343223 | TCL1B | 0.2605 |
| ENSP00000455908 | SOGA3 | 0.2605 |
| ENSP00000236877 | SLC8A2 | 0.2605 |
| ENSP00000357915 | SLC22A16 | 0.2605 |
| ENSP00000390637 | SLC14A1 | 0.2605 |
| ENSP00000245908 | SH2D3A | 0.2605 |
| ENSP00000342313 | SAMD11 | 0.2605 |
| ENSP00000454437 | RP4-576H24.4 | 0.2605 |
| ENSP00000417176 | RNF183 | 0.2605 |
| ENSP00000397415 | RLN3 | 0.2605 |
| ENSP00000198536 | PILRA | 0.2605 |
| ENSP00000268896 | PCTP | 0.2605 |
| ENSP00000381611 | PCDHGA10 | 0.2605 |
| ENSP00000431748 | PCDHA2 | 0.2605 |
| ENSP00000304235 | OR1L6 | 0.2605 |
| ENSP00000363522 | OR13A1 | 0.2605 |
| ENSP00000321929 | NME9 | 0.2605 |
| ENSP00000363899 | NIPSNAP3A | 0.2605 |

| | | |
|---|---|---|
| ENSP00000334197 | KRTAP10-11 | 0.2605 |
| ENSP00000377616 | KRT222 | 0.2605 |
| ENSP00000350570 | IFT20 | 0.2605 |
| ENSP00000398971 | IFRD2 | 0.2605 |
| ENSP00000382670 | GRXCR1 | 0.2605 |
| ENSP00000228887 | GPRC5D | 0.2605 |
| ENSP00000229955 | GPR63 | 0.2605 |
| ENSP00000303149 | GPR27 | 0.2605 |
| ENSP00000362550 | FTSJD2 | 0.2605 |
| ENSP00000238256 | FKBP15 | 0.2605 |
| ENSP00000252530 | FAM98C | 0.2605 |
| ENSP00000354688 | FAM45A | 0.2605 |
| ENSP00000309786 | FAM21D | 0.2605 |
| ENSP00000375267 | FAM127B | 0.2605 |
| ENSP00000268793 | DPEP3 | 0.2605 |
| ENSP00000306407 | DBNDD1 | 0.2605 |
| ENSP00000232508 | CYB561D2 | 0.2605 |
| ENSP00000302543 | CXXC5 | 0.2605 |
| ENSP00000355470 | CNST | 0.2605 |
| ENSP00000348757 | CCDC151 | 0.2605 |
| ENSP00000328495 | C21orf128 | 0.2605 |
| ENSP00000454565 | C17orf103 | 0.2605 |
| ENSP00000318852 | BPNT1 | 0.2605 |
| ENSP00000376148 | ARMC6 | 0.2605 |
| ENSP00000298032 | ARMC3 | 0.2605 |
| ENSP00000399547 | ANKRD63 | 0.2605 |
| ENSP00000459356 | ENSG00000262314 | 0.2605 |
| ENSP00000347338 | ZNF607 | 0.2579 |
| ENSP00000377461 | ZNF559 | 0.2579 |
| ENSP00000354663 | ZNF438 | 0.2579 |
| ENSP00000299687 | ZNF407 | 0.2579 |
| ENSP00000354206 | ZNF219 | 0.2579 |
| ENSP00000335437 | ZNF20 | 0.2579 |
| ENSP00000431265 | ZFP82 | 0.2579 |
| ENSP00000361740 | WBP5 | 0.2579 |
| ENSP00000387100 | TPRN | 0.2579 |
| ENSP00000303992 | TMEM43 | 0.2579 |
| ENSP00000297477 | TMEM184A | 0.2579 |
| ENSP00000206380 | TMEM101 | 0.2579 |
| ENSP00000276480 | ST18 | 0.2579 |
| ENSP00000357251 | SOGA3 | 0.2579 |
| ENSP00000309741 | SLCO4C1 | 0.2579 |
| ENSP00000420939 | SEPP1 | 0.2579 |
| ENSP00000220478 | SCG3 | 0.2579 |
| ENSP00000453436 | RP11-467N20.5 | 0.2579 |
| ENSP00000296292 | RFT1 | 0.2579 |
| ENSP00000358919 | PSRC1 | 0.2579 |
| ENSP00000405718 | PNMA6A | 0.2579 |
| ENSP00000357986 | PLEKHA1 | 0.2579 |
| ENSP00000371073 | PLCXD1 | 0.2579 |
| ENSP00000381589 | PCDHGA11 | 0.2579 |
| ENSP00000353343 | OR52M1 | 0.2579 |
| ENSP00000391397 | MT1G | 0.2579 |

| | | |
|---|---|---|
| ENSP00000307093 | MAP6 | 0.2579 |
| ENSP00000300591 | LOXHD1 | 0.2579 |
| ENSP00000381298 | LONRF1 | 0.2579 |
| ENSP00000353893 | LGALS14 | 0.2579 |
| ENSP00000316737 | LCE1D | 0.2579 |
| ENSP00000334287 | KRTAP21-2 | 0.2579 |
| ENSP00000368872 | INSC | 0.2579 |
| ENSP00000385422 | GPR61 | 0.2579 |
| ENSP00000327417 | GPR39 | 0.2579 |
| ENSP00000367125 | GPR153 | 0.2579 |
| ENSP00000365550 | GKAP1 | 0.2579 |
| ENSP00000368547 | FEZ2 | 0.2579 |
| ENSP00000258200 | FBXL8 | 0.2579 |
| ENSP00000318974 | FAM133A | 0.2579 |
| ENSP00000419235 | FAM115A | 0.2579 |
| ENSP00000261486 | EPB41L4A | 0.2579 |
| ENSP00000274458 | COMMD10 | 0.2579 |
| ENSP00000262622 | CHST8 | 0.2579 |
| ENSP00000309649 | CA5A | 0.2579 |
| ENSP00000322108 | C9orf84 | 0.2579 |
| ENSP00000331167 | C11orf35 | 0.2579 |
| ENSP00000397759 | BSDC1 | 0.2579 |
| ENSP00000332788 | ANO9 | 0.2579 |
| ENSP00000326572 | ANKRD62 | 0.2579 |
| ENSP00000313809 | AMBN | 0.2579 |
| ENSP00000300176 | AGFG2 | 0.2579 |
| ENSP00000423134 | ENSG00000250765 | 0.2579 |
| ENSP00000332325 | ZNF703 | 0.2553 |
| ENSP00000296600 | ZNF474 | 0.2553 |
| ENSP00000354964 | ZNF318 | 0.2553 |
| ENSP00000253159 | ZNF236 | 0.2553 |
| ENSP00000380087 | ZNF213 | 0.2553 |
| ENSP00000345392 | ZNF2 | 0.2553 |
| ENSP00000262259 | ZNF175 | 0.2553 |
| ENSP00000254166 | ZNF132 | 0.2553 |
| ENSP00000278590 | ZC3H12C | 0.2553 |
| ENSP00000346603 | ZBTB45 | 0.2553 |
| ENSP00000365899 | VSX1 | 0.2553 |
| ENSP00000322339 | VN1R1 | 0.2553 |
| ENSP00000257632 | UPK3B | 0.2553 |
| ENSP00000281441 | TMEM45B | 0.2553 |
| ENSP00000358559 | SYT6 | 0.2553 |
| ENSP00000366827 | ST8SIA6 | 0.2553 |
| ENSP00000301717 | SPSB3 | 0.2553 |
| ENSP00000338034 | SPRYD4 | 0.2553 |
| ENSP00000364054 | SPIN3 | 0.2553 |
| ENSP00000375909 | SPHKAP | 0.2553 |
| ENSP00000380354 | SPATA22 | 0.2553 |
| ENSP00000302400 | SMR3B | 0.2553 |
| ENSP00000390750 | SMIM19 | 0.2553 |
| ENSP00000298966 | SMCO4 | 0.2553 |
| ENSP00000301335 | SLC43A2 | 0.2553 |
| ENSP00000428331 | SFTA3 | 0.2553 |

| | | |
|---|---|---|
| ENSP00000285947 | SETD9 | 0.2553 |
| ENSP00000384182 | SCFD2 | 0.2553 |
| ENSP00000431198 | RP11-585F1.10 | 0.2553 |
| ENSP00000295755 | RETNLB | 0.2553 |
| ENSP00000401328 | PRSS48 | 0.2553 |
| ENSP00000256103 | PMP2 | 0.2553 |
| ENSP00000385892 | PLEKHB2 | 0.2553 |
| ENSP00000366876 | PHF13 | 0.2553 |
| ENSP00000381605 | PCDHGA8 | 0.2553 |
| ENSP00000304419 | OR2B2 | 0.2553 |
| ENSP00000254627 | OC90 | 0.2553 |
| ENSP00000329295 | NXPH3 | 0.2553 |
| ENSP00000323184 | MYCBPAP | 0.2553 |
| ENSP00000263268 | MREG | 0.2553 |
| ENSP00000442688 | MFSD5 | 0.2553 |
| ENSP00000371345 | METTL22 | 0.2553 |
| ENSP00000315731 | METRNL | 0.2553 |
| ENSP00000267102 | LMBR1L | 0.2553 |
| ENSP00000463483 | KRT222 | 0.2553 |
| ENSP00000156499 | KLK14 | 0.2553 |
| ENSP00000344424 | KIAA0247 | 0.2553 |
| ENSP00000368402 | KCTD4 | 0.2553 |
| ENSP00000279392 | HIRIP3 | 0.2553 |
| ENSP00000344125 | GTDC2 | 0.2553 |
| ENSP00000328672 | GPRIN3 | 0.2553 |
| ENSP00000369344 | GPR150 | 0.2553 |
| ENSP00000455298 | GOLGA8S | 0.2553 |
| ENSP00000399637 | GOLGA8I | 0.2553 |
| ENSP00000376548 | GALNTL5 | 0.2553 |
| ENSP00000227756 | GALNT18 | 0.2553 |
| ENSP00000306888 | FAM151A | 0.2553 |
| ENSP00000359820 | FAM122C | 0.2553 |
| ENSP00000354461 | FAM109A | 0.2553 |
| ENSP00000296420 | EMCN | 0.2553 |
| ENSP00000342012 | DMKN | 0.2553 |
| ENSP00000262585 | DENND3 | 0.2553 |
| ENSP00000276127 | CPXCR1 | 0.2553 |
| ENSP00000251166 | CORO7 | 0.2553 |
| ENSP00000387209 | CEP19 | 0.2553 |
| ENSP00000376250 | CCDC50 | 0.2553 |
| ENSP00000330240 | CCDC34 | 0.2553 |
| ENSP00000395706 | CCDC14 | 0.2553 |
| ENSP00000379310 | CASC1 | 0.2553 |
| ENSP00000357106 | CADM3 | 0.2553 |
| ENSP00000349358 | C12orf29 | 0.2553 |
| ENSP00000367878 | C11orf49 | 0.2553 |
| ENSP00000367104 | BFSP1 | 0.2553 |
| ENSP00000396976 | BAHD1 | 0.2553 |
| ENSP00000348635 | AMICA1 | 0.2553 |
| ENSP00000391200 | ZNF692 | 0.2526 |
| ENSP00000320188 | ZNF579 | 0.2526 |
| ENSP00000347045 | ZNF43 | 0.2526 |
| ENSP00000268655 | ZNF174 | 0.2526 |

| | | |
|---|---|---|
| ENSP00000371715 | ZG16B | 0.2526 |
| ENSP00000274030 | USP53 | 0.2526 |
| ENSP00000350716 | TUSC1 | 0.2526 |
| ENSP00000310829 | TTC18 | 0.2526 |
| ENSP00000039989 | TTC17 | 0.2526 |
| ENSP00000334708 | TMEM150A | 0.2526 |
| ENSP00000418803 | TIMMDC1 | 0.2526 |
| ENSP00000240615 | TAS2R8 | 0.2526 |
| ENSP00000456609 | STRA6 | 0.2526 |
| ENSP00000265404 | STAP1 | 0.2526 |
| ENSP00000414302 | SRSF12 | 0.2526 |
| ENSP00000328149 | SPATA8 | 0.2526 |
| ENSP00000422185 | SORCS2 | 0.2526 |
| ENSP00000370669 | SLC8A3 | 0.2526 |
| ENSP00000424424 | SLC4A9 | 0.2526 |
| ENSP00000352497 | SLC25A44 | 0.2526 |
| ENSP00000263512 | SLC10A3 | 0.2526 |
| ENSP00000264313 | SLAIN2 | 0.2526 |
| ENSP00000345243 | SIGLEC10 | 0.2526 |
| ENSP00000442339 | SAP25 | 0.2526 |
| ENSP00000447650 | RP11-644F5.10 | 0.2526 |
| ENSP00000368144 | PRR5L | 0.2526 |
| ENSP00000318270 | PROM2 | 0.2526 |
| ENSP00000368720 | PLEKHN1 | 0.2526 |
| ENSP00000295645 | PDCL2 | 0.2526 |
| ENSP00000306095 | OR10H2 | 0.2526 |
| ENSP00000273347 | NXPE3 | 0.2526 |
| ENSP00000450924 | NKX1-2 | 0.2526 |
| ENSP00000361596 | NEURL2 | 0.2526 |
| ENSP00000315878 | MS4A6A | 0.2526 |
| ENSP00000366095 | MOG | 0.2526 |
| ENSP00000333539 | MIPOL1 | 0.2526 |
| ENSP00000333183 | MBD3L3 | 0.2526 |
| ENSP00000209718 | KRT23 | 0.2526 |
| ENSP00000339340 | KCTD21 | 0.2526 |
| ENSP00000262487 | ISM1 | 0.2526 |
| ENSP00000242607 | HVCN1 | 0.2526 |
| ENSP00000398064 | HIGD1A | 0.2526 |
| ENSP00000182377 | FAR2 | 0.2526 |
| ENSP00000288228 | FAM81A | 0.2526 |
| ENSP00000365080 | FAM155A | 0.2526 |
| ENSP00000282041 | EPG5 | 0.2526 |
| ENSP00000256545 | EMC7 | 0.2526 |
| ENSP00000315098 | EDDM3A | 0.2526 |
| ENSP00000288710 | DRC1 | 0.2526 |
| ENSP00000417710 | DPPA2 | 0.2526 |
| ENSP00000359788 | DDX26B | 0.2526 |
| ENSP00000311313 | CST6 | 0.2526 |
| ENSP00000296953 | CREBRF | 0.2526 |
| ENSP00000223336 | COA1 | 0.2526 |
| ENSP00000328487 | CCDC87 | 0.2526 |
| ENSP00000344655 | CCDC41 | 0.2526 |
| ENSP00000368503 | C4orf46 | 0.2526 |

| | | |
|---|---|---|
| ENSP00000356886 | C1orf110 | 0.2526 |
| ENSP00000005260 | BAIAP2L1 | 0.2526 |
| ENSP00000313422 | ARL6IP4 | 0.2526 |
| ENSP00000314103 | ANKRD34A | 0.2526 |
| ENSP00000299164 | ADAMTS15 | 0.2526 |
| ENSP00000349161 | ZNF790 | 0.2500 |
| ENSP00000409463 | ZNF740 | 0.2500 |
| ENSP00000349098 | ZNF74 | 0.2500 |
| ENSP00000347730 | ZNF536 | 0.2500 |
| ENSP00000406201 | ZNF160 | 0.2500 |
| ENSP00000392095 | ZNF146 | 0.2500 |
| ENSP00000286760 | WHAMM | 0.2500 |
| ENSP00000429216 | TRNP1 | 0.2500 |
| ENSP00000291416 | TRIM62 | 0.2500 |
| ENSP00000338989 | TRIM16 | 0.2500 |
| ENSP00000347748 | TPRA1 | 0.2500 |
| ENSP00000334611 | TMPRSS11A | 0.2500 |
| ENSP00000338607 | THEM6 | 0.2500 |
| ENSP00000369500 | SYAP1 | 0.2500 |
| ENSP00000382231 | SMIM11 | 0.2500 |
| ENSP00000296327 | SLC51A | 0.2500 |
| ENSP00000353677 | SLC17A2 | 0.2500 |
| ENSP00000405676 | RP11-565P22.6 | 0.2500 |
| ENSP00000419057 | RP11-159G9.5 | 0.2500 |
| ENSP00000334958 | RLTPR | 0.2500 |
| ENSP00000354963 | RASGEF1C | 0.2500 |
| ENSP00000325589 | PTPRCAP | 0.2500 |
| ENSP00000374013 | PRB2 | 0.2500 |
| ENSP00000292330 | PPP1R35 | 0.2500 |
| ENSP00000340125 | PPP1R17 | 0.2500 |
| ENSP00000352458 | PLGLB2 | 0.2500 |
| ENSP00000329403 | PCP4 | 0.2500 |
| ENSP00000429018 | PCDHGB2 | 0.2500 |
| ENSP00000252085 | PCDHGA12 | 0.2500 |
| ENSP00000349719 | OR2L8 | 0.2500 |
| ENSP00000331545 | OR1G1 | 0.2500 |
| ENSP00000424126 | MCTP1 | 0.2500 |
| ENSP00000331435 | MBD3L5 | 0.2500 |
| ENSP00000370801 | MBD3L4 | 0.2500 |
| ENSP00000303758 | LUZP1 | 0.2500 |
| ENSP00000343331 | LIPI | 0.2500 |
| ENSP00000318607 | LDLRAD3 | 0.2500 |
| ENSP00000296564 | KIAA0947 | 0.2500 |
| ENSP00000321544 | KBTBD11 | 0.2500 |
| ENSP00000366234 | IL31 | 0.2500 |
| ENSP00000341267 | HMGN3 | 0.2500 |
| ENSP00000299192 | HEATR3 | 0.2500 |
| ENSP00000363433 | GPRIN2 | 0.2500 |
| ENSP00000363136 | GPR3 | 0.2500 |
| ENSP00000355551 | GPR137B | 0.2500 |
| ENSP00000370844 | GPR12 | 0.2500 |
| ENSP00000446479 | FICD | 0.2500 |
| ENSP00000431459 | FAM89B | 0.2500 |

| | | |
|---|---|---|
| ENSP00000352955 | FAM3A | 0.2500 |
| ENSP00000419088 | FAM162A | 0.2500 |
| ENSP00000280057 | FAM124A | 0.2500 |
| ENSP00000331734 | DSCR6 | 0.2500 |
| ENSP00000257215 | DAGLA | 0.2500 |
| ENSP00000301391 | CYB5D2 | 0.2500 |
| ENSP00000227348 | CRTAM | 0.2500 |
| ENSP00000223208 | CEP41 | 0.2500 |
| ENSP00000338258 | CDC42EP4 | 0.2500 |
| ENSP00000221922 | CCDC9 | 0.2500 |
| ENSP00000298050 | CCDC67 | 0.2500 |
| ENSP00000285273 | CA10 | 0.2500 |
| ENSP00000318119 | C9orf139 | 0.2500 |
| ENSP00000347301 | BEGAIN | 0.2500 |
| ENSP00000384894 | AQP12B | 0.2500 |
| ENSP00000409937 | ALS2CR11 | 0.2500 |
| ENSP00000368761 | ZNF827 | 0.2474 |
| ENSP00000352836 | ZNF675 | 0.2474 |
| ENSP00000366853 | ZNF658 | 0.2474 |
| ENSP00000363747 | ZNF33A | 0.2474 |
| ENSP00000255198 | ZBED3 | 0.2474 |
| ENSP00000289953 | WFDC8 | 0.2474 |
| ENSP00000383599 | WDR47 | 0.2474 |
| ENSP00000355932 | VASH2 | 0.2474 |
| ENSP00000311219 | TRIM59 | 0.2474 |
| ENSP00000301924 | TRIM35 | 0.2474 |
| ENSP00000305664 | TRABD | 0.2474 |
| ENSP00000348639 | TMPRSS11F | 0.2474 |
| ENSP00000258439 | TMEM127 | 0.2474 |
| ENSP00000355323 | TEX28 | 0.2474 |
| ENSP00000385347 | TAPT1 | 0.2474 |
| ENSP00000289805 | SPATA2L | 0.2474 |
| ENSP00000312774 | SPACA4 | 0.2474 |
| ENSP00000269053 | SPACA3 | 0.2474 |
| ENSP00000333519 | SLC24A3 | 0.2474 |
| ENSP00000266560 | RBP5 | 0.2474 |
| ENSP00000384160 | RBM33 | 0.2474 |
| ENSP00000405932 | PROX2 | 0.2474 |
| ENSP00000329880 | PHACTR1 | 0.2474 |
| ENSP00000274569 | PCYOX1L | 0.2474 |
| ENSP00000461862 | PCDHGB3 | 0.2474 |
| ENSP00000430024 | PCDHGA7 | 0.2474 |
| ENSP00000307726 | OR2C1 | 0.2474 |
| ENSP00000371786 | ONECUT3 | 0.2474 |
| ENSP00000384551 | NXPH1 | 0.2474 |
| ENSP00000311687 | NIPAL4 | 0.2474 |
| ENSP00000308330 | MYEOV | 0.2474 |
| ENSP00000307706 | MT1E | 0.2474 |
| ENSP00000256441 | MRPS36 | 0.2474 |
| ENSP00000312834 | MLXIP | 0.2474 |
| ENSP00000258436 | MFSD9 | 0.2474 |
| ENSP00000329918 | MEX3B | 0.2474 |
| ENSP00000359339 | MB21D1 | 0.2474 |

| | | |
|---|---|---|
| ENSP00000243314 | MAGEA9B | 0.2474 |
| ENSP00000419901 | L1TD1 | 0.2474 |
| ENSP00000365756 | KIAA2013 | 0.2474 |
| ENSP00000279024 | KIAA1755 | 0.2474 |
| ENSP00000413445 | KIAA1324L | 0.2474 |
| ENSP00000435150 | KIAA0408 | 0.2474 |
| ENSP00000262580 | GSDMD | 0.2474 |
| ENSP00000290795 | GPBP1L1 | 0.2474 |
| ENSP00000255945 | GIMAP4 | 0.2474 |
| ENSP00000363709 | FRRS1L | 0.2474 |
| ENSP00000298223 | FOLR2 | 0.2474 |
| ENSP00000344855 | FBXL14 | 0.2474 |
| ENSP00000335082 | FAM91A1 | 0.2474 |
| ENSP00000328426 | FAM208B | 0.2474 |
| ENSP00000329040 | FAM174B | 0.2474 |
| ENSP00000422338 | FAM173B | 0.2474 |
| ENSP00000340427 | ERMP1 | 0.2474 |
| ENSP00000295571 | EOGT | 0.2474 |
| ENSP00000339587 | DFNA5 | 0.2474 |
| ENSP00000345374 | CXXC11 | 0.2474 |
| ENSP00000274368 | CRHBP | 0.2474 |
| ENSP00000262428 | COTL1 | 0.2474 |
| ENSP00000348074 | CLEC9A | 0.2474 |
| ENSP00000361929 | CERCAM | 0.2474 |
| ENSP00000262717 | CDH20 | 0.2474 |
| ENSP00000314099 | CA5B | 0.2474 |
| ENSP00000340220 | C7orf26 | 0.2474 |
| ENSP00000255174 | C20orf111 | 0.2474 |
| ENSP00000215376 | C19orf26 | 0.2474 |
| ENSP00000317671 | C12orf40 | 0.2474 |
| ENSP00000359674 | C10orf62 | 0.2474 |
| ENSP00000319281 | BASP1 | 0.2474 |
| ENSP00000250693 | ART1 | 0.2474 |
| ENSP00000324277 | APOLD1 | 0.2474 |
| ENSP00000315371 | ANO5 | 0.2474 |
| ENSP00000284268 | ANKH | 0.2474 |
| ENSP00000366015 | ANGPTL7 | 0.2474 |
| ENSP00000255152 | ZSWIM3 | 0.2447 |
| ENSP00000321963 | ZSCAN4 | 0.2447 |
| ENSP00000323879 | ZNRF2 | 0.2447 |
| ENSP00000396402 | ZNF793 | 0.2447 |
| ENSP00000391641 | ZNF780B | 0.2447 |
| ENSP00000365906 | ZNF615 | 0.2447 |
| ENSP00000338860 | ZNF444 | 0.2447 |
| ENSP00000211936 | ZNF184 | 0.2447 |
| ENSP00000277540 | WDR85 | 0.2447 |
| ENSP00000329654 | WBSCR17 | 0.2447 |
| ENSP00000353971 | TTI2 | 0.2447 |
| ENSP00000226230 | TMEM97 | 0.2447 |
| ENSP00000281961 | TMEM178A | 0.2447 |
| ENSP00000304032 | STARD5 | 0.2447 |
| ENSP00000418593 | SNX21 | 0.2447 |
| ENSP00000332062 | SNX20 | 0.2447 |

| | | |
|---|---|---|
| ENSP00000327467 | SMDT1 | 0.2447 |
| ENSP00000338627 | SLC6A16 | 0.2447 |
| ENSP00000325414 | SERTAD3 | 0.2447 |
| ENSP00000294435 | RBP7 | 0.2447 |
| ENSP00000357122 | PYHIN1 | 0.2447 |
| ENSP00000465932 | PRCD | 0.2447 |
| ENSP00000344140 | PPP1R32 | 0.2447 |
| ENSP00000447852 | POC1B-GALNT4 | 0.2447 |
| ENSP00000247226 | PLEKHG3 | 0.2447 |
| ENSP00000356634 | PAPPA2 | 0.2447 |
| ENSP00000322939 | OR56B1 | 0.2447 |
| ENSP00000327585 | OR1D2 | 0.2447 |
| ENSP00000330075 | OGFOD3 | 0.2447 |
| ENSP00000363431 | NPY4R | 0.2447 |
| ENSP00000310631 | MRGPRD | 0.2447 |
| ENSP00000264968 | MGAT4A | 0.2447 |
| ENSP00000334219 | MEGF8 | 0.2447 |
| ENSP00000426359 | MCIDAS | 0.2447 |
| ENSP00000328410 | MACC1 | 0.2447 |
| ENSP00000379651 | LILRA6 | 0.2447 |
| ENSP00000417116 | LBX2 | 0.2447 |
| ENSP00000375238 | KRTAP2-1 | 0.2447 |
| ENSP00000452237 | KRT86 | 0.2447 |
| ENSP00000225899 | KRT32 | 0.2447 |
| ENSP00000355720 | JMJD4 | 0.2447 |
| ENSP00000410925 | GPR42 | 0.2447 |
| ENSP00000296862 | GPR111 | 0.2447 |
| ENSP00000456927 | GOLGA8M | 0.2447 |
| ENSP00000223145 | GLCCI1 | 0.2447 |
| ENSP00000307617 | GGNBP2 | 0.2447 |
| ENSP00000336729 | GALNT16 | 0.2447 |
| ENSP00000366233 | GABBR1 | 0.2447 |
| ENSP00000379026 | FBXW10 | 0.2447 |
| ENSP00000374328 | FAXC | 0.2447 |
| ENSP00000380734 | FAM98B | 0.2447 |
| ENSP00000362187 | FAM102A | 0.2447 |
| ENSP00000358857 | EMD | 0.2447 |
| ENSP00000389455 | DIRC3 | 0.2447 |
| ENSP00000216775 | CPNE6 | 0.2447 |
| ENSP00000450144 | CHURC1 | 0.2447 |
| ENSP00000309285 | CCDC96 | 0.2447 |
| ENSP00000330361 | C8orf33 | 0.2447 |
| ENSP00000367330 | C12orf73 | 0.2447 |
| ENSP00000325508 | C11orf71 | 0.2447 |
| ENSP00000299353 | C10ORF32 | 0.2447 |
| ENSP00000361281 | BEST4 | 0.2447 |
| ENSP00000337144 | AQP12A | 0.2447 |
| ENSP00000358105 | APH1A | 0.2447 |
| ENSP00000285243 | ANKRD40 | 0.2447 |
| ENSP00000308606 | AGR3 | 0.2447 |
| ENSP00000361083 | ADIRF | 0.2447 |
| ENSP00000415978 | FAM74A2 | 0.2447 |
| ENSP00000282292 | ZNF773 | 0.2421 |

| | | |
|---|---|---|
| ENSP00000380237 | ZNF691 | 0.2421 |
| ENSP00000324441 | ZNF610 | 0.2421 |
| ENSP00000252840 | ZNF557 | 0.2421 |
| ENSP00000347755 | ZNF140 | 0.2421 |
| ENSP00000250160 | WISP1 | 0.2421 |
| ENSP00000362111 | TSPAN6 | 0.2421 |
| ENSP00000249861 | THAP10 | 0.2421 |
| ENSP00000373539 | STAB2 | 0.2421 |
| ENSP00000225276 | ST6GALNAC2 | 0.2421 |
| ENSP00000290902 | SPON2 | 0.2421 |
| ENSP00000417806 | SMIM4 | 0.2421 |
| ENSP00000335557 | SLX4IP | 0.2421 |
| ENSP00000367137 | SLC17A4 | 0.2421 |
| ENSP00000385899 | SDK1 | 0.2421 |
| ENSP00000451369 | RP11-371E8.4 | 0.2421 |
| ENSP00000310335 | REP15 | 0.2421 |
| ENSP00000240651 | PYROXD1 | 0.2421 |
| ENSP00000354498 | PHLDB1 | 0.2421 |
| ENSP00000322530 | PGBD5 | 0.2421 |
| ENSP00000226524 | PF4V1 | 0.2421 |
| ENSP00000379691 | PDXDC1 | 0.2421 |
| ENSP00000265192 | PAIP2 | 0.2421 |
| ENSP00000371377 | OR1A2 | 0.2421 |
| ENSP00000305207 | OR1A1 | 0.2421 |
| ENSP00000306070 | NUDT6 | 0.2421 |
| ENSP00000346335 | NRDE2 | 0.2421 |
| ENSP00000411584 | MSANTD1 | 0.2421 |
| ENSP00000379156 | MPRIP | 0.2421 |
| ENSP00000245551 | MIF4GD | 0.2421 |
| ENSP00000362136 | MANEAL | 0.2421 |
| ENSP00000310880 | MAL | 0.2421 |
| ENSP00000410481 | LTB | 0.2421 |
| ENSP00000377558 | KRT35 | 0.2421 |
| ENSP00000251691 | KIAA1244 | 0.2421 |
| ENSP00000261244 | KIAA0586 | 0.2421 |
| ENSP00000299732 | IQCD | 0.2421 |
| ENSP00000306523 | INSM2 | 0.2421 |
| ENSP00000259212 | IL36RN | 0.2421 |
| ENSP00000348815 | HYLS1 | 0.2421 |
| ENSP00000374268 | GXYLT2 | 0.2421 |
| ENSP00000331600 | GPR173 | 0.2421 |
| ENSP00000455826 | GOLGA8T | 0.2421 |
| ENSP00000426691 | GOLGA8K | 0.2421 |
| ENSP00000456401 | GOLGA8J | 0.2421 |
| ENSP00000301329 | GLOD4 | 0.2421 |
| ENSP00000223293 | GIMAP2 | 0.2421 |
| ENSP00000305334 | FSTL5 | 0.2421 |
| ENSP00000308292 | FAM170B | 0.2421 |
| ENSP00000289921 | FAM160B2 | 0.2421 |
| ENSP00000297435 | DEFA4 | 0.2421 |
| ENSP00000417289 | CTAGE8 | 0.2421 |
| ENSP00000420384 | CST7 | 0.2421 |
| ENSP00000361926 | CNPY3 | 0.2421 |

| | | |
|---|---|---|
| ENSP00000255784 | CCDC134 | 0.2421 |
| ENSP00000408730 | CCDC124 | 0.2421 |
| ENSP00000313816 | CCDC111 | 0.2421 |
| ENSP00000360642 | CC2D1B | 0.2421 |
| ENSP00000349732 | C3orf18 | 0.2421 |
| ENSP00000382794 | C14orf164 | 0.2421 |
| ENSP00000434385 | ADIG | 0.2421 |
| ENSP00000377078 | AAMDC | 0.2421 |
| ENSP00000467286 | ZNF763 | 0.2395 |
| ENSP00000469582 | ZNF729 | 0.2395 |
| ENSP00000364405 | ZNF367 | 0.2395 |
| ENSP00000307774 | ZNF239 | 0.2395 |
| ENSP00000315664 | ZNF18 | 0.2395 |
| ENSP00000361800 | WDR34 | 0.2395 |
| ENSP00000350102 | TOR4A | 0.2395 |
| ENSP00000256255 | TMEM66 | 0.2395 |
| ENSP00000312615 | TMEM134 | 0.2395 |
| ENSP00000407497 | SPINK8 | 0.2395 |
| ENSP00000388741 | SPATA3 | 0.2395 |
| ENSP00000358794 | SLC16A4 | 0.2395 |
| ENSP00000219315 | SETD6 | 0.2395 |
| ENSP00000356071 | SERAC1 | 0.2395 |
| ENSP00000431971 | SAMD1 | 0.2395 |
| ENSP00000218340 | RP2 | 0.2395 |
| ENSP00000312134 | PRG2 | 0.2395 |
| ENSP00000218224 | PQBP1 | 0.2395 |
| ENSP00000417038 | PHACTR2 | 0.2395 |
| ENSP00000363242 | PGBD3 | 0.2395 |
| ENSP00000300778 | OR51Q1 | 0.2395 |
| ENSP00000326225 | OR2T8 | 0.2395 |
| ENSP00000265634 | NPTX2 | 0.2395 |
| ENSP00000395232 | MTFR2 | 0.2395 |
| ENSP00000359819 | MOSPD1 | 0.2395 |
| ENSP00000287701 | HMBOX1 | 0.2395 |
| ENSP00000264779 | GPBP1 | 0.2395 |
| ENSP00000398615 | GOLGA6L6 | 0.2395 |
| ENSP00000278187 | GAS2 | 0.2395 |
| ENSP00000332757 | FUT10 | 0.2395 |
| ENSP00000221498 | DKKL1 | 0.2395 |
| ENSP00000417128 | DEFB121 | 0.2395 |
| ENSP00000310901 | CSRP2 | 0.2395 |
| ENSP00000326538 | CLEC18C | 0.2395 |
| ENSP00000297788 | CCDC136 | 0.2395 |
| ENSP00000318912 | CA13 | 0.2395 |
| ENSP00000284437 | C4orf19 | 0.2395 |
| ENSP00000295966 | C3orf67 | 0.2395 |
| ENSP00000362376 | C10orf35 | 0.2395 |
| ENSP00000328698 | C10orf107 | 0.2395 |
| ENSP00000338072 | AVPR2 | 0.2395 |
| ENSP00000350686 | ANKLE2 | 0.2395 |
| ENSP00000292530 | ZNF333 | 0.2368 |
| ENSP00000366542 | ZNF165 | 0.2368 |
| ENSP00000343581 | ZFP30 | 0.2368 |

| | | |
|---|---|---|
| ENSP00000261789 | TM9SF1 | 0.2368 |
| ENSP00000268138 | TICRR | 0.2368 |
| ENSP00000258991 | TEX2 | 0.2368 |
| ENSP00000385122 | SYCE3 | 0.2368 |
| ENSP00000309189 | SPERT | 0.2368 |
| ENSP00000261369 | SNX24 | 0.2368 |
| ENSP00000313121 | SNX14 | 0.2368 |
| ENSP00000371036 | SIRPD | 0.2368 |
| ENSP00000370126 | SERF1B | 0.2368 |
| ENSP00000297613 | RPP25L | 0.2368 |
| ENSP00000282003 | RNF219 | 0.2368 |
| ENSP00000303276 | RNASE2 | 0.2368 |
| ENSP00000280571 | RILPL2 | 0.2368 |
| ENSP00000354361 | RFWD3 | 0.2368 |
| ENSP00000264644 | RAPGEFL1 | 0.2368 |
| ENSP00000253796 | RAMP2 | 0.2368 |
| ENSP00000387654 | PCYOX1 | 0.2368 |
| ENSP00000441802 | PCDH7 | 0.2368 |
| ENSP00000341171 | MTSS1L | 0.2368 |
| ENSP00000343212 | MAP3K7CL | 0.2368 |
| ENSP00000472698 | L34079.2 | 0.2368 |
| ENSP00000405032 | GSG1 | 0.2368 |
| ENSP00000348550 | FRMD6 | 0.2368 |
| ENSP00000237281 | FBXO30 | 0.2368 |
| ENSP00000442365 | EML2 | 0.2368 |
| ENSP00000369976 | EGFL6 | 0.2368 |
| ENSP00000320303 | DNAJC28 | 0.2368 |
| ENSP00000253381 | DEFB118 | 0.2368 |
| ENSP00000339511 | CXorf27 | 0.2368 |
| ENSP00000337209 | CCDC68 | 0.2368 |
| ENSP00000370077 | CCDC171 | 0.2368 |
| ENSP00000426159 | C6orf223 | 0.2368 |
| ENSP00000326879 | C5orf22 | 0.2368 |
| ENSP00000231512 | C5orf15 | 0.2368 |
| ENSP00000384965 | C22orf15 | 0.2368 |
| ENSP00000394472 | BTBD18 | 0.2368 |
| ENSP00000379290 | BCAS1 | 0.2368 |
| ENSP00000381844 | ATL3 | 0.2368 |
| ENSP00000298694 | ARHGEF40 | 0.2368 |
| ENSP00000216099 | APOBEC3D | 0.2368 |
| ENSP00000234816 | ANGPTL1 | 0.2368 |
| ENSP00000426528 | AMER2 | 0.2368 |
| ENSP00000374071 | ADAMTS20 | 0.2368 |
| ENSP00000249005 | A4GALT | 0.2368 |
| ENSP00000300619 | ZNF91 | 0.2342 |
| ENSP00000374185 | ZNF841 | 0.2342 |
| ENSP00000254321 | ZNF700 | 0.2342 |
| ENSP00000324605 | ZNF660 | 0.2342 |
| ENSP00000322427 | ZNF611 | 0.2342 |
| ENSP00000382237 | TMEM26 | 0.2342 |
| ENSP00000383594 | TMEM242 | 0.2342 |
| ENSP00000379901 | TMEM106B | 0.2342 |
| ENSP00000331500 | TEX19 | 0.2342 |

| | | |
|---|---|---|
| ENSP00000415564 | TCEAL4 | 0.2342 |
| ENSP00000363573 | SRSF10 | 0.2342 |
| ENSP00000377176 | SPATA7 | 0.2342 |
| ENSP00000326070 | SLC41A3 | 0.2342 |
| ENSP00000295959 | RPP14 | 0.2342 |
| ENSP00000275423 | RBAK | 0.2342 |
| ENSP00000356574 | QSOX1 | 0.2342 |
| ENSP00000165524 | PRLH | 0.2342 |
| ENSP00000301286 | PLIN4 | 0.2342 |
| ENSP00000381628 | PCDHA12 | 0.2342 |
| ENSP00000357448 | PBXIP1 | 0.2342 |
| ENSP00000323872 | P2RY11 | 0.2342 |
| ENSP00000263084 | OVCA2 | 0.2342 |
| ENSP00000461388 | OVCA2 | 0.2342 |
| ENSP00000356110 | NUCKS1 | 0.2342 |
| ENSP00000382883 | MAGEB17 | 0.2342 |
| ENSP00000230538 | LAMA4 | 0.2342 |
| ENSP00000332805 | KRTAP8-1 | 0.2342 |
| ENSP00000375430 | KRTAP3-1 | 0.2342 |
| ENSP00000382777 | KRTAP13-2 | 0.2342 |
| ENSP00000371328 | KLRC3 | 0.2342 |
| ENSP00000193391 | IMPG2 | 0.2342 |
| ENSP00000341794 | IL1F10 | 0.2342 |
| ENSP00000255039 | HAPLN2 | 0.2342 |
| ENSP00000333744 | GPR19 | 0.2342 |
| ENSP00000307831 | GPR113 | 0.2342 |
| ENSP00000298110 | GPR101 | 0.2342 |
| ENSP00000349884 | GALP | 0.2342 |
| ENSP00000265825 | FSCN3 | 0.2342 |
| ENSP00000344572 | FIBP | 0.2342 |
| ENSP00000296438 | FBXW12 | 0.2342 |
| ENSP00000307798 | FAM90A1 | 0.2342 |
| ENSP00000400099 | FAM65A | 0.2342 |
| ENSP00000364814 | FAM131C | 0.2342 |
| ENSP00000432104 | EBLN2 | 0.2342 |
| ENSP00000344432 | DOK7 | 0.2342 |
| ENSP00000354830 | DDIT4L | 0.2342 |
| ENSP00000307132 | CST5 | 0.2342 |
| ENSP00000304544 | COMMD5 | 0.2342 |
| ENSP00000326407 | CLEC1A | 0.2342 |
| ENSP00000284224 | CHST9 | 0.2342 |
| ENSP00000365401 | CCDC22 | 0.2342 |
| ENSP00000412150 | CCDC121 | 0.2342 |
| ENSP00000364260 | C9orf156 | 0.2342 |
| ENSP00000448536 | C12orf75 | 0.2342 |
| ENSP00000339637 | BRAT1 | 0.2342 |
| ENSP00000442521 | BEX2 | 0.2342 |
| ENSP00000356404 | B3GALT2 | 0.2342 |
| ENSP00000305502 | ASCC2 | 0.2342 |
| ENSP00000340297 | ARHGEF10 | 0.2342 |
| ENSP00000371983 | AFAP1 | 0.2342 |
| ENSP00000352846 | ZNF808 | 0.2316 |
| ENSP00000331927 | ZNF789 | 0.2316 |

| | | |
|---|---|---|
| ENSP00000311768 | ZNF706 | 0.2316 |
| ENSP00000380310 | ZNF676 | 0.2316 |
| ENSP00000319305 | ZNF572 | 0.2316 |
| ENSP00000376010 | ZNF566 | 0.2316 |
| ENSP00000407130 | ZNF410 | 0.2316 |
| ENSP00000324064 | ZNF354C | 0.2316 |
| ENSP00000411097 | ZNF275 | 0.2316 |
| ENSP00000380315 | ZNF208 | 0.2316 |
| ENSP00000349568 | WDR96 | 0.2316 |
| ENSP00000359160 | TRMT13 | 0.2316 |
| ENSP00000364938 | TMEM82 | 0.2316 |
| ENSP00000259746 | TMEM63B | 0.2316 |
| ENSP00000359449 | TMEM185A | 0.2316 |
| ENSP00000259782 | TINAG | 0.2316 |
| ENSP00000296125 | TGM4 | 0.2316 |
| ENSP00000357739 | SPRR2E | 0.2316 |
| ENSP00000254853 | SLC52A1 | 0.2316 |
| ENSP00000355467 | SCCPDH | 0.2316 |
| ENSP00000430242 | SBSN | 0.2316 |
| ENSP00000337732 | RUNDC3B | 0.2316 |
| ENSP00000314992 | RRH | 0.2316 |
| ENSP00000453573 | RP13-996F3.5 | 0.2316 |
| ENSP00000265981 | RNF141 | 0.2316 |
| ENSP00000385927 | RMDN1 | 0.2316 |
| ENSP00000366070 | RILPL1 | 0.2316 |
| ENSP00000254661 | RAMP1 | 0.2316 |
| ENSP00000333456 | RAI2 | 0.2316 |
| ENSP00000279575 | PRB4 | 0.2316 |
| ENSP00000380880 | PQLC1 | 0.2316 |
| ENSP00000264231 | POPDC2 | 0.2316 |
| ENSP00000346127 | PLEKHB1 | 0.2316 |
| ENSP00000463094 | PIEZO2 | 0.2316 |
| ENSP00000324403 | PHF21B | 0.2316 |
| ENSP00000312070 | PCDHGC3 | 0.2316 |
| ENSP00000231134 | PCDHB5 | 0.2316 |
| ENSP00000436557 | PCDHA5 | 0.2316 |
| ENSP00000244623 | OR2B6 | 0.2316 |
| ENSP00000407978 | NKX1-1 | 0.2316 |
| ENSP00000419000 | LSAMP | 0.2316 |
| ENSP00000221797 | LGALS13 | 0.2316 |
| ENSP00000257696 | HILPDA | 0.2316 |
| ENSP00000339750 | GTDC1 | 0.2316 |
| ENSP00000319673 | GPR89A | 0.2316 |
| ENSP00000261654 | GPR133 | 0.2316 |
| ENSP00000343890 | GLTPD1 | 0.2316 |
| ENSP00000370262 | FREM1 | 0.2316 |
| ENSP00000334364 | DEFB106B | 0.2316 |
| ENSP00000369009 | CXorf23 | 0.2316 |
| ENSP00000299663 | CLEC4E | 0.2316 |
| ENSP00000357149 | CD1E | 0.2316 |
| ENSP00000235933 | CD160 | 0.2316 |
| ENSP00000064571 | CBLN4 | 0.2316 |
| ENSP00000295622 | C3orf30 | 0.2316 |

| | | |
|---|---|---|
| ENSP00000332809 | C2orf71 | 0.2316 |
| ENSP00000373586 | C2orf54 | 0.2316 |
| ENSP00000360320 | C1orf177 | 0.2316 |
| ENSP00000319179 | C1orf172 | 0.2316 |
| ENSP00000326846 | C14orf28 | 0.2316 |
| ENSP00000377369 | C14orf166B | 0.2316 |
| ENSP00000360312 | BSND | 0.2316 |
| ENSP00000354536 | ASTN1 | 0.2316 |
| ENSP00000303570 | ANKMY2 | 0.2316 |
| ENSP00000362524 | ANGPTL2 | 0.2316 |
| ENSP00000346478 | ADAMTSL2 | 0.2316 |
| ENSP00000269829 | ZNF544 | 0.2289 |
| ENSP00000379168 | ZNF287 | 0.2289 |
| ENSP00000282869 | ZNF117 | 0.2289 |
| ENSP00000322872 | ZKSCAN5 | 0.2289 |
| ENSP00000285279 | VOPP1 | 0.2289 |
| ENSP00000329548 | TUSC5 | 0.2289 |
| ENSP00000422188 | TTC23L | 0.2289 |
| ENSP00000362122 | TNMD | 0.2289 |
| ENSP00000280734 | TMEM86A | 0.2289 |
| ENSP00000366243 | TMEM2 | 0.2289 |
| ENSP00000005286 | TMEM132A | 0.2289 |
| ENSP00000324651 | TMEM108 | 0.2289 |
| ENSP00000405482 | SVOPL | 0.2289 |
| ENSP00000322652 | SNX16 | 0.2289 |
| ENSP00000385025 | SMCR8 | 0.2289 |
| ENSP00000359376 | SLC17A9 | 0.2289 |
| ENSP00000326933 | SERTAD2 | 0.2289 |
| ENSP00000367173 | RNF207 | 0.2289 |
| ENSP00000351536 | QSOX2 | 0.2289 |
| ENSP00000396732 | PRDM7 | 0.2289 |
| ENSP00000349274 | PPP1R26 | 0.2289 |
| ENSP00000228705 | PPM1H | 0.2289 |
| ENSP00000414208 | POM121C | 0.2289 |
| ENSP00000382323 | OTOG | 0.2289 |
| ENSP00000324769 | OR4C6 | 0.2289 |
| ENSP00000272641 | NXPH2 | 0.2289 |
| ENSP00000371126 | NUTM2A | 0.2289 |
| ENSP00000307722 | NSG2 | 0.2289 |
| ENSP00000362841 | NKAIN1 | 0.2289 |
| ENSP00000347170 | NEIL1 | 0.2289 |
| ENSP00000257776 | MRAP2 | 0.2289 |
| ENSP00000381928 | MGARP | 0.2289 |
| ENSP00000354583 | MFAP3L | 0.2289 |
| ENSP00000244333 | LYPD3 | 0.2289 |
| ENSP00000301263 | LY6D | 0.2289 |
| ENSP00000311905 | LTBP4 | 0.2289 |
| ENSP00000360753 | LCN8 | 0.2289 |
| ENSP00000342215 | KIR2DL3 | 0.2289 |
| ENSP00000353129 | KCTD8 | 0.2289 |
| ENSP00000362836 | KCTD20 | 0.2289 |
| ENSP00000300098 | GPR182 | 0.2289 |
| ENSP00000379946 | GLTSCR1 | 0.2289 |

| | | |
|---|---|---|
| ENSP00000243913 | GCNT7 | 0.2289 |
| ENSP00000248598 | FGL2 | 0.2289 |
| ENSP00000363482 | FAM21C | 0.2289 |
| ENSP00000263256 | DESI1 | 0.2289 |
| ENSP00000319831 | DEF6 | 0.2289 |
| ENSP00000360167 | DCAF12L1 | 0.2289 |
| ENSP00000265136 | COBL | 0.2289 |
| ENSP00000295324 | CDC42EP3 | 0.2289 |
| ENSP00000296238 | CAMK2N2 | 0.2289 |
| ENSP00000399947 | ASAH2C | 0.2289 |
| ENSP00000299155 | AMN | 0.2289 |
| ENSP00000356632 | ABRACL | 0.2289 |
| ENSP00000366342 | ASB16-AS1 | 0.2289 |
| ENSP00000351482 | ZNF707 | 0.2263 |
| ENSP00000282296 | ZNF551 | 0.2263 |
| ENSP00000417424 | ZNF480 | 0.2263 |
| ENSP00000356064 | TULP4 | 0.2263 |
| ENSP00000402855 | TREML1 | 0.2263 |
| ENSP00000330264 | TMPRSS9 | 0.2263 |
| ENSP00000331640 | TMEM186 | 0.2263 |
| ENSP00000453399 | TMEM185B | 0.2263 |
| ENSP00000424707 | TMEM167A | 0.2263 |
| ENSP00000326244 | TMEM151A | 0.2263 |
| ENSP00000354774 | SPRYD7 | 0.2263 |
| ENSP00000405210 | SPANXN4 | 0.2263 |
| ENSP00000427407 | SMIM20 | 0.2263 |
| ENSP00000366645 | SCP2D1 | 0.2263 |
| ENSP00000350976 | RTP2 | 0.2263 |
| ENSP00000418667 | RGAG4 | 0.2263 |
| ENSP00000270077 | PSG9 | 0.2263 |
| ENSP00000401932 | PRSS45 | 0.2263 |
| ENSP00000378072 | PNPLA1 | 0.2263 |
| ENSP00000304192 | PCNX | 0.2263 |
| ENSP00000361486 | PCIF1 | 0.2263 |
| ENSP00000342448 | OR5W2 | 0.2263 |
| ENSP00000319601 | OR4N2 | 0.2263 |
| ENSP00000378516 | OR10W1 | 0.2263 |
| ENSP00000307552 | NINJ2 | 0.2263 |
| ENSP00000444617 | NHSL2 | 0.2263 |
| ENSP00000355198 | MAGEB3 | 0.2263 |
| ENSP00000327533 | LYG2 | 0.2263 |
| ENSP00000332103 | LSMD1 | 0.2263 |
| ENSP00000405165 | LIMS3 | 0.2263 |
| ENSP00000381488 | KRTAP9-1 | 0.2263 |
| ENSP00000359768 | KIAA1586 | 0.2263 |
| ENSP00000347178 | HSPB9 | 0.2263 |
| ENSP00000456894 | GOLGA8H | 0.2263 |
| ENSP00000369907 | FAM154A | 0.2263 |
| ENSP00000409256 | EFCAB4A | 0.2263 |
| ENSP00000371847 | DEFB125 | 0.2263 |
| ENSP00000264192 | CYTIP | 0.2263 |
| ENSP00000318258 | CSRNP3 | 0.2263 |
| ENSP00000359594 | CLCA4 | 0.2263 |

| | | |
|---|---|---|
| ENSP00000256785 | CFHR5 | 0.2263 |
| ENSP00000252992 | CEP85 | 0.2263 |
| ENSP00000361336 | CDH22 | 0.2263 |
| ENSP00000362085 | C9orf16 | 0.2263 |
| ENSP00000322061 | C7 | 0.2263 |
| ENSP00000386190 | C2orf16 | 0.2263 |
| ENSP00000355609 | C1orf131 | 0.2263 |
| ENSP00000284694 | C10orf90 | 0.2263 |
| ENSP00000400157 | B3GNT9 | 0.2263 |
| ENSP00000386280 | APOL6 | 0.2263 |
| ENSP00000357943 | ANXA9 | 0.2263 |
| ENSP00000379568 | ACBD5 | 0.2263 |
| ENSP00000360069 | ZNF831 | 0.2237 |
| ENSP00000440403 | ZNF678 | 0.2237 |
| ENSP00000422344 | ZNF550 | 0.2237 |
| ENSP00000278772 | ZNF343 | 0.2237 |
| ENSP00000262990 | ZNF330 | 0.2237 |
| ENSP00000418719 | ZNF273 | 0.2237 |
| ENSP00000457704 | ZNF10 | 0.2237 |
| ENSP00000217299 | XKR7 | 0.2237 |
| ENSP00000190983 | WISP2 | 0.2237 |
| ENSP00000331087 | TSSC4 | 0.2237 |
| ENSP00000301599 | TMEM88 | 0.2237 |
| ENSP00000340668 | TMEM248 | 0.2237 |
| ENSP00000292894 | THAP8 | 0.2237 |
| ENSP00000225777 | SYNGR2 | 0.2237 |
| ENSP00000351075 | SUSD2 | 0.2237 |
| ENSP00000310814 | STARD6 | 0.2237 |
| ENSP00000009041 | STARD3NL | 0.2237 |
| ENSP00000369080 | SPEF1 | 0.2237 |
| ENSP00000316012 | SPAG11A | 0.2237 |
| ENSP00000346990 | SCARA5 | 0.2237 |
| ENSP00000364956 | PLEKHM2 | 0.2237 |
| ENSP00000330278 | PLEKHH1 | 0.2237 |
| ENSP00000406909 | PHOSPHO1 | 0.2237 |
| ENSP00000429808 | PCDHA3 | 0.2237 |
| ENSP00000355082 | PCDH18 | 0.2237 |
| ENSP00000359382 | PASD1 | 0.2237 |
| ENSP00000319511 | OR4K5 | 0.2237 |
| ENSP00000307852 | NUDT18 | 0.2237 |
| ENSP00000286733 | NAAA | 0.2237 |
| ENSP00000318086 | MAP7D3 | 0.2237 |
| ENSP00000337950 | LYNX1 | 0.2237 |
| ENSP00000270452 | LILRB4 | 0.2237 |
| ENSP00000381542 | KRTAP5-10 | 0.2237 |
| ENSP00000330746 | KRTAP20-2 | 0.2237 |
| ENSP00000334985 | KRTAP19-5 | 0.2237 |
| ENSP00000246646 | KRT38 | 0.2237 |
| ENSP00000167588 | KRT20 | 0.2237 |
| ENSP00000305200 | IL1RAPL1 | 0.2237 |
| ENSP00000238609 | IFI27L2 | 0.2237 |
| ENSP00000371929 | HTR3D | 0.2237 |
| ENSP00000355228 | HMGN2 | 0.2237 |

| | | |
|---|---|---|
| ENSP00000305839 | GPRIN1 | 0.2237 |
| ENSP00000319250 | GPR62 | 0.2237 |
| ENSP00000281703 | GLT1D1 | 0.2237 |
| ENSP00000323075 | GAPT | 0.2237 |
| ENSP00000267426 | FITM1 | 0.2237 |
| ENSP00000259988 | FGFBP1 | 0.2237 |
| ENSP00000355614 | FAM89A | 0.2237 |
| ENSP00000351138 | FAM73B | 0.2237 |
| ENSP00000319336 | FAM216B | 0.2237 |
| ENSP00000254624 | EFR3A | 0.2237 |
| ENSP00000361276 | DYDC1 | 0.2237 |
| ENSP00000307860 | DIRC1 | 0.2237 |
| ENSP00000392568 | DEFB130 | 0.2237 |
| ENSP00000246105 | DEFB129 | 0.2237 |
| ENSP00000226951 | CLNK | 0.2237 |
| ENSP00000298527 | CLEC1B | 0.2237 |
| ENSP00000263284 | CCDC61 | 0.2237 |
| ENSP00000313601 | CC2D1A | 0.2237 |
| ENSP00000374183 | CAMSAP1 | 0.2237 |
| ENSP00000355306 | C1orf174 | 0.2237 |
| ENSP00000369739 | BPHL | 0.2237 |
| ENSP00000453153 | BLID | 0.2237 |
| ENSP00000442793 | BEAN1 | 0.2237 |
| ENSP00000249044 | APOL5 | 0.2237 |
| ENSP00000256737 | ANO3 | 0.2237 |
| ENSP00000310459 | AKIP1 | 0.2237 |
| ENSP00000257359 | ADAMTS8 | 0.2237 |
| ENSP00000455585 | ZNF853 | 0.2211 |
| ENSP00000337122 | ZNF354A | 0.2211 |
| ENSP00000337473 | ZNF324B | 0.2211 |
| ENSP00000336719 | ZNF226 | 0.2211 |
| ENSP00000263805 | ZNF106 | 0.2211 |
| ENSP00000372361 | ZMYM5 | 0.2211 |
| ENSP00000362360 | ZHX3 | 0.2211 |
| ENSP00000428966 | ZFP41 | 0.2211 |
| ENSP00000264031 | UPK2 | 0.2211 |
| ENSP00000279907 | UHRF1BP1L | 0.2211 |
| ENSP00000357575 | TTC40 | 0.2211 |
| ENSP00000370419 | TTC38 | 0.2211 |
| ENSP00000353785 | TPPP | 0.2211 |
| ENSP00000293826 | TNFSF12-TNFSF13 | 0.2211 |
| ENSP00000361311 | TMEM53 | 0.2211 |
| ENSP00000422431 | TMEM158 | 0.2211 |
| ENSP00000273695 | TM4SF19 | 0.2211 |
| ENSP00000273590 | TCTA | 0.2211 |
| ENSP00000217428 | SPINT3 | 0.2211 |
| ENSP00000295958 | SMIM14 | 0.2211 |
| ENSP00000266077 | SLC2A4RG | 0.2211 |
| ENSP00000344801 | SLC24A2 | 0.2211 |
| ENSP00000383939 | SETD5 | 0.2211 |
| ENSP00000436496 | RP11-481A20.11 | 0.2211 |
| ENSP00000358703 | RIPPLY2 | 0.2211 |
| ENSP00000262753 | POF1B | 0.2211 |

| | | |
|---|---|---|
| ENSP00000258526 | PLXNC1 | 0.2211 |
| ENSP00000362778 | PI16 | 0.2211 |
| ENSP00000231137 | PCDHB7 | 0.2211 |
| ENSP00000194155 | PCDHB2 | 0.2211 |
| ENSP00000342008 | OR2T27 | 0.2211 |
| ENSP00000329448 | NUTM1 | 0.2211 |
| ENSP00000222674 | NPVF | 0.2211 |
| ENSP00000295440 | NPPC | 0.2211 |
| ENSP00000302037 | MFF | 0.2211 |
| ENSP00000357829 | METTL10 | 0.2211 |
| ENSP00000357772 | LCE2C | 0.2211 |
| ENSP00000335660 | KRTAP19-2 | 0.2211 |
| ENSP00000352941 | KCTD18 | 0.2211 |
| ENSP00000361173 | KCNE1L | 0.2211 |
| ENSP00000327786 | IZUMO1 | 0.2211 |
| ENSP00000380041 | INCA1 | 0.2211 |
| ENSP00000301790 | HRASLS5 | 0.2211 |
| ENSP00000255695 | HRASLS2 | 0.2211 |
| ENSP00000291481 | HAPLN4 | 0.2211 |
| ENSP00000427690 | GYPB | 0.2211 |
| ENSP00000398454 | GOLGA8N | 0.2211 |
| ENSP00000313933 | GLTSCR1L | 0.2211 |
| ENSP00000399938 | FLYWCH1 | 0.2211 |
| ENSP00000351740 | FAM114A1 | 0.2211 |
| ENSP00000463517 | EGFL8 | 0.2211 |
| ENSP00000354643 | DIO1 | 0.2211 |
| ENSP00000254337 | DCAF15 | 0.2211 |
| ENSP00000372459 | CTIF | 0.2211 |
| ENSP00000470240 | CTD-2192J16.22 | 0.2211 |
| ENSP00000332602 | CTAG1B | 0.2211 |
| ENSP00000371193 | CPPED1 | 0.2211 |
| ENSP00000261883 | CILP | 0.2211 |
| ENSP00000249014 | CDC42EP1 | 0.2211 |
| ENSP00000295226 | CCDC140 | 0.2211 |
| ENSP00000376823 | C7orf49 | 0.2211 |
| ENSP00000386184 | C5orf54 | 0.2211 |
| ENSP00000352704 | C20orf112 | 0.2211 |
| ENSP00000307765 | C17orf62 | 0.2211 |
| ENSP00000306320 | C14orf142 | 0.2211 |
| ENSP00000331581 | C11orf87 | 0.2211 |
| ENSP00000414390 | C11orf70 | 0.2211 |
| ENSP00000262126 | ANKRD12 | 0.2211 |
| ENSP00000434258 | ZNF623 | 0.2211 |
| ENSP00000388311 | ZNF845 | 0.2184 |
| ENSP00000252799 | ZNF747 | 0.2184 |
| ENSP00000329264 | ZNF662 | 0.2184 |
| ENSP00000377504 | ZNF414 | 0.2184 |
| ENSP00000420418 | ZNF398 | 0.2184 |
| ENSP00000354680 | ZNF266 | 0.2184 |
| ENSP00000270014 | ZNF155 | 0.2184 |
| ENSP00000398798 | ZNF142 | 0.2184 |
| ENSP00000361411 | ZCCHC24 | 0.2184 |
| ENSP00000347997 | UBQLN3 | 0.2184 |

| | | |
|---|---|---|
| ENSP00000357906 | TNFAIP8L2 | 0.2184 |
| ENSP00000325561 | TMC8 | 0.2184 |
| ENSP00000342322 | TM7SF3 | 0.2184 |
| ENSP00000217320 | TLDC2 | 0.2184 |
| ENSP00000421489 | TIGD5 | 0.2184 |
| ENSP00000176763 | STK10 | 0.2184 |
| ENSP00000241274 | SLITRK3 | 0.2184 |
| ENSP00000345295 | SH2D4B | 0.2184 |
| ENSP00000376421 | SDK2 | 0.2184 |
| ENSP00000354080 | SCUBE1 | 0.2184 |
| ENSP00000303070 | SCGB1D1 | 0.2184 |
| ENSP00000301995 | SCAND1 | 0.2184 |
| ENSP00000438042 | SAMD8 | 0.2184 |
| ENSP00000311712 | RTP1 | 0.2184 |
| ENSP00000455755 | RP11-12J10.3 | 0.2184 |
| ENSP00000364246 | PLA2G2D | 0.2184 |
| ENSP00000252087 | PCDHGC5 | 0.2184 |
| ENSP00000355419 | PCDH11Y | 0.2184 |
| ENSP00000307164 | PATE1 | 0.2184 |
| ENSP00000362682 | OLFML2A | 0.2184 |
| ENSP00000297130 | MYOZ3 | 0.2184 |
| ENSP00000271139 | MOB3C | 0.2184 |
| ENSP00000410818 | MAD2L1BP | 0.2184 |
| ENSP00000470948 | LRLE1 | 0.2184 |
| ENSP00000357759 | LCE1E | 0.2184 |
| ENSP00000381198 | KIAA1468 | 0.2184 |
| ENSP00000263468 | KIAA1377 | 0.2184 |
| ENSP00000341490 | ISM2 | 0.2184 |
| ENSP00000302676 | GPR22 | 0.2184 |
| ENSP00000271883 | GON4L | 0.2184 |
| ENSP00000323217 | GOLGA8R | 0.2184 |
| ENSP00000452706 | GOLGA6L9 | 0.2184 |
| ENSP00000391085 | GOLGA6D | 0.2184 |
| ENSP00000419485 | GCSAM | 0.2184 |
| ENSP00000396160 | FBRSL1 | 0.2184 |
| ENSP00000259963 | FAM8A1 | 0.2184 |
| ENSP00000257515 | FAM189A2 | 0.2184 |
| ENSP00000333779 | EVI2B | 0.2184 |
| ENSP00000228865 | CREBL2 | 0.2184 |
| ENSP00000299721 | CPLX4 | 0.2184 |
| ENSP00000341360 | COBLL1 | 0.2184 |
| ENSP00000329158 | CLRN1 | 0.2184 |
| ENSP00000371505 | CLEC6A | 0.2184 |
| ENSP00000341051 | CLEC18B | 0.2184 |
| ENSP00000219197 | CBLN1 | 0.2184 |
| ENSP00000358107 | CA14 | 0.2184 |
| ENSP00000322469 | C3orf38 | 0.2184 |
| ENSP00000338885 | C2CD2L | 0.2184 |
| ENSP00000217195 | C20orf27 | 0.2184 |
| ENSP00000377545 | C1QTNF2 | 0.2184 |
| ENSP00000297290 | BRI3 | 0.2184 |
| ENSP00000456645 | ALG9 | 0.2184 |
| ENSP00000375387 | AL356585.1 | 0.2184 |

| | | |
|---|---|---|
| ENSP00000357565 | ZUFSP | 0.2158 |
| ENSP00000254323 | ZSWIM4 | 0.2158 |
| ENSP00000325038 | ZNF836 | 0.2158 |
| ENSP00000415774 | ZNF732 | 0.2158 |
| ENSP00000342818 | ZNF669 | 0.2158 |
| ENSP00000417696 | ZNF525 | 0.2158 |
| ENSP00000464872 | ZNF519 | 0.2158 |
| ENSP00000262894 | ZNF225 | 0.2158 |
| ENSP00000391742 | ZMYND15 | 0.2158 |
| ENSP00000452959 | ZMYND11 | 0.2158 |
| ENSP00000332750 | ZKSCAN8 | 0.2158 |
| ENSP00000250823 | VCY1B | 0.2158 |
| ENSP00000370479 | VCX3A | 0.2158 |
| ENSP00000290079 | TMEM141 | 0.2158 |
| ENSP00000317000 | TM6SF1 | 0.2158 |
| ENSP00000220420 | TGM5 | 0.2158 |
| ENSP00000272438 | TEX261 | 0.2158 |
| ENSP00000356536 | TEDDM1 | 0.2158 |
| ENSP00000383866 | SZRD1 | 0.2158 |
| ENSP00000240333 | SLC35B1 | 0.2158 |
| ENSP00000227918 | SCGB2A2 | 0.2158 |
| ENSP00000353769 | SCAF1 | 0.2158 |
| ENSP00000369547 | S100G | 0.2158 |
| ENSP00000303206 | REG1B | 0.2158 |
| ENSP00000266022 | RBM6 | 0.2158 |
| ENSP00000326018 | PRX | 0.2158 |
| ENSP00000372649 | PRR23A | 0.2158 |
| ENSP00000308926 | PPM1J | 0.2158 |
| ENSP00000326706 | PLEKHO2 | 0.2158 |
| ENSP00000346227 | PKIB | 0.2158 |
| ENSP00000305926 | OTUD7A | 0.2158 |
| ENSP00000302639 | OR5AR1 | 0.2158 |
| ENSP00000259357 | OR1J1 | 0.2158 |
| ENSP00000397900 | MRO | 0.2158 |
| ENSP00000277526 | LCN9 | 0.2158 |
| ENSP00000375107 | KRTAP19-6 | 0.2158 |
| ENSP00000378292 | KRT80 | 0.2158 |
| ENSP00000337850 | KPTN | 0.2158 |
| ENSP00000358956 | IRAK1BP1 | 0.2158 |
| ENSP00000261796 | IL17B | 0.2158 |
| ENSP00000353695 | GSTCD | 0.2158 |
| ENSP00000365624 | GRIPAP1 | 0.2158 |
| ENSP00000260843 | GPR87 | 0.2158 |
| ENSP00000423159 | GOLGA8O | 0.2158 |
| ENSP00000300576 | GOLGA6C | 0.2158 |
| ENSP00000319726 | FRMD8 | 0.2158 |
| ENSP00000362684 | FAM167B | 0.2158 |
| ENSP00000354597 | DENND4B | 0.2158 |
| ENSP00000382951 | DEFB130 | 0.2158 |
| ENSP00000215770 | DDTL | 0.2158 |
| ENSP00000262210 | CSPP1 | 0.2158 |
| ENSP00000378464 | CPLX3 | 0.2158 |
| ENSP00000222032 | CNFN | 0.2158 |

| | | |
|---|---|---|
| ENSP00000221804 | CLC | 0.2158 |
| ENSP00000381428 | CGGBP1 | 0.2158 |
| ENSP00000310031 | CDRT4 | 0.2158 |
| ENSP00000339390 | CDH26 | 0.2158 |
| ENSP00000420298 | CD200 | 0.2158 |
| ENSP00000285871 | CCDC146 | 0.2158 |
| ENSP00000421871 | C5orf20 | 0.2158 |
| ENSP00000351642 | BCAS4 | 0.2158 |
| ENSP00000356094 | AVPR1B | 0.2158 |
| ENSP00000368237 | ATL2 | 0.2158 |
| ENSP00000264992 | ASTE1 | 0.2158 |
| ENSP00000392678 | AKIRIN1 | 0.2158 |
| ENSP00000470441 | AC012313.1 | 0.2158 |
| ENSP00000284322 | ABI3BP | 0.2158 |
| ENSP00000342974 | ZNF791 | 0.2132 |
| ENSP00000355459 | ZNF670 | 0.2132 |
| ENSP00000292841 | ZNF585A | 0.2132 |
| ENSP00000339823 | ZNF546 | 0.2132 |
| ENSP00000465578 | ZNF529 | 0.2132 |
| ENSP00000302310 | ZNF396 | 0.2132 |
| ENSP00000466519 | ZNF229 | 0.2132 |
| ENSP00000248211 | ZNF10 | 0.2132 |
| ENSP00000354453 | ZFP2 | 0.2132 |
| ENSP00000433457 | ZBTB44 | 0.2132 |
| ENSP00000411004 | XG | 0.2132 |
| ENSP00000320532 | WFDC9 | 0.2132 |
| ENSP00000406144 | TTC39A | 0.2132 |
| ENSP00000358529 | TSPAN2 | 0.2132 |
| ENSP00000299413 | TRIM44 | 0.2132 |
| ENSP00000395538 | TNRC18 | 0.2132 |
| ENSP00000393161 | TMEM151B | 0.2132 |
| ENSP00000454261 | TMEM114 | 0.2132 |
| ENSP00000307142 | TEX37 | 0.2132 |
| ENSP00000409016 | TBRG1 | 0.2132 |
| ENSP00000327509 | STAC2 | 0.2132 |
| ENSP00000337991 | SPTY2D1 | 0.2132 |
| ENSP00000311291 | SLCO2A1 | 0.2132 |
| ENSP00000320688 | SLC25A36 | 0.2132 |
| ENSP00000336801 | SLC25A26 | 0.2132 |
| ENSP00000358742 | SFR1 | 0.2132 |
| ENSP00000381081 | RNASE4 | 0.2132 |
| ENSP00000355969 | RD3 | 0.2132 |
| ENSP00000295927 | PTX3 | 0.2132 |
| ENSP00000365908 | PRUNE2 | 0.2132 |
| ENSP00000301015 | PIEZO1 | 0.2132 |
| ENSP00000204549 | PDCD7 | 0.2132 |
| ENSP00000381594 | PCDHGB7 | 0.2132 |
| ENSP00000239446 | PCDHB10 | 0.2132 |
| ENSP00000231484 | PCDH12 | 0.2132 |
| ENSP00000296358 | OTOP1 | 0.2132 |
| ENSP00000300179 | NYAP1 | 0.2132 |
| ENSP00000334872 | MT1F | 0.2132 |
| ENSP00000432845 | MEX3A | 0.2132 |

| | | |
|---|---|---|
| ENSP00000314560 | MAP6D1 | 0.2132 |
| ENSP00000267838 | LYSMD2 | 0.2132 |
| ENSP00000361048 | LURAP1 | 0.2132 |
| ENSP00000333887 | KRTAP22-1 | 0.2132 |
| ENSP00000399475 | JMJD8 | 0.2132 |
| ENSP00000324570 | GYLTL1B | 0.2132 |
| ENSP00000315106 | GPR137C | 0.2132 |
| ENSP00000457904 | GOLGA8Q | 0.2132 |
| ENSP00000388606 | GOLGA6L10 | 0.2132 |
| ENSP00000370439 | FLJ27365 | 0.2132 |
| ENSP00000381982 | FER1L6 | 0.2132 |
| ENSP00000342023 | FBXO38 | 0.2132 |
| ENSP00000379294 | FAM172A | 0.2132 |
| ENSP00000462333 | ENGASE | 0.2132 |
| ENSP00000271764 | EIF2D | 0.2132 |
| ENSP00000435010 | DEFB134 | 0.2132 |
| ENSP00000326309 | DEFB124 | 0.2132 |
| ENSP00000335281 | DEFB105B | 0.2132 |
| ENSP00000280083 | CTAGE5 | 0.2132 |
| ENSP00000331766 | CLECL1 | 0.2132 |
| ENSP00000329318 | CECR6 | 0.2132 |
| ENSP00000349773 | CDC42SE1 | 0.2132 |
| ENSP00000284481 | C8orf12 | 0.2132 |
| ENSP00000232519 | C3orf14 | 0.2132 |
| ENSP00000359115 | C20orf195 | 0.2132 |
| ENSP00000317905 | C17orf96 | 0.2132 |
| ENSP00000239125 | C10orf95 | 0.2132 |
| ENSP00000366477 | C10orf112 | 0.2132 |
| ENSP00000170150 | BPIFB2 | 0.2132 |
| ENSP00000358038 | BEND3 | 0.2132 |
| ENSP00000348799 | ATF7IP2 | 0.2132 |
| ENSP00000385741 | APOBEC3H | 0.2132 |
| ENSP00000258888 | ALPK3 | 0.2132 |
| ENSP00000382091 | ALKBH5 | 0.2132 |
| ENSP00000451476 | AKAP2 | 0.2132 |
| ENSP00000469799 | AC091948.1 | 0.2132 |
| ENSP00000323348 | ZNF645 | 0.2105 |
| ENSP00000334685 | ZNF568 | 0.2105 |
| ENSP00000361602 | ZNF503 | 0.2105 |
| ENSP00000302770 | ZNF296 | 0.2105 |
| ENSP00000409318 | ZNF230 | 0.2105 |
| ENSP00000362435 | ZMYM6NB | 0.2105 |
| ENSP00000216602 | ZFYVE21 | 0.2105 |
| ENSP00000378503 | ZBTB9 | 0.2105 |
| ENSP00000434142 | TPD52L1 | 0.2105 |
| ENSP00000338165 | TMEM39B | 0.2105 |
| ENSP00000343507 | TMEM198 | 0.2105 |
| ENSP00000270560 | TM4SF5 | 0.2105 |
| ENSP00000295924 | TIPARP | 0.2105 |
| ENSP00000462963 | TEFM | 0.2105 |
| ENSP00000332359 | TCEAL2 | 0.2105 |
| ENSP00000335247 | STARD10 | 0.2105 |
| ENSP00000367494 | SPIRE2 | 0.2105 |

| | | |
|---|---|---|
| ENSP00000366390 | SPDYC | 0.2105 |
| ENSP00000362574 | S100PBP | 0.2105 |
| ENSP00000363270 | RUFY4 | 0.2105 |
| ENSP00000369320 | RS1 | 0.2105 |
| ENSP00000456566 | RNF151 | 0.2105 |
| ENSP00000355385 | R3HDM4 | 0.2105 |
| ENSP00000428325 | PYDC2 | 0.2105 |
| ENSP00000268281 | PRSS36 | 0.2105 |
| ENSP00000211413 | PRRT1 | 0.2105 |
| ENSP00000376848 | PRIMA1 | 0.2105 |
| ENSP00000345968 | PGLYRP2 | 0.2105 |
| ENSP00000287196 | PARP6 | 0.2105 |
| ENSP00000416829 | OARD1 | 0.2105 |
| ENSP00000439228 | MTRNR2L1 | 0.2105 |
| ENSP00000370761 | LYRM5 | 0.2105 |
| ENSP00000417330 | KRTAP5-7 | 0.2105 |
| ENSP00000335567 | KRTAP19-4 | 0.2105 |
| ENSP00000298622 | JAKMIP3 | 0.2105 |
| ENSP00000302724 | INSL5 | 0.2105 |
| ENSP00000291560 | HSF2BP | 0.2105 |
| ENSP00000352606 | HAPLN3 | 0.2105 |
| ENSP00000338290 | GZF1 | 0.2105 |
| ENSP00000332900 | GPR97 | 0.2105 |
| ENSP00000421586 | GOLGA6L4 | 0.2105 |
| ENSP00000356389 | GINM1 | 0.2105 |
| ENSP00000341782 | GDAP1L1 | 0.2105 |
| ENSP00000298542 | FRMD7 | 0.2105 |
| ENSP00000259698 | FAM65B | 0.2105 |
| ENSP00000254528 | EMILIN2 | 0.2105 |
| ENSP00000360190 | DEFB110 | 0.2105 |
| ENSP00000359571 | CXorf66 | 0.2105 |
| ENSP00000357900 | CUZD1 | 0.2105 |
| ENSP00000309772 | CPED1 | 0.2105 |
| ENSP00000325655 | CHCHD5 | 0.2105 |
| ENSP00000382508 | CDIP1 | 0.2105 |
| ENSP00000303158 | CCDC8 | 0.2105 |
| ENSP00000375087 | C8orf44 | 0.2105 |
| ENSP00000343242 | C7orf29 | 0.2105 |
| ENSP00000371205 | C2orf48 | 0.2105 |
| ENSP00000347712 | C2CD4A | 0.2105 |
| ENSP00000288048 | C1orf158 | 0.2105 |
| ENSP00000316465 | C18orf56 | 0.2105 |
| ENSP00000376307 | C14orf80 | 0.2105 |
| ENSP00000367189 | C11orf83 | 0.2105 |
| ENSP00000368152 | ALKBH6 | 0.2105 |
| ENSP00000359489 | AFF2 | 0.2105 |
| ENSP00000340683 | ZNF823 | 0.2079 |
| ENSP00000316527 | ZNF609 | 0.2079 |
| ENSP00000295208 | ZNF514 | 0.2079 |
| ENSP00000337368 | ZNF224 | 0.2079 |
| ENSP00000361868 | ZMAT1 | 0.2079 |
| ENSP00000338807 | ZBTB49 | 0.2079 |
| ENSP00000297533 | TMUB1 | 0.2079 |

| | | |
|---|---|---|
| ENSP00000389039 | SYT14 | 0.2079 |
| ENSP00000421686 | SPDYE2 | 0.2079 |
| ENSP00000280191 | SPATA4 | 0.2079 |
| ENSP00000340982 | SPAG8 | 0.2079 |
| ENSP00000341963 | RSC1A1 | 0.2079 |
| ENSP00000184183 | ROPN1 | 0.2079 |
| ENSP00000223271 | RARRES2 | 0.2079 |
| ENSP00000257836 | PRRG4 | 0.2079 |
| ENSP00000280800 | PLBD2 | 0.2079 |
| ENSP00000306918 | PCDHGC4 | 0.2079 |
| ENSP00000304234 | PCDHA10 | 0.2079 |
| ENSP00000303822 | OR7G2 | 0.2079 |
| ENSP00000332068 | OR5P3 | 0.2079 |
| ENSP00000320077 | OR4D11 | 0.2079 |
| ENSP00000349930 | OR11G2 | 0.2079 |
| ENSP00000368920 | NHLRC3 | 0.2079 |
| ENSP00000372857 | NEK11 | 0.2079 |
| ENSP00000410910 | MUSTN1 | 0.2079 |
| ENSP00000303398 | MTBP | 0.2079 |
| ENSP00000318352 | MRFAP1 | 0.2079 |
| ENSP00000381494 | KRTAP2-2 | 0.2079 |
| ENSP00000375108 | KRTAP19-1 | 0.2079 |
| ENSP00000440915 | KIAA0391 | 0.2079 |
| ENSP00000424151 | KCTD16 | 0.2079 |
| ENSP00000281772 | KANSL1L | 0.2079 |
| ENSP00000271843 | JTB | 0.2079 |
| ENSP00000296318 | IL17RD | 0.2079 |
| ENSP00000335511 | HTR3E | 0.2079 |
| ENSP00000386076 | GUCD1 | 0.2079 |
| ENSP00000274721 | GFRA3 | 0.2079 |
| ENSP00000221856 | FSD1 | 0.2079 |
| ENSP00000303508 | FRMD3 | 0.2079 |
| ENSP00000359935 | FPGT | 0.2079 |
| ENSP00000344307 | FOCAD | 0.2079 |
| ENSP00000332879 | FLRT2 | 0.2079 |
| ENSP00000342604 | FAM184A | 0.2079 |
| ENSP00000391524 | DENND6B | 0.2079 |
| ENSP00000355760 | DACT2 | 0.2079 |
| ENSP00000367746 | CLLU1 | 0.2079 |
| ENSP00000301407 | CGB1 | 0.2079 |
| ENSP00000301488 | CDK2AP2 | 0.2079 |
| ENSP00000329482 | CCSER1 | 0.2079 |
| ENSP00000304065 | C2orf27B | 0.2079 |
| ENSP00000295297 | C1QTNF7 | 0.2079 |
| ENSP00000262498 | C16orf80 | 0.2079 |
| ENSP00000355022 | C16orf59 | 0.2079 |
| ENSP00000343686 | BTBD8 | 0.2079 |
| ENSP00000359682 | BRS3 | 0.2079 |
| ENSP00000454697 | BLOC1S5-TXNDC5 | 0.2079 |
| ENSP00000345773 | BEND7 | 0.2079 |
| ENSP00000246785 | BCL2L12 | 0.2079 |
| ENSP00000315383 | ASPRV1 | 0.2079 |
| ENSP00000348064 | ART3 | 0.2079 |

| | | |
|---|---|---|
| ENSP00000436682 | ARMS2 | 0.2079 |
| ENSP00000354569 | GNRHR2 | 0.2079 |
| ENSP00000466433 | LINC00493 | 0.2079 |
| ENSP00000444747 | ZNF835 | 0.2053 |
| ENSP00000345333 | ZNF69 | 0.2053 |
| ENSP00000270649 | ZNF614 | 0.2053 |
| ENSP00000337852 | ZNF576 | 0.2053 |
| ENSP00000333776 | ZNF479 | 0.2053 |
| ENSP00000378792 | ZNF169 | 0.2053 |
| ENSP00000318898 | ZNF114 | 0.2053 |
| ENSP00000232974 | ZBTB47 | 0.2053 |
| ENSP00000364677 | ZBTB12 | 0.2053 |
| ENSP00000414721 | WBP1L | 0.2053 |
| ENSP00000216211 | UPK3A | 0.2053 |
| ENSP00000316740 | TTC29 | 0.2053 |
| ENSP00000367227 | TMEM8B | 0.2053 |
| ENSP00000369699 | TMEM27 | 0.2053 |
| ENSP00000390407 | TMEM213 | 0.2053 |
| ENSP00000315387 | TMEM102 | 0.2053 |
| ENSP00000378166 | TMCO6 | 0.2053 |
| ENSP00000288014 | THTPA | 0.2053 |
| ENSP00000011473 | SYPL1 | 0.2053 |
| ENSP00000367794 | SRPX | 0.2053 |
| ENSP00000359529 | SPANXN2 | 0.2053 |
| ENSP00000359221 | RWDD3 | 0.2053 |
| ENSP00000452844 | RP13-996F3.4 | 0.2053 |
| ENSP00000389709 | RNF212 | 0.2053 |
| ENSP00000333358 | RNASE10 | 0.2053 |
| ENSP00000256585 | REG4 | 0.2053 |
| ENSP00000409315 | RBM18 | 0.2053 |
| ENSP00000286380 | RAET1L | 0.2053 |
| ENSP00000358120 | PLEKHO1 | 0.2053 |
| ENSP00000273371 | PLA1A | 0.2053 |
| ENSP00000309515 | PHYHD1 | 0.2053 |
| ENSP00000352672 | PGLYRP4 | 0.2053 |
| ENSP00000231173 | PCDHB15 | 0.2053 |
| ENSP00000420840 | PCDHA1 | 0.2053 |
| ENSP00000261592 | NOL4 | 0.2053 |
| ENSP00000062104 | NNAT | 0.2053 |
| ENSP00000246190 | NECAB3 | 0.2053 |
| ENSP00000345789 | MUM1 | 0.2053 |
| ENSP00000296468 | MFSD8 | 0.2053 |
| ENSP00000293745 | KRT72 | 0.2053 |
| ENSP00000438244 | KLRF2 | 0.2053 |
| ENSP00000320794 | KIAA2018 | 0.2053 |
| ENSP00000301659 | GSDMA | 0.2053 |
| ENSP00000441032 | GRAMD1A | 0.2053 |
| ENSP00000396441 | GPATCH4 | 0.2053 |
| ENSP00000353142 | GAL3ST4 | 0.2053 |
| ENSP00000322926 | FARP1 | 0.2053 |
| ENSP00000302578 | FAM84B | 0.2053 |
| ENSP00000366901 | FAM216A | 0.2053 |
| ENSP00000261844 | FAM214A | 0.2053 |

| | | |
|---|---|---|
| ENSP00000354958 | FAM189B | 0.2053 |
| ENSP00000368876 | FAM179A | 0.2053 |
| ENSP00000293443 | FAM171A2 | 0.2053 |
| ENSP00000375268 | FAM127C | 0.2053 |
| ENSP00000342805 | FAIM | 0.2053 |
| ENSP00000409382 | EFCAB4B | 0.2053 |
| ENSP00000364943 | DDAH2 | 0.2053 |
| ENSP00000301408 | CGB5 | 0.2053 |
| ENSP00000353717 | CCDC144A | 0.2053 |
| ENSP00000260743 | CALHM2 | 0.2053 |
| ENSP00000329926 | CALHM1 | 0.2053 |
| ENSP00000302260 | C8orf46 | 0.2053 |
| ENSP00000340761 | BRI3BP | 0.2053 |
| ENSP00000303486 | BAHCC1 | 0.2053 |
| ENSP00000327832 | ATAT1 | 0.2053 |
| ENSP00000245543 | ARMC7 | 0.2053 |
| ENSP00000353114 | AHNAK2 | 0.2053 |
| ENSP00000349496 | ADPRH | 0.2053 |
| ENSP00000306241 | ENSG00000229450 | 0.2053 |
| ENSP00000302502 | ZSCAN32 | 0.2026 |
| ENSP00000373274 | ZNF860 | 0.2026 |
| ENSP00000454241 | ZNF747 | 0.2026 |
| ENSP00000333802 | ZNF599 | 0.2026 |
| ENSP00000310033 | ZNF596 | 0.2026 |
| ENSP00000393835 | ZNF506 | 0.2026 |
| ENSP00000244576 | ZNF391 | 0.2026 |
| ENSP00000338927 | ZNF385A | 0.2026 |
| ENSP00000292928 | ZNF382 | 0.2026 |
| ENSP00000351042 | ZNF100 | 0.2026 |
| ENSP00000399664 | ZFP69B | 0.2026 |
| ENSP00000219281 | USB1 | 0.2026 |
| ENSP00000359698 | UBTD1 | 0.2026 |
| ENSP00000269383 | TRIM65 | 0.2026 |
| ENSP00000343398 | TMPPE | 0.2026 |
| ENSP00000260403 | TMEM62 | 0.2026 |
| ENSP00000341539 | TCAIM | 0.2026 |
| ENSP00000418193 | SPRR2F | 0.2026 |
| ENSP00000363001 | SMPDL3B | 0.2026 |
| ENSP00000359521 | SLITRK2 | 0.2026 |
| ENSP00000361681 | SLC25A53 | 0.2026 |
| ENSP00000309751 | SLC16A13 | 0.2026 |
| ENSP00000269389 | SECTM1 | 0.2026 |
| ENSP00000272732 | SCRN3 | 0.2026 |
| ENSP00000358739 | RWDD2A | 0.2026 |
| ENSP00000252655 | RSPH3 | 0.2026 |
| ENSP00000419786 | RGAG1 | 0.2026 |
| ENSP00000299638 | POLR2M | 0.2026 |
| ENSP00000389913 | PLEKHM1 | 0.2026 |
| ENSP00000381636 | PCDHA11 | 0.2026 |
| ENSP00000416193 | P2RX6 | 0.2026 |
| ENSP00000225328 | P2RX5 | 0.2026 |
| ENSP00000262637 | OVOL3 | 0.2026 |
| ENSP00000298642 | OR4K2 | 0.2026 |

| | | |
|---|---|---|
| ENSP00000321338 | OR4C5 | 0.2026 |
| ENSP00000347767 | NEK5 | 0.2026 |
| ENSP00000380603 | NCKAP5 | 0.2026 |
| ENSP00000241527 | MTERFD2 | 0.2026 |
| ENSP00000314042 | MRGPRX4 | 0.2026 |
| ENSP00000357904 | LYSMD1 | 0.2026 |
| ENSP00000379951 | LEPREL2 | 0.2026 |
| ENSP00000344420 | KRTAP1-3 | 0.2026 |
| ENSP00000369438 | KRTAP10-10 | 0.2026 |
| ENSP00000251646 | KRT33B | 0.2026 |
| ENSP00000455951 | KLRK1 | 0.2026 |
| ENSP00000336940 | KIAA1958 | 0.2026 |
| ENSP00000283303 | GPR115 | 0.2026 |
| ENSP00000290823 | GPR114 | 0.2026 |
| ENSP00000356737 | GORAB | 0.2026 |
| ENSP00000428518 | GFRA2 | 0.2026 |
| ENSP00000356432 | FAM5C | 0.2026 |
| ENSP00000363753 | FAM206A | 0.2026 |
| ENSP00000280987 | FAM177A1 | 0.2026 |
| ENSP00000410768 | FAM135A | 0.2026 |
| ENSP00000240364 | FAM117A | 0.2026 |
| ENSP00000333380 | EGFL8 | 0.2026 |
| ENSP00000375416 | DKFZP666M039 | 0.2026 |
| ENSP00000371813 | DEFB132 | 0.2026 |
| ENSP00000359759 | CT45A5 | 0.2026 |
| ENSP00000306776 | CCDC110 | 0.2026 |
| ENSP00000312770 | C2orf69 | 0.2026 |
| ENSP00000376337 | BTBD6 | 0.2026 |
| ENSP00000228936 | ART4 | 0.2026 |
| ENSP00000347433 | APCDD1 | 0.2026 |
| ENSP00000222033 | ZNRF4 | 0.2000 |
| ENSP00000335091 | ZNRF1 | 0.2000 |
| ENSP00000329638 | ZNF804B | 0.2000 |
| ENSP00000323945 | ZNF771 | 0.2000 |
| ENSP00000301042 | ZNF641 | 0.2000 |
| ENSP00000333223 | ZNF470 | 0.2000 |
| ENSP00000308578 | ZNF431 | 0.2000 |
| ENSP00000196489 | ZNF416 | 0.2000 |
| ENSP00000221722 | ZIM2 | 0.2000 |
| ENSP00000327427 | ZFP41 | 0.2000 |
| ENSP00000381109 | ZCWPW1 | 0.2000 |
| ENSP00000316794 | ZCCHC5 | 0.2000 |
| ENSP00000289407 | TSPAN33 | 0.2000 |
| ENSP00000254051 | TNS4 | 0.2000 |
| ENSP00000456881 | TMX2-CTNND1 | 0.2000 |
| ENSP00000162044 | TMEM161A | 0.2000 |
| ENSP00000406482 | THSD7A | 0.2000 |
| ENSP00000054650 | THAP3 | 0.2000 |
| ENSP00000261778 | TANGO6 | 0.2000 |
| ENSP00000381468 | SPINK13 | 0.2000 |
| ENSP00000366690 | SPATC1 | 0.2000 |
| ENSP00000383143 | SLITRK6 | 0.2000 |
| ENSP00000333638 | SLC52A2 | 0.2000 |

| | | |
|---|---|---|
| ENSP00000206544 | SLC22A17 | 0.2000 |
| ENSP00000414750 | SKOR2 | 0.2000 |
| ENSP00000440273 | SH2B2 | 0.2000 |
| ENSP00000272433 | SFXN5 | 0.2000 |
| ENSP00000355979 | SERTAD4 | 0.2000 |
| ENSP00000418693 | RWDD2B | 0.2000 |
| ENSP00000298690 | RNASE7 | 0.2000 |
| ENSP00000344413 | PSG5 | 0.2000 |
| ENSP00000371307 | PPAPDC2 | 0.2000 |
| ENSP00000194152 | PCDHB4 | 0.2000 |
| ENSP00000330049 | OR4Q3 | 0.2000 |
| ENSP00000386167 | OR2A25 | 0.2000 |
| ENSP00000303862 | OR10A2 | 0.2000 |
| ENSP00000307549 | NPTX1 | 0.2000 |
| ENSP00000359340 | NKAIN4 | 0.2000 |
| ENSP00000337998 | NCKAP5L | 0.2000 |
| ENSP00000267853 | MYZAP | 0.2000 |
| ENSP00000356214 | MYCT1 | 0.2000 |
| ENSP00000337222 | MYADM | 0.2000 |
| ENSP00000262102 | MTUS1 | 0.2000 |
| ENSP00000330587 | MT1H | 0.2000 |
| ENSP00000016913 | MS4A12 | 0.2000 |
| ENSP00000352513 | MPZL1 | 0.2000 |
| ENSP00000260453 | MNS1 | 0.2000 |
| ENSP00000292524 | LRRC14 | 0.2000 |
| ENSP00000259006 | LIMD2 | 0.2000 |
| ENSP00000351047 | ICA1L | 0.2000 |
| ENSP00000311502 | HEG1 | 0.2000 |
| ENSP00000058691 | HEBP2 | 0.2000 |
| ENSP00000297977 | HDX | 0.2000 |
| ENSP00000308080 | GTF2IRD2B | 0.2000 |
| ENSP00000014914 | GPRC5A | 0.2000 |
| ENSP00000263733 | FAM20B | 0.2000 |
| ENSP00000340474 | EXD3 | 0.2000 |
| ENSP00000472922 | DKFZP547L112 | 0.2000 |
| ENSP00000404151 | CEP170B | 0.2000 |
| ENSP00000381553 | CCDC11 | 0.2000 |
| ENSP00000425786 | C4orf22 | 0.2000 |
| ENSP00000300006 | C16orf45 | 0.2000 |
| ENSP00000403987 | C15orf40 | 0.2000 |
| ENSP00000350314 | BCO2 | 0.2000 |
| ENSP00000286353 | ACPL2 | 0.2000 |
| ENSP00000448403 | ENSG00000257755 | 0.2000 |
| ENSP00000375593 | ZSCAN5A | 0.1974 |
| ENSP00000385099 | ZNF792 | 0.1974 |
| ENSP00000461425 | ZNF66 | 0.1974 |
| ENSP00000344791 | ZNF600 | 0.1974 |
| ENSP00000332861 | ZNF530 | 0.1974 |
| ENSP00000379054 | ZNF488 | 0.1974 |
| ENSP00000303889 | ZNF30 | 0.1974 |
| ENSP00000288177 | ZNF19 | 0.1974 |
| ENSP00000366509 | ZKSCAN4 | 0.1974 |
| ENSP00000400836 | ZBTB8B | 0.1974 |

| | | |
|---|---|---|
| ENSP00000249330 | VGF | 0.1974 |
| ENSP00000354919 | VEPH1 | 0.1974 |
| ENSP00000240587 | TSHZ3 | 0.1974 |
| ENSP00000378050 | TRPT1 | 0.1974 |
| ENSP00000309532 | TP53I11 | 0.1974 |
| ENSP00000238936 | TMEM180 | 0.1974 |
| ENSP00000296595 | TMEM161B | 0.1974 |
| ENSP00000357807 | THEM5 | 0.1974 |
| ENSP00000310796 | THAP2 | 0.1974 |
| ENSP00000375093 | TAS2R31 | 0.1974 |
| ENSP00000331532 | SPATA32 | 0.1974 |
| ENSP00000335292 | SLC51B | 0.1974 |
| ENSP00000300650 | RNF214 | 0.1974 |
| ENSP00000330269 | RESP18 | 0.1974 |
| ENSP00000259351 | RALGPS1 | 0.1974 |
| ENSP00000369636 | PXDC1 | 0.1974 |
| ENSP00000246794 | PRRG2 | 0.1974 |
| ENSP00000265627 | PON3 | 0.1974 |
| ENSP00000358316 | PLEKHS1 | 0.1974 |
| ENSP00000239449 | PCDHB14 | 0.1974 |
| ENSP00000156084 | OTUD5 | 0.1974 |
| ENSP00000285600 | OR4K1 | 0.1974 |
| ENSP00000343062 | OR2T2 | 0.1974 |
| ENSP00000386138 | OR1C1 | 0.1974 |
| ENSP00000319071 | OR11H6 | 0.1974 |
| ENSP00000318997 | OR11H4 | 0.1974 |
| ENSP00000371994 | NYNRIN | 0.1974 |
| ENSP00000374237 | NXPE2 | 0.1974 |
| ENSP00000369014 | NDNF | 0.1974 |
| ENSP00000369849 | MEDAG | 0.1974 |
| ENSP00000381150 | MBLAC1 | 0.1974 |
| ENSP00000414712 | MAP7 | 0.1974 |
| ENSP00000352163 | LYPD2 | 0.1974 |
| ENSP00000347649 | LEPREL4 | 0.1974 |
| ENSP00000335503 | KRTAP20-1 | 0.1974 |
| ENSP00000334834 | KRTAP13-4 | 0.1974 |
| ENSP00000330720 | KRTAP11-1 | 0.1974 |
| ENSP00000325525 | KIR3DL2 | 0.1974 |
| ENSP00000264257 | IL1RL2 | 0.1974 |
| ENSP00000253680 | HSH2D | 0.1974 |
| ENSP00000248098 | HN1L | 0.1974 |
| ENSP00000354313 | GRAMD4 | 0.1974 |
| ENSP00000399235 | FOLR3 | 0.1974 |
| ENSP00000347714 | FCAR | 0.1974 |
| ENSP00000313159 | FBXO34 | 0.1974 |
| ENSP00000010299 | FAM76A | 0.1974 |
| ENSP00000398617 | FAM71E2 | 0.1974 |
| ENSP00000376071 | FAM117B | 0.1974 |
| ENSP00000368362 | FABP9 | 0.1974 |
| ENSP00000420976 | DLEU7 | 0.1974 |
| ENSP00000297866 | CXorf22 | 0.1974 |
| ENSP00000307741 | CMTM8 | 0.1974 |
| ENSP00000293845 | CCDC42 | 0.1974 |

| | | |
|---|---|---|
| ENSP00000286688 | C8orf37 | 0.1974 |
| ENSP00000331720 | C16orf72 | 0.1974 |
| ENSP00000288757 | C12orf43 | 0.1974 |
| ENSP00000376799 | C11orf65 | 0.1974 |
| ENSP00000292566 | ALKBH4 | 0.1974 |
| ENSP00000361383 | AIF1L | 0.1974 |
| ENSP00000472810 | AC090673.2 | 0.1974 |
| ENSP00000255746 | ZNF679 | 0.1947 |
| ENSP00000421915 | ZNF672 | 0.1947 |
| ENSP00000403975 | ZNF668 | 0.1947 |
| ENSP00000340004 | ZNF564 | 0.1947 |
| ENSP00000410734 | ZNF562 | 0.1947 |
| ENSP00000366384 | ZNF311 | 0.1947 |
| ENSP00000333725 | ZNF26 | 0.1947 |
| ENSP00000291182 | ZNF235 | 0.1947 |
| ENSP00000366273 | ZNF157 | 0.1947 |
| ENSP00000366899 | ZNF133 | 0.1947 |
| ENSP00000374592 | ZC3H12D | 0.1947 |
| ENSP00000294664 | WDR63 | 0.1947 |
| ENSP00000320416 | VPS37D | 0.1947 |
| ENSP00000331305 | TOB2 | 0.1947 |
| ENSP00000322300 | TMTC2 | 0.1947 |
| ENSP00000424040 | TAS2R50 | 0.1947 |
| ENSP00000334042 | TAC4 | 0.1947 |
| ENSP00000414920 | SPATA24 | 0.1947 |
| ENSP00000404438 | SOHLH1 | 0.1947 |
| ENSP00000336627 | SLITRK4 | 0.1947 |
| ENSP00000261187 | SLC16A7 | 0.1947 |
| ENSP00000351086 | RPS6KL1 | 0.1947 |
| ENSP00000373752 | RNF180 | 0.1947 |
| ENSP00000316628 | RNF152 | 0.1947 |
| ENSP00000313385 | RELL1 | 0.1947 |
| ENSP00000377502 | PHLDB2 | 0.1947 |
| ENSP00000438685 | PCDHB17 | 0.1947 |
| ENSP00000253807 | PCDHAC1 | 0.1947 |
| ENSP00000436042 | PCDHA9 | 0.1947 |
| ENSP00000364107 | PAGE2 | 0.1947 |
| ENSP00000303864 | OR8I2 | 0.1947 |
| ENSP00000333724 | OR6X1 | 0.1947 |
| ENSP00000368987 | OR6C75 | 0.1947 |
| ENSP00000308689 | OR10AD1 | 0.1947 |
| ENSP00000437563 | NKG2-E | 0.1947 |
| ENSP00000377411 | NDUFA4L2 | 0.1947 |
| ENSP00000383392 | NCAM2 | 0.1947 |
| ENSP00000369146 | MT1M | 0.1947 |
| ENSP00000379571 | MRGPRX3 | 0.1947 |
| ENSP00000349575 | MOB3A | 0.1947 |
| ENSP00000276654 | LRP12 | 0.1947 |
| ENSP00000375429 | KRTAP3-2 | 0.1947 |
| ENSP00000366976 | KRTAP1-4 | 0.1947 |
| ENSP00000220244 | KIAA1199 | 0.1947 |
| ENSP00000355250 | KIAA0753 | 0.1947 |
| ENSP00000335196 | GLDN | 0.1947 |

| | | |
|---|---|---|
| ENSP00000398277 | GCNT6 | 0.1947 |
| ENSP00000276185 | FRMPD3 | 0.1947 |
| ENSP00000330432 | FAM83F | 0.1947 |
| ENSP00000453973 | EPDR1 | 0.1947 |
| ENSP00000318264 | ELMOD3 | 0.1947 |
| ENSP00000327796 | DCAF4L1 | 0.1947 |
| ENSP00000304327 | COPRS | 0.1947 |
| ENSP00000378577 | CLDN3 | 0.1947 |
| ENSP00000292779 | CCDC116 | 0.1947 |
| ENSP00000276704 | C8orf76 | 0.1947 |
| ENSP00000367359 | C6orf62 | 0.1947 |
| ENSP00000243189 | C1orf63 | 0.1947 |
| ENSP00000280756 | C12orf23 | 0.1947 |
| ENSP00000364643 | BPIFB3 | 0.1947 |
| ENSP00000310622 | APOBEC4 | 0.1947 |
| ENSP00000383520 | AL354822.1 | 0.1947 |
| ENSP00000380293 | ZNF99 | 0.1921 |
| ENSP00000378642 | ZNF785 | 0.1921 |
| ENSP00000394380 | ZNF625 | 0.1921 |
| ENSP00000301073 | ZNF524 | 0.1921 |
| ENSP00000319716 | ZNF101 | 0.1921 |
| ENSP00000272321 | WDPCP | 0.1921 |
| ENSP00000233615 | WBP1 | 0.1921 |
| ENSP00000353998 | VKORC1L1 | 0.1921 |
| ENSP00000232496 | TUSC2 | 0.1921 |
| ENSP00000307640 | TTC36 | 0.1921 |
| ENSP00000418767 | TREML2 | 0.1921 |
| ENSP00000341677 | TPD52L3 | 0.1921 |
| ENSP00000368858 | TMEM14B | 0.1921 |
| ENSP00000340088 | THEG | 0.1921 |
| ENSP00000353542 | SPRR2D | 0.1921 |
| ENSP00000429168 | SLC25A30 | 0.1921 |
| ENSP00000418707 | SLAIN1 | 0.1921 |
| ENSP00000314023 | SIDT2 | 0.1921 |
| ENSP00000446058 | SHD | 0.1921 |
| ENSP00000245105 | SH3TC1 | 0.1921 |
| ENSP00000323191 | SAGE1 | 0.1921 |
| ENSP00000356272 | RMND1 | 0.1921 |
| ENSP00000344299 | PTAR1 | 0.1921 |
| ENSP00000254765 | POPDC3 | 0.1921 |
| ENSP00000222968 | PDAP1 | 0.1921 |
| ENSP00000231130 | PCDHB3 | 0.1921 |
| ENSP00000289269 | PCDHAC2 | 0.1921 |
| ENSP00000289272 | PCDHA13 | 0.1921 |
| ENSP00000281631 | PARP8 | 0.1921 |
| ENSP00000308595 | OR5R1 | 0.1921 |
| ENSP00000334393 | OR4F5 | 0.1921 |
| ENSP00000335575 | OR1J2 | 0.1921 |
| ENSP00000316782 | NBPF3 | 0.1921 |
| ENSP00000371553 | NANOGNB | 0.1921 |
| ENSP00000305766 | MRGPRX1 | 0.1921 |
| ENSP00000300952 | MIDN | 0.1921 |
| ENSP00000375479 | KRTAP10-2 | 0.1921 |

| | | |
|---|---|---|
| ENSP00000316681 | KIAA1731 | 0.1921 |
| ENSP00000315295 | KIAA1549L | 0.1921 |
| ENSP00000318406 | KIAA0319L | 0.1921 |
| ENSP00000259205 | IL36G | 0.1921 |
| ENSP00000301917 | GPR25 | 0.1921 |
| ENSP00000320207 | GOLGA6L1 | 0.1921 |
| ENSP00000293981 | FLYWCH2 | 0.1921 |
| ENSP00000361345 | FAM78A | 0.1921 |
| ENSP00000393667 | FAM126B | 0.1921 |
| ENSP00000300714 | ENTHD2 | 0.1921 |
| ENSP00000262547 | DZANK1 | 0.1921 |
| ENSP00000248879 | DGCR6L | 0.1921 |
| ENSP00000347810 | DEFB107B | 0.1921 |
| ENSP00000363128 | DEC1 | 0.1921 |
| ENSP00000332449 | CRIP1 | 0.1921 |
| ENSP00000338838 | CHID1 | 0.1921 |
| ENSP00000359563 | CDR1 | 0.1921 |
| ENSP00000427108 | CDKN2AIP | 0.1921 |
| ENSP00000333374 | CCDC60 | 0.1921 |
| ENSP00000374260 | CCDC149 | 0.1921 |
| ENSP00000222374 | CADM4 | 0.1921 |
| ENSP00000390153 | C6orf203 | 0.1921 |
| ENSP00000367564 | C19orf54 | 0.1921 |
| ENSP00000466104 | C17orf61-PLSCR3 | 0.1921 |
| ENSP00000285697 | C16orf87 | 0.1921 |
| ENSP00000354227 | C11orf80 | 0.1921 |
| ENSP00000349244 | C10orf137 | 0.1921 |
| ENSP00000309644 | BOD1 | 0.1921 |
| ENSP00000369518 | ATRAID | 0.1921 |
| ENSP00000258417 | ARMC9 | 0.1921 |
| ENSP00000318770 | AQP11 | 0.1921 |
| ENSP00000383692 | APITD1-CORT | 0.1921 |
| ENSP00000350509 | AIG1 | 0.1921 |
| ENSP00000352177 | ADAM29 | 0.1921 |
| ENSP00000432816 | ACRV1 | 0.1921 |
| ENSP00000470969 | AC004076.9 | 0.1921 |
| ENSP00000261880 | AAGAB | 0.1921 |
| ENSP00000404074 | ZSCAN9 | 0.1895 |
| ENSP00000412999 | ZNF805 | 0.1895 |
| ENSP00000400997 | ZNF780A | 0.1895 |
| ENSP00000320050 | ZNF580 | 0.1895 |
| ENSP00000327538 | ZNF534 | 0.1895 |
| ENSP00000329141 | ZNF37A | 0.1895 |
| ENSP00000333775 | XAGE2 | 0.1895 |
| ENSP00000343871 | TMTC4 | 0.1895 |
| ENSP00000257663 | TMEM60 | 0.1895 |
| ENSP00000306564 | TMEM42 | 0.1895 |
| ENSP00000340477 | TMEM179 | 0.1895 |
| ENSP00000361138 | TMEM164 | 0.1895 |
| ENSP00000374274 | SSC5D | 0.1895 |
| ENSP00000285013 | SLFN13 | 0.1895 |
| ENSP00000217254 | SLC52A3 | 0.1895 |
| ENSP00000312942 | SEZ6 | 0.1895 |

| | | |
|---|---|---|
| ENSP00000346892 | SERF1A | 0.1895 |
| ENSP00000290216 | SCRN2 | 0.1895 |
| ENSP00000358902 | SAMD10 | 0.1895 |
| ENSP00000377942 | RSPRY1 | 0.1895 |
| ENSP00000457544 | RP11-77K12.7 | 0.1895 |
| ENSP00000454374 | RP11-468E2.2 | 0.1895 |
| ENSP00000358617 | PHTF1 | 0.1895 |
| ENSP00000231136 | PCDHB6 | 0.1895 |
| ENSP00000354293 | PCDHB16 | 0.1895 |
| ENSP00000434655 | PCDHA8 | 0.1895 |
| ENSP00000363873 | OXLD1 | 0.1895 |
| ENSP00000248073 | OR7C1 | 0.1895 |
| ENSP00000351466 | OR5AC2 | 0.1895 |
| ENSP00000312403 | OR2Y1 | 0.1895 |
| ENSP00000310773 | OR1L1 | 0.1895 |
| ENSP00000299481 | NLRP14 | 0.1895 |
| ENSP00000297142 | NEUROD6 | 0.1895 |
| ENSP00000262384 | N4BP1 | 0.1895 |
| ENSP00000304713 | MSANTD4 | 0.1895 |
| ENSP00000309521 | LIME1 | 0.1895 |
| ENSP00000444350 | LHFPL3 | 0.1895 |
| ENSP00000382590 | KRTAP5-4 | 0.1895 |
| ENSP00000381489 | KRTAP4-1 | 0.1895 |
| ENSP00000328017 | KRBA2 | 0.1895 |
| ENSP00000013222 | INMT | 0.1895 |
| ENSP00000288098 | IL34 | 0.1895 |
| ENSP00000415823 | IGFL1 | 0.1895 |
| ENSP00000334805 | H1FNT | 0.1895 |
| ENSP00000328070 | GLTPD2 | 0.1895 |
| ENSP00000329292 | GKN2 | 0.1895 |
| ENSP00000384593 | GAREML | 0.1895 |
| ENSP00000344579 | FSCB | 0.1895 |
| ENSP00000395848 | FAM90A26 | 0.1895 |
| ENSP00000420954 | FAM156B | 0.1895 |
| ENSP00000353887 | FAM153A | 0.1895 |
| ENSP00000304642 | FAM134B | 0.1895 |
| ENSP00000397679 | ENDOU | 0.1895 |
| ENSP00000378635 | DRP2 | 0.1895 |
| ENSP00000371644 | DEFB136 | 0.1895 |
| ENSP00000296955 | DCBLD1 | 0.1895 |
| ENSP00000228515 | CSRNP2 | 0.1895 |
| ENSP00000359310 | CSAG1 | 0.1895 |
| ENSP00000406327 | COX20 | 0.1895 |
| ENSP00000263655 | CNRIP1 | 0.1895 |
| ENSP00000295304 | CHAC2 | 0.1895 |
| ENSP00000379242 | CDRT1 | 0.1895 |
| ENSP00000311035 | CD200R1 | 0.1895 |
| ENSP00000320251 | C3orf17 | 0.1895 |
| ENSP00000295148 | C2orf44 | 0.1895 |
| ENSP00000309561 | C19orf70 | 0.1895 |
| ENSP00000269221 | C18orf8 | 0.1895 |
| ENSP00000377971 | ANKLE1 | 0.1895 |
| ENSP00000383639 | AC145212.1 | 0.1895 |

| | | |
|---|---|---|
| ENSP00000311181 | SNURFL | 0.1895 |
| ENSP00000351530 | ZXDA | 0.1868 |
| ENSP00000345339 | ZSCAN31 | 0.1868 |
| ENSP00000379689 | ZNF765 | 0.1868 |
| ENSP00000328245 | ZNF71 | 0.1868 |
| ENSP00000380840 | ZNF709 | 0.1868 |
| ENSP00000338770 | ZNF420 | 0.1868 |
| ENSP00000299237 | ZNF319 | 0.1868 |
| ENSP00000298585 | ZMYND19 | 0.1868 |
| ENSP00000384967 | VSTM2A | 0.1868 |
| ENSP00000377381 | UBTD2 | 0.1868 |
| ENSP00000301272 | TMIGD2 | 0.1868 |
| ENSP00000303148 | TMEM37 | 0.1868 |
| ENSP00000323068 | TMEM168 | 0.1868 |
| ENSP00000353093 | TCEAL8 | 0.1868 |
| ENSP00000275200 | TAAR8 | 0.1868 |
| ENSP00000340703 | SPRR2B | 0.1868 |
| ENSP00000351503 | SPATS2L | 0.1868 |
| ENSP00000367965 | SOWAHA | 0.1868 |
| ENSP00000379057 | SMCR7 | 0.1868 |
| ENSP00000406026 | SH2D5 | 0.1868 |
| ENSP00000461269 | SCIMP | 0.1868 |
| ENSP00000334040 | RSBN1L | 0.1868 |
| ENSP00000280606 | PRSS53 | 0.1868 |
| ENSP00000301039 | PROCA1 | 0.1868 |
| ENSP00000436426 | PCDHA7 | 0.1868 |
| ENSP00000328144 | OR7A17 | 0.1868 |
| ENSP00000369728 | OR52J3 | 0.1868 |
| ENSP00000313936 | OR2AE1 | 0.1868 |
| ENSP00000300182 | MS4A6E | 0.1868 |
| ENSP00000452252 | MIA2 | 0.1868 |
| ENSP00000336817 | LUZP2 | 0.1868 |
| ENSP00000157600 | LMCD1 | 0.1868 |
| ENSP00000371606 | KRTAP5-1 | 0.1868 |
| ENSP00000381005 | KRTAP12-3 | 0.1868 |
| ENSP00000383225 | KRTAP10-4 | 0.1868 |
| ENSP00000355319 | KIAA1737 | 0.1868 |
| ENSP00000462172 | KIAA0040 | 0.1868 |
| ENSP00000278071 | ITPRIP | 0.1868 |
| ENSP00000259213 | IL36B | 0.1868 |
| ENSP00000223026 | HYAL4 | 0.1868 |
| ENSP00000436500 | GRAMD1B | 0.1868 |
| ENSP00000362485 | GARNL3 | 0.1868 |
| ENSP00000348489 | FBRS | 0.1868 |
| ENSP00000318182 | FAM156A | 0.1868 |
| ENSP00000365759 | DDR1 | 0.1868 |
| ENSP00000309538 | DAPL1 | 0.1868 |
| ENSP00000349989 | CXorf24 | 0.1868 |
| ENSP00000399356 | CT62 | 0.1868 |
| ENSP00000263401 | COMMD9 | 0.1868 |
| ENSP00000216420 | CGRRF1 | 0.1868 |
| ENSP00000353706 | CDC42SE2 | 0.1868 |
| ENSP00000361392 | CCDC24 | 0.1868 |

| | | |
|---|---|---|
| ENSP00000329360 | CCDC137 | 0.1868 |
| ENSP00000404132 | C8orf59 | 0.1868 |
| ENSP00000416290 | C7orf25 | 0.1868 |
| ENSP00000389014 | C5orf42 | 0.1868 |
| ENSP00000386807 | C4orf48 | 0.1868 |
| ENSP00000352447 | C1orf116 | 0.1868 |
| ENSP00000242784 | C19orf43 | 0.1868 |
| ENSP00000286067 | C10orf12 | 0.1868 |
| ENSP00000313172 | BVES | 0.1868 |
| ENSP00000375248 | BHLHA9 | 0.1868 |
| ENSP00000343674 | ASTL | 0.1868 |
| ENSP00000471625 | AC003005.4 | 0.1868 |
| ENSP00000341179 | AC002472.13 | 0.1868 |
| ENSP00000359802 | ZNF75D | 0.1842 |
| ENSP00000223459 | ZNF688 | 0.1842 |
| ENSP00000312141 | ZNF654 | 0.1842 |
| ENSP00000392410 | ZNF587B | 0.1842 |
| ENSP00000331540 | ZNF570 | 0.1842 |
| ENSP00000375582 | ZNF552 | 0.1842 |
| ENSP00000365407 | ZNF549 | 0.1842 |
| ENSP00000350576 | ZNF441 | 0.1842 |
| ENSP00000327143 | ZNF354B | 0.1842 |
| ENSP00000196482 | ZNF324 | 0.1842 |
| ENSP00000262085 | ZNF282 | 0.1842 |
| ENSP00000401947 | ZNF223 | 0.1842 |
| ENSP00000337081 | ZNF112 | 0.1842 |
| ENSP00000472867 | ZIK1 | 0.1842 |
| ENSP00000343348 | VMAC | 0.1842 |
| ENSP00000396918 | URGCP | 0.1842 |
| ENSP00000256367 | TTC9 | 0.1842 |
| ENSP00000338533 | TTC33 | 0.1842 |
| ENSP00000261177 | TSPAN11 | 0.1842 |
| ENSP00000438125 | TPCN1 | 0.1842 |
| ENSP00000275954 | TMEM47 | 0.1842 |
| ENSP00000303028 | TM4SF20 | 0.1842 |
| ENSP00000299290 | TBATA | 0.1842 |
| ENSP00000332721 | TANGO2 | 0.1842 |
| ENSP00000344831 | SLC25A40 | 0.1842 |
| ENSP00000218197 | SLC25A14 | 0.1842 |
| ENSP00000392353 | PSMG4 | 0.1842 |
| ENSP00000297767 | PRSS37 | 0.1842 |
| ENSP00000357733 | PRR9 | 0.1842 |
| ENSP00000331845 | PRR14L | 0.1842 |
| ENSP00000382485 | PROL1 | 0.1842 |
| ENSP00000310117 | PPP1R14B | 0.1842 |
| ENSP00000319231 | PHLDA2 | 0.1842 |
| ENSP00000265260 | PCNP | 0.1842 |
| ENSP00000239444 | PCDHB8 | 0.1842 |
| ENSP00000359600 | ODF2L | 0.1842 |
| ENSP00000380251 | NUDT19 | 0.1842 |
| ENSP00000350874 | METTL9 | 0.1842 |
| ENSP00000362707 | MANBAL | 0.1842 |
| ENSP00000254072 | KRTAP9-8 | 0.1842 |

| | | |
|---|---|---|
| ENSP00000383216 | KRTAP10-12 | 0.1842 |
| ENSP00000329165 | KRT36 | 0.1842 |
| ENSP00000375608 | KIR3DL1 | 0.1842 |
| ENSP00000302924 | IL17D | 0.1842 |
| ENSP00000350387 | HYAL2 | 0.1842 |
| ENSP00000322617 | HTR3C | 0.1842 |
| ENSP00000229633 | HINT3 | 0.1842 |
| ENSP00000351430 | GYPE | 0.1842 |
| ENSP00000257626 | GSAP | 0.1842 |
| ENSP00000324553 | GPR156 | 0.1842 |
| ENSP00000370057 | FRMPD4 | 0.1842 |
| ENSP00000357086 | FCRL6 | 0.1842 |
| ENSP00000353794 | FBXL22 | 0.1842 |
| ENSP00000400513 | FASTKD1 | 0.1842 |
| ENSP00000312017 | FAM57A | 0.1842 |
| ENSP00000358170 | FAM204A | 0.1842 |
| ENSP00000354891 | FAM163A | 0.1842 |
| ENSP00000417970 | FAM120B | 0.1842 |
| ENSP00000353650 | FABP12 | 0.1842 |
| ENSP00000406027 | EPM2AIP1 | 0.1842 |
| ENSP00000363694 | EPB41L4B | 0.1842 |
| ENSP00000375259 | DKFZP667F0711 | 0.1842 |
| ENSP00000472467 | CXCL17 | 0.1842 |
| ENSP00000366178 | CST9L | 0.1842 |
| ENSP00000357156 | CD5L | 0.1842 |
| ENSP00000462883 | CCDC79 | 0.1842 |
| ENSP00000242819 | CCDC70 | 0.1842 |
| ENSP00000380941 | C21orf67 | 0.1842 |
| ENSP00000454926 | C16orf52 | 0.1842 |
| ENSP00000304676 | ATOH8 | 0.1842 |
| ENSP00000370227 | AREGB | 0.1842 |
| ENSP00000329793 | ZNF85 | 0.1816 |
| ENSP00000341165 | ZNF772 | 0.1816 |
| ENSP00000320627 | ZNF7 | 0.1816 |
| ENSP00000300850 | ZNF646 | 0.1816 |
| ENSP00000242804 | ZNF442 | 0.1816 |
| ENSP00000240731 | ZNF211 | 0.1816 |
| ENSP00000471905 | ZNF17 | 0.1816 |
| ENSP00000297737 | ZMAT4 | 0.1816 |
| ENSP00000354543 | TSC22D2 | 0.1816 |
| ENSP00000368138 | TRIQK | 0.1816 |
| ENSP00000365844 | TRIM39 | 0.1816 |
| ENSP00000379030 | TRIM16L | 0.1816 |
| ENSP00000407685 | TNXB | 0.1816 |
| ENSP00000314116 | TMEM107 | 0.1816 |
| ENSP00000348132 | STK31 | 0.1816 |
| ENSP00000006658 | SPATA20 | 0.1816 |
| ENSP00000265909 | SNX19 | 0.1816 |
| ENSP00000342518 | SLC35F4 | 0.1816 |
| ENSP00000258403 | SLC19A3 | 0.1816 |
| ENSP00000415200 | SIGLEC5 | 0.1816 |
| ENSP00000423660 | SH3TC2 | 0.1816 |
| ENSP00000331258 | SGK196 | 0.1816 |

| | | |
|---|---|---|
| ENSP00000404860 | SCHIP1 | 0.1816 |
| ENSP00000383421 | RBM11 | 0.1816 |
| ENSP00000375717 | PTOV1 | 0.1816 |
| ENSP00000361338 | PPAPDC3 | 0.1816 |
| ENSP00000289672 | PKD1L1 | 0.1816 |
| ENSP00000317144 | PIBF1 | 0.1816 |
| ENSP00000290722 | PGLYRP3 | 0.1816 |
| ENSP00000322801 | OR52N2 | 0.1816 |
| ENSP00000350836 | OR2L13 | 0.1816 |
| ENSP00000386174 | OR2A12 | 0.1816 |
| ENSP00000291890 | NCR1 | 0.1816 |
| ENSP00000325808 | LRRN4CL | 0.1816 |
| ENSP00000334696 | KRTAP19-7 | 0.1816 |
| ENSP00000340083 | KRCC1 | 0.1816 |
| ENSP00000387535 | IRGQ | 0.1816 |
| ENSP00000324882 | IQUB | 0.1816 |
| ENSP00000365529 | GPR158 | 0.1816 |
| ENSP00000318674 | FBXL16 | 0.1816 |
| ENSP00000463817 | FAM27E3 | 0.1816 |
| ENSP00000468308 | FAM20A | 0.1816 |
| ENSP00000328347 | FAM110C | 0.1816 |
| ENSP00000337146 | ENOX2 | 0.1816 |
| ENSP00000425048 | DTWD2 | 0.1816 |
| ENSP00000371825 | DEFB127 | 0.1816 |
| ENSP00000303550 | CYTL1 | 0.1816 |
| ENSP00000371234 | CYS1 | 0.1816 |
| ENSP00000358884 | CYB561D1 | 0.1816 |
| ENSP00000299665 | CLEC4D | 0.1816 |
| ENSP00000250699 | CHRNA10 | 0.1816 |
| ENSP00000356386 | CFHR4 | 0.1816 |
| ENSP00000301200 | CDC42EP5 | 0.1816 |
| ENSP00000253079 | CCDC62 | 0.1816 |
| ENSP00000341368 | C6orf132 | 0.1816 |
| ENSP00000378441 | C6orf1 | 0.1816 |
| ENSP00000258457 | C2orf49 | 0.1816 |
| ENSP00000356912 | C1orf111 | 0.1816 |
| ENSP00000350704 | C1orf109 | 0.1816 |
| ENSP00000301419 | C19orf48 | 0.1816 |
| ENSP00000351937 | C17orf80 | 0.1816 |
| ENSP00000256969 | C12orf39 | 0.1816 |
| ENSP00000294244 | C11orf84 | 0.1816 |
| ENSP00000369372 | BEND2 | 0.1816 |
| ENSP00000470691 | ZSCAN5D | 0.1789 |
| ENSP00000365443 | ZSCAN5C | 0.1789 |
| ENSP00000287461 | ZNF689 | 0.1789 |
| ENSP00000351280 | ZNF429 | 0.1789 |
| ENSP00000331577 | ZNF397 | 0.1789 |
| ENSP00000370055 | UBL3 | 0.1789 |
| ENSP00000357842 | TUFT1 | 0.1789 |
| ENSP00000323645 | TTC39C | 0.1789 |
| ENSP00000352284 | TMEM139 | 0.1789 |
| ENSP00000253435 | TEX101 | 0.1789 |
| ENSP00000283141 | SYCP2L | 0.1789 |

| | | |
|---|---|---|
| ENSP00000206020 | SPAG7 | 0.1789 |
| ENSP00000420863 | SERINC5 | 0.1789 |
| ENSP00000439287 | RIMS4 | 0.1789 |
| ENSP00000350552 | RFX4 | 0.1789 |
| ENSP00000228811 | PRR4 | 0.1789 |
| ENSP00000342709 | PRR19 | 0.1789 |
| ENSP00000359198 | PPDPF | 0.1789 |
| ENSP00000416673 | PATL2 | 0.1789 |
| ENSP00000284288 | PANX3 | 0.1789 |
| ENSP00000334441 | OR10J5 | 0.1789 |
| ENSP00000353988 | OR10A3 | 0.1789 |
| ENSP00000264596 | NEIL3 | 0.1789 |
| ENSP00000368688 | LYRM7 | 0.1789 |
| ENSP00000410926 | LMLN | 0.1789 |
| ENSP00000340434 | LDOC1L | 0.1789 |
| ENSP00000007735 | KRT33A | 0.1789 |
| ENSP00000335388 | KIR3DX1 | 0.1789 |
| ENSP00000265272 | JAKMIP2 | 0.1789 |
| ENSP00000293756 | IP6K3 | 0.1789 |
| ENSP00000344860 | IGFL3 | 0.1789 |
| ENSP00000350848 | HMGN5 | 0.1789 |
| ENSP00000349493 | GUCA2A | 0.1789 |
| ENSP00000344446 | FGD6 | 0.1789 |
| ENSP00000237642 | FAM47E-STBD1 | 0.1789 |
| ENSP00000421364 | ECSCR | 0.1789 |
| ENSP00000362629 | DMRTC1 | 0.1789 |
| ENSP00000367422 | DLEU1 | 0.1789 |
| ENSP00000268676 | DEF8 | 0.1789 |
| ENSP00000353128 | DCAF12L2 | 0.1789 |
| ENSP00000459439 | CTD-3148I10.1 | 0.1789 |
| ENSP00000362029 | CIZ1 | 0.1789 |
| ENSP00000423567 | CCDC153 | 0.1789 |
| ENSP00000357507 | C1orf43 | 0.1789 |
| ENSP00000327506 | C16orf54 | 0.1789 |
| ENSP00000302918 | C11orf40 | 0.1789 |
| ENSP00000344929 | BPIFB6 | 0.1789 |
| ENSP00000257336 | BIVM | 0.1789 |
| ENSP00000219204 | ARL2BP | 0.1789 |
| ENSP00000472703 | AL035252.1 | 0.1789 |
| ENSP00000367432 | AJAP1 | 0.1789 |
| ENSP00000471312 | AC011530.4 | 0.1789 |
| ENSP00000380174 | ZSCAN29 | 0.1763 |
| ENSP00000247930 | ZNF777 | 0.1763 |
| ENSP00000344095 | ZNF683 | 0.1763 |
| ENSP00000375660 | ZNF320 | 0.1763 |
| ENSP00000321049 | ZNF227 | 0.1763 |
| ENSP00000462054 | ZFP92 | 0.1763 |
| ENSP00000216268 | ZBED4 | 0.1763 |
| ENSP00000323087 | WSCD1 | 0.1763 |
| ENSP00000280190 | WDR17 | 0.1763 |
| ENSP00000368544 | VIT | 0.1763 |
| ENSP00000316699 | TTC7A | 0.1763 |
| ENSP00000373485 | TSNAXIP1 | 0.1763 |

| | | |
|---|---|---|
| ENSP00000318409 | TMEM176B | 0.1763 |
| ENSP00000296059 | TM4SF18 | 0.1763 |
| ENSP00000202625 | TGM6 | 0.1763 |
| ENSP00000300258 | TCP10L | 0.1763 |
| ENSP00000258770 | TBRG4 | 0.1763 |
| ENSP00000312368 | SNX31 | 0.1763 |
| ENSP00000374125 | SIGLEC15 | 0.1763 |
| ENSP00000371040 | RLN2 | 0.1763 |
| ENSP00000307155 | RBMY1F | 0.1763 |
| ENSP00000286070 | RBM45 | 0.1763 |
| ENSP00000258083 | PRADC1 | 0.1763 |
| ENSP00000311153 | PILRB | 0.1763 |
| ENSP00000292140 | PHLDB3 | 0.1763 |
| ENSP00000334642 | PDZD8 | 0.1763 |
| ENSP00000263563 | PALD1 | 0.1763 |
| ENSP00000352671 | OSCAR | 0.1763 |
| ENSP00000323853 | OR8K5 | 0.1763 |
| ENSP00000353516 | OR2W3 | 0.1763 |
| ENSP00000277216 | OR13C4 | 0.1763 |
| ENSP00000359464 | OGFRL1 | 0.1763 |
| ENSP00000279028 | OCSTAMP | 0.1763 |
| ENSP00000378996 | NREP | 0.1763 |
| ENSP00000345684 | NBPF10 | 0.1763 |
| ENSP00000333800 | MRGPRX2 | 0.1763 |
| ENSP00000338706 | MID1IP1 | 0.1763 |
| ENSP00000453042 | LY6K | 0.1763 |
| ENSP00000329943 | LRRC36 | 0.1763 |
| ENSP00000352420 | LDLRAD4 | 0.1763 |
| ENSP00000375076 | KRTAP4-4 | 0.1763 |
| ENSP00000409964 | KIAA1430 | 0.1763 |
| ENSP00000324901 | IQCK | 0.1763 |
| ENSP00000301284 | HDGFRP2 | 0.1763 |
| ENSP00000334540 | GPR141 | 0.1763 |
| ENSP00000351473 | GIMAP5 | 0.1763 |
| ENSP00000410007 | FBXO46 | 0.1763 |
| ENSP00000428646 | FBXO41 | 0.1763 |
| ENSP00000335040 | FAM129C | 0.1763 |
| ENSP00000367229 | CEP68 | 0.1763 |
| ENSP00000377547 | CCNJL | 0.1763 |
| ENSP00000238156 | CCDC92 | 0.1763 |
| ENSP00000414964 | CCDC107 | 0.1763 |
| ENSP00000404464 | CCBE1 | 0.1763 |
| ENSP00000324204 | C7orf41 | 0.1763 |
| ENSP00000230301 | C6orf118 | 0.1763 |
| ENSP00000304410 | C2orf68 | 0.1763 |
| ENSP00000333592 | C21orf90 | 0.1763 |
| ENSP00000287859 | C1orf27 | 0.1763 |
| ENSP00000382157 | C14orf182 | 0.1763 |
| ENSP00000473193 | AC087645.1 | 0.1763 |
| ENSP00000354305 | ZSCAN12 | 0.1737 |
| ENSP00000265827 | ZNF800 | 0.1737 |
| ENSP00000404127 | ZNF709 | 0.1737 |
| ENSP00000346729 | ZNF589 | 0.1737 |

| | | |
|---|---|---|
| ENSP00000243643 | ZNF415 | 0.1737 |
| ENSP00000255129 | ZNF334 | 0.1737 |
| ENSP00000418897 | ZNF322 | 0.1737 |
| ENSP00000429803 | ZNF260 | 0.1737 |
| ENSP00000435310 | WDR65 | 0.1737 |
| ENSP00000456594 | TMEM178B | 0.1737 |
| ENSP00000456850 | THEGL | 0.1737 |
| ENSP00000380762 | TEN1 | 0.1737 |
| ENSP00000457168 | SZT2 | 0.1737 |
| ENSP00000299140 | SPATA19 | 0.1737 |
| ENSP00000415998 | SLC48A1 | 0.1737 |
| ENSP00000360398 | SLC25A27 | 0.1737 |
| ENSP00000326924 | SCAPER | 0.1737 |
| ENSP00000464100 | RP11-51F16.8 | 0.1737 |
| ENSP00000280258 | PRSS23 | 0.1737 |
| ENSP00000239666 | PDZD11 | 0.1737 |
| ENSP00000323805 | PALM2 | 0.1737 |
| ENSP00000325203 | OR6T1 | 0.1737 |
| ENSP00000331823 | OR5P2 | 0.1737 |
| ENSP00000369157 | OR10A4 | 0.1737 |
| ENSP00000331086 | ODF4 | 0.1737 |
| ENSP00000342156 | NCR3 | 0.1737 |
| ENSP00000418668 | MURC | 0.1737 |
| ENSP00000457957 | MEF2BNB-MEF2B | 0.1737 |
| ENSP00000312273 | LGI4 | 0.1737 |
| ENSP00000222224 | LENG1 | 0.1737 |
| ENSP00000402297 | LACTBL1 | 0.1737 |
| ENSP00000304437 | KNDC1 | 0.1737 |
| ENSP00000197268 | KIAA1467 | 0.1737 |
| ENSP00000365172 | KDELC1 | 0.1737 |
| ENSP00000360822 | KCNT1 | 0.1737 |
| ENSP00000380467 | JMJD7 | 0.1737 |
| ENSP00000265239 | IQCG | 0.1737 |
| ENSP00000270642 | IGLON5 | 0.1737 |
| ENSP00000417580 | IAH1 | 0.1737 |
| ENSP00000287474 | FRRS1 | 0.1737 |
| ENSP00000323034 | FAM83A | 0.1737 |
| ENSP00000294829 | FAM71A | 0.1737 |
| ENSP00000464743 | FAM215A | 0.1737 |
| ENSP00000361254 | FAM213A | 0.1737 |
| ENSP00000433343 | FAM118B | 0.1737 |
| ENSP00000302105 | CRYGA | 0.1737 |
| ENSP00000340568 | COX8C | 0.1737 |
| ENSP00000334767 | CCDC84 | 0.1737 |
| ENSP00000239830 | CCDC77 | 0.1737 |
| ENSP00000269503 | CBLN2 | 0.1737 |
| ENSP00000412886 | CAMTA2 | 0.1737 |
| ENSP00000364219 | CAMK2N1 | 0.1737 |
| ENSP00000297145 | C7orf60 | 0.1737 |
| ENSP00000442411 | C21orf119 | 0.1737 |
| ENSP00000331691 | C12orf60 | 0.1737 |
| ENSP00000340667 | C11orf96 | 0.1737 |
| ENSP00000378409 | C10orf54 | 0.1737 |

| | | |
|---|---|---|
| ENSP00000411217 | CC2D2B | 0.1737 |
| ENSP00000443399 | POM121L4P | 0.1737 |
| ENSP00000351883 | ZSCAN5B | 0.1711 |
| ENSP00000329738 | ZSCAN30 | 0.1711 |
| ENSP00000350295 | ZNF816 | 0.1711 |
| ENSP00000459566 | ZNF75A | 0.1711 |
| ENSP00000413411 | ZNF724P | 0.1711 |
| ENSP00000409514 | ZNF717 | 0.1711 |
| ENSP00000367300 | ZNF425 | 0.1711 |
| ENSP00000388864 | ZNF419 | 0.1711 |
| ENSP00000300870 | ZNF267 | 0.1711 |
| ENSP00000334854 | ZACN | 0.1711 |
| ENSP00000346466 | WDR91 | 0.1711 |
| ENSP00000328800 | WBP2NL | 0.1711 |
| ENSP00000367952 | UCMA | 0.1711 |
| ENSP00000279968 | TMEM218 | 0.1711 |
| ENSP00000376304 | TMEM121 | 0.1711 |
| ENSP00000345152 | TMEM120B | 0.1711 |
| ENSP00000243286 | TCEAL3 | 0.1711 |
| ENSP00000316609 | SWI5 | 0.1711 |
| ENSP00000357737 | SPRR2G | 0.1711 |
| ENSP00000357389 | SLC50A1 | 0.1711 |
| ENSP00000278426 | SLC43A1 | 0.1711 |
| ENSP00000376794 | SLC35G2 | 0.1711 |
| ENSP00000419249 | RIIAD1 | 0.1711 |
| ENSP00000417451 | RBMXL3 | 0.1711 |
| ENSP00000272203 | PLEKHA6 | 0.1711 |
| ENSP00000393860 | PLEKHA2 | 0.1711 |
| ENSP00000395505 | PATE3 | 0.1711 |
| ENSP00000302867 | OR7G3 | 0.1711 |
| ENSP00000310788 | OR5J2 | 0.1711 |
| ENSP00000328215 | OR56A4 | 0.1711 |
| ENSP00000326232 | OR52D1 | 0.1711 |
| ENSP00000467123 | NLRP7 | 0.1711 |
| ENSP00000409370 | NLRP2 | 0.1711 |
| ENSP00000387380 | NECAB1 | 0.1711 |
| ENSP00000362170 | NAIF1 | 0.1711 |
| ENSP00000363418 | MTFR1L | 0.1711 |
| ENSP00000415222 | MS4A4E | 0.1711 |
| ENSP00000458290 | METTL4 | 0.1711 |
| ENSP00000293274 | LYZL6 | 0.1711 |
| ENSP00000264094 | LOXL3 | 0.1711 |
| ENSP00000347635 | KRTAP13-1 | 0.1711 |
| ENSP00000344976 | IL1RAPL2 | 0.1711 |
| ENSP00000367130 | HES3 | 0.1711 |
| ENSP00000446872 | GATC | 0.1711 |
| ENSP00000358510 | FUNDC2 | 0.1711 |
| ENSP00000239906 | FAM53C | 0.1711 |
| ENSP00000317289 | FAM220A | 0.1711 |
| ENSP00000320520 | FAM211B | 0.1711 |
| ENSP00000429726 | FAM159A | 0.1711 |
| ENSP00000316938 | DNASE1L2 | 0.1711 |
| ENSP00000319126 | DEFB112 | 0.1711 |

| | | |
|---|---|---|
| ENSP00000356385 | CFHR2 | 0.1711 |
| ENSP00000315945 | CD163L1 | 0.1711 |
| ENSP00000262659 | CCM2L | 0.1711 |
| ENSP00000344749 | CCDC37 | 0.1711 |
| ENSP00000354888 | CCDC152 | 0.1711 |
| ENSP00000296824 | CCDC127 | 0.1711 |
| ENSP00000378145 | CCDC109B | 0.1711 |
| ENSP00000334289 | C9orf85 | 0.1711 |
| ENSP00000366402 | C9orf135 | 0.1711 |
| ENSP00000297001 | C7orf72 | 0.1711 |
| ENSP00000340887 | C5orf47 | 0.1711 |
| ENSP00000341139 | C3orf62 | 0.1711 |
| ENSP00000345107 | C2orf88 | 0.1711 |
| ENSP00000402389 | C1QTNF5 | 0.1711 |
| ENSP00000417061 | C1orf222 | 0.1711 |
| ENSP00000329920 | C19orf59 | 0.1711 |
| ENSP00000435352 | B3GNT6 | 0.1711 |
| ENSP00000416125 | ARHGEF38 | 0.1711 |
| ENSP00000274487 | ADAMTS19 | 0.1711 |
| ENSP00000387851 | AC104809.3 | 0.1711 |
| ENSP00000388519 | FAM74A1 | 0.1711 |
| ENSP00000340841 | ZNF621 | 0.1684 |
| ENSP00000303915 | ZNF561 | 0.1684 |
| ENSP00000353491 | ZNF460 | 0.1684 |
| ENSP00000309640 | XXYLT1 | 0.1684 |
| ENSP00000386141 | XKR9 | 0.1684 |
| ENSP00000218056 | WDR13 | 0.1684 |
| ENSP00000305069 | TMEM192 | 0.1684 |
| ENSP00000297205 | STEAP1 | 0.1684 |
| ENSP00000267260 | SRRM4 | 0.1684 |
| ENSP00000451542 | SOHLH2 | 0.1684 |
| ENSP00000433282 | SLC22A18AS | 0.1684 |
| ENSP00000306396 | RNF187 | 0.1684 |
| ENSP00000382492 | RADIL | 0.1684 |
| ENSP00000302319 | PRY | 0.1684 |
| ENSP00000336552 | PKIA | 0.1684 |
| ENSP00000259883 | PGBD1 | 0.1684 |
| ENSP00000307234 | PCDHB1 | 0.1684 |
| ENSP00000322486 | OTOS | 0.1684 |
| ENSP00000235532 | OSCP1 | 0.1684 |
| ENSP00000367649 | OR5D16 | 0.1684 |
| ENSP00000395369 | NBPF16 | 0.1684 |
| ENSP00000425864 | MEF2BNB | 0.1684 |
| ENSP00000290536 | M1AP | 0.1684 |
| ENSP00000364992 | LY6G6F | 0.1684 |
| ENSP00000339374 | LGALS12 | 0.1684 |
| ENSP00000340546 | KRTAP4-5 | 0.1684 |
| ENSP00000295878 | KIAA1407 | 0.1684 |
| ENSP00000324191 | KCNRG | 0.1684 |
| ENSP00000371054 | INSL6 | 0.1684 |
| ENSP00000381970 | GIN1 | 0.1684 |
| ENSP00000260447 | GCHFR | 0.1684 |
| ENSP00000237172 | FILIP1 | 0.1684 |

| | | |
|---|---|---|
| ENSP00000356481 | FAM129A | 0.1684 |
| ENSP00000313140 | EXD2 | 0.1684 |
| ENSP00000233712 | EVA1A | 0.1684 |
| ENSP00000278505 | ENDOD1 | 0.1684 |
| ENSP00000302289 | EMB | 0.1684 |
| ENSP00000372136 | DEFA1B | 0.1684 |
| ENSP00000375783 | DACT3 | 0.1684 |
| ENSP00000368511 | CXorf58 | 0.1684 |
| ENSP00000359596 | CLCA2 | 0.1684 |
| ENSP00000356779 | CCDC181 | 0.1684 |
| ENSP00000432870 | CABYR | 0.1684 |
| ENSP00000351422 | C19orf60 | 0.1684 |
| ENSP00000343493 | C17orf99 | 0.1684 |
| ENSP00000335229 | C17orf82 | 0.1684 |
| ENSP00000329106 | BPY2 | 0.1684 |
| ENSP00000371085 | BAIAP2L2 | 0.1684 |
| ENSP00000363416 | AUNIP | 0.1684 |
| ENSP00000352976 | AMZ2 | 0.1684 |
| ENSP00000369238 | ADAM32 | 0.1684 |
| ENSP00000329303 | LOC619207 | 0.1684 |
| ENSP00000360583 | ZYG11A | 0.1658 |
| ENSP00000398089 | ZNF821 | 0.1658 |
| ENSP00000321132 | ZNF554 | 0.1658 |
| ENSP00000353652 | ZNF528 | 0.1658 |
| ENSP00000379368 | ZNF35 | 0.1658 |
| ENSP00000250076 | ZNF232 | 0.1658 |
| ENSP00000361646 | ZMYND12 | 0.1658 |
| ENSP00000321309 | VCX2 | 0.1658 |
| ENSP00000230256 | UNC93A | 0.1658 |
| ENSP00000352265 | TPGS1 | 0.1658 |
| ENSP00000316940 | TMEM31 | 0.1658 |
| ENSP00000375053 | TMEM203 | 0.1658 |
| ENSP00000342148 | TMEM167B | 0.1658 |
| ENSP00000211314 | TMEM14A | 0.1658 |
| ENSP00000275560 | SRCRB4D | 0.1658 |
| ENSP00000299272 | SPIC | 0.1658 |
| ENSP00000361597 | SPATA25 | 0.1658 |
| ENSP00000295213 | SPATA18 | 0.1658 |
| ENSP00000449500 | RNASEK | 0.1658 |
| ENSP00000368869 | PRR16 | 0.1658 |
| ENSP00000306900 | PRND | 0.1658 |
| ENSP00000318075 | PLEKHJ1 | 0.1658 |
| ENSP00000352173 | PLAC1 | 0.1658 |
| ENSP00000469886 | PINLYP | 0.1658 |
| ENSP00000269919 | PGPEP1 | 0.1658 |
| ENSP00000309230 | PEAK1 | 0.1658 |
| ENSP00000345491 | PCDHB13 | 0.1658 |
| ENSP00000308764 | OR52I2 | 0.1658 |
| ENSP00000324557 | OR2M7 | 0.1658 |
| ENSP00000355432 | OR2M5 | 0.1658 |
| ENSP00000352710 | OR2M2 | 0.1658 |
| ENSP00000353044 | OR2L3 | 0.1658 |
| ENSP00000359384 | OOEP | 0.1658 |

| | | |
|---|---|---|
| ENSP00000331857 | NLRP9 | 0.1658 |
| ENSP00000342137 | NCCRP1 | 0.1658 |
| ENSP00000300184 | MS4A7 | 0.1658 |
| ENSP00000350833 | MPPED2 | 0.1658 |
| ENSP00000364985 | LY6G6D | 0.1658 |
| ENSP00000378733 | LBH | 0.1658 |
| ENSP00000372286 | KRTAP27-1 | 0.1658 |
| ENSP00000376213 | KIAA1683 | 0.1658 |
| ENSP00000452581 | IFRG15 | 0.1658 |
| ENSP00000421917 | HMGXB3 | 0.1658 |
| ENSP00000297468 | GPR146 | 0.1658 |
| ENSP00000238667 | FLVCR2 | 0.1658 |
| ENSP00000326652 | FAM71F1 | 0.1658 |
| ENSP00000286692 | DRAM2 | 0.1658 |
| ENSP00000361795 | DBNDD2 | 0.1658 |
| ENSP00000284617 | CCSAP | 0.1658 |
| ENSP00000343290 | C9orf171 | 0.1658 |
| ENSP00000340864 | C1QTNF1 | 0.1658 |
| ENSP00000356700 | C1orf105 | 0.1658 |
| ENSP00000299088 | C14orf93 | 0.1658 |
| ENSP00000366317 | C10orf114 | 0.1658 |
| ENSP00000332389 | BRICD5 | 0.1658 |
| ENSP00000403910 | ATP5SL | 0.1658 |
| ENSP00000386485 | APOPT1 | 0.1658 |
| ENSP00000442163 | AL590867.1 | 0.1658 |
| ENSP00000324196 | AATK | 0.1658 |
| ENSP00000331465 | ZNF84 | 0.1632 |
| ENSP00000317125 | ZNF726 | 0.1632 |
| ENSP00000301310 | ZNF582 | 0.1632 |
| ENSP00000400878 | ZNF234 | 0.1632 |
| ENSP00000306351 | ZNF182 | 0.1632 |
| ENSP00000415070 | ZNF177 | 0.1632 |
| ENSP00000263849 | ZC2HC1A | 0.1632 |
| ENSP00000364739 | XAGE1C | 0.1632 |
| ENSP00000296679 | WDR41 | 0.1632 |
| ENSP00000334148 | WDR25 | 0.1632 |
| ENSP00000319486 | TSPAN16 | 0.1632 |
| ENSP00000356211 | TMEM183A | 0.1632 |
| ENSP00000327234 | TMCO5A | 0.1632 |
| ENSP00000240691 | TAS2R9 | 0.1632 |
| ENSP00000448219 | TAS2R38 | 0.1632 |
| ENSP00000388731 | SSFA2 | 0.1632 |
| ENSP00000351884 | SPANXC | 0.1632 |
| ENSP00000428541 | SMIM12 | 0.1632 |
| ENSP00000387617 | SMCO2 | 0.1632 |
| ENSP00000428612 | SLC10A5 | 0.1632 |
| ENSP00000372449 | RP11-408E5.4 | 0.1632 |
| ENSP00000262765 | QRICH2 | 0.1632 |
| ENSP00000358714 | PRSS35 | 0.1632 |
| ENSP00000223864 | PLGRKT | 0.1632 |
| ENSP00000363654 | PALM2-AKAP2 | 0.1632 |
| ENSP00000355374 | OSGIN1 | 0.1632 |
| ENSP00000345563 | OR7D2 | 0.1632 |

| | | |
|---|---|---|
| ENSP00000326259 | OR52H1 | 0.1632 |
| ENSP00000365451 | OR4D1 | 0.1632 |
| ENSP00000333680 | NFAM1 | 0.1632 |
| ENSP00000325402 | MYPOP | 0.1632 |
| ENSP00000200691 | MT3 | 0.1632 |
| ENSP00000340519 | LRRC37B | 0.1632 |
| ENSP00000361036 | LHFPL1 | 0.1632 |
| ENSP00000356121 | LEMD1 | 0.1632 |
| ENSP00000411070 | LCE6A | 0.1632 |
| ENSP00000420723 | KRTAP5-8 | 0.1632 |
| ENSP00000398619 | KRTAP25-1 | 0.1632 |
| ENSP00000372276 | KRTAP20-3 | 0.1632 |
| ENSP00000372272 | KRTAP19-8 | 0.1632 |
| ENSP00000251343 | KHNYN | 0.1632 |
| ENSP00000310729 | HERPUD2 | 0.1632 |
| ENSP00000369943 | GCOM1 | 0.1632 |
| ENSP00000373565 | FAM83H | 0.1632 |
| ENSP00000339207 | FAM122B | 0.1632 |
| ENSP00000358456 | F8A1 | 0.1632 |
| ENSP00000381086 | CXorf36 | 0.1632 |
| ENSP00000364348 | CCDC180 | 0.1632 |
| ENSP00000395995 | CCDC141 | 0.1632 |
| ENSP00000340776 | CCDC108 | 0.1632 |
| ENSP00000361025 | C9orf96 | 0.1632 |
| ENSP00000307129 | C8orf74 | 0.1632 |
| ENSP00000412215 | C4orf45 | 0.1632 |
| ENSP00000295079 | C2orf47 | 0.1632 |
| ENSP00000323199 | C18orf32 | 0.1632 |
| ENSP00000328640 | C11orf72 | 0.1632 |
| ENSP00000342197 | BTNL8 | 0.1632 |
| ENSP00000364632 | BPIFB4 | 0.1632 |
| ENSP00000360899 | BEND5 | 0.1632 |
| ENSP00000328083 | ARHGEF37 | 0.1632 |
| ENSP00000344577 | APOL3 | 0.1632 |
| ENSP00000315915 | ANXA2R | 0.1632 |
| ENSP00000352248 | ANXA10 | 0.1632 |
| ENSP00000281471 | AMN1 | 0.1632 |
| ENSP00000410395 | C12orf52 | 0.1632 |
| ENSP00000309812 | ZNF80 | 0.1605 |
| ENSP00000386393 | ZNF630 | 0.1605 |
| ENSP00000301215 | ZNF526 | 0.1605 |
| ENSP00000397693 | ZNF28 | 0.1605 |
| ENSP00000375822 | ZNF222 | 0.1605 |
| ENSP00000342019 | ZNF189 | 0.1605 |
| ENSP00000420727 | ZNF181 | 0.1605 |
| ENSP00000221327 | ZNF180 | 0.1605 |
| ENSP00000269499 | ZCCHC2 | 0.1605 |
| ENSP00000361875 | WFDC5 | 0.1605 |
| ENSP00000431371 | VKORC1 | 0.1605 |
| ENSP00000414144 | TMEM236 | 0.1605 |
| ENSP00000372982 | TMEM200C | 0.1605 |
| ENSP00000293261 | TMEM143 | 0.1605 |
| ENSP00000440509 | TIFAB | 0.1605 |

| | | |
|---|---|---|
| ENSP00000364482 | STK19 | 0.1605 |
| ENSP00000366081 | SSX4B | 0.1605 |
| ENSP00000369611 | SPZ1 | 0.1605 |
| ENSP00000341442 | SNTN | 0.1605 |
| ENSP00000366283 | SLITRK5 | 0.1605 |
| ENSP00000273905 | SLC10A6 | 0.1605 |
| ENSP00000410041 | SKIDA1 | 0.1605 |
| ENSP00000290894 | SHF | 0.1605 |
| ENSP00000368502 | SCGB2B2 | 0.1605 |
| ENSP00000229903 | SAYSD1 | 0.1605 |
| ENSP00000356444 | SAMD5 | 0.1605 |
| ENSP00000283243 | PLA2R1 | 0.1605 |
| ENSP00000473005 | PIH1 | 0.1605 |
| ENSP00000341911 | PALM | 0.1605 |
| ENSP00000364110 | PAGE2B | 0.1605 |
| ENSP00000305640 | OR6F1 | 0.1605 |
| ENSP00000303096 | OR5A1 | 0.1605 |
| ENSP00000323224 | OR52N4 | 0.1605 |
| ENSP00000324369 | OR2T35 | 0.1605 |
| ENSP00000352904 | NT5C1B | 0.1605 |
| ENSP00000375063 | NLRP5 | 0.1605 |
| ENSP00000391116 | NBPF12 | 0.1605 |
| ENSP00000300303 | NAT16 | 0.1605 |
| ENSP00000368468 | MCUR1 | 0.1605 |
| ENSP00000324175 | MAMSTR | 0.1605 |
| ENSP00000375622 | LAIR1 | 0.1605 |
| ENSP00000339238 | KRTAP24-1 | 0.1605 |
| ENSP00000375237 | KRTAP2-3 | 0.1605 |
| ENSP00000372278 | KRTAP20-4 | 0.1605 |
| ENSP00000357840 | KIAA1919 | 0.1605 |
| ENSP00000307461 | KIAA1024 | 0.1605 |
| ENSP00000371927 | GPR78 | 0.1605 |
| ENSP00000415890 | GPR35 | 0.1605 |
| ENSP00000305107 | GIMAP8 | 0.1605 |
| ENSP00000158009 | FNDC8 | 0.1605 |
| ENSP00000346918 | DHRS4-AS1 | 0.1605 |
| ENSP00000322191 | DEFB104B | 0.1605 |
| ENSP00000368245 | CXorf21 | 0.1605 |
| ENSP00000421736 | CR1L | 0.1605 |
| ENSP00000402505 | CPAMD8 | 0.1605 |
| ENSP00000299295 | CHODL | 0.1605 |
| ENSP00000315111 | C8orf42 | 0.1605 |
| ENSP00000420610 | C6orf201 | 0.1605 |
| ENSP00000406925 | C4orf26 | 0.1605 |
| ENSP00000418511 | C21orf62 | 0.1605 |
| ENSP00000360244 | C1orf87 | 0.1605 |
| ENSP00000360045 | C1orf141 | 0.1605 |
| ENSP00000386499 | C16orf11 | 0.1605 |
| ENSP00000443007 | C12orf36 | 0.1605 |
| ENSP00000253362 | BPIFA2 | 0.1605 |
| ENSP00000367360 | BLACE | 0.1605 |
| ENSP00000266581 | AMIGO2 | 0.1605 |
| ENSP00000470580 | AC027228.1 | 0.1605 |

| | | |
|---|---|---|
| ENSP00000360245 | C10orf131 | 0.1605 |
| ENSP00000394086 | ZNF84 | 0.1579 |
| ENSP00000410890 | ZNF783 | 0.1579 |
| ENSP00000348673 | ZNF770 | 0.1579 |
| ENSP00000355251 | ZNF511 | 0.1579 |
| ENSP00000301547 | ZNF443 | 0.1579 |
| ENSP00000471077 | ZNF417 | 0.1579 |
| ENSP00000349882 | ZNF248 | 0.1579 |
| ENSP00000463741 | ZNF19 | 0.1579 |
| ENSP00000275766 | ZC3HAV1L | 0.1579 |
| ENSP00000362560 | VSTM2L | 0.1579 |
| ENSP00000404251 | VGLL4 | 0.1579 |
| ENSP00000416168 | TRANK1 | 0.1579 |
| ENSP00000292114 | TMEM199 | 0.1579 |
| ENSP00000296582 | TMEM184C | 0.1579 |
| ENSP00000233047 | TMEM159 | 0.1579 |
| ENSP00000278826 | TMEM138 | 0.1579 |
| ENSP00000410852 | TMEM132C | 0.1579 |
| ENSP00000389466 | TGM7 | 0.1579 |
| ENSP00000397002 | TEX22 | 0.1579 |
| ENSP00000278550 | TENM4 | 0.1579 |
| ENSP00000302077 | SLFN12 | 0.1579 |
| ENSP00000357116 | SAMD3 | 0.1579 |
| ENSP00000308820 | RIC3 | 0.1579 |
| ENSP00000300557 | PRR15L | 0.1579 |
| ENSP00000472066 | PRED60 | 0.1579 |
| ENSP00000221462 | PPP1R37 | 0.1579 |
| ENSP00000338067 | PKIG | 0.1579 |
| ENSP00000360054 | PHACTR3 | 0.1579 |
| ENSP00000364089 | PAGE3 | 0.1579 |
| ENSP00000364261 | OTUD3 | 0.1579 |
| ENSP00000323982 | OR8H2 | 0.1579 |
| ENSP00000318878 | OR4F21 | 0.1579 |
| ENSP00000321419 | OR4C3 | 0.1579 |
| ENSP00000324534 | OR14C36 | 0.1579 |
| ENSP00000251921 | NXPE1 | 0.1579 |
| ENSP00000253262 | NUTMF | 0.1579 |
| ENSP00000279206 | NUDT22 | 0.1579 |
| ENSP00000334437 | NUDT17 | 0.1579 |
| ENSP00000363520 | NIPAL3 | 0.1579 |
| ENSP00000325978 | LRRC31 | 0.1579 |
| ENSP00000275635 | LAT2 | 0.1579 |
| ENSP00000328270 | KRTAP4-6 | 0.1579 |
| ENSP00000375151 | KRTAP4-3 | 0.1579 |
| ENSP00000366923 | IGFBPL1 | 0.1579 |
| ENSP00000372059 | IFITM5 | 0.1579 |
| ENSP00000349364 | IFFO1 | 0.1579 |
| ENSP00000299633 | HDGFRP3 | 0.1579 |
| ENSP00000230012 | HDGFL1 | 0.1579 |
| ENSP00000258456 | GPR45 | 0.1579 |
| ENSP00000345060 | GPR179 | 0.1579 |
| ENSP00000452571 | GATSL2 | 0.1579 |
| ENSP00000362570 | FNDC5 | 0.1579 |

| | | |
|---|---|---|
| ENSP00000270620 | FAM71E1 | 0.1579 |
| ENSP00000341565 | FAM111B | 0.1579 |
| ENSP00000369371 | EQTN | 0.1579 |
| ENSP00000359396 | DPPA5 | 0.1579 |
| ENSP00000316193 | DNASE1L3 | 0.1579 |
| ENSP00000325954 | CDHR3 | 0.1579 |
| ENSP00000379566 | CCHCR1 | 0.1579 |
| ENSP00000295171 | CCDC74A | 0.1579 |
| ENSP00000347586 | CCDC69 | 0.1579 |
| ENSP00000359299 | CCDC18 | 0.1579 |
| ENSP00000341684 | CCDC15 | 0.1579 |
| ENSP00000295124 | CCDC138 | 0.1579 |
| ENSP00000350856 | C8orf86 | 0.1579 |
| ENSP00000386139 | C5orf55 | 0.1579 |
| ENSP00000266542 | C1RL | 0.1579 |
| ENSP00000298699 | C12orf50 | 0.1579 |
| ENSP00000227349 | C11orf63 | 0.1579 |
| ENSP00000367528 | AARD | 0.1579 |
| ENSP00000375229 | DEFB117 | 0.1579 |
| ENSP00000468862 | ZNF714 | 0.1553 |
| ENSP00000364645 | ZNF484 | 0.1553 |
| ENSP00000281523 | ZNF385D | 0.1553 |
| ENSP00000467018 | ZNF223 | 0.1553 |
| ENSP00000340590 | ZCCHC16 | 0.1553 |
| ENSP00000361755 | WFDC6 | 0.1553 |
| ENSP00000370420 | VCX3B | 0.1553 |
| ENSP00000222275 | UPK1A | 0.1553 |
| ENSP00000273610 | UCN2 | 0.1553 |
| ENSP00000401477 | TMEM232 | 0.1553 |
| ENSP00000394178 | TMEM182 | 0.1553 |
| ENSP00000316532 | TMEM132E | 0.1553 |
| ENSP00000272643 | THSD7B | 0.1553 |
| ENSP00000436450 | TAS2R46 | 0.1553 |
| ENSP00000375091 | TAS2R19 | 0.1553 |
| ENSP00000331474 | SYNDIG1L | 0.1553 |
| ENSP00000298596 | STOX1 | 0.1553 |
| ENSP00000329565 | SPDYE3 | 0.1553 |
| ENSP00000417063 | SPATA6L | 0.1553 |
| ENSP00000318900 | SOBP | 0.1553 |
| ENSP00000296444 | SHISA5 | 0.1553 |
| ENSP00000417556 | RP11-101E3.5 | 0.1553 |
| ENSP00000383093 | RFPL3S | 0.1553 |
| ENSP00000250831 | RBMY1J | 0.1553 |
| ENSP00000372484 | RBMY1B | 0.1553 |
| ENSP00000305005 | PSG8 | 0.1553 |
| ENSP00000258229 | PCNXL2 | 0.1553 |
| ENSP00000278360 | PAMR1 | 0.1553 |
| ENSP00000326708 | OVCH1 | 0.1553 |
| ENSP00000342356 | OSTN | 0.1553 |
| ENSP00000444134 | OR7G1 | 0.1553 |
| ENSP00000324111 | OR5AS1 | 0.1553 |
| ENSP00000344040 | OR2S2 | 0.1553 |
| ENSP00000386222 | OR2F2 | 0.1553 |

| | | |
|---|---|---|
| ENSP00000361397 | NUTMG | 0.1553 |
| ENSP00000394623 | NUTM2B | 0.1553 |
| ENSP00000409898 | NLRP11 | 0.1553 |
| ENSP00000385903 | NAA60 | 0.1553 |
| ENSP00000219162 | MT4 | 0.1553 |
| ENSP00000245564 | MSTO1 | 0.1553 |
| ENSP00000321455 | MICU3 | 0.1553 |
| ENSP00000252229 | MICB | 0.1553 |
| ENSP00000379201 | METTL21D | 0.1553 |
| ENSP00000386049 | MBD5 | 0.1553 |
| ENSP00000219168 | LYRM1 | 0.1553 |
| ENSP00000321026 | LURAP1L | 0.1553 |
| ENSP00000274382 | LIX1 | 0.1553 |
| ENSP00000334560 | KRTAP6-2 | 0.1553 |
| ENSP00000264651 | KRT24 | 0.1553 |
| ENSP00000291860 | KIR3DL3 | 0.1553 |
| ENSP00000259211 | IL36A | 0.1553 |
| ENSP00000361569 | IER5L | 0.1553 |
| ENSP00000348316 | HN1 | 0.1553 |
| ENSP00000394954 | GSG1L | 0.1553 |
| ENSP00000261530 | GPATCH2L | 0.1553 |
| ENSP00000380488 | GALNT9 | 0.1553 |
| ENSP00000280236 | FSIP1 | 0.1553 |
| ENSP00000321386 | FBXO39 | 0.1553 |
| ENSP00000297532 | FASTK | 0.1553 |
| ENSP00000328307 | FAM47B | 0.1553 |
| ENSP00000332148 | DZIP1L | 0.1553 |
| ENSP00000383115 | COLEC12 | 0.1553 |
| ENSP00000402355 | CDRT15 | 0.1553 |
| ENSP00000373827 | CCDC150 | 0.1553 |
| ENSP00000283233 | CCDC148 | 0.1553 |
| ENSP00000331625 | C3orf35 | 0.1553 |
| ENSP00000396192 | C1QTNF9B-AS1 | 0.1553 |
| ENSP00000453969 | C15orf52 | 0.1553 |
| ENSP00000341610 | C15orf48 | 0.1553 |
| ENSP00000300399 | BPIFC | 0.1553 |
| ENSP00000364603 | BPIFA3 | 0.1553 |
| ENSP00000376417 | ARMC2 | 0.1553 |
| ENSP00000274979 | ANO7 | 0.1553 |
| ENSP00000321627 | ANKFN1 | 0.1553 |
| ENSP00000177648 | ALPK1 | 0.1553 |
| ENSP00000364567 | ADPRHL1 | 0.1553 |
| ENSP00000350540 | GYG2P1 | 0.1553 |
| ENSP00000410466 | ZNF90 | 0.1526 |
| ENSP00000339314 | ZNF70 | 0.1526 |
| ENSP00000321848 | ZNF671 | 0.1526 |
| ENSP00000379451 | ZNF418 | 0.1526 |
| ENSP00000397178 | ZNF300 | 0.1526 |
| ENSP00000402644 | TRIM16 | 0.1526 |
| ENSP00000322097 | TPRXL | 0.1526 |
| ENSP00000004103 | TMEM176A | 0.1526 |
| ENSP00000313408 | TMC6 | 0.1526 |
| ENSP00000292090 | TLCD1 | 0.1526 |

| | | |
|---|---|---|
| ENSP00000365200 | TEX30 | 0.1526 |
| ENSP00000375095 | TAS2R13 | 0.1526 |
| ENSP00000386439 | SYNC | 0.1526 |
| ENSP00000308727 | SUSD5 | 0.1526 |
| ENSP00000248933 | SEZ6L | 0.1526 |
| ENSP00000453854 | SECISBP2L | 0.1526 |
| ENSP00000432487 | SAAL1 | 0.1526 |
| ENSP00000305151 | PWWP2A | 0.1526 |
| ENSP00000244296 | PSG1 | 0.1526 |
| ENSP00000394510 | PRR12 | 0.1526 |
| ENSP00000332123 | PROSAPIP1 | 0.1526 |
| ENSP00000355260 | PPP1R14C | 0.1526 |
| ENSP00000304188 | OR8U1 | 0.1526 |
| ENSP00000248072 | OR7C2 | 0.1526 |
| ENSP00000384563 | OR6B2 | 0.1526 |
| ENSP00000386151 | OR6B1 | 0.1526 |
| ENSP00000305011 | OR4K14 | 0.1526 |
| ENSP00000355429 | OR2T5 | 0.1526 |
| ENSP00000355431 | OR2T4 | 0.1526 |
| ENSP00000283225 | OR14K1 | 0.1526 |
| ENSP00000361104 | OBP2B | 0.1526 |
| ENSP00000368389 | NGRN | 0.1526 |
| ENSP00000382856 | MTRNR2L4 | 0.1526 |
| ENSP00000288087 | MDP1 | 0.1526 |
| ENSP00000366418 | LZIC | 0.1526 |
| ENSP00000364978 | LY6G6C | 0.1526 |
| ENSP00000352601 | LRP10 | 0.1526 |
| ENSP00000357736 | LELP1 | 0.1526 |
| ENSP00000345797 | KRBOX4 | 0.1526 |
| ENSP00000350881 | GRAMD1C | 0.1526 |
| ENSP00000391465 | GAGE12I | 0.1526 |
| ENSP00000264703 | FNDC4 | 0.1526 |
| ENSP00000331397 | FAM43B | 0.1526 |
| ENSP00000378710 | FAM135B | 0.1526 |
| ENSP00000364324 | FAM120C | 0.1526 |
| ENSP00000464167 | CTD-2510F5.6 | 0.1526 |
| ENSP00000352295 | CGB2 | 0.1526 |
| ENSP00000365465 | CDSN | 0.1526 |
| ENSP00000381272 | CD200R1L | 0.1526 |
| ENSP00000256652 | CD101 | 0.1526 |
| ENSP00000373304 | CCDC174 | 0.1526 |
| ENSP00000343087 | CCDC157 | 0.1526 |
| ENSP00000407763 | CCDC122 | 0.1526 |
| ENSP00000252939 | CALY | 0.1526 |
| ENSP00000414893 | C9orf53 | 0.1526 |
| ENSP00000427820 | C8orf34 | 0.1526 |
| ENSP00000330596 | C22orf29 | 0.1526 |
| ENSP00000299191 | C16orf78 | 0.1526 |
| ENSP00000301031 | C16orf55 | 0.1526 |
| ENSP00000433721 | C11orf54 | 0.1526 |
| ENSP00000314914 | AMER3 | 0.1526 |
| ENSP00000256412 | ADAMDEC1 | 0.1526 |
| ENSP00000366511 | AC025278.1 | 0.1526 |

| | | |
|---|---|---|
| ENSP00000425679 | SNX29P2 | 0.1526 |
| ENSP00000390179 | ZNF527 | 0.1500 |
| ENSP00000388421 | ZNF487 | 0.1500 |
| ENSP00000381304 | ZFP90 | 0.1500 |
| ENSP00000362047 | XKRX | 0.1500 |
| ENSP00000310303 | TOPAZ1 | 0.1500 |
| ENSP00000319009 | TMEM45A | 0.1500 |
| ENSP00000303987 | TMEM223 | 0.1500 |
| ENSP00000438863 | TMEM206 | 0.1500 |
| ENSP00000296776 | TMEM174 | 0.1500 |
| ENSP00000400500 | THAP5 | 0.1500 |
| ENSP00000371243 | TEX33 | 0.1500 |
| ENSP00000357796 | TCHHL1 | 0.1500 |
| ENSP00000311257 | STOX2 | 0.1500 |
| ENSP00000453502 | ST20 | 0.1500 |
| ENSP00000341324 | SRFBP1 | 0.1500 |
| ENSP00000315554 | SPEM1 | 0.1500 |
| ENSP00000354886 | SLC25A47 | 0.1500 |
| ENSP00000357199 | RRNAD1 | 0.1500 |
| ENSP00000272324 | REG3G | 0.1500 |
| ENSP00000372105 | RBMY1E | 0.1500 |
| ENSP00000372124 | RBMY1D | 0.1500 |
| ENSP00000415026 | PRRT4 | 0.1500 |
| ENSP00000240189 | PRAMEF2 | 0.1500 |
| ENSP00000469432 | PKD1L2 | 0.1500 |
| ENSP00000320017 | PHYHIP | 0.1500 |
| ENSP00000351325 | PATE2 | 0.1500 |
| ENSP00000325076 | OR10G4 | 0.1500 |
| ENSP00000389948 | NT5C3B | 0.1500 |
| ENSP00000343891 | NLRP13 | 0.1500 |
| ENSP00000358203 | NBPF20 | 0.1500 |
| ENSP00000372202 | N6AMT2 | 0.1500 |
| ENSP00000340467 | LRRC16B | 0.1500 |
| ENSP00000270238 | LMTK3 | 0.1500 |
| ENSP00000328444 | KRTAP4-8 | 0.1500 |
| ENSP00000334866 | KRTAP15-1 | 0.1500 |
| ENSP00000256544 | KATNBL1 | 0.1500 |
| ENSP00000308782 | GP6 | 0.1500 |
| ENSP00000313423 | GFRA4 | 0.1500 |
| ENSP00000438970 | FLJ22184 | 0.1500 |
| ENSP00000263266 | FAM83E | 0.1500 |
| ENSP00000334285 | FAM170A | 0.1500 |
| ENSP00000349336 | FAM163B | 0.1500 |
| ENSP00000370812 | ESM1 | 0.1500 |
| ENSP00000303779 | EME2 | 0.1500 |
| ENSP00000263196 | DGCR2 | 0.1500 |
| ENSP00000424598 | DEFB4B | 0.1500 |
| ENSP00000389792 | DCDC1 | 0.1500 |
| ENSP00000354028 | CYB561 | 0.1500 |
| ENSP00000469018 | CTD-3138B18.4 | 0.1500 |
| ENSP00000323978 | CRIPAK | 0.1500 |
| ENSP00000291715 | CLDND2 | 0.1500 |
| ENSP00000433998 | CKLF-CMTM1 | 0.1500 |

| | | |
|---|---|---|
| ENSP00000415528 | CCDC81 | 0.1500 |
| ENSP00000339280 | CCDC30 | 0.1500 |
| ENSP00000379754 | CCDC125 | 0.1500 |
| ENSP00000358798 | CALHM3 | 0.1500 |
| ENSP00000316137 | C22orf43 | 0.1500 |
| ENSP00000249079 | C22orf23 | 0.1500 |
| ENSP00000252032 | C20orf194 | 0.1500 |
| ENSP00000364370 | C20orf144 | 0.1500 |
| ENSP00000354968 | C1orf191 | 0.1500 |
| ENSP00000344218 | C1orf162 | 0.1500 |
| ENSP00000298298 | C10orf25 | 0.1500 |
| ENSP00000307041 | BNC1 | 0.1500 |
| ENSP00000276416 | BIN3 | 0.1500 |
| ENSP00000363118 | ASAH2B | 0.1500 |
| ENSP00000313513 | ANKAR | 0.1500 |
| ENSP00000316224 | ALS2CR8 | 0.1500 |
| ENSP00000303769 | AKNA | 0.1500 |
| ENSP00000247087 | AHDC1 | 0.1500 |
| ENSP00000365394 | MICA | 0.1500 |
| ENSP00000437781 | ENSG00000228006 | 0.1500 |
| ENSP00000376110 | ZNF493 | 0.1474 |
| ENSP00000321370 | ZBED2 | 0.1474 |
| ENSP00000335038 | VSTM2B | 0.1474 |
| ENSP00000342938 | UPK3BL | 0.1474 |
| ENSP00000362562 | TMEM54 | 0.1474 |
| ENSP00000366528 | TMEM252 | 0.1474 |
| ENSP00000351732 | TMC2 | 0.1474 |
| ENSP00000366058 | SYNDIG1 | 0.1474 |
| ENSP00000424400 | SPATS1 | 0.1474 |
| ENSP00000405202 | SPANXB2 | 0.1474 |
| ENSP00000255674 | RTTN | 0.1474 |
| ENSP00000265806 | R3HCC1 | 0.1474 |
| ENSP00000416126 | PRAP1 | 0.1474 |
| ENSP00000386359 | PPP1R1C | 0.1474 |
| ENSP00000367655 | PKHD1L1 | 0.1474 |
| ENSP00000362987 | PHYHIPL | 0.1474 |
| ENSP00000411439 | PATE4 | 0.1474 |
| ENSP00000248058 | OR7A10 | 0.1474 |
| ENSP00000436424 | OR4D10 | 0.1474 |
| ENSP00000350248 | OR14A16 | 0.1474 |
| ENSP00000272907 | NYAP2 | 0.1474 |
| ENSP00000274605 | N4BP3 | 0.1474 |
| ENSP00000266263 | MTFP1 | 0.1474 |
| ENSP00000321737 | MROH1 | 0.1474 |
| ENSP00000371811 | MICU2 | 0.1474 |
| ENSP00000471094 | LRRC3DN | 0.1474 |
| ENSP00000325713 | LRRC37A3 | 0.1474 |
| ENSP00000383219 | KRTAP10-6 | 0.1474 |
| ENSP00000305263 | KRT28 | 0.1474 |
| ENSP00000356560 | KIAA1614 | 0.1474 |
| ENSP00000310338 | KIAA1551 | 0.1474 |
| ENSP00000364526 | KIAA1462 | 0.1474 |
| ENSP00000386711 | JAKMIP1 | 0.1474 |

| | | |
|---|---|---|
| ENSP00000376824 | IFI27L1 | 0.1474 |
| ENSP00000298251 | HEPACAM | 0.1474 |
| ENSP00000363582 | GGNBP1 | 0.1474 |
| ENSP00000265342 | FSTL4 | 0.1474 |
| ENSP00000366995 | FRMPD1 | 0.1474 |
| ENSP00000365167 | FHAD1 | 0.1474 |
| ENSP00000358820 | EPS8L3 | 0.1474 |
| ENSP00000344563 | CLEC12B | 0.1474 |
| ENSP00000388457 | CLDND1 | 0.1474 |
| ENSP00000349971 | CEACAM3 | 0.1474 |
| ENSP00000278520 | CCDC82 | 0.1474 |
| ENSP00000297324 | C8orf48 | 0.1474 |
| ENSP00000346931 | C6orf120 | 0.1474 |
| ENSP00000310182 | C4orf32 | 0.1474 |
| ENSP00000387124 | C2orf78 | 0.1474 |
| ENSP00000388953 | C21orf37 | 0.1474 |
| ENSP00000310801 | C20orf201 | 0.1474 |
| ENSP00000420716 | C1orf228 | 0.1474 |
| ENSP00000286031 | C1orf112 | 0.1474 |
| ENSP00000415027 | C16orf96 | 0.1474 |
| ENSP00000216445 | C14orf105 | 0.1474 |
| ENSP00000261250 | C12orf4 | 0.1474 |
| ENSP00000334848 | C11orf74 | 0.1474 |
| ENSP00000359050 | C10orf76 | 0.1474 |
| ENSP00000470752 | AL117190.3 | 0.1474 |
| ENSP00000265707 | ADAM18 | 0.1474 |
| ENSP00000472832 | AC090427.1 | 0.1474 |
| ENSP00000289788 | ZSCAN23 | 0.1447 |
| ENSP00000361784 | ZNF684 | 0.1447 |
| ENSP00000320347 | ZFP3 | 0.1447 |
| ENSP00000223273 | YAE1D1 | 0.1447 |
| ENSP00000362991 | XKR8 | 0.1447 |
| ENSP00000377428 | WDR52 | 0.1447 |
| ENSP00000331062 | VSTM4 | 0.1447 |
| ENSP00000335623 | VSIG10L | 0.1447 |
| ENSP00000344942 | UNC119B | 0.1447 |
| ENSP00000365714 | TTYH1 | 0.1447 |
| ENSP00000335004 | TRABD2A | 0.1447 |
| ENSP00000315635 | TMEM25 | 0.1447 |
| ENSP00000358999 | TMEM187 | 0.1447 |
| ENSP00000343635 | TLDC1 | 0.1447 |
| ENSP00000240687 | TAS2R7 | 0.1447 |
| ENSP00000332163 | SPRR4 | 0.1447 |
| ENSP00000360272 | SORBS1 | 0.1447 |
| ENSP00000339834 | SNRNP48 | 0.1447 |
| ENSP00000326445 | SHISA3 | 0.1447 |
| ENSP00000354622 | RUNDC1 | 0.1447 |
| ENSP00000276173 | RIPPLY1 | 0.1447 |
| ENSP00000438590 | RHNO1 | 0.1447 |
| ENSP00000367318 | RGP1 | 0.1447 |
| ENSP00000292125 | PSG6 | 0.1447 |
| ENSP00000335675 | PRELID2 | 0.1447 |
| ENSP00000386133 | POM121L12 | 0.1447 |

| | | |
|---|---|---|
| ENSP00000317175 | PLEKHD1 | 0.1447 |
| ENSP00000303111 | OR5AP2 | 0.1447 |
| ENSP00000317482 | OR4F4 | 0.1447 |
| ENSP00000367664 | OR4A5 | 0.1447 |
| ENSP00000313384 | OR1E1 | 0.1447 |
| ENSP00000303151 | OR1B1 | 0.1447 |
| ENSP00000355441 | OR14A2 | 0.1447 |
| ENSP00000394546 | NHSL1 | 0.1447 |
| ENSP00000262146 | MTFR1 | 0.1447 |
| ENSP00000431287 | METTL12 | 0.1447 |
| ENSP00000367185 | LYPD5 | 0.1447 |
| ENSP00000372910 | LY6G6E | 0.1447 |
| ENSP00000382815 | KIAA2026 | 0.1447 |
| ENSP00000244314 | IRGC | 0.1447 |
| ENSP00000253410 | HIGD1B | 0.1447 |
| ENSP00000364180 | GPSM3 | 0.1447 |
| ENSP00000269209 | GAREM | 0.1447 |
| ENSP00000308591 | GAL3ST3 | 0.1447 |
| ENSP00000361413 | FIBCD1 | 0.1447 |
| ENSP00000315247 | FAM71C | 0.1447 |
| ENSP00000360437 | FAM210B | 0.1447 |
| ENSP00000365585 | FAM182B | 0.1447 |
| ENSP00000267594 | FAM181A | 0.1447 |
| ENSP00000312753 | FAM109B | 0.1447 |
| ENSP00000307821 | FADS6 | 0.1447 |
| ENSP00000359772 | CT45A2 | 0.1447 |
| ENSP00000260988 | CRYGB | 0.1447 |
| ENSP00000272367 | CLEC4F | 0.1447 |
| ENSP00000329860 | CCDC172 | 0.1447 |
| ENSP00000440832 | CACFD1 | 0.1447 |
| ENSP00000349114 | C7orf76 | 0.1447 |
| ENSP00000331214 | C5orf50 | 0.1447 |
| ENSP00000195455 | C4orf6 | 0.1447 |
| ENSP00000333208 | C2orf82 | 0.1447 |
| ENSP00000315557 | C2orf57 | 0.1447 |
| ENSP00000302274 | C1QTNF4 | 0.1447 |
| ENSP00000401362 | C15orf41 | 0.1447 |
| ENSP00000353854 | C15orf39 | 0.1447 |
| ENSP00000261318 | C12orf49 | 0.1447 |
| ENSP00000311447 | ASPHD1 | 0.1447 |
| ENSP00000284142 | ASB17 | 0.1447 |
| ENSP00000301190 | ANKRD33 | 0.1447 |
| ENSP00000253109 | ANGPTL6 | 0.1447 |
| ENSP00000464976 | ZNF850 | 0.1421 |
| ENSP00000311957 | ZNF738 | 0.1421 |
| ENSP00000293725 | ZNF563 | 0.1421 |
| ENSP00000466051 | ZNF404 | 0.1421 |
| ENSP00000341528 | ZNF34 | 0.1421 |
| ENSP00000468720 | ZNF253 | 0.1421 |
| ENSP00000370447 | VCX | 0.1421 |
| ENSP00000351157 | TXNDC15 | 0.1421 |
| ENSP00000246801 | TSKS | 0.1421 |
| ENSP00000341031 | TPRG1 | 0.1421 |

| | | |
|---|---|---|
| ENSP00000264728 | TMEM40 | 0.1421 |
| ENSP00000254742 | TMEM128 | 0.1421 |
| ENSP00000357811 | TEX36 | 0.1421 |
| ENSP00000341652 | TBCCD1 | 0.1421 |
| ENSP00000349459 | SPINK14 | 0.1421 |
| ENSP00000275988 | SPIN2B | 0.1421 |
| ENSP00000244926 | SCGB1D2 | 0.1421 |
| ENSP00000344545 | SCGB1C1 | 0.1421 |
| ENSP00000293273 | RDM1 | 0.1421 |
| ENSP00000265732 | RBM48 | 0.1421 |
| ENSP00000332215 | PSG3 | 0.1421 |
| ENSP00000325638 | PRM3 | 0.1421 |
| ENSP00000359286 | PNMA3 | 0.1421 |
| ENSP00000462046 | PIRT | 0.1421 |
| ENSP00000350474 | OR8D1 | 0.1421 |
| ENSP00000328402 | OR6C76 | 0.1421 |
| ENSP00000325682 | OR2B11 | 0.1421 |
| ENSP00000343521 | OR1J4 | 0.1421 |
| ENSP00000437910 | MTRNR2L5 | 0.1421 |
| ENSP00000334922 | KRTAP9-4 | 0.1421 |
| ENSP00000332690 | KRTAP6-1 | 0.1421 |
| ENSP00000375428 | KRTAP3-3 | 0.1421 |
| ENSP00000357304 | KIAA0907 | 0.1421 |
| ENSP00000271417 | ILDR2 | 0.1421 |
| ENSP00000279168 | GPHA2 | 0.1421 |
| ENSP00000367172 | GKN1 | 0.1421 |
| ENSP00000295092 | FAM84A | 0.1421 |
| ENSP00000358454 | F8A3 | 0.1421 |
| ENSP00000201647 | EPS8L1 | 0.1421 |
| ENSP00000256039 | DYDC2 | 0.1421 |
| ENSP00000251250 | DTWD1 | 0.1421 |
| ENSP00000335538 | DEFB131 | 0.1421 |
| ENSP00000332723 | COLEC10 | 0.1421 |
| ENSP00000403400 | CCPG1 | 0.1421 |
| ENSP00000370658 | CCDC91 | 0.1421 |
| ENSP00000319006 | CCDC71 | 0.1421 |
| ENSP00000384416 | CCDC129 | 0.1421 |
| ENSP00000351967 | C9orf169 | 0.1421 |
| ENSP00000324741 | C7orf43 | 0.1421 |
| ENSP00000356951 | C1orf192 | 0.1421 |
| ENSP00000465432 | C19orf53 | 0.1421 |
| ENSP00000301246 | C19orf33 | 0.1421 |
| ENSP00000415662 | C17orf105 | 0.1421 |
| ENSP00000350075 | C15orf38 | 0.1421 |
| ENSP00000260723 | BTBD16 | 0.1421 |
| ENSP00000383407 | AF165138.7 | 0.1421 |
| ENSP00000350418 | ZNF98 | 0.1395 |
| ENSP00000405699 | ZNF837 | 0.1395 |
| ENSP00000471000 | ZNF616 | 0.1395 |
| ENSP00000351939 | ZNF574 | 0.1395 |
| ENSP00000223428 | ZNF510 | 0.1395 |
| ENSP00000364728 | XAGE1D | 0.1395 |
| ENSP00000368025 | WDR87 | 0.1395 |

| | | |
|---|---|---|
| ENSP00000416289 | WDR27 | 0.1395 |
| ENSP00000254816 | TRIM47 | 0.1395 |
| ENSP00000349296 | TMEM71 | 0.1395 |
| ENSP00000365176 | TMEM51 | 0.1395 |
| ENSP00000305533 | THAP9 | 0.1395 |
| ENSP00000385006 | THAP4 | 0.1395 |
| ENSP00000303777 | TEX13B | 0.1395 |
| ENSP00000264659 | SRCIN1 | 0.1395 |
| ENSP00000237201 | SPACA1 | 0.1395 |
| ENSP00000355428 | SH3BP5L | 0.1395 |
| ENSP00000424474 | SAMD14 | 0.1395 |
| ENSP00000457512 | RNF31 | 0.1395 |
| ENSP00000369341 | RFESD | 0.1395 |
| ENSP00000358532 | RBM20 | 0.1395 |
| ENSP00000384770 | PSG4 | 0.1395 |
| ENSP00000407653 | PRR22 | 0.1395 |
| ENSP00000386166 | PRR21 | 0.1395 |
| ENSP00000319590 | PRR18 | 0.1395 |
| ENSP00000350358 | PRAMEF12 | 0.1395 |
| ENSP00000298705 | PPP1R36 | 0.1395 |
| ENSP00000383394 | POM121L7 | 0.1395 |
| ENSP00000347933 | PLGLB1 | 0.1395 |
| ENSP00000330280 | OR8B8 | 0.1395 |
| ENSP00000322435 | OR6B3 | 0.1395 |
| ENSP00000432417 | OR5M11 | 0.1395 |
| ENSP00000373196 | OR5H6 | 0.1395 |
| ENSP00000308270 | OR5B3 | 0.1395 |
| ENSP00000322088 | OR52E2 | 0.1395 |
| ENSP00000322754 | OR51S1 | 0.1395 |
| ENSP00000311688 | OR1S1 | 0.1395 |
| ENSP00000381960 | OOSP1 | 0.1395 |
| ENSP00000357402 | NKAIN2 | 0.1395 |
| ENSP00000363513 | NCMAP | 0.1395 |
| ENSP00000338641 | MUM1L1 | 0.1395 |
| ENSP00000439985 | MTRNR2L7 | 0.1395 |
| ENSP00000361097 | MMRN2 | 0.1395 |
| ENSP00000311320 | LYG1 | 0.1395 |
| ENSP00000342711 | LY6H | 0.1395 |
| ENSP00000373191 | LNP1 | 0.1395 |
| ENSP00000377583 | KRTAP2-4 | 0.1395 |
| ENSP00000225550 | KRT37 | 0.1395 |
| ENSP00000392204 | KLLN | 0.1395 |
| ENSP00000298569 | KIAA1191 | 0.1395 |
| ENSP00000287275 | GLYATL2 | 0.1395 |
| ENSP00000315474 | GIMAP7 | 0.1395 |
| ENSP00000371127 | GAGE2C | 0.1395 |
| ENSP00000308137 | FOLR1 | 0.1395 |
| ENSP00000283946 | FBXO36 | 0.1395 |
| ENSP00000420140 | FAM71F2 | 0.1395 |
| ENSP00000298784 | FAM35A | 0.1395 |
| ENSP00000358518 | F8A2 | 0.1395 |
| ENSP00000270824 | EVA1B | 0.1395 |
| ENSP00000391594 | ERV3-1 | 0.1395 |

| | | |
|---|---|---|
| ENSP00000330375 | EPGN | 0.1395 |
| ENSP00000361001 | EFCAB14 | 0.1395 |
| ENSP00000338613 | CRYGN | 0.1395 |
| ENSP00000362968 | CLPSL1 | 0.1395 |
| ENSP00000281129 | CEP128 | 0.1395 |
| ENSP00000371787 | CEMP1 | 0.1395 |
| ENSP00000265334 | CDKL3 | 0.1395 |
| ENSP00000351727 | CCER1 | 0.1395 |
| ENSP00000304355 | CCDC126 | 0.1395 |
| ENSP00000292574 | CCDC105 | 0.1395 |
| ENSP00000288462 | C9orf43 | 0.1395 |
| ENSP00000380380 | C9orf38 | 0.1395 |
| ENSP00000326110 | C5orf30 | 0.1395 |
| ENSP00000399392 | C3orf52 | 0.1395 |
| ENSP00000335041 | C2orf76 | 0.1395 |
| ENSP00000311218 | C1orf100 | 0.1395 |
| ENSP00000322238 | C14orf119 | 0.1395 |
| ENSP00000348337 | C13orf35 | 0.1395 |
| ENSP00000347601 | C11orf92 | 0.1395 |
| ENSP00000346600 | C11orf48 | 0.1395 |
| ENSP00000358212 | C10orf82 | 0.1395 |
| ENSP00000395461 | BTBD19 | 0.1395 |
| ENSP00000470409 | AP003062.1 | 0.1395 |
| ENSP00000272972 | ANKMY1 | 0.1395 |
| ENSP00000286190 | ALS2CR12 | 0.1395 |
| ENSP00000358932 | ABHD16B | 0.1395 |
| ENSP00000406318 | ZNF880 | 0.1368 |
| ENSP00000399863 | ZNF839 | 0.1368 |
| ENSP00000269394 | ZNF750 | 0.1368 |
| ENSP00000268154 | ZNF710 | 0.1368 |
| ENSP00000457423 | ZNF625 | 0.1368 |
| ENSP00000302603 | ZNF556 | 0.1368 |
| ENSP00000470209 | ZNF257 | 0.1368 |
| ENSP00000447879 | ZBED6 | 0.1368 |
| ENSP00000367879 | XK | 0.1368 |
| ENSP00000357234 | TSACC | 0.1368 |
| ENSP00000369959 | TMEM239 | 0.1368 |
| ENSP00000364166 | TMEM225 | 0.1368 |
| ENSP00000386163 | TMEM14E | 0.1368 |
| ENSP00000340969 | TEX264 | 0.1368 |
| ENSP00000342538 | STMN4 | 0.1368 |
| ENSP00000360913 | SPATA6 | 0.1368 |
| ENSP00000342075 | SIMC1 | 0.1368 |
| ENSP00000364865 | RP11-65D24.2 | 0.1368 |
| ENSP00000364822 | RP11-410N8.4 | 0.1368 |
| ENSP00000263765 | PRDM11 | 0.1368 |
| ENSP00000328915 | PRAMEF7 | 0.1368 |
| ENSP00000294952 | PPP1R21 | 0.1368 |
| ENSP00000396445 | OSGIN2 | 0.1368 |
| ENSP00000330338 | OR52L1 | 0.1368 |
| ENSP00000369568 | OR51B6 | 0.1368 |
| ENSP00000305469 | OR1S2 | 0.1368 |
| ENSP00000248384 | OR1E2 | 0.1368 |

| | | |
|---|---|---|
| ENSP00000366365 | OR14J1 | 0.1368 |
| ENSP00000360214 | OPALIN | 0.1368 |
| ENSP00000329584 | ODF3L1 | 0.1368 |
| ENSP00000396080 | NUTM2D | 0.1368 |
| ENSP00000441365 | MARVELD1 | 0.1368 |
| ENSP00000333071 | LRRC37A2 | 0.1368 |
| ENSP00000327675 | LRRC14B | 0.1368 |
| ENSP00000358314 | LIX1L | 0.1368 |
| ENSP00000340988 | LDLRAD2 | 0.1368 |
| ENSP00000288873 | KRTCAP3 | 0.1368 |
| ENSP00000375109 | KRTAP13-3 | 0.1368 |
| ENSP00000310573 | KRT25 | 0.1368 |
| ENSP00000257765 | KHDC1 | 0.1368 |
| ENSP00000301678 | ITFG3 | 0.1368 |
| ENSP00000368405 | GDPGP1 | 0.1368 |
| ENSP00000355444 | GCSAML | 0.1368 |
| ENSP00000359827 | FAM73A | 0.1368 |
| ENSP00000305596 | FAM71B | 0.1368 |
| ENSP00000359333 | FAM69A | 0.1368 |
| ENSP00000310057 | FAM53A | 0.1368 |
| ENSP00000357586 | FAM26D | 0.1368 |
| ENSP00000392452 | FAM219A | 0.1368 |
| ENSP00000265018 | FAM184B | 0.1368 |
| ENSP00000386657 | FAM183B | 0.1368 |
| ENSP00000349175 | FAM180B | 0.1368 |
| ENSP00000396593 | DYTN | 0.1368 |
| ENSP00000312702 | DEFB114 | 0.1368 |
| ENSP00000465845 | CTB-102L5.4 | 0.1368 |
| ENSP00000349456 | CLCC1 | 0.1368 |
| ENSP00000402203 | CEACAM18 | 0.1368 |
| ENSP00000279249 | CDC42EP2 | 0.1368 |
| ENSP00000374292 | CCDC57 | 0.1368 |
| ENSP00000366173 | CBY3 | 0.1368 |
| ENSP00000335616 | C9orf47 | 0.1368 |
| ENSP00000364964 | C6orf25 | 0.1368 |
| ENSP00000371061 | C5orf51 | 0.1368 |
| ENSP00000337044 | C5orf24 | 0.1368 |
| ENSP00000413228 | C3orf55 | 0.1368 |
| ENSP00000381631 | C2orf73 | 0.1368 |
| ENSP00000419417 | C1orf52 | 0.1368 |
| ENSP00000345972 | C1orf168 | 0.1368 |
| ENSP00000396936 | C17orf72 | 0.1368 |
| ENSP00000340644 | C15orf43 | 0.1368 |
| ENSP00000321360 | C14orf177 | 0.1368 |
| ENSP00000308368 | C12orf76 | 0.1368 |
| ENSP00000341013 | AMTN | 0.1368 |
| ENSP00000414037 | AL592170.1 | 0.1368 |
| ENSP00000440385 | AL138764.1 | 0.1368 |
| ENSP00000469716 | AC090574.1 | 0.1368 |
| ENSP00000349853 | ENSG00000197511 | 0.1368 |
| ENSP00000443539 | BRWD1-IT2 | 0.1368 |
| ENSP00000414887 | ZNF879 | 0.1342 |
| ENSP00000457715 | ZNF865 | 0.1342 |

| | | |
|---|---|---|
| ENSP00000392024 | ZNF844 | 0.1342 |
| ENSP00000395733 | ZNF737 | 0.1342 |
| ENSP00000347043 | ZNF649 | 0.1342 |
| ENSP00000333660 | ZNF571 | 0.1342 |
| ENSP00000324056 | ZNF48 | 0.1342 |
| ENSP00000431202 | ZNF345 | 0.1342 |
| ENSP00000367595 | TPRG1L | 0.1342 |
| ENSP00000446264 | TMEM75 | 0.1342 |
| ENSP00000363714 | TMEM245 | 0.1342 |
| ENSP00000368737 | TMEM170B | 0.1342 |
| ENSP00000454404 | TMEM170A | 0.1342 |
| ENSP00000381204 | TMEM123 | 0.1342 |
| ENSP00000447731 | TMEM116 | 0.1342 |
| ENSP00000330877 | TEX40 | 0.1342 |
| ENSP00000323795 | TEX35 | 0.1342 |
| ENSP00000403925 | TBCEL | 0.1342 |
| ENSP00000258704 | SPDYE1 | 0.1342 |
| ENSP00000417147 | SMIM7 | 0.1342 |
| ENSP00000388220 | SGK110 | 0.1342 |
| ENSP00000411948 | SAPCD1 | 0.1342 |
| ENSP00000357701 | S100A3 | 0.1342 |
| ENSP00000342780 | RP11-113D6.10 | 0.1342 |
| ENSP00000356607 | RALGPS2 | 0.1342 |
| ENSP00000367894 | PRRG1 | 0.1342 |
| ENSP00000321242 | PRNT | 0.1342 |
| ENSP00000216446 | PLEK2 | 0.1342 |
| ENSP00000246081 | OTOR | 0.1342 |
| ENSP00000328090 | OTOP3 | 0.1342 |
| ENSP00000367650 | OR5L2 | 0.1342 |
| ENSP00000409094 | OR52I1 | 0.1342 |
| ENSP00000319654 | OR4M1 | 0.1342 |
| ENSP00000315047 | OR4F17 | 0.1342 |
| ENSP00000347965 | OR2T6 | 0.1342 |
| ENSP00000357132 | OR10X1 | 0.1342 |
| ENSP00000309907 | NBPF14 | 0.1342 |
| ENSP00000300187 | MS4A14 | 0.1342 |
| ENSP00000388137 | MPPED1 | 0.1342 |
| ENSP00000256186 | MICALCL | 0.1342 |
| ENSP00000360988 | LUZP4 | 0.1342 |
| ENSP00000418491 | LCN10 | 0.1342 |
| ENSP00000411198 | KRTAP4-16P | 0.1342 |
| ENSP00000380996 | KIAA1211L | 0.1342 |
| ENSP00000303928 | KIAA0232 | 0.1342 |
| ENSP00000370849 | ITPRIPL2 | 0.1342 |
| ENSP00000326500 | IL11RA | 0.1342 |
| ENSP00000311528 | GPR162 | 0.1342 |
| ENSP00000373363 | GOLM1 | 0.1342 |
| ENSP00000360240 | GLYATL3 | 0.1342 |
| ENSP00000371144 | GAGE2B | 0.1342 |
| ENSP00000346560 | FILIP1L | 0.1342 |
| ENSP00000334430 | FAM9C | 0.1342 |
| ENSP00000332615 | FAM169B | 0.1342 |
| ENSP00000451998 | EML5 | 0.1342 |

| | | |
|---|---|---|
| ENSP00000335481 | EMID1 | 0.1342 |
| ENSP00000014935 | DNASE1L1 | 0.1342 |
| ENSP00000359664 | CTBS | 0.1342 |
| ENSP00000250340 | CLEC11A | 0.1342 |
| ENSP00000006724 | CEACAM7 | 0.1342 |
| ENSP00000308873 | CCDC74B | 0.1342 |
| ENSP00000345470 | CCDC38 | 0.1342 |
| ENSP00000309836 | CCDC13 | 0.1342 |
| ENSP00000434593 | C8orf82 | 0.1342 |
| ENSP00000283905 | C7orf31 | 0.1342 |
| ENSP00000355735 | C6orf70 | 0.1342 |
| ENSP00000385467 | C22orf46 | 0.1342 |
| ENSP00000355840 | C1orf65 | 0.1342 |
| ENSP00000332162 | C1orf64 | 0.1342 |
| ENSP00000357511 | C1orf189 | 0.1342 |
| ENSP00000376103 | C19orf12 | 0.1342 |
| ENSP00000413728 | C12orf71 | 0.1342 |
| ENSP00000367353 | C12orf42 | 0.1342 |
| ENSP00000414687 | C11orf82 | 0.1342 |
| ENSP00000307879 | C11orf45 | 0.1342 |
| ENSP00000348751 | BTN3A2 | 0.1342 |
| ENSP00000430000 | ATXN7L3B | 0.1342 |
| ENSP00000457539 | APOBR | 0.1342 |
| ENSP00000384224 | AP000525.1 | 0.1342 |
| ENSP00000471799 | AL049747.1 | 0.1342 |
| ENSP00000360485 | AKAP14 | 0.1342 |
| ENSP00000396505 | AC142381.1 | 0.1342 |
| ENSP00000451382 | ENSG00000259141 | 0.1342 |
| ENSP00000410198 | ZSCAN2 | 0.1316 |
| ENSP00000351391 | ZNF781 | 0.1316 |
| ENSP00000291598 | ZNF583 | 0.1316 |
| ENSP00000261560 | ZNF430 | 0.1316 |
| ENSP00000375820 | ZNF233 | 0.1316 |
| ENSP00000251269 | ZNF221 | 0.1316 |
| ENSP00000363545 | ZDBF2 | 0.1316 |
| ENSP00000381436 | VGLL3 | 0.1316 |
| ENSP00000331298 | UBALD2 | 0.1316 |
| ENSP00000342570 | TREML4 | 0.1316 |
| ENSP00000361811 | TP53TG5 | 0.1316 |
| ENSP00000455099 | TMEM88B | 0.1316 |
| ENSP00000374234 | TMEM72 | 0.1316 |
| ENSP00000361095 | TMEM69 | 0.1316 |
| ENSP00000348463 | TMEM236 | 0.1316 |
| ENSP00000380929 | TCP10 | 0.1316 |
| ENSP00000366875 | SPATA31A1 | 0.1316 |
| ENSP00000358599 | SMIM11P1 | 0.1316 |
| ENSP00000216471 | SAMD15 | 0.1316 |
| ENSP00000456010 | RP11-831H9.16 | 0.1316 |
| ENSP00000375397 | RNF208 | 0.1316 |
| ENSP00000340369 | RINL | 0.1316 |
| ENSP00000237696 | RARRES1 | 0.1316 |
| ENSP00000447679 | PRR24 | 0.1316 |
| ENSP00000253008 | PRDM12 | 0.1316 |

| | | |
|---|---|---|
| ENSP00000332134 | PRAMEF1 | 0.1316 |
| ENSP00000364295 | PQLC2 | 0.1316 |
| ENSP00000426296 | PCP4L1 | 0.1316 |
| ENSP00000301529 | OR8J3 | 0.1316 |
| ENSP00000324958 | OR4C15 | 0.1316 |
| ENSP00000339726 | OR14I1 | 0.1316 |
| ENSP00000348033 | OR11L1 | 0.1316 |
| ENSP00000307130 | OR10H3 | 0.1316 |
| ENSP00000332511 | OLFML1 | 0.1316 |
| ENSP00000371722 | NRSN2 | 0.1316 |
| ENSP00000291971 | NLRP8 | 0.1316 |
| ENSP00000374408 | MROH2A | 0.1316 |
| ENSP00000374482 | MRGPRE | 0.1316 |
| ENSP00000314518 | LYSMD3 | 0.1316 |
| ENSP00000404432 | LIMS3L | 0.1316 |
| ENSP00000228799 | ITFG2 | 0.1316 |
| ENSP00000330374 | GIMAP6 | 0.1316 |
| ENSP00000386110 | GAGE2D | 0.1316 |
| ENSP00000416935 | FOLR4 | 0.1316 |
| ENSP00000370391 | FAM9A | 0.1316 |
| ENSP00000345029 | FAM47A | 0.1316 |
| ENSP00000351783 | FAM222A | 0.1316 |
| ENSP00000454591 | FAM188B2 | 0.1316 |
| ENSP00000307954 | FAM174A | 0.1316 |
| ENSP00000413196 | FAM160A1 | 0.1316 |
| ENSP00000384604 | FAM150B | 0.1316 |
| ENSP00000362975 | FAM13C | 0.1316 |
| ENSP00000442304 | FAM132B | 0.1316 |
| ENSP00000341597 | FAM114A2 | 0.1316 |
| ENSP00000472919 | ERVV-2 | 0.1316 |
| ENSP00000383396 | DEFB116 | 0.1316 |
| ENSP00000381703 | DEFB113 | 0.1316 |
| ENSP00000247306 | CTAG2 | 0.1316 |
| ENSP00000394183 | CDKN2AIPNL | 0.1316 |
| ENSP00000364501 | CD300LD | 0.1316 |
| ENSP00000353462 | CASS4 | 0.1316 |
| ENSP00000299957 | CASC4 | 0.1316 |
| ENSP00000288502 | C9orf91 | 0.1316 |
| ENSP00000381937 | C4orf29 | 0.1316 |
| ENSP00000264434 | C2orf42 | 0.1316 |
| ENSP00000253461 | C16orf95 | 0.1316 |
| ENSP00000452773 | C15orf57 | 0.1316 |
| ENSP00000320849 | C12orf68 | 0.1316 |
| ENSP00000352992 | ART5 | 0.1316 |
| ENSP00000387907 | ANKUB1 | 0.1316 |
| ENSP00000323096 | AMIGO3 | 0.1316 |
| ENSP00000393631 | ADM5 | 0.1316 |
| ENSP00000383515 | AC002321.1 | 0.1316 |
| ENSP00000300811 | ZNF428 | 0.1289 |
| ENSP00000340132 | ZNF383 | 0.1289 |
| ENSP00000337466 | WFDC10B | 0.1289 |
| ENSP00000403438 | TMEM150C | 0.1289 |
| ENSP00000347195 | TMEM110 | 0.1289 |

| | | |
|---|---|---|
| ENSP00000378289 | SYNPO2L | 0.1289 |
| ENSP00000363388 | SUSD1 | 0.1289 |
| ENSP00000359707 | SAMD13 | 0.1289 |
| ENSP00000326253 | RTBDN | 0.1289 |
| ENSP00000457654 | RP11-77K12.1 | 0.1289 |
| ENSP00000402914 | RP11-685N3.1 | 0.1289 |
| ENSP00000387046 | PPM1M | 0.1289 |
| ENSP00000379183 | PANX2 | 0.1289 |
| ENSP00000436004 | OR5M10 | 0.1289 |
| ENSP00000331572 | OR56A3 | 0.1289 |
| ENSP00000321246 | OR56A1 | 0.1289 |
| ENSP00000369729 | OR51A2 | 0.1289 |
| ENSP00000324831 | OR4P4 | 0.1289 |
| ENSP00000319322 | OR4K13 | 0.1289 |
| ENSP00000326349 | OR2G2 | 0.1289 |
| ENSP00000386209 | OR2A2 | 0.1289 |
| ENSP00000340748 | OR13H1 | 0.1289 |
| ENSP00000334115 | OR10T2 | 0.1289 |
| ENSP00000331789 | OR10J3 | 0.1289 |
| ENSP00000302437 | OR10G3 | 0.1289 |
| ENSP00000407521 | NUTM2E | 0.1289 |
| ENSP00000333627 | NKAIN3 | 0.1289 |
| ENSP00000334998 | MT1B | 0.1289 |
| ENSP00000344551 | MORN2 | 0.1289 |
| ENSP00000468007 | LUZP6 | 0.1289 |
| ENSP00000359204 | LPPR4 | 0.1289 |
| ENSP00000287585 | LHFPL4 | 0.1289 |
| ENSP00000470257 | hsa-mir-150 | 0.1289 |
| ENSP00000305193 | HS1BP3 | 0.1289 |
| ENSP00000429367 | FAM92A1 | 0.1289 |
| ENSP00000304078 | FAM83B | 0.1289 |
| ENSP00000283357 | FAM81B | 0.1289 |
| ENSP00000333394 | FAM104B | 0.1289 |
| ENSP00000458866 | CTB-133G6.1 | 0.1289 |
| ENSP00000377470 | CNP | 0.1289 |
| ENSP00000358491 | CMC4 | 0.1289 |
| ENSP00000466140 | CGB1 | 0.1289 |
| ENSP00000320232 | CCDC168 | 0.1289 |
| ENSP00000402239 | CCDC159 | 0.1289 |
| ENSP00000273936 | CABS1 | 0.1289 |
| ENSP00000364613 | C9orf89 | 0.1289 |
| ENSP00000216071 | C22orf31 | 0.1289 |
| ENSP00000362001 | C20orf62 | 0.1289 |
| ENSP00000371572 | C1QTNF9B | 0.1289 |
| ENSP00000371497 | C1QTNF3 | 0.1289 |
| ENSP00000361603 | C1orf50 | 0.1289 |
| ENSP00000380960 | C17orf67 | 0.1289 |
| ENSP00000300575 | C16orf92 | 0.1289 |
| ENSP00000325144 | C15orf53 | 0.1289 |
| ENSP00000364515 | BTBD17 | 0.1289 |
| ENSP00000471539 | AC091150.1 | 0.1289 |
| ENSP00000320096 | ZNF784 | 0.1263 |
| ENSP00000346348 | ZNF774 | 0.1263 |

| | | |
|---|---|---|
| ENSP00000371789 | ZCCHC3 | 0.1263 |
| ENSP00000374037 | VPS9D1 | 0.1263 |
| ENSP00000250825 | VCY | 0.1263 |
| ENSP00000386126 | UMODL1 | 0.1263 |
| ENSP00000348996 | TSGA13 | 0.1263 |
| ENSP00000411645 | TMPRSS7 | 0.1263 |
| ENSP00000331466 | TMEM95 | 0.1263 |
| ENSP00000380646 | TMEM80 | 0.1263 |
| ENSP00000364041 | SPIN2A | 0.1263 |
| ENSP00000274432 | SPATA9 | 0.1263 |
| ENSP00000349132 | SPATA31A3 | 0.1263 |
| ENSP00000275227 | SLC18B1 | 0.1263 |
| ENSP00000312550 | SEZ6L2 | 0.1263 |
| ENSP00000463832 | RP11-41O4.1 | 0.1263 |
| ENSP00000327168 | PRR7 | 0.1263 |
| ENSP00000368199 | PGPEP1L | 0.1263 |
| ENSP00000384801 | PCNXL4 | 0.1263 |
| ENSP00000284287 | OR8A1 | 0.1263 |
| ENSP00000347418 | OR5H2 | 0.1263 |
| ENSP00000329056 | OR4C46 | 0.1263 |
| ENSP00000324913 | OR4C16 | 0.1263 |
| ENSP00000332185 | OR2V2 | 0.1263 |
| ENSP00000339256 | NIPAL2 | 0.1263 |
| ENSP00000289749 | NBL1 | 0.1263 |
| ENSP00000375904 | LGALS16 | 0.1263 |
| ENSP00000364764 | LAYN | 0.1263 |
| ENSP00000293525 | KRT86 | 0.1263 |
| ENSP00000370568 | KIAA1644 | 0.1263 |
| ENSP00000243213 | IL13RA2 | 0.1263 |
| ENSP00000251296 | IGSF21 | 0.1263 |
| ENSP00000366926 | IGFL4 | 0.1263 |
| ENSP00000344403 | FSIP2 | 0.1263 |
| ENSP00000264669 | FASTKD3 | 0.1263 |
| ENSP00000339681 | FAM78B | 0.1263 |
| ENSP00000431905 | FAM71D | 0.1263 |
| ENSP00000351345 | FAM150A | 0.1263 |
| ENSP00000181796 | FAM107B | 0.1263 |
| ENSP00000274217 | FAM105A | 0.1263 |
| ENSP00000384832 | FAM104A | 0.1263 |
| ENSP00000383398 | DEFB115 | 0.1263 |
| ENSP00000417674 | CYLC2 | 0.1263 |
| ENSP00000331556 | CYLC1 | 0.1263 |
| ENSP00000221954 | CEACAM4 | 0.1263 |
| ENSP00000224756 | CCSER2 | 0.1263 |
| ENSP00000320649 | CCDC89 | 0.1263 |
| ENSP00000258776 | C7orf69 | 0.1263 |
| ENSP00000391404 | C4orf51 | 0.1263 |
| ENSP00000445446 | C3orf33 | 0.1263 |
| ENSP00000221671 | C19orf44 | 0.1263 |
| ENSP00000386218 | C16orf3 | 0.1263 |
| ENSP00000328423 | C15orf60 | 0.1263 |
| ENSP00000381115 | C11orf93 | 0.1263 |
| ENSP00000376767 | C11orf57 | 0.1263 |

| | | |
|---|---|---|
| ENSP00000321464 | C10orf67 | 0.1263 |
| ENSP00000363226 | C10orf53 | 0.1263 |
| ENSP00000361776 | BEX4 | 0.1263 |
| ENSP00000315357 | ARL6IP6 | 0.1263 |
| ENSP00000376705 | ANO4 | 0.1263 |
| ENSP00000416897 | AC008394.1 | 0.1263 |
| ENSP00000407425 | SKA2L | 0.1263 |
| ENSP00000468098 | SNHG16 | 0.1263 |
| ENSP00000384000 | ZNF681 | 0.1237 |
| ENSP00000386845 | ZNF385B | 0.1237 |
| ENSP00000310856 | VN1R4 | 0.1237 |
| ENSP00000167106 | VASH1 | 0.1237 |
| ENSP00000373066 | TXNRD3NB | 0.1237 |
| ENSP00000355649 | SPHAR | 0.1237 |
| ENSP00000451866 | SMIM13 | 0.1237 |
| ENSP00000391101 | SLFN14 | 0.1237 |
| ENSP00000462754 | SLC25A52 | 0.1237 |
| ENSP00000365503 | SHISA7 | 0.1237 |
| ENSP00000457849 | RP11-613M10.8 | 0.1237 |
| ENSP00000455681 | RP11-48B14.2 | 0.1237 |
| ENSP00000467209 | RP11-125O5.2 | 0.1237 |
| ENSP00000234195 | RMDN2 | 0.1237 |
| ENSP00000379154 | RASGEF1A | 0.1237 |
| ENSP00000259845 | PSORS1C2 | 0.1237 |
| ENSP00000438757 | PRR20C | 0.1237 |
| ENSP00000309087 | PLAC8L1 | 0.1237 |
| ENSP00000307159 | OR8B12 | 0.1237 |
| ENSP00000303076 | OR5B2 | 0.1237 |
| ENSP00000369573 | OR51B4 | 0.1237 |
| ENSP00000332110 | OR4N5 | 0.1237 |
| ENSP00000313803 | OR3A1 | 0.1237 |
| ENSP00000355443 | OR2C3 | 0.1237 |
| ENSP00000386175 | OR2A1 | 0.1237 |
| ENSP00000363911 | OR13C5 | 0.1237 |
| ENSP00000419718 | OFCC1 | 0.1237 |
| ENSP00000365014 | NXNL2 | 0.1237 |
| ENSP00000368396 | LYRM9 | 0.1237 |
| ENSP00000361518 | LRRC73 | 0.1237 |
| ENSP00000326324 | LRRC37A | 0.1237 |
| ENSP00000375482 | KRTAP6-3 | 0.1237 |
| ENSP00000334798 | KRT26 | 0.1237 |
| ENSP00000383630 | KIR3DL2 | 0.1237 |
| ENSP00000359219 | KAZALD1 | 0.1237 |
| ENSP00000274766 | KAAG1 | 0.1237 |
| ENSP00000329904 | IQCF2 | 0.1237 |
| ENSP00000248089 | HCFC1R1 | 0.1237 |
| ENSP00000427428 | FAM218A | 0.1237 |
| ENSP00000349902 | FAM115C | 0.1237 |
| ENSP00000363386 | FAM110D | 0.1237 |
| ENSP00000366170 | CST9 | 0.1237 |
| ENSP00000293925 | CRAMP1L | 0.1237 |
| ENSP00000430073 | CNBD1 | 0.1237 |
| ENSP00000432172 | CCDC17 | 0.1237 |

| | | |
|---|---|---|
| ENSP00000362392 | C9orf117 | 0.1237 |
| ENSP00000328069 | C6orf58 | 0.1237 |
| ENSP00000355787 | C6orf123 | 0.1237 |
| ENSP00000316457 | C20orf197 | 0.1237 |
| ENSP00000386203 | C1orf229 | 0.1237 |
| ENSP00000252453 | C19orf80 | 0.1237 |
| ENSP00000398350 | C11orf68 | 0.1237 |
| ENSP00000359018 | AKNAD1 | 0.1237 |
| ENSP00000391536 | AC021860.1 | 0.1237 |
| ENSP00000268271 | SNX29 | 0.1237 |
| ENSP00000437878 | ZNF595 | 0.1211 |
| ENSP00000413660 | ZNF492 | 0.1211 |
| ENSP00000361871 | WFDC12 | 0.1211 |
| ENSP00000378138 | TMEM98 | 0.1211 |
| ENSP00000261556 | TMEM260 | 0.1211 |
| ENSP00000356057 | TMEM181 | 0.1211 |
| ENSP00000365776 | TMC4 | 0.1211 |
| ENSP00000283547 | TEX29 | 0.1211 |
| ENSP00000334879 | SOWAHB | 0.1211 |
| ENSP00000381895 | SMCO3 | 0.1211 |
| ENSP00000289877 | SLC45A1 | 0.1211 |
| ENSP00000357072 | SLAMF9 | 0.1211 |
| ENSP00000325776 | SERTM1 | 0.1211 |
| ENSP00000450085 | RNASEK-C17orf49 | 0.1211 |
| ENSP00000354109 | RGS22 | 0.1211 |
| ENSP00000297164 | RELL2 | 0.1211 |
| ENSP00000356828 | RCSD1 | 0.1211 |
| ENSP00000293922 | PTX4 | 0.1211 |
| ENSP00000471817 | PRED62 | 0.1211 |
| ENSP00000218230 | PCSK1N | 0.1211 |
| ENSP00000470965 | PCDP1 | 0.1211 |
| ENSP00000332528 | OTOP2 | 0.1211 |
| ENSP00000386160 | OR52B4 | 0.1211 |
| ENSP00000325128 | OR4A16 | 0.1211 |
| ENSP00000291231 | OR3A3 | 0.1211 |
| ENSP00000366336 | OR2H1 | 0.1211 |
| ENSP00000317357 | OR13D1 | 0.1211 |
| ENSP00000363913 | OR13C3 | 0.1211 |
| ENSP00000324251 | OR10K2 | 0.1211 |
| ENSP00000364164 | OR10G9 | 0.1211 |
| ENSP00000460371 | OCLM | 0.1211 |
| ENSP00000455442 | NUPR1L | 0.1211 |
| ENSP00000364702 | NBPF9 | 0.1211 |
| ENSP00000299432 | MSS51 | 0.1211 |
| ENSP00000378506 | MSANTD3 | 0.1211 |
| ENSP00000300190 | MS4A5 | 0.1211 |
| ENSP00000470473 | MROH5 | 0.1211 |
| ENSP00000307513 | MRC2 | 0.1211 |
| ENSP00000344071 | METTL24 | 0.1211 |
| ENSP00000299952 | MARVELD3 | 0.1211 |
| ENSP00000318185 | LRRC42 | 0.1211 |
| ENSP00000371220 | LOH12CR2 | 0.1211 |
| ENSP00000302297 | LGI3 | 0.1211 |

| | | |
|---|---|---|
| ENSP00000392189 | KRTAP9-3 | 0.1211 |
| ENSP00000349154 | KIAA1841 | 0.1211 |
| ENSP00000264229 | KIAA1211 | 0.1211 |
| ENSP00000454268 | KIAA1024L | 0.1211 |
| ENSP00000300961 | JSRP1 | 0.1211 |
| ENSP00000327344 | IGIP | 0.1211 |
| ENSP00000337014 | HFE2 | 0.1211 |
| ENSP00000406164 | GMNC | 0.1211 |
| ENSP00000259989 | FGFBP2 | 0.1211 |
| ENSP00000367193 | FBXO48 | 0.1211 |
| ENSP00000356096 | FAM72A | 0.1211 |
| ENSP00000342576 | FAM221A | 0.1211 |
| ENSP00000417496 | FAM19A1 | 0.1211 |
| ENSP00000373726 | FAM159B | 0.1211 |
| ENSP00000422293 | FAM153C | 0.1211 |
| ENSP00000272342 | ETAA1 | 0.1211 |
| ENSP00000332806 | EMILIN3 | 0.1211 |
| ENSP00000401597 | DTHD1 | 0.1211 |
| ENSP00000372126 | DEFA1 | 0.1211 |
| ENSP00000462745 | DCAF8L2 | 0.1211 |
| ENSP00000406145 | CTXN2 | 0.1211 |
| ENSP00000379047 | CCDC51 | 0.1211 |
| ENSP00000383456 | C9orf152 | 0.1211 |
| ENSP00000259983 | C6orf52 | 0.1211 |
| ENSP00000365076 | C6orf47 | 0.1211 |
| ENSP00000380270 | C5orf28 | 0.1211 |
| ENSP00000281146 | C4orf33 | 0.1211 |
| ENSP00000424737 | C4orf21 | 0.1211 |
| ENSP00000215582 | C19orf21 | 0.1211 |
| ENSP00000465517 | C18orf21 | 0.1211 |
| ENSP00000351248 | C16orf47 | 0.1211 |
| ENSP00000348145 | C14orf178 | 0.1211 |
| ENSP00000340672 | ARMCX3 | 0.1211 |
| ENSP00000308149 | AMZ1 | 0.1211 |
| ENSP00000386846 | AC106876.2 | 0.1211 |
| ENSP00000366356 | OR5V1 | 0.1211 |
| ENSP00000420093 | NG38 | 0.1211 |
| ENSP00000293471 | ZNF613 | 0.1184 |
| ENSP00000354694 | ZNF485 | 0.1184 |
| ENSP00000382511 | ZNF195 | 0.1184 |
| ENSP00000326967 | ZNF121 | 0.1184 |
| ENSP00000305065 | ZBBX | 0.1184 |
| ENSP00000364766 | XAGE1B | 0.1184 |
| ENSP00000302938 | WFDC13 | 0.1184 |
| ENSP00000369798 | UCN3 | 0.1184 |
| ENSP00000451229 | TMEM253 | 0.1184 |
| ENSP00000388684 | TMEM247 | 0.1184 |
| ENSP00000372720 | TMEM241 | 0.1184 |
| ENSP00000395244 | TMEM229A | 0.1184 |
| ENSP00000346981 | TMEM207 | 0.1184 |
| ENSP00000397843 | TMA7 | 0.1184 |
| ENSP00000374014 | TM6SF2 | 0.1184 |
| ENSP00000326841 | SPATS2 | 0.1184 |

| | | |
|---|---|---|
| ENSP00000319796 | SERINC4 | 0.1184 |
| ENSP00000296506 | SCRG1 | 0.1184 |
| ENSP00000318032 | RPRML | 0.1184 |
| ENSP00000338288 | RNASE11 | 0.1184 |
| ENSP00000455434 | RD3L | 0.1184 |
| ENSP00000321179 | RBM44 | 0.1184 |
| ENSP00000277570 | PROSER2 | 0.1184 |
| ENSP00000332034 | PROSER1 | 0.1184 |
| ENSP00000274853 | PPP1R18 | 0.1184 |
| ENSP00000350022 | OR8D2 | 0.1184 |
| ENSP00000334721 | OR6P1 | 0.1184 |
| ENSP00000329153 | OR6C70 | 0.1184 |
| ENSP00000393889 | OR5K2 | 0.1184 |
| ENSP00000322866 | OR52N5 | 0.1184 |
| ENSP00000322724 | OR51H1P | 0.1184 |
| ENSP00000300773 | OR51B5 | 0.1184 |
| ENSP00000373136 | OR2H2 | 0.1184 |
| ENSP00000355436 | OR2AK2 | 0.1184 |
| ENSP00000360841 | OBP2A | 0.1184 |
| ENSP00000455906 | MUC22 | 0.1184 |
| ENSP00000442159 | MTRNR2L10 | 0.1184 |
| ENSP00000339971 | MROH8 | 0.1184 |
| ENSP00000342840 | LYSMD4 | 0.1184 |
| ENSP00000239367 | LRP11 | 0.1184 |
| ENSP00000219837 | KNOP1 | 0.1184 |
| ENSP00000280020 | KIAA1328 | 0.1184 |
| ENSP00000265068 | KIAA1257 | 0.1184 |
| ENSP00000297063 | KIAA0895 | 0.1184 |
| ENSP00000307951 | HIGD2B | 0.1184 |
| ENSP00000381473 | HIGD1C | 0.1184 |
| ENSP00000311657 | GRAMD2 | 0.1184 |
| ENSP00000361124 | GNG5P2 | 0.1184 |
| ENSP00000365426 | GGACT | 0.1184 |
| ENSP00000371117 | GAGE12E | 0.1184 |
| ENSP00000367319 | FBXO47 | 0.1184 |
| ENSP00000045083 | FAM65C | 0.1184 |
| ENSP00000351040 | FAM217B | 0.1184 |
| ENSP00000419502 | FAM211A | 0.1184 |
| ENSP00000409009 | FAM195B | 0.1184 |
| ENSP00000329137 | FAM132A | 0.1184 |
| ENSP00000364561 | FAM120AOS | 0.1184 |
| ENSP00000315265 | DYNAP | 0.1184 |
| ENSP00000294485 | DRAXIN | 0.1184 |
| ENSP00000402151 | CTC-203F4.1 | 0.1184 |
| ENSP00000450461 | CEP95 | 0.1184 |
| ENSP00000382000 | CDRT15L2 | 0.1184 |
| ENSP00000359845 | CCDC160 | 0.1184 |
| ENSP00000290418 | CCDC142 | 0.1184 |
| ENSP00000346345 | C9orf163 | 0.1184 |
| ENSP00000359505 | C6orf57 | 0.1184 |
| ENSP00000415517 | C6orf10 | 0.1184 |
| ENSP00000404583 | C5orf60 | 0.1184 |
| ENSP00000303490 | C5orf34 | 0.1184 |

| | | |
|---|---|---|
| ENSP00000253407 | C1QL1 | 0.1184 |
| ENSP00000429399 | C1orf210 | 0.1184 |
| ENSP00000322609 | C1orf173 | 0.1184 |
| ENSP00000331363 | C19orf68 | 0.1184 |
| ENSP00000373594 | C15orf27 | 0.1184 |
| ENSP00000280325 | C11orf53 | 0.1184 |
| ENSP00000419126 | C10orf88 | 0.1184 |
| ENSP00000358067 | C10orf85 | 0.1184 |
| ENSP00000371724 | BPY2C | 0.1184 |
| ENSP00000471635 | ARHGEF18 | 0.1184 |
| ENSP00000258749 | AOAH | 0.1184 |
| ENSP00000455075 | AC114783.1 | 0.1184 |
| ENSP00000389658 | AC079341.1 | 0.1184 |
| ENSP00000472293 | AC026407.1 | 0.1184 |
| ENSP00000247706 | ABHD8 | 0.1184 |
| ENSP00000341274 | AAK1 | 0.1184 |
| ENSP00000246000 | FAM182A | 0.1184 |
| ENSP00000366908 | FAM201A | 0.1184 |
| ENSP00000307801 | ZPLD1 | 0.1158 |
| ENSP00000429266 | ZNF829 | 0.1158 |
| ENSP00000333980 | ZNF749 | 0.1158 |
| ENSP00000467889 | ZNF607 | 0.1158 |
| ENSP00000337555 | ZNF548 | 0.1158 |
| ENSP00000379464 | ZNF134 | 0.1158 |
| ENSP00000349593 | ZHX1-C8ORF76 | 0.1158 |
| ENSP00000286049 | XAGE2B | 0.1158 |
| ENSP00000364013 | TMEFF1 | 0.1158 |
| ENSP00000215917 | SRRD | 0.1158 |
| ENSP00000359534 | SPANXN3 | 0.1158 |
| ENSP00000226460 | SMR3A | 0.1158 |
| ENSP00000304401 | SLFNL1 | 0.1158 |
| ENSP00000273173 | SLC22A14 | 0.1158 |
| ENSP00000401535 | RP1-130H16.18 | 0.1158 |
| ENSP00000251776 | ROPN1B | 0.1158 |
| ENSP00000219301 | PRSS54 | 0.1158 |
| ENSP00000321691 | PDDC1 | 0.1158 |
| ENSP00000386653 | PCDH20 | 0.1158 |
| ENSP00000398729 | PAPPA-AS1 | 0.1158 |
| ENSP00000344101 | OR6N2 | 0.1158 |
| ENSP00000373193 | OR5K1 | 0.1158 |
| ENSP00000278409 | OR5F1 | 0.1158 |
| ENSP00000322784 | OR5AK2 | 0.1158 |
| ENSP00000322823 | OR52N1 | 0.1158 |
| ENSP00000333684 | OR52A1 | 0.1158 |
| ENSP00000321729 | OR51V1 | 0.1158 |
| ENSP00000323952 | OR51F2 | 0.1158 |
| ENSP00000386195 | OR4E2 | 0.1158 |
| ENSP00000302863 | OR1L3 | 0.1158 |
| ENSP00000205194 | NAT14 | 0.1158 |
| ENSP00000462879 | LYRM9 | 0.1158 |
| ENSP00000296877 | LEAP2 | 0.1158 |
| ENSP00000360696 | LCN12 | 0.1158 |
| ENSP00000255992 | KRBA1 | 0.1158 |

| | | |
|---|---|---|
| ENSP00000379607 | KNCN | 0.1158 |
| ENSP00000242109 | KIAA0087 | 0.1158 |
| ENSP00000412060 | GREB1L | 0.1158 |
| ENSP00000402962 | FKSG52 | 0.1158 |
| ENSP00000343279 | FAM83G | 0.1158 |
| ENSP00000344331 | FAM69C | 0.1158 |
| ENSP00000350260 | FAM219B | 0.1158 |
| ENSP00000424007 | CTC-534A2.2 | 0.1158 |
| ENSP00000341128 | CLUL1 | 0.1158 |
| ENSP00000324767 | CHDC2 | 0.1158 |
| ENSP00000354730 | CHAMP1 | 0.1158 |
| ENSP00000005180 | CCL26 | 0.1158 |
| ENSP00000432622 | CCDC163P | 0.1158 |
| ENSP00000358718 | CCDC147 | 0.1158 |
| ENSP00000328054 | CCDC144NL | 0.1158 |
| ENSP00000371824 | C9orf66 | 0.1158 |
| ENSP00000395281 | C9orf116 | 0.1158 |
| ENSP00000428230 | C8orf17 | 0.1158 |
| ENSP00000292586 | C5orf45 | 0.1158 |
| ENSP00000357226 | C1orf61 | 0.1158 |
| ENSP00000380116 | C19orf55 | 0.1158 |
| ENSP00000384286 | C17orf51 | 0.1158 |
| ENSP00000369150 | C15orf59 | 0.1158 |
| ENSP00000450685 | C14orf64 | 0.1158 |
| ENSP00000318999 | C11orf16 | 0.1158 |
| ENSP00000376992 | BCL2L15 | 0.1158 |
| ENSP00000470770 | AC004466.1 | 0.1158 |
| ENSP00000359055 | NBPF4 | 0.1158 |
| ENSP00000389841 | ZNF852 | 0.1132 |
| ENSP00000310472 | ZNF624 | 0.1132 |
| ENSP00000333595 | ZNF285 | 0.1132 |
| ENSP00000389543 | TSRM | 0.1132 |
| ENSP00000363980 | TMEM246 | 0.1132 |
| ENSP00000338164 | TMEM217 | 0.1132 |
| ENSP00000333697 | TMEM179B | 0.1132 |
| ENSP00000264771 | TMEM175 | 0.1132 |
| ENSP00000361852 | TMCO2 | 0.1132 |
| ENSP00000456635 | TEX38 | 0.1132 |
| ENSP00000357465 | TDRD10 | 0.1132 |
| ENSP00000456026 | SMLR1 | 0.1132 |
| ENSP00000322832 | ROGDI | 0.1132 |
| ENSP00000332530 | RNLS | 0.1132 |
| ENSP00000334153 | RFTN1 | 0.1132 |
| ENSP00000385706 | PSG2 | 0.1132 |
| ENSP00000359378 | PRRG3 | 0.1132 |
| ENSP00000414034 | PRR26 | 0.1132 |
| ENSP00000237353 | PMFBP1 | 0.1132 |
| ENSP00000351211 | OR6C6 | 0.1132 |
| ENSP00000368989 | OR6C3 | 0.1132 |
| ENSP00000435416 | OR5M1 | 0.1132 |
| ENSP00000432011 | OR52B2 | 0.1132 |
| ENSP00000320560 | OR2D3 | 0.1132 |
| ENSP00000367219 | OR13J1 | 0.1132 |

| | | |
|---|---|---|
| ENSP00000455068 | METRN | 0.1132 |
| ENSP00000368964 | MAP7D2 | 0.1132 |
| ENSP00000348936 | LEKR1 | 0.1132 |
| ENSP00000367882 | LANCL3 | 0.1132 |
| ENSP00000375147 | KRTAP16-1 | 0.1132 |
| ENSP00000251643 | KRT12 | 0.1132 |
| ENSP00000346283 | HRCT1 | 0.1132 |
| ENSP00000350402 | HHLA2 | 0.1132 |
| ENSP00000337854 | HEXDC | 0.1132 |
| ENSP00000270590 | GPR32 | 0.1132 |
| ENSP00000354645 | GPATCH3 | 0.1132 |
| ENSP00000343636 | GFRAL | 0.1132 |
| ENSP00000359034 | FNDC7 | 0.1132 |
| ENSP00000295150 | FAM228A | 0.1132 |
| ENSP00000343115 | FAM222B | 0.1132 |
| ENSP00000411372 | FAM200A | 0.1132 |
| ENSP00000227065 | FAM149A | 0.1132 |
| ENSP00000473153 | ERVV-1 | 0.1132 |
| ENSP00000456548 | ELFN1 | 0.1132 |
| ENSP00000378312 | EFCAB5 | 0.1132 |
| ENSP00000359777 | CT45A1 | 0.1132 |
| ENSP00000357660 | CLRN3 | 0.1132 |
| ENSP00000393719 | CLEC17A | 0.1132 |
| ENSP00000328336 | CEND1 | 0.1132 |
| ENSP00000293889 | CCDC78 | 0.1132 |
| ENSP00000277657 | CCDC7 | 0.1132 |
| ENSP00000391504 | CCDC173 | 0.1132 |
| ENSP00000373828 | CCDC154 | 0.1132 |
| ENSP00000366050 | C9orf40 | 0.1132 |
| ENSP00000391218 | C9orf173 | 0.1132 |
| ENSP00000293604 | C6orf136 | 0.1132 |
| ENSP00000264387 | C2orf83 | 0.1132 |
| ENSP00000369986 | C21orf88 | 0.1132 |
| ENSP00000356363 | C1orf53 | 0.1132 |
| ENSP00000235307 | C1orf21 | 0.1132 |
| ENSP00000390224 | C1orf132 | 0.1132 |
| ENSP00000215531 | C19orf77 | 0.1132 |
| ENSP00000401335 | C16orf90 | 0.1132 |
| ENSP00000299320 | C16orf71 | 0.1132 |
| ENSP00000324920 | C14orf39 | 0.1132 |
| ENSP00000329698 | C12orf56 | 0.1132 |
| ENSP00000406541 | C11orf21 | 0.1132 |
| ENSP00000356590 | AXDND1 | 0.1132 |
| ENSP00000390941 | ATCAY | 0.1132 |
| ENSP00000373860 | ASPDH | 0.1132 |
| ENSP00000409950 | ANHX | 0.1132 |
| ENSP00000403980 | AC002365.1 | 0.1132 |
| ENSP00000412272 | ZNF503-AS2 | 0.1132 |
| ENSP00000315870 | ZNF575 | 0.1105 |
| ENSP00000354734 | WISP3 | 0.1105 |
| ENSP00000266673 | TMEM19 | 0.1105 |
| ENSP00000343799 | RNF222 | 0.1105 |
| ENSP00000398342 | PPP1R14D | 0.1105 |

| | | |
|---|---|---|
| ENSP00000309233 | POMZP3 | 0.1105 |
| ENSP00000392726 | POM121L2 | 0.1105 |
| ENSP00000317818 | PIANP | 0.1105 |
| ENSP00000380872 | PGBD4 | 0.1105 |
| ENSP00000354800 | OR5D13 | 0.1105 |
| ENSP00000349945 | OR5B17 | 0.1105 |
| ENSP00000303834 | OR5A2 | 0.1105 |
| ENSP00000321426 | OR52E4 | 0.1105 |
| ENSP00000333184 | OR4F15 | 0.1105 |
| ENSP00000341291 | OR2G6 | 0.1105 |
| ENSP00000401966 | OR1M1 | 0.1105 |
| ENSP00000459028 | OR1D5 | 0.1105 |
| ENSP00000357134 | OR10R2 | 0.1105 |
| ENSP00000310704 | OR10H5 | 0.1105 |
| ENSP00000311477 | OR10AG1 | 0.1105 |
| ENSP00000364627 | NXPE4 | 0.1105 |
| ENSP00000350377 | MCTP2 | 0.1105 |
| ENSP00000375234 | KRTAP4-9 | 0.1105 |
| ENSP00000333993 | KRTAP17-1 | 0.1105 |
| ENSP00000301656 | KRT27 | 0.1105 |
| ENSP00000431179 | KIAA0754 | 0.1105 |
| ENSP00000360829 | GLT6D1 | 0.1105 |
| ENSP00000318437 | FDCSP | 0.1105 |
| ENSP00000422769 | DNAH10OS | 0.1105 |
| ENSP00000330509 | DEXI | 0.1105 |
| ENSP00000361650 | CRIP3 | 0.1105 |
| ENSP00000325301 | CDPF1 | 0.1105 |
| ENSP00000377577 | CCDC176 | 0.1105 |
| ENSP00000259229 | CCDC115 | 0.1105 |
| ENSP00000368931 | CCDC112 | 0.1105 |
| ENSP00000361556 | C9orf50 | 0.1105 |
| ENSP00000348302 | C3orf27 | 0.1105 |
| ENSP00000225805 | C17orf75 | 0.1105 |
| ENSP00000371829 | BPY2B | 0.1105 |
| ENSP00000382673 | FAM205B | 0.1105 |
| ENSP00000385643 | POM121L1P | 0.1105 |
| ENSP00000353058 | ZNF517 | 0.1079 |
| ENSP00000411032 | ZNF284 | 0.1079 |
| ENSP00000331626 | ZKSCAN2 | 0.1079 |
| ENSP00000328016 | TNFAIP8L3 | 0.1079 |
| ENSP00000364502 | TMEM255B | 0.1079 |
| ENSP00000388431 | TMEM251 | 0.1079 |
| ENSP00000290871 | TEPP | 0.1079 |
| ENSP00000337461 | STPG1 | 0.1079 |
| ENSP00000297819 | SSMEM1 | 0.1079 |
| ENSP00000411734 | SRXN1 | 0.1079 |
| ENSP00000322640 | SPATA31E1 | 0.1079 |
| ENSP00000347153 | SPATA31A7 | 0.1079 |
| ENSP00000329825 | SPATA31A6 | 0.1079 |
| ENSP00000366847 | SPATA31A5 | 0.1079 |
| ENSP00000406957 | SPATA31A2 | 0.1079 |
| ENSP00000437635 | SLFN12L | 0.1079 |
| ENSP00000315214 | RP11-429E11.3 | 0.1079 |

| | | |
|---|---|---|
| ENSP00000457748 | RGSL1 | 0.1079 |
| ENSP00000416033 | PROB1 | 0.1079 |
| ENSP00000235347 | PRAMEF10 | 0.1079 |
| ENSP00000164640 | PDZD4 | 0.1079 |
| ENSP00000368986 | OR6C65 | 0.1079 |
| ENSP00000327540 | OR51B2 | 0.1079 |
| ENSP00000369731 | OR51A4 | 0.1079 |
| ENSP00000316284 | OR2Z1 | 0.1079 |
| ENSP00000329210 | OR2T10 | 0.1079 |
| ENSP00000355430 | OR2T1 | 0.1079 |
| ENSP00000305055 | OR2K2 | 0.1079 |
| ENSP00000307447 | OR2AG1 | 0.1079 |
| ENSP00000386208 | OR2A5 | 0.1079 |
| ENSP00000366352 | OR11A1 | 0.1079 |
| ENSP00000445383 | OR10G2 | 0.1079 |
| ENSP00000360255 | OPN5 | 0.1079 |
| ENSP00000382804 | ODF3B | 0.1079 |
| ENSP00000342411 | NRN1L | 0.1079 |
| ENSP00000358372 | NBPF8 | 0.1079 |
| ENSP00000443339 | MTRNR2L3 | 0.1079 |
| ENSP00000351363 | MSMB | 0.1079 |
| ENSP00000383578 | MGC39584 | 0.1079 |
| ENSP00000383851 | KIAA1522 | 0.1079 |
| ENSP00000242315 | KIAA1045 | 0.1079 |
| ENSP00000331385 | KIAA0825 | 0.1079 |
| ENSP00000299505 | KIAA0355 | 0.1079 |
| ENSP00000315386 | KDELC2 | 0.1079 |
| ENSP00000438895 | IZUMO3 | 0.1079 |
| ENSP00000344430 | IFITM10 | 0.1079 |
| ENSP00000192314 | GAL3ST2 | 0.1079 |
| ENSP00000360379 | FAM209A | 0.1079 |
| ENSP00000262109 | ERICH1 | 0.1079 |
| ENSP00000313226 | CTXN1 | 0.1079 |
| ENSP00000391409 | CDHR4 | 0.1079 |
| ENSP00000359403 | CD99L2 | 0.1079 |
| ENSP00000361599 | CCDC23 | 0.1079 |
| ENSP00000457511 | CCDC179 | 0.1079 |
| ENSP00000267406 | CBLN3 | 0.1079 |
| ENSP00000382561 | C8orf22 | 0.1079 |
| ENSP00000315370 | C5orf46 | 0.1079 |
| ENSP00000366203 | C1orf127 | 0.1079 |
| ENSP00000386230 | C19orf73 | 0.1079 |
| ENSP00000463159 | C19orf47 | 0.1079 |
| ENSP00000323686 | C15orf54 | 0.1079 |
| ENSP00000362762 | BAI2 | 0.1079 |
| ENSP00000215906 | ASPHD2 | 0.1079 |
| ENSP00000457780 | AC073043.2 | 0.1079 |
| ENSP00000469046 | AC018445.1 | 0.1079 |
| ENSP00000471878 | AC005606.1 | 0.1079 |
| ENSP00000383517 | AC002321.2 | 0.1079 |
| ENSP00000426996 | ENSG00000178803 | 0.1079 |
| ENSP00000339585 | ZNF449 | 0.1053 |
| ENSP00000243938 | WFDC3 | 0.1053 |

| | | |
|---|---|---|
| ENSP00000275358 | VWDE | 0.1053 |
| ENSP00000381672 | TP53TG3 | 0.1053 |
| ENSP00000375859 | TMEM91 | 0.1053 |
| ENSP00000310110 | TMEM255A | 0.1053 |
| ENSP00000415030 | TMEM171 | 0.1053 |
| ENSP00000303999 | TMEM133 | 0.1053 |
| ENSP00000413163 | TMEM130 | 0.1053 |
| ENSP00000357425 | SMPDL3A | 0.1053 |
| ENSP00000297307 | SLC35G3 | 0.1053 |
| ENSP00000341867 | SH2D6 | 0.1053 |
| ENSP00000426225 | RP11-542P2.1 | 0.1053 |
| ENSP00000406579 | RP11-124D2.6 | 0.1053 |
| ENSP00000295984 | PRRT3 | 0.1053 |
| ENSP00000301698 | PRR25 | 0.1053 |
| ENSP00000235349 | PRAMEF4 | 0.1053 |
| ENSP00000278855 | PLAC1L | 0.1053 |
| ENSP00000330808 | OTOL1 | 0.1053 |
| ENSP00000342836 | OR6C74 | 0.1053 |
| ENSP00000368990 | OR6C1 | 0.1053 |
| ENSP00000341581 | OR52B6 | 0.1053 |
| ENSP00000369738 | OR51T1 | 0.1053 |
| ENSP00000345163 | OR51F1 | 0.1053 |
| ENSP00000386137 | OR2A14 | 0.1053 |
| ENSP00000323895 | OR10D3 | 0.1053 |
| ENSP00000418802 | NBPF11 | 0.1053 |
| ENSP00000293892 | MSLNL | 0.1053 |
| ENSP00000424768 | MSANTD3-TMEFF1 | 0.1053 |
| ENSP00000311862 | MS4A10 | 0.1053 |
| ENSP00000235332 | MIIP | 0.1053 |
| ENSP00000384640 | MGC10955 | 0.1053 |
| ENSP00000337240 | MFSD11 | 0.1053 |
| ENSP00000365261 | LST1 | 0.1053 |
| ENSP00000369702 | LHFPL2 | 0.1053 |
| ENSP00000377576 | KRTAP9-9 | 0.1053 |
| ENSP00000357008 | ITLN2 | 0.1053 |
| ENSP00000472005 | HBCBP | 0.1053 |
| ENSP00000366628 | GPR157 | 0.1053 |
| ENSP00000358397 | FAM72B | 0.1053 |
| ENSP00000383933 | FAM19A5 | 0.1053 |
| ENSP00000368758 | CTXN3 | 0.1053 |
| ENSP00000331784 | C7orf13 | 0.1053 |
| ENSP00000386146 | C6orf226 | 0.1053 |
| ENSP00000296847 | C6orf195 | 0.1053 |
| ENSP00000423422 | C5orf17 | 0.1053 |
| ENSP00000362743 | C2orf72 | 0.1053 |
| ENSP00000370997 | C2orf50 | 0.1053 |
| ENSP00000284881 | C21orf91 | 0.1053 |
| ENSP00000360210 | C20orf85 | 0.1053 |
| ENSP00000335285 | C1QL4 | 0.1053 |
| ENSP00000358095 | C1orf54 | 0.1053 |
| ENSP00000362133 | C1orf122 | 0.1053 |
| ENSP00000399075 | C18orf42 | 0.1053 |
| ENSP00000284245 | C16orf74 | 0.1053 |

| | | |
|---|---|---|
| ENSP00000386169 | C12orf61 | 0.1053 |
| ENSP00000447057 | C12orf45 | 0.1053 |
| ENSP00000417246 | C10orf128 | 0.1053 |
| ENSP00000328631 | ARMCX2 | 0.1053 |
| ENSP00000319412 | ARMC10 | 0.1053 |
| ENSP00000249116 | APOBEC3A | 0.1053 |
| ENSP00000299415 | AC007431.1 | 0.1053 |
| ENSP00000391835 | FLJ00096 | 0.1053 |
| ENSP00000341236 | ZNF695 | 0.1026 |
| ENSP00000373278 | ZCWPW2 | 0.1026 |
| ENSP00000311343 | WDR49 | 0.1026 |
| ENSP00000357080 | VSIG8 | 0.1026 |
| ENSP00000298530 | TMEM52B | 0.1026 |
| ENSP00000456333 | TMEM210 | 0.1026 |
| ENSP00000342169 | TEX9 | 0.1026 |
| ENSP00000265993 | TCTN3 | 0.1026 |
| ENSP00000324551 | SSUH2 | 0.1026 |
| ENSP00000424058 | SPDYE2L | 0.1026 |
| ENSP00000366731 | SPATA31A4 | 0.1026 |
| ENSP00000454370 | SMKR1 | 0.1026 |
| ENSP00000264852 | SIDT1 | 0.1026 |
| ENSP00000435188 | RP11-831H9.11 | 0.1026 |
| ENSP00000362230 | RP11-268J15.5 | 0.1026 |
| ENSP00000382030 | RAD51AP2 | 0.1026 |
| ENSP00000319140 | PSG11 | 0.1026 |
| ENSP00000371271 | PRH2 | 0.1026 |
| ENSP00000328422 | PP13439 | 0.1026 |
| ENSP00000331643 | PGBD2 | 0.1026 |
| ENSP00000263174 | PALMD | 0.1026 |
| ENSP00000323606 | OR6C2 | 0.1026 |
| ENSP00000323354 | OR5M8 | 0.1026 |
| ENSP00000373194 | OR5K3 | 0.1026 |
| ENSP00000321506 | OR4X1 | 0.1026 |
| ENSP00000305424 | OR1F1 | 0.1026 |
| ENSP00000354707 | OR10Z1 | 0.1026 |
| ENSP00000335596 | OR10H1 | 0.1026 |
| ENSP00000372724 | LY6G5C | 0.1026 |
| ENSP00000297423 | KIAA0146 | 0.1026 |
| ENSP00000336861 | IQCH | 0.1026 |
| ENSP00000453364 | HCG27 | 0.1026 |
| ENSP00000401310 | FRG2B | 0.1026 |
| ENSP00000469038 | FLJ14816 | 0.1026 |
| ENSP00000392909 | FLJ00104 | 0.1026 |
| ENSP00000321962 | FIBIN | 0.1026 |
| ENSP00000351632 | FAM3D | 0.1026 |
| ENSP00000357892 | FAM24B | 0.1026 |
| ENSP00000417581 | FAM199X | 0.1026 |
| ENSP00000377807 | FAM122A | 0.1026 |
| ENSP00000420075 | EFCC1 | 0.1026 |
| ENSP00000420854 | EFCAB12 | 0.1026 |
| ENSP00000331479 | CYB5D1 | 0.1026 |
| ENSP00000470478 | CTD-3088G3.8 | 0.1026 |
| ENSP00000308279 | C9orf131 | 0.1026 |

| | | |
|---|---|---|
| ENSP00000454153 | C5orf63 | 0.1026 |
| ENSP00000332875 | C2orf70 | 0.1026 |
| ENSP00000380920 | C19orf38 | 0.1026 |
| ENSP00000267485 | C14orf37 | 0.1026 |
| ENSP00000278601 | C11orf52 | 0.1026 |
| ENSP00000268314 | ARMC5 | 0.1026 |
| ENSP00000354991 | ALPK2 | 0.1026 |
| ENSP00000472412 | AC187652.1 | 0.1026 |
| ENSP00000471653 | AC018692.2 | 0.1026 |
| ENSP00000396627 | FAM157A | 0.1026 |
| ENSP00000403609 | ENSG00000240215 | 0.1026 |
| ENSP00000468311 | MIR7-3HG | 0.1026 |
| ENSP00000361598 | ZSWIM1 | 0.1000 |
| ENSP00000353699 | WDR72 | 0.1000 |
| ENSP00000352172 | VSIG10 | 0.1000 |
| ENSP00000311760 | TTC9B | 0.1000 |
| ENSP00000253934 | TMEM204 | 0.1000 |
| ENSP00000320757 | TMEM150B | 0.1000 |
| ENSP00000441459 | SDIM1 | 0.1000 |
| ENSP00000464803 | RP11-795F19.5 | 0.1000 |
| ENSP00000437523 | RP11-571M6.6 | 0.1000 |
| ENSP00000344996 | PALM3 | 0.1000 |
| ENSP00000307734 | OR6Q1 | 0.1000 |
| ENSP00000321196 | OR56B4 | 0.1000 |
| ENSP00000404102 | OR2V1 | 0.1000 |
| ENSP00000324687 | OR2T33 | 0.1000 |
| ENSP00000331774 | OR2T29 | 0.1000 |
| ENSP00000299459 | OR2D2 | 0.1000 |
| ENSP00000209540 | OR1I1 | 0.1000 |
| ENSP00000289451 | OR10K1 | 0.1000 |
| ENSP00000333593 | NXPH4 | 0.1000 |
| ENSP00000327718 | MYADML2 | 0.1000 |
| ENSP00000274897 | MLIP | 0.1000 |
| ENSP00000280082 | MIA2 | 0.1000 |
| ENSP00000414517 | MAMLD1 | 0.1000 |
| ENSP00000334463 | LYPD6 | 0.1000 |
| ENSP00000384670 | KIAA1210 | 0.1000 |
| ENSP00000410360 | KIAA0125 | 0.1000 |
| ENSP00000293405 | IZUMO2 | 0.1000 |
| ENSP00000416095 | INE1 | 0.1000 |
| ENSP00000384183 | GATSL3 | 0.1000 |
| ENSP00000354869 | GATSL1 | 0.1000 |
| ENSP00000456337 | FLJ27352 | 0.1000 |
| ENSP00000472934 | FKSG61 | 0.1000 |
| ENSP00000360376 | FAM209B | 0.1000 |
| ENSP00000417711 | FAM205A | 0.1000 |
| ENSP00000329995 | FAM186A | 0.1000 |
| ENSP00000367356 | FAM171A1 | 0.1000 |
| ENSP00000405222 | DCAF8L1 | 0.1000 |
| ENSP00000299340 | CYYR1 | 0.1000 |
| ENSP00000410400 | CT47A11 | 0.1000 |
| ENSP00000390661 | CLEC2L | 0.1000 |
| ENSP00000294600 | CCDC27 | 0.1000 |

| | | |
|---|---|---|
| ENSP00000357079 | CCDC19 | 0.1000 |
| ENSP00000315614 | C8orf47 | 0.1000 |
| ENSP00000338228 | C5orf56 | 0.1000 |
| ENSP00000330426 | C1QTNF8 | 0.1000 |
| ENSP00000360824 | C1orf185 | 0.1000 |
| ENSP00000355241 | C19orf45 | 0.1000 |
| ENSP00000386557 | C19orf24 | 0.1000 |
| ENSP00000321519 | C19orf18 | 0.1000 |
| ENSP00000341412 | C11orf34 | 0.1000 |
| ENSP00000361199 | C10orf99 | 0.1000 |
| ENSP00000303710 | C10orf68 | 0.1000 |
| ENSP00000339802 | ANKRD34B | 0.1000 |
| ENSP00000469960 | AL138815.2 | 0.1000 |
| ENSP00000320396 | AL033381.1 | 0.1000 |
| ENSP00000471898 | AC099780.1 | 0.1000 |
| ENSP00000322899 | ZNF843 | 0.0974 |
| ENSP00000328397 | VMO1 | 0.0974 |
| ENSP00000262074 | TTC23 | 0.0974 |
| ENSP00000433816 | TSPAN19 | 0.0974 |
| ENSP00000356135 | TMEM81 | 0.0974 |
| ENSP00000361355 | TMEM254 | 0.0974 |
| ENSP00000331965 | TLCD2 | 0.0974 |
| ENSP00000363345 | SLC46A2 | 0.0974 |
| ENSP00000355064 | SHISA4 | 0.0974 |
| ENSP00000297354 | SBSPON | 0.0974 |
| ENSP00000349931 | PRAMEF8 | 0.0974 |
| ENSP00000310632 | OR8S1 | 0.0974 |
| ENSP00000325078 | OR2AJ1 | 0.0974 |
| ENSP00000342697 | OR2AG2 | 0.0974 |
| ENSP00000302199 | OR10V1 | 0.0974 |
| ENSP00000314324 | OR10Q1 | 0.0974 |
| ENSP00000239614 | MSANTD2 | 0.0974 |
| ENSP00000278865 | MS4A3 | 0.0974 |
| ENSP00000382476 | MROH2B | 0.0974 |
| ENSP00000366387 | MAMDC2 | 0.0974 |
| ENSP00000406478 | LILRB5 | 0.0974 |
| ENSP00000245620 | LILRB3 | 0.0974 |
| ENSP00000251390 | LILRA3 | 0.0974 |
| ENSP00000251377 | LILRA2 | 0.0974 |
| ENSP00000251372 | LILRA1 | 0.0974 |
| ENSP00000246070 | LAMP5 | 0.0974 |
| ENSP00000339356 | KLRG2 | 0.0974 |
| ENSP00000402153 | IQCJ | 0.0974 |
| ENSP00000442291 | IQCC | 0.0974 |
| ENSP00000472367 | HUG1 | 0.0974 |
| ENSP00000464833 | HSH2D | 0.0974 |
| ENSP00000289448 | HMHB1 | 0.0974 |
| ENSP00000415299 | FAM221B | 0.0974 |
| ENSP00000366582 | FAM196B | 0.0974 |
| ENSP00000372290 | FAM193A | 0.0974 |
| ENSP00000437812 | FAM106A | 0.0974 |
| ENSP00000334501 | DMRTC1B | 0.0974 |
| ENSP00000324025 | CGREF1 | 0.0974 |

| | | |
|---|---|---|
| ENSP00000376522 | CEP112 | 0.0974 |
| ENSP00000280245 | CCDC83 | 0.0974 |
| ENSP00000259870 | C6orf15 | 0.0974 |
| ENSP00000349669 | C5orf48 | 0.0974 |
| ENSP00000402915 | C2orf74 | 0.0974 |
| ENSP00000298943 | C1QL3 | 0.0974 |
| ENSP00000357082 | C1orf204 | 0.0974 |
| ENSP00000345102 | C19orf35 | 0.0974 |
| ENSP00000386452 | C17orf104 | 0.0974 |
| ENSP00000260276 | C11orf1 | 0.0974 |
| ENSP00000404304 | ARMCX4 | 0.0974 |
| ENSP00000386791 | AP000322.54 | 0.0974 |
| ENSP00000381711 | AL162389.1 | 0.0974 |
| ENSP00000472121 | AL136376.1 | 0.0974 |
| ENSP00000387081 | AC140481.2 | 0.0974 |
| ENSP00000386773 | AC013269.5 | 0.0974 |
| ENSP00000469669 | AC002985.3 | 0.0974 |
| ENSP00000329365 | ZNF730 | 0.0974 |
| ENSP00000372122 | C18orf63 | 0.0974 |
| ENSP00000322265 | ZNF620 | 0.0947 |
| ENSP00000356674 | ZBTB37 | 0.0947 |
| ENSP00000364720 | XAGE1E | 0.0947 |
| ENSP00000364240 | UBXN10 | 0.0947 |
| ENSP00000317595 | TPPP2 | 0.0947 |
| ENSP00000369840 | TEX26 | 0.0947 |
| ENSP00000355797 | TCP10L2 | 0.0947 |
| ENSP00000335392 | SPATA12 | 0.0947 |
| ENSP00000229570 | SMIM8 | 0.0947 |
| ENSP00000320430 | S100Z | 0.0947 |
| ENSP00000455300 | RP1-27O5.3 | 0.0947 |
| ENSP00000311398 | RNASE8 | 0.0947 |
| ENSP00000471531 | PRED57 | 0.0947 |
| ENSP00000365343 | PRAMEF3 | 0.0947 |
| ENSP00000441685 | PDZD9 | 0.0947 |
| ENSP00000346611 | OR8B3 | 0.0947 |
| ENSP00000364152 | OR8B2 | 0.0947 |
| ENSP00000304807 | OR6Y1 | 0.0947 |
| ENSP00000357127 | OR6K3 | 0.0947 |
| ENSP00000444054 | OR52E8 | 0.0947 |
| ENSP00000333196 | OR51M1 | 0.0947 |
| ENSP00000409316 | OR4F29 | 0.0947 |
| ENSP00000328934 | OR2T11 | 0.0947 |
| ENSP00000366374 | OR2J3 | 0.0947 |
| ENSP00000366372 | OR2J2 | 0.0947 |
| ENSP00000297913 | OR1Q1 | 0.0947 |
| ENSP00000306974 | OR1N1 | 0.0947 |
| ENSP00000373047 | OR12D2 | 0.0947 |
| ENSP00000333900 | OIT3 | 0.0947 |
| ENSP00000472384 | MIA | 0.0947 |
| ENSP00000291759 | LILRA4 | 0.0947 |
| ENSP00000301202 | LAIR2 | 0.0947 |
| ENSP00000366984 | KRT40 | 0.0947 |
| ENSP00000264735 | HRASLS | 0.0947 |

| | | |
|---|---|---|
| ENSP00000361662 | GUCA2B | 0.0947 |
| ENSP00000302833 | GIMAP1 | 0.0947 |
| ENSP00000360757 | FAM69B | 0.0947 |
| ENSP00000409423 | FAM47E | 0.0947 |
| ENSP00000265299 | FAM188B | 0.0947 |
| ENSP00000257894 | FAM186B | 0.0947 |
| ENSP00000398979 | CYB561A3 | 0.0947 |
| ENSP00000353013 | CLEC14A | 0.0947 |
| ENSP00000360381 | CDCP2 | 0.0947 |
| ENSP00000333915 | CCDC42B | 0.0947 |
| ENSP00000365169 | C9orf153 | 0.0947 |
| ENSP00000289989 | C8orf58 | 0.0947 |
| ENSP00000378601 | C8orf31 | 0.0947 |
| ENSP00000322582 | C4orf17 | 0.0947 |
| ENSP00000395549 | C22orf34 | 0.0947 |
| ENSP00000358965 | C1orf194 | 0.0947 |
| ENSP00000311390 | C16orf91 | 0.0947 |
| ENSP00000324672 | C16orf89 | 0.0947 |
| ENSP00000316898 | C12orf54 | 0.0947 |
| ENSP00000472951 | AC138517.1 | 0.0947 |
| ENSP00000472801 | AC007204.1 | 0.0947 |
| ENSP00000364382 | AAED1 | 0.0947 |
| ENSP00000409652 | ZNF766 | 0.0921 |
| ENSP00000347210 | ZNF736 | 0.0921 |
| ENSP00000447987 | ZNF727 | 0.0921 |
| ENSP00000309330 | ZNF680 | 0.0921 |
| ENSP00000318753 | WFDC11 | 0.0921 |
| ENSP00000351244 | VN1R2 | 0.0921 |
| ENSP00000368007 | TMEM240 | 0.0921 |
| ENSP00000350050 | TMEM229B | 0.0921 |
| ENSP00000342162 | TMEM221 | 0.0921 |
| ENSP00000372192 | TMEM211 | 0.0921 |
| ENSP00000295658 | TMEM169 | 0.0921 |
| ENSP00000444736 | TAS2R30 | 0.0921 |
| ENSP00000441624 | TAS2R20 | 0.0921 |
| ENSP00000364363 | SMIM5 | 0.0921 |
| ENSP00000462106 | SMIM21 | 0.0921 |
| ENSP00000405610 | S100A7L2 | 0.0921 |
| ENSP00000287143 | PRG3 | 0.0921 |
| ENSP00000472225 | PRED58 | 0.0921 |
| ENSP00000410024 | PHGR1 | 0.0921 |
| ENSP00000397881 | PET117 | 0.0921 |
| ENSP00000303469 | OR52A5 | 0.0921 |
| ENSP00000322156 | OR51L1 | 0.0921 |
| ENSP00000321447 | OR4S1 | 0.0921 |
| ENSP00000325065 | OR4A15 | 0.0921 |
| ENSP00000366376 | OR2J1 | 0.0921 |
| ENSP00000376633 | OR2F1 | 0.0921 |
| ENSP00000420502 | OR2A7 | 0.0921 |
| ENSP00000334452 | OR13F1 | 0.0921 |
| ENSP00000259362 | OR13C9 | 0.0921 |
| ENSP00000438815 | OR13C2 | 0.0921 |
| ENSP00000449002 | OR11H12 | 0.0921 |

| | | |
|---|---|---|
| ENSP00000312470 | OR10A6 | 0.0921 |
| ENSP00000470526 | MGC4771 | 0.0921 |
| ENSP00000357602 | LRRC27 | 0.0921 |
| ENSP00000301219 | LILRA5 | 0.0921 |
| ENSP00000359498 | FMR1NB | 0.0921 |
| ENSP00000242505 | FAM149B1 | 0.0921 |
| ENSP00000403396 | FAM126A | 0.0921 |
| ENSP00000312767 | CTD-2373H9.6 | 0.0921 |
| ENSP00000262932 | CNPY4 | 0.0921 |
| ENSP00000373948 | C7orf63 | 0.0921 |
| ENSP00000364784 | C6orf48 | 0.0921 |
| ENSP00000455385 | C3orf83 | 0.0921 |
| ENSP00000294889 | C1orf115 | 0.0921 |
| ENSP00000440920 | C14orf132 | 0.0921 |
| ENSP00000359782 | BEND6 | 0.0921 |
| ENSP00000381780 | ARHGEF33 | 0.0921 |
| ENSP00000445063 | AC105052.1 | 0.0921 |
| ENSP00000443070 | LINC00273 | 0.0921 |
| ENSP00000339830 | TMEM220 | 0.0895 |
| ENSP00000457962 | TGFBR3L | 0.0895 |
| ENSP00000334668 | SAMD7 | 0.0895 |
| ENSP00000451258 | RP11-293M10.1 | 0.0895 |
| ENSP00000313110 | OR6S1 | 0.0895 |
| ENSP00000302057 | OR5AU1 | 0.0895 |
| ENSP00000307751 | OR4X2 | 0.0895 |
| ENSP00000327525 | OR4F6 | 0.0895 |
| ENSP00000311605 | OR4B1 | 0.0895 |
| ENSP00000412752 | OR4A47 | 0.0895 |
| ENSP00000334068 | OR13C8 | 0.0895 |
| ENSP00000326718 | OR10A7 | 0.0895 |
| ENSP00000470694 | MGC4294 | 0.0895 |
| ENSP00000470117 | LUZPP1 | 0.0895 |
| ENSP00000266031 | HYAL1 | 0.0895 |
| ENSP00000377980 | HEPACAM2 | 0.0895 |
| ENSP00000317743 | HARBI1 | 0.0895 |
| ENSP00000365057 | GPANK1 | 0.0895 |
| ENSP00000295569 | FAM19A4 | 0.0895 |
| ENSP00000429896 | FAM178B | 0.0895 |
| ENSP00000340138 | DLEU2L | 0.0895 |
| ENSP00000367490 | DEPDC4 | 0.0895 |
| ENSP00000246020 | CSTL1 | 0.0895 |
| ENSP00000222902 | CCL24 | 0.0895 |
| ENSP00000472120 | CATX-2 | 0.0895 |
| ENSP00000318395 | C5orf64 | 0.0895 |
| ENSP00000408527 | C2orf61 | 0.0895 |
| ENSP00000327764 | C22orf26 | 0.0895 |
| ENSP00000272520 | C1QL2 | 0.0895 |
| ENSP00000437532 | C16orf93 | 0.0895 |
| ENSP00000386487 | C14orf180 | 0.0895 |
| ENSP00000229281 | C12orf57 | 0.0895 |
| ENSP00000367449 | C10orf111 | 0.0895 |
| ENSP00000361917 | ARMCX1 | 0.0895 |
| ENSP00000371968 | AC074389.6 | 0.0895 |

| | | |
|---|---|---|
| ENSP00000469964 | AC007461.1 | 0.0895 |
| ENSP00000332404 | TMIGD1 | 0.0868 |
| ENSP00000419712 | TMEM8C | 0.0868 |
| ENSP00000371318 | TMEM74B | 0.0868 |
| ENSP00000446514 | TMEM235 | 0.0868 |
| ENSP00000329794 | TCEAL7 | 0.0868 |
| ENSP00000367911 | SYCE1L | 0.0868 |
| ENSP00000304762 | SPACA5B | 0.0868 |
| ENSP00000372410 | RNASE13 | 0.0868 |
| ENSP00000306657 | OR5B12 | 0.0868 |
| ENSP00000362792 | OR1N2 | 0.0868 |
| ENSP00000322295 | OR10AB1P | 0.0868 |
| ENSP00000419194 | MSMP | 0.0868 |
| ENSP00000315626 | FAM101A | 0.0868 |
| ENSP00000335325 | CCDC73 | 0.0868 |
| ENSP00000258214 | CCDC102A | 0.0868 |
| ENSP00000360191 | APCDD1L | 0.0868 |
| ENSP00000472724 | AC003043.1 | 0.0868 |
| ENSP00000447931 | ZNF878 | 0.0842 |
| ENSP00000419397 | ZNF782 | 0.0842 |
| ENSP00000471593 | ZNF728 | 0.0842 |
| ENSP00000379458 | ZNF586 | 0.0842 |
| ENSP00000313443 | ZNF491 | 0.0842 |
| ENSP00000335042 | ZNF486 | 0.0842 |
| ENSP00000403130 | TMEM233 | 0.0842 |
| ENSP00000312672 | TMEM136 | 0.0842 |
| ENSP00000361765 | TCEAL5 | 0.0842 |
| ENSP00000341988 | SPATA31D1 | 0.0842 |
| ENSP00000305847 | SPACA5 | 0.0842 |
| ENSP00000328335 | SMIM10 | 0.0842 |
| ENSP00000465978 | RP11-477N12.3 | 0.0842 |
| ENSP00000365266 | PRAMEF17 | 0.0842 |
| ENSP00000365289 | PRAMEF16 | 0.0842 |
| ENSP00000341333 | PRAMEF14 | 0.0842 |
| ENSP00000308714 | OR9Q2 | 0.0842 |
| ENSP00000302606 | OR9I1 | 0.0842 |
| ENSP00000348449 | OR8B4 | 0.0842 |
| ENSP00000335535 | OR6N1 | 0.0842 |
| ENSP00000311038 | OR6M1 | 0.0842 |
| ENSP00000357126 | OR6K6 | 0.0842 |
| ENSP00000448811 | OR6C68 | 0.0842 |
| ENSP00000279791 | OR5M9 | 0.0842 |
| ENSP00000312208 | OR5M3 | 0.0842 |
| ENSP00000335529 | OR5L1 | 0.0842 |
| ENSP00000320302 | OR5AN1 | 0.0842 |
| ENSP00000328878 | OR52E6 | 0.0842 |
| ENSP00000332473 | OR51J1 | 0.0842 |
| ENSP00000369559 | OR51I1 | 0.0842 |
| ENSP00000322593 | OR51G2 | 0.0842 |
| ENSP00000350222 | OR51D1 | 0.0842 |
| ENSP00000352305 | OR51A7 | 0.0842 |
| ENSP00000319217 | OR4L1 | 0.0842 |
| ENSP00000304077 | OR4K15 | 0.0842 |

| | | |
|---|---|---|
| ENSP00000305970 | OR4D5 | 0.0842 |
| ENSP00000330904 | OR2T34 | 0.0842 |
| ENSP00000323423 | OR2AP1 | 0.0842 |
| ENSP00000306607 | OR1L8 | 0.0842 |
| ENSP00000259466 | OR1L4 | 0.0842 |
| ENSP00000318834 | OR10H4 | 0.0842 |
| ENSP00000389072 | OR10G8 | 0.0842 |
| ENSP00000358228 | NBPF24 | 0.0842 |
| ENSP00000356733 | MROH9 | 0.0842 |
| ENSP00000381857 | MROH6 | 0.0842 |
| ENSP00000319388 | MAMDC4 | 0.0842 |
| ENSP00000389760 | GATS | 0.0842 |
| ENSP00000363529 | FAM83C | 0.0842 |
| ENSP00000273146 | FAM198A | 0.0842 |
| ENSP00000420174 | ERVFRD-1 | 0.0842 |
| ENSP00000269720 | CTB-55O6.8 | 0.0842 |
| ENSP00000421868 | CCDC169-SOHLH2 | 0.0842 |
| ENSP00000360682 | C9orf142 | 0.0842 |
| ENSP00000335500 | C7orf57 | 0.0842 |
| ENSP00000368623 | C1orf159 | 0.0842 |
| ENSP00000382318 | C14orf23 | 0.0842 |
| ENSP00000386960 | C10orf55 | 0.0842 |
| ENSP00000394705 | AL353791.1 | 0.0842 |
| ENSP00000472130 | AL161784.1 | 0.0842 |
| ENSP00000469538 | AC104057.1 | 0.0842 |
| ENSP00000469305 | AC093323.1 | 0.0842 |
| ENSP00000349693 | ENSG00000197426 | 0.0842 |
| ENSP00000374049 | VWA3A | 0.0816 |
| ENSP00000256362 | VRTN | 0.0816 |
| ENSP00000257637 | TMEM243 | 0.0816 |
| ENSP00000428459 | TMEM200B | 0.0816 |
| ENSP00000380858 | ST7-OT4 | 0.0816 |
| ENSP00000469270 | SBP1 | 0.0816 |
| ENSP00000465021 | RP11-127I20.4 | 0.0816 |
| ENSP00000306324 | PWWP2B | 0.0816 |
| ENSP00000328768 | PRR23B | 0.0816 |
| ENSP00000347211 | PRAMEF6 | 0.0816 |
| ENSP00000365302 | PRAMEF13 | 0.0816 |
| ENSP00000334934 | OR9Q1 | 0.0816 |
| ENSP00000307598 | OR9K2 | 0.0816 |
| ENSP00000377799 | OR6C4 | 0.0816 |
| ENSP00000373195 | OR5H15 | 0.0816 |
| ENSP00000362784 | OR5C1 | 0.0816 |
| ENSP00000319197 | OR4K17 | 0.0816 |
| ENSP00000347428 | OR2L5 | 0.0816 |
| ENSP00000449598 | MYRFL | 0.0816 |
| ENSP00000330612 | MRGPRG | 0.0816 |
| ENSP00000305138 | FAM195A | 0.0816 |
| ENSP00000395340 | FAM185A | 0.0816 |
| ENSP00000419539 | CTAGE4 | 0.0816 |
| ENSP00000424711 | CLRN2 | 0.0816 |
| ENSP00000469474 | CCDC177 | 0.0816 |
| ENSP00000386450 | C7orf34 | 0.0816 |

| | | |
|---|---|---|
| ENSP00000347322 | C6orf89 | 0.0816 |
| ENSP00000372651 | C3orf72 | 0.0816 |
| ENSP00000471714 | AC068620.1 | 0.0816 |
| ENSP00000302657 | ABHD15 | 0.0816 |
| ENSP00000301665 | TMEM99 | 0.0789 |
| ENSP00000279396 | TMEM219 | 0.0789 |
| ENSP00000383270 | SMIM2 | 0.0789 |
| ENSP00000323928 | OR8H3 | 0.0789 |
| ENSP00000437629 | OR6J1 | 0.0789 |
| ENSP00000401706 | OR5H14 | 0.0789 |
| ENSP00000346575 | OR5H1 | 0.0789 |
| ENSP00000335025 | OR5D18 | 0.0789 |
| ENSP00000348368 | OR52R1 | 0.0789 |
| ENSP00000386180 | OR3A2 | 0.0789 |
| ENSP00000352604 | OR2T3 | 0.0789 |
| ENSP00000326301 | OR2G3 | 0.0789 |
| ENSP00000299454 | OR10A5 | 0.0789 |
| ENSP00000300226 | MS4A8 | 0.0789 |
| ENSP00000359404 | KIAA1107 | 0.0789 |
| ENSP00000338532 | FAM53B | 0.0789 |
| ENSP00000357588 | FAM26E | 0.0789 |
| ENSP00000407749 | FAM25G | 0.0789 |
| ENSP00000464272 | FAM25E | 0.0789 |
| ENSP00000341481 | FAM25C | 0.0789 |
| ENSP00000413896 | FAM25B | 0.0789 |
| ENSP00000342790 | FAM25A | 0.0789 |
| ENSP00000342336 | FAM180A | 0.0789 |
| ENSP00000297203 | C7orf62 | 0.0789 |
| ENSP00000373575 | C6orf163 | 0.0789 |
| ENSP00000330267 | C15orf32 | 0.0789 |
| ENSP00000459753 | AC027763.2 | 0.0789 |
| ENSP00000380480 | ZNF891 | 0.0763 |
| ENSP00000410545 | ZNF814 | 0.0763 |
| ENSP00000395629 | ZNF812 | 0.0763 |
| ENSP00000342021 | ZNF788 | 0.0763 |
| ENSP00000473243 | ZNF788 | 0.0763 |
| ENSP00000394248 | ZNF716 | 0.0763 |
| ENSP00000411132 | ZNF619 | 0.0763 |
| ENSP00000465960 | ZNF585B | 0.0763 |
| ENSP00000472277 | ZNF548 | 0.0763 |
| ENSP00000282282 | ZNF547 | 0.0763 |
| ENSP00000468271 | ZFP112 | 0.0763 |
| ENSP00000311122 | TMEM52 | 0.0763 |
| ENSP00000380671 | SMCO1 | 0.0763 |
| ENSP00000456213 | RP4-734P14.4 | 0.0763 |
| ENSP00000454561 | RP11-122A3.2 | 0.0763 |
| ENSP00000353537 | OR5B21 | 0.0763 |
| ENSP00000334418 | OR4C12 | 0.0763 |
| ENSP00000324583 | OR2T12 | 0.0763 |
| ENSP00000313833 | LCN15 | 0.0763 |
| ENSP00000373254 | KRBOX1 | 0.0763 |
| ENSP00000359978 | HHLA3 | 0.0763 |
| ENSP00000468743 | CTD-3116E22.4 | 0.0763 |

| | | |
|---|---|---|
| ENSP00000471607 | CTD-3105H18.18 | 0.0763 |
| ENSP00000471613 | CTD-3105H18.16 | 0.0763 |
| ENSP00000473166 | CTD-2561J22.3 | 0.0763 |
| ENSP00000473043 | CTD-2192J16.20 | 0.0763 |
| ENSP00000466387 | CTC-398G3.6 | 0.0763 |
| ENSP00000455948 | CLEC19A | 0.0763 |
| ENSP00000342445 | CLDN4 | 0.0763 |
| ENSP00000369431 | CAAP1 | 0.0763 |
| ENSP00000450114 | C14orf79 | 0.0763 |
| ENSP00000357356 | AL603926.1 | 0.0763 |
| ENSP00000441995 | AC073343.1 | 0.0763 |
| ENSP00000472160 | AC003006.7 | 0.0763 |
| ENSP00000321812 | ZNF776 | 0.0737 |
| ENSP00000472894 | ZNF547 | 0.0737 |
| ENSP00000237275 | ZC2HC1B | 0.0737 |
| ENSP00000343366 | VSTM1 | 0.0737 |
| ENSP00000301057 | TP53I13 | 0.0737 |
| ENSP00000421652 | RP11-1396O13.13 | 0.0737 |
| ENSP00000275275 | PNLDC1 | 0.0737 |
| ENSP00000448789 | OR9A4 | 0.0737 |
| ENSP00000316518 | OR9A2 | 0.0737 |
| ENSP00000318956 | OR52K2 | 0.0737 |
| ENSP00000302422 | OR52K1 | 0.0737 |
| ENSP00000394047 | CTD-3105H18.14 | 0.0737 |
| ENSP00000458124 | CTD-2006C1.10 | 0.0737 |
| ENSP00000438285 | CCDC177 | 0.0737 |
| ENSP00000343171 | C2orf80 | 0.0737 |
| ENSP00000305875 | C20orf196 | 0.0737 |
| ENSP00000341787 | BTNL3 | 0.0737 |
| ENSP00000471857 | AC096677.1 | 0.0737 |
| ENSP00000455659 | AC002310.13 | 0.0737 |
| ENSP00000383873 | TTC34 | 0.0711 |
| ENSP00000384370 | STEAP1B | 0.0711 |
| ENSP00000463047 | RP11-111H3.1 | 0.0711 |
| ENSP00000375562 | RBM12B-AS1 | 0.0711 |
| ENSP00000419944 | PXT1 | 0.0711 |
| ENSP00000352626 | OR6K2 | 0.0711 |
| ENSP00000399627 | LCN6 | 0.0711 |
| ENSP00000334072 | TMEM212 | 0.0684 |
| ENSP00000432353 | PBOV1 | 0.0684 |
| ENSP00000322546 | OR51G1 | 0.0684 |
| ENSP00000386143 | HEPN1 | 0.0684 |
| ENSP00000383682 | FAM72D | 0.0684 |
| ENSP00000387298 | ERICH2 | 0.0684 |
| ENSP00000367926 | CXorf30 | 0.0684 |
| ENSP00000369963 | C20orf141 | 0.0684 |
| ENSP00000361463 | AL391421.1 | 0.0684 |
| ENSP00000446137 | AL360154.1 | 0.0684 |
| ENSP00000419119 | OR10C1 | 0.0658 |
| ENSP00000393556 | FLJ20373 | 0.0658 |
| ENSP00000382675 | ENHO | 0.0658 |
| ENSP00000355752 | C1orf95 | 0.0658 |
| ENSP00000359401 | C1orf146 | 0.0658 |

| | | |
|---|---|---|
| ENSP00000416549 | AC109135.1 | 0.0658 |
| ENSP00000394653 | IQCF5 | 0.0632 |
| ENSP00000360166 | CXorf64 | 0.0632 |
| ENSP00000382026 | C4orf3 | 0.0632 |
| ENSP00000248984 | C22orf24 | 0.0632 |
| ENSP00000371529 | C22orf42 | 0.0605 |
| ENSP00000384683 | RNASE9 | 0.0553 |
